# Controlling phosphorus(v)-stereogenicity *via* organocatalytic acylative desymmetrisation of bisphenols

**DOI:** 10.1039/d6sc05320c

**Published:** 2026-08-20

**Authors:** Ffion M. Platt, Martha I. Prindl, Zhanyu Zhou, David B. Cordes, Heena Panchal, Andrew D. Smith

**Affiliations:** a EaStCHEM, School of Chemistry, University of St Andrews St Andrews Fife KY16 9ST UK ads10@st-andrews.ac.uk; b Chemical Development, PT&D, AstraZeneca Etherow Building, Silk Road Business Park, Charter Way Macclesfield Cheshire SK10 2NA UK

## Abstract

The enantioselective construction of phosphorus(v)-stereogenic centres is of broad importance due to the utility of these compounds both as ligands in catalysis and as scaffolds in medicinal chemistry. This manuscript demonstrates the effective acylative desymmetrisation of *ortho*-bisphenols to access P(v)-stereogenic products using low 1 mol% loadings of the chiral isothiourea HyperBTM as a Lewis base catalyst and readily available anhydride feedstock starting materials. The scope and limitations of this method have been investigated (24 examples, up to 95% yield, 98 : 2 er). Control experiments reveal the role of *bis*-acylation in the amplification of stereocontrol through a chiroablative kinetic resolution of the monoester product. A chromatography-free process towards the standard starting material has been developed on decagram scale, facilitating the catalytic application of the process on multigram scale, with 98% catalyst recovery. Synthesis of a polymer-supported isothiourea catalyst facilitated transfer of the catalytic reaction into continuous flow in a packed-bed reactor.

## Abstract 2

An effective acylative desymmetrisation of *ortho*-bisphenols to access P(v)-stereogenic products using the isothiourea catalyst HyperBTM has been developed (24 examples, up to 95% yield, 98 : 2 er) and translated to continuous flow.

## Graphical abstract

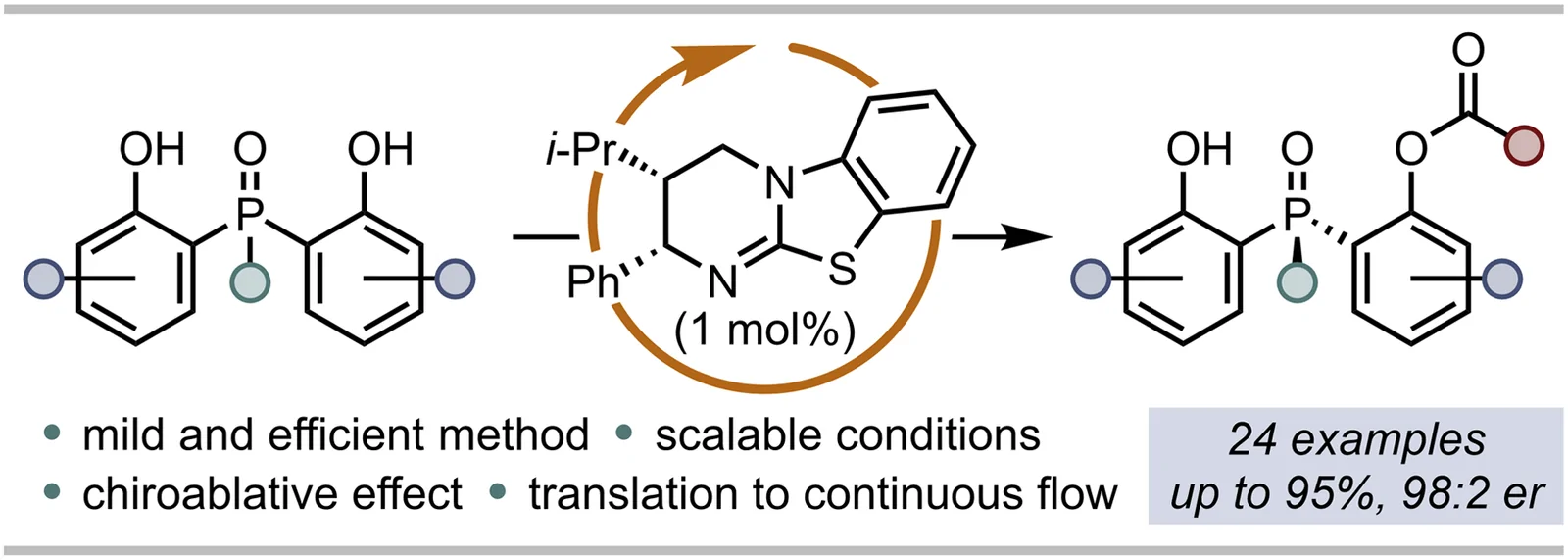

## Introduction

Organophosphorus compounds bearing P(v)-stereogenic centres are of great importance across multiple industries including pharmaceuticals, agrochemicals and chemical synthesis.^1–3^ The utility of these compounds is exemplified by Vemlidy, a Protide prodrug developed by Gilead Sciences for the treatment of HIV and hepatitis B; Zytron, a herbicide developed by Dow for weed control in turf; and (*R*)-PAMPO, which is a precursor to both chiral phosphine ligands (*R*)-DiPAMP and (*R*)-CAMP, which are used in enantioselective catalysis (Fig. 1A).^4,5^ Owing to their importance, the catalytic synthesis of enantiomerically-enriched organophosphorus compounds bearing a P(v)-stereogenic centre has received considerable recent attention, with resolution and desymmetrisation strategies developed in the areas of transition-metal catalysis, organocatalysis and enzymatic catalysis.^6–18^

**Fig. 1:**
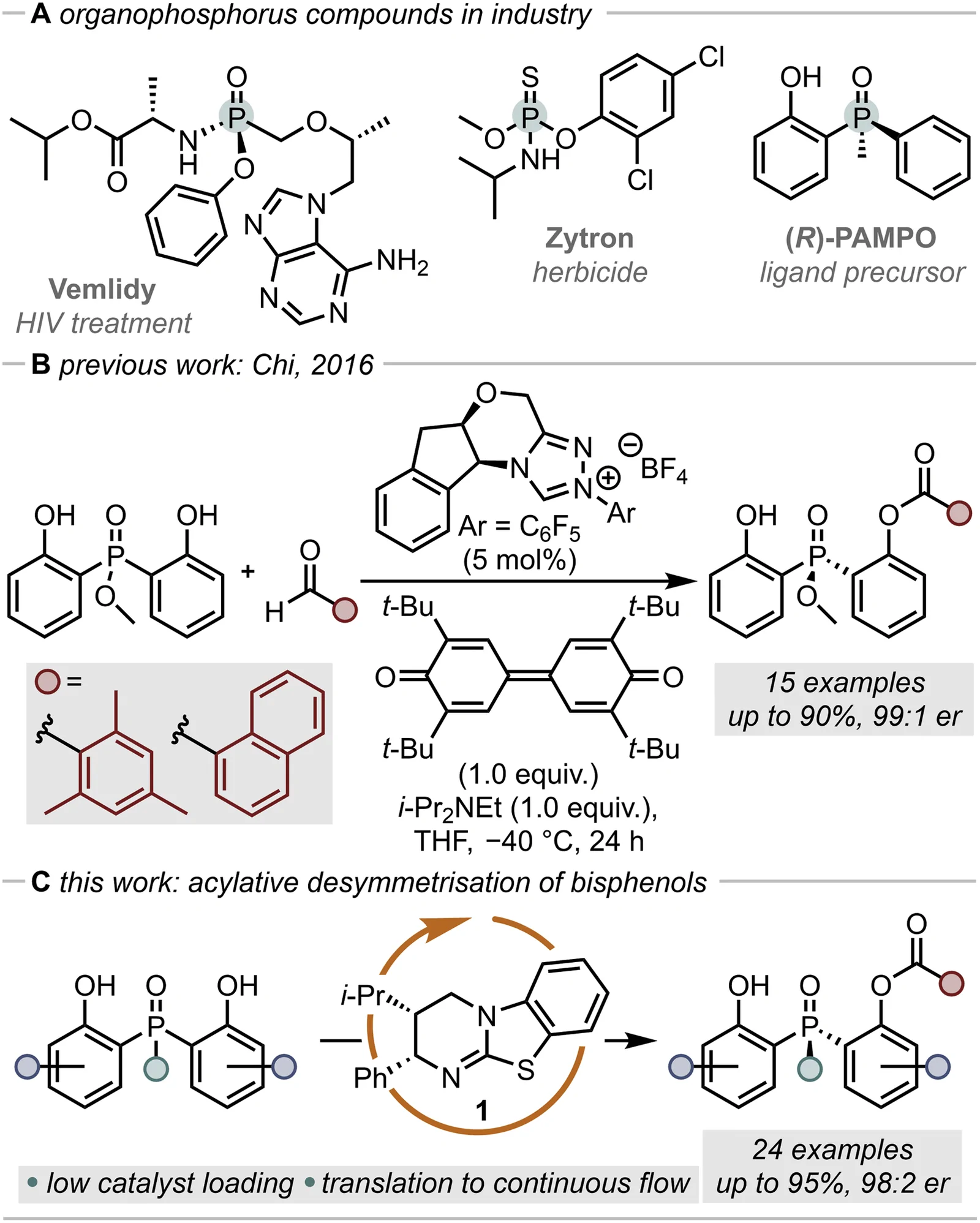
(A) importance of organophosphorus compounds; (B) previous work, Ar = C_6_F_6_ or 2,4,6-trichlorophenyl; (C) this work.

The acylative desymmetrisation of prochiral phosphorus derivatives is an effective approach towards these compounds, as demonstrated by Chi's work using an NHC catalyst (5 mol%) in the desymmetrisation of bisphenol phosphinates to obtain the corresponding esters in high yields and enantioselectivity (Fig. 1B).^19^ Chi's process illustrated an elegant approach to P(v)-stereogenic centres, with the products showing application as chiral bidentate Lewis base catalysts in an asymmetric aldol reaction. However, the reaction scope of the desymmetrisation demonstrated compatibility with only limited substitution patterns on the phenol scaffold, required a bulky mesityl- or 1-naphthaldehyde for effective enantiodiscrimination, and also required a stoichiometric amount of quinone oxidant. All of these factors led to limited utility of this method, as well as poor atom economy. More recently, Guo and co-workers presented a chiral pyridine *N*-oxide-catalysed acylative desymmetrisation of the same substrates, demonstrating low catalyst loadings of 0.05 mol% across 21 substrates, with up to 97% yield and 98 : 2 er.^20^ While this method was tolerant to less sterically-hindered alkyl-based acyl sources, the reaction scope was mainly limited to variation of the acyl source, with few examples varying the core bisphenol phosphinate scaffold. Concurrently, the Zhang group presented an alternative approach using a chiral thiourea catalyst,^21^ allowing the reaction to be performed at room temperature and still achieve high enantiocontrol (up to >99 : 1 er). Here, the scope was extended to some C(4,5)-substituted phenol groups, with a range of interesting product derivatisations displayed; however attempted extension to *meta*-bisphenol phosphinate scaffolds gave poor stereocontrol (53 : 47 er). The *meta*-bisphenol scaffold demonstrates a widely recognised problem of achieving remote stereocontrol in this system. During the preparation of this manuscript, the Xu group disclosed a Lewis base-catalysed approach to similar scaffolds using chiral DMAP derivatives. However for maximum selectivity their reaction process required cryogenic temperatures and chlorinated solvents to achieve high enantiocontrol (up to 99 : 1 er), while the scope was limited to C(4,5)-substituted phenol groups.^22^

Isothioureas have been widely used as chiral Lewis base organocatalysts in enantioselective acyl transfer processes since they were first established for the kinetic resolution of secondary alcohols by Birman and Li in 2006.^23^ Building upon our previous work on the atroposelective acylative enantioselective desymmetrisation of prochiral bisphenol phosphinates using the chiral isothiourea (2*S*,3*R*)-HyperBTM 1 as an acyl transfer catalyst was considered (Fig. 1C). Herein, we disclose a complementary approach to prior art, providing efficient access to highly enantioenriched phosphinate esters across a large variety of scaffolds. Translation of this process into continuous flow using a packed bed reactor and a polymer-supported isothiourea catalyst is also demonstrated. Mechanistic studies show the effect of a chiroablative pathway on product distribution and enantioselectivity.

## Results and discussion

### Investigation of optimal reaction conditions

Initial investigation began with ethyl bis(2-hydroxyphenyl) phosphinate 2, a slight excess of isobutyric anhydride (1.03 equiv.), 2,6-lutidine as a base and chiral isothiourea catalyst (2*S*,3*R*)-HyperBTM 1 (10 mol%) in toluene (0.1 M) at room temperature for 5 h, which provided phosphinate ester 3 in 72% yield with exceptional enantioselectivity (98 : 2 er), however 17% diester side-product 4 was also observed (Fig. 2, entry 1). In previous studies,^24^ the alternative isothiourea catalyst (*R*)-BTM has shown improved enantioselectivity for acyl transfer reactions on phenolic substrates; application in this case led to product in high enantioselectivity (60%, 98 : 2 er) but also led to increased formation (28%) of diester 4 (entry 2). In past literature processes high dilution^21^ or slow addition of reaction components over several hours^19^ has been necessary to minimise the formation of diester 4, so the concentration was decreased to 0.025 M (entry 3), which led to a reduction in diester formation (<1%) but also marginally reduced enantioselectivity (96%, 96 : 4 er) over a slightly longer reaction time of 6 h. Reducing the temperature to 0 °C and increasing the reaction time did not improve yield or enantioselectivity (entry 4, 94%, 96 : 4 er) so room temperature was preferred. Reducing the catalyst loading to 1 mol% at low concentration (entry 5) led to a decrease in both yield and enantioselectivity (77%, 90 : 10 er). However, by reverting to higher concentration (0.1 M) at this lower catalyst loading, a compromise between yield of 3, high enantioselectivity, and low formation of diester 4 was observed (entry 6 : 90%, 95 : 5 er, [<1%]). This offers comparable results to entry 3 alongside lower cost. A control experiment without catalyst (entry 7) showed a significant racemic background reaction (47% conversion) under these reaction conditions, which must be outcompeted in the presence of the isothiourea catalyst due to the high enantioselectivity observed. The absolute configuration within (*R*)-3 was unambiguously established by single crystal X-ray analysis (Fig. 2A). Notably an intramolecular hydrogen bond was observed between the phenolic hydrogen and the phosphinate ester P=O in the solid state and in solution as observed by a sharp downfield singlet (*δ*_H_ 11.8 ppm in d_8_-toluene).

**Fig. 2:**
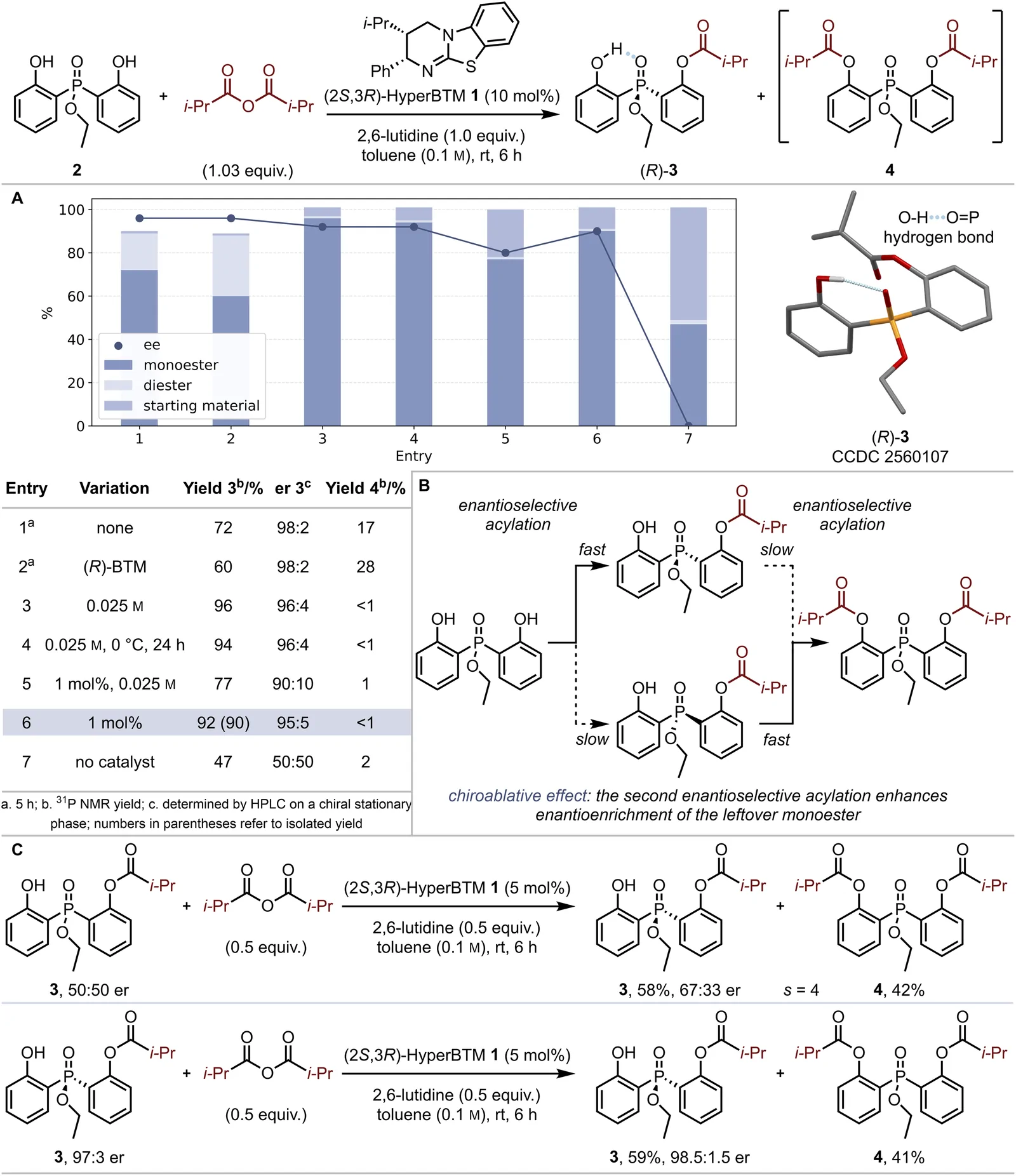
(A) Optimisation and absolute configuration; (B) chiroablative effect; (C) mechanistic control experiments. In line with best practice in the literature, *s* value is reported to the nearest integer and calculated using the equation *s* = ln[(1 − conv)(1 − ee(alcohol))]/ln[(1 − conv)(1 + ee(alcohol))] where alcohol = monoester 3.

### Investigation into the chiroablative effect

Throughout the extensive optimisation (see SI) a general trend indicated the formation of monoester 3 with highest enantiocontrol was observed alongside high yields of achiral diester 4. This led to an investigation into the potential for chiroablation in the second acylation step to form diester 4 (Fig. 2B). In principle, both acylation steps to form monoester 3 and diester 4 can proceed enantioselectively, with product distribution and selectivity dependent on (a) enantioselectivity of both acylation steps and (b) reaction conversion to both monoester 3 and diester 4.^25,26^ In theory the second acylation step could favour either enantiomer of monoester 3, leading to either amplification or erosion of enantioselectivity. To probe the validity of chiroablation and understand which enantiomer the second acylation favoured, the kinetic resolution (KR) of (±)-monoester 3 was performed, providing recovered bisphenol starting material (*R*)-3 in 67 : 33 er at 42% conversion (*s* = 4).^27,28^ This is consistent with preferential acylation of (*S*)-3 to form diester 4 (Fig. 2C).^28^ Although the selectivity of this KR is only moderate, the sequential nature of the desymmetrisation and KR has a significant impact on the enantioselectivity of monoester 3. To further demonstrate this effect, a highly enantioenriched sample of monoester 3 (97 : 3 er) was subjected to KR conditions, giving monoester 3 in 98.5 : 1.5 er at 42% conversion. Taken together, these processes indicate that the second acylation event is chiroablative – the formation of diester 4 enhances the enantioenrichment of the remaining monoester 3.^29,30^ However, given the moderate selectivity for this KR process, and the high yield and enantiocontrol of the monoester products (Fig. 3), this KR process is not the main contributor to the observed enantioselectivity of the monoester product that arises from the catalytic desymmetrisation. In some cases within the scope this phenomenon was exploited to increase the enantioselectivity of the monoester product, albeit to the detriment of product yield.

**Fig. 3:**
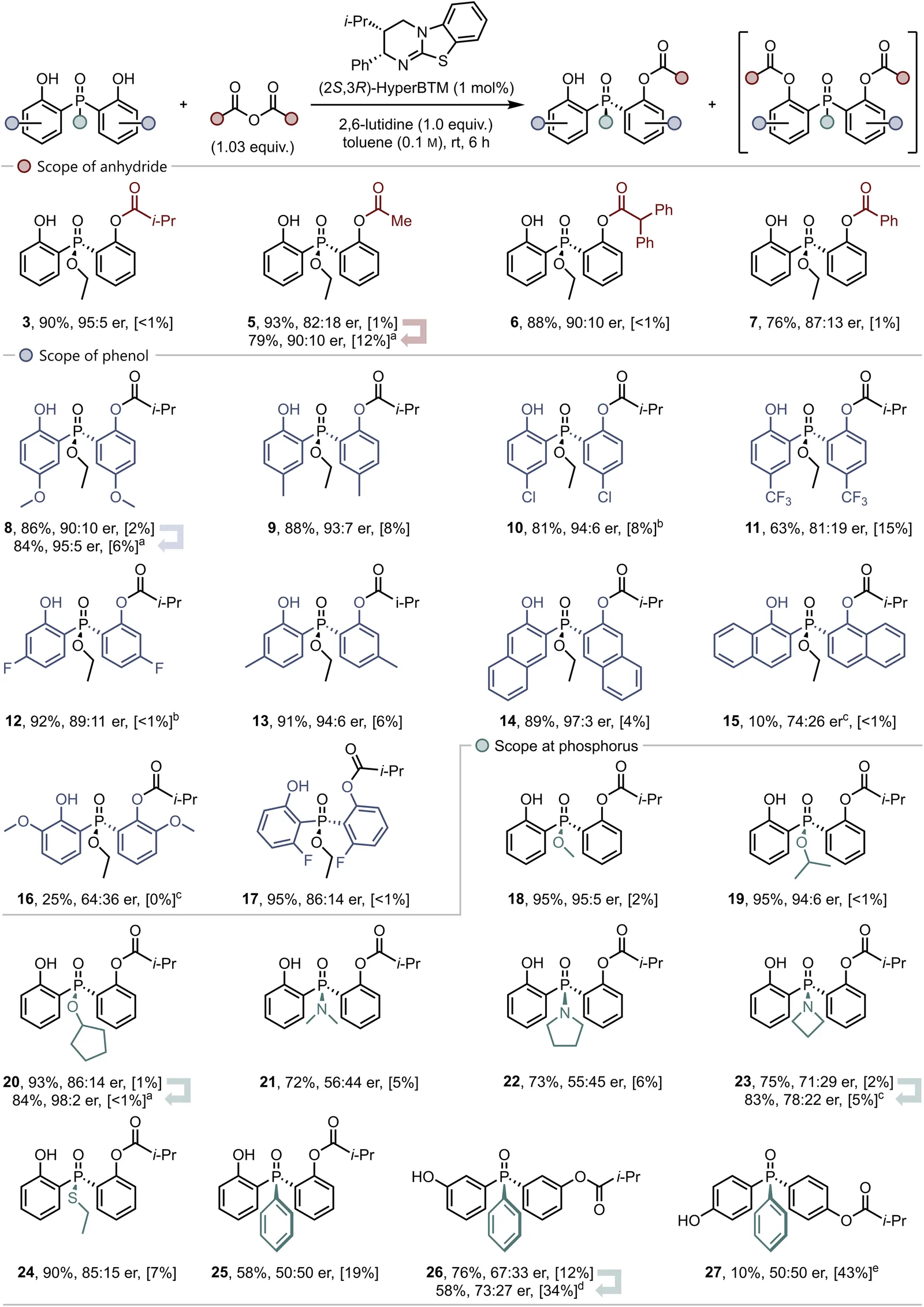
Scope and limitations of the process. All yields are of isolated product, er determined by HPLC analysis on a chiral stationary phase, reaction scale 0.1–0.2 mmol. Deviations from standard conditions: ^*a*^0 °C, HyperBTM (10 mol%), isobutyric anhydride (1.2 equiv.); ^*b*^HyperBTM (2.5 mol%); ^*c*^HyperBTM (10 mol%); ^*d*^isobutyric anhydride (1.25 equiv.); ^*e*^HyperBTM (10 mol%), isobutyric anhydride (1.25 equiv.), toluene: acetone (5 : 1).

### Scope and limitations

With optimised conditions in hand, the scope and limitations of the process were explored (Fig. 3). While the main aim was to develop an effective catalytic process at low catalyst loadings (1 mol%), selected variants were tested at 10 mol% catalyst loading either at room temperature or 0 °C to show that enhanced product enantioselectivity could be achieved if desired. Variation of the acyl group from the isobutyrate 3 (90%, 95 : 5 er) to acetate 5 led to similar reactivity, but reduced enantioselectivity (93%, 82 : 18 er) that could be enhanced with the use of 10 mol% catalyst (79%, 90 : 10 er). The sterically demanding diphenylacetate ester 6 was also generated similarly (88%, 90 : 10 er), whereas the benzoate ester 7 was formed in slightly reduced yield, albeit with no significant amount of diester side-product observed (76%, 87 : 13 er).

The effect of substitution of the phenol was investigated next, with an electron-donating 5-MeO group giving ester 8 in high yield and enantioselectivity (86%, 90 : 10 er) that could be improved with the use of 10 mol% catalyst loading at 0 °C (84%, 95 : 5 er). Both 5-Me and 5-Cl substituents were well-tolerated (**9–10**, 81–88%, 93 : 7–94 : 6 er), however 8% diester side-product was observed for the 5-Cl substrate 10. Incorporating the electron-withdrawing 5-F_3_C substituent led to 15% diester side-product and reduced enantioselectivity of ester 11, suggestive of a change in relative rates of acylation events. Substitution in the C(4)-position was well-tolerated, with 4-F, 4-Me and 2-naphthyl substituted esters **12–14** formed in high yields and high enantioselectivity (89–92%, 89 : 11–97 : 3 er). Application to a 1-naphthyl substituent 15 led to significant suppression of acylation and poor enantiodiscrimination even at 10 mol% catalyst loading (10%, 74 : 26 er), likely due to increased steric hindrance. Previous work in this area found that the presence of multiple *ortho*-substituents prevented effective enantioselective acylation of phenolic substrates.^30^ Interestingly, in this case 3-MeO-substituted ester 16 was accessed, albeit in low yield and poor enantiocontrol even at 10 mol% catalyst loading (25%, 64 : 36 er). Pleasingly, 6-F substitution was well-tolerated, providing access to ester 17 in 95% yield, with 86 : 14 er.

Variation of the phosphinate substituent was next explored. Changing the phosphinate OEt substituent to OMe 18 was well tolerated with high enantiocontrol (95%, 95 : 5 er) while extension to the α-branched O*i*-Pr 19 substituent also performed well (95%, 94 : 6 er). Further application to the α-branched cyclic *O*-cyclopentyl substituted ester 20 gave good reactivity but reduced enantiocontrol (93%, 86 : 14 er) that could be significantly enhanced with the use of 10 mol% catalyst loading at 0 °C (84%, 98 : 2 er). Interestingly in this case no increase in diester side-product was observed. Extension of this process to a range of nitrogen-based substituents was also investigated but led to esters 21 and 22 with poor enantiocontrol (72–73%, 55 : 45–56 : 44 er); although the azetidinyl substituted ester 23 was isolated with moderate enantioselectivity (75%, 71 : 29 er), which could be improved by using 10 mol% catalyst loading (83%, 78 : 22 er), albeit with a slight increase to 5% diester side-product. The corresponding phosphinothioate performed well, giving 24 in 90% yield and 85 : 15 er. Disappointingly, a P-Ph substituent gave 25 in only moderate yield and essentially racemic form (58%, 50 : 50 er) alongside a significant amount of diester side-product (19%). In previous studies on the desymmetrisation of these scaffolds, a particular challenge was garnering remote enantiocontrol for *meta*-OH acylation. In this methodology, the P-Ph *meta*-bisphenol performed marginally better than its *ortho*-counterpart, providing 26 in 76% yield with 67 : 33 er, and 12% diester side-product. To further improve the enantioselectivity for this compound, the chiroablative effect was exploited, using an excess of acylating agent, which provided 26 in 58% yield (73 : 27 er). Extension of the methodology to the P-Ph *para*-bisphenol gave a disappointing 10% yield of ester 27 in 50 : 50 er, which is consistent with previous literature^19^ and the wider issue of garnering remote stereocontrol in organocatalytic systems. Interestingly, in this reaction a significant amount of the diester side-product of 27 was observed (43%). A potential explanation for this observed reactivity relates to the insolubility of the corresponding *para*-bisphenol in the reaction medium, and the solubility of the mono- and diester products.

Further expansion of the methodology to NBoc-protected aminophenol scaffold as well as the P–O-benzyl, P–O-allyl and P–O-propargyl variations were not possible due to instability towards the phospha-Fries rearrangement conditions required to access their corresponding *ortho*-bisphenols (conditions shown in Fig. 4, see SI for further details).

**Fig. 4:**
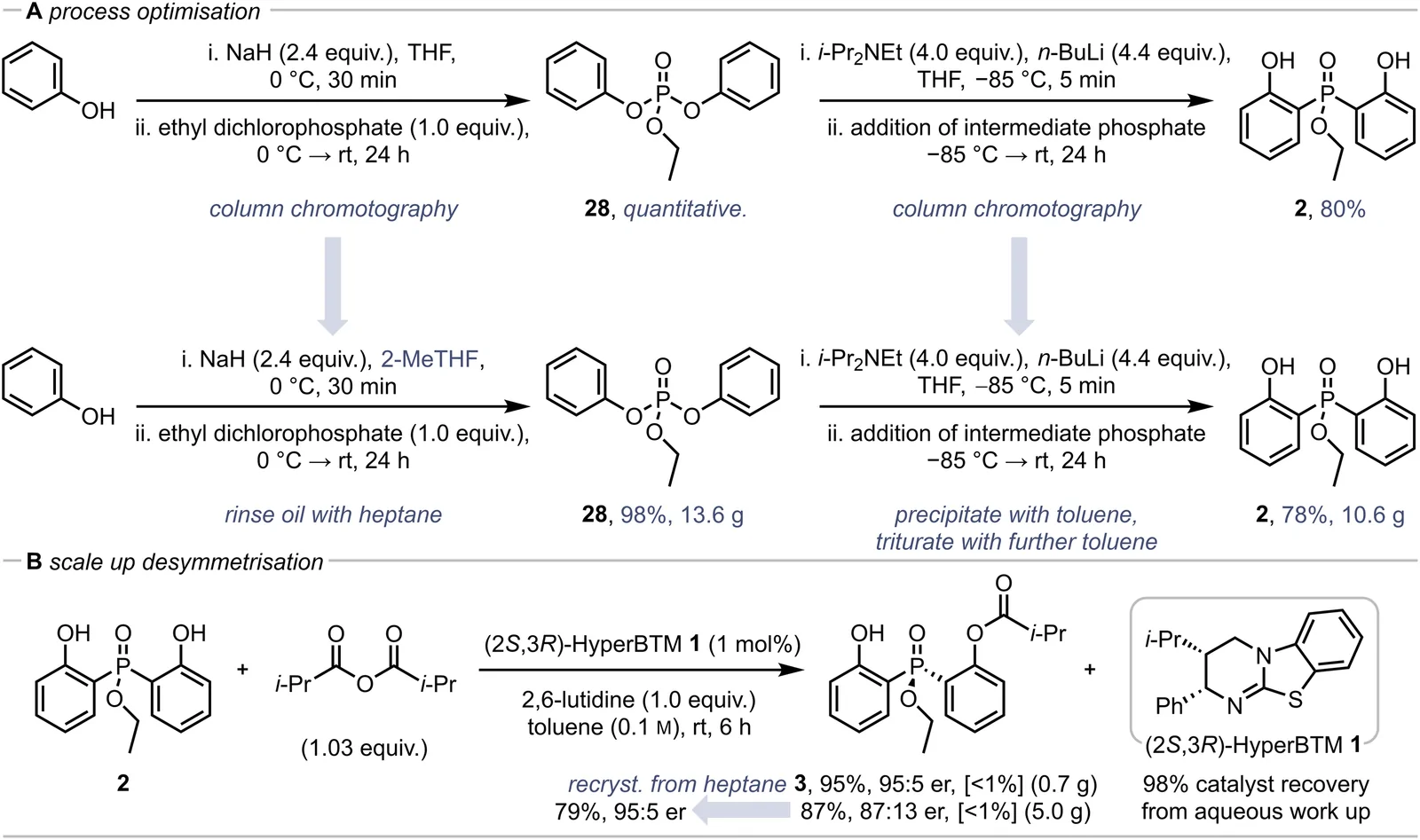
(A) optimisation of starting material synthesis; (B) scale up desymmetrisation. All yields are of isolated products, er determined by HPLC analysis on a chiral stationary phase.

### Process optimisation

In order to scale up the catalytic desymmetrisation more starting material 2 was required. The literature to bisphenol 2 began with deprotonation of phenol with NaH in THF at 0 °C followed by displacement of both chloride groups from ethyl dichlorophosphate to generate phosphate intermediate 28 in quantitative yield (Fig. 4A).^21^ Firstly, the swap to a greener solvent 2-MeTHF was made.^31^ Secondly, since the reaction is quantitative, column chromatography can be avoided and the mineral oil from NaH removed by sonicating the oily phosphate 28 with heptane. This process gave the intermediate phosphate 28 in 98% yield in high purity (>99%) on decagram scale. For the second step of the process LDA was formed *in situ*, then the intermediate phosphate 28 added to trigger a phospha-Fries rearrangement to generate bisphenol 2. To facilitate isolation on scale, as bisphenol 2 is a solid, purification by precipitation with cold toluene, followed by trituration in further cold toluene afforded bisphenol 2 in 78% yield and high purity (>98%) on decagram scale. With these minor changes to the process a significant reduction in solvent usage was achieved, and the starting material was obtained in high yield and purity.

### Scale up desymmetrisation

A preliminary scale up of the catalytic process to 500 mg scale accessed 3 in an improved 95% yield with 95 : 5 er (Fig. 4B). A further increase to multigram scale in an EasyMax vessel equipped with an overhead stirrer gave 3 in slightly reduced yield and enantioselectivity, albeit with efficient suppression of diester side-product 4 (87%, 87 : 13 er, [<1%]). Monoester product 3 crystallised from the reaction mixture in high purity (>99%), negating the need for column chromatography. Upon recrystallisation from heptane a scalemic mixture preferentially crystallised (60 : 40 er), however concentration and crystallisation of the mother liquor gave highly enantioenriched crystals of 3 (79%, 95 : 5 er). Furthermore, catalyst 1 was recovered in 98% yield *via* aqueous acidic workup followed by basifying and back-extraction into the organic phase (see SI).

### Catalyst recyclability testing

To aid catalyst recovery and recyclability the polystyrene-supported variant of the chiral isothiourea catalyst 29 was synthesised by demethylation of (2*S*,3*R*)-8-methoxyHyperBTM and subsequent immobilization onto Merrifield resin *via* previously established methodology (see SI for full details).^32^ Fixing the catalyst to a solid polystyrene support allows the isothiourea catalyst to be recovered from the reaction mixture by filtration, and simply washed, dried and reused (see SI). The advantage of catalyst recycling in the field of organocatalysis is that it can make up for the typically higher catalyst loadings that are seen compared to metal catalysis, while maintaining advantages such as low cost of goods and low toxicity. To test the proposed recyclability of the polystyrene-supported isothiourea catalyst 29, batch reactions were carried out sequentially and the catalyst recovered *via* filtration and dried in between. Since the optimised process used 1 mol% HyperBTM 1, the equivalent polymer-supported catalyst was used (Fig. 5A). The first reaction gave 94% yield of monoester 3, however only moderate enantioselectivity was observed (82 : 18 er, navy dots, Fig. 5A). Unfortunately, each time the catalyst was recycled a drop in yield of monester 3 was observed (dark blue bars, Fig. 5A) due to loss of catalyst during the recovery process on such small scale. To circumvent this the series was repeated with 5 mol% catalyst loading (Fig. 5B). Gratifyingly, this led to much steadier yield and enantioselectivity (dark blue bar and navy dots, Fig. 5B) across the first 100 turnovers, with effective suppression of diester 4 throughout, effectively demonstrating the recyclability of polymer-supported isothiourea catalyst 29.

**Fig. 5:**
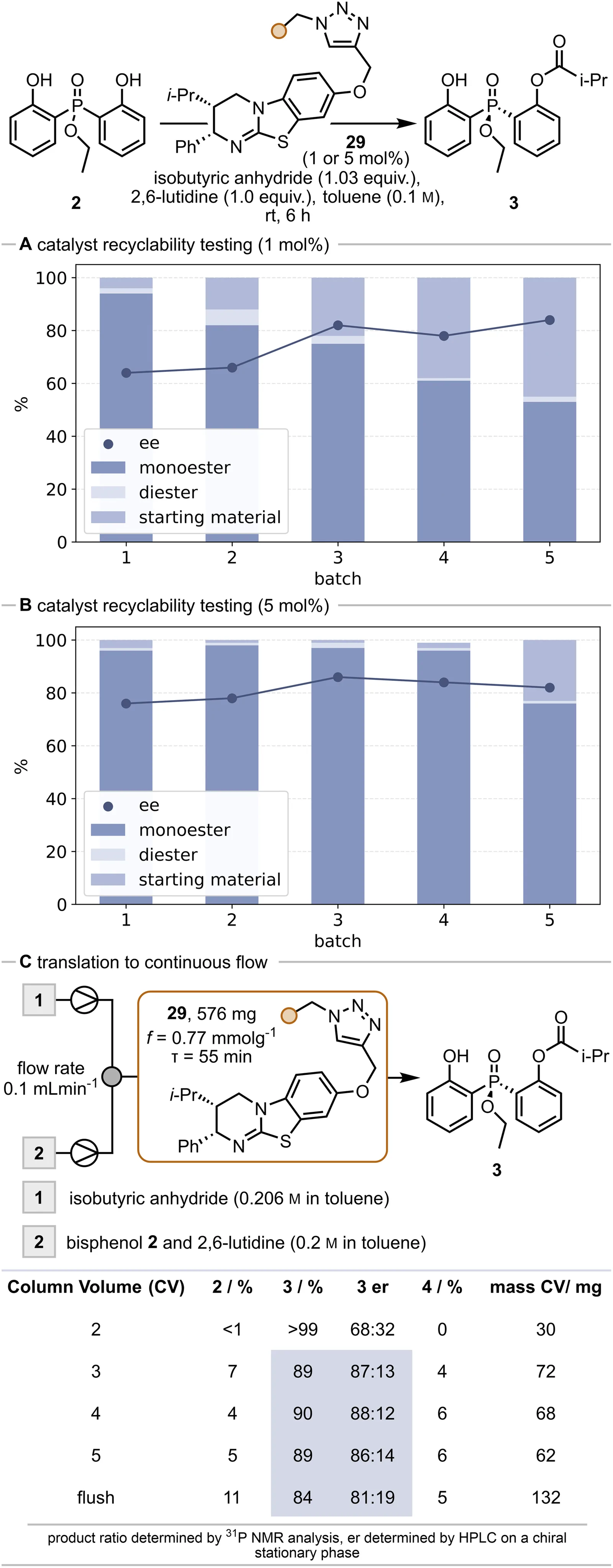
(A) Catalyst recyclability testing (1 mol%); (B) catalyst recyclability testing (5 mol%); (C) translation into continuous flow.

### Translation to continuous flow

To further demonstrate the applicability of this process across different technologies the immobilised catalyst was incorporated into a continuous flow setup using a packed bed reactor (Fig. 5C). These reactors allow the reaction solution to pass through the polymer-supported catalyst, embedded between filters, to achieve continuous product formation. Compared to conventional batch reactors this strategy allows for higher effective equivalents of the catalyst to substrate, which can lead to improved efficiency, and remove the additional filtration step to recover the catalyst. The polystyrene-supported isothiourea catalyst 29, (576 mg, 0.44 mmol) was loaded in an Omnifit column to create a vertical packed-bed reactor (flow from bottom to top). Based on previous demonstration of kinetic resolutions in flow,^33^ solutions of isobutyric anhydride in toluene (0.206 M = 1.03 equiv.) in one syringe and 2,6-lutidine (0.2 M = 1.0 equiv.) and ethyl bis(2-hydroxyphenyl)phosphinate 2 (0.2 M = 1.0 equiv.) in toluene in another syringe were delivered to the reactor bed *via* a mixing T-piece using a syringe pump with a combined flow rate of 0.1 mL min^−1^; equating to a residence time (*τ*) of 55 min (see SI for further details). The conversion was monitored across each column volume (CV) through the reactor, and found to stabilise at 3 CV, where it was held for two further CVs before flushing the system with toluene. From 3 CV to 5 CV was determined to be the steady state of the continuous flow reaction, and the yield and enantioselectivity were measured for these fractions, giving comparable results to the scale up procedure (Fig. 4) of 87% isolated yield of 3 in 87 : 13 er.

### Proposed mechanism

A proposed mechanism for this acylative desymmetrisation process is shown in Fig. 6A. Acylation of (2*S*,3*R*)-HyperBTM 1 with isobutyric anhydride generates the intermediate acyl ammonium ion pair 30. Following the established model developed in previous work by ourselves and others,^34,35^ preferential reaction of the acyl ammonium 30 with prochiral bisphenol 2 selectively generates enantioenriched monoester (*R*)-3, followed by regeneration of the free isothiourea catalyst 1 from the isothiouronium-carboxylate ion pair 32 by 2,6-lutidine. By analogy to previous computational studies, the observed selectivity in this acylation can be rationalised using the proposed transition state assembly 31. This is predicated upon the inclusion of a 1,5-O⋯S chalcogen bonding interaction 
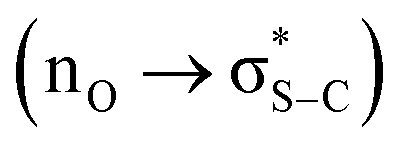
 between the acyl oxygen and the sulfur atom of the isothiourea catalyst, which ensures coplanarity between the two species and acts as a conformational lock.^35–55^ The isobutyrate counterion deprotonates the phenol, activating it towards acylation, while participating in a non-classical hydrogen-bonding interaction between the acidic α–H of the substrate bound catalyst and the carbonyl.^34,35^ Crystallographic analysis shows that in the solid state prochiral bisphenol phosphinate 2 adopts a dimeric assembly through inter- and intramolecular hydrogen bonding interactions between the phenolic hydroxyl group and the phosphoryl oxygen (OH⋯O=P) (Fig. 6). A similar intramolecular hydrogen-bond interaction is observed in the reaction solvent, as evidenced by the ^1^H NMR shift and appearance of the hydroxyl proton for bisphenol 2, which appears as a sharp singlet at *δ*_H_ 10.5 ppm (in d_8_-toluene). It is assumed that this intramolecular hydrogen bond will result in a preorganised conformation of the bisphenol in the reaction mixture and transition state, which interplays cooperatively with the observed catalyst-controlled stereodiscrimination.

**Fig. 6:**
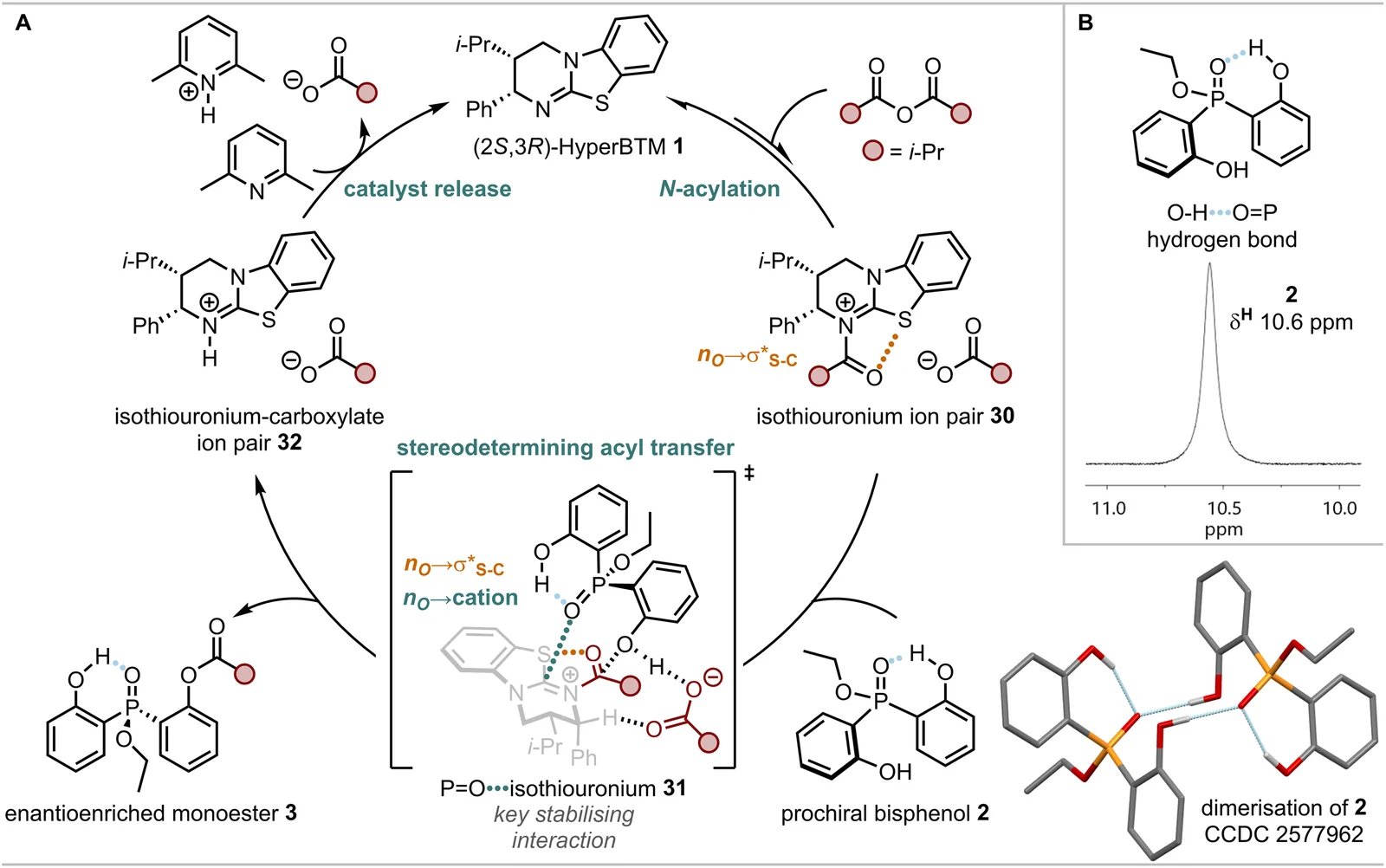
(A) proposed mechanistic cycle and stereochemical rationale for the enantioselective desymmetrisation of bisphenols and crystal structure of bisphenol starting material 2 showing hydrogen bonding and dimerisation in the solid state. Hydrogens not involved in hydrogen bonding and minor components of disorder are omitted for clarity; (B) ^1^H NMR spectra of prochiral bisphenol phosphinate 2 demonstrating hydrogen bonding in the reaction solvent.

## Conclusion

This manuscript reports the development of an enantioselective desymmetrisation of bisphenol phosphinates using the chiral isothiourea catalyst (2*S*,3*R*)-HyperBTM 1. The scope and limitations of this process have been explored, with good to excellent yields and moderate to high enantioselectivity observed across a broad range of substrate derivatives incorporating varied substitution on the aryl ring as well as at phosphorus. Furthermore, control experiments provided mechanistic insight into the role of bis-acylation in enhancing enantioenrichment. A chromatography-free process towards the bisphenol starting material was developed on decagram scale, and the catalytic reaction is amenable to scale up on multigram scale using bench grade solvents and reagents. Furthermore, the process was applied in continuous flow using a polymer-supported variant of the catalyst to afford monoester 3 in high yield and moderate enantioselectivity.

## Author contributions

FMP and ADS conceived the project. FMP and MIP carried out all experimental studies in consultation with ZZ, HP and ADS. DBC carried out single crystal X-ray analysis. FMP and ADS wrote the manuscript. All authors agreed on the finalised version of the manuscript.

## Conflicts of interest

The authors declare no competing interests.

## Supplementary Material

SC-017-D6SC05320C-s001

SC-017-D6SC05320C-s002

## Data Availability

CCDC 2560107 ((*R*)-3), 2577962 (2) and 2577963 (S41) contain the supplementary crystallographic data for this paper.^56*a*–*c*^ All data (experimental procedures and characterization data) that support the findings of this study are available within the article and its supplementary information (SI). The research data supporting this publication can be accessed at: https://doi.org/10.17630/76b1670f-72f8-4590-a50d-29cb97d07b9f, data underpinning: “Controlling phosphorus(v)-stereogenicity *via* organocatalytic acylative desymmetrisation of bisphenols”, University of St Andrews Research Portal. Supplementary information is available. See DOI: https://doi.org/10.1039/d6sc05320c.
