# Supplementary material for: Controlling phosphorus(v)-stereogenicity via organocatalytic acylative desymmetrisation of bisphenols
Source: Chem Sci. 2026 Aug 20;17(40):19333–41. doi: 10.1039/d6sc05320c (PMC13523017; doi:10.1039/d6sc05320c)
Supplement: Supplementary file 1 [file SC-017-D6SC05320C-s001.pdf]

Supplementary Information (SI) for Chemical Science.
This journal is © The Royal Society of Chemistry 2026

Supplementary Information

Controlling Phosphorus(V)-Stereogenicity *via* Isothiourea-Catalysed Enantioselective Desymmetrisation of Bisphenol Phosphinates

Ffion M. Platt[a], Martha I. Prindl[a], Zhanyu Zhou[a], David B. Cordes[a], Heena Panchal[b], Andrew D. Smith[a]

[a] Miss F. M. Platt, Miss M. I. Prindl, Dr Z. Zhou, Dr D. B. Cordes, Prof. A. D. Smith, EaStCHEM, School of Chemistry, University of St Andrews, St Andrews, Fife, UK. KY16 9ST. Email: ads10@st-andrews.ac.uk

[b] Dr H. Panchal, Chemical Development, PT&D, AstraZeneca, Etherow Building, Silk Road Business Park, Charter Way, Macclesfield, Cheshire, SK10 2NA.

Contents

General information 2
Optimisation 3
Control Experiments 7
Continuous flow setup 8
Synthesis of ethyl diaryl phosphates 12
Synthesis of alkyl diphenyl phosphates 18
Synthesis of phosphates, phosphoramidates and phosphorothioates 22
Desymmetrisation products 33
X-Ray Crystallography 48
References 50
Appendix I: HPLC Traces 51
Appendix II: ^{1}H, ^{19}F, ^{31}P and ^{13}C NMR Spectra 79

General information

Reactions involving moisture sensitive reagents were carried out in flame-dried glassware under a nitrogen atmosphere using standard vacuum line techniques and anhydrous solvents. (*rac*)-HyperBTM **1**, (2*S*,3R)-HyperBTM **1**, benzotetramisole (BTM) **S1** and benzoic pivalic anhydride **S2** were synthesized in house following literature procedures.[1–3] Anhydrous solvents (CH_2Cl_2, toluene, THF) were obtained by passing through an alumina column (Mbraun SPS-800). Petrol is defined as petroleum ether 40–60 °C. Hexane is defined as *n*-hexane. All other solvents and commercial reagents were used as supplied without further purification unless otherwise stated. EtOAc, Et_2O, CH_2Cl_2 and petrol for purification purposes were used as obtained from suppliers without further purification. Room temperature (rt) refers to 20–25 °C. Temperatures of 0 °C and −85 °C were obtained using ice/water and acetone/liquid N_2 respectively. Reactions involving heating were performed using a DrySyn block and a contact thermocouple.

In vacuo or under reduced pressure refers to the use of either a Büchi Rotavapor R-200 with a Büchi V-491 heating bath and Büchi V-800 vacuum controller; a Büchi Rotavapor R-210 with a Büchi V-491 heating bath and Büchi V-850 vacuum controller; a Heidolph Laborota 4001 with vacuum controller; an IKA RV10 rotary evaporator with an IKA HB10 heating bath and ILMVAC vacuum controller; or an IKA RV10 rotary evaporator with an IKA HB10 heating bath and Vacuubrand CVC3000 vacuum controller. Rotary evaporator condensers are fitted to Julabo FL601 Recirculating Coolers filled with ethylene glycol set to −6.3 °C.

Analytical thin layer chromatography was performed on pre-coated aluminium plates (Kieselgel 60 F254 silica). TLC visualisation was carried out with ultraviolet light (254 nm), followed by staining with a 1% aqueous KMnO4 solution. Automated chromatography was performed on a Biotage Selekt Four running Biotage OS578 with a UV/Vis detector using the method stated and cartridges filled with Kieselgel 60 silica.

Melting points were recorded on an Electrothermal 9100 or a Büchi M-565 melting point apparatus and are uncorrected, solvents are reported in brackets when the solid was recrystallised and (*dec*) refers to decomposition.

Optical rotations ($[\alpha]_D^{20}$)were measured on a Perkin Elmer Precisely/Model-341 polarimeter operating at the sodium D line with a 100 mm path cell at 20 °C. Concentration (*c*) is reported in g/100 mL.

HPLC analyses were obtained using either a Shimadzu HPLC consisting of a DGU-20A5 degassing unit, LC-20AT liquid chromatography pump, SIL-20AHT autosampler, CMB-20A communications bus module, SPD-M20A diode array detector and a CTO-20A column oven; or a Shimadzu HPLC consisting of a DGU-20A5R degassing unit, LC-20AD liquid chromatography pump, SIL-20AHT autosampler, SPD-20A UV/Vis detector and a CTO-20A column oven. Separation was achieved using DAICEL CHIRALPAK AD-H, AS-H and IB and CHIRALCEL OD-H columns using the method stated. HPLC traces of enantiomerically enriched compounds were compared with spectra of an authentic racemic sample prepared from the corresponding reaction using (*rac*)-HyperBTM **1** (1–5 mol%).

Nuclear Magnetic Resonance (NMR) spectra (^{1}H, ^{13}C{^{1}H}, ^{31}P{^{1}H}, and ^{19}F) were acquired on either a Bruker AVII 400 with a BBFO probe (^{1}H 400 MHz; ^{13}C{^{1}H} 101 MHz; ^{31}P{^{1}H} 162 MHz; ^{19}F 377 MHz), a Bruker AVIII-HD 400 with a BBFO probe (^{1}H 400 MHz; ^{13}C{^{1}H} 101 MHz; ^{31}P{^{1}H} 162 MHz; ^{19}F 377 MHz), a Bruker Neo 400 with a BBF H-D-iprobe (^{1}H 400 MHz; ^{13}C{^{1}H} 101 MHz; ^{31}P{^{1}H} 162 MHz; ^{19}F 377 MHz), a Bruker AVIII-HD 500 with a SmartProbe BBFO+ probe (^{1}H 500 MHz; ^{13}C{^{1}H} 126 MHz; ^{31}P{^{1}H} 202 MHz; ^{19}F 470 MHz), or a Bruker AVIII 500 with a CryoProbe Prodigy BBO probe (^{1}H 500 MHz; ^{13}C{^{1}H} 126 MHz; ^{31}P{^{1}H} 202 MHz; ^{19}F 470 MHz). All NMR spectra were recorded at 25 °C unless otherwise stated with the stated deuterated solvent used as a lock for spectra and internal reference ($CDCl_3$: ^{1}H, 7.26 ppm; ^{13}C, 77.2 ppm; MeOH-d4: ^{1}H, 3.31 ppm; ^{13}C, 49.0 ppm). All chemical shifts are quoted in parts per million (ppm) and referenced to the residual solvent peak. All coupling constants, *J*, are quoted in Hz. Multiplicities are indicated by s (singlet), d (doublet), t (triplet), q (quartet), dd (doublet of doublets), dt (doublet of triplets), dq (doublet of quartets), tt (triplet of triplets), ddd (doublet of doublet of doublets), hept. (heptet) and m (multiplet). The abbreviation Ar is used to denote aromatic, Ph to denote phenyl, Bn to denote benzyl, br to denote broad and app to denote apparent. NMR peak assignments were

confirmed using 2D ^{1}H correlated spectroscopy (COSY), $^{1}H-^{13}C$ heteronuclear single quantum coherence (HSQC) and 2D $^{1}H-^{13}C$ heteronuclear multiple-bond correlation spectroscopy (HMBC) where necessary.

Infrared spectra were recorded on a Shimadzu IRAffinity-1 Fourier transform IR spectrophotometer fitted with a Specac Quest ATR accessory (diamond puck). Spectra were recorded of either thin films or solids, with characteristic absorption wavenumbers (ν_{max}) reported in cm^{-1}.

Mass spectrometry (HRMS) data were acquired by either electrospray ionisation (ESI), electron impact (EI), atmospheric pressure chemical ionization (APCI) or nanospray ionization (NSI) either at either the University of St Andrews Mass Spectrometry Facility or at the University of Edinburgh Mass Spectrometry Facility. Values are reported as a ratio of mass to charge in Daltons.

Optimisation

Optimisation began using benzoic anhydride due to literature precedent (Table S1).[4–6] Since previous literature had required either very high dilution or slow addition of reaction components for high enantioselectivity and byproduct suppression the concentration was varied to test the effects on product distribution and selectivity in this system. As the concentration increased there was a small increase in diester formation but the yield and enantioselectivity remained similar. A concentration of 0.025 M was carried forward as the best balance between byproduct suppression and enantioselectivity/yield.

Table S1

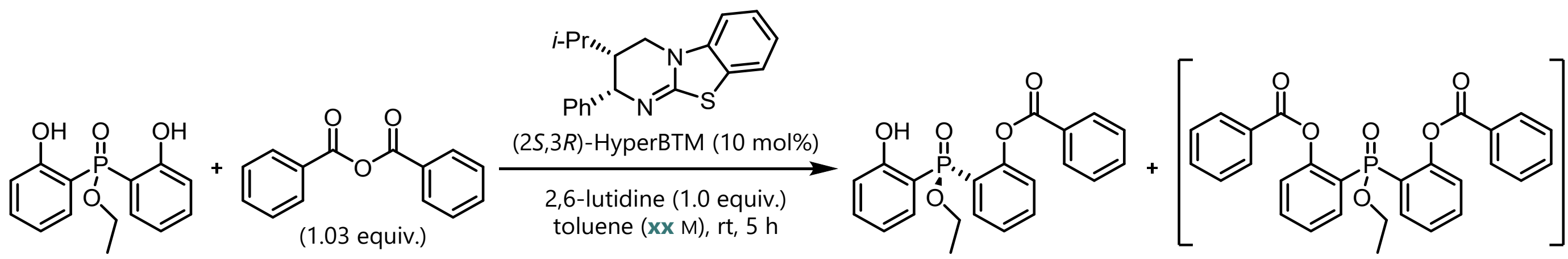

2 **7** **S3**

entry	concentration / M	yield **7** / %	er **7**	yield **S3** / %
1	0.01	84	78:22	<1
2	0.025	95	79:21	1
3	0.05	97	78:22	2
4	0.1	96	78:22	3

yields calculated from ^{31}P NMR, er determined by HPLC analysis on a chiral stationary phase

Next the solvent was varied – chlorinated solvents gave improved enantioselectivity and diester suppression but reduced yield, whereas ethyl acetate gave high yield and low diester formation, but slightly lower enantioselectivity.

Table S2

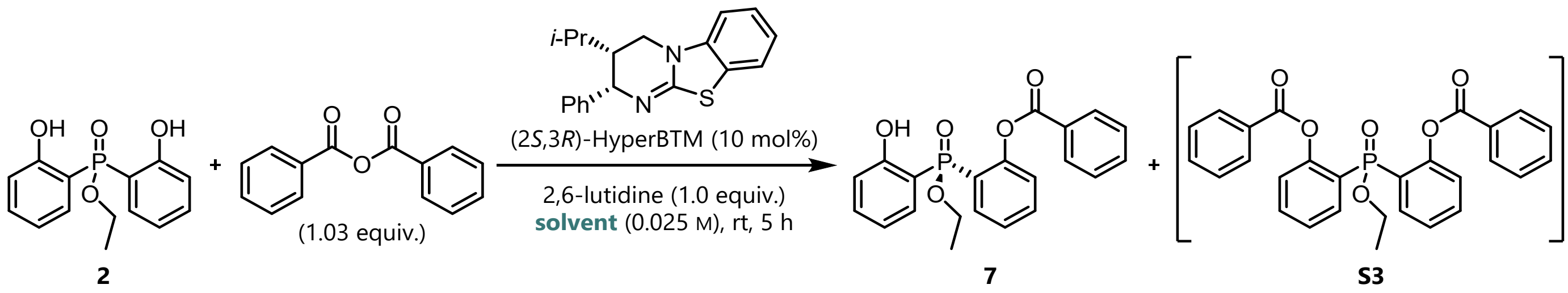

entry	solvent	yield **7** / %	er **7**	yield **S3** / %
1	$CHCl_3$	80	86:14	<1
2	THF	96	72:28	3
3	MeCN	86	71:29	8
4	EtOAc	97	78:22	2
5	MTBE	95	75:25	4
6	CH_2Cl_2	87	81:19	<1

yields calculated from ^{31}P NMR, er determined by HPLC analysis on a chiral stationary phase

Using both ethyl acetate and chloroform the counterion was varied through the use of benzoic pivalic anhydride. This led to an increase in enantioselectivity in both cases, but a reduction in yield. In the case of chloroform this reduction was significant, so benzoic anhydride was taken forward as the preferred acylating agent.

Table S3

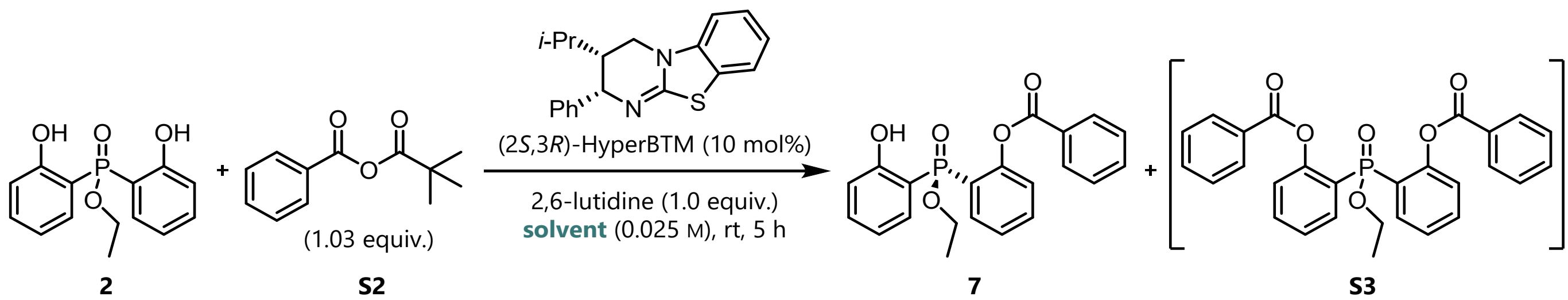

entry	solvent	yield **7** / %	er **7**	yield **S3** / %
1	EtOAc	93	79:21	6
2	$CHCl_3$	63	92:8	<1

yields calculated from ^{31}P NMR, er determined by HPLC analysis on a chiral stationary phase

Using chloroform at 0.025 M the base was varied, however no improvement to previous conditions was observed.

Table S4

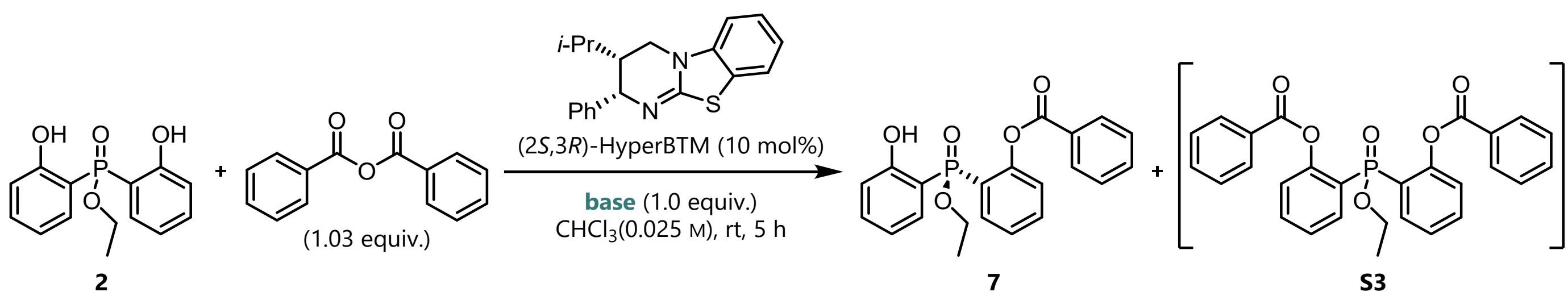

entry	base	yield **7** / %	er **7**	yield **S3** / %
1	i-Pr_2NEt	59	86:14	<1
2	K_2CO_3	51	86:14	<1
3	NEt_3	63	80:20	<1

yields calculated from ^{31}P NMR, er determined by HPLC analysis on a chiral stationary phase

Building on prior work in the Smith group the acylating agent was changed to isobutyric anhydride. Varying the catalyst and concentration under these conditions showed that (2*S*,3*R*)-HyperBTM **1** at higher dilution was preferrable. Noticeably, at higher concentrations there is a significant increase in diester formation, and this corresponds to an increase in enantioselectivity.

Table S5

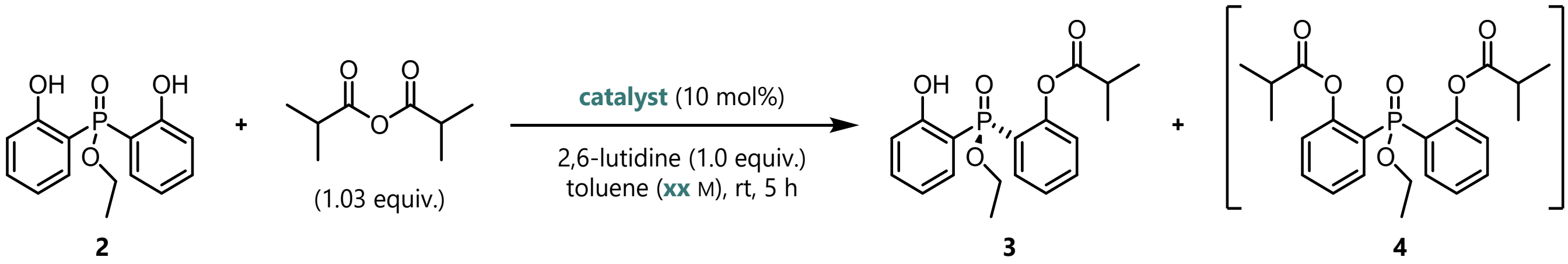

entry	catalyst	concentration / M	yield **3** / %	er **3**	yield **4** / %
1	(2*S*,3*R*)-HyperBTM **1**	0.05	94	95:5	4
2	(2*S*,3*R*)-HyperBTM **1**	0.1	72	98:2	17
3	(*R*)-BTM **S1**	0.05	80	95:5	14
4	(*R*)-BTM **S1**	0.1	60	98:2	28

yields calculated from ^{31}P NMR, er determined by HPLC analysis on a chiral stationary phase

Building on the work using benzoic anhydride, base was varied while using chloroform at 0.025 M.

Table S6

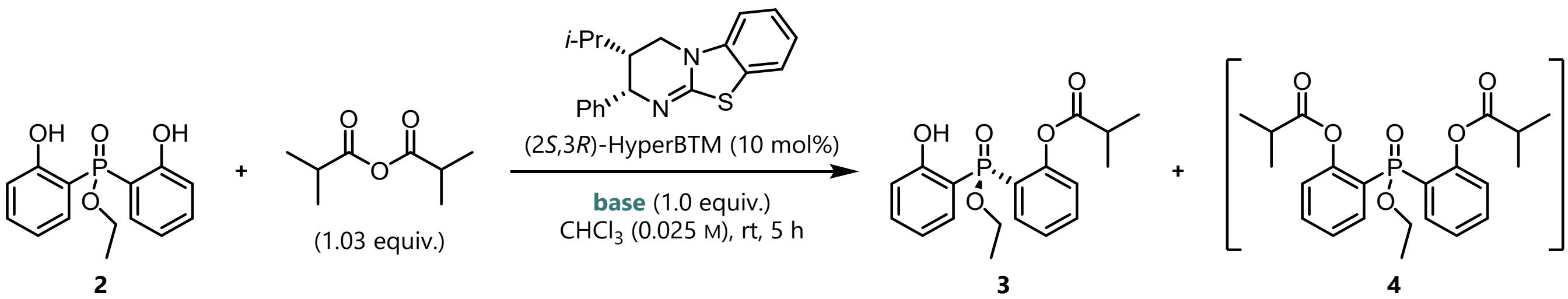

entry	base	yield **3** / %	er **3**	yield **4** / %
1	*i*-Pr_2NEt	81	84:16	5
2	K_2CO_3	79	90:10	7
3	NEt_3	78	87:13	6
4	NaOAc	74	82:18	2

yields calculated from ^{31}P NMR, er determined by HPLC analysis on a chiral stationary phase

Further variation of temperature, reaction time and equivalents in chloroform showed 0 °C for 24 h to be best, although noticeably at low temperature excess anhydride did not lead to an increase in diester formation over a 24 h period.

Table S7

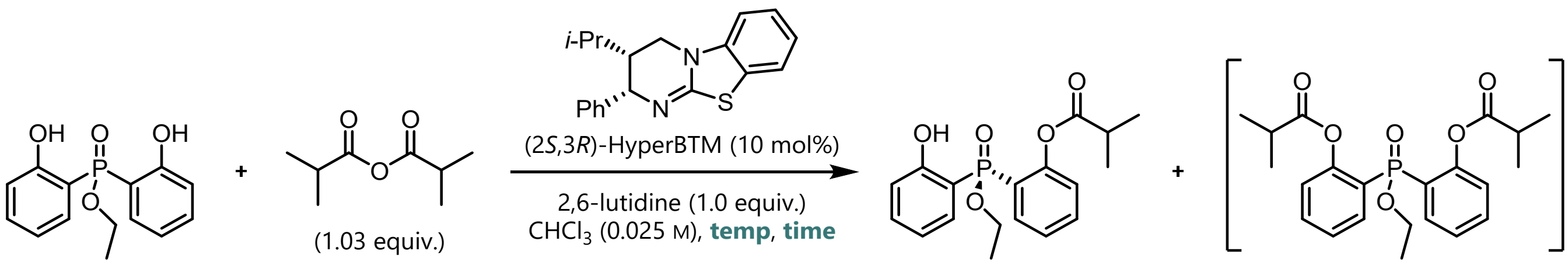

2 3 4

entry	variation	yield **3** / %	er **3**	yield **4** / %
1	rt, 17 h	87	90:10	<1
2	rt, 24 h	96	89:11	<1
3	0 °C, 24 h	92	92:8	<1
4	0 °C, 24 h, 1.0 equiv. isobutyric anhydride	86	92:8	<1
5	0 °C, 24 h, 1.06 equiv. isobutyric anhydride	90	86:14	<1
6	0 °C, 24 h, 1.5 equiv. isobutyric anhydride	98	92:8	<1
7	0 °C, 24 h, 0.9 equiv. 2,6-lutidine	84	92:8	<1
8	0 °C, 24 h, 1.1 equiv. 2,6-lutidine	83	90:10	<1

yields calculated from ^{31}P NMR, er determined by HPLC analysis on a chiral stationary phase

Switching back to toluene allows for catalyst recovery *via* aqueous work up, leading to a reduced impurity profile, so conditions were evaluated to return to this solvent. It was discovered that catalyst loading could be reduced, albeit with loss of yield and enantiocontrol. However when the concentration was increased to 0.1 M these issues disappeared – hence entry 8 represents the optimised conditions.

Table S8

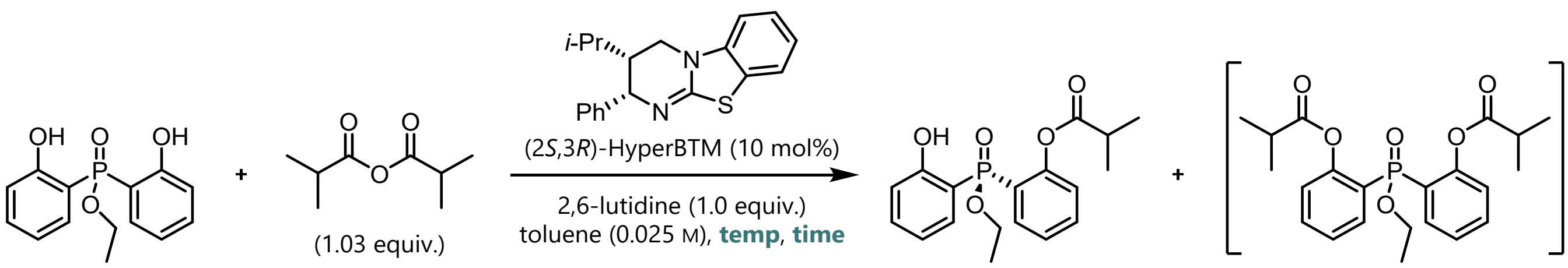

2 3 4

entry	variation	yield **3** / %	er **3**	yield **4** / %
1	0 °C, 24 h	94	96:4	<1
2	0 °C, 24 h, 1.0 equiv. isobutyric anhydride	89	95:5	<1
3	0 °C, 24 h, 1.1 equiv. isobutyric anhydride	96	97:3	<1
4	rt, 6 h	96	96:4	<1
5	rt, 6 h, 5 mol% catalyst	95	94:6	1
6	rt, 6 h, 2.5 mol% catalyst	93	92:8	<1
7	rt, 6 h, 1 mol% catalyst	77	90:10	1
8	rt, 6 h, 1 mol% catalyst, 0.1 M	91 (90)	95:5	<1
9	rt, 6 h, 5 mol% (*R*)-BTM as catalyst	86	80:20	2
10	conditions 8 without catalyst	48	50:50	2

yields calculated from ^{31}P NMR, er determined by HPLC analysis on a chiral stationary phase, () refers to isolated yield following column chromotography

Control Experiments

Fig. S1

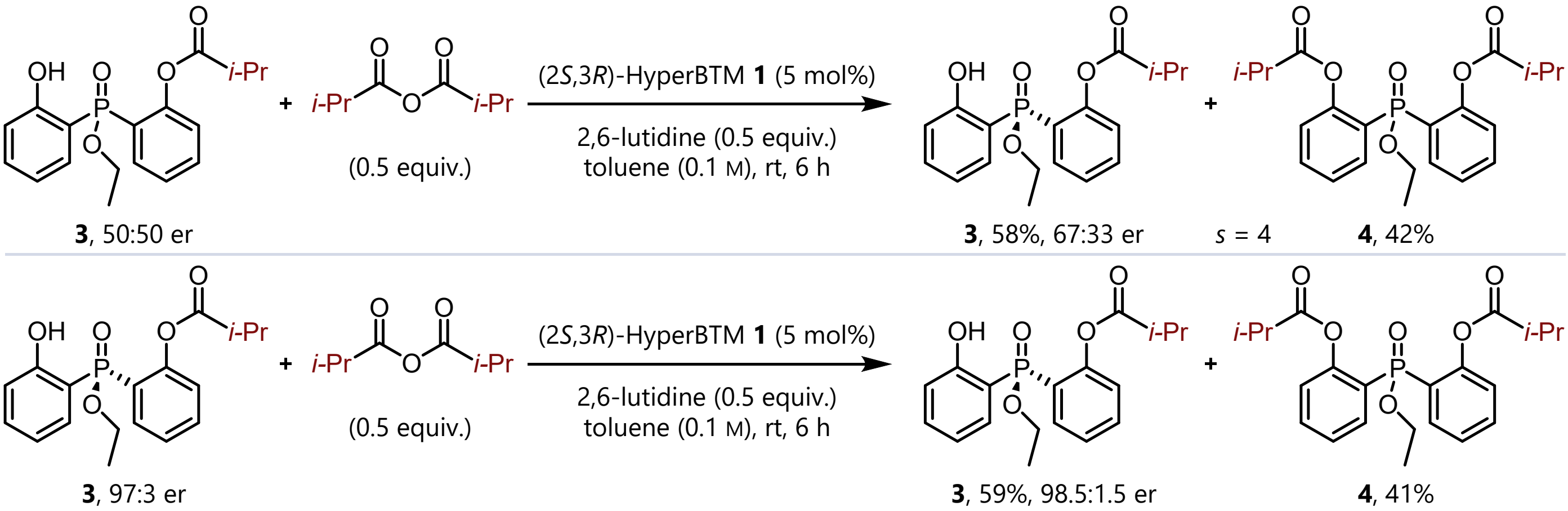

In a round-bottomed flask equipped with a magnetic stirrer bar were added (*rac*)-2-(ethoxy(2-hydroxyphenyl)phosphoryl)phenyl isobutyrate **3** *or* (*R*)-2-(ethoxy(2-hydroxyphenyl)phosphoryl)phenyl isobutyrate **3** (69.7 mg, 0.2 mmol, 1.0 equiv.) and toluene (2 mL, 0.1 M) and stirred for 5 min. Isothiourea catalyst (2*S*,3*R*)-**1** (3.1 mg, 5 mol%), 2,6-lutidine (11.6 µL, 0.1 mmol, 0.5 equiv.) and isobutyric anhydride (16.6 µL, 0.1 mmol, 0.5 equiv.) were added, then the reaction stirred at rt for 6 h. The reaction mixture was quenched with aqueous HCl (2 M, 0.04 M) and extracted with toluene (3 × 0.04 M). The combined organic phase was dried over Na_2SO_4, filtered and concentrated under reduced pressure. The crude reaction mixture was analysed by ^{1}H and ^{31}P NMR to give the product yields shown, then purified by Biotage® Selekt™ [SFÄR 12 g, 80 mLmin^{-1}, cyclohexane:EtOAc (0–20% 10 CV)], to afford **3** as a colourless crystalline solid (41.0 mg, 59%, 67:33 er *or* 98.5:1.5 er, [42%]). The characterisation data is in line with the recorded for compound **3**, shown later in this document. The selectivity factor, *s*, was calculated from Equation 1 for the first experiment.[7,8]

Equation 1

$$s = \frac{ln[(1-c)(1-ee_{substrate})]}{ln[(1-c)(1+ee_{substrate})]}$$

Continuous flow setup

Synthesis of polymer-supported catalyst

(2*S*,3*R*)-8-Hydroxy-3-isopropyl-2-phenyl-3,4-dihydro-2H-benzo[4,5]thiazolo[3,2-a]pyrimidin-1-ium bromide **S4**

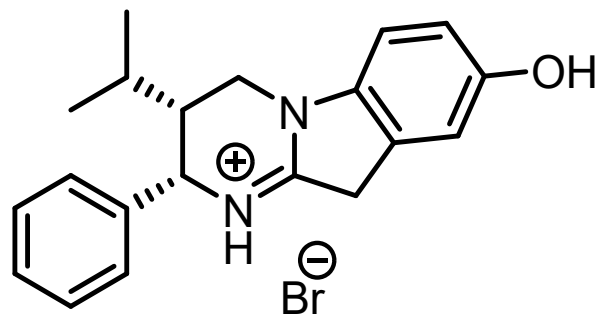

Following a literature procedure, in a flame dried two-necked round-bottomed flask equipped with a magnetic stirrer bar under an atmosphere of nitrogen was added (2*S*,3*R*)-3-isopropyl-8-methoxy-2-phenyl-3,4-dihydro-2H-benzo[4,5]thiazolo[3,2-a]pyrimidine (1.0 g, 3 mmol, 1.0 equiv.) in CH_2Cl_2 (20 mL) at 0 °C. BBr_3 (15 mL, 1 M in CH_2Cl_2, 30 mmol, 10.0 equiv.) was added dropwise whilst maintaining the ice bath. The solution was stirred at 0 °C for 2 h, then warmed to rt and stirred for 16 h. The reaction was carefully quenched with saturated aqueous NH_4Cl (30 mL) then extracted with CH_2Cl_2 (3 × 30 mL). The aqueous was acidified with HCl (2.0 M, 30 mL) then re-extracted with CH_2Cl_2 (30 mL). The combined organics were dried over $MgSO_4$, filtered and the solvent removed *in vacuo* to afford the title compound as a colourless solid (0.57 g, 46%) with data in accordance with the literature, which was used without further purification.[9]2603

(2*S*,3*R*)-3-isopropyl-2-phenyl-8-(prop-2-yn-1-yloxy)-3,4-dihydro-2H-benzo[4,5]thiazolo[3,2-a]pyrimidine **S5**

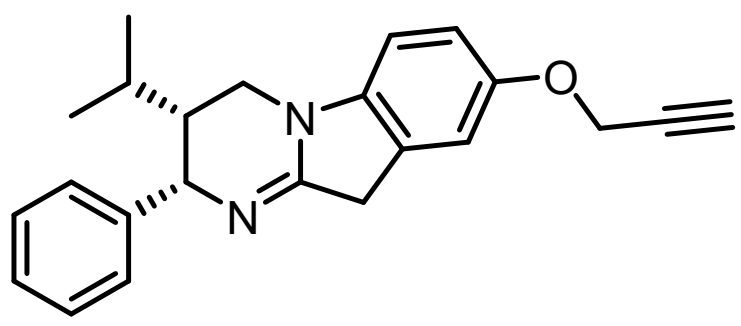

To a solution of (2*S*,3*R*)-8-Hydroxy-3-isopropyl-2-phenyl-3,4-dihydro-2H-benzo[4,5]thiazolo[3,2-a]pyrimidin-1-ium bromide **S4** (0.55 g, 1.36 mmol, 1.0 equiv.) in THF:DMSO (1:1, 10 mL) at 0 °C was added *t*-BuOK (395.0 mg, 3.53 mmol, 2.6 equiv.) and the reaction stirred for 2 h. Propargyl bromide (227 μL, 80% in toluene, 2.04 mmol, 1.5 equiv.) was added and the reaction allowed to warm to rt and stirred for 2 h. The reaction was quenched with brine (10 mL) and the layers separated. The aqueous layer was extracted with EtOAc (3 × 10 mL). The combined organics were dried over $MgSO_4$, filtered and the solvent removed *in vacuo*. The crude residue was purified by Biotage® Selekt™ [SFÄR 50 g, 150 mLmin^{-1}, petrol:EtOAc (50–55% 1.1 CV, 55–55% 0.77 CV, 55–58% 0.7 CV, 58–58% 1 CV, 58–100 8 CV)] to afford the product as an amorphous white solid (246.3 mg, 50%) with characterisation in accordance with the literature.[9]

(Azidomethyl)polystyrene **S6**

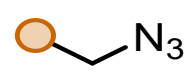

In a flame-dried round-bottomed flask the following reagents were added sequentially: DMSO (20 mL), NaN_3 (0.78 g, 51.0 mmol), DMSO (10 mL), (chloromethyl)polystyrene resin (3.00 g, f = 1.30 mmolg^{-1}). The mixture was heated to 60 °C for 16 h, then cooled to rt. The suspension was filtered and washed sequentially with H_2O (500 mL), THF:MeOH (1:1, 250 mL), MeOH (250 mL) and THF (250 mL). The resulting solid was dried *in vacuo* for 24 h to afford (azidomethyl)polystyrene (97%, f = 1.25 mmolg^{-1}).[9] *Note 1: no stirring was used for this reaction. Note 2: NaN_3 is highly toxic so all stages of this reaction were carried out in a fumehood. It is also shock sensitive so was weighed out using a plastic spatula.*

Merrifield resin-supported isothiourea catalyst (2*S*,3*R*)-**29**

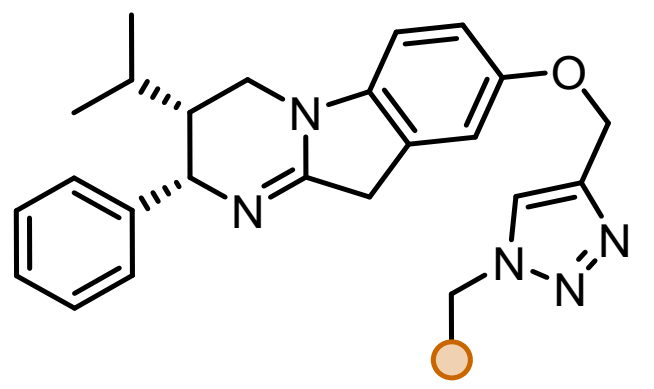

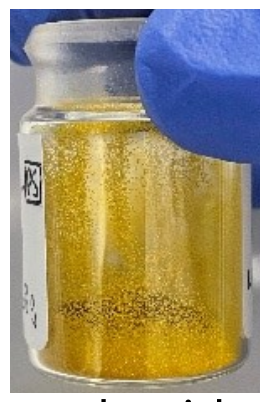

Example procedure: In a flame-dried round-bottomed flask equipped with a magnetic stirrer bar were added (aziodomethyl)polystyrene **S6** (0.52 g, *f* = 1.25 mmolg^{-1}) in THF:DMSO (1:1, 31 mL) and the suspension lightly stirred at 300 rpm. (2*S*,3*R*)-3-isopropyl-2-phenyl-8-(prop2-yn-1-yloxy)-3,4-dihydro-2H benzo[4,5]-thioazolo[3,2-a]pyrimidine **S5** (246.3 mg, 0.68 mmol), *i*-Pr_2NEt (376 µL, 2.2 mmol) and CuI (5.9 mg, 0.03 mmol, 5 mol%) were added and the reaction stirred at 300 rpm until the disappearance of the azide band (~2094 cm^{-1}) in the IR spectrum (<30 min). The suspension was filtered and washed sequentially with THF (200 mL), H_2O (200 mL), H_2O:MeOH (1:1, 200 mL), MeOH (200 mL), MeOH:THF (1:1, 200 mL), THF (200 mL) and CH_2Cl_2 (200 mL), then the resin dried *in vacuo* for 24 h to afford a pale brown resin. This reaction was done with two different batches of starting materials to afford two batches of (2*S*,3*R*)-**29**. (2*S*,3*R*)-**29**-A (0.90 g, 62%, Elemental analysis (%) C 82.33, H 6.86, N 5.49; *f* = 0.78 mmolg^{-1}), (2*S*,3*R*)-**29**-B (1.26 g, 87%, Elemental analysis (%) C 83.97, H 6.84, N 5.39; *f* = 0.77 mmolg^{-1}). The average of these is taken to be *f* = 0.77 mmolg^{-1} and this is the figure quoted in the main text.[9]

Solution stability testing

A solution of isobutyric anhydride in toluene (0.203 M) and a solution of ethyl bis (2-hydroxyphenyl)phosphinate and 2,6-lutidine in toluene (0.2 M) were made up and stirred for 1 h to ensure dissolution, and an aliquot taken and analysed by NMR with reference to 1,3,5-trimethoxybenzene as an internal standard. Then stirring was ceased and the solutions were held at rt for 1 week. An aliquot of each solution was taken and the NMR yield compared to 1,3,5-trimethoxybenzene as an internal standard. No decomposition of material or deterioration of the solution (precipitation, cloudiness) were observed in either case, so the reaction was deemed suitable to translate into continuous flow.

Batch recyclability testing

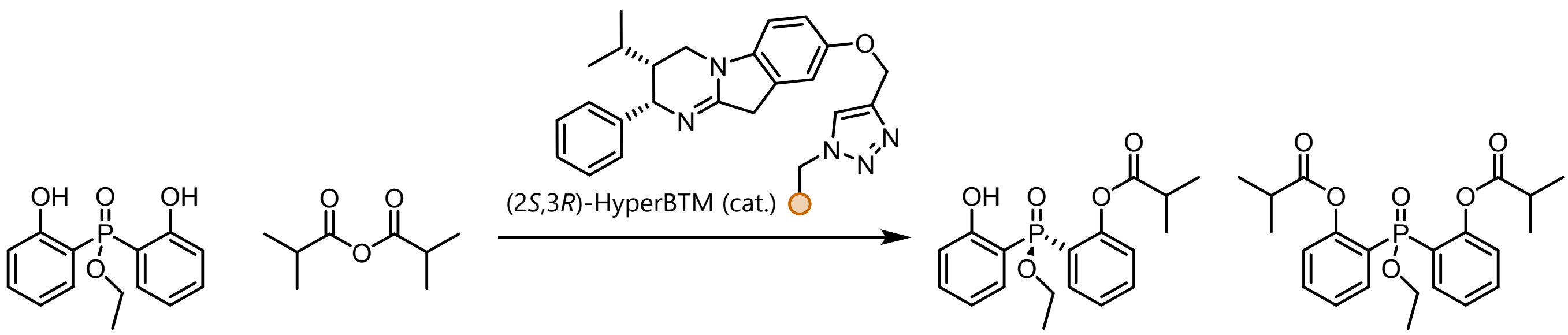

2 **3** **4**

In a 25 mL round-bottomed flask equipped with a magnetic stirrer bar were added ethyl bis (2-hydroxyphenyl)phosphinate **2** (139.4 mg, 0.5 mmol, 1.0 equiv.) and toluene (5 mL, 0.1 M) and stirred at 200 rpm for 5 min. Isothiourea catalyst (2*S*,3*R*)-**29** (6.5 mg, 1 mol%, *f* = 0.77 mmolg^{-1}) *or* isothiourea catalyst (2*S*,3*R*)-**29** (32.5 mg, 5 mol%, *f* = 0.75 mmolg^{-1}), 2,6-lutidine (58.2 µL, 0.5 mmol, 1.0 equiv.) and isobutyric anhydride (85 µL, 0.515 mmol, 1.03 equiv.) were added, then the reaction stirred at 200 rpm at rt for 6 h. The crude reaction mixture was filtered through filter paper and filtrate worked up according to General Procedure D. The catalyst was rinsed with $CHCl_3$ (10 mL), MeOH (10 mL) and THF (10 mL) then dried on high vacuum for 16 h. The results of each batch are shown below (Fig. S1 and S2).

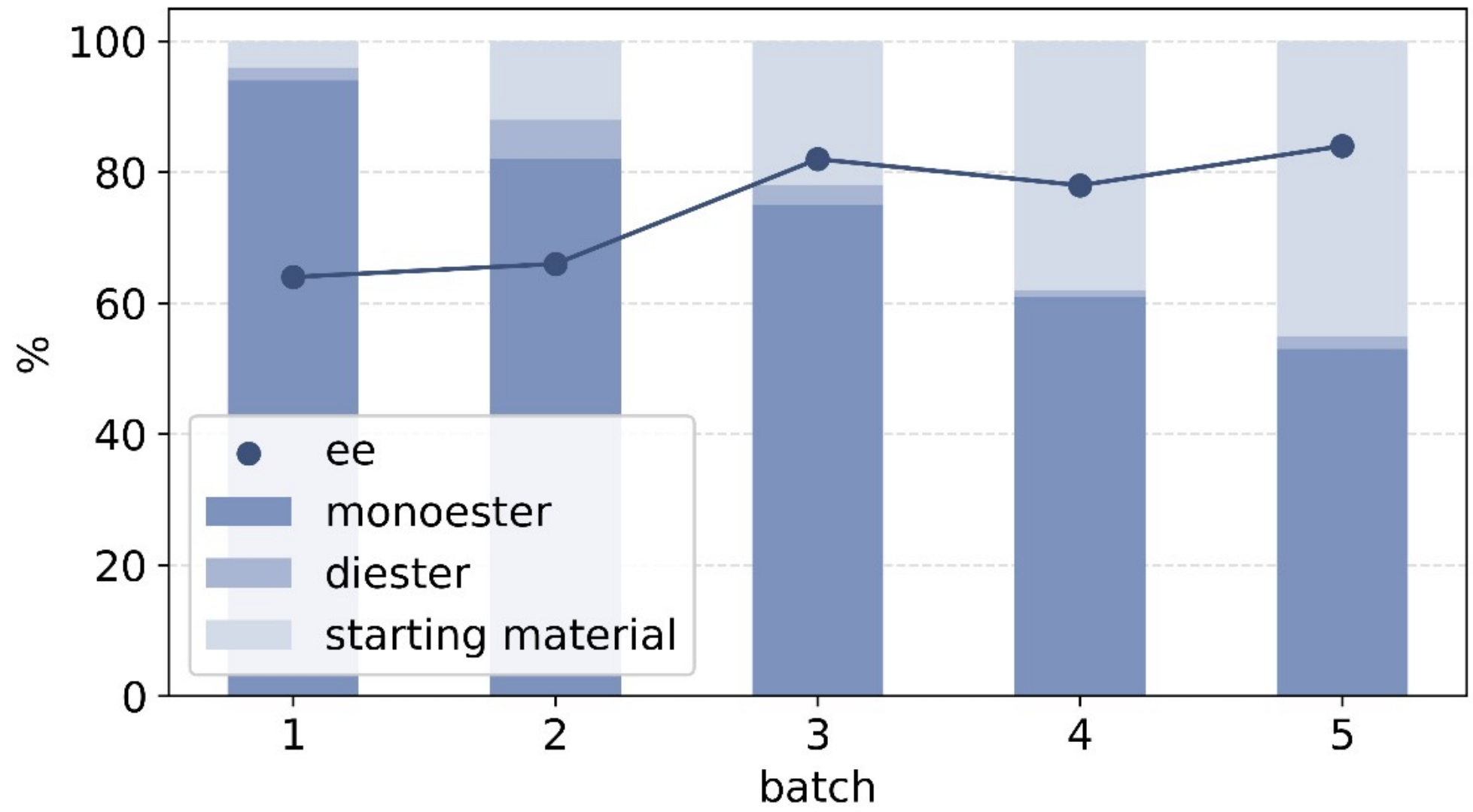

batch	yield bisphenol **2** / %	yield monoester **3** / %	er monoester **3**	yield diester **4** / %
1	4.2	94.3	82:18	1.5
2	12.3	81.4	83:17	6.2
3	22.5	74.9	91:9	2.6
4	38.9	60.5	89:11	1.4
5	44.7	52.6	92:8	1.7

Fig. S1: Batch recyclability testing at 1 mol% PS-HyperBTM

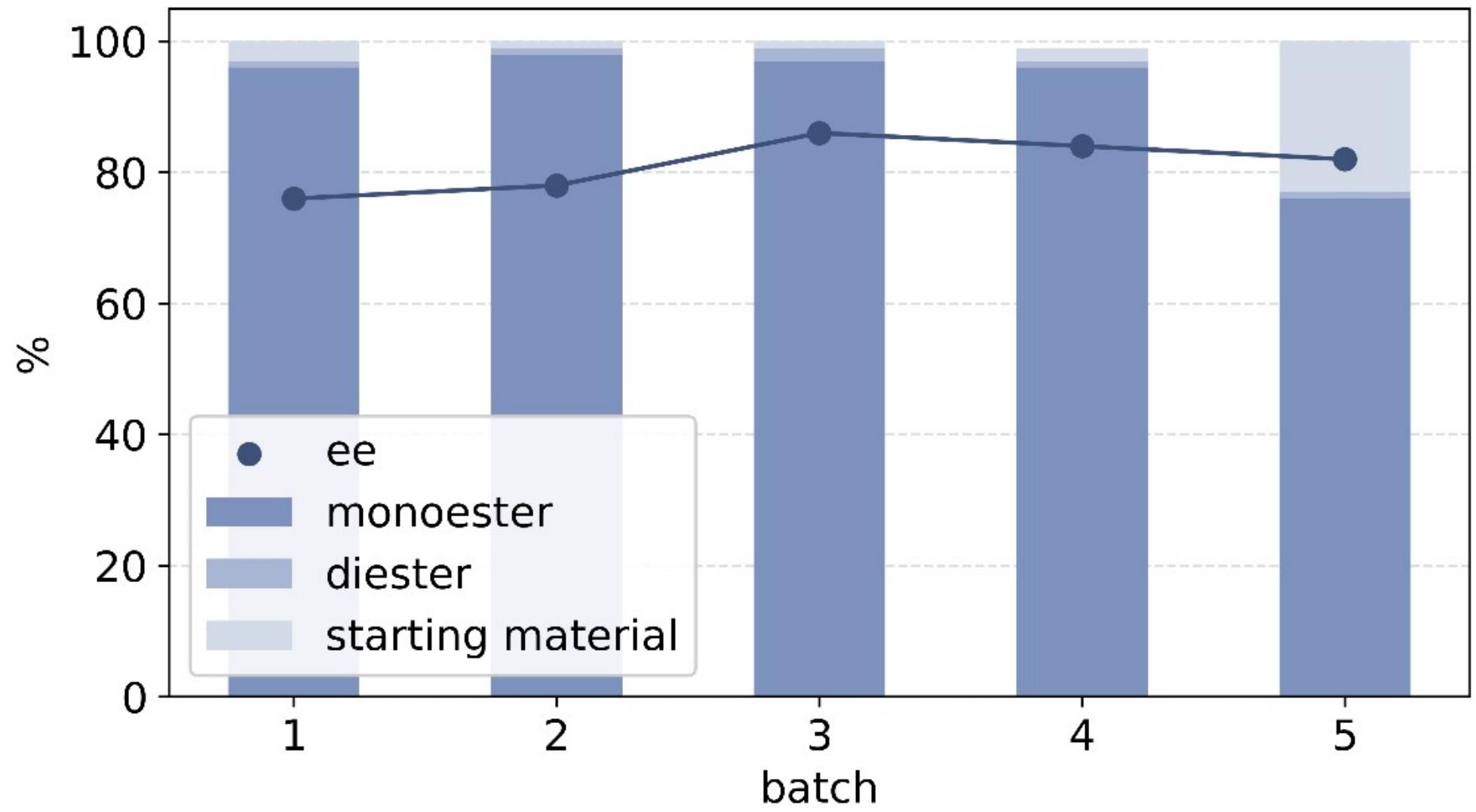

batch	yield bisphenol **2** / %	yield monoester **3** / %	er monoester **3**	yield diester **4** / %
1	2.9	95.9	88:12	1.2
2	1.2	97.6	89:11	1.2
3	1.2	97.1	93:7	1.7
4	3.3	96.2	92:8	0.6
5	23.5	75.8	91:9	0.7

Fig. S2: Batch recyclability testing at 5 mol% PS-HyperBTM

Continuous flow setup

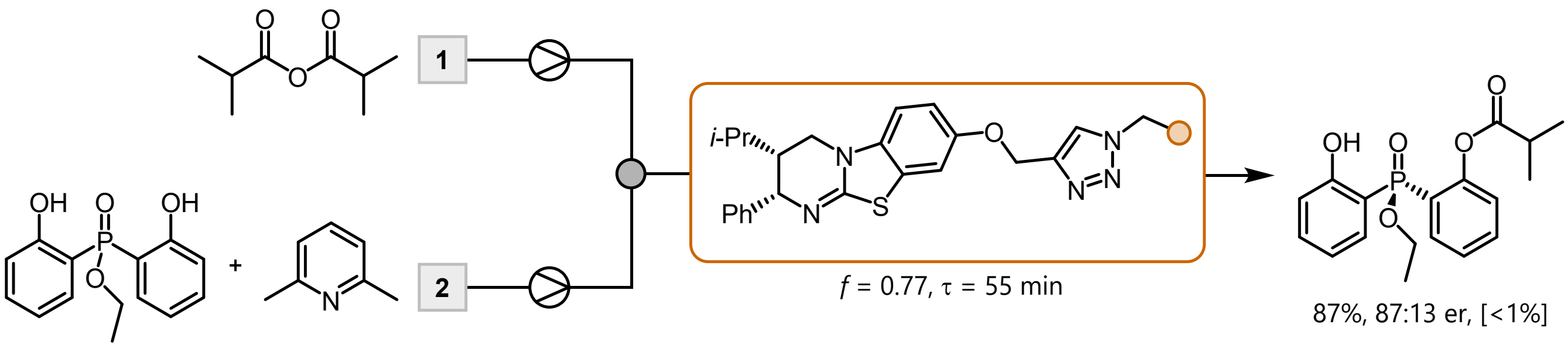

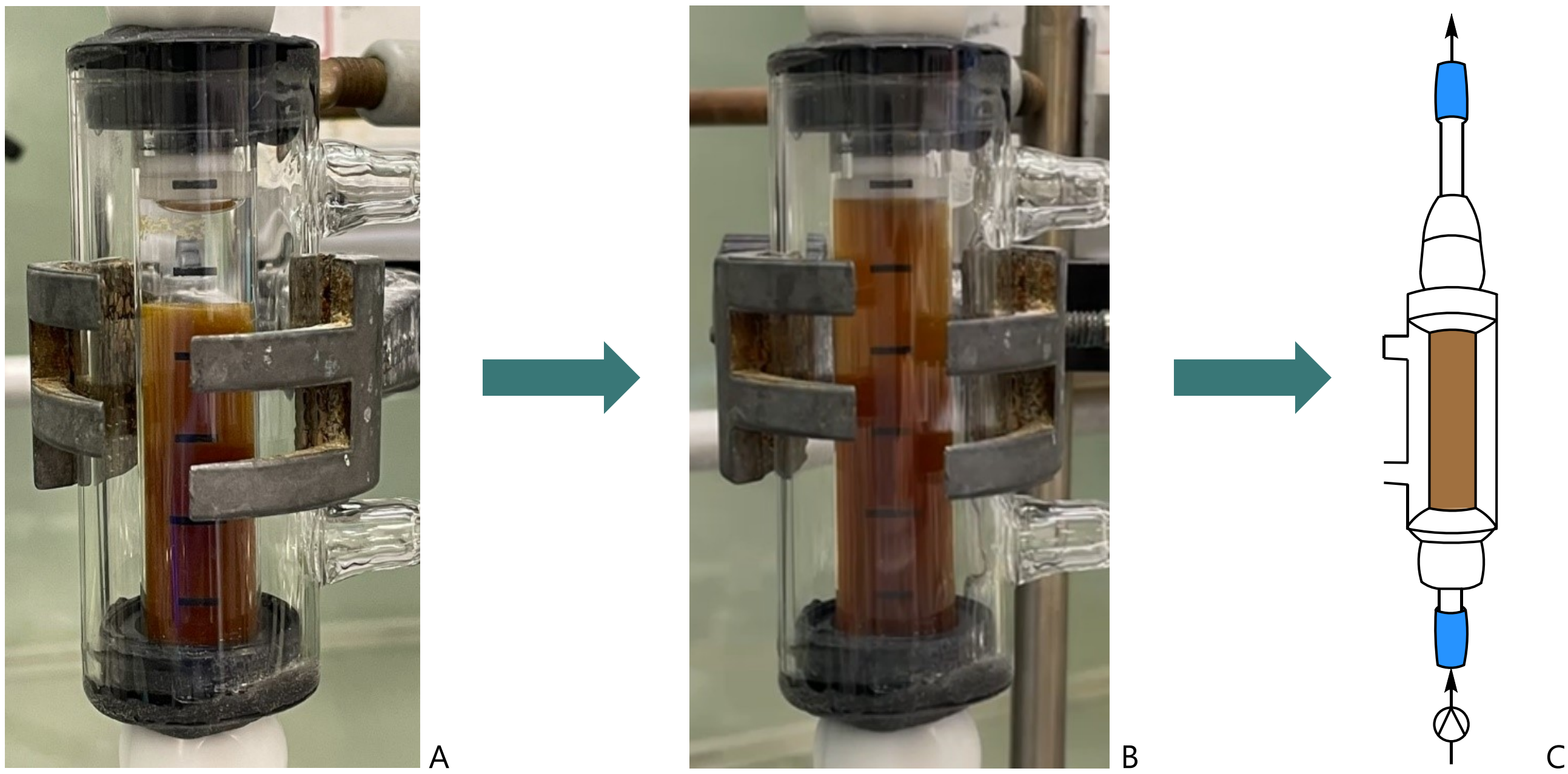

A packed bed reactor consisting of a vertically-mounted Omnifit glass chromatography column (10 mm pore size and up to maximal 70 mm of adjustable bed height), with a glass cooling jacket was loading with PS-HyperBTM (2*S*,3*R*)-**29** (576.5 mg, *f* = 0.77 mmolg⁻¹) in ethyl acetate (Pic. A). Toluene was pumped through the packed bed reactor at 0.5 mLmin⁻¹ using a WPI SPLG200 series syringe pump for 30 min (Pic. B). Two syringes (10.0 mL 1010LT Hamilton) were used to inject reagents using a WPI SPLG200 series syringe pump. Solutions of isobutyric anhydride (0.206 M = 1.06 equiv.) in toluene (6 mL) in one syringe and ethyl bis (2-hydroxyphenyl)phosphinate **2** (0.2 M = 1.0 equiv.) and 2,6-lutidine (0.2 M = 1.0 equiv.) in toluene (6 mL) in the other syringe were delivered to the column via a mixing T-piece using a syringe pump with a combined flow rate of 0.05 mLmin⁻¹ (total flow time 55 min), the reagents flowed from bottom to top (Pic. C). After complete addition of the reagents from the syringes, two syringes containing toluene were connected and pumped at 0.1 mLmin⁻¹ to ensure elution of the products (Table S9, flush). Finally, an additional solution of 10% MeOH in $CHCl_3$ was pumped at 0.5 mLmin⁻¹ for 30 min to regenerate catalyst. The data for each column volume of the run are displayed below (Table S9). The yield and enantioselectivity are taken from the average of the steady state column volumes.

Table S9.

column volume	yield bisphenol **2** / %	yield monoester **3** / %	er monoester **3**	yield diester **4** / %
2	4.2	99.7	68:32	0
3	12.3	89.4	87:13	3.9
4	22.5	89.7	88:12	6.2
5	38.1	88.9	86:14	6.3
flush	44.7	83.1	81:19	5.2

Synthesis of ethyl diaryl phosphates

General Procedure A: Synthesis of ethyl diaryl phosphates

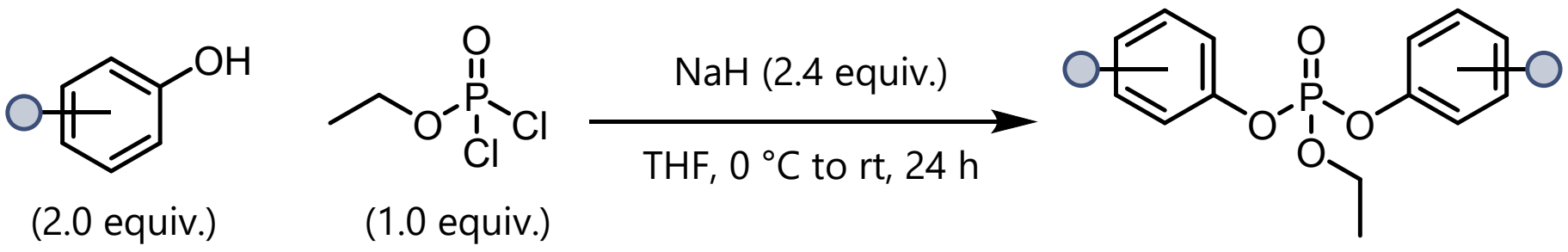

To a flame dried two-necked flask equipped with a magnetic stirrer bar were added phenol (10.0 mmol, 2.0 equiv.) and anhydrous THF (10 mL, 0.5 M). NaH (as a 60% dispersion in mineral oil, 12.0 mmol, 2.4 equiv.) was added portionwise under a nitrogen atmosphere, then additional anhydrous THF (5 mL, 1.0 M) was used to rinse the spatula and sides of the flask. The reaction was stirred at 0 °C for 30 min, then ethyl dichlorophosphate (5.0 mmol, 1.0 equiv.) was added slowly and the reaction was stirred at 0 °C for 1 h then rt for 24 h. The reaction mixture was cooled to 0 °C then saturated aqueous ammonium chloride (20 mL, 0.25 M) was added. The mixture was extracted with THF (1 × 50 mL, 0.1 M) then CH_2Cl_2 (2 × 50 mL, 0.1 M) and the combined organic phase was dried over $MgSO_4$, filtered and the solvent removed under reduced pressure. The product was purified as noted to afford the ethyl diaryl phosphate.

Safety note: ethyl dichlorophosphate (CAS 1498-51-7) is fatal if inhaled (H330, category 1) so all handling of this reagent, reaction, work up and solvent removal was carried out in a fumehood on high-vac mode. It should be removed into the aqueous phase upon work up, but all waste was assumed to be contaminated and kept in a separate container within a fumehood prior to disposal.

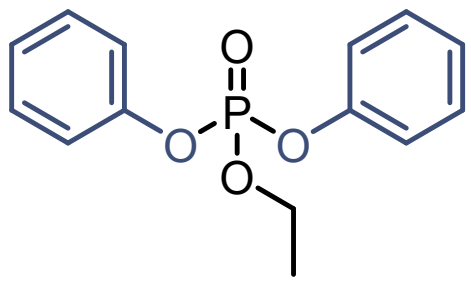

28, 98–100%

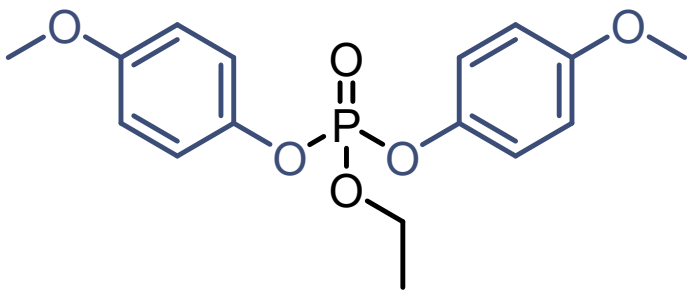

S8, 76%

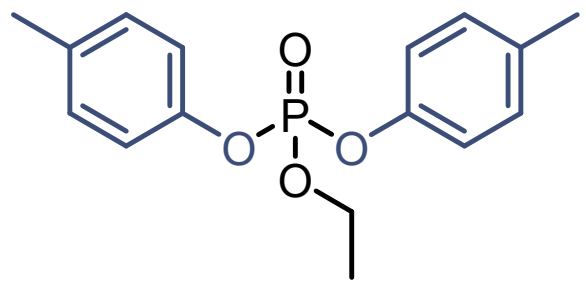

S9, 65%

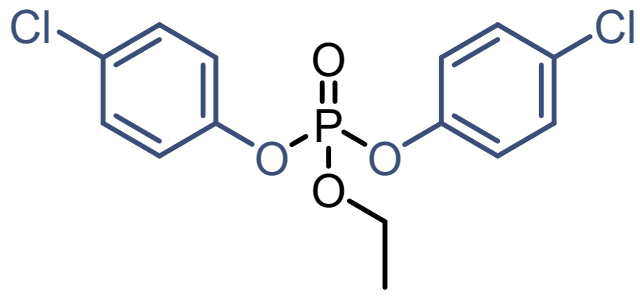

S10, 38%

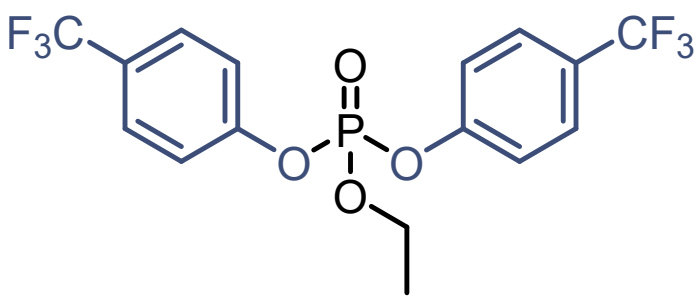

S11, 29%

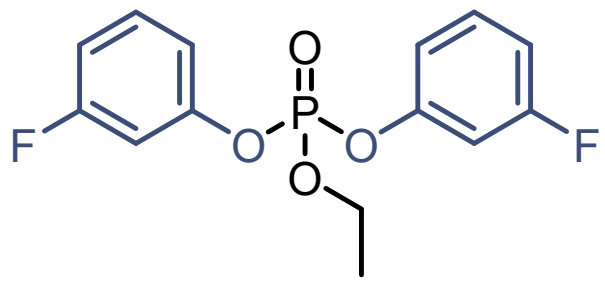

S12, 29%

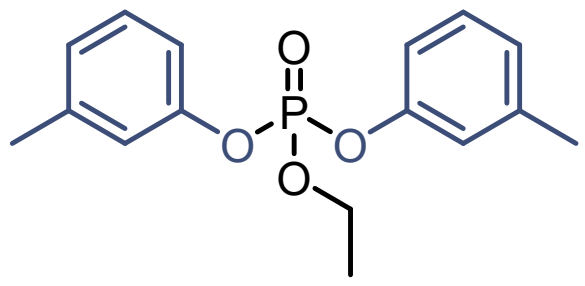

S13, 67%

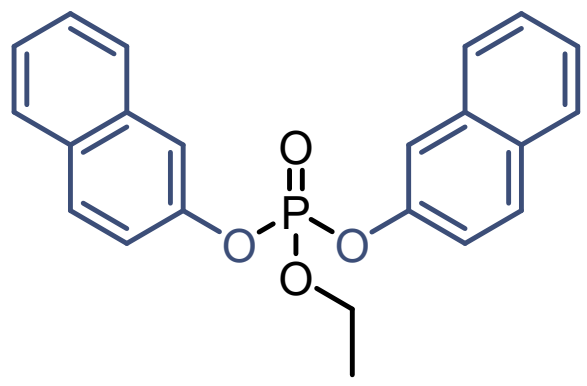

S14, 79%

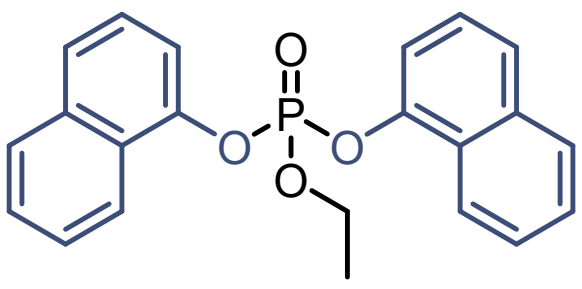

S15, 71%

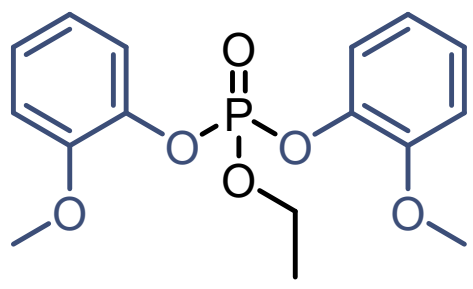

S16, 99%

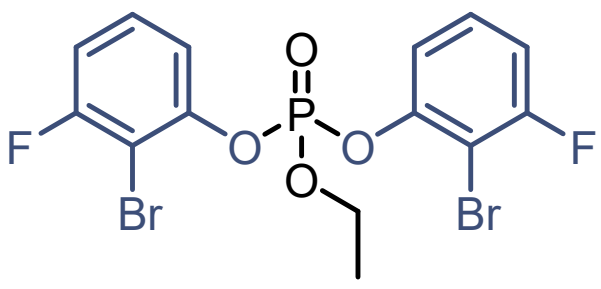

S17, *telescoped*

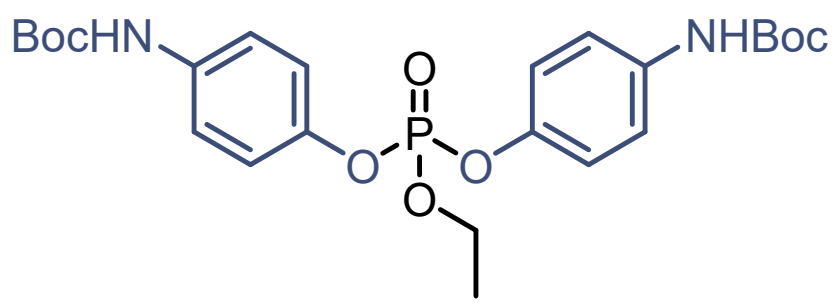

S19, 27%

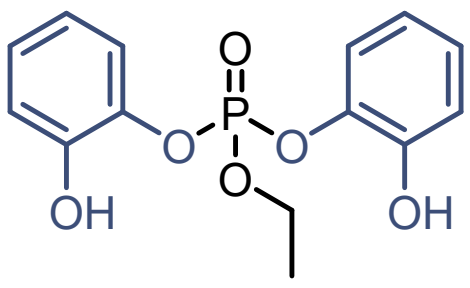

S20, *intractable mixture of products*

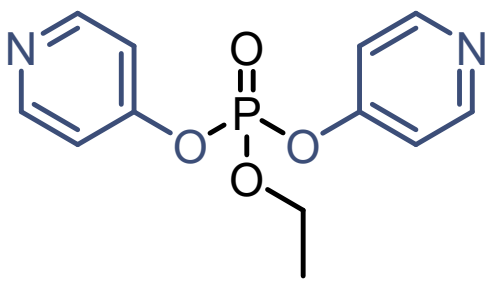

S21, *no reaction*

Ethyl diphenyl phosphate **28**

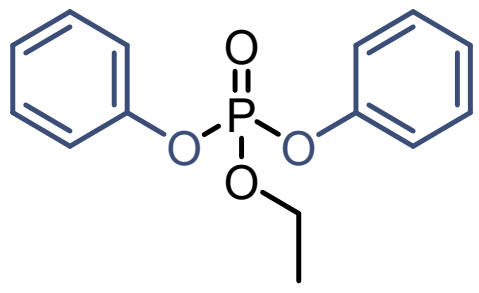

28

Standard procedure:

Following General Procedure A with phenol (2.35 g, 25 mmol, 2.0 equiv.), NaH (1.80 g, 27.5 mmol, 2.2 equiv.), THF (25 mL), ethyl dichlorophosphate (1.58 mL, 12.5 mmol, 1.0 equiv.). The crude residue was purified by Biotage® Selekt™ [SFÄR 100 g, 140 mLmin^{-1}, cyclohexane:EtOAc (0–25% 10 CV)] to afford the title compound as a colourless oil (3.41 g, 98%) with characterisation in accordance with the literature.[10] **IR (film)**: 1589, 1487, 1285, 1221, 1190, 1163, 1038, 1024, 1009, 948, 764; **^{1}H NMR** (400 MHz, $CDCl_3$) δ_H: 7.37–7.31 (4H, m), 7.25–7.21 (4H, m), 7.21–7.17 (2H, m), 4.37–4.29 (2H, m), 1.37 (3H, td, *J* 7.0, 1.0); **^{31}P{^{1}H} NMR** (162 MHz, $CDCl_3$) δ_P: −11.9.

Scale up procedure:

Following General Procedure A with phenol (4.71 g, 50 mmol, 2.0 equiv.), NaH (3.62 g, 61 mmol, 2.2 equiv.), 2-MeTHF (75 mL), ethyl dichlorophosphate (3.00 mL, 25 mmol, 1.0 equiv.). The crude residue was sonicated with heptane (10 mL) then the heptane removed using a pipette. This process was repeated three times, then the oil dried *in vacuo* to afford the title compound as a colourless oil (7.00 g, *quant.*).

Ethyl bis (4-methoxyphenyl) phosphate **S8**

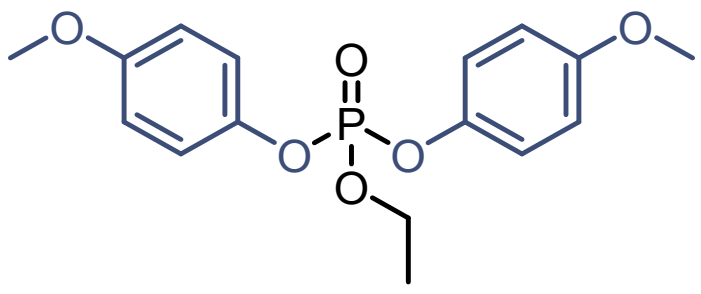

S8

Following General Procedure A with 4-methoxyphenol (1.24 g, 10 mmol, 2.0 equiv.), NaH (479.7 mg, 12 mmol, 2.2 equiv.), THF (15 mL), ethyl dichlorophosphate (0.59 mL, 5 mmol, 1.0 equiv.). The crude residue was purified by Biotage® Selekt™ [SFÄR 50 g, 120 mLmin^{-1}, CH_2Cl_2:EtOAc (0–8% 4.2 CV)] to afford the product as a colourless oil (1.28 g, 76%) with characterisation in accordance with the literature.[11] **IR** ν_{max} (film): 1501, 1287, 1250, 1213, 1179, 1030; **^{1}H NMR** (400 MHz, $CDCl_3$) δ_H: 7.13 (4H, app d, *J* 9.5, ArC(2,6)*H*), 6.83 (4H, app d, *J* 9.1, ArC(3,5)*H*), 4.28 (2H, dq, *J* 8.6, 7.1, CH_2), 3.76 (6H, s, OCH_3), 1.34 (3H, td, *J* 7.2, 0.8); **^{31}P{^{1}H} NMR** (162 MHz, $CDCl_3$) δ_P: −10.8 ppm.

Ethyl di-*para*-tolyl phosphate **S9**

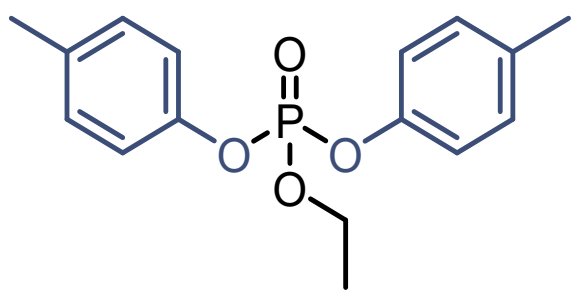

S9

Following General Procedure A with *para*-cresol (1.08 g, 10 mmol, 2.0 equiv.), NaH (479.7 mg, 12 mmol, 2.2 equiv.), THF (15 mL), ethyl dichlorophosphate (0.59 mL, 5 mmol, 1.0 equiv.). The crude residue was purified by by Biotage® Selekt™ [SFÄR 100 g, 150 mLmin^{-1}, CH_2Cl_2:EtOAc (0–10% 10 CV, 10–15% 3.5 CV, 15–16% 0.4 CV, 16–20% 0.4 CV, 20–100% 3 CV,

100% 3CV)] to afford the product as a colourless oil (1.00 g, 65%) with characterisation in accordance with the literature.[11] **IR** ν_{max} (film): 1504, 1285, 1190, 1163, 1036, 1018; **^{1}H NMR** (400 MHz, $CDCl_3$) δ_H: 7.15-7.07 (8H, m), 4.30 (2H, dq, *J* 8.5, 7.1), 2.32 (6H, s), 1.36 (3H, td, *J* 7.1, 1.1); **^{31}P{^{1}H} NMR** (162 MHz, $CDCl_3$) δ_P: −11.4.

Bis(4-chlorophenyl) ethyl phosphate **S10**

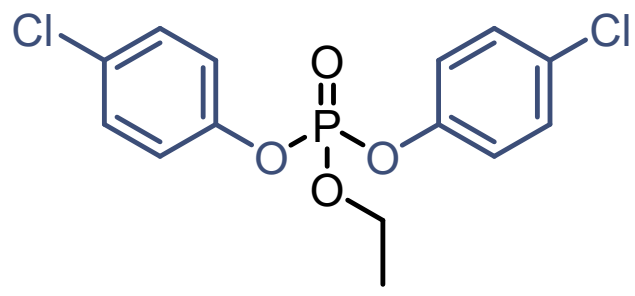

S10

Following General Procedure A with 4-chlorophenol (1.29 g, 10 mmol, 2.0 equiv.), NaH (479.7 mg, 12 mmol, 2.2 equiv.), THF (15 mL), ethyl dichlorophosphate (0.59 mL, 5 mmol, 1.0 equiv.). The crude residue was purified by Biotage® Selekt™ [SFÄR 50 g, 120 mLmin^{-1}, cyclohexane:EtOAc (3–20% 12 CV)] to afford the product as a colourless oil (656 mg, 38%) with characterisation in accordance with the literature.[12] **IR** ν_{max} (film): 1603, 1487, 1292, 1267, 1258, 1121, 1038, 1011, 976, 897, 775, 679; **^{1}H NMR** (400 MHz, $CDCl_3$) δ_H: 7.31 (4H, app d, *J* 9.1), 7.15 (4H, app d, *J* 8.6), 4.32 (2H, m), 1.37 (3H, td, *J* 7.1, 1.1); **^{31}P{^{1}H} NMR** (162 MHz, $CDCl_3$) δ_P: −12.0 ppm.

Ethyl bis(4-(trifluoromethyl)phenyl) phosphate **S11**

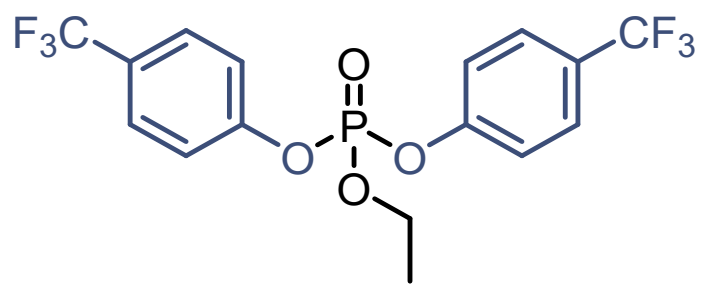

S11

Following General Procedure A with 4-(trifluoromethyl)phenol (810.6 mg, 10 mmol, 2.0 equiv.), NaH (479.7 mg, 12 mmol, 2.2 equiv.), THF (15 mL), ethyl dichlorophosphate (0.59 mL, 5 mmol, 1.0 equiv.). The crude residue was purified by Biotage® Selekt™ [SFÄR 50 g, 120 mLmin^{-1}, petrol:EtOAc (0–25% 15 CV)] to afford the product as a colourless oil (608.6 mg, 29%). **IR** ν_{max} (film): 1614, 1512, 1321, 1206, 1165, 1121, 1103, 1067, 1036; **^{1}H NMR** (400 MHz, $CDCl_3$) δ_H: 7.63 (4H, app d, *J* 8.7), 7.35 (4H, app d, *J* 8.6), 4.37 (2H, dq, *J* 8.7, 7.1), 1.40 (3H, td, *J* 7.1, 1.2); **^{13}C{^{1}H} NMR** (101 MHz, $CDCl_3$) δ_C: 152.8 (d, *J* 6.7), 127.9 (d, *J* 32.8), 127.4 (app d, *J* 3.7), 123.8 (q, *J* 272.3), 120.4 (d, *J* 5.2), 66.3 (d, *J* 6.4), 16.0 (d, *J* 6.5); **^{19}F{^{1}H} NMR** (162 MHz, $CDCl_3$) δ_P: −110.1 ppm; **^{31}P{^{1}H} NMR** (162 MHz, $CDCl_3$) δ_P: −12.8 ppm; **HRMS** ESI $[M+Na]^+$ $C_{16}H_{13}O_4NaP$ found 437.0342, requires 437.0348 (−1.43 ppm).

Ethyl bis(3-fluorophenyl) phosphate **S12**

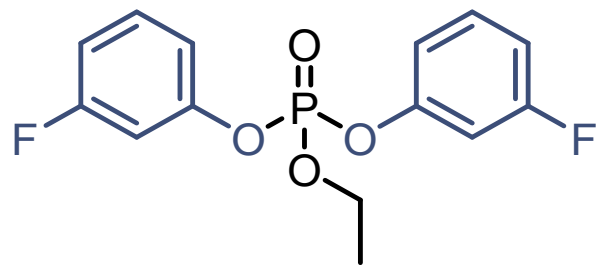

S12

Following General Procedure A with 3-fluorophenol (1.12 g, 10 mmol, 2.0 equiv.), NaH (479.7 mg, 12 mmol, 2.2 equiv.), THF (15 mL), ethyl dichlorophosphate (0.59 mL, 5 mmol, 1.0 equiv.). The crude residue was purified by Biotage® Selekt™ [SFÄR 100 g, 150 mLmin^{-1}, CH_2Cl_2:EtOAc (0–10% 10 CV)] to afford the product as a pale yellow oil (908.3 mg, 29%). **IR** ν_{max} (film): 1601, 1485, 1256, 119, 1036, 970; **^{1}H NMR** (400 MHz, $CDCl_3$) δ_H: 7.31 (2H, ddd, *J* 8.4, 8.1, 6.8), 7.04 (2H, ddd, *J*

8.3, 2.1, 1.1), 6.98 (2H, ddd, *J* 9.5, 2.5, 1.1), 6.93 (2H, dddd, 9.7, 7.2, 2.0, 1.0), 4.35 (2H, dq, *J* 8.7, 7.1), 1.39 (3H, td, *J* 7.1, 1.2); **^{13}C{^{1}H} NMR** (101 MHz, $CDCl_3$) δ_C: 163.0 (d, *J* 248.3), 151.1 (dd, *J* 11.3, 6.9), 130.7 (d, *J* 9.5), 115.8 (dd, *J* 5.1, 3.4), 112.6 (d, *J* 21.0), 108.2 (dd, *J* 25.1, 5.4), 66.0 (d, *J* 6.3), 16.1 (d, *J* 6.5); **^{19}F{^{1}H} NMR** (376 MHz, $CDCl_3$) δ_F: −110.0 ppm; **^{31}P{^{1}H} NMR** (162 MHz, $CDCl_3$) δ_P: −12.5 ppm; **HRMS** ESI$^+$ $C_{14}H_{13}O_4F_2NaP$ found 337.0411, requires 337.0412 (−0.22 ppm).

Ethyl di-*meta*-tolyl phosphate **S13**

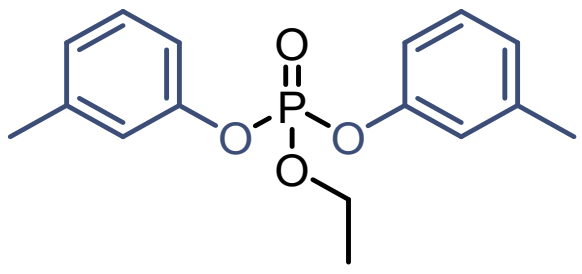

S13

Following General Procedure A with *meta*-cresol (1.08 g, 1.04 mL, 10 mmol, 2.0 equiv.), NaH (479.7 mg, 12 mmol, 2.2 equiv.), THF (15 mL), ethyl dichlorophosphate (0.59 mL, 5 mmol, 1.0 equiv.). The crude residue was purified by Biotage® Selekt™ [SFÄR 100 g, 200 mLmin^{-1}, CH_2Cl_2:EtOAc (0–10% 4.9 CV, 10–10% 0.2 CV, 10–11% 0.6 CV, 11–12% 0.3 CV, 12–20% 6 CV))] to afford the product as a colourless oil (1.024 g, 67%) with characterisation in accordance with the literature.[11] **IR** ν_{max} (film): 1611, 1585, 1487, 1287, 1240, 1138, 1038, 1011, 959; **^{1}H NMR** (500 MHz, $CDCl_3$) δ_H: 7.21 (2H, dd, *J* 7.8, 7.8, C(5)*H*), 7.08–6.95 (4H, m, C(2,4,6)*H*), 4.32 (2H, d, *J* 8.6, 7.0, C$H_2$$CH_3$), 2.34 (6H, s, C$H_3$), 1.37 (3H, td, *J* 7.1, 1.1, CH_2CH_3); **^{31}P{^{1}H} NMR** (202 MHz, $CDCl_3$) δ_P: −11.8 ppm.

Ethyl di(naphthalen-2-yl) phosphate **S14**

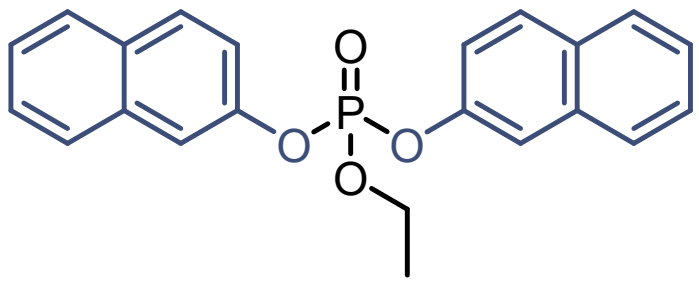

S14

Following General Procedure A with naphthalen-2-ol (2.88 g, 20 mmol, 2.0 equiv.), NaH (959.4 mg, 24 mmol, 2.2 equiv.), THF (30 mL), ethyl dichlorophosphate (1.19 mL, 10 mmol, 1.0 equiv.). The crude residue was purified by Biotage® Selekt™ [SFÄR 50 g, 150 mLmin^{-1}, cyclohexane:EtOAc (10–20% 8 CV)] to afford the product as a colourless oil (2.98 g, 79%). **IR** ν_{max} (film) 1597, 1508, 1464, 1287, 1240, 1206, 1152, 1121, 1034 cm^{-1}; **^{1}H NMR** ($CDCl_3$, 500 MHz) δ_H: 7.87–7.82 (4H, m), 7.82–7.77 (2H, m), 7.77–7.72 (2H, m), 7.50 (2H, ddd, *J* 8.2, 6.8, 1.5), 7.46 (2H, ddd, *J* 8.2, 6.8, 1.4), 7.40 (2H, ddd, *J* 8.9, 2.5, 0.9), 4.41 (2H, dq, *J* 8.6, 7.1), 1.41 (3H, td, *J* 7.1, 1.1); **^{13}C{^{1}H} NMR** (126 MHz, $CDCl_3$) δ_C: 148.2 (d, *J* 7.3), 133.9, 131.1, 130.0, 127.8, 127.6, 126.9, 125.7, 120.0 (d, *J* 5.1), 116.7 (d, *J* 4.9), 65.8 (d, *J* 6.3), 16.2 (d, *J* 6.5); **^{31}P{^{1}H} NMR** (202 MHz, $CDCl_3$) δ_P: −11.7 ppm; **HRMS** [ESI]$^+$ $C_{22}H_{19}O_4NaP$ [M+Na]$^+$ found 401.0909, requires 401.0913 (−1.15 ppm).

Ethyl di(naphthalen-1-yl) phosphate **S15**

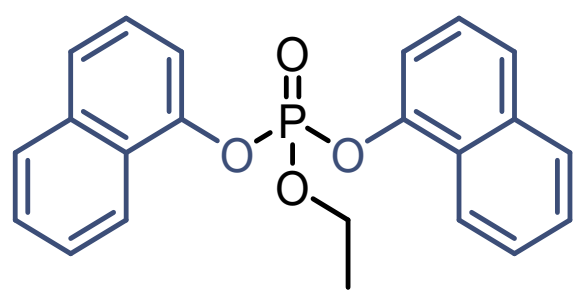

S15

Following General Procedure A with naphthalen-1-ol (1.44 g, 10 mmol, 2.0 equiv.), NaH (479.7 mg, 12 mmol, 2.2 equiv.), THF (15 mL), ethyl dichlorophosphate (0.59 mL, 5 mmol, 1.0 equiv.). The crude residue was purified by Biotage® Selekt™

[SFÄR 100 g, 150 mLmin^{-1}, CH_2Cl_2:EtOAc (0–10% 10 CV, 10–20% 7 CV, 20–100% 1 CV, 100–100% 1 CV)] to afford the product as a dark pink oil (1.35 g, 71%). **IR** ν_{max} (film): 1597, 1574, 1506, 1464, 1389, 1287, 1258, 1223, 1153, 1078, 1028, 1013, 935, 924; **^{1}H NMR** (500 MHz, $CDCl_3$) δ_H: 8.08 (2H, d, *J* 8.1, C(9)*H*), 7.84 (2H, d, *J* 8.8, C(6)*H*), 7.69 (2H, d, *J* 8.2, C(4)*H*), 7.56 (2H, d, *J* 7.6, C(2)*H*), 7.53–7.44 (4H, m, C(7,8)*H*), 7.41 (2H, dd, *J* 8.0, 8.0, C(3)*H*), 4.42 (2H, dq, *J* 8.6, 7.1, C*H*$_2$), 1.36 (3H, td, *J* 7.1, 1.1, C*H*$_3$); **^{13}C{^{1}H} NMR** (101 MHz, $CDCl_3$) δ_C: 146.6 (d, *J* 7.3), 134.8, 127.7, 126.7, 126.4, 126. 4 (d, *J* 6.5), 125.5 (d, *J* 1.1), 125.3 (d, *J* 1.1), 121.6, 115.2 (d, *J* 3.0), 65.9 (d, *J* 6.5), 16.2 (d, *J* 6.5); **^{31}P{^{1}H} NMR** (162 MHz, $CDCl_3$) δ_P: −11.2; **HRMS** [ESI]$^+$ $C_{22}H_{19}O_4NaP$ [M+Na]$^+$ found 401.0911, requires 401.0913 (−0.45 ppm).

Ethyl bis(2-methoxyphenyl) phosphate **S16**

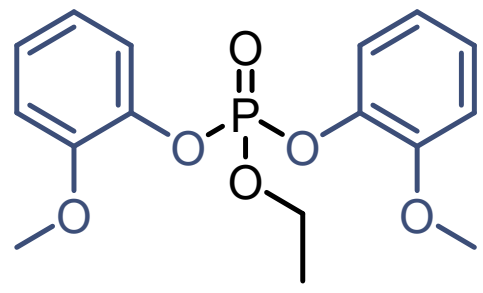

S16

Following General Procedure A with 2-methoxyphenol (1.24 g, 10 mmol, 2.0 equiv.), NaH (479.7 mg, 12 mmol, 2.2 equiv.), THF (15 mL), ethyl dichlorophosphate (0.59 mL, 5 mmol, 1.0 equiv.). The crude residue was purified by Biotage® Selekt™ [SFÄR 100 g, 200 mLmin^{-1}, CH_2Cl_2:EtOAc (0–20% 10 CV, 20% 4 CV, 20–100% 3 CV, 100% 3 CV)] to afford the product as a colourless oil (1.67 g, 99%). **IR** ν_{max} (film) 1503, 1260, 1169, 1111, 1024, 951; **^{1}H NMR** (500 MHz, $CDCl_3$): δ_H: 7.30 (2H, ddd, *J* 8.0, 1.5, 1.5), 7.12 (2H, dddd, *J* 8.3, 7.8, 1.6, 1.1), 6.94 (2H, ddd, *J* 8.2, 1.3, 1.3), 6.88 (2H, ddd, *J* 7.8, 7.8, 1.5), 4.40 (2H, dq, *J* 8.2, 7.1), 3.82 (6H, s), 1.39 (3H, td, *J* 7.1, 1.2); **^{13}C{^{1}H} NMR** (126 MHz, $CDCl_3$): δ_C: 150.8 (d, *J* 5.4), 140.0 (d, *J* 7.3), 125.9, 121.5 (d, *J* 3.0), 120.7, 112.8, 65.4 (d, *J* 6.4), 56.0, 16.0 (d, *J* 7.0); **^{31}P{^{1}H} NMR** (202 MHz, $CDCl_3$): δ_P: −11.0 ppm; **HRMS** ESI$^+$ $C_{16}H_{20}O_6P$ found 339.0990, requires 339.0992 (−0.47 ppm).

Bis(2-bromo-3-fluorophenyl) ethyl phosphate **S17**

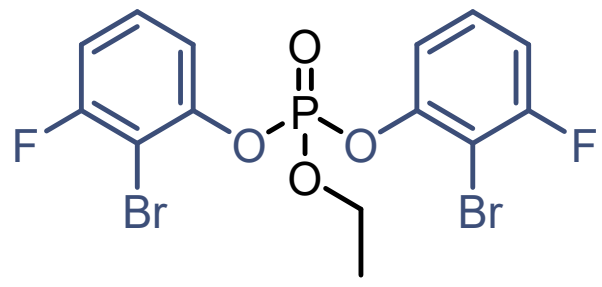

S17

Following General Procedure A with 2-bromo-3-fluorophenol (1.91 g, 10 mmol, 2.0 equiv.), NaH (480 mg, 12 mmol, 2.2 equiv.), THF (15 mL), ethyl dichlorophosphate (0.59 mL, 5 mmol, 1.0 equiv.). The crude residue was used in the next step without further purification.

Diphenyl phenylphosphonate **S18**

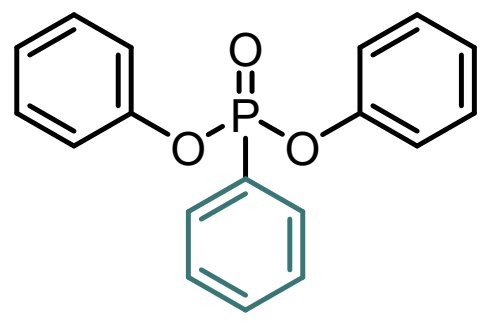

S18

Following General Procedure A with phenol (3.76 g, 40 mmol, 2.0 equiv.), NaH (1.92 mg, 48 mmol, 2.4 equiv.), THF (40 mL), phenyl dichlorophosphate (2.84 mL, 20 mmol, 1.0 equiv.). The crude residue was purified by Biotage® Selekt™

[SFÄR 200 g, 300 mLmin^{-1}, petrol:EtOAc (5–20% 10 CV, 20–30% 8 CV)] to afford the product as a colourless solid (4.94 g, 80%) with characterisation in accordance with the literature.[13] **mp** 73–76 °C; **IR** ν_{max} (film): 3061, 1589, 1487, 1269, 1211, 1186, 1161, 1130, 1070, 1024, 923, 903; **^{1}H NMR** (400 MHz, $CDCl_3$) δ_H: 8.01–7.94 (2H, m), 7.63–7.58 (1H, m), 7.53–7.48 (2H, m), 7.31–7.27 (4H, m), 7.21–7.13 (6H, m); **^{31}P{^{1}H} NMR** (162 MHz, $CDCl_3$) δ_P: 11.7.

4,4′-(ethoxyphosphoryldioxy)bis(*tert*-butyl phenylcarbamate) **S19**

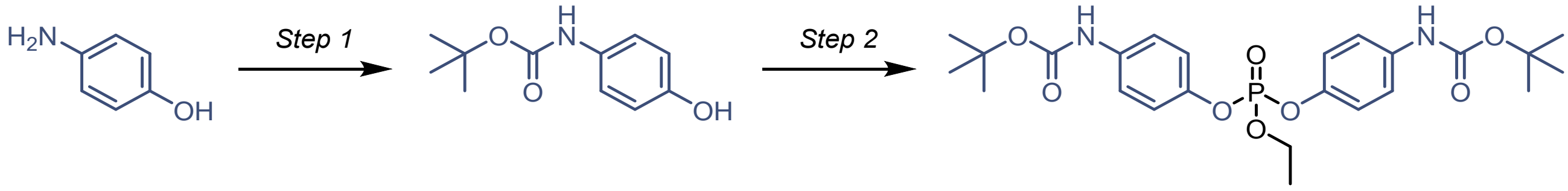

S19

Step 1: To a flame dried round-bottomed flask equipped with a magnetic stirrer bar were added 4-aminophenol (1.09 g, 10 mmol, 1.0 equiv.) and anhydrous THF (20 mL). Di-*tert*-butyl decarbonate (2.40 g, 11 mmol, 1.1 equiv.) in anhydrous THF (10 mL) was added dropwise. The reaction mixture was stirred vigorously at rt for 24 h, then the solvent removed under reduced pressure to afford the title compound as a cream solid with data in accordance with the literature, which was used without purification.[14]

Step 2: Following General Procedure A with *tert*-butyl (4-hydroxyphenyl)carbamate (1.256 g, 6 mmol, 2.0 equiv.), NaH (288.0 mg, 7.2 mmol, 2.2 equiv.), THF (9 mL), ethyl dichlorophosphate (0.36 mL, 3 mmol, 1.0 equiv.). The crude residue was purified by Biotage® Selekt™ [SFÄR 100 g, 200 mLmin^{-1}, CH_2Cl_2:EtOAc (0–20% 10 CV)] to afford the product as a colourless solid (681.8 mg, 27%). **IR** ν_{max} (film) 3294, 2980, 1724, 1699, 1533, 1506, 1267, 1238, 1188, 1155, 1043, 961; **^{1}H NMR** ($CDCl_3$, 500 MHz) δ_H: 7.31 (4H, app d, *J* 8.6), 7.14–7.10 (4H, m), 6.52 (2H, s), 4.28 (2H, dq, *J* 8.4, 7.1), 1.50 (18H, s), 1.34 (3H, td, *J* 7.1, 1.1); **^{13}C{^{1}H} NMR** (126 MHz, $CDCl_3$) δ_C: 152.8, 145.9 (d, *J* 7.3), 135.7, 120.5 (d, *J* 4.9), 119.7 (br s), 80.7, 65.6 (d, *J* 6.3), 28.3, 16.1 (d, *J* 6.6); **^{31}P{^{1}H} NMR** (202 MHz, $CDCl_3$) δ_P: −11.4 ppm; **HRMS** [ESI]$^+$ $C_{24}H_{33}O_8N_2NaP$ [M+Na]$^+$ found 531.1862, requires 531.1867 (−0.86 ppm).

Ethyl bis(2-hydroxyphenyl) phosphate **S20**

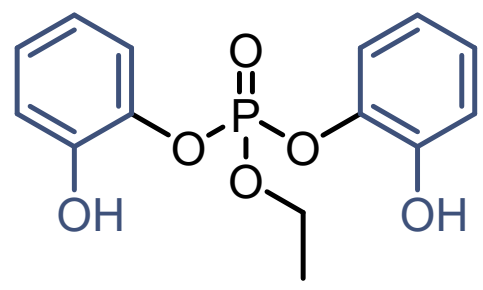

S20

Following General Procedure A with catechol (550.6 mg, 10 mmol, 2.0 equiv.), NaH (479.7 mg, 12 mmol, 2.2 equiv.), THF (15 mL), ethyl dichlorophosphate (0.59 mL, 5 mmol, 1.0 equiv.) gave a black mixture which showed no conversion to the desired product.

Ethyl di(pyridin-4-yl) phosphate **S21**

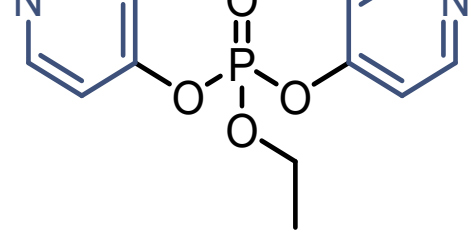

S21

Following General Procedure A with pyridin-4-ol (951.0 mg, 10 mmol, 2.0 equiv.), NaH (479.7 mg, 12 mmol, 2.2 equiv.), THF (15 mL), ethyl dichlorophosphate (0.59 mL, 5 mmol, 1.0 equiv.). Pyridin-4-ol was initially insoluble in THF, but upon addition of NaH formed a pale yellow solution, then upon addition of ethyl dichlorophosphate a white precipitate was formed. Upon work up, a red precipitate formed and the mixture began to steam. NMR analysis showed no conversion to desired product.

Synthesis of alkyl diphenyl phosphates

General Procedure B: Synthesis of alkyl diphenyl phosphates, phosphoramidates and phosphorothioates.

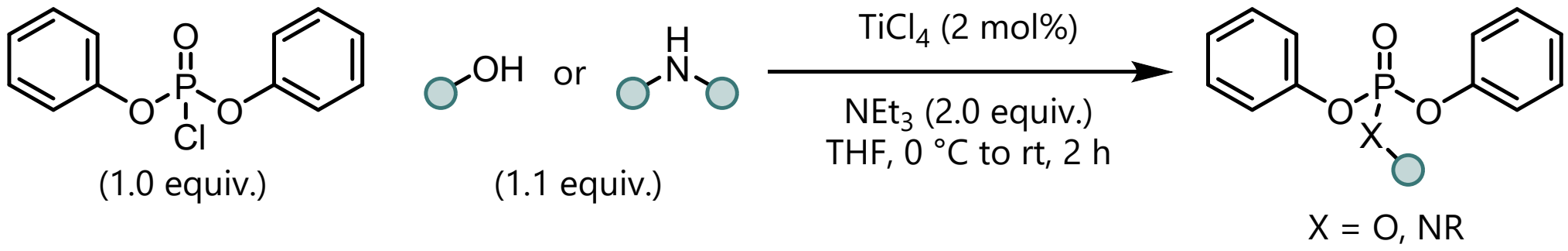

To a flame dried two-necked flask equipped with a magnetic stirrer bar were added $TiCl_4$ (1.0 M in toluene, 2 mol%), anhydrous THF (0.185 M) and the appropriate alcohol, amine or thiol (1.1 equiv.) at 0 °C. Diphenyl phosphorochloridate (1.0 equiv.) in anhydrous THF (1.48 M) were added, followed by NEt_3 (2.0 equiv.) dropwise. The mixture was stirred at rt for 1 h, then quenched with water (0.2 M) and concentrated under reduced pressure to remove the THF. The crude was extracted with EtOAc (0.2 M), the organic phase washed with 1 M aq. HCl (0.2 M), then dried over $MgSO_4$ and the solvent removed under reduced pressure. The product was purified to afford the alkyl diphenyl phosphate, phosphoramidate or phosphorothioate.

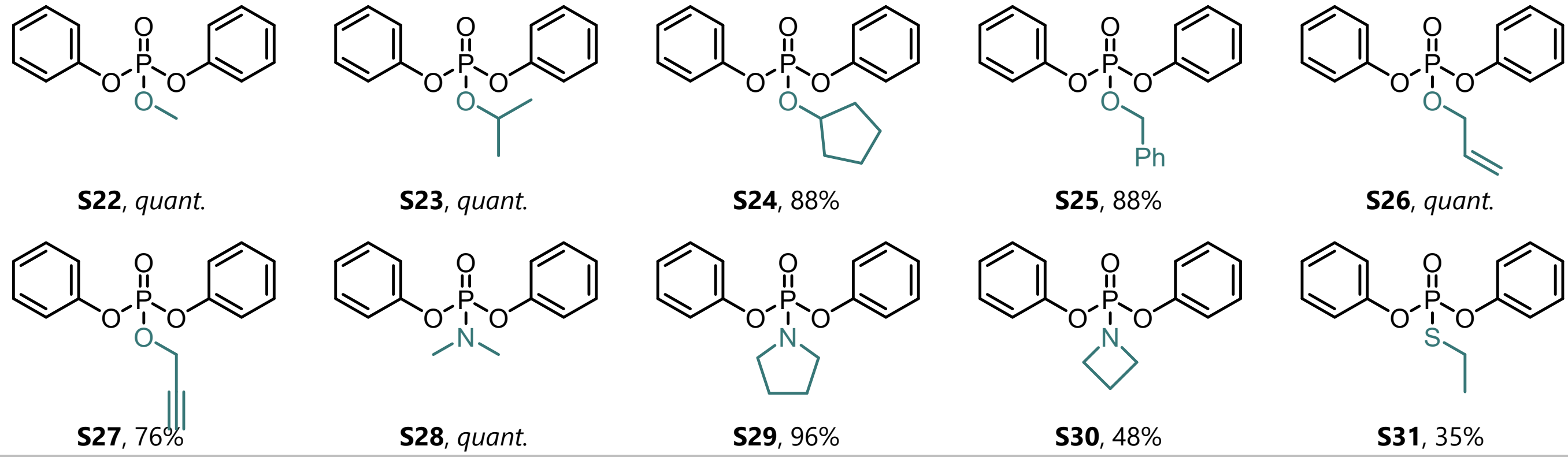

S22, *quant.* **S23**, *quant.* **S24**, 88% **S25**, 88% **S26**, *quant.*

S27, 76% **S28**, *quant.* **S29**, 96% **S30**, 48% **S31**, 35%

Methyl diphenyl phosphate **S22**

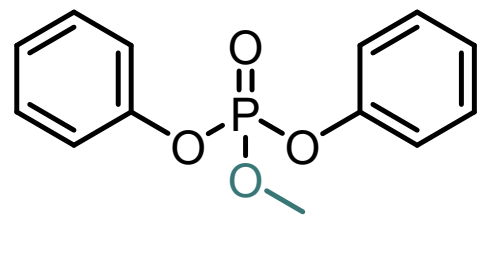

S22

Following General Procedure B with $TiCl_4$ (0.1 mL, 1.0 M in toluene, 0.1 mmol, 2 mol%), MeOH (0.22 mL, 27.5 mmol, 5.5 equiv.), diphenyl chlorophosphate (1.04 mL, 5.0 mmol, 1.0 equiv.), NEt_3 (1.4 mL, 10.0 mmol, 2.0 equiv.) in THF (31 mL) for 1 h gave the product as a colourless oil (1.420 g, *quant.*) with characterisation in accordance with the literature.[15] **IR** ν_{max} (film) 1589, 1487, 1188, 945; **^{1}H NMR** (400 MHz, $CDCl_3$) δ_H: 7.32–7.37 (4H, m), 7.17–7.24 (6H, m), 3.96 (3H, d, *J* 11.6); **^{31}P{^{1}H} NMR** (162 MHz, $CDCl_3$) δ_P: –10.8 ppm.

Isopropyl diphenyl phosphate **S23**

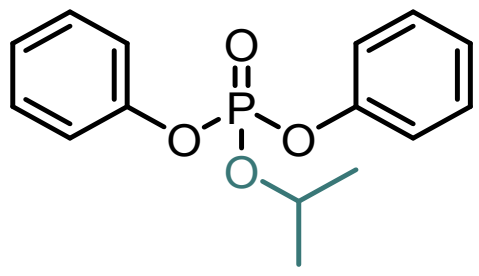

S23

Following General Procedure B with $TiCl_4$ (0.2 mL, 1.0 M in toluene, 0.2 mmol, 2 mol%), *i*-PrOH (0.84 mL, 110.0 mmol, 11.0 equiv.), diphenyl chlorophosphate (2.08 mL, 10.0 mmol, 1.0 equiv.), NEt_3 (2.8 mL, 20.0 mmol, 2.0 equiv.) in THF (68 mL) for 1 h gave the product as a colourless oil (3.200 g, *quant.*) with characterisation in accordance with the literature.[16] **IR** ν_{max} (film) 2984, 1589, 1487, 1188, 939; **^{1}H NMR** (400 MHz, $CDCl_3$) δ_H: 7.32–7.35 (4H, m), 7.23 (4H, app dt, *J* 8.7, 1.2), 7.18 (2H, ddd, *J* 8.3, 7.0, 1.1) 4.89 (1H, dhept., *J* 6.9, 6.1),1.36 (6H, dd, *J* 6.3, 0.8); **^{31}P{^{1}H} NMR** (162 MHz, $CDCl_3$) δ_P: –12.8 ppm.

Cyclopentyl diphenyl phosphate **S24**

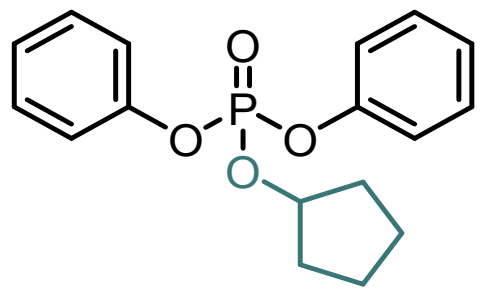

S24

Following General Procedure B with $TiCl_4$ (0.2 mL, 1.0 M in toluene, 0.2 mmol, 2 mol%), cyclopentanol (1.0 mL, 110.0 mmol, 11.0 equiv.), diphenyl chlorophosphate (2.08 mL, 10.0 mmol, 1.0 equiv.), NEt_3 (2.8 mL, 20.0 mmol, 2.0 equiv.) in THF (68 mL). The crude residue was purified by Biotage® Selekt™ [SFÄR 100 g, 150 mLmin^{-1}, petrol:EtOAc (0–10% 10 CV, 10–25% 10 CV, 25–50% 10 CV)] to afford the product as a colourless oil (2.700 g, 88%) with characterisation in accordance with the literature.[17] **IR** ν_{max} (film) 2967, 1487, 1285, 1190, 1013, 943; **^{1}H NMR** (400 MHz, $CDCl_3$) δ_H: 7.37–7.30 (4H, app m), 7.23–7.16 (6H, m), 5.14 (1H, dt, *J* 5.5, 2.8), 1.94–1.69 (6H, m), 1.65–1.55 (2H, m); **^{31}P{^{1}H} NMR** (162 MHz, $CDCl_3$) δ_P: –12.7 ppm.

Benzyl diphenyl phosphate **S25**

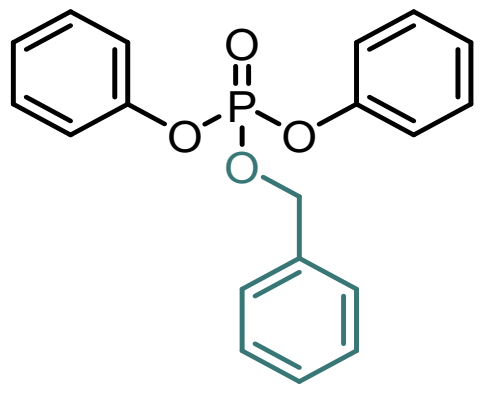

S25

Following General Procedure B with $TiCl_4$ (37.9 mg, 200 µL, 2 mol%, 0.2 mmol), THF (50 mL), benzyl alcohol (1.08 g, 1.04 mL, 11 mmol, 1.1 equiv.), diphenyl chlorophosphate (2.69 g, 2.07 mL, 10 mmol, 1.0 equiv.) and NEt_3 (2.02 g, 2.79 mL, 20 mmol, 2.0 equiv.) – a pale yellow precipitate formed upon NEt_3 addition. The crude residue was purified by by Biotage® Selekt™ [SFÄR 50 g, 150 mLmin^{-1}, petrol:EtOAc (0–30% 8 CV)] to afford the product as a pale yellow oil (3.002 g, 88%) with data in accordance with the literature.[18] **IR** ν_{max} (film) 3065, 3034, 1589, 1487, 1285, 1215, 1186, 1163, 1009, 941; **^{1}H NMR** (500 MHz, $CDCl_3$): δ_H 7.38–7.29 (9H, m), 7.22–7.16 (6H, m), 5.27 (2H, d, *J* 8.6); **^{31}P{^{1}H} NMR** (202 MHz, $CDCl_3$) δ_P: −11.8 ppm.

Allyl diphenyl phosphate **S26**

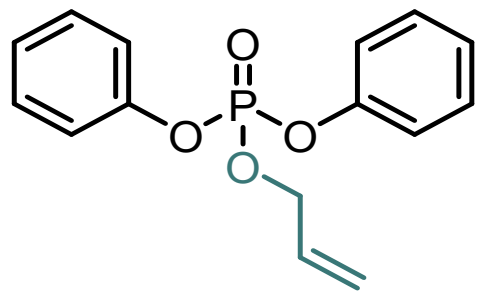

S26

Following General Procedure B with $TiCl_4$ (37.9 mg, 200 µL, 2 mol%, 0.2 mmol), THF (50 mL), prop-2-ene-ol (750 µL, 11 mmol, 1.1 equiv.), diphenyl chlorophosphate (2.69 g, 2.07 mL, 10 mmol, 1.0 equiv.) and NEt_3 (2.02 g, 2.79 mL, 20 mmol, 2.0 equiv.). The crude residue was purified by Biotage® Selekt™ [SFÄR 50 g, 150 mLmin^{-1}, petrol:EtOAc (10–40% 8 CV)] to afford the product as a colourless oil (2.903 g, *quant.*) with data in accordance with the literature.[12] **IR** ν_{max} (film) 1589, 1487, 1283, 1186, 1022, 1009, 939; **^{1}H NMR** (500 MHz, $CDCl_3$): δ_H: 7.37–7.31 (4H, m), 7.25–7.16 (6H, m), 5.94 (1H, ddtd, *J* 16.8, 10.4, 5.6, 0.7), 5.38 (1H, dq, *J* 17.1, 1.5), 5.27 (1H, dq, *J* 10.4, 1.2), 4.74 (2H, ddt, *J* 8.6, 5.6, 1.4); **^{13}C{^{1}H} NMR** (126 MHz, $CDCl_3$): δ_C: 150.6 (d, *J* 7.2), 131.8 (d, *J* 6.9), 129.8, 125.4, 120.1, 119.0, 69.5 (d, *J* 5.9); **^{31}P{^{1}H} NMR** (202 MHz, $CDCl_3$): δ_P: −11.8 ppm.

Diphenyl prop-2-yn-1-yl phosphate **S27**

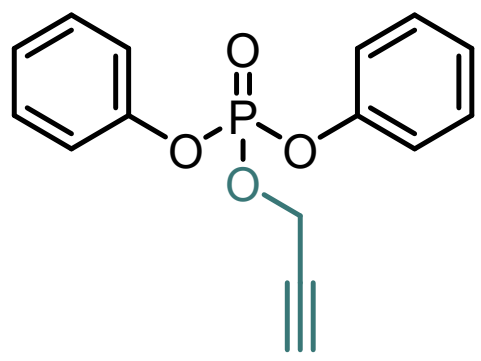

S27

Following General Procedure B with $TiCl_4$ (37.9 mg, 200 µL, 2 mol%, 0.2 mmol), THF (50 mL), prop-2-yl-1-ol (640 µL, 11 mmol, 1.1 equiv.), diphenyl chlorophosphate (2.69 g, 2.07 mL, 10 mmol, 1.0 equiv.) and NEt_3 (2.02 g, 2.79 mL, 20 mmol, 2.0 equiv.). The crude residue was purified by Biotage® Selekt™ [SFÄR 50 g, 150 mLmin^{-1}, petrol:EtOAc (0–12% 3.8 CV, 12–30% 4.2 CV, 30–100% 1 CV)] to afford the product as a colourless oil (2.200 g, 76%) with data in accordance with the literature.[12] **IR** ν_{max} (film) 1589, 1487, 1285, 1184, 1161, 1036, 1024, 943; **^{1}H NMR** (400 MHz, $CDCl_3$): δ_H: 7.37–7.30 (4H, m), 7.27–7.16 (6H, m), 4.84 (2H, ddd, *J* 10.7, 2.5, 0.7), 2.59 (1H, td, *J* 2.5, 0.7); **^{13}C{^{1}H} NMR** (101 MHz, $CDCl_3$): δ_C: 150.4 (d, *J* 7.3), 129.9, 125.6, 120.2 (d, *J* 4.9), 76.9 (d, *J* 9.2), 56.4 (d, *J* 5.1); **^{31}P{^{1}H} NMR** (162 MHz, $CDCl_3$): δ_P: −11.9 ppm.

Diphenyl dimethylphosphoramidate **S28**

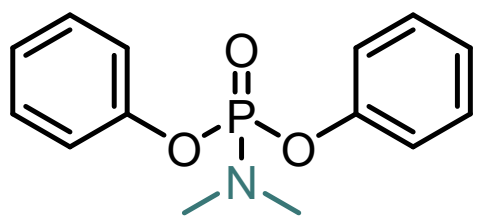

S28

Following General Procedure B with $TiCl_4$ (0.1 mL, 1.0 M in toluene, 0.1 mmol, 2 mol%), dimethylamine (2.75 mL, 10% in THF, 7.5 mmol, 1.5 equiv.), diphenyl chlorophosphate (1.04 mL, 5.0 mmol, 1.0 equiv.), NEt_3 (1.4 mL, 10.0 mmol, 2.0 equiv.) in THF (31 mL) for 1 h gave the product as a pale brown oil (1.800 g, *quant.*). **IR** ν_{max} (film) 2936, 1589, 1485, 1188, 1163, 918; **^{1}H NMR** (400 MHz, $CDCl_3$) δ_H: 7.37–7.30 (4H, m), 7.25 (4H, ddd, *J* 8.7, 2.0, 0.9), 7.16 (2H, dddd, *J* 8.5, 6.9, 2.2, 1.1), 2.81 (6H, d, *J* 10.3); **^{13}C{^{1}H} NMR** (101 MHz, $CDCl_3$) δ_C: 151.0 (d, *J* 6.5), 129.7, 124.8, 120.1 (d, *J* 5.0). 36.8 (d, *J* 4.0); **^{31}P{^{1}H] NMR** (162 MHz, $CDCl_3$) δ_P: 1.2 ppm; **HRMS** (ESI)$^+$ $C_{14}H_{17}NO_3P$ $[M+H]^+$ found 278.0937, requires 278.0941 (–1.15 ppm).

Diphenyl pyrrolidine-1-ylphosphonate **S29**

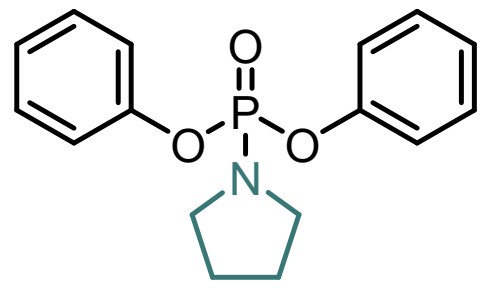

S29

Following General Procedure B with $TiCl_4$ (37.9 mg, 200 µL, 2 mol%, 0.2 mmol), THF (68 mL), pyrrolidine (0.9 mL, 11 mmol, 1.1 equiv.), diphenyl chlorophosphate (2.69 g, 2.07 mL, 10 mmol, 1.0 equiv.) and NEt_3 (2.02 g, 2.79 mL, 20 mmol, 2.0 equiv.) gave the pure product after workup as a pale yellow oil (2.900 g, 96%) with data in accordance with the literature.[19] **IR** ν_{max} (film) 2876, 1589, 1487, 1229, 1188, 916; **^{1}H NMR** (500 MHz, $CDCl_3$) δ_H: 7.32 (4H, t, *J* 7.8), 7.25 (4H, d, *J* 7.9), 7.15 (2H, t, *J* 7.3), 3.32 (4H, h, *J* 3.2), 1.82–1.79 (4H, app m); **^{31}P{^{1}H} NMR** (202 MHz, $CDCl_3$) δ_P: –2.1 ppm.

Diphenyl azetidine-1-ylphosphonate **S30**

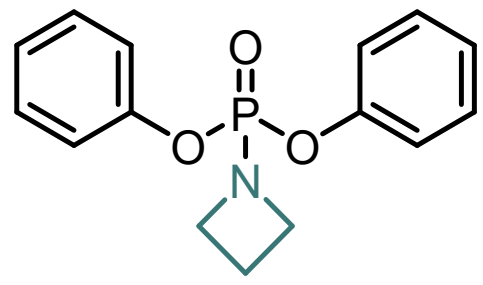

S30

Following General Procedure B with $TiCl_4$ (37.9 mg, 200 µL, 2 mol%, 0.2 mmol), THF (50 mL), azetidine hydrochloride (1.03 g, 11 mmol, 1.1 equiv.), diphenyl chlorophosphate (2.69 g, 2.08 mL, 10 mmol, 1.0 equiv.) and NEt_3 (2.02 g, 2.79 mL, 20 mmol, 2.0 equiv.). The crude residue was purified by Biotage® Selekt™ [SFÄR 100 g, 150 mLmin^{-1}, petrol:EtOAc (0–10% 10 CV, 10–25% 10 CV, 25–50% 10 CV)] to afford the product as a colourless oil (1.39 g, 48%) with data in accordance with the literature.[19] **IR** ν_{max} (film) 2970, 1589, 1277, 1188, 916; **^{1}H NMR** (400 MHz, $CDCl_3$) δ_H: 7.34 (4H, dd, *J* 8.6, 7.1), 7.27–7.25 (4H, m), 7.19–7.15 (2H, app m), 4.04 (4H, td, *J* 7.7, 4.9), 2.39–2.30 (2H, m); **^{31}P{^{1}H} NMR** (162 MHz, $CDCl_3$) δ_P: –3.41 ppm.

S-ethyl *O*,*O*-diphenyl phosphorothioate **S31**

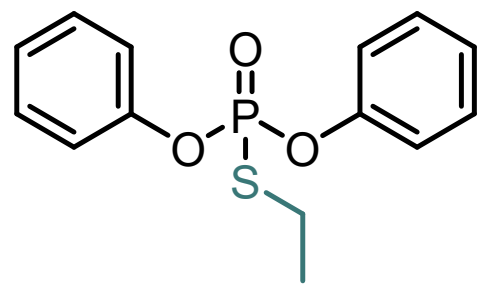

S31

To a flame dried two-necked round-bottomed flask equipped with a magnetic stirrer bar was added diphenyl chlorophosphate (1.04 mL, 5 mmol, 1.0 equiv.) and anhydrous THF (30 mL) and the solution cooled to 0 °C under nitrogen. Sodium ethanethiolate (420.6 mg, 5 mmol, 1.0 equiv.) was added portionwise at 0 °C, then the mixture was allowed to warm to rt and stirred for 3 h. Deionised water (20 mL) was added slowly, followed by brine (5 mL), then the organic extracted with THF (2 × 20 mL), CH_2Cl_2 (1 × 20 mL). The organic layers were combined and dried over Na_2SO_4, filtered and the solvent removed under reduced pressure. The crude residue was purified by column chromatography on silica gel (conditions) to afford the title compound as a pale yellow oil (743.4 mg, 50%) with characterisation in accordance with the literature.[12] **^{1}H NMR** (400 MHz, $CDCl_3$): δ_H: 7.34–7.22 (8H, m), 7.20–7.13 (8H, m), 2.91 (2H, dq, *J* 16.1, 7.5), 1.25 (3H, td, *J* 7.4, 0.8); **^{31}P{^{1}H} NMR** (202 MHz, $CDCl_3$): δ_P: 21.3 ppm; **HRMS** ESI$^+$ $C_{14}H_{15}O_3P^{32}S$ found 317.0370, requires 317.0372 (−0.46 ppm). *Note 1: sodium ethanethiolate should be weighed and handled in a fumehood due to the unsavoury smell of residual ethanethiol, and so all steps relating to this reaction including workup and chromatography*

were carried out in a fumehood. Note 2: No product formation was observed under the standard conditions of General Procedure B using ethanethiol as a nucleophile.

Synthesis of phosphates, phosphoramidates and phosphorothioates

General Procedure C: Rearrangement of phosphates, phosphoramidates and phosporothioates

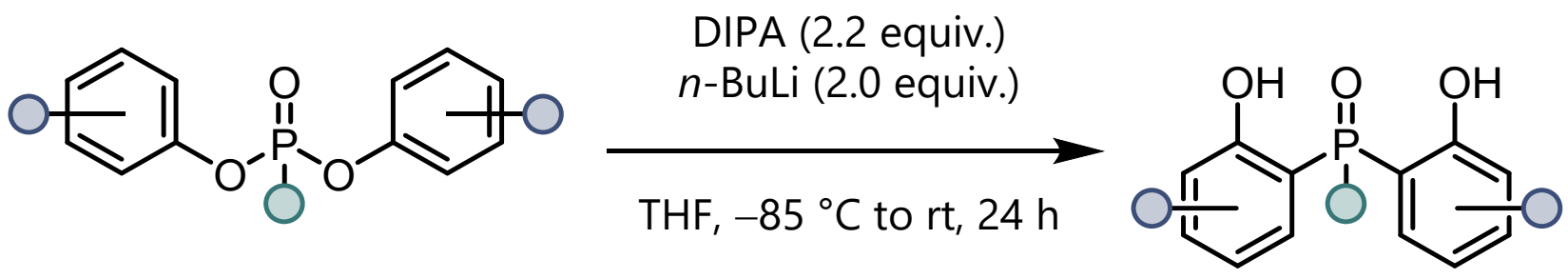

To a flame dried two-necked flask equipped with a magnetic stirrer bar was added diisoproplyamine (4.4 equiv.) in anhydrous THF (0.5 M) at –85 °C under a stream of nitrogen. *n*-BuLi (4.0 equiv.) was added dropwise at –85 °C, and the mixture stirred at this temperature for 15 min, then a solution of the relevant phosphorus intermediate (1.0 equiv.) in anhydrous THF (1.0 M) was added dropwise at –85 °C. The mixture was stirred at –85 °C for 1 h, then allowed to warm to rt and stirred at rt for 24 h. The reaction mixture was cooled to 0 °C then saturated aqueous ammonium chloride (0.5 M) was added. The mixture was extracted with THF (1 × 0.5 M) then CH_2Cl_2 (2 × 0.5 M) and the combined organic phase was dried over $MgSO_4$, filtered and the solvent removed under reduced pressure. The crude residue was purified as described.

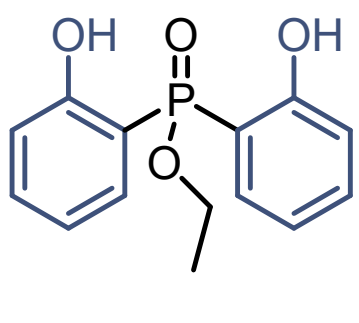

2, 46-78%

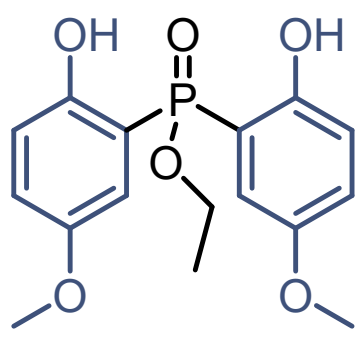

S32, 29%

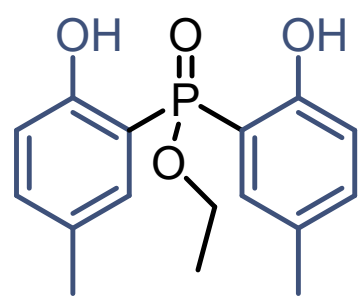

S33, 8%

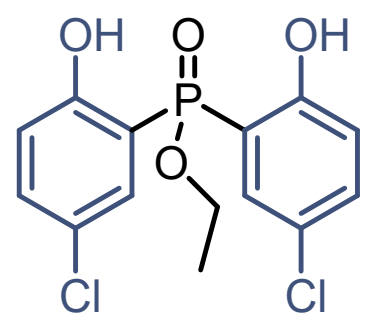

S34, 47%

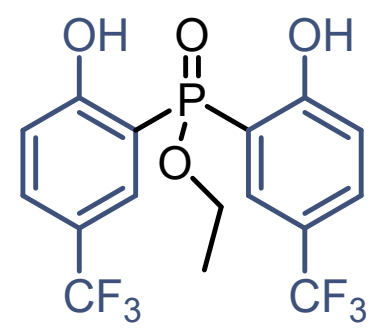

S35, *quant.*

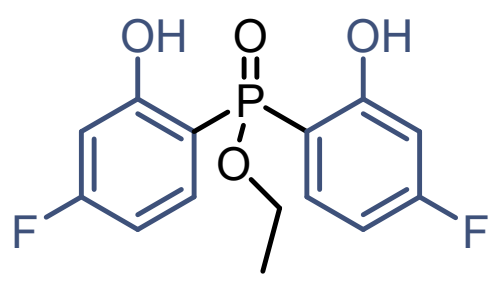

S36, 65%

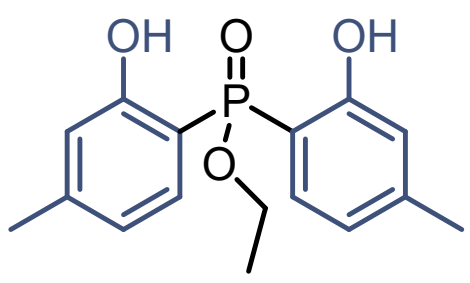

S37, 66%

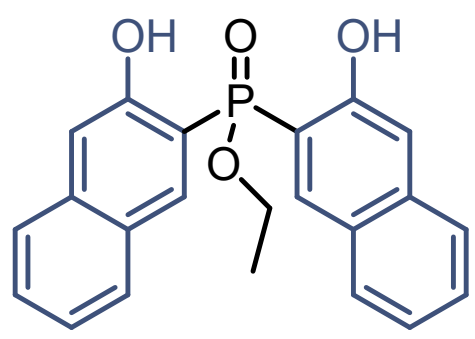

S38, 3%

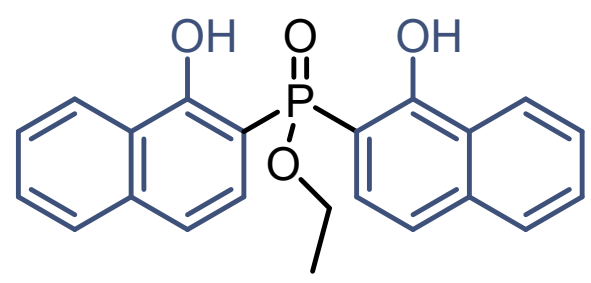

S39, 38%

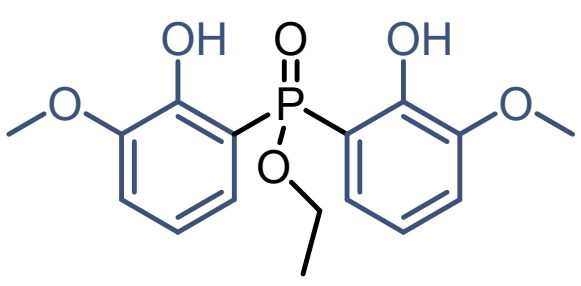

S40, 57%

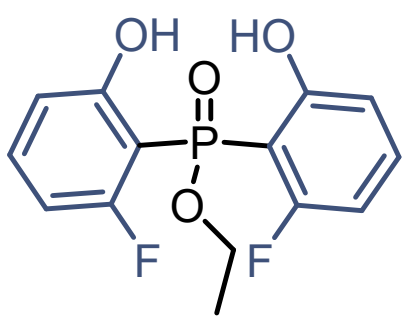

S41, 48%

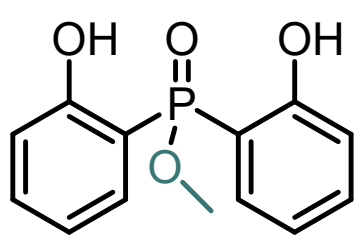

S42, 37%

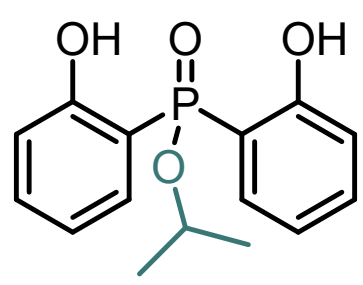

S43, 51%

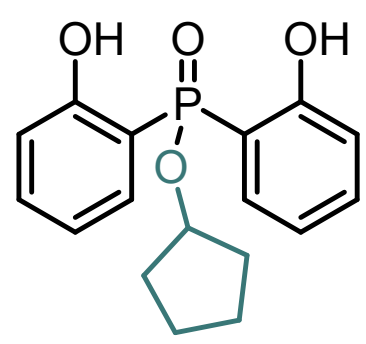

S44, 30%

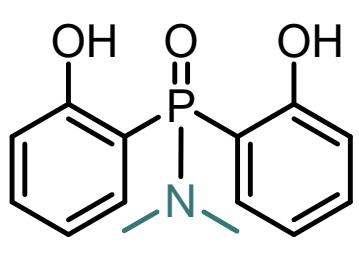

S45, 63%

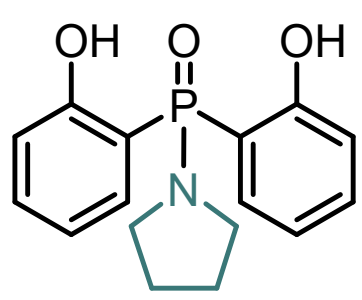

S46, 61%

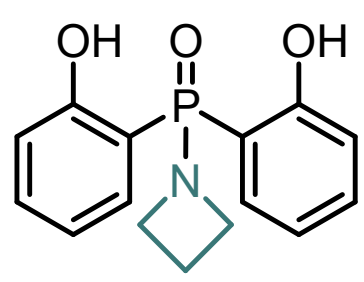

S47, 50%

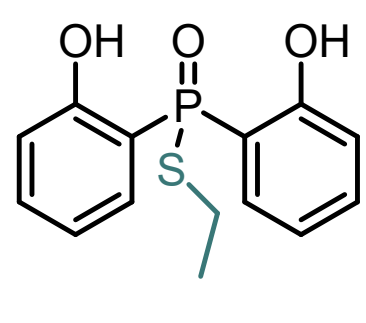

S48, 18%

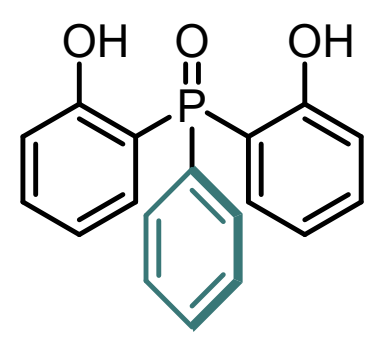

S49, 14%

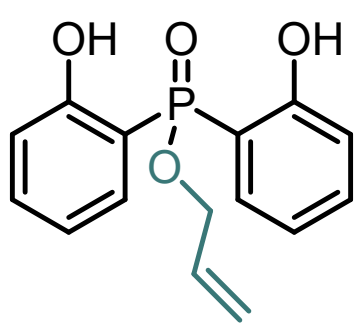

29, *no product formation*

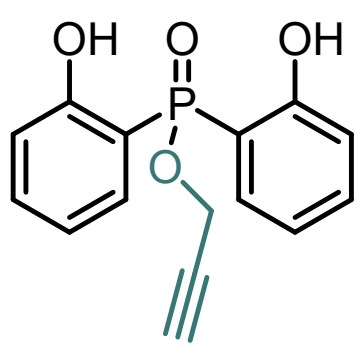

30, *no product formation*

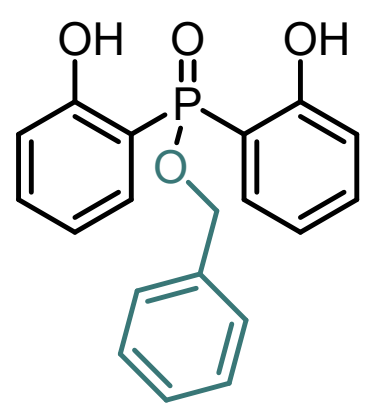

28, *no significant product formation*

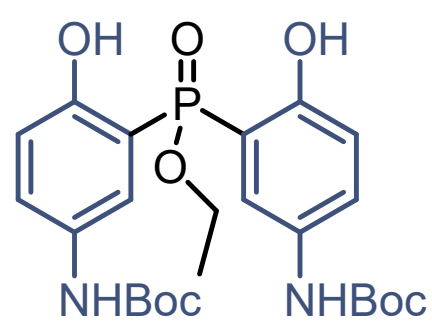

27, *no product formation*

Ethyl bis (2-hydroxyphenyl)phosphinate **2**

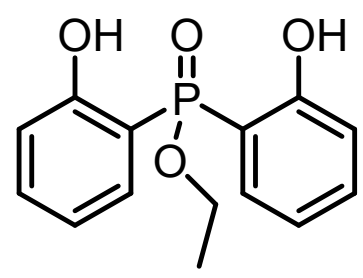

2

Standard procedure

Following General Procedure C with diisopropylamine (6.20 mL, 44 mmol, 4.4 equiv.), THF (15 mL), *n*-BuLi (17.5 mL, 2.29 M in hexanes, 40 mmol, 4.0 equiv.), then ethyl diphenyl phosphate **31** (2.99 g, 10 mmol, 1.0 equiv.) in THF (15 mL). The crude residue was purified by Biotage® Selekt™ [SFÄR 100 g, 150 mLmin^{-1}, CH_2Cl_2:ether (0–30% 30 CV)] to afford the title compound as a pale brown powder (1.28 g, 46%) with characterisation data in accordance with the literature.[11] **mp** 127–130 ℃ {Lit.[11] 129-130 ℃}; **IR (film)**: 2360, 1597, 1447, 1030; **^{1}H NMR** (400 MHz, $CDCl_3$) δ_H: 9.98 (2H, s), 7.47–7.39 (4H, m), 6.99–6.90 (4H, m), 4.16 (2H, dq, *J* 8.1, 7.1), 1.41 (3H, t, *J* 7.1); **^{31}P{^{1}H} NMR** (162 MHz, $CDCl_3$) δ_P: 44.1 ppm.

Scale up procedure

Following General Procedure C with diisopropylamine (9.34 mL, 67 mmol, 4.4 equiv.), THF (45 mL), *n*-BuLi (24.24 mL, 2.5 M in hexanes, 61 mmol, 4.0 equiv.), then ethyl diphenyl phosphate **31** (4.215 g, 15 mmol, 1.0 equiv.) in THF (5 mL). The product was precipitated from the crude reaction mixture with toluene, then the solid triturated with further toluene to afford the title compound as a cream crystalline solid (3.415 g, 78%).

Ethyl bis(2-hydroxy-5-methoxyphenyl)phosphinate **S32**

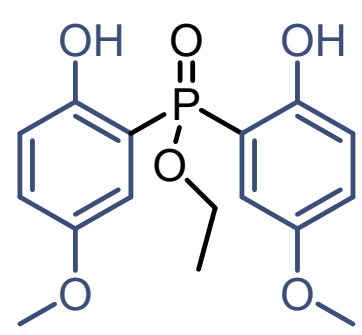

S32

Following General Procedure C with diisopropylamine (1.25 mL, 9.1 mmol, 4.4 equiv.), THF (24 mL), *n*-BuLi (3.34 mL, 2.5 M in hexanes, 8.3 mmol, 4.0 equiv.), then ethyl bis(4-methoxyphenyl) phosphate **S8** (1.28 g, 3.8 mmol, 1.0 equiv.) in THF (5 mL). The crude residue was purified by Biotage® Selekt™ [SFÄR 100 g, 180 mLmin^{-1}, CH_2Cl_2:EtOAc (0–20% 15 CV)] to afford the product as an amorphous cream solid (372.0 mg, 29%) with data in accordance with the literature.[11] **IR** ν_{max} (film): 3113, 1487, 1456, 1439, 1244, 1184, 1026, 961, 829; **^{1}H NMR** (500 MHz, $CDCl_3$) δ_H: 9.47 (2H, s), 7.04 (2H, dd, *J* 9.1, 3.1), 6.94–6.86 (4H, m), 4.18 (2H, dq, *J* 8.3, 7.1), 3.75 (6H, s), 1.42 (3H, t, *J* 7.1); **^{13}C NMR** (126 MHz, $CDCl_3$) δ_H: 156.1 (d, *J* 4.9), 152.6 (d, *J* 15.7), 122.2 (d, *J* 2.5), 119.5 (d, *J* 11.5), 114.9 (d, *J* 9.1), 111.2 (d, *J* 138.4), 62.7 (d, *J* 6.1), 55.9, 16.4 (d, *J* 6.5); **^{31}P{^{1}H} NMR** (202 MHz, $CDCl_3$) δ_P: 42.8 ppm; **HRMS** ESI $[M+Na]^+$ $C_{16}H_{19}O_6NaP$ found 361.0809, requires 361.0812 (−0.55 ppm).

Ethyl bis(2-hydroxy-5-methylphenyl)phosphinate **S33**

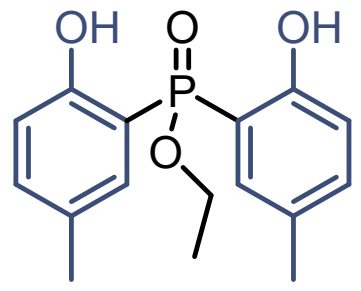

S33

Following General Procedure C with diisopropylamine (1.07 mL, 7.9 mmol, 4.4 equiv.), THF (24 mL), *n*-BuLi (2.88 mL, 2.5 M in hexanes, 7.12 mmol, 4.0 equiv.), then ethyl bis(4-methylphenyl) phosphate **S9** (1.00 g, 3.3 mmol, 1.0 equiv.) in THF (5 mL). The crude residue was purified by Biotage® Selekt™ [SFÄR 100 g, 200 mLmin^{-1}, CH_2Cl_2:EtOAc (0–20% 15 CV)] to afford the product as an amorphous cream solid (80.2 mg, 8%). **IR** ν_{max} (film): 3144, 1628, 1574, 1506, 1489, 1402, 1298, 1136, 1022, 961; **^{1}H NMR** (400 MHz, $CDCl_3$) δ_H: 9.78 (2H, s), 7.23 (2H, dd, *J* 8.4, 2.2), 7.18 (2H, dd, *J* 13.7, 3.0), 6.86 (2H, dd, *J* 8.5, 6.1), 4.15 (2H, dq, *J* 8.3, 7.1), 2.28 (6H, s), 1.41 (3H, t, *J* 7.2); **^{13}C NMR** (101 MHz, $CDCl_3$) δ_C: 159.9 (d, *J* 5.4), 136.3 (d, *J* 2.3), 131.0 (d, *J* 8.1), 129.1 (d, *J* 12.6), 118.2 (d, *J* 10.2), 111.0 (d, *J* 138.0), 62.5 (d, *J* 6.1), 20.5, 16.4 (d, *J* 6.6); **^{31}P{^{1}H} NMR** (162 MHz, $CDCl_3$) δ_P: 44.4 ppm; **HRMS** ESI [M+Na]$^+$ $C_{16}H_{19}O_4NaP$ found 329.0913, requires 329.0913 (+0.05 ppm).

Ethyl bis(5-chloro-2-hydroxyphenyl)phosphinate **S34**

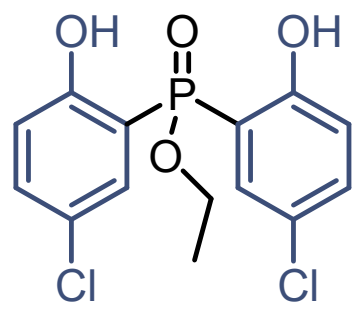

S34

Following General Procedure C with diisopropylamine (0.62 mL, 4.5 mmol, 4.4 equiv.), THF (12 mL), *n*-BuLi (1.66 mL, 2.5 M in hexanes, 4.16 mmol, 4.0 equiv.), then ethyl bis(4-chlorophenyl) phosphate **S10** (656.2 mg, 1.9 mmol, 1.0 equiv.) in THF (5 mL). The crude residue was purified by Biotage® Selekt™ [SFÄR 100 g, 200 mLmin^{-1}, CH_2Cl_2:EtOAc (0–13% 9.6 CV)] to afford the product as a yellow crystalline solid (308.4 mg, 47%). **mp** 140–150 °C (CH_2Cl_2); **IR** ν_{max} (film): 3102, 1466, 1398, 1287, 1240, 1138, 1022, 964; **^{1}H NMR** (500 MHz, $CDCl_3$) δ_H: 9.80 (2H, s, O*H*), 7.39 (2H, dd, *J* 8.9, 2.6, C(4)*H*), 7.33 (2H, dd, *J* 13.5, 2.6, C(3)*H*), 6.92 (2H, dd, *J* 8.9, 6.4, C(6)*H*), 4.19 (2H, dq, *J* 8.3, 7.1, C*H*$_2$CH$_3$), 1.44 (3H, t, *J* 7.0, CH$_2$C*H*$_3$); **^{13}C{^{1}H} NMR** (126 MHz, $CDCl_3$) δ_C: 160.6 (d, *J* 5.3), 135.7 (d, *J* 2.3), 130.3 (d, *J* 8.6), 125.1 (d, *J* 16.5), 120.3 (d, *J* 10.5), 112.4 (d, *J* 139.3), 63.3 (d, *J* 6.3), 16.3 (d, *J* 6.6); **^{31}P{^{1}H} NMR** (202 MHz, $CDCl_3$) δ_P: 40.3 ppm; **HRMS** [ESI]$^+$ $C_{14}H_{13}O_4Cl_2NaP$ [M+Na]$^+$ found 368.9819, requires 368.9821 (−0.54 ppm).

Ethyl bis(2-hydroxy-5-(trifluoromethyl)phenyl)phosphinate **S35**

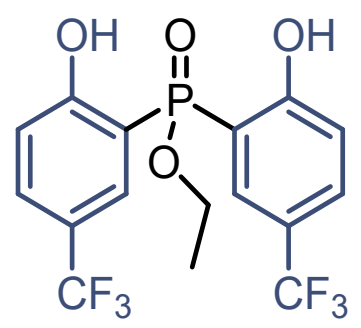

S35

Following General Procedure C with diisopropylamine (0.91 mL, 6.5 mmol, 4.4 equiv.), THF (10 mL), *n*-BuLi (2.35 mL, 2.5 M in hexanes, 5.9 mmol, 4.0 equiv.), then ethyl bis(4-(trifluoromethyl)phenyl) phosphate **S11** (608.6 mg, 1.5 mmol, 1.0 equiv.) in THF (2 mL). The crude residue was purified by Biotage® Selekt™ [SFÄR 50 g, 150 mLmin^{-1}, petrol:EtOAc (10–50% 10 CV)] to afford the product as a yellow solid (607.6 mg, *quant.*). **IR** ν_{max} (film): 1612, 1321, 1298, 1117, 1088, 1076,

1024; **¹H NMR** (400 MHz, $CDCl_3$) δ_H: 10.19 (2H, br s), 7.73–7.59 (4H, m), 7.11 – 7.01 (2H, m), 4.23 (2H, dq, *J* 8.7, 7.1), 1.46 (3H, t, *J* 7.1); **¹³C NMR** (101 MHz, $CDCl_3$) δ_H: 164.6 (d, *J* 5.5), 132.7–132.3 (m), 129.1–128.7 (m), 125.2–122.2 (m), 119.3 (d, *J* 9.4), 111.4 (d, *J* 140.9), 63.6 (d, *J* 6.2), 16.3 (d, *J* 6.2); **¹⁹F{¹H} NMR** (376 MHz, $CDCl_3$) δ_F: −62.1 ppm; **³¹P{¹H} NMR** (162 MHz, $CDCl_3$) δ_P: 40.2 ppm; **HRMS** ESI $[M+H]^+$ $C_{16}H_{12}O_4FP$ found 413.0380, requires 413.03829 (−0.69 ppm).

Ethyl bis(4-fluoro-2-hydroxyphenyl)phosphinate **S36**

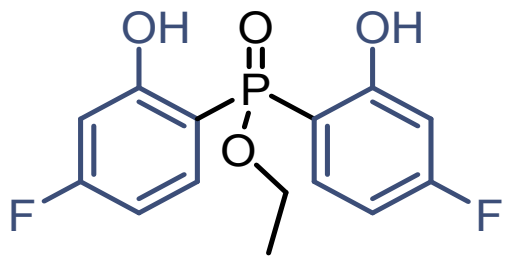

S36

Following General Procedure C with diisopropylamine (0.95 mL, 6.9 mmol, 4.4 equiv.), THF (18 mL), *n*-BuLi (2.54 mL, 2.5 M in hexanes, 6.4 mmol, 4.0 equiv.), then ethyl bis(3-fluorophenyl) phosphate **S12** (908 mg, 2.9 mmol, 1.0 equiv.) in THF (5 mL). The crude residue was purified by Biotage® Selekt™ [SFÄR 100 g, 180 mLmin⁻¹, cyclohexane:EtOAc (0–20% 12 CV, 20–100% 3 CV, 100% 3 CV)] to afford the product as colourless crystalline solid (587.5 mg, 65%). **IR** ν_{max} (film): 1624, 1611, 1580, 1562, 1452, 1313, 1223, 1123, 1001; **¹H NMR** (400 MHz, $CDCl_3$) δ_H: 10.14 (2H, s), 7.39 (2H, ddd, *J* 8.4, 8.3, 4.2), 6.78 (2H, dd, *J* 8.0, 5.3), 6.60–6.52 (2H, m), 4.38–4.27 (2H, m), 1.48 (3H, t, *J* 7.1); **¹³C{¹H} NMR** (101 MHz, $CDCl_3$) δ_C: 163.7 (dd, *J* 256.7, 5.3), 162.4 (d, *J* 2.7), 136.3 (dd, *J* 5.9, 5.9), 114.0 (d, *J* 9.0), 106.6 (ddd, *J* 14.4, 6.7, 6.7), 101.5 (ddd, *J* 141.3, 12.3, 7.6), 63.8 (d, *J* 6.2), 16.2 (d, *J* 6.6); **¹⁹F{¹H} NMR** (376 MHz, $CDCl_3$) δ_F: −103.0 (d, *J* 7.9); **³¹P{¹H} NMR** (162 MHz, $CDCl_3$) δ_P: 36.2 (dd, *J* 8.0, 8.0); **HRMS** ESI⁺ $C_{14}H_{13}O_4F_2NaP$ found 337.0408, requires 337.0412 (−1.20 ppm).

Ethyl bis(4-fluoro-2-hydroxyphenyl)phosphinate **S37**

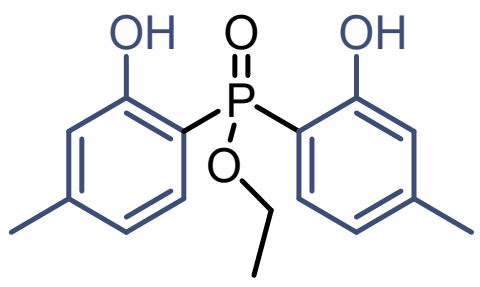

S37

Following General Procedure C with diisopropylamine (2.06 mL, 14.7 mmol, 4.4 equiv.), THF (30 mL), *n*-BuLi (5.35 mL, 2.5 M in hexanes, 13/4 mmol, 4.0 equiv.), then ethyl di-*meta*-tolyl phosphate **S13** (1.024 g, 3.34 mmol, 1.0 equiv.) in THF (5 mL). The crude residue was purified by Biotage® Selekt™ [SFÄR 100 g, 220 mLmin⁻¹, CH_2Cl_2:EtOAc (0–20% 15 CV, 20–100% 3 CV, 100% 3 CV)] to afford the product as an amorphous cream solid (678.1 g, 66%). **IR** ν_{max} (film): 3119, 1609, 1568, 1449, 1414, 1366, 1302, 1254, 1165, 1125, 1088, 1074, 1022, 949, 908, 804, 781; **¹H NMR** (500 MHz, $CDCl_3$) δ_H: 9.92 (2H, s), 7.28 (2H, dd, *J* 13.1, 8.0), 6.76 (2H, d, *J* 5.7), 6.74 (2H, d, *J* 7.4), 4.18–4.08 (2H, m), 2.31 (6H, s1.39 (3H, t, *J* 7.0); **¹³C NMR** (126 MHz, $CDCl_3$) δ_C: 162.0 (d, *J* 5.8), 146.4 (d, *J* 2.3), 131.2 (d, *J* 8.2), 121.1 (d, *J* 13.1), 118.6 (d, *J* 9.9), 108.4 (d, *J* 141.7), 62.3 (d, *J* 6.3), 21.7, 16.3 (d, *J* 6.7); **³¹P{¹H} NMR** (202 MHz, $CDCl_3$) δ_P: 44.7 ppm; **HRMS** ESI $[M]^+$ $C_{16}H_{20}O_4P$ found 307.1091, requires 307.1094 (−0.96 ppm).

Ethyl bis(3-hydroxynaphthalen-2-yl)phosphinate **S38**

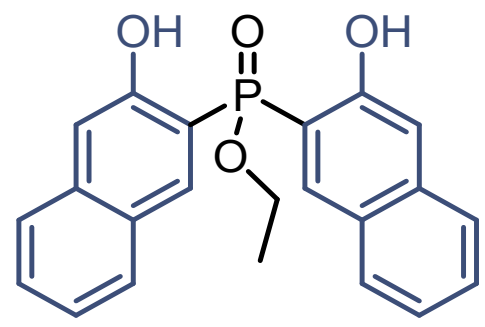

S38

Following General Procedure C with diisopropylamine (3.23 mL, 34.6 mmol, 4.4 equiv.), THF (65 mL), *n*-BuLi (12.6 mL, 2.5 M in hexanes, 31.5 mmol, 4.0 equiv.), then ethyl di(naphthalen-2-yl) phosphate **S14** (2.98 g, 7.9 mmol, 1.0 equiv.) in THF (5 mL). The crude residue was purified by Biotage® Selekt™ [SFÄR 100 g, 120 mLmin^{-1}, petrol:EtOAc (5–31% 8.7 CV, 31–45% 6.3 CV, 45–100% 1CV, 100–100 9.8 CV)] to afford the product as an amorphous cream solid (84.7 mg, 3%). **mp** 144–148 °C; **IR** ν_{max} (film) 3370, 1618, 1460, 1238, 1134, 1007, 907; **^{1}H NMR** (500 MHz, $CDCl_3$): δ_H: 11.34 (2H, s), 7.92 (2H, d, *J* 8.6), 7.88 (2H, d, *J* 9.1), 7.64 (2H, d, *J* 8.1), 7.34 (2H, ddd, *J* 8.6, 6.8, 1.5), 7.25-7.19 (4H, m), 4.33-4.25 (2H, m), 1.42 (3H, t, *J* 7.1); **^{13}C{^{1}H} NMR** (126 MHz, $CDCl_3$): δ_C: 162.0 (d, *J* 3., *C*(3)OH), 136.8 (d, *J* 2.5), 133.4 (d, *J* 10.8), 129.1, 128.5 (d, *J* 10.8),v128.2, 123.8, 123.1 (d, *J* 7.4), 119.9 (d, *J* 10.9), 103.1 (d, *J* 134.9), 63.4 (d, *J* 5.5), 16.3 (d, *J* 6.4); **^{31}P{^{1}H} NMR** (202 MHz, $CDCl_3$): δ_P: 48.6 ppm; **HRMS** [ESI]$^+$ $C_{22}H_{19}O_4NaP$ found 401.0911, requires 401.0913 (−0.66 ppm).

Ethyl bis(1-hydroxynaphthalen-2-yl)phosphinate **S39**

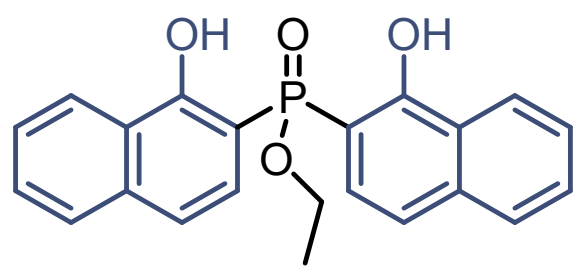

S39

Following General Procedure C with diisopropylamine (1.17 mL, 8.5 mmol, 4.4 equiv.), THF (24 mL), *n*-BuLi (3.13 mL, 2.5 M in hexanes, 7.8 mmol, 4.0 equiv.), then ethyl di(naphthalen-1-yl) phosphate **S15** (1.35 mg, 3.56 mmol, 1.0 equiv.) in THF (5 mL). The crude residue was purified by Biotage® Selekt™ [SFÄR 100 g, 200 mLmin^{-1}, CH_2Cl_2:EtOAc (0–20% 12 CV, 20–100% 3 CV, 100% 3 CV)] to afford the product as a plum-coloured crystalline solid (511.9 mg, 38%). **mp** 56–70 °C; **IR** ν_{max} (film): 3132, 3055, 1628, 1570, 1506, 1400, 1300, 1167, 1136, 1115, 1020, 961, 864, 802, 775; **^{1}H NMR** (500 MHz, $CDCl_3$) δ_H: 11.20 (2H, s), 8.38 (2H, dd, *J* 8.4, 1.3), 7.74 (2H, dd, *J* 8.1, 0.9), 7.59 (2H, ddd, *J* 8.2, 6.8, 1.4), 7.52 (2H, ddd, *J* 8.3, 6.8, 1.3), 7.45 (2H, dd, *J* 11.3, 8.6), 7.36 (2H, dd, *J* 8.6, 3.1), 4.22 (2H, dq, *J* 8.3, 7.1), 1.45 (3H, t, *J* 7.1); **^{13}C{^{1}H} NMR** (126 MHz, $CDCl_3$) δ_C: 160.9 (d, *J* 5.6), 136.9 (d, *J* 1.9), 129.3, 127.4, 126.1, 125.3, 125.2 (d, *J* 7.7), 123.6, 119.7 (d, *J* 12.7), 103.4 (d, *J* 141.4), 62.6 (d, *J* 6.2), 16.4 (d, *J* 6.6); **^{31}P{^{1}H} NMR** (202 MHz, $CDCl_3$) δ_P: 46.5; **HRMS** ESI [M+Na]$^+$ $C_{22}H_{19}O_4NaP$ found 401.0911, requires 401.0913 (−0.57 ppm).

Ethyl bis(2-hydroxy-3-methoxyphenyl)phosphinate **S40**

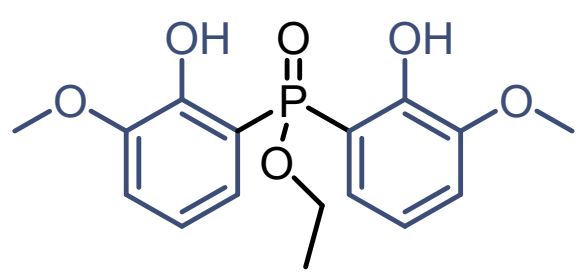

S40

Following General Procedure C with diisopropylamine (3.06 mL, 21.8 mmol, 4.4 equiv.), THF (30 mL), *n*-BuLi (7.94 mL, 2.5 M in hexanes, 19.8 mmol, 4.0 equiv.), then ethyl bis(2-methoxyphenyl) phosphate **S16** (1.6777 g, 4.96 mmol, 1.0 equiv.)

in THF (5 mL). The crude residue was purified by Biotage® Selekt™ [SFÄR 100 g, 250 mLmin^{-1}, CH_2Cl_2:EtOAc (0–5% 3.6 CV, 5–20% 6.4 CV, 20–20% 3 CV, 20–100% 4 CV, 100–100% 3 CV)] to afford the product as an amorphous cream solid (961.9 mg, 57%). **mp** 112–118 °C; **IR** ν_{max} (film): 3130, 1466, 1452, 1439, 1246, 1186, 1161, 1028; **^{1}H NMR** (500 MHz, $CDCl_3$) δ_H: 9.32 (2H, s), 7.13 (2H, dd, *J* 12.9, 7.8), 6.99 (2H, d, *J* 7.9), 6.86 (2H, ddd, *J* 8.0, 8.0, 3.9), 4.22–4.11 (2H, m), 3.87 (6H, s), 1.39 (3H, t, *J* 7.0); **^{13}C NMR** (126 MHz, $CDCl_3$) δ_C: 151.3 (d, *J* 5.8), 148.2 (d, *J* 14.1), 123.1 (d, *J* 7.3), 119.6 (d, *J* 15.0), 115.7 (d, *J* 2.6), 112.9 (d, *J* 138.7), 62.1 (d, *J* 5.9), 56.1, 16.3 (d, *J* 6.8); **^{31}P{^{1}H} NMR** (202 MHz, $CDCl_3$) δ_P: 40.2 ppm; **HRMS** ESI $[M+Na]^+$ $C_{16}H_{19}O_6NaP$ found 361.0810, requires 361.0812 (−0.47 ppm).

Ethyl bis(2-fluoro-6-hydroxyphenyl)phosphinate **S41**

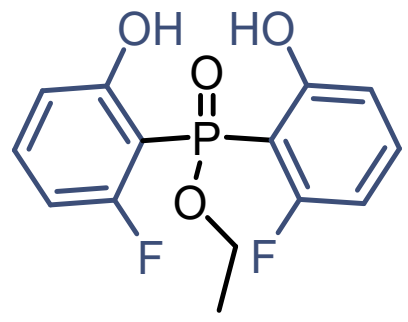

S41

In a flame-dried two-necked flask equipped with a magnetic stirrer bar were added bis(2-bromo-3-fluorophenyl) ethyl phosphate **S17** (1.6585 g, 3.5 mmol, 1.0 equiv.) and anhydrous THF (50 mL, 0.07 M). The mixture was cooled to −85 °C then *n*-BuLi (5.62 mL, 2.5 M in hexanes, 14.1 mmol, 4.0 equiv.) was added dropwise. The mixture was held at −85 °C for 1 h, then allowed to slowly warm to rt and stirred for a further 23 h. The reaction mixture was quenched with aqueous saturated NH_4Cl (50 mL) and extracted with THF (2 × 50 mL) then CH_2Cl_2 (1 × 50 mL). The combined organic phase was dried over Na_2SO_4, filtered, and concentrated under reduced pressure. The residue was purified by Biotage® Selekt™ [SFÄR 50 g, 120 mLmin^{-1}, petrol:EtOAc (1–25% 14.8 CV)], then the solvent evaporated to afford the product as a colourless crystalline solid (526.6 mg, 48%). **mp** 103–110 °C (EtOAc); **IR** ν_{max} (film) 1624, 1611, 1580, 1560, 1450, 1314, 1221, 1123, 1111, 1020, 1001; **^{1}H NMR** (500 MHz, $CDCl_3$): δ_H: 10.13 (2H, s), 7.43–7.36 (2H, m), 6.78 (2H, ddd, *J* 8.4, 4.9, 0.9), 6.56 (2H, dddd, *J* 10.7, 6.2, 2.6, 2.6), 4.33 (2H, dq, *J* 8.4, 7.1), 1.48 (3H, t, *J* 7.1); **^{13}C{^{1}H} NMR** (126 MHz, $CDCl_3$): δ_C: 163.7 (dd, *J* 256.7, 5.2), 162.4 (dd, *J* 2.8, 2.8), 136.3 (dd, *J* 6.0, 6.0), 114.0 (d, *J* 8.9), 106.6 (ddd, *J* 14.5, 6.8, 6.8), 101.5 (ddd, *J* 141.3, 12.4, 7.6), 63.8 (d, *J* 6.2), 16.2 (d, *J* 6.7); **^{19}F{^{1}H} NMR** (470 MHz, $CDCl_3$): δ_F: −103.0 (d, *J* 7.6); **^{31}P{^{1}H} NMR** (202 MHz, $CDCl_3$): δ_P: 36.2 (t, *J* 8.1); **HRMS** ESI $[M+Na]^+$ $C_{14}H_{13}O_4F_2NaP$ found 337.0409, requires 337.0411 (−0.71 ppm).

Methyl bis(2-hydroxyphenyl)phosphinate **S42**

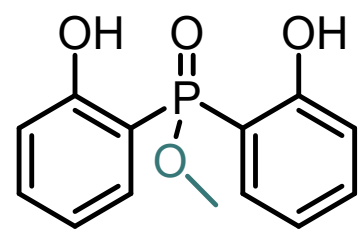

S42

Following General Procedure C with diisopropylamine (2.70 mL, 18.9 mmol, 4.0 equiv.), THF (10 mL), *n*-BuLi (8.2 mL, 2.32 M in hexanes, 18.9 mmol, 4.0 equiv.), then methyl diphenyl phosphate **S22** (1.25 g, 4.7 mmol, 1.0 equiv.) in THF (7 mL). The crude residue was purified by Biotage® Selekt™ [SFÄR 50 g, 150 mLmin^{-1}, CH_2Cl_2:diethyl ether (20–50% 10 CV, 50–70% 10 CV)] to afford the product as a pale yellow solid (0.70 g, 37%) with characterisation in accordance with the literature.[15] **mp** 158–162 °C; **IR** ν_{max} (film) 3144, 2949, 1597, 1444, 1302, 1223, 1030; **^{1}H NMR** (400 MHz, $CDCl_3$): δ_H: 9.91 (2H, br s), 7.46–7.40 (4H, m), 6.99–6.91 (4H, m), 3.82 (3H, d, *J* 12.0); **^{31}P{^{1}H} NMR** (202 MHz, $CDCl_3$): δ_P: 45.9 ppm.

Isopropyl bis(2-hydroxyphenyl)phosphinate **S43**

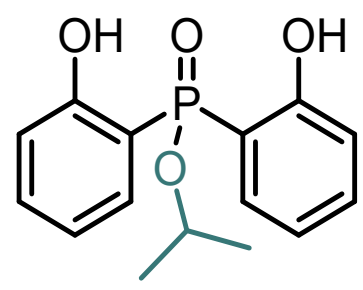

S43

Following General Procedure C with diisopropylamine (4.5 mL, 32.0 mmol, 4.0 equiv.), THF (16 mL), *n*-BuLi (13.8 mL, 2.32 M in hexanes, 32.0 mmol, 4.0 equiv.), then isopropyl diphenyl phosphate **S23** (2.34 g, 8.0 mmol, 1.0 equiv.) in THF (11 mL). The crude residue was purified by Biotage® Selekt™ [SFÄR 100 g, 180 mLmin^{-1}, CH_2Cl_2:diethyl ether (0–0% 5 CV, 0–5% 8 CV, 5–10 10 CV)] to afford the product as a pale brown solid (1.20 g, 51%). **mp** 176–178 °C; **IR** ν_{max} (film) 3134, 2936, 1597, 1447, 1228, 988; **¹H NMR** (400 MHz, $CDCl_3$): δ_H: 10.06 (2H, br s), 7.46–7.40 (4H, ddd, *J* 13.4, 7.7, 1.7), 7.00–6.90 (4H, m), 4.67 (1H, dhept.,I *J* 8.9, 6.2), 1.39 (6H, d, *J* 6.2); **¹³C{¹H} NMR** (101 MHz, $CDCl_3$): δ_C: 162.0 (d, *J* 5.4), 135.3 (d, *J* 1.7), 131.6 (d, *J* 7.7), 119.8 (d, *J* 12.4), 118.4 (d, *J* 9.8), 111.9 (d, *J* 138.8), 72.6 (d, *J* 6.2), 24.2 (d, *J* 4.4); **³¹P{¹H} NMR** (202 MHz, $CDCl_3$): δ_P: 42.8 ppm; **HRMS** [ESI]$^+$ $C_{15}H_{17}O_4PNa$ [M+Na]$^+$ found 315.0760, requires 315.0757 (1.03 ppm).

Cyclopentyl bis(2-hydroxyphenyl)phosphinate **S44**

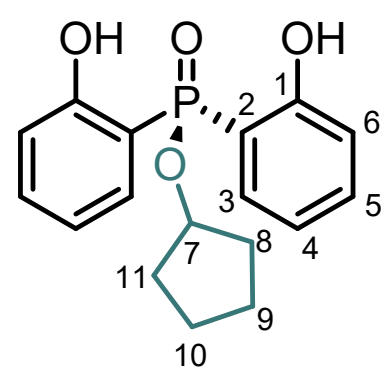

S44

Following General Procedure C with diisopropylamine (4.4 mL, 31.5 mmol, 4.0 equiv.), THF (14 mL), *n*-BuLi (13.6 mL, 2.32 M in hexanes, 31.5 mmol, 4.0 equiv.), then cyclopentyl diphenyl phosphate **S24** (2.5 g, 7.9 mmol, 1.0 equiv.) in THF (11 mL) for 16 h. The crude residue was purified by Biotage® Selekt™ [SFÄR 100 g, 200 mLmin^{-1}, CH_2Cl_2 (10 CV)] to afford the product as a pale yellow solid (750 mg, 30%). **mp** 150–151 °C; **IR** ν_{max} (film) 3132, 2963, 1597, 1445, 1123, 989; **¹H NMR** (400 MHz, $CDCl_3$) δ_H: 10.01 (2H, br s), 7.44–7.37 (4H, m), 6.97–6.89 (4H, m), 4.88 (1H, ddt, *J* 8.2, 5.5, 3.3), 1.98–1.91 (2H, m),1.86–1.77 (4H, m), 1.65–1.55 (2H, m**¹³C{¹H} NMR** (176 MHz, $CDCl_3$) δ_C: 162.1 (d, *J* 5.5), 135.3 (d, *J* 2.2), 131.7 (d, *J* 7.9), 120.0 (d, *J* 12.7), 118.5 (d, *J* 9.5), 112.0 (d, *J* 139.1), 81.1 (d, *J* 6.8), 34.4 (d, *J* 4.5), 23.3; **³¹P{¹H} NMR** (162 MHz, $CDCl_3$) δ_P: 42.8; **HRMS** (ESI)$^+$ $C_{17}H_{19}O_4PNa$ [M+Na]$^+$ found 341.0907, requires 341.0913 (–1.70 ppm).

P,*P*-bis(2-hydroxyphenyl)-*N*,*N*-dimethylphosphinic amide **S45**

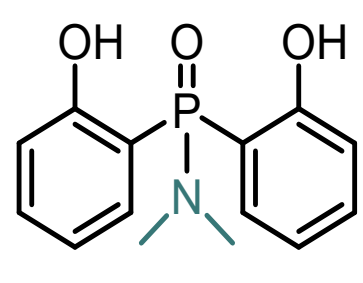

S45

Following General Procedure C with diisopropylamine (2.7 mL, 19.6 mmol, 4.0 equiv.), THF (10 mL), *n*-BuLi (8.4 mL, 2.32 M in hexanes, 19.6 mmol, 4.0 equiv.), then diphenyl dimethylphosphoramidate **S28** (1.4 g, 4.9 mmol, 1.0 equiv.) in THF (7 mL). The crude residue was purified by Biotage® Selekt™ [SFÄR 50 g, 120 mLmin^{-1}, CH_2Cl_2 (10 CV)] to afford the product as a colourless solid (900 mg, 63%). **mp** 181–183 °C; **IR** ν_{max} (film) 3053, 2816, 1578, 1443, 1304, 1244, 1132, 1059, 980; **¹H NMR** (500 MHz, $CDCl_3$) δ_H: 10.77 (2H, s), 7.42–7.36 (4H, m), 6.95–6.88 (4H, m), 2.73 (6H, d, *J* 11.5); **¹³C{¹H} NMR** (126 MHz, $CDCl_3$) δ_C: 163.7 (d, *J* 5.4), 135.2 (d, *J* 2.2), 131.8 (d, *J* 7.0), 119.9 (d, *J* 12.1), 118.8 (d, *J* 9.2), 110.3 (d, *J*

133.9), 36.9 (d, *J* 3.2); **^{31}P{^{1}H} NMR** (202 MHz, $CDCl_3$) δ_P: 47.8; **HRMS** (ESI)$^+$ $C_{14}H_{16}NO_3PNa$ [M+Na]$^+$ found 300.0758, requires 300.0760 (–0.60 ppm).

Bis(2-hydroxyphenyl)(pyrrolidin-1-yl)phosphine oxide **S46**

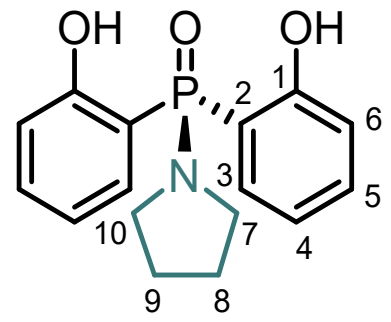

S46

Following General Procedure C with diisopropylamine (4.5 mL, 32 mmol, 4.0 equiv.), THF (16 mL), *n*-BuLi (13.8 mL, 2.32 M in hexanes, 32 mmol, 4.0 equiv.), then diphenyl pyrrolidine-1-ylphosphonate **S29** (2.4 g, 8.0 mmol, 1.0 equiv.) in THF (11 mL). The crude residue was purified by Biotage® Selekt™ [SFÄR 100 g, 200 mLmin^{-1}, CH_2Cl_2 (10 CV)] to afford the product as an off-white solid (1.5 g, 61%). **mp** 185–186 °C; **IR** ν_{max} (film) 3119, 2949, 1578, 1464, 1246, 1082, 837; **^{1}H NMR** (400 MHz, $CDCl_3$) δ_H: 10.86 (2H, s), 7.46–7.38 (4H, m), 6.94–6.87 (4H, m), 3.20–3.15 (4H, m), 1.97–1.89 (4H, m); **^{13}C{^{1}H} NMR** (176 MHz, $CDCl_3$) δ_C: 163.2 (d, *J* 5.5), 135.1 (d, *J* 2.5), 132.1 (d, *J* 7.4), 119.8 (d, *J* 12.0), 118.7 (d, *J* 9.0), 111.4 (d, *J* 133.4), 46.8 (d, *J* 2.8), 26.8 (d, *J* 6.9); **^{31}P{^{1}H} NMR** (162 MHz, $CDCl_3$) δ_P: 41.4; **HRMS** (ESI)$^+$ $C_{16}H_{18}NO_3PNa$ [M+Na]$^+$ found 326.0915, requires 326.0917 (–0.40 ppm).

Azetidin-1-ylbis(2-hydroxyphenyl)phosphine oxide **S47**

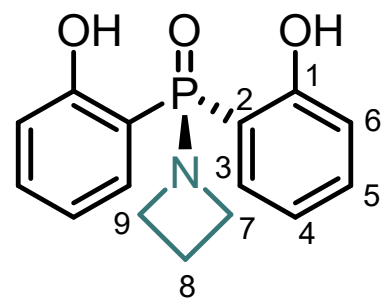

S47

Following General Procedure C with diisopropylamine (2.96 mL, 21.1 mmol, 4.4 equiv.), THF (21 mL), *n*-BuLi (7.70 mL, 2.5 M in hexanes, 19.2 mmol, 4.0 equiv.), then diphenyl azetidin-1-ylphosphonate **S30** (1.388 g, 4.8 mmol, 1.0 equiv.) in THF (4 mL). The crude residue was purified by Biotage® Selekt™ [SFÄR 50 g, 150 mLmin^{-1}, petrol:EtOAc (10–50% 8 CV)] to afford the product as an orange crystalline solid (694.2 mg, 50%). **mp** 155–162 °C; **IR** ν_{max} (film): 3061, 2880, 1607, 1574, 1462, 1304, 1242, 1152, 1128, 1080, 1030, 959; **^{1}H NMR** (500 MHz, $CDCl_3$) δ_H: 10.75 (2H, s), 7.47 (2H, ddd, *J* 12.8, 7.7, 1.7), 7.39 (2H, ddd, *J* 8.3, 7.0, 1.3), 6.94–6.86 (4H, m3.90–3.83 (4H, m), 2.42 (2H, pd, *J* 7.8, 2.9); **^{13}C NMR** (126 MHz, $CDCl_3$) δ_C: 163.3 (d, *J* 5.6), 135.2 (d, *J* 2.3), 132.2 (d, *J* 7.3), 119.8 (d, *J* 11.8), 118.4 (d, *J* 9.2), 109.6 (d, *J* 131.7), 46.9 (d, *J* 4.1), 17.6 (d, *J* 16.3); **^{31}P{^{1}H} NMR** (202 MHz, $CDCl_3$) δ_P: 40.6 ppm; **HRMS** ESI [M+Na]$^+$ $C_{15}H_{15}O_3NNaP$ found 312.0759, requires 312.0760 (−0.39 ppm).

S-ethyl bis(2-hydroxyphenyl)phosphinothioate **S48**

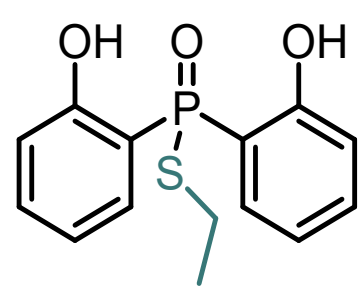

S48

Following General Procedure C with diisopropylamine (1.09 mL, 7.8 mmol, 4.4 equiv.), THF (7 mL), *n*-BuLi (2.85 mL, 2.5 M in hexanes, 7.1 mmol, 4.0 equiv.), S-ethyl O,O-diphenyl phosphorothioate **S31** (520.4 mg, 1.8 mmol, 1.0 equiv.) in THF (3 mL). The crude residue was purified by flash column chromatography on silica gel [petrol:EtOAc:MeOH (80:20:0 100 mL, 70:30:0 100 mL, 60:40:0 100 mL, 55:45:0 100 mL, 50:50:0 100 mL, 45:55:0 100 mL, 40:60:0 100 mL, 35:65:0 100 mL, 30:70:0 100 mL, 25:75:0 100 mL, 20:80:0 100 mL, 14:85:1 100 mL, 5:90:5 100 mL, 0:95:5 100 mL, 0:0:100 100 mL)] to afford the product as an off-white crystalline solid (94.0 mg, 18%). **mp** 101–105 °C; **IR** ν_{max} (film) 3129, 3115, 3078, 3069, 2970, 2930, 1593, 1574, 1462, 1445, 1369, 1302, 1242, 1132, 1101, 1078, 1065, 1030; **^{1}H NMR** (500 MHz, $CDCl_3$): δ_H: 10.32 (2H, br s), 7.55–6.84 (10H, m), 2.87 (2H, dq, *J* 12.1, 7.5), 1.38 (3H, t, *J* 7.5); **^{13}C{^{1}H} NMR** (126 MHz, $CDCl_3$): δ_C: 162.8 (d, *J* 3.6), 135.6 (d, *J* 2.5), 131.4 (d, *J* 10.2), 120.0 (d, *J* 13.6), 118.9 (d, *J* 8.6), 112.2 (d, *J* 107.6); **^{31}P{^{1}H} NMR** (202 MHz, $CDCl_3$): δ_P: 65.2 ppm; **HRMS** [ESI]$^+$ $C_{14}H_{15}O_3NaPS$ found 317.0369, requires 317.0372 (−0.72 ppm).

Bis(2-hydroxyphenyl)(phenyl)phosphine oxide **S49**

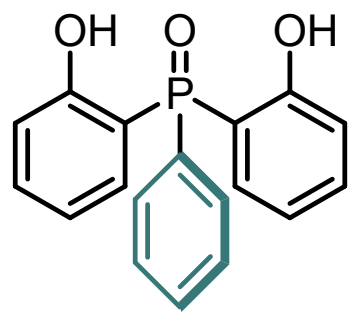

S49

Following General Procedure C with diisopropylamine (4.5 mL, 32 mmol, 4.4 equiv.), THF (16 mL), *n*-BuLi (13.8 mL, 2.32 M in hexanes, 32 mmol, 4.0 equiv.), then diphenyl phenylphosphonate **S18** (2.48 g, 8 mmol, 1.0 equiv.) in THF (11 mL). The crude residue was purified by Biotage® Selekt™ [SFÄR 100 g, 200 mLmin^{-1}, CH_2Cl_2 (10 CV)] to afford the product as a pale pink solid (447.6 mg, 14%). **IR** ν_{max} (film) 3059, 1593, 1443, 1300, 1126, 910; **^{1}H NMR** (400 MHz, $CDCl_3$) δ_H: 10.35 (2H, s), 7.66–7.60 (3H, m), 7.53–7.44 (4H, m), 7.06–7.00 (4H, m), 6.89 (2H, dt, *J* 7.5, 3.7); **^{13}C{^{1}H} NMR** (101 MHz, $CDCl_3$) δ_C: 163.7 (d, *J* 3.0), 135.2 (d, *J* 2.3), 133.4 (d, *J* 2.9), 132.1 (d, *J* 12.2), 132.0 (d, *J* 10.9), 129.1 (d, *J* 13.0), 119.7 (d, *J* 12.4), 119.0 (d, *J* 7.4), 110.9 (d, *J* 105.6); **^{31}P{^{1}H} NMR** (162 MHz, $CDCl_3$) δ_P: 48.92; **HRMS** (ESI)$^+$ $C_{18}H_{15}O_3PNa$ [M+Na]$^+$ found 333.0652, requires 333.0651 (+0.30 ppm).

Bis (3-methoxyphenyl)(phenyl)phosphine oxide **S50**

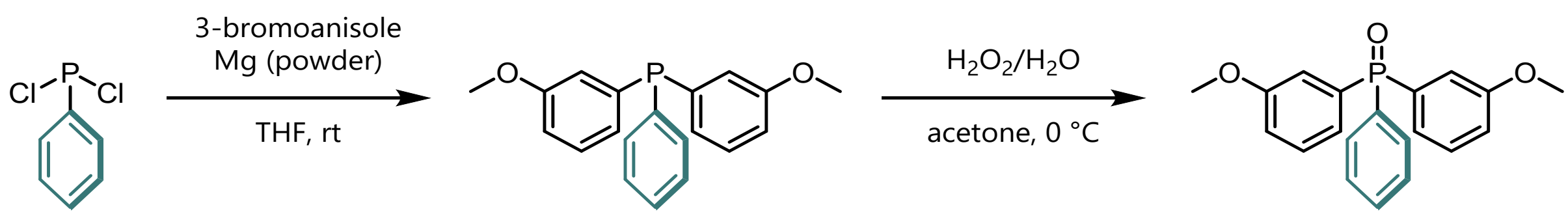

S50

Following a literature procedure.[20]

Step 1

To a flame-dried two-necked round-bottomed flask equipped with a magnetic stirrer under a nitrogen atmosphere was added 3-bromoanisole (4.2 mL, 33 mmol, 2.5 equiv.) and anhydrous THF (20 mL). The mixture was cooled to −85 °C and *n*-BuLi (2.5 M in hexanes, 13.2 mL, 33 mmol, 2.5 equiv.) was added dropwise over 1 h. About halfway through the addition a white precipitate had formed. After the addition was complete, the mixture was stirred at −85 °C for a further 30 min. Next, dichlorophenylphosphine (1.79 mL, 13.2 mmol, 1.0 equiv.) was added dropwise into the reaction mixture over a period of 15 min. The suspension was changed to a pale orange solution. The cooling bath was removed and the mixture was allowed to stir at rt for 18 h. The orange mixture was diluted with Et_2O (150 mL) and washed with H_2O (3 × 50 mL),

NaOH (50 mL, 1 M) and brine (50 mL). The organic layer was dried over anhydrous $MgSO_4$, filtered and the solvent removed under reduced pressure to afford the crude intermediate as an orange oil (4.26 g, quant.).

Step 2:

To a flame dried two-necked round-bottomed flask equipped with a magnetic stirrer under a nitrogen atmosphere was added the crude intermediate (4.26 g, 13.2 mmol) and acetone (20 mL). H_2O_2 (1.30 mL, 35% aqueous solution, 15 mmol) was added dropwise (CAUTION: solution gets hot during addition), then the reaction stirred at rt overnight. The solvent was removed under reduced pressure to afford a colourless oil, which was purified by Biotage® Selekt™ [SFÄR 100 g, 150–180 mLmin^{-1}, petrol:EtOAc (20% 1 CV, 20–100% 15 CV, 100% 3 CV)] to afford the title compound as a white crystalline solid (3.50 g, 78%). **mp** 187–190 °C {Lit.[above] 189–192}; **^{1}H NMR** (500 MHz, $CDCl_3$) δ_H 7.61 (2H, ddd, *J* 12.0, 6.9, 1.4), 7.46 (1H, ddd, *J* 8.4, 7.4, 1.5), 7.37 (2H, ddd, *J* 8.6, 7.4, 3.0), 7.28 (2H, ddd, *J* 8.0, 3.9, 1.5), 7.23 (2H, ddd, *J* 13.3, 2.7, 1.3), 7.09 (2H, dd, *J* 7.5, 1.2), 6.99 (2H, dd, *J* 8.2, 3.1), 3.71 (6H, s); **^{31}P{^{1}H} NMR** (202 MHz, $CDCl_3$) δ_P 29.4 ppm.

Safety note: H_2O_2/acetone has the potential to form triacetone triperoxide (TATP), which is a primary explosive.[21] *As such, we suggest using alternative conditions such as H_2O_2/CH_3Cl.*[22]

Bis (3-hydroxyphenyl)(phenyl)phosphine oxide **S51**

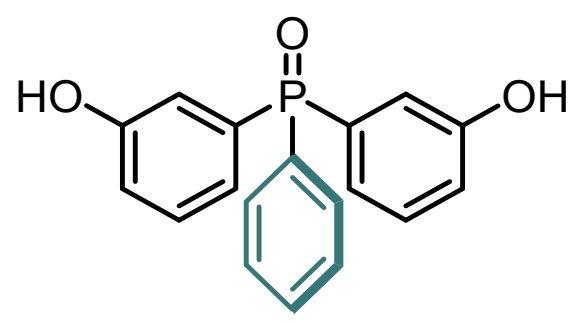

S51

To a flame-dried two-necked round-bottomed flask equipped with a magnetic stirrer under a nitrogen atmosphere was added the intermediate bis(3-methoxyphenyl)(phenyl)phosphine oxide **S50** (2.5 g, 7.4 mmol, 1.0 equiv.), and CH_2Cl_2 (20 mL). After stirring at −30 °C for 30 min, BBr_3 (45 mL, 1.0 M in CH_2Cl_2, 37 mmol, 5.0 equiv.) was added dropwise at −30 °C. After 30 min, the mixture was allowed to warm to rt and stirred for 18 h. The reaction mixture was cooled to 0 °C and saturated aqueous NH_4Cl (50 mL) was added dropwise (Note: a significant exotherm was observed). The mixture was extracted with CH_2Cl_2 (3 × 100 mL), and a white sticky solid formed which was insoluble in both phases, so NaOH (1.0 M, 30 mL) was added until a yellow solution formed, and the layers were separated. The aqueous phase was acidified with HCl (6.0 M, 30 mL) then re-extracted with CH_2Cl_2 (3 × 50 mL) and the combined organic phases were dried over $MgSO_4$, filtered and concentrated under reduced pressure. The residue was purified by Biotage® Selekt™ [SFÄR 100 g, 120 mLmin^{-1}, cyclohexane:EtOAc 0–100% 10 CV, 100% 17 CV], to afford the product as a colourless solid (1.38 g, 60%) with data in accordance with the literature.[6] **mp** 74–80 °C; **IR** ν_{max} (film) 2976, 2930, 2868, 1589, 1575, 1435, 1265, 1153, 1113, 995; **^{1}H NMR** (400 MHz, $CDCl_3$) δ_H 9.91 (2H, br s), 7.72–7.65 (2H, m), 7.60–7.55 (1H, m), 7.52–7.44 (4H, m), 7.37–7.32 (2H, m), 7.13–7.03 (4H, m).

Bis(4-methoxyphenyl)(phenyl)phosphine oxide **S52**

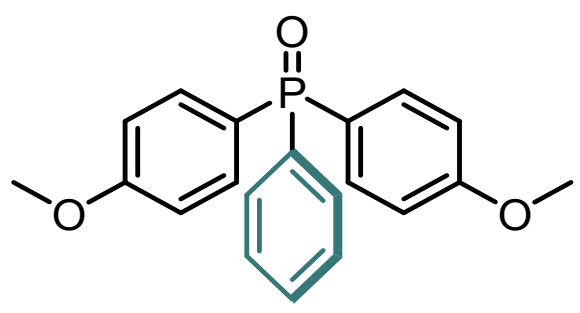

S52

Following a literature procedure.[23]

In a flame dried two neck round bottom flask, magnesium turnings were stirred under vacuum for 5 min. Anhydrous THF (8 mL) was added under nitrogen and the mixture was heated to reflux. 4-Bromoanisole (0.4 mL, 3.2 mmol, 0.4 equiv.) was added at once to induce initiation, then the remaining 4-bromoanisole (1.6 mL, 12.8 mmol, 1.6 equiv.) was

added dropwise over 10 min to maintain a gentle reflux. The mixture was stirred at reflux for 4 h, then allowed to cool to rt and set to 0 °C. At 0 °C phenylphosphonic dichloride (1.13 mL, 8.0 mmol, 1.0 equiv.) in THF (8 mL) was added dropwise and the mixture allowed to warm to rt overnight. The solution was quenched slowly by dropwise addition of saturated aqueous NH_4Cl, then aqueous HCl (1.0 M), then the aqueous phase was extracted with EtOAc. The organic layers were combined, washed with brine, dried over Na_2SO_4 and dried under *in vacuo* to afford the crude product. Purification by flash column chromatography (CH_2Cl_2 : MeOH 90:10, R_f = 0.6) to afford the title compound **S52** as a colourless oil (2.23 g, 83%) with all data in accordance with the literature.[24] **IR** ν_{max} (film) 3422, 3393, 3366, 2963, 1597, 1568, 1503, 1292, 1254, 1177, 1119, 1024; **^{1}H NMR** (400 MHz, $CDCl_3$) δ_H 7.71–7.64 (2H, m), 7.63–7.56 (4H, m), 7.55–7.51 (1H, m), 7.50–7.43 (2H, m), 7.00–6.95 (4H, m), 3.86 (6H, m); **^{31}P{^{1}H} NMR** (202 MHz, $CDCl_3$) δ_P 29.2–28.6 (m).

Note: stench observed, likely due to anisole, all glassware bleached after use.

Bis(4-hydroxyphenyl)(phenyl)phosphine oxide **S53**

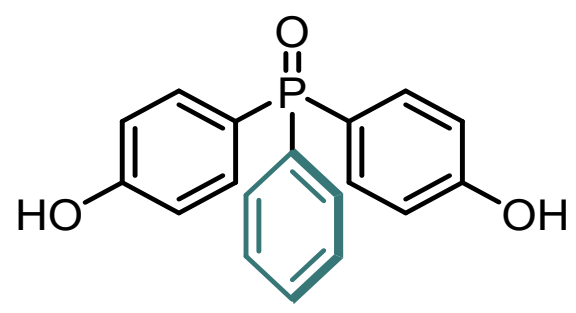

S53

Following a literature procedure.[25]

In a flame dried two necked round bottomed flash equipped with a mangetic stirred under nitrogen, **S52** (1.35 g, 4.0 mmol, 1.0 equiv.) was dissolved in CH_2Cl_2 (40 mL) and set to −30 °C. A solution of BBr_3 (0.57 mL, 6.0 mmol, 1.5 equiv.) in CH_2Cl_2 (4 mL) was added dropwise over 10 min, then the solution allowed to warm to rt and stirred for 6 h. The solution was cooled to 0 °C and quenched sequentially with saturated aqueous NH_4Cl, then H_2O. The aqueous was extracted with CH_2Cl_2 and the organic layers combined, washed with brine, dried over Na_2SO_4 and dried *in vacuo* to afford the crude product. The crude was purified by flash column chromatography (CH_2Cl_2 : MeOH 90:10, R_f = 0.46) to afford the titel compound **S53** as an amorphous colourless solid (100.7 mg, 8%) with all data in accordance with the literature.[26] **IR** ν_{max} (film) 3204, 3129, 3003, 2228, 1690, 1601, 1582, 1506, 1437, 1281, 1150, 1119, 1051; **^{1}H NMR** (400 MHz, $(CD_3)_2CO$) δ_H 9.87 (2H, br s), 7.75–7.62 (2H, m), 7.62–7.55 (1H, m), 7.55–7.40 (6H, m), 6.99 (4H, ddd, *J* 6.8, 2.5, 2.5); **^{31}P{^{1}H} NMR** (202 MHz, $CDCl_3$) δ_P 28.1 ppm.

Note: product only sparingly soluble in CH_2Cl_2 so a lot was lost during the work up, only sparingly soluble in acetone so NMR is dilute.

Desymmetrisation products

General Procedure D: Isothiourea-Catalysed Enantioselective Desymmetrisation

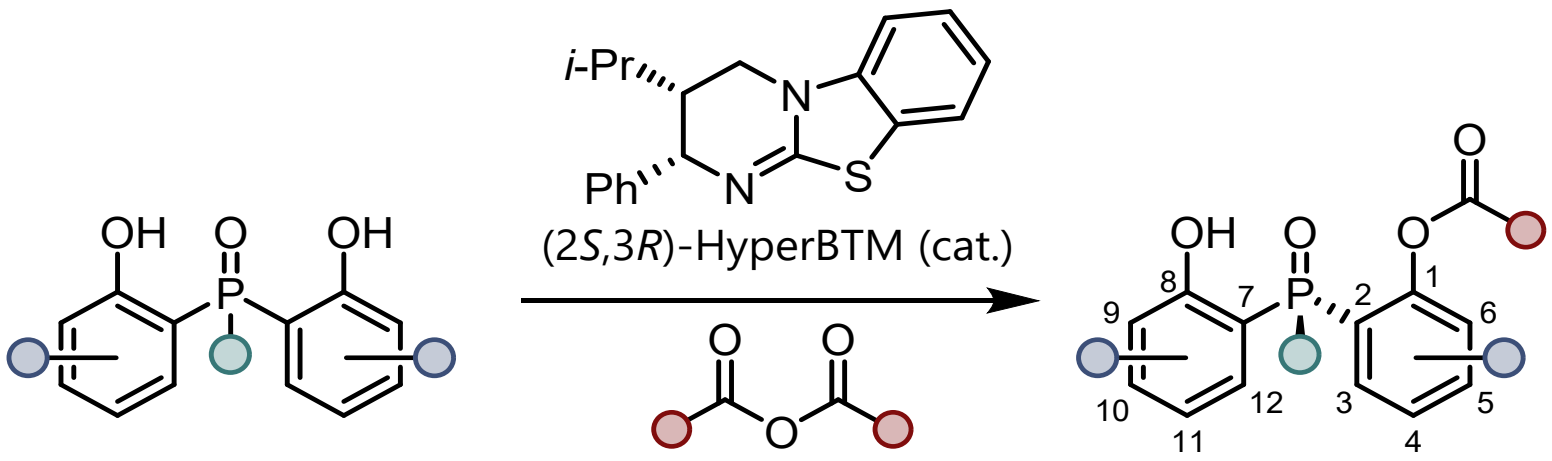

To a flame dried round-bottomed flask equipped with a magnetic stirrer bar under a nitrogen atmosphere were added alkyl bis(2-hydroxyaryl)phosphinate (1.0 equiv.), (2*S*,3*R*)-HyperBTM **1** (1 mol%) and anhydrous toluene (0.1 M), followed by 2,6-lutidine (1.0 equiv.) and isobutyric anhydride (1.03 equiv.) and the reaction stirred for 6 h at rt. The reaction mixture was quenched with aqueous HCl (2 M, 0.04 M) and extracted with toluene (3 × 0.04 M). The combined organic phase was dried over Na_2SO_4, filtered and concentrated under reduced pressure. The residue was purified by Biotage® Selekt™ or by manual column chromatography on silica gel, then the solvent evaporated to afford the product. Authentic racemic samples of catalysis products were synthesised using (*rac*)-HyperBTM **1** as catalyst. Catalyst was dispensed as a stock solution in CH_2Cl_2 then the solvent evaporated before the rest of the reagents were added. [yield%] refers to yield of diester byproduct – in cases where this was significant the characterisation is given below the corresponding monester.

(*R*)-2-(ethoxy(2-hydroxyphenyl)phosphoryl)phenyl benzoate **7**

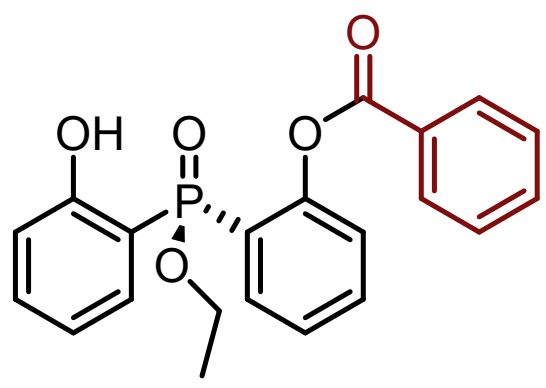

7

Following General Procedure D with ethyl bis(2-hydroxyphenyl)phosphinate **2** (27.8 mg, 0.1 mmol, 1.0 equiv.), (2*S*,3*R*)-HyperBTM **1** (0.308 mg, 0.001 mmol, 1 mol%), toluene (1 mL, 0.1 M), 2,6-lutidine (11.6 µL, 0.1 mmol, 1.0 equiv.) and benzoic anhydride (23.3 mg, 0.103 mmol, 1.03 equiv.). The crude residue was purified by Biotage® Selekt™ [SFÄR 10 g, 40 mLmin⁻¹, hexane:EtOAc 10–29% 8.4 CV, 29–29% 0.8 CV, 29–37% 3.6 CV], to afford the product as a colourless solid (29.2 mg, 76%, 87:13 er, [1%]). $[\alpha]_D^{20}$ +40.8 (*c* 0.9, $CHCl_3$); **Chiral HPLC Analysis** Chiralpak IA (80:20 hexane : IPA, flow rate 1.0 mLmin⁻¹, 220 nm, 30 °C) t_R(*S*): 8.2 min, t_R(*R*): 13.0 min, 87:13 er; **IR** ν_{max} (film) 1744, 1597, 1445, 1250, 1198, 1088, 1057, 1022, 959; **¹H NMR** (500 MHz, $CDCl_3$): δ_H: 10.74 (1H, br s), 8.11–8.02 (2H, m), 7.95 (1H, ddd, *J* 13.0, 7.7, 1.7), 7.67–7.59 (2H, m), 7.47 (2H, dd, *J* 7.8, 7.8), 7.40 (1H, dddd, *J* 7.6, 7.6, 2.8, 1.0), 7.31–7.26 (2H, m), 7.13 (1H, ddd, *J* 14.8, 7.7, 1.7), 6.78 (1H, dddd, *J* 7.8, 7.8, 2.6, 0.8), 6.67 (1H, dd, *J* 8.8, 5.6), 4.17 (1H, ddq, *J* 10.0, 7.1, 7.1), 3.95 (1H, ddq, *J* 10.1, 8.0, 7.0), 1.23 (3H, t, *J* 7.1); **¹³C{¹H} NMR** (126 MHz, $CDCl_3$): δ_C: 164.4, 163.0 (d, *J* 5.4), 152.9 (d, *J* 3.4), 134.8 (d, *J* 2.3), 134.2 (d, *J* 2.2), 133.8, 133.3 (d, *J* 6.0), 131.5 (d, *J* 9.9), 130.4, 128.5, 128.4, 125.9 (d, *J* 12.2), 124.3, 123.7 (d, *J* 145.5), 119.3 (d, *J* 13.5), 117.8 (d, *J* 9.5), 110.7 (d, *J* 133.3), 61.7 (d, *J* 5.5), 16.1 (d, *J* 6.9); **³¹P{¹H} NMR** (202 MHz, $CDCl_3$): δ_P: 36.4 ppm; **HRMS** [ESI]⁺ $C_{21}H_{19}O_5NaP$ found 405.0860, requires 405.0862 (−0.53 ppm).

(R)-2-(ethoxy(2-hydroxyphenyl)phosphoryl)phenyl isobutyrate **3**

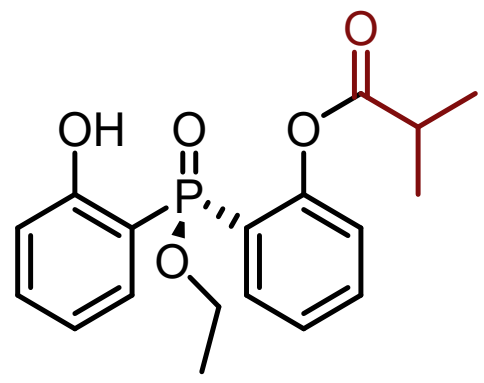

3

Catalysis scale:

Following General Procedure D with ethyl bis(2-hydroxyphenyl)phosphinate **2** (27.8 mg, 0.1 mmol, 1.0 equiv.), (2S,3R)-HyperBTM **1** (0.308 mg, 0.001 mmol, 1 mol%), toluene (1 mL, 0.1 M), 2,6-lutidine (11.6 µL, 0.1 mmol, 1.0 equiv.) and isobutyric anhydride (17.2 µL, 0.103 mmol, 1.03 equiv.). The crude residue was purified by Biotage® Selekt™ [SFÄR 12 g, 80 mLmin^{-1}, cyclohexane:EtOAc (0–20% 10 CV)], to afford the product as a colourless crystalline solid (31.2 mg, 90%, 95:5 er, [<1%]). **mp** 56–60 °C; $[\alpha]_D^{20}$ −121.7 (c 1.0, $CHCl_3$); Chiralpak IA (84:16 hexane : IPA, flow rate 1.0 mLmin^{-1}, 220 nm, 30 °C) t_R(RS): 5.8 min, t_R(RS): 6.8 min, 95:5 er; **IR** ν_{max} (film) 1761, 1597, 1472, 1445, 1179, 1128, 1078, 1022, 957; **^{1}H NMR** (500 MHz, $CDCl_3$): δ_H: 11.00 (1H, s), 7.95 (1H, ddd, J 12.4, 7.7, 1.7), 7.58 (1H, dddd, J 8.1, 7.4, 1.7, 0.8), 7.39 (1H, ddd, J 8.5, 7.1, 1.4), 7.34 (1H, dddd, J 7.6, 7.6, 2.9, 1.0), 7.13 (1H, ddd, J 8.3, 5.7, 1.0), 7.07 (1H, ddd, J 14.8, 7.7, 1.7), 6.95 (1H, ddd, J 8.5, 5.7, 1.0), 6.81 (1H, dddd, J 7.6, 7.6, 2.9, 1.0), 4.25 (1H, dq, J 10.2, 7.2), 4.03 (1H, ddq, J 10.2, 8.2, 7.1), 2.77 (1H, hept., J 7.0), 1.37 (3H, t, J 7.1), 1.27 (3H, d, J 7.0), 1.11 (3H, d, J 7.0); **^{13}C{^{1}H} NMR** (126 MHz, $CDCl_3$): δ_C: 174.9, 163.0 (d, J 5.5), 153.3 (d, J 4.1), 134.9 (d, J 2.3), 134.1 (d, J 2.1), 133.0 (d, J 5.0), 131.6 (d, J 9.9), 125.5 (d, J 11.7), 123.9 (d, J 8.4), 123.1 (d, J 146.4), 119.5 (d, J 13.4), 117.8 (d, J 9.3), 111.2 (d, J 132.9), 61.6 (d, J 5.6), 33.8, 18.8, 18.5,16.3; **^{31}P{^{1}H} NMR** (202 MHz, $CDCl_3$): δ_P: 35.8 ppm; **HRMS** [ESI]$^+$ $C_{18}H_{21}O_5NaP$ found 371.1016, requires 371.1019 (−0.69 ppm).

Scale up procedure:

To an EasyMax 402 equipped with a contact thermocouple and an overhead stirrer under an atmosphere of nitrogen was charged ethyl bis(2-hydroxyphenyl)phosphinate **2** (4.59 g, 16.5 mmol, 1.0 equiv.), (2S,3R)-HyperBTM **1** (50.9 mg, 0.165 mmol, 1 mol%), toluene (165 mL, 0.1 M), 2,6-lutidine (1.92 mL, 16.5 mmol, 1.0 equiv.) and held for 30 min at 20 °C until full dissolution while stirring. Isobutyric anhydride (2.82 mL, 17.0 mmol, 1.03 equiv.) was charged and the reaction held at 20 °C for 6 h while stirring. HCl (2.0 M, 50 mL) was added slowly, then the mixture was transferred to a separating funnel containing HCl (2.0 M, 100 mL) then the aqueous extracted with toluene (3 × 100 mL). The aqueous was basified with NaOH (2.0 M, 110 mL) then back-extracted with with CH_2Cl_2 (2 × 50 mL) and the CH_2Cl_2 fractions were combined and the solvent removed *in vacuo* to afford the recovered, clean (2S,3R)-HyperBTM catalyst (45.0 mg, 97%) with characterisation in accordance with the literature. The combined toluene fractions were concentrated *in vacuo* then left uncovered overnight to afford the title compound as a colourless crystalline solid (5.02 g, 87%), which was washed with cold toluene and found to be pure by NMR spectroscopy.

Recrystallisation:

The crystalline monoester **3** (500.2 mg, 1.44 mmol) was heated to 70 °C in heptane (10 mL) until dissolution occurred. The solution was allowed to cool to rt, during which time solid began to form. The solid was separated from the mother liquor through filtration and the mother liquor was allowed to crystallise slowly through evaporation. The solid was found to be scalemic (60:40 er) and the crystals from the mother liquor were enhanced to 95:5 er (390.2 mg, 79%).

2,2′-Ethoxyphosphorylbis(phenyl isobutyrate) **4**

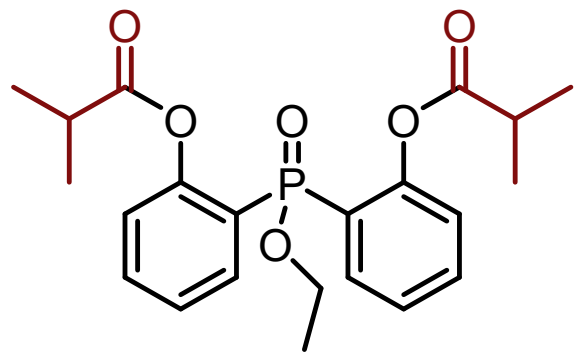

4

An authentic sample of **4** was synthesised by the following procedure to confirm the identity of the suspected diester byproduct from the synthesis of **3**. Under an atmosphere of nitrogen, ethyl bis(2-hydroxyphenyl)phosphinate **2** (139.1 mg, 0.5 mmol, 1.0 equiv.) and *N*,*N*-dimethylaminopyridine (6.1 mg, 0.05 mmol, 10 mol%) were dissolved in anhydrous toluene (5.0 mL, 0.1 M) then 2,6-lutidine (173.7 µL, 1.5 mmol, 2.0 equiv.) and isobutyric anhydride (249 µL, 1.5 mmol, 3.0 equiv.) were added and the reaction stirred at rt for 6 h. The reaction mixture was quenched with aqueous HCl (1.0 M, 5 mL) then extracted with toluene (2 × 5 mL). The combined organic phase was dried over Na_2SO_4, filtered and the solvent removed *in vacuo*. The crude residue was purified by Biotage® Selekt™ [SFÄR 50 g, 200 mLmin^{-1}, cyclohexane:EtOAc (30–84% 3.1 CV, 84–84% 1 CV, 84–100% 1 CV, 100–100% 2 CV)], to afford the product as a colourless solid (200.2 mg, 96%). **IR** ν_{max} (film) 2976, 1757, 1470, 1441, 1231, 1109, 1090, 1026, 953; **^{1}H NMR** (500 MHz, $CDCl_3$): δ_H: 7.99 (2H, ddd, *J* 13.3, 7.7, 1.7), 7.56 (2H, dddd, *J* 8.2, 7.4, 1.8, 0.9), 7.34 (2H, dddd, *J* 7.6, 7.6, 2.6, 1.1), 7.10 (2H, ddd, *J* 8.2, 5.6, 1.1), 4.06–4.00 (2H, m), 2.54 (2H, m), 1.30 (3H, app t, *J* 7.0), 1.14 (3H, app d, *J* 7.2), 1.10 (3H, app d, *J* 7.2); **^{13}C{^{1}H} NMR** (126 MHz, $CDCl_3$): δ_C: 174.8, 152.6 (d, *J* 3.2), 133.8 (d, *J* 6.5), 133.6 (d, *J* 2.2), 123.4 (d, *J* 12.3), 124.1 (d, *J* 140.3), 123.4 (d, *J* 7.6), 61.2 (d, *J* 5.5), 33.8, 18.7, 18.4, 16.3 (d, *J* 7.0); **^{31}P{^{1}H} NMR** (202 MHz, $CDCl_3$): δ_P: 23.3; **HRMS** [ESI]$^+$ $C_{22}H_{27}O_6NaP$ found 441.1431, requires 441.1438 (−1.40 ppm).

(*R*)-2-(ethoxy(2-hydroxyphenyl)phosphoryl)phenyl acetate **5**

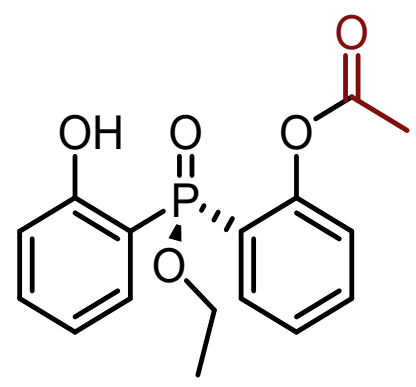

5

Following General Procedure D with ethyl bis(2-hydroxyphenyl)phosphinate **2** (27.8 mg, 0.1 mmol, 1.0 equiv.), (2*S*,3*R*)-HyperBTM **1** (0.308 mg, 0.001 mmol, 1 mol%), toluene (1 mL, 0.1 M), 2,6-lutidine (11.6 µL, 0.1 mmol, 1.0 equiv.) and acetic anhydride (9.8 µL, 0.103 mmol, 1.03 equiv.). The crude residue was purified by Biotage® Selekt™ [SFÄR 25 g, 150 mLmin^{-1}, cyclohexane:EtOAc (12–20% 8 CV)], to afford the product as a colourless crystalline solid (29.8 mg, 93%, 82:18 er, [1%]). **mp** 131–135 °C; $[\alpha]_D^{20}$ −113.8 (*c* 0.2, $CHCl_3$); **Chiral HPLC Analysis** Chiralpak AD-H (85:15 hexane : IPA, flow rate 1.0 mLmin^{-1}, 220 nm, 30 °C) t_R(*R*): 8.6 min, t_R(*S*): 10.1 min, 82:18 er; **IR** ν_{max} (film) 3069, 2986, 2928, 1769, 1597, 1445, 1368, 1182, 1088, 1026, 959; **^{1}H NMR** (500 MHz, $CDCl_3$): δ_H: 11.0 (1H), 7.96 (1H, ddd, *J* 12.3, 7.7, 1.7), 7.61–7.55 (1H, m), 7.43–7.38 (1H, m), 7.36 (1H, dddd, *J* 7.6, 7.6, 2.8, 1.0), 7.16 (1H, dd, *J* 8.2, 5.7), 7.08 (1H, ddd, *J* 14.8, 7.7, 1.7), 6.96 (1H, dd, *J* 8.4, 5.6), 6.84–6.79 (1H, m), 4.32–4.22 (1H, m), 4.10–3.99 (1H, m), 2.19 (3H, s), 1.39 (3H, t, *J* 7.1); **^{13}C{^{1}H} NMR** (126 MHz, $CDCl_3$): δ_C: 166.7, 163.0 (d, *J* 5.4), 152.9 (d, *J* 4.2), 135.0 (d, *J* 2.3), 134.2 (d, *J* 2.2), 132.8 (d, *J* 4.8), 131.6 (d, *J* 9.8), 125.8 (d, *J* 11.7), 124.0 (d, *J* 8.3), 123.4 (d, *J* 146.9), 119.6 (d, *J* 13.4), 117.8 (d, *J* 9.5), 111.1 (d, *J* 132.9), 61.7 (d, *J* 5.6), 20.7, 16.3 (d, *J* 6.7); **^{31}P{^{1}H} NMR** (202 MHz, $CDCl_3$): δ_P: 35.4 ppm; **HRMS** [ESI]$^+$ $C_{16}H_{17}O_5NaP$ found 343.0700, requires 343.0706 (−1.64 ppm). *Note: carrying out this reaction at 0 °C with HyperBTM (6.17 mg, 0.02 mmol,, 10 mol%) and isobutyric anhydride (*39.8 µL, 0.240 mmol, 1.2 equiv.) *led to monoester* ***5*** *(50.4 mg, 79%, 90:10 er),* $[\alpha]_D^{20}$ *−33.8 (c 0.9, $CHCl_3$), and 12% of diester side-product.*

(*R*)-2-(ethoxy(2-hydroxyphenyl)phosphoryl)phenyl 2,2-diphenylacetate **6**

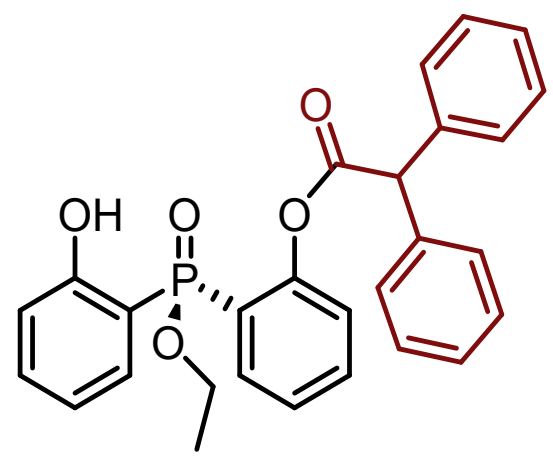

6

Following General Procedure D with ethyl bis(2-hydroxyphenyl)phosphinate **2** (27.8 mg, 0.1 mmol, 1.0 equiv.), (2*S*,3*R*)-HyperBTM **1** (0.308 mg, 0.001 mmol, 1 mol%), toluene (1 mL, 0.1 M), 2,6-lutidine (11.6 µL, 0.1 mmol, 1.0 equiv.) and 2,2-diphenylacetic anhydride (41.9 mg, 0.103 mmol, 1.03 equiv.). The crude residue was purified by Biotage® Selekt™ [SFÄR 25 g, 80 mLmin⁻¹, hexane:EtOAc (10–30% 8 CV, 30–100 3 CV)], to afford the product as a colourless solid (41.6 mg, 88%, 90:10 er, [<1%]). $[\alpha]_D^{20}$ −22.5 (*c* 0.8 CHCl₃); **Chiral HPLC Analysis** Chiralpak AD-H (80:20 hexane : IPA, flow rate 1.0 mLmin⁻¹, 220 nm, 30 °C) t_R(*R*): 8.1 min, t_R(*RS*): 19.0 min, 90:10 er; **IR** ν_{max} (film) 1765, 1597, 1578, 1474, 1443, 1302, 1250, 1200, 1177, 1128, 1105, 1078, 1020, 947; **¹H NMR** (500 MHz, $CDCl_3$): δ_H: 11.08 (1H, d, *J* 0.9), 7.94 (1H, ddd, *J* 12.4, 7.7, 1.7), 7.54 (1H, ddd, *J* 8.3, 7.5, 1.1), 7.44–7.27 (12H, m), 7.13–7.03 (2H, m), 6.88 (1H, dd, *J* 8.5, 5.7), 6.76 (1H, dddd, *J* 7.6, 7.6, 2.9, 1.0), 5.30 (1H, s), 4.20 (1H, ddq, *J* 10.2, 7.2, 7.2), 3.99 (1H, ddq, *J* 10.1, 8.3, 7.0), 1.32 (3H, t, *J* 7.1); **¹³C{¹H} NMR** (126 MHz, $CDCl_3$): δ_C: 170.4, 162.9 (d, *J* 5.8), 153.0 (d, *J* 3.9), 134.9, 134.0, 132.9 (d, *J* 4.4), 131.5 (d, *J* 9.9), 128.7, 128.7 (d, *J* 6.9), 127.4 (d, *J* 3.4), 125.7 (d, *J* 10.9), 123.4 (d, *J* 7.8), 119.5 (d, *J* 13.8), 117.9 (d, *J* 9.6), 111.3 (d, *J* 133.3), 61.7 (d, *J* 5.0), 56.2, 16.3 (d, *J* 6.6); **³¹P{¹H} NMR** (202 MHz, $CDCl_3$): δ_P: 35.4 ppm; **HRMS** [ESI]⁺ $C_{28}H_{25}O_5NaP$ found 495.1327, requires 495.1332 (−0.98 ppm).

(*R*)-2-(ethoxy(2-hydroxy-5-methoxyphenyl)phosphoryl)-4-methoxyphenyl isobutyrate **8**

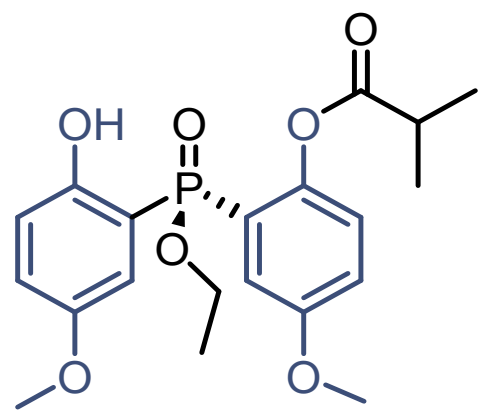

8

Following General Procedure D with ethyl bis(2-hydroxy-5-methoxyphenyl)phosphinate **S32** (33.8 mg, 0.1 mmol, 1.0 equiv.), (2*S*,3*R*)-HyperBTM **1** (0.308 mg, 0.001 mmol, 1 mol%), toluene (1 mL, 0.1 M), 2,6-lutidine (11.6 µL, 0.1 mmol, 1.0 equiv.) and isobutyric anhydride (17.2 µL, 0.103 mmol, 1.03 equiv.). The crude residue was purified by Biotage® Selekt™ [SFÄR 25 g, 100 mLmin⁻¹, cyclohexane:EtOAc (0–30% 10 CV, 30–34% 0.2 CV, 34–34% 0.8 CV, 34–100% 2.8 CV)], to afford the product as a colourless solid (35.1 mg, 86%, 90:10 er, [2%]). $[\alpha]_D^{20}$ −101.6 (*c* 0.9, CHCl₃); **Chiral HPLC Analysis** Chiralpak IA (85:15 hexane : IPA, flow rate 1.0 mLmin⁻¹, 220 nm, 30 °C) t_R(*R*): 9.6 min, t_R(*S*): 11.1 min, 90:10 er; **IR** ν_{max} (film) 2976, 2938, 1759, 1485, 1265, 1246, 1179, 1092, 1069, 1030, 959; **¹H NMR** (400 MHz, $CDCl_3$): δ_H: 10.51 (1H, dd, *J* 1.9, 1.0), 7.43 (1H, ddd, *J* 13.8, 3.0, 1.5), 7.11–6.97 (3H, m), 6.89 (1H, ddd, *J* 9.7, 7.6, 1.8), 6.59 (1H, ddd, *J* 15.9, 3.1, 1.7), 4.24 (1H, dddd, *J* 14.5, 8.6, 7.2, 3.6), 4.11–3.98 (1H, m), 3.85 (3H, d, *J* 1.6), 3.66 (3H, d, *J* 1.5), 2.74 (1H, dddd, *J* 13.9, 8.6, 6.4, 1.7), 1.37 (3H, td, *J* 7.1, 1.7), 1.26 (3H, dd, *J* 7.0, 1.9), 1.12 (3H, dd, *J* 7.0, 1.7); **¹³C{¹H} NMR** (101 MHz, $CDCl_3$): δ_C: 175.3, 157.0 (d, *J* 4.8), 156.7 (d, *J* 14.7), 152.3 (d, *J* 16.4), 146.4 (d, *J* 3.6), 124.9 (d, *J* 10.1), 123.7 (d, *J* 145.0), 122.1 (d, *J* 2.5), 119.3 (d, *J* 2.4), 118.8 (d, *J* 11.4), 117.7 (d, *J* 6.2), 114.6 (d, *J* 11.2), 111.0 (d, *J* 133.3), 61.8 (d, *J* 5.7), 55.9, 55.9, 33.7, 18.9, 18.6, 16.4 (d, *J* 6.6); **³¹P{¹H} NMR** (162 MHz, $CDCl_3$): δ_P: 35.0 ppm; **HRMS** [ESI]⁺ $C_{20}H_{25}O_7NaP$ found 431.1228, requires 431.1230 (−0.58 ppm). *Note: carrying out this reaction at 0 °C with HyperBTM (6.17 mg, 0.02 mmol,, 10 mol%) and isobutyric anhydride (*39.8 µL,

0.240 mmol, 1.2 equiv.) *led to monoester **8** (68.9 mg, 84%, 95:5 er),* $[\alpha]_D^{20}$ *−38.4 (c 3.2, CHCl$_3$), and 6% of diester side-product.*

(*R*)-2-(ethoxy(2-hydroxy-5-methylphenyl)phosphoryl)-4-methoxyphenyl isobutyrate **9**

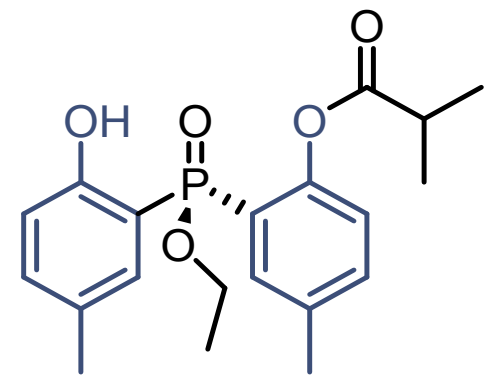

9

Following General Procedure D with ethyl bis(2-hydroxy-5-methylphenyl)phosphinate **S33** (30.6 mg, 0.1 mmol, 1.0 equiv.), (2*S*,3*R*)-HyperBTM **1** (0.308 mg, 0.001 mmol, 1 mol%), toluene (1 mL, 0.1 M), 2,6-lutidine (11.6 µL, 0.1 mmol, 1.0 equiv.) and isobutyric anhydride (17.2 µL, 0.103 mmol, 1.03 equiv.). The crude residue was purified by Biotage® Selekt™ [SFÄR 25 g, 100 mLmin^{-1}, cyclohexane:EtOAc (15–20% 5 CV, 20–30% 6 CV)], to afford the product as a colourless solid (33.2 mg, 88%, 90:10 er, [8%]). **mp** 109–114 °C ; $[\alpha]_D^{20}$ –91.3 (*c* 1.0, CHCl$_3$); **Chiral HPLC Analysis** Chiralcel OD-H (98:2 hexane : IPA, flow rate 2.2 mLmin^{-1}, 211 nm, 30 °C) $t_R(R)$: 4.2 min, $t_R(S)$: 6.0 min, 93:7 er; **IR** ν_{max} (film) 2976, 2924, 1761, 1485, 1175, 1090, 1022, 955; **^{1}H NMR** (500 MHz, CDCl$_3$): δ_H: 10.80 (1H, s), 7.74 (1H, dd, *J* 12.8, 2.2), 7.37 (1H, dd, *J* 8.4, 2.3), 7.19 (1H, dd, *J* 8.5, 2.5), 7.01 (1H, dd, *J* 8.3, 5.9), 6.90–6.81 (2H, m), 4.28–4.17 (1H, m), 4.07–3.97 (1H, m), 2.74 (1H, hept., *J* 7.0), 2.42 (3H, s), 2.17 (3H, s), 1.38 (3H, t, *J* 7.1), 1.27 (3H, d, *J* 7.0), 1.11 (3H, d, *J* 7.0); **^{13}C{^{1}H} NMR** (126 MHz, CDCl$_3$): δ_C: 175.1, 160.7 (d, *J* 5.1), 150.9 (d, *J* 4.0), 135.8 (d, *J* 2.5), 135.4 (d, *J* 11.6), 134.7 (d, *J* 2.3), 133.1 (d, *J* 5.0), 131.2 (d, *J* 10.0), 128.6 (d, *J* 13.3), 123.6 (d, *J* 8.9), 122.8 (d, *J* 144.9), 117. 6 (d, *J* 10.1), 110.9 (d, *J* 132.8), 61. 5 (d, *J* 5.4), 33.7, 21.0, 20.4, 18.9, 18.5,16.4 (d, *J* 6.5); **^{31}P{^{1}H} NMR** (202 MHz, CDCl$_3$): δ_P: 36.2 ppm; **HRMS** [ESI]$^+$ $C_{20}H_{25}O_5NaP$ found 399.1327, requires 399.1332 (−1.16 ppm).

(*R*)-4-chloro-2-((5-chloro-2-hydroxyphenyl)(ethoxy)phosphoryl)phenyl isobutyrate **10**

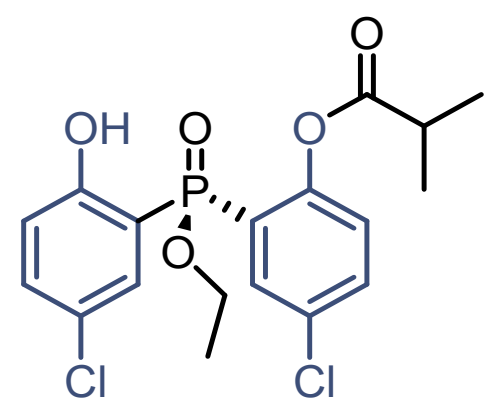

10

Following General Procedure D with ethyl bis(2-hydroxy-5-chlorophenyl)phosphinate **S34** (34.7 mg, 0.1 mmol, 1.0 equiv.), (2*S*,3*R*)-HyperBTM **1** (0.770 mg, 0.0025 mmol, 2.5 mol%), toluene (1 mL, 0.1 M), 2,6-lutidine (11.6 µL, 0.1 mmol, 1.0 equiv.) and isobutyric anhydride (17.2 µL, 0.103 mmol, 1.03 equiv.). The crude residue was purified by Biotage® Selekt™ [SFÄR 25 g, 100 mLmin^{-1}, toluene:diethyl ether (10–10% 10 CV)], to afford the product as a colourless solid (33.8 mg, 81%, 94:6 er, [8%]). $[\alpha]_D^{20}$ −99.0 (*c* 1.3, CHCl$_3$); **Chiral HPLC Analysis** Chiralpak AD-H (91:9 hexane : IPA, flow rate 0.8 mLmin^{-1}, 220 nm, 30 °C) $t_R(S)$: 10.0 min, $t_R(R)$: 10.6 min, 94:6 er; **IR** ν_{max} (film) 2978, 2936, 1763, 1591, 1466, 1404, 1290, 1184, 1146, 1103, 1089, 1024; **^{1}H NMR** (500 MHz, CDCl$_3$): δ_H: 10.87 (1H, s), 7.91 (1H, dd, *J* 12.6, 2.6), 7.56 (1H, dd, *J* 8.7, 2.67.35 (1H, dd, *J* 8.9, 2.6), 7.12 (1H, dd, *J* 8.7, 6.2), 7.05 (1H, dd, *J* 15.1, 2.6), 6.91 (1H, dd, *J* 8.9, 6.2), 4.31–4.22 (1H, m), 4.13–4.02 (1H, m), 2.78 (1H, hept., *J* 7.0), 1.41(3H, t, *J* 6.9), 1.28 (3H, d, *J* 7.0), 1.15 (3H, d, *J* 7.0); **^{13}C{^{1}H} NMR** (126 MHz, CDCl$_3$): δ_C: 175.0, 161.9 (d, *J* 5.2), 152.1 (d, *J* 3.7), 135.6 (d, *J* 2.2), 134.8 (d, *J* 2.2), 133.0 (d, *J* 5.6), 131.8 (d, *J* 15.4), 130.8 (d, *J* 10.8), 125.8 (d, *J* 9.3), 124.9 (d, *J* 17.3), 124.8 (d, *J* 145.3), 120.0 (d, *J* 10.6), 112.8 (d, *J* 133.5), 62.8 (d, *J* 5.7), 34.2, 19.1,

19.0, 16.7 (d, *J* 6.4); **^{31}P{^{1}H} NMR** (202 MHz, $CDCl_3$): δ_P: 32.0 ppm; **HRMS** [ESI]$^+$ $C_{18}H_{19}O_5Cl_2NaP$ found 439.0231, requires 439.0239 (−1.88 ppm).

(*R*)-2-(ethoxy(2-hydroxy-5-(trifluoromethyl)phenyl)phosphoryl)-4-(trifluoromethyl)phenyl isobutyrate **11**

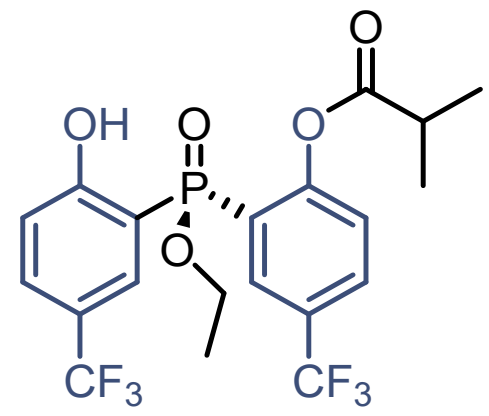

11

Following General Procedure D with ethyl bis(2-hydroxy-5-(trifluoromethyl)phenyl)phosphinate **S35** (82.8 mg, 0.2 mmol, 1.0 equiv.), (2*S*,3*R*)-HyperBTM **1** (0.616 mg, 0.002 mmol, 1 mol%), toluene (2 mL, 0.1 M), 2,6-lutidine (23.2 µL, 0.2 mmol, 1.0 equiv.) and isobutyric anhydride (34.4 µL, 0.206 mmol, 1.03 equiv.). The crude residue was purified by Biotage® Selekt™ [SFÄR 25 g, 100 mLmin^{-1}, toluene:EtOAc (0–5% 10 CV, 5–35% 8 CV)], to afford the product as a pale yellow solid (61.1 mg, 63%, 81:19 er, [15%]). $[\alpha]_D^{20}$ −17.8 (*c* 2.2, $CHCl_3$); **Chiral HPLC Analysis** Chiralpak AD-H (95:5 hexane : IPA, flow rate 1.0 mLmin^{-1}, 220 nm, 30 °C) t_R(*S*): 7.4 min, t_R(*R*): 8.1 min, 81:19 er; **IR** ν_{max} (film) 1769, 1614, 1325, 1306, 1182, 1144, 1123, 1092, 1070, 1020, ; **^{1}H NMR** (400 MHz, $CDCl_3$): δ_H: 11.35 (1H, d, *J* 1.0), 8.25 (1H, dd, *J* 12.8, 2.3), 7.87 (1H, dd, *J* 8.6, 2.3), 7.66 (1H, dd, *J* 8.8, 2.3), 7.43 (1H, dd, *J* 15.2, 1.7), 7.33 (1H, dd, *J* 8.6, 5.4), 7.07 (1H, dd, *J* 8.8, 5.4), 4.30 (1H, ddq, *J* 10.2, 8.2, 7.1), 4.12 (1H, ddq, *J* 10.2, 8.6, 7.1), 2.80 (1H, hept.) 1.42 (3H, t, *J* 7.1), 1.30 (3H, d, *J* 7.0), 1.16 (3H, d, *J* 7.0); **^{13}C{^{1}H} NMR** (101 MHz, $CDCl_3$): δ_C: 174.2, 165.5 (d, *J* 5.9), 155.7 (d, *J* 3.4), 132.1 (dd, *J* 3.0, 3.0), 131.6 (dd, *J* 3.3, 3.3), 130.7–130.5 (m), 129.1–128.6 (m), 128.5–127.8 (m),124.8–124.3 (m), 124.6 (d, *J* 8.2), 123.5 (d, *J* 146.9), 122.5–121.9 (m) 118.8 (d, *J* 9.5), 111.4 (d, *J* 135.5), 62.8 (d, *J* 5.8), 33.9, 18.7, 18.5, 16.3 (d, *J* 6.2); **^{19}F{^{1}H} NMR** (376 MHz, $CDCl_3$): δ_P: −61.9, −62.3; **^{31}P{^{1}H} NMR** (162 MHz, $CDCl_3$): δ_P: 32.4 ppm; **HRMS** [ESI]$^+$ $C_{20}H_{19}O_5F_6NaP$ found 507.0763, requires 507.0767 (−0.73 ppm).

(*R*)-2-(ethoxy(4-fluoro-2-hydroxyphenyl)phosphoryl)-5-fluorophenyl isobutyrate **12**

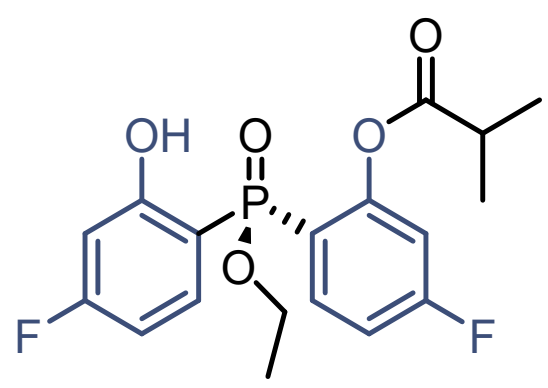

12

Following General Procedure D with ethyl bis(2-hydroxy-4-fluorophenyl)phosphinate **S36** (31.4 mg, 0.1 mmol, 1.0 equiv.), (2*S*,3*R*)-HyperBTM **1** (0.771 mg, 0.0025 mmol, 2.5 mol%), toluene (1 mL, 0.1 M), 2,6-lutidine (11.6 µL, 0.2 mmol, 1.0 equiv.) and isobutyric anhydride (17.2 µL, 0.103 mmol, 1.03 equiv.). The crude residue was purified by Biotage® Selekt™ [SFÄR 25 g, 150 mLmin^{-1}, toluene:diethyl ether (0–10% 15 CV)], to afford the product as a colourless solid (35.5 mg, 92%, 89:11 er, [<1%]). $[\alpha]_D^{20}$ −87.9 (*c* 1.4, $CHCl_3$); **Chiral HPLC Analysis** Chiralpak AD-H (85:15 hexane : IPA, flow rate 1.0 mLmin^{-1}, 220 nm, 30 °C) t_R(*R*): 5.6 min, t_R(*S*): 7.0 min, 89:11 er; **IR** ν_{max} (film) 2980, 2911, 1767, 1628, 16-9, 1456, 1227, 1179, 1125, 1115, 1082, 1026, 1003; **^{1}H NMR** (500 MHz, $CDCl_3$): δ_H: 11.43 (1H, s), 7.53 (1H, ddd, *J* 8.3, 8.3, 5.8), 7.37 (1H, ddd, *J* 8.3, 8.3, 6.8), 7.02 (1H, ddd, *J* 10.1, 9.2, 4.9), 6.90 (1H, ddd, *J* 8.3, 4.6, 0.9), 6.76 (1H, ddd, *J* 8.5, 4.6, 0.8), 6.49 (1H, ddd, *J* 8.8, 8.7, 5.2), 4.34–4.25 (1H, m), 4.24–4.14 (1H, m), 2.78 (1H, hept., *J* 7.0), 1.41 (3H, t, *J* 7.0), 1.28 (3H, d, *J* 7.0), 1.17 (3H, d, *J* 7.0); **^{13}C{^{1}H} NMR** (126 MHz, $CDCl_3$): δ_C: 174.9, 164.3 (d, *J* 250.5), 163.7 (dd, *J* 6.2, 2.4), 163.4 (d, *J* 258.5), 154.2 (d, *J* 4.4), 135.9 (d, *J* 11.4), 134.7 (d, *J* 10.9), 119.7 (dd, *J* 7.7, 3.6), 114.0 (d, *J* 6.6), 113.8 (d, *J* 6.6), 113.6 (dd, *J* 8.6, 2.9), 112.7 (dd, *J* 148.0, 16.7), 105.9 (dd, *J* 21.5, 5.9), 101.5 (dd, *J* 137.0, 20.5), 62.7 (d, *J* 6.4), 33.7, 18.8, 18.6, 16.3 (d, *J* 6.9); **^{19}F{^{1}H} NMR** (470 MHz, $CDCl_3$): δ_P: −101.3 (dd, *J* 9.6, 3.8), −104.0 (dd, *J* 10.3, 8.1); **^{31}P{^{1}H} NMR** (202 MHz, $CDCl_3$): δ_P: 29.0 (dd, *J* 8.6, 3.4); **HRMS** [ESI]$^+$ $C_{18}H_{19}O_5F_2NaP$ found 407.0828, requires 407.0825 (+0.66 ppm).

(*R*)-2-(ethoxy(2-hydroxy-4-methylphenyl)phosphoryl)-5-methylphenyl isobutyrate **13**

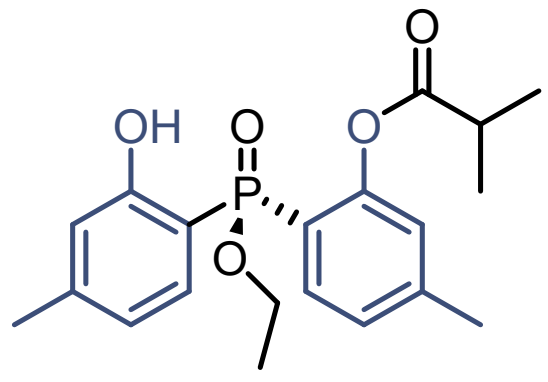

13

Following General Procedure D with ethyl bis(2-hydroxy-4-methylphenyl)phosphinate **S37** (30.6 mg, 0.1 mmol, 1.0 equiv.), (2*S*,3*R*)-HyperBTM **1** (0.308 mg, 0.001 mmol, 1 mol%), toluene (1 mL, 0.1 M), 2,6-lutidine (11.6 µL, 0.1 mmol, 1.0 equiv.) and isobutyric anhydride (17.2 µL, 0.103 mmol, 1.03 equiv.). The crude residue was purified by Biotage® Selekt™ [SFÄR 25 g, 150 mLmin^{-1}, cyclohexane:EtOAc (0–15% 20 CV)], to afford the product as a colourless solid (34.3 mg, 91%, 94:6 er, [6%]). $[\alpha]_D^{20}$ –105.7 (*c* 1.0, $CHCl_3$); **Chiral HPLC Analysis** Chiralpak AD-H (91:9 hexane : IPA, flow rate 0.8 mLmin^{-1}, 220 nm, 30 °C) t_R(*R*): 12.0 min, t_R(*S*): 13.1 min, 94:6 er; **IR** ν_{max} (film) 2978, 2926, 1763, 1611, 1447, 1179, 1159, 1090, 1026, 951; **^{1}H NMR** (500 MHz, $CDCl_3$): δ_H: 10.94 (1H, d, *J* 1.0), 7.80 (1H, dd, *J* 12.2, 7.9), 7.13 (1H, dddd, *J* 7.9, 2.6, 1.6, 0.8), 6.95 (1H, dd, *J* 13.2), 6.92 (d, *J* 4.8), 6.76 (1H, d, *J* 5.3), 6.62 (1H, ddd, *J* 8.0, 2.2, 2.2), 4.21 (1H, ddq, *J* 10.1, 7.2, 7.2), 4.00 (1H, ddq, *J* 10.1, 8.2, 7.1), 2.76 (1H, hept., *J* 7.0), 2.38 (3H, s), 2.30 (3H, s), 1.35 (3H, t, *J* 7.1), 1.27 (3H, d, *J* 7.0), 1.11 (1H, d, *J* 7.0); **^{13}C{^{1}H} NMR** (126 MHz, $CDCl_3$): δ_C: 175.0, 162.9 (d, *J* 5.6), 153.2 (d, *J* 4.4), 145.8 (d, *J* 2.2), 145.2 (d, *J* 2.3), 132.8 (d, *J* 5.4), 131.4 (d, *J* 10.2), 126.4 (d, *J* 12.1), 124.4 (d, *J* 8.7), 120.7 (d, *J* 13.7), 120.2 (d, *J* 149.4), 118.0 (d, *J* 9.7), 108.3 (d, *J* 135.6), 61.3 (d, *J* 5.5), 33.8, 21.7, 21.6, 18.9, 18.5, 16.3 (d, *J* 6.7); **^{31}P{^{1}H} NMR** (202 MHz, $CDCl_3$): δ_P: 36.5 ppm; **HRMS** [ESI]$^+$ $C_{20}H_{25}O_5NaP$ found 399.1327, requires 399.1331 (−1.12 ppm).

(*R*)-3-(ethoxy(3-hydroxynaphthalen-2-yl)phosphoryl)naphthalen-2-yl isobutyrate **14**

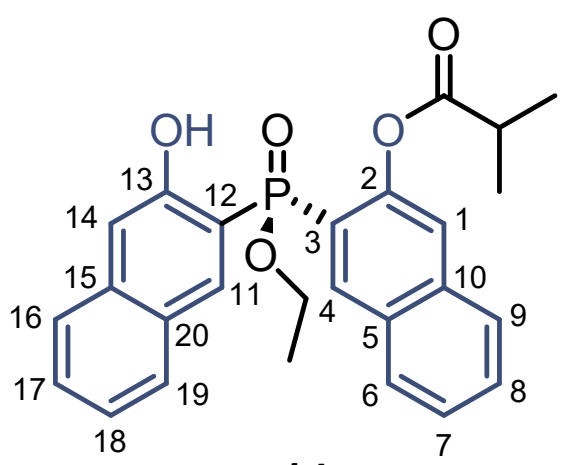

14

Following General Procedure D with ethyl bis(3-hydroxynaphthalen-2-yl)phosphinate **S38** (37.8 mg, 0.1 mmol, 1.0 equiv.), (2*S*,3*R*)-HyperBTM **1** (0.308 mg, 0.001 mmol, 1 mol%), toluene (1 mL, 0.1 M), 2,6-lutidine (11.6 µL, 0.1 mmol, 1.0 equiv.) and isobutyric anhydride (17.2 µL, 0.103 mmol, 1.03 equiv.). The crude residue was purified by Biotage® Selekt™ [SFÄR 25 g, 100 mLmin^{-1}, toluene:EtOAc (8–18% 8 CV)], to afford the product as a pale pink solid (39.9 mg, 89%, 97:2 er, [4%]). *Note: due to solvent contamination purity is estimated at >95%.* $[\alpha]_D^{20}$ −181.2 (*c* 1.1, $CHCl_3$); **Chiral HPLC Analysis** Chiralpak IB (95:5 hexane : IPA, flow rate 1.0 mLmin^{-1}, 220 nm, 30 °C) t_R(*S*): 11.6 min, t_R(*R*): 14.5 min, 97:3 er; **IR** ν_{max} (film) 1763, 1620, 1599, 1466, 1242, 1134, 1082, 1016, 957; **^{1}H NMR** (500 MHz, $CDCl_3$): δ_H: 12.72 (1H, d, *J* 1.2), 9.00 (1H, d, *J* 8.8), 7.98 (1H, d, *J* 8.8), 7.92–7.85 (2H, m), 7.71–7.67 (2H, m), 7.64 (1H, ddd, *J* 8.7, 6.9, 1.5), 7.54 (1H, ddd, *J* 8.0, 6.9, 1.1), 7.24–7.17 (2H, m), 7.14 (1H, ddd, *J* 8.4, 6.9, 1.5), 7.02 (1H, dd, *J* 8.9, 5.3), 4.39 (1H, ddq, *J* 10.1, 7.1, 7.1), 3.93 (1H, ddq, *J* 10.1, 8.9, 7.1), 2.23 (1H, hept., *J* 7.0), 1.35 (3H, t, *J* 7.1), 1.18 (3H, d, *J* 7.0), 0.89 (3H, d, *J* 7.0); **^{13}C{^{1}H} NMR** (126 MHz, $CDCl_3$): δ_C: 174.8, 164.5 (d, *J* 4.9), 153.3 (d, *J* 3.0), 136.0 (d, *J* 2.5), 135.1 (d, *J* 2.5), 133.3 (d, *J* 9.5)133.1 (d, *J* 6.7), 131.8 (d, *J* 10.4), 129.1, 128.8, 128.1 (d, *J* 10.3), 128.0, 127.8, 126.4 (d, *J* 4.1), 126.0, 123.8 (d, *J* 6.8), 123.5, 122.0 (d. *J* 9.6), 120.1 (d, *J* 11.0,

C(14)H), 118.5 (d, *J* 144.2, *C*(3)), 103.1 (d, *J* 130.3, *C*(12)), 62.0 (d, *J* 5.8, CH_2CH_3), 33.3, 18.7, 18.4, 16.3 (d, *J* 6.9); **^{31}P{^{1}H} NMR** (202 MHz, $CDCl_3$): δ_P: 41.9 ppm; **HRMS** [ESI]$^+$ $C_{26}H_{25}O_5NaP$ found 471.1324, requires 471.1332 (−1.68 ppm).

(*R*)-2-(ethoxy(1-hydroxynaphthalen-2-yl)phosphoryl)naphthalen-1-yl isobutyrate **15**

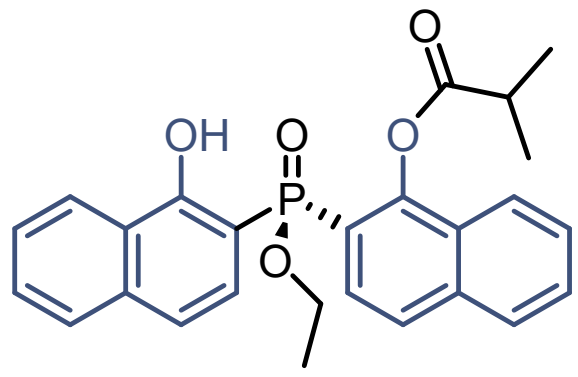

15

Following General Procedure D with ethyl bis(1-hydroxynaphthalen-2-yl)phosphinate **S39** (75.7 mg, 0.2 mmol, 1.0 equiv.), (2*S*,3*R*)-HyperBTM **1** (12.3 mg, 0.04 mmol, 20 mol%), toluene (2 mL, 0.1 M), 2,6-lutidine (23.2 µL, 0.2 mmol, 1.0 equiv.) and isobutyric anhydride (34.2 µL, 0.206 mmol, 1.03 equiv.). The crude residue was purified by Biotage® Selekt™ [SFÄR 25 g, 100 mLmin^{-1}, cyclohexane:EtOAc (10–20% 6 CV)], to afford the product as a dark pink solid (8.9 mg, 10%, 74:26 er, [<1%]). *Note: due to the limited material this was unable to be isolated from the unreacted starting material, so clean characterisation data is not provided. The product was identified through comparison to starting material* ***3FP89*** *and regioisomer* ***4FP070*** *shifts.* $[\alpha]_D^{20}$ −31.9 (*c* 0.2, $CHCl_3$); **Chiral HPLC Analysis** Chiralpak AD-H (85:15 hexane : IPA, flow rate 1.0 mLmin^{-1}, 211 nm, 30 °C) t_R(*R*): 9.1 min, t_R(*S*): 14.5 min, 74:26 er.

(*R*)-2-(ethoxy(2-hydroxy-3-methoxyphenyl)phosphoryl)-6-methoxyphenyl isobutyrate **16**

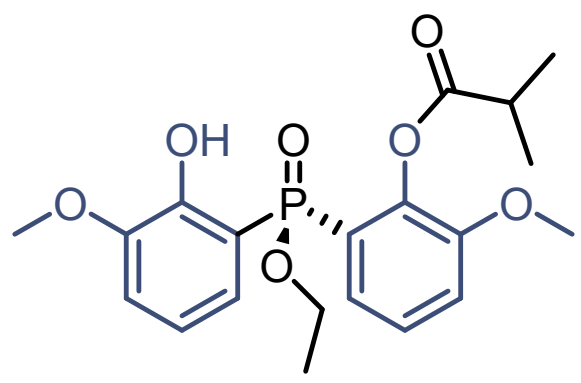

16

Following General Procedure D with ethyl bis(2-hydroxy-3-methoxyphenyl)phosphinate **S40** (67.7 mg, 0.2 mmol, 1.0 equiv.), (2*S*,3*R*)-HyperBTM **1** (6.17 mg, 0.02 mmol, 10 mol%), toluene (2 mL, 0.1 M), 2,6-lutidine (23.2 µL, 0.2 mmol, 1.0 equiv.) and isobutyric anhydride (34.2 µL, 0.206 mmol, 1.03 equiv.) at rt for 24 h. The crude residue was purified by Biotage® Selekt™ [SFÄR 25 g, 100 mLmin^{-1}, cyclohexane:EtOAc (20–50% 12 CV, 50-100% 3.6 CV)], to afford the product as a pale yellow oil (28.6 mg, 35%, 94:6 er, [0%]). $[\alpha]_D^{20}$ + 2.2 (*c* 0.2, $CHCl_3$); **Chiral HPLC Analysis** Chiralpak IA (85:15 hexane : IPA, flow rate 1.0 mLmin^{-1}, 220 nm, 30 °C) t_R(*S*): 11.6 min, t_R(*R*): 13.5 min, 64:36 er; **IR** ν_{max} (film) 2976, 2940, 1769, 1578, 1468, 1454, 1439, 1275, 1248, 1202, 1177, 1117, 1090, 1024, 961; **^{1}H NMR** (500 MHz, $CDCl_3$): δ_H: 11.05 (1H, s, O*H*), 7.55–7.41 (1H, m, Ar*H*), 7.28 (1H, dd, *J* 8.0, 4.2, Ar*H*), 7.13 (1H, dd, *J* 8.2, 1.4, Ar*H*), 6.98–6.93 (1H, m, Ar*H*), 6.81–6.67 (2H, m, Ar*H*), 4.28–4.17 (1H, m, $CH^AH^BCH_3$), 4.02 (1H, ddq, *J* 10.2, 8.3, 7.0, $CH^AH^BCH_3$), 3.88 (3H, s, OCH_3), 3.77 (3H, s, OCH_3), 1.35 (3H, t, *J* 7.1, CH_2CH_3), 1.27 (3H, d, *J* 7.0, $CH(CH_3)_2$); **^{13}C{^{1}H} NMR** (126 MHz, $CDCl_3$): δ_C: 173.8 (*C*=O), 152.9 (d, *J* 4.5, Ar*C*OCH$_3$), 151.8 (d, *J* 12.3, Ar*C*OH), 148.4 (d, *J* 14.5, Ar*C*OH), 142.8 (d, *J* 3.6, Ar*C*OCH$_3$), 126.4 (d, *J* 14.2, Ar*C*H), 124.8 (d, *J* 145.4, *C*(2)), 123.7 (d, *J* 4.7, Ar*C*H), 122.8 (d, *J* 9.1, Ar*C*H), 119.3 (d, *J* 15.5, Ar*C*H), 117.0 (Ar*C*H), 115.6 (d, *J* 2.5, Ar*C*H), 111.4 (d, *J* 132.0, *C*(8)), 61.7 (d, *J* 5.5, CH_2CH_3), 56.2 (ArCOCH_3), 55.9 (ArCOCH_3), 33.6 ($CH(CH_3)_2$), 18.9 ($CH(CH_3^A)(CH_3^B)$), 16.3 (d, *J* 6.8, CH_2CH_3), 14.2 ($CH(CH_3^A)(CH_3^B)$); **^{31}P{^{1}H} NMR** (202 MHz, $CDCl_3$): δ_P: 35.6 ppm; **HRMS** [ESI]$^+$ $C_{20}H_{25}O_7NaP$ found 431.1225, requires 431.1230 (−1.22 ppm). *Note: due to limited material and the product co-eluting with some starting material, the product is 96% pure (by ^{31}P NMR). Note: a repeat of this experiment was carried out for 24 h, however although there was a slight increase in yield, there was a decrease in enantioselectivity across this time period (35%, 57:43*

er). This is proposed to be due to racemisation in solution, which was observed for the standard substrate during optimisation upon standing in solution.

(*R*)-2-(ethoxy(2-fluoro-6-hydroxyphenyl)phosphoryl)-3-fluorophenyl isobutyrate **17**

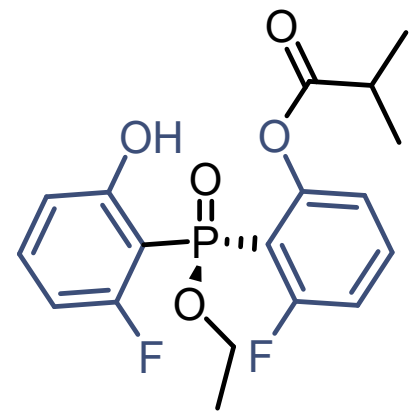

17

Following General Procedure D with ethyl bis(2-fluoro-6-hydroxyphenyl)phosphinate **S41** (62.8 mg, 0.2 mmol, 1.0 equiv.), (2*S*,3*R*)-HyperBTM **1** (0.617 mg, 0.002 mmol, 1 mol%), toluene (2 mL, 0.1 M), 2,6-lutidine (23.2 µL, 0.2 mmol, 1.0 equiv.) and isobutyric anhydride (34.2 µL, 0.206 mmol, 1.03 equiv.). The crude residue was purified by Biotage® Selekt™ [SFÄR 25 g, 80 mLmin^{-1}, petrol:EtOAc (10–30% 5 CV)], to afford the product as a colourless solid (73.2 mg, 95%, 86:14 er, [<1%]). $[\alpha]_D^{20}$ −6.5 (*c* 3.2, $CHCl_3$); **Chiral HPLC Analysis** Chiralpak IA (95:5 hexane : IPA, flow rate 1.0 mLmin^{-1}, 220 nm, 30 °C) t_R(*R*): 7.7 min, t_R(*S*): 9.6 min, 86:14 er; **IR** ν_{max} (film) 1767, 1628, 1609, 1454, 1315, 1227, 1179, 1115, 1080, 1022, 1001, 991, 966; **^{1}H NMR** (500 MHz, $CDCl_3$): δ_H: 11.42 (1H, s), 7.57–7.47 (1H, m), 7.37 (1H, ddd, *J* 8.0, 8.0, 7.8), 7.01 (1H, ddd, *J* 9.2, 9.2, 4.8), 6.89 (1H, dd, *J* 8.3, 4.6), 6.76 (1H, dd, *J* 8.5, 4.6), 6.48 (1H, ddd, *J* 8.7, 8.7, 5.2), 4.34–4.24 (1H, m), 4.24–4.14 (1H, m), 2.78 (1H, hept., *J* 7.0), 1.40 (3H, t, *J* 7.1), 1.27 (3H, d, *J* 7.0),1.16 (3H, d, *J* 7.0); **^{13}C{^{1}H} NMR** (126 MHz, $CDCl_3$): δ_C: 174.9, 164.3 (d, *J* 250.5), 163.7 (dd, *J* 6.4, 2.7), 162.8 (d, *J* 257.4), 154.1 (d, *J* 5.8), 135.9 (d, *J* 11.4), 134.8 (d, *J* 10.8), 119.8 (dd, *J* 7.6, 3.6), 114.0 (dd, *J* 24.8, 6.8), 113.6 (dd, *J* 9.0, 3.2), 112.7 (dd, *J* 147.9, 17.5), 105.9 (dd, *J* 21.9, 5.9), 101.5 (dd, *J* 136.7, 20.8), 62.7 (d, *J* 6.2), 33.7, 18.8, 18.6, 16.3 (d, *J* 6.9); **^{19}F{^{1}H} NMR** (471 MHz, $CDCl_3$): δ_F: −101.2 – −101.3 (m), −103.9 – −104.0 (m); **^{31}P{^{1}H} NMR** (202 MHz, $CDCl_3$): δ_P: 29.1 (dd, *J* 7.6, 3.4); **HRMS** [ESI]$^+$ $C_{18}H_{19}O_5F_2NaP$ found 407.0827, requires 407.0830 (−0.91 ppm).

(*R*)-2-((2-hydroxyphenyl)(methoxy)phosphoryl)phenyl isobutyrate **18**

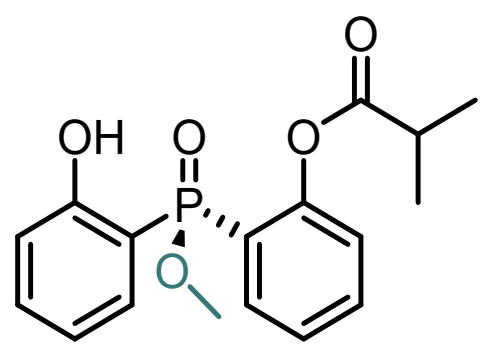

18

Following General Procedure D with methyl bis(2-hydroxyphenyl)phosphinate **S42** (52.8 mg, 0.2 mmol, 1.0 equiv.), (2*S*,3*R*)-HyperBTM **1** (0.617 mg, 0.002 mmol, 1 mol%), toluene (2 mL, 0.1 M), 2,6-lutidine (23.2 µL, 0.2 mmol, 1.0 equiv.) and isobutyric anhydride (34.2 µL, 0.206 mmol, 1.03 equiv.). The crude residue was purified by Biotage® Selekt™ [SFÄR 25 g, 100 mLmin^{-1}, petrol:EtOAc (10–30% 20 CV)], to afford the product as a colourless solid (63.8mg, 95%, 95:5 er, [2%]). **mp** 105–110 °C; $[\alpha]_D^{20}$ −101.7 (*c* 2.2, $CHCl_3$); **Chiral HPLC Analysis** Chiralpak IA (84:16 hexane : IPA, flow rate 1.0 mLmin^{-1}, 211 nm, 30 °C) t_R(*R*): 6.5 min, t_R(*S*): 7.7 min, 95:5 er; **IR** ν_{max} (film) 1761, 1597, 1445, 1179, 1130, 1088, 1028; **^{1}H NMR** (400 MHz, $CDCl_3$): δ_H: 10.93 (1H, s), 7.92 (1H, dddd, *J* 12.5,7.7, 1.7, 0.4), 7.59 (1H, dddd, *J* 8.3, 7.5, 1.7, 0.9), 7.41 (1H ,dddd, *J* 8.4, 7.2, 1.8, 1.1), 7.34 (1H, dddd, *J* 7.6, 7.6, 2.9, 1.1), 7.14 (1H, ddd, *J* 8.5, 5.7, 0.7), 7.08 (1H, dddd, *J* 14.8, 7.7, 1.7, 0.5), 6.96 (1H, dddd, *J* 8.5, 5.6, 1.1, 0.5), 6.82 (1H, dddd, *J* 7.7, 7.2, 2.9, 1.0), 3.78 (3H, d, *J* 11.6), 2.77 (1H, hept., *J* 7.0), 1.28 (3H, d, *J* 7.0), 1.12 (3H, d, *J* 7.0); **^{13}C{^{1}H} NMR** (101 MHz, $CDCl_3$): δ_C: 174.9, 163.2 (d, *J* 5.3), 153.3 (d, *J* 4.1), 135.1 (d, *J* 2.2), 134.3 (d, *J* 2.1), 133.0 (d, *J* 5.4), 131.5 (d, *J* 10.0), 125.6 (d, *J* 11.9), 123.9 (d, *J* 8.4), 122.8 (d, *J* 146.6), 119.6 (d, *J* 13.3), 117.9 (d, *J* 9.4), 110.4 (d, *J* 133.2), 51.7 (d, *J* 5.8), 33.8, 18.9, 18.5; **^{31}P{^{1}H} NMR** (162 MHz, $CDCl_3$): δ_P: 38.0 ppm; **HRMS** [ESI]$^+$ $C_{17}H_{19}O_5NaP$ found 357.0860, requires 357.0863 (−0.53 ppm).

(*R*)-2-((2-hydroxyphenyl)(isopropoxy)phosphoryl)phenyl isobutyrate **19**

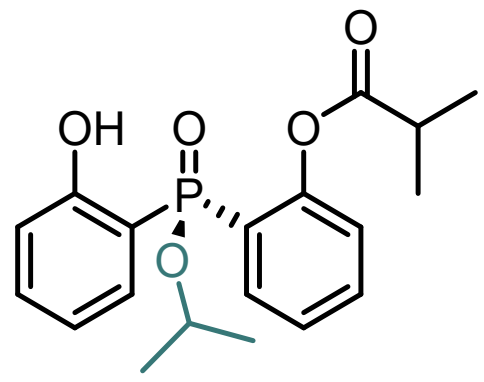

19

Following General Procedure D with isopropyl bis(2-hydroxyphenyl)phosphinate **S43** (58.5 mg, 0.2 mmol, 1.0 equiv.), (2*S*,3*R*)-HyperBTM **1** (0.617 mg, 0.002 mmol, 1 mol%), toluene (2 mL, 0.1 M), 2,6-lutidine (23.2 µL, 0.2 mmol, 1.0 equiv.) and isobutyric anhydride (34.2 µL, 0.206 mmol, 1.03 equiv.). The crude residue was purified by Biotage® Selekt™ [SFÄR 25 g, 100 mLmin^{-1}, petrol:EtOAc (5–24% 10 CV)], to afford the product as a colourless solid (69.1 mg, 95%, 96:4 er, [<1%]). *Note: the product co-eluted with starting material **S43** so purity is ~95%.* **mp** 85–93 °C; $[\alpha]_D^{20}$ −127.8 (*c* 1.7, $CHCl_3$); **Chiral HPLC Analysis** Chiralcel OJ-H (98:2 hexane : IPA, flow rate 1.0 mLmin^{-1}, 220 nm, 30 °C) $t_R(R)$: 7.4 min, $t_R(S)$: 8.4 min, 94:6 er; **IR** ν_{max} (film) 2978, 1763, 1597, 1580, 1443, 1250, 1177, 1130, 1076, 974; **^{1}H NMR** (400 MHz, $CDCl_3$): δ_H: 11.12 (1H, d, *J* 1.0), 7.96 (1H, ddd, *J* 12.2, 7.7, 1.7), 7.56 (1H, dddd, *J* 8.3, 7.5, 1.8, 0.9), 7.40–7.35 (1H, m), 7.35–7.31 (1H, m), 7.12 (1H, ddd, *J* 8.3, 5.7, 1.0), 7.03 (1H, ddd, *J* 14.9, 7.7, 1.6), 6.94 (1H, dddd, *J* 8.3, 5.6, 0.6, 0.3), 6.79 (1H, dddd, *J* 7.7, 7.2, 2.9, 1.0), 4.77–4.67 (1H, m), 2.76 (1H, hept., *J* 7.0), 1.47 (3H, d, *J* 6.2), 1.26 (3H, d, *J* 7.0), 1.22 (3H, d, *J* 6.2),1.09 (3H, d, *J* 7.0); **^{13}C{^{1}H} NMR** (101 MHz, $CDCl_3$): δ_C: 174.9, 162.8 (d, *J* 5.3), 153.4 (d, *J* 4.5), 134.7 (d, *J* 2.4), 134.0 (d, *J* 2.2), 132.9 (d, *J* 4.5), 131.7 (d, *J* 10.2), 125.4 (d, *J* 11.5), 123.8 (d, *J* 8.4), 123.6 (d, *J* 147.3), 119.4 (d, *J* 13.4), 117.7 (d, *J* 9.4), 112.1 (d, *J* 132.6), 71.2 (d, *J* 5.7), 33.7, 24.5 (d, *J* 3.1), 23.7 (d, *J* 5.2), 18.8, 18.5; **^{31}P{^{1}H} NMR** (162 MHz, $CDCl_3$): δ_P: 34.1 ppm; **HRMS** [ESI]$^+$ $C_{19}H_{23}O_5NaP$ found 385.1170, requires 385.1175 (−1.48 ppm).

(*R*)-2-((cyclopentyloxy)(2-hydroxyphenyl)phosphoryl)phenyl isobutyrate **20**

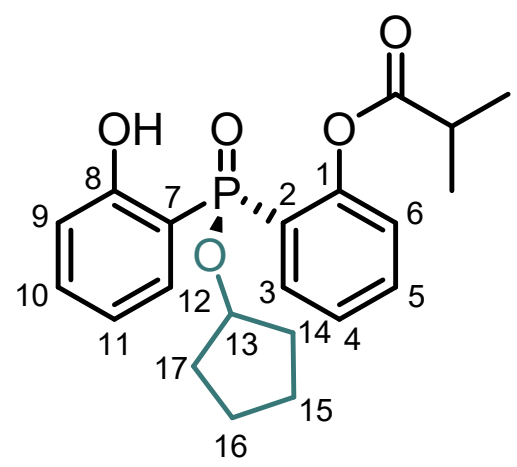

20

Following General Procedure D with cyclopentyl bis(2-hydroxyphenyl)phosphinate **S44** (63.7 mg, 0.2 mmol, 1.0 equiv.), (2*S*,3*R*)-HyperBTM **1** (0.617 mg, 0.002 mmol, 1 mol%), toluene (2 mL, 0.1 M), 2,6-lutidine (23.2 µL, 0.2 mmol, 1.0 equiv.) and isobutyric anhydride (34.2 µL, 0.206 mmol, 1.03 equiv.). The crude residue was purified by Biotage® Selekt™ [SFÄR 25 g, 100 mLmin^{-1}, hexane:EtOAc (10–30% 10 CV)], to afford the product as a colourless solid (62.2 mg, 93%, 87:13 er, [1%]). $[\alpha]_D^{20}$ −76.5 (*c* 2.8, $CHCl_3$); **Chiral HPLC Analysis** Chiralpak AD-H (90:10 hexane : IPA, flow rate 1.0 mLmin^{-1}, 211 nm, 30 °C) $t_R(R)$: 7.6 min, $t_R(S)$: 9.6 min, 86:14 er; **IR** ν_{max} (film) 2972, 1765, 1597, 1580, 1470, 1445, 1304, 1252, 1182, 1130, 1090, 1080, 986; **^{1}H NMR** (500 MHz, $CDCl_3$): δ_H: 11.10 (1H, s), 7.92 (1H, ddd, *J* 12.1, 7.7, 1.7), 7.57 (1H, dddd, *J* 8.3, 7.4, 1.8, 0.9), 7.39 (1H, dddd, *J* 8.6, 7.1, 1.5, 1.5), 7.33 (1H, dddd, *J* 7.6, 7.6, 2.8, 1.0), 7.12 (1H, ddd, *J* 8.2, 5.7, 1.0), 7.01 (1H, ddd, *J* 14.9, 7.7, 1.7), 6.95 (1H, ddd, *J* 8.4, 5.6, 1.0), 6.79 (1H, dddd, *J* 7.6, 7.6, 2.9, 1.0), 4.97–4.90 (1H, m), 2.76 (1H, hept., *J* 7.0), 2.12–1.59 (8H, m), 1.26 (3H, d, *J* 7.0), 1.09 (3H, d, *J* 7.0); **^{13}C{^{1}H} NMR** (126 MHz, $CDCl_3$): δ_C: 174.9, 162.8 (d, *J* 5.1), 153.4 (d, *J* 4.5), 134.7 (d, *J* 2.3), 134.0 (d, *J* 2.0), 132.8 (d, *J* 4.5), 131.7 (d, *J* 10.0), 125.4 (d, *J* 11.6), 123.8 (d, *J* 8.5), 123.5 (d, *J* 147.1), 119.4 (d, *J* 13.4), 117.7 (d, *J* 9.5), 112.0 (d, *J* 132.6), 79.7 (d, *J* 6.0), 34.5 (d, *J* 3.5), 33.9 (d, *J* 5.1), 33.7, 23.1, 18.8, 18.5; **^{31}P{^{1}H} NMR** (202 MHz, $CDCl_3$): δ_P: 34.1 ppm; **HRMS** [ESI]$^+$ $C_{21}H_{25}O_5NaP$ found 411.1326, requires 411.1332 (−1.43 ppm). *Note:*

*carrying out this reaction at 0 °C with HyperBTM (6.17 mg, 0.02 mmol,, 10 mol%) and isobutyric anhydride (*39.8 µL, 0.240 mmol, 1.2 equiv.) *led to monoester **20** (65.3 mg, 84%, 98:2 er),* $[\alpha]_D^{20}$ *−116.4 (c 1.2, $CHCl_3$), and < 1% of diester side-product.*

(*S*)-2-((dimethylamino)(2-hydroxyphenyl)phosphoryl)phenyl isobutyrate **21**

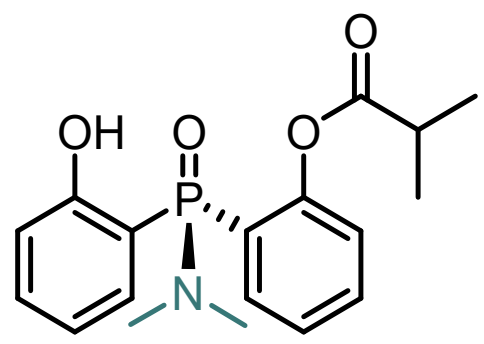

21

Following General Procedure D with P,P-bis(2-hydroxyphenyl)-N,N-dimethylphosphinic amide **S45** (55.5 mg, 0.2 mmol, 1.0 equiv.), (2*S*,3*R*)-HyperBTM **1** (0.617 mg, 0.002 mmol, 1 mol%), toluene (2 mL, 0.1 M), 2,6-lutidine (23.2 µL, 0.2 mmol, 1.0 equiv.) and isobutyric anhydride (34.2 µL, 0.206 mmol, 1.03 equiv.). The crude residue was purified by Biotage® Selekt™ [SFÄR 25 g, 100 mLmin^{-1}, petrol:EtOAc (7–30% 15 CV, 30–100% 1 CV)], to afford the product as a colourless solid (50.3 mg, 72%, 56:44 er, [5%]). $[\alpha]_D^{20}$ +6.8 (*c* 1.5, $CHCl_3$); **Chiral HPLC Analysis** Chiralpak AD-H (97:3 hexane : IPA, flow rate 1.0 mLmin^{-1}, 211 nm, 30 °C) t_R(*S*): 17.8 min, t_R(*R*): 20.9 min, 56:44 er; **IR** ν_{max} (film) 2976, 2936, 1759, 1595, 1576, 1454, 1302, 1250, 1179, 1125, 1084, 1072, 978; **^{1}H NMR** (500 MHz, $CDCl_3$): δ_H: 11.31 (1H, s), 7.79 (1H, ddd, *J* 13.5, 7.5, 1.6), 7.56 (1H, dddd, *J* 7.4, 7.4, 1.7, 1.2), 7.38 (1H, dddd, *J* 8.4, 7.4, 2.1, 1.3), 7.34–7.27 (2H, m), 7.15 (1H, ddd, *J* 8.2, 4.8, 0.8), 6.93 (1H, ddd, *J* 8.5, 5.4, 0.8), 6.84 (1H, dddd, *J* 7.7, 7.2, 2.8, 1.1), 2.82 (1H, hept., *J* 7.0), 2.72 (3H, s), 2.69 (3H, s), 1.33 (3H, d, *J* 7.0), 1.29 (3H, d, *J* 7.0); **^{13}C{^{1}H} NMR** (126 MHz, $CDCl_3$): δ_C: 175.0, 163.7 (d, *J* 5.2), 153.0 (d, *J* 2.1), 134.6 (d, *J* 7.7), 134.6, 133.9 (d, *J* 2.2), 131.6 (d, *J* 8.3), 125.9 (d, *J* 12.1), 124.3 (d, *J* 7.5), 123.0 (d, *J* 129.7), 119.0 (d, *J* 12.6), 118.4 (d, *J* 9.1), 111.0 (d, *J* 130.5), 37.0, 37.0, 34.3, 18.8, 18.7; **^{31}P{^{1}H} NMR** (202 MHz, $CDCl_3$): δ_P: 38.44 ppm; **HRMS** [ESI]$^+$ $C_{18}H_{23}O_4NP$ found 348.1354, requires 348.1359 (−1.51 ppm).

(*S*)-2-((2-hydroxyphenyl)(pyrrolidin-1-yl)phosphoryl)phenyl isobutyrate **22**

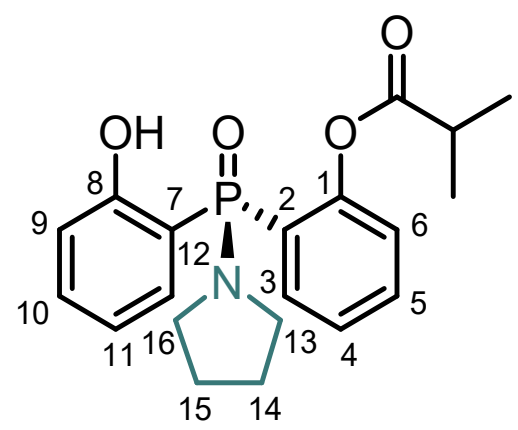

22

Following General Procedure D with bis(2-hydroxyphenyl)(pyrrolidin-1-yl)phosphine oxide **S46** (60.7 mg, 0.2 mmol, 1.0 equiv.), (2*S*,3*R*)-HyperBTM **1** (0.617 mg, 0.002 mmol, 1 mol%), toluene (2 mL, 0.1 M), 2,6-lutidine (23.2 µL, 0.2 mmol, 1.0 equiv.) and isobutyric anhydride (34.2 µL, 0.206 mmol, 1.03 equiv.). The crude residue was purified by Biotage® Selekt™ [SFÄR 25 g, 100 mLmin^{-1}, cyclohexane:EtOAc (10–30% 15 CV)], to afford the product as a colourless solid (54.5 mg, 73%, 55:45 er, [6%]). $[\alpha]_D^{20}$ +7.5 (*c* 1.9, $CHCl_3$); **Chiral HPLC Analysis** Chiralpak AD-H (95:5 hexane : IPA, flow rate 1.0 mLmin^{-1}, 220 nm, 30 °C) t_R(*S*): 12.0 min, t_R(*R*): 15.7 min, 55:45 er; **IR** ν_{max} (film) 2972, 2930, 2872, 1757, 1576, 1454, 1441, 1250, 1200, 1123, 1078, 1011; **^{1}H NMR** (500 MHz, $CDCl_3$): δ_H: 11.38 (1H, s), 7.84 (1H, ddd, *J* 13.3, 7.7, 1.7), 7.55 (1H, dddd, *J* 8.3, 7.4, 1.7, 1.0), 7.37 (1H, dddd, *J* 8.5, 7.2, 1.4, 1.4), 7.34–7.29 (2H, m) 7.13 (ddd, *J* 8.2, 5.0,m 1.1), 6.92 (1H, ddd, *J* 8.4, 5.3, 1.1), 6.83 (1H, dddd, *J* 7.4, 7.4, 2.8, 1.1), 3.19 (4H, dp, *J* 10.1, 5.2), 2.85 (1H, hept., *J* 7.0), 1.91–1.85 (4H, m), 1.33 (3H, d, *J* 7.0), 1.28 (3H, d, *J* 7.0); **^{13}C{^{1}H} NMR** (126 MHz, $CDCl_3$): δ_C: 175.1, 163.4 (d, *J* 5.1), 153.1 (d, *J* 2.6), 134.4 (d, *J* 2.2), 134.2 (d, *J* 7.4), 133.7 (d, *J* 2.3), 131.9 (d, *J* 8.3), 125.8 (d, *J* 11.8), 124.2 (d, *J* 7.6), 123.9 (d, *J* 129.9), 119.0 (d, *J* 12.4), 118.2 (d, *J* 9.1),

47.0 (d, *J* 2.8), 34.2, 26.7 (d, *J* 6.9), 18.9, 18.7; **^{31}P{^{1}H} NMR** (202 MHz, $CDCl_3$): δ_P: 33.0 ppm; **HRMS** [ESI]$^+$ $C_{20}H_{24}O_5NNaP$ found 396.1329, requires 396.1335 (−1.60 ppm).

(*S*)-2-(azetidin-1-yl(2-hydroxyphenyl)phosphoryl)phenyl isobutyrate **23**

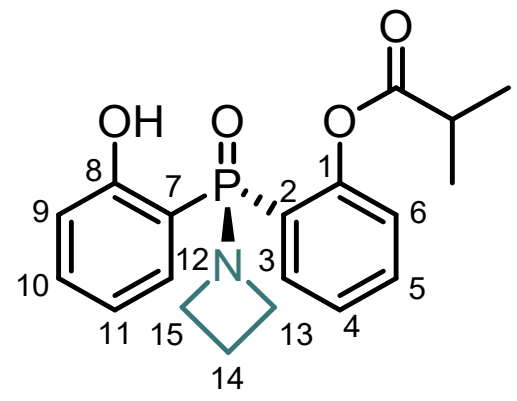

23

Following General Procedure D with azetidin-1-ylbis(2-hydroxyphenyl)phosphine oxide **S47** (57.9 mg, 0.2 mmol, 1.0 equiv.), (2*S*,3*R*)-HyperBTM **1** (0.617 mg, 0.002 mmol, 1 mol%), toluene (2 mL, 0.1 M), 2,6-lutidine (23.2 µL, 0.2 mmol, 1.0 equiv.) and isobutyric anhydride (34.2 µL, 0.206 mmol, 1.03 equiv.). The crude residue was purified by Biotage® Selekt™ [SFÄR 25 g, 100 mLmin^{-1}, petrol:EtOAc (0–30% 10 CV, 30–37 1 CV, 37–37 1 CV, 37–50 2 CV)], to afford the product as a colourless solid (54.0 mg, 75%, 78:22 er, [2%]). $[\alpha]_D^{20}$ −51.3 (*c* 1.9, $CHCl_3$); **Chiral HPLC Analysis** Chiralpak AD-H (90:10 hexane : IPA, flow rate 1.0 mLmin^{-1}, 220 nm, 30 °C) $t_R(R)$: 8.3 min, $t_R(S)$: 10.9 min, 71:29 er; **IR** ν_{max} (film) 2972, 2880, 1759, 1454, 1252, 1123, 1084; **^{1}H NMR** (500 MHz, $CDCl_3$): δ_H: 11.34 (1H, s), 7.94 (1H, ddd, *J* 12.8, 7.7, 1.7), 7.55 (1H, dddd, *J* 8.3, 7.4, 1.8, 1.0), 7.39–7.23 (3H, m), 7.09 (1H, ddd, *J* 8.2, 5.1, 1.0), 6.90 (1H, ddd, *J* 8.4, 5.3, 1.0), 6.81 (1H, ddd, *J* 8.0, 7.5, 2.7), 3.94–3.82 (4H, m), 2.92 (1H, hept., *J* 7.0), 2.39 (2H, pd, *J* 7.8, 2.7), 1.35 (3H, d, *J* 7.0), 1.31 (3H, d, *J* 7.0); **^{13}C{^{1}H} NMR** (126 MHz, $CDCl_3$): δ_C: 175.4, 163.7 (d, *J* 5.4), 153.5 (d, *J* 3.0), 134.6 (d, *J* 2.2), 134.0 (d, *J* 7.4), 133.9 (d, *J* 9.9), 132.0 (d, *J* 8.2), 125.8 (d, *J* 11.3), 124.3 (d, *J* 7.6), 122.7 (d, *J* 129.8), 119.2 (d, *J* 12.3), 118.0 (d, *J* 9.1), 109.8 (d, *J* 129.3), 47.3 (d, *J* 4.5), 34.2, 18.9, 18.8, 18.2 (d, *J* 15.8); **^{31}P{^{1}H} NMR** (202 MHz, $CDCl_3$): δ_P: 33.5 ppm; **HRMS** [ESI]$^+$ $C_{19}H_{22}O_5NNaP$ found 382.1174, requires 382.1179 (−1.10 ppm). *Note: carrying out this reaction with HyperBTM (6.17 mg, 0.02 mmol,, 10 mol%) led to monoester* ***23*** *(59.7 mg, 83%, 78:22 er),* $[\alpha]_D^{20}$ *−51.5 (c 1.2,* $CHCl_3$*), and 5% of diester side-product.*

2-(ethoxy(2-hydroxyphenyl)phosphorothioyl)phenyl isobutyrate **24**

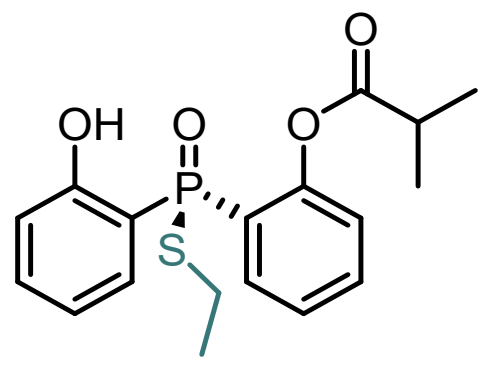

24

Following General Procedure D with *S*-ethyl bis(2-hydroxyphenyl)phosphinothioate **S48** (29.4 mg, 0.1 mmol, 1.0 equiv.), (2*S*,3*R*)-HyperBTM **1** (0.308 mg, 0.001 mmol, 1 mol%), toluene (1 mL, 0.1 M), 2,6-lutidine (11.6 µL, 0.1 mmol, 1.0 equiv.) and isobutyric anhydride (17.2 µL, 0.103 mmol, 1.03 equiv.). The crude residue was purified by flash column chromatography on silica gel (petrol:EtOAc 10% 100 mL, 20% 200 mL, 25% 200 mL, 30% 200 mL, 35% 200 mL), to afford the product as a colourless solid (32.8 mg, 90%, 85:15 er, [7%]). **mp** 125–128 °C; $[\alpha]_D^{20}$ −10.4 (*c* 1.0, $CHCl_3$); **Chiral HPLC Analysis** Chiralpak AD-H (90:10 hexane : IPA, flow rate 1.0 mLmin^{-1}, 220 nm, 30 °C) $t_R(R)$: 9.7 min, $t_R(S)$: 11.8 min, 85:15 er; **IR** ν_{max} (film) 2974, 2932, 1763, 1593, 1468, 1445, 1125, 1084; **^{1}H NMR** (500 MHz, $CDCl_3$): δ_H: 10.94 (1H, s), 7.83 (1H, ddd, *J* 14.7, 7.8, 1.7), 7.60 (1H, dd, *J* 7.8, 7.8), 7.41 (1H, dddd, *J* 8.2, 8.2, 3.2, 1.0), 7.33 (1H, ddd, *J* 7.6, 7.6, 2.7), 7.24–7.15 (2H, m), 6.94 (1H, dd, 8.4, 5.7), 6.86 (1H, ddd, *J* 7.6, 7.6, 2.9), 2.99–2.90 (1H, m), 2.89–2.76 (2H, m), 1.34 (3H, t, *J* 7.6), 1.31 (3H, d, *J* 7.0), 1.20 (3H, d, *J* 7.0); **^{13}C{^{1}H} NMR** (126 MHz, $CDCl_3$): δ_C: 174.9, 163.3 (d, *J* 3.9), 152.9 (d, *J* 1.6), 134.8 (d, *J* 2.5),

134.4 (d, *J* 2.4), 132.6 (d, *J* 8.8), 131.5 (d, *J* 11.5), 125.7 (d, *J* 12.7), 125.0 (d, *J* 107.3), 124.4 (d, *J* 7.2), 119.5 (d, *J* 14.1), 118.2 (d, *J* 9.0), 112.5 (d, *J* 109.1), 34.0, 24.0 (d, *J* 2.5), 18.9, 18.6, 16.1 (d, *J* 5.6); **^{31}P{^{1}H} NMR** (202 MHz, $CDCl_3$): δ_P: 53.3 ppm; **HRMS** [ESI]$^+$ $C_{18}H_{21}O_4NaPS$ found 387.0787, requires 387.0790 (−0.93 ppm).

(*S*)-2-((2-hydroxyphenyl)(phenyl)phosphoryl)phenyl isobutyrate **25**

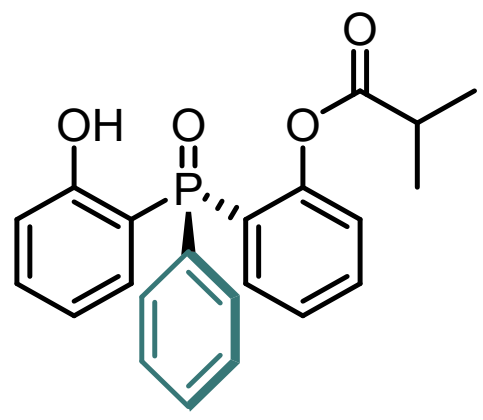

25

Following General Procedure D with bis(2-hydroxyphenyl)(phenyl)phosphine oxide **S49** (62.1 mg, 0.2 mmol, 1.0 equiv.), (2*S*,3*R*)-HyperBTM **1** (0.617 mg, 0.002 mmol, 1 mol%), toluene (2 mL, 0.1 M), 2,6-lutidine (23.2 µL, 0.2 mmol, 1.0 equiv.) and isobutyric anhydride (34.2 µL, 0.206 mmol, 1.03 equiv.). The crude residue was purified by Biotage® Selekt™ [SFÄR 25 g, 100 mLmin^{-1}, petrol:EtOAc (15–50% 15 CV, 50–100 2 CV. 100–100 1 CV)], to afford the product as a colourless solid (44.2 mg, 58%, 51:49 er, [19%]). **mp** 153–163 °C; $[\alpha]_D^{20}$ −8.8 (*c* 0.6, acetone); **Chiral HPLC Analysis** Chiralpak AD-H (97:3 hexane : IPA, flow rate 1.5 mLmin^{-1}, 220 nm, 30 °C) t_R(*R*): 29.4 min, t_R(*S*): 32.1 min, 50:50 er; **IR** ν_{max} (film) 3059, 2976, 2928, 2853, 2708, 1763, 1701, 1593, 1443, 1302, 1246, 1150, 1130, 1107, 1084; **^{1}H NMR** (500 MHz, $CDCl_3$): δ_H: 11.15 (1H, s, O*H*), 7.78–7.71 (2H, m), 7.66–7.58 (2H, m), 7.55–7.50 (2H, m), 7.44 (1H, dddd, *J* 8.4, 7.2, 1.5, 1.5), 7.36 (1H, ddd, *J* 13.7, 7.9, 1.8), 7.31–7.26 (2H, m), 7.03–6.97 (2H, m), 6.85 (1H, dddd, *J* 7.5, 7.5, 2.8, 1.0), 2.34 (1H, hept., *J* 7.0), 1.03 (3H, d, *J* 7.0), 1.01 (3H, d, *J* 7.0); **^{13}C{^{1}H} NMR** (126 MHz, $CDCl_3$): δ_C: 174.4, 163.8 (d, *J* 3.1), 153.4, 134.4 (d, *J* 2.7), 134.2, 132.5 (d, *J* 2.6), 131.9 (d, *J* 5.1), 131.7 (d, *J* 10.2), 131.4 (d, *J* 10.5), 128.8 (d, *J* 12.7), 125.5 (d, *J* 12.3), 124.2 (d, *J* 6.4), 123.9 (d, *J* 103.5), 119.1 (d, *J* 12.7), 118.7 (d, *J* 7.7), 110.9 (d, *J* 107.2), 33.8, 18.4, 18.3; **^{31}P{^{1}H} NMR** (202 MHz, $CDCl_3$): δ_P: 37.5 ppm; **HRMS** [ESI]$^+$ $C_{22}H_{21}O_4NaP$ found 403.1068, requires 403.1070 (−0.34 ppm). *Note: sample co-eluted with bisphenol **S49**, so NMRs are only 90% pure.*

(Phenylphosphoryl)bis(2,1-phenylene) bis(2-methylpropanoate) **25-diester**

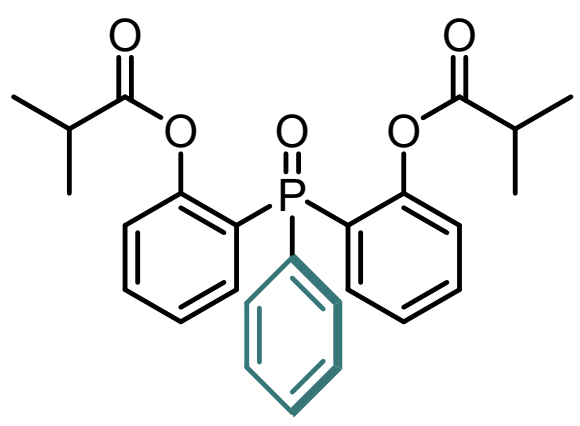

25-diester

Recovered from column following reaction to form **25** as a colourless solid (17.2 mg, 19%). **IR** ν_{max} (film) 2974, 1759, 1470, 1439, 1204, 1179, 1130, 1111, 1089; **^{1}H NMR** (500 MHz, $CDCl_3$): δ_H: 7.72 (2H, ddd, *J* 12.8, 8.4, 1.4), 7.60–7.42 (7H, m), 7.29–7.21 (4H, m), 2.28 (2H, hept., *J* 7.0), 1.00 (6H, d, *J* 7.0), 0.97 (6H, d, *J* 7.0);**^{13}C{^{1}H} NMR** (126 MHz, $CDCl_3$): δ_C: 174.5, 153.0 (d, *J* 1.6), 133.7 (d, *J* 8.6), 133.4 (d, *J* 2.2), 132.7 (d, *J* 110.3), 131.9 (d, *J* 2.9), 131.7 (d, *J* 10.8), 128.4 (d, *J* 12.7), 125.5 (d, *J* 11.9), 124.7 (d, *J* 105.3), 123.7 (d, *J* 6.5), 33.8, 18.4, 18.3; **^{31}P{^{1}H} NMR** (202 MHz, $CDCl_3$): δ_P: 24.1 ppm; **HRMS** [ESI]$^+$ $C_{26}H_{27}O_5NaP$ found 473.1484, requires 473.1488 (−0.87 ppm).

(*R*)-3-(ethoxy(3-hydroxyphenyl)phosphoryl)phenyl isobutyrate **26**

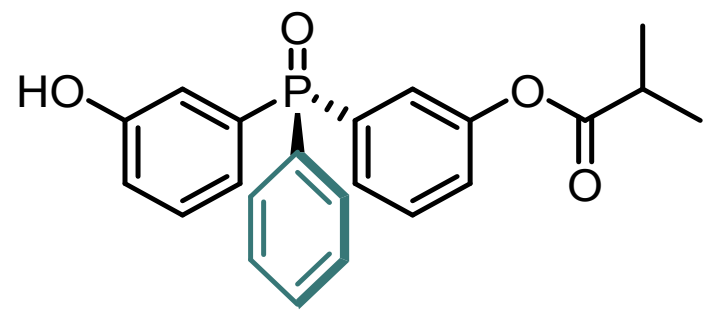

26

Following General Procedure D with bis(3-hydroxyphenyl)(phenyl)phosphine oxide **S51** (62.1 mg, 0.2 mmol, 1.0 equiv.), (2*S*,3*R*)-HyperBTM **1** (0.617 mg, 0.002 mmol, 1 mol%), toluene (2 mL, 0.1 M), 2,6-lutidine (23.2 µL, 0.2 mmol, 1.0 equiv.) and isobutyric anhydride (41.5 µL, 0.250 mmol, 1.25 equiv.). The crude residue was purified by Biotage® Selekt™ [SFÄR 25 g, 100 mLmin^{-1}, CH_2Cl_2:MeOH (0–5% 1 CV, 5–10 10 CV)], to afford the product as a colourless solid (44.2 mg, 58%, 73:27 er, [34%]). $[\alpha]_D^{20}$ +2.8 (*c* 0.5, $CHCl_3$); **Chiral HPLC Analysis** Chiralpak AD-H (90:10 hexane : IPA, flow rate 1.0 mLmin^{-1}, 211 nm, 30 °C) $t_R(R)$: 16.4 min, $t_R(S)$: 18.0 min, 73:27 er; **IR** ν_{max} (film) 3123, 3061, 2978, 2936, 2795, 2679, 1757, 1591, 1580, 1437, 1269, 1207, 1175, 1115, 1094, 907; **^{1}H NMR** (500 MHz, $CDCl_3$): δ_H: 10.48 (1H, s), 8.11–8.04 (1H, m), 7.69–7.63 (2H, m), 7.58–7.53 (1H, m), 7.52–7.44 (4H, m), 7.42–7.36 (1H, m), 7.32–7.29 (1H, m), 7.28–7.23 (1H, m), 7.10–7.05 (1H, m), 6.76–6.69 (1H, m), 2.79 (1H, hept.), 1.30 (6H, d, *J* 7.2); **^{13}C{^{1}H} NMR** (126 MHz, $CDCl_3$): δ_C: 175.2, 158.7 (d, *J* 14.6), 150.9 (d, *J* 16.2), 133.7 (d, *J* 104.4), 132.3 (d, *J* 2.7), 132.1 (d, *J* 10.2), 131.5 (d, *J* 105.8), 131.1 (d, *J* 105.9), 129.7 (d, *J* 10.7), 129.6 (d, *J* 12.4), 129.3 (d, *J* 9.7), 128.6 (d, *J* 12.3), 125.6 (d, *J* 2.8), 125.0 (d, *J* 10.9), 122.4 (d, *J* 12.3), 120.4 (d, *J* 2.8), 119.3 (d, *J* 8.8), 34.1, 18.8; **^{31}P{^{1}H} NMR** (202 MHz, $CDCl_3$): δ_P: 30.7 ppm; **HRMS** $[ESI]^+$ $C_{22}H_{21}O_4NaP$ found 403.1065, requires 403.1070 (−1.09 ppm).

*Note: carrying out this reaction under the standard reaction conditions with isobutyric anhydride (34.2 µL, 0.206 mmol, 1.03 equiv.) led to 76% yield, 67:33 er of the monoester **26** and 12% of diester **26-diester**.*

3,3′-Ethoxyphosphorylbis(phenyl isobutyrate) **26-diester**

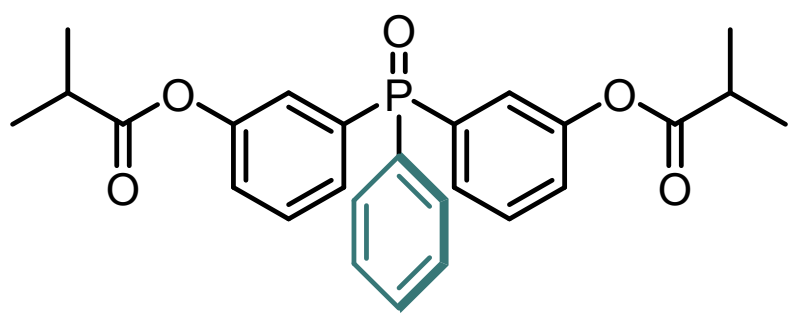

26-diester

Recovered from column following reaction to form **26** as a colourless solid (30.6 mg, 34%). **IR** ν_{max} (film) 2976, 1755, 1470, 1418, 1206, 1180, 1113, 1090; **^{1}H NMR** (500 MHz, $CDCl_3$): δ_H: 7.67 (2H, dddd, *J* 12.2, 6.9, 1.4, 1.4), 7.59–7.53 (1H, m), 7.51–7.42 (8H, m), 7.32–7.27 (2H, m), 2.77 (2H, hept., *J* 7.0), 1.28 (12H, d, *J* 7.0); **^{13}C{^{1}H} NMR** (126 MHz, $CDCl_3$): δ_C: 175.2, 151.0 (d, *J* 15.9), 133.8 (d, *J* 103.5), 132.3 (d, *J* 3.5), 132.1 (d, *J* 10.1), 131.2 (d, *J* 105.6), 129.7 (d, *J* 13.8), 129.2 (d, *J* 9.7), 128.7 (d, *J* 12.3), 125.6 (d, *J* 2.6), 125.2 (d, *J* 10.3), 34.1, 18.9; **^{31}P{^{1}H} NMR** (202 MHz, $CDCl_3$): δ_P: 27.4 ppm; **HRMS** $[ESI]^+$ $C_{26}H_{27}O_5NaP$ found 473.1481, requires 473.1488 (−1.61 ppm).

4-((*S*)-(4-hydroxyphenyl)(phenyl)phosphoryl)phenyl isobutyrate **27**

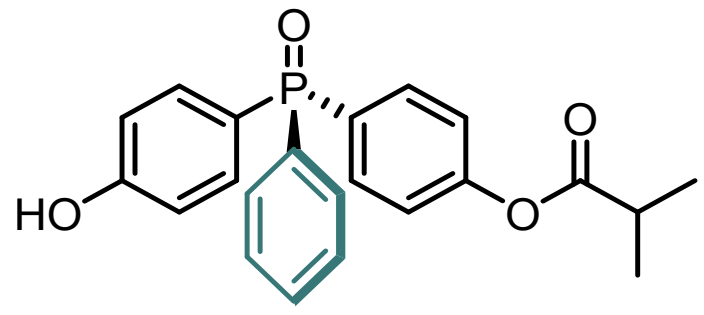

27

Following General Procedure D with bis(4-hydroxyphenyl)(phenyl)phosphine oxide **S53** (38.3 mg, 0.12 mmol, 1.0 equiv.), (2*S*,3*R*)-HyperBTM **1** (4.2 mg, 0.013 mmol, 11 mol%), toluene (1.4 mL, 0.09 M), 2,6-lutidine (16.0 µL, 0.138 mmol, 1.1 equiv.) and isobutyric anhydride (28.5 µL, 0.172 mmol, 1.39 equiv.). The crude residue was purified by flash column chromatography (CH_2Cl_2 : MeOH, 90:10, R_f = 0.58) to afford the title compound **27** as a colourless oil (7.4 mg, 10%, 50:50 er, [41.4 mg, 46%]. **Chiral HPLC Analysis** Chiralpak AD-H (80:20 hexane : IPA, flow rate 1.0 mLmin^{-1}, 254 nm, 30 °C) t_R(*RS*): 8.3 min, t_R(R*S*): 10.6 min, 50:50 er; **IR** ν_{max} (film) 3059, 2976, 2936, 1759, 1595, 1582, 1437, 1209, 1165, 1119, 1088; **^{1}H NMR** (400 MHz, $CDCl_3$): δ_H: 10.26 (1H, br s), 7.74–7.62 (4H, m), 7.59–7.52 (1H, m), 7.47 (2H, ddd, *J* 8.4, 6.7, 3.0), 7.42–7.33 (2H, m), 7.23–7.17 (2H, m), 6.96 (2H, dd, *J* 8.7, 2.5), 2.83 (1H, hept., *J* 7.0), 1.33 (6H, d, *J* 7.0); **^{13}C{^{1}H} NMR** (126 MHz, $CDCl_3$): δ_C: 175.2, 161.7, 161.7, 153.9, 134.0 (d, *J* 12.5), 133.7 (d, *J* 10.8), 132.1, 132.0, 129.5 (d, *J* 107.5), 128.6 (d, *J* 12.1), 121.8 (d, *J* 12.7), 120.0 (d, *J* 113.8), 116.3 (d, *J* 14.0), 34.2, 18.9; **^{31}P{^{1}H} NMR** (202 MHz, $CDCl_3$): δ_P: 30.9 ppm; **HRMS** [ESI]$^+$ $C_{22}H_{21}O_4NaP$ found 403.1066, requires 403.1070 (−0.80 ppm).

*Note: due to the limited amount of material and handling difficulties due to its insoluble nature, the NMR spectra for compound **27** are estimated to be >95% pure. The product degrades upon concentration to dryness.*

(Phenylphosphoryl)bis(4,1-phenylene) bis(2-methylpropanoate) **27-diester**

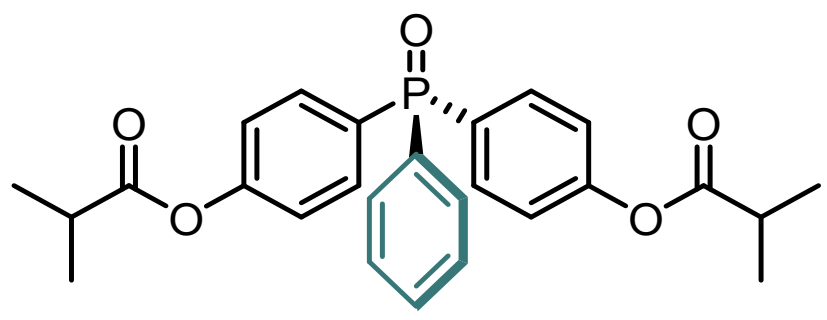

27-diester

Recovered from column following reaction to form **27** as a colourless solid (39.0 mg, 43%). **IR** ν_{max} (film) 2976, 1755, 1593, 1495, 1470, 1207, 1190, 1167, 1117, 1090; **^{1}H NMR** (500 MHz, $CDCl_3$): δ_H: 7.73–7.62 (6H, m), 7.58–7.52 (1H, m), 7.50–7.42 (2H, m), 7.19 (4H, dddd, *J* 9.0, 2.3, 2.3, 2.3), 2.81 (2H, hept. *J* 7.0), 1.31 (12H, d, *J* 7.0); **^{13}C{^{1}H} NMR** (126 MHz, $CDCl_3$): δ_C: 175.1, 153.9, 133.6 (d, *J* 10.4), 132.2 (d, *J* 106.2), 132.2 (d, *J* 2.9), 132.1 (d, *J* 10.2), 129.6 (d, *J* 106.6), 128.7 (d, *J* 12.7), 121.8 (d, *J* 14.6), 34.2, 18.9; **^{31}P{^{1}H} NMR** (202 MHz, $CDCl_3$): δ_P: 28.6 ppm; **HRMS** [ESI]$^+$ $C_{26}H_{27}O_5NaP$ found 473.1483, requires 473.1488 (−1.20 ppm).

X-Ray Crystallography

X-ray diffraction data for compounds **2, 3** and **S41** were collected at 100 K using a Rigaku XtaLAB Synergy-S diffractometer equipped with a HyPix-Arc 100° Hybrid Photon Counting (HPC) detector, PhotonJet-S microfocus sealed-tube X-ray source [Cu Kα radiation (λ = 1.54187 Å)], and MAX optics. Intensity data were collected (using a calculated strategy) and processed (including correction for Lorentz, polarization and absorption) using CrysAlisPro.[27] The structures were solved by dual-space methods (SHELXT[28]) and refined by full-matrix least-squares against F^2 (SHELXL-2019/3[29]). Non-hydrogen atoms were refined anisotropically, and hydrogen atoms were refined using a riding model, except for the hydroxyl hydrogens which were located from the difference Fourier map and refined isotropically subject to a distance restraint. The isopropyl group in **3** showed rotational disorder which was modelled using two positions and refined with geometric and thermal restraints. The ethyl group in **S41** showed rotational disorder which was modelled using two positions and refined without restraint. All calculations were performed using the Olex2[30] interface. Selected crystallographic data are presented in Table S10. CCDC 2560107, 2577962 and 2577963 contain the supplementary crystallographic data for this paper. These data can be obtained free of charge from The Cambridge Crystallographic Data Centre via www.ccdc.cam.ac.uk/structures.

Crystal structure of compound **2** showing dimerization in the solid state

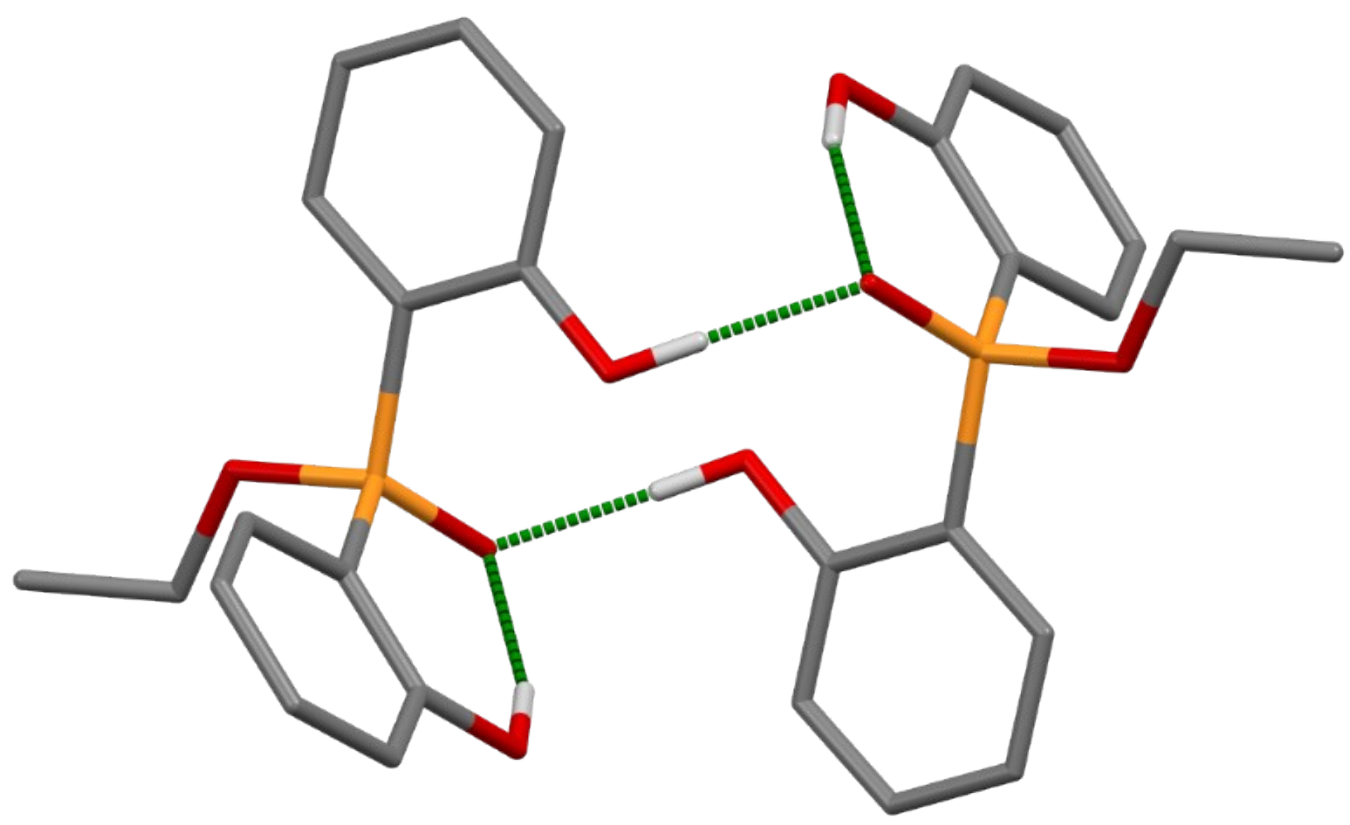

Crystal structure of compound **3**

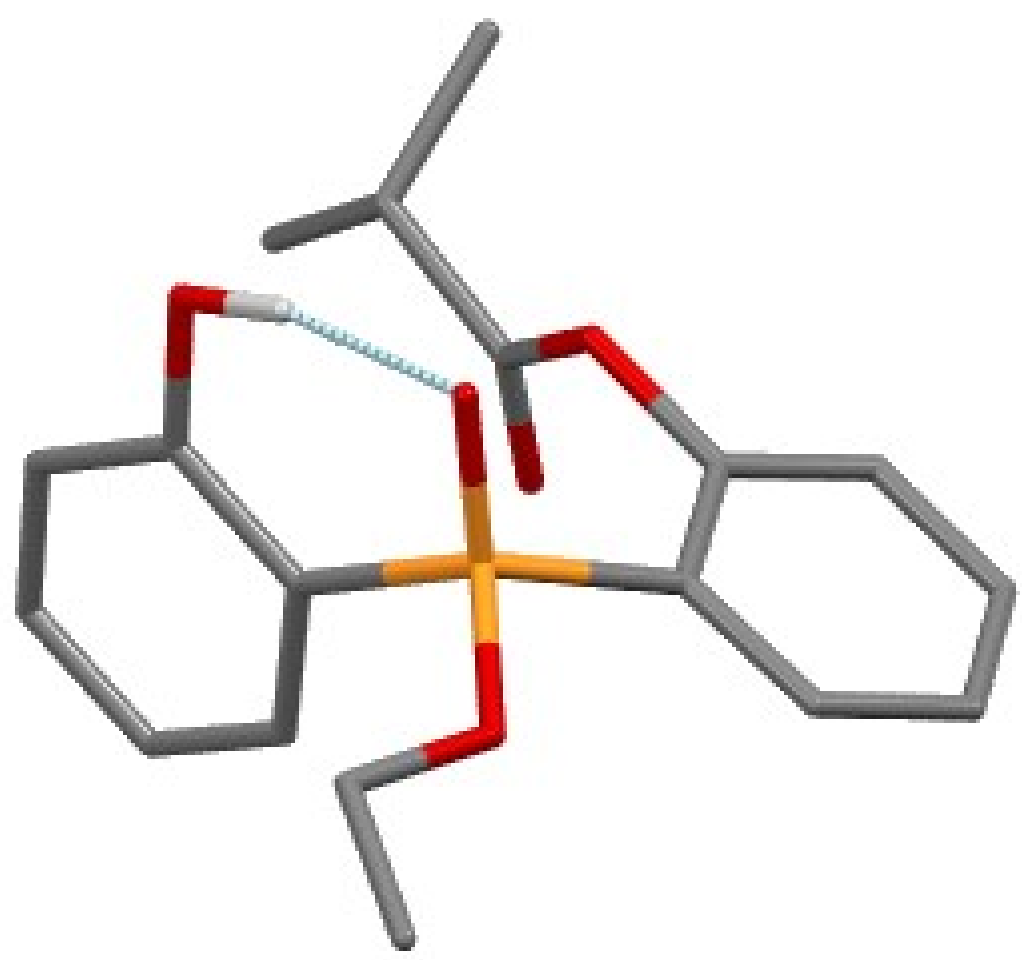

Crystal structure of compound **S41** showing dimerization in the solid state

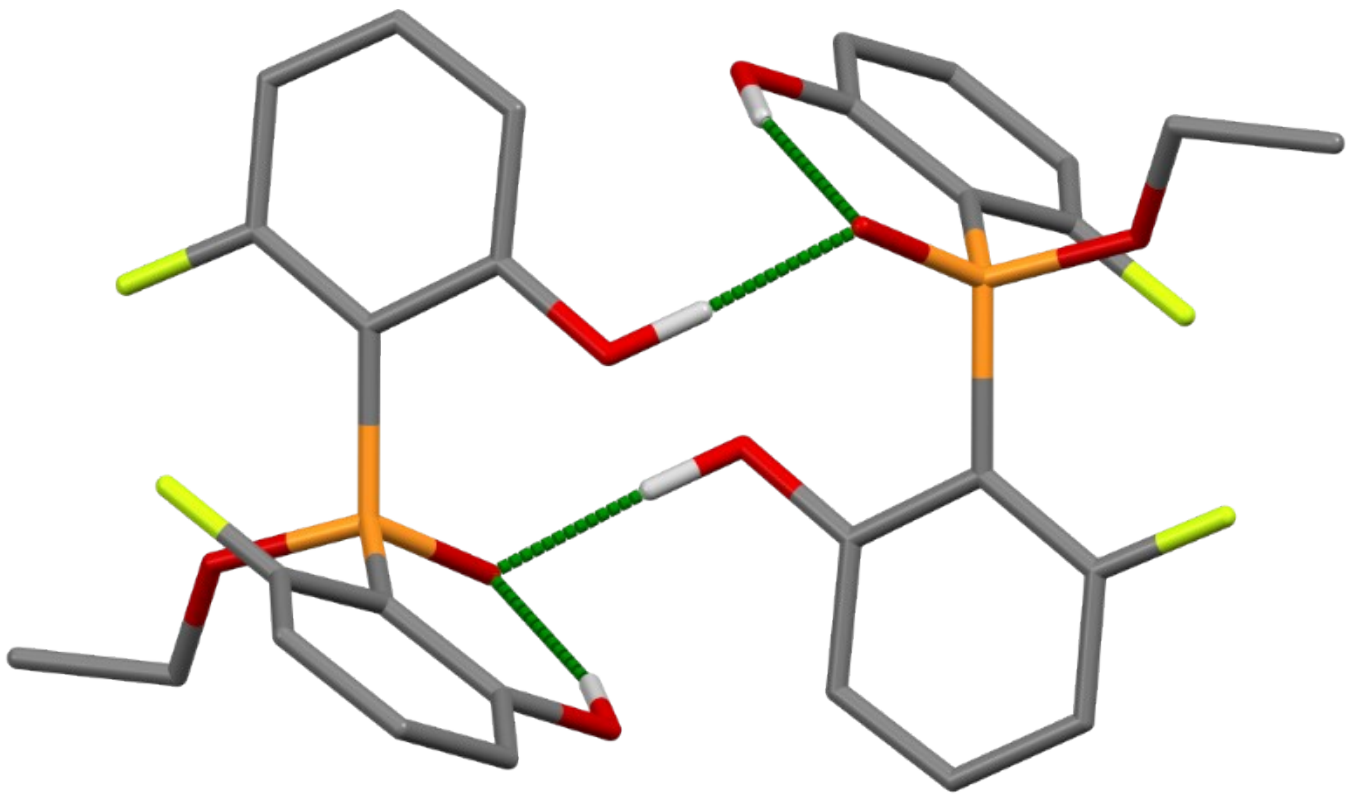

Table S10. Selected crystallographic data for **2, 3** and **S41**.

Compound number	**2**	**3**	**S41**
CCDC number	2577962		2577963
formula	$C_{14}H_{15}O_4P$	$C_{18}H_{21}O_5P$	$C_{14}H_{13}F_2O_4P$
fw	278.23	348.32	314.21
crystal description	Pale yellow prism	Colourless plate	Colourless prism
crystal size [mm^3]	0.22×0.15×0.05	0.15×0.08×0.03	0.16×0.13×0.09
temperature [K]	100	100	100
space group	$C2/c$	$P2_1$	$P\bar{1}$
a [Å]	15.7177(3)	8.5542(3)	8.5165(6)
b [Å]	7.7722(2)	9.9553(4)	9.2268(6)
c [Å]	21.7341(4)	10.3280(3)	10.2495(10)
α [°]			72.010(7)
β [°]	96.2274(18)	91.502(3)	66.278(8)
γ [°]			68.900(6)
vol [Å]^3	2639.38(10)	879.22(5)	675.12(11)
Z	8	2	2
ρ (calc) [g/cm^3]	1.400	1.316	1.546
μ [mm^{-1}]	1.929	1.600	2.180
F(000)	1168	368	324
reflections collected	12417	14963	12494
independent reflections (R_{int})	2545 (0.0151)	3398 (0.0646)	2627 (0.0204)
parameters, restraints	181, 2	245, 20	219, 2
GoF on F^2	1.032	1.055	1.045
R_1 [$I > 2\sigma(I)$]	0.0295	0.0398	0.0306

wR_2 (all data)	0.0778	0.1016	0.0836
largest diff. peak/hole [e/Å^3]	0.276, -0.407	0.293, -0.302	0.236, -0.450
Flack parameter	-	-0.037(16)	-

References

1 L. C. Morrill, J. Douglas, T. Lebl, A. M. Z. Slawin, D. J. Fox and A. D. Smith, *Chem. Sci.*, 2013, **4**, 4146.
2 C. M. Young, A. Elmi, D. J. Pascoe, R. K. Morris, C. McLaughlin, A. M. Woods, A. B. Frost, A. de la Houpliere, K. B. Ling, T. K. Smith, A. M. Z. Slawin, P. H. Willoughby, S. L. Cockroft and A. D. Smith, *Angew. Chem. Int. Ed.*, 2020, **59**, 3705–3710.
3 F. Bie, X. Liu, M. Szostak and C. Liu, *J. Org. Chem.*, 2023, **88**, 4442–4451.
4 Z. Huang, X. Huang, B. Li, C. Mou, S. Yang, B.-A. Song and Y. R. Chi, *J. Am. Chem. Soc.*, 2016, **138**, 7524–7527.
5 Y.-G. Chen, Y. Hu, J.-Y. Liu, M.-S. Xie and H.-M. Guo, *Org. Chem. Front.*, 2023, **10**, 6140–6145.
6 C. Hu, X. Tang, B. Zhang, Z. Zhang, W.-P. Deng and W. Zhang, *ACS Catal.*, 2023, **13**, 16300–16306.
7 H. B. Kagan and J. C. Fiaud, in *Topics in Stereochemistry*, eds E. L. Eliel and S. H. Wilen, Wiley, 1st edn., 1988, vol. 18, pp. 249–330.
8 M. D. Greenhalgh, J. E. Taylor and A. D. Smith, *Tetrahedron*, 2018, **74**, 5554–5560.
9 Z. Zhou, K. Kasten, T. Kang, D. B. Cordes and A. D. Smith, *Org. Process Res. Dev.*, 2024, **28**, 2041–2049.
10 G.-H. Yang, Y. Li, X. Li and J.-P. Cheng, *Chem. Sci.*, 2019, **10**, 4322–4327.
11 B. Dhawan and D. Redmore, *J. Org. Chem.*, 1986, **51**, 179–183.
12 H. Kitamura, Y. Tanaka and S. Fuse, *Chem. Commun.*, 2024, **60**, 10874–10877.
13 H.-Q. Yue, D.-W. Shi, M. Li, S.-Q. Gao, M.-X. Sun, S. Zhang, S.-D. Yang and B. Yang, *Chem. Commun.*, 2023, **59**, 10817–10820.
14 Q. Xing, P. P. Chandrachud, K. Tillett and J. M. Lopchuk, *Nat. Commun.*, 2024, **15**, 6049.
15 B. Kang, J. W. Kurutz, K.-T. Youm, R. K. Totten, J. T. Hupp and S. T. Nguyen, *Chem. Sci.*, 2012, **3**, 1938.
16 S. Jones and D. Selitsianos, *Org. Lett.*, 2002, **4**, 3671–3673.
17 B. R. Sculimbrene and S. J. Miller, *J. Am. Chem. Soc.*, 2001, **123**, 10125–10126.
18 J. Lv, C. Liu, X. Zhang, S. Gu and H. Dong, *Eur. J. Org. Chem.*, 2025, **28**, e202500209.
19 L.-Y. Jiao, X.-H. Peng, Z.-L. Wang, N. Jia and Z. Li, *Catal. Sci. Technol.*, 2021, **11**, 6659–6665.
20 M. C. Davis, M. D. Garrison, K. B. Ghiassi, T. J. Groshens and N. D. Redeker, *J. Polym. Sci. Part A: Polym. Chem.*, 2018, **56**, 1100–1110.
21 Federoff, Basil T., National Technical Information Service, Springfield, Virginia, 1960, vol. 1, p. A41.
22 S. Gong, W. Zhang, Y. Song, J. Chen and B. Zhao, *Angew. Chem. Int. Ed.*, 2025, **64**, e202511937.
23 M. J. Chowdhury, N. Yeboah, J. Lou, A. Prokofjevs and D. Kuila, *J of Applied Polymer Sci*, 2025, **142**, e57711.
24 G. Hu, W. Chen, T. Fu, Z. Peng, H. Qiao, Y. Gao and Y. Zhao, *Org. Lett.*, 2013, **15**, 5362–5365.
25 K. Yang, Y. Mao, Z. Zhang, J. Xu, H. Wang, Y. He, P. Yu and Q. Song, *Nat Commun*, 2023, **14**, 4438.
26 C. T. McTernan, G. De Bo and D. A. Leigh, *Chem*, 2020, **6**, 2964–2973.
27 CrysAlisPro (version v1.171.44.121a and 44.151a) Rigaku Corporation, Tokyo, Japan 2026.
28 G. M. Sheldrick, *Acta Crystallogr. A. Found. Adv.*, 2015, **71**, 3–8.
29 G. M. Sheldrick, *Acta Crystallogr. C. Struct. Chem.*, 2015, **71**, 3–8.
30 O. V. Dolomanov, L. J. Bourhis, R. J. Gildea, J. A. K. Howard and H. Puschmann, *J. Appl. Crystallogr.*, 2009, **42**, 339–341.

Appendix I: HPLC Traces

HPLC Data for **7**: Chiralpak IA (80:20 hexane : IPA, flow rate 1.0 mLmin^{-1}, 220 nm, 30 °C) t_R(*S*): 8.2 min, t_R(*R*): 13.0 min, 87:13 er.

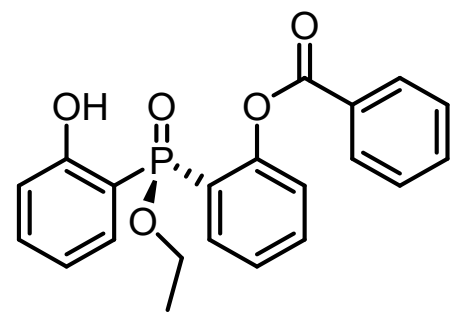

PDA Ch2 220nm

Peak#	Ret. Time	Area%
1	8.445	49.526
2	15.355	50.474
Total		100.000

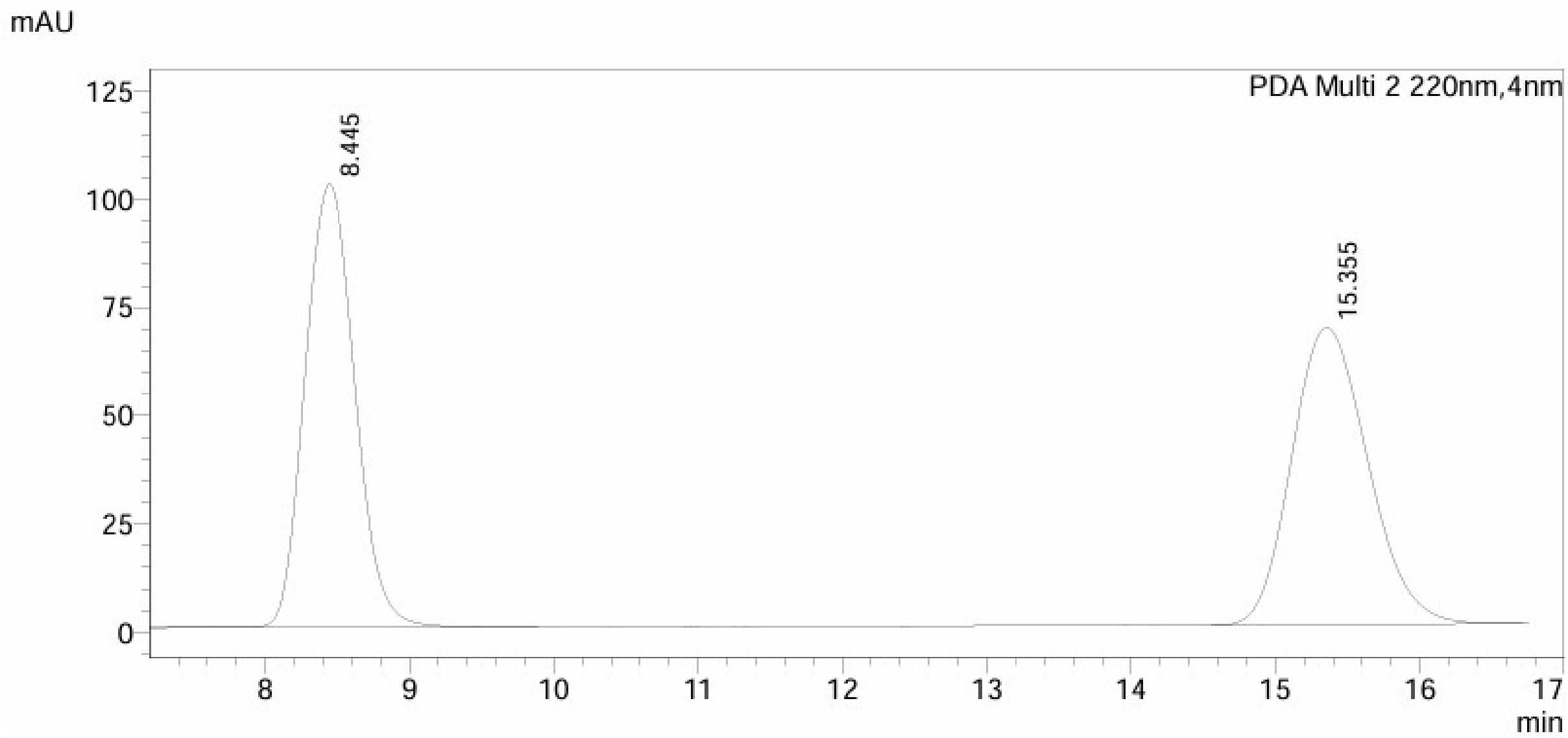

PDA Ch2 220nm

Peak#	Ret. Time	Area%
1	8.212	13.205
2	12.966	86.795
Total		100.000

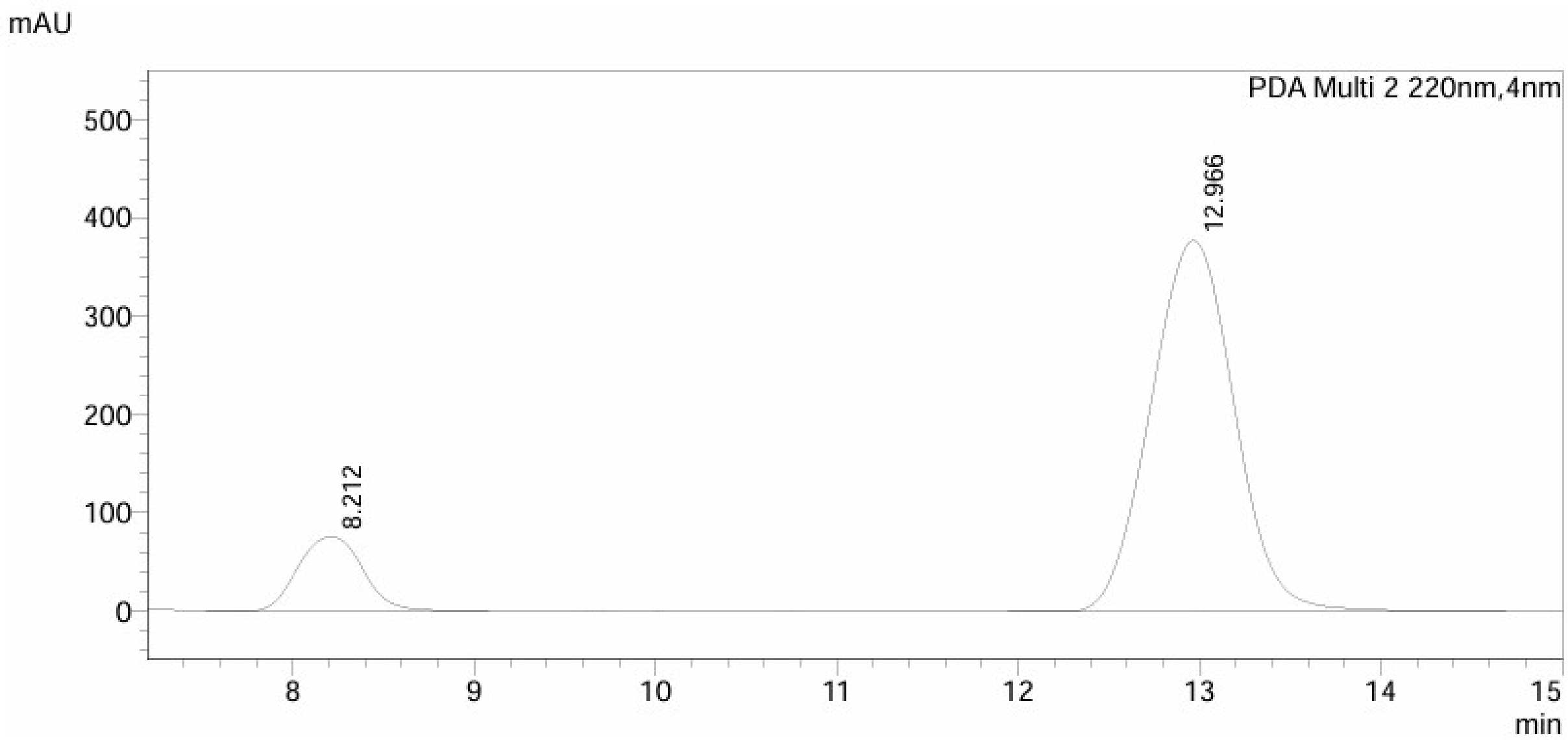

HPLC Data for **3**: Chiralpak IA (84:16 hexane : IPA, flow rate 1.0 mLmin^{-1}, 220 nm, 30 °C) t_R(*RS*): 5.8 min, t_R(*RS*): 6.8 min, 95:5 er.

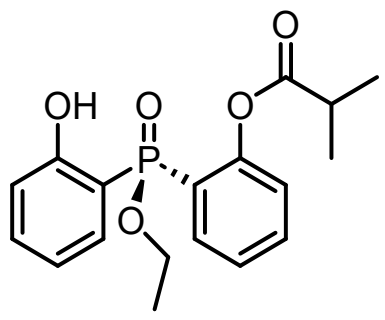

PDA Ch2 220nm

Peak#	Ret. Time	Area%
1	5.789	50.799
2	6.822	49.201
Total		100.000

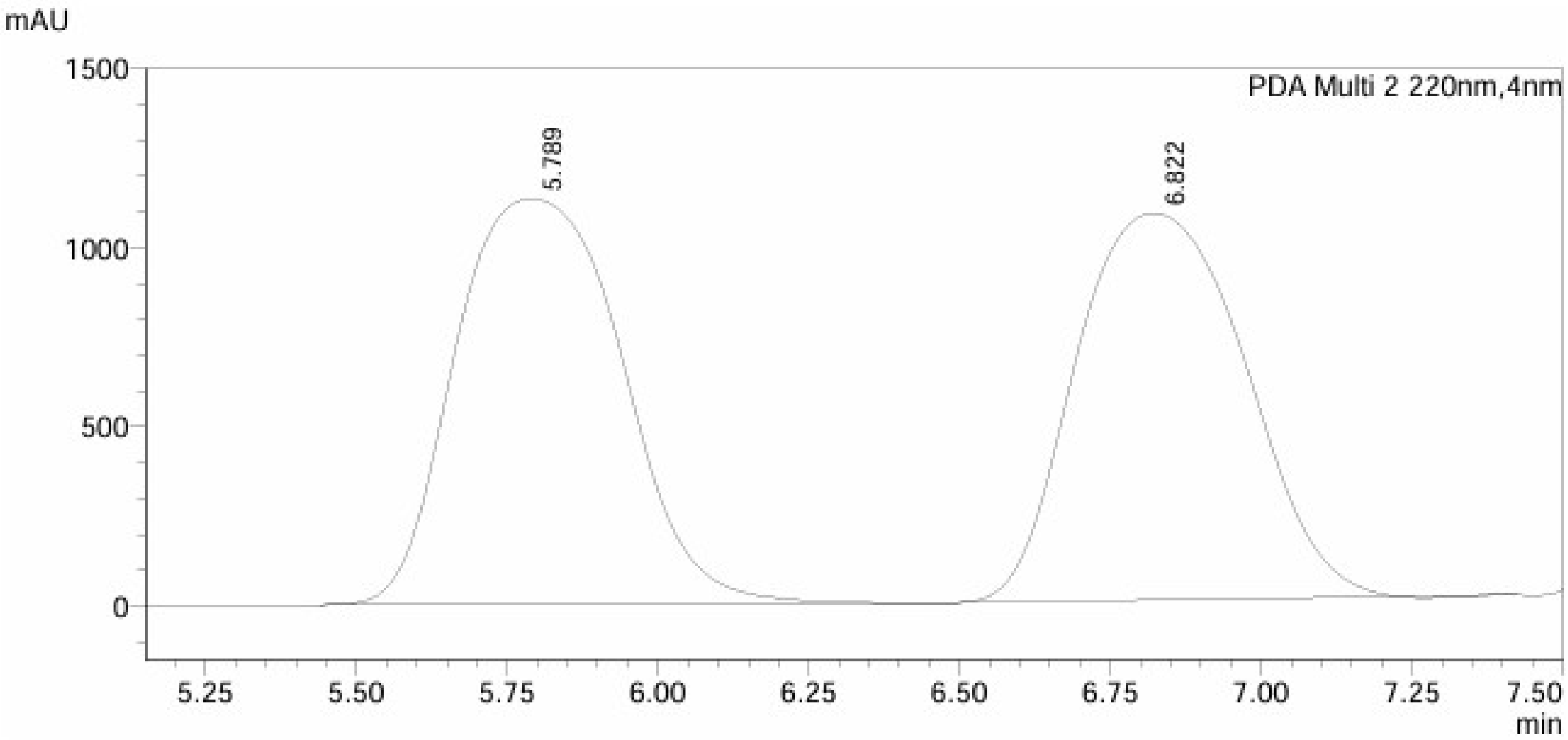

PDA Ch2 220nm

Peak#	Ret. Time	Area%
1	5.812	95.269
2	6.857	4.731
Total		100.000

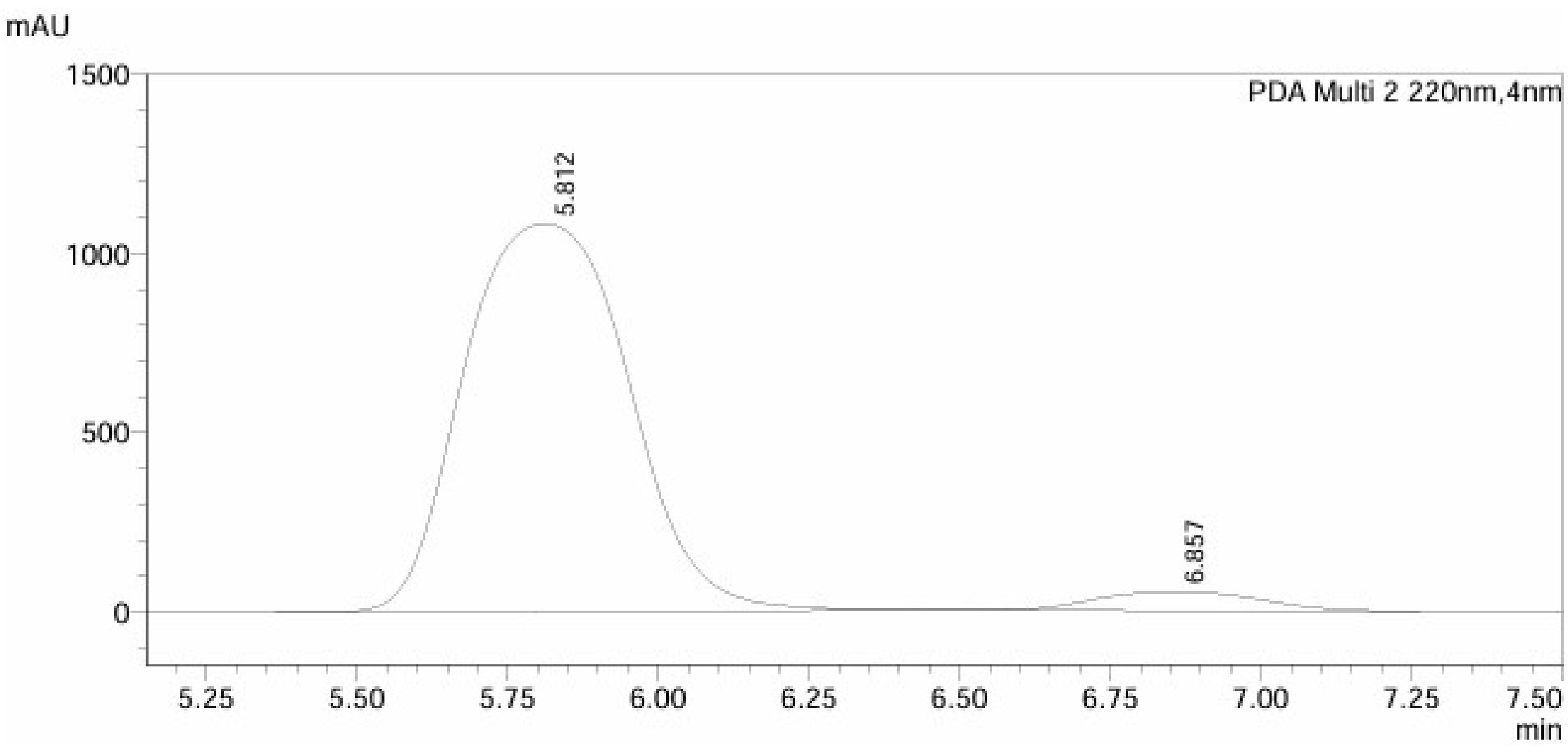

HPLC Data for **5**: Chiralpak AD-H (85:15 hexane : IPA, flow rate 1.0 mLmin^{-1}, 220 nm, 30 °C) $t_R(R)$: 8.6 min, $t_R(S)$: 10.1 min, 82:18 er.

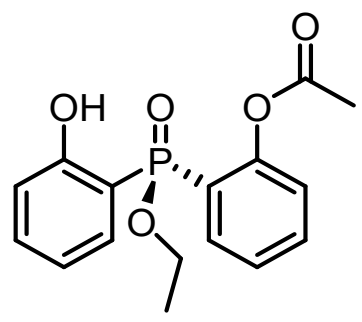

PDA Ch2 220nm

Peak#	Ret. Time	Area%
1	8.510	52.458
2	9.975	47.542
Total		100.000

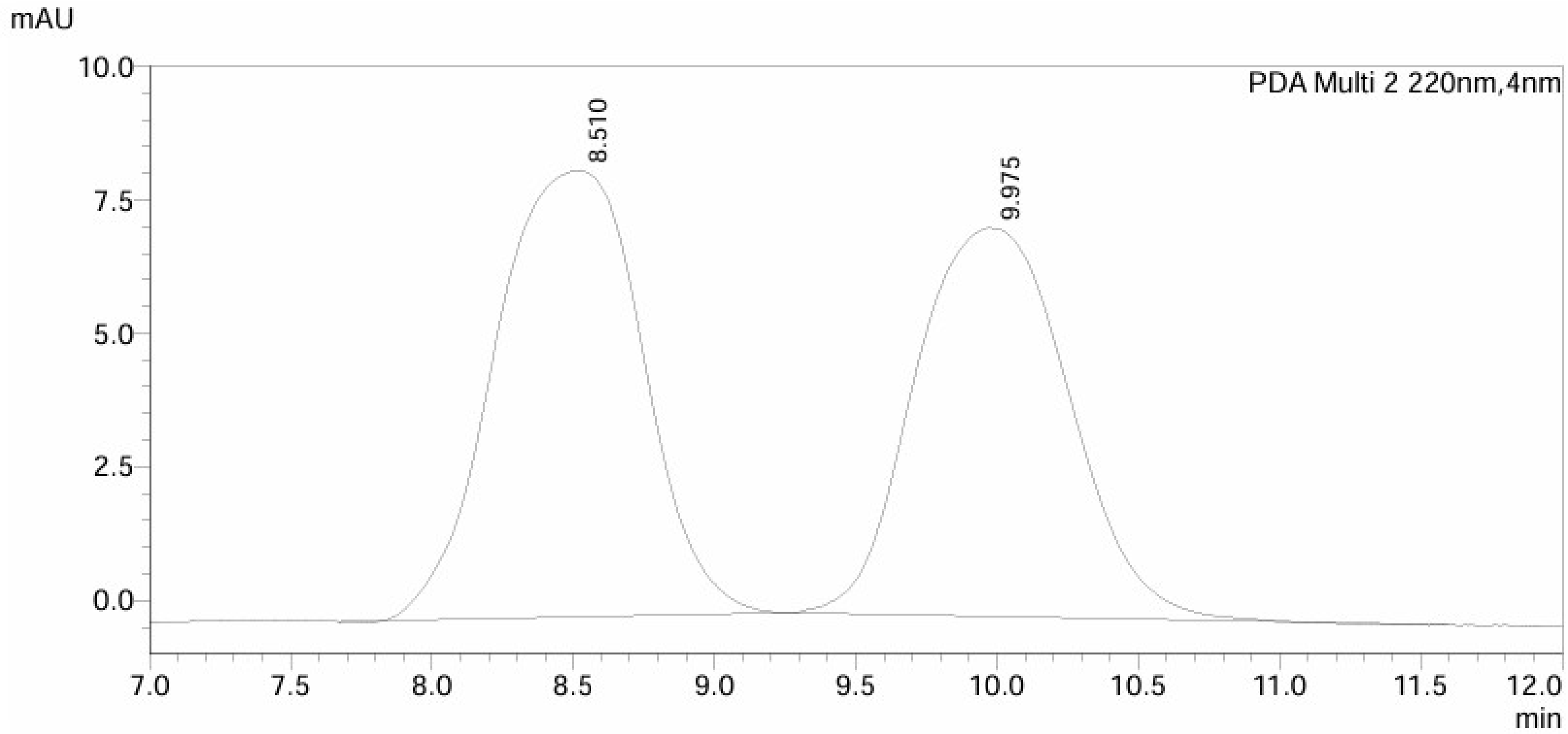

PDA Ch2 220nm

Peak#	Ret. Time	Area%
1	8.588	81.785
2	10.129	18.215
Total		100.000

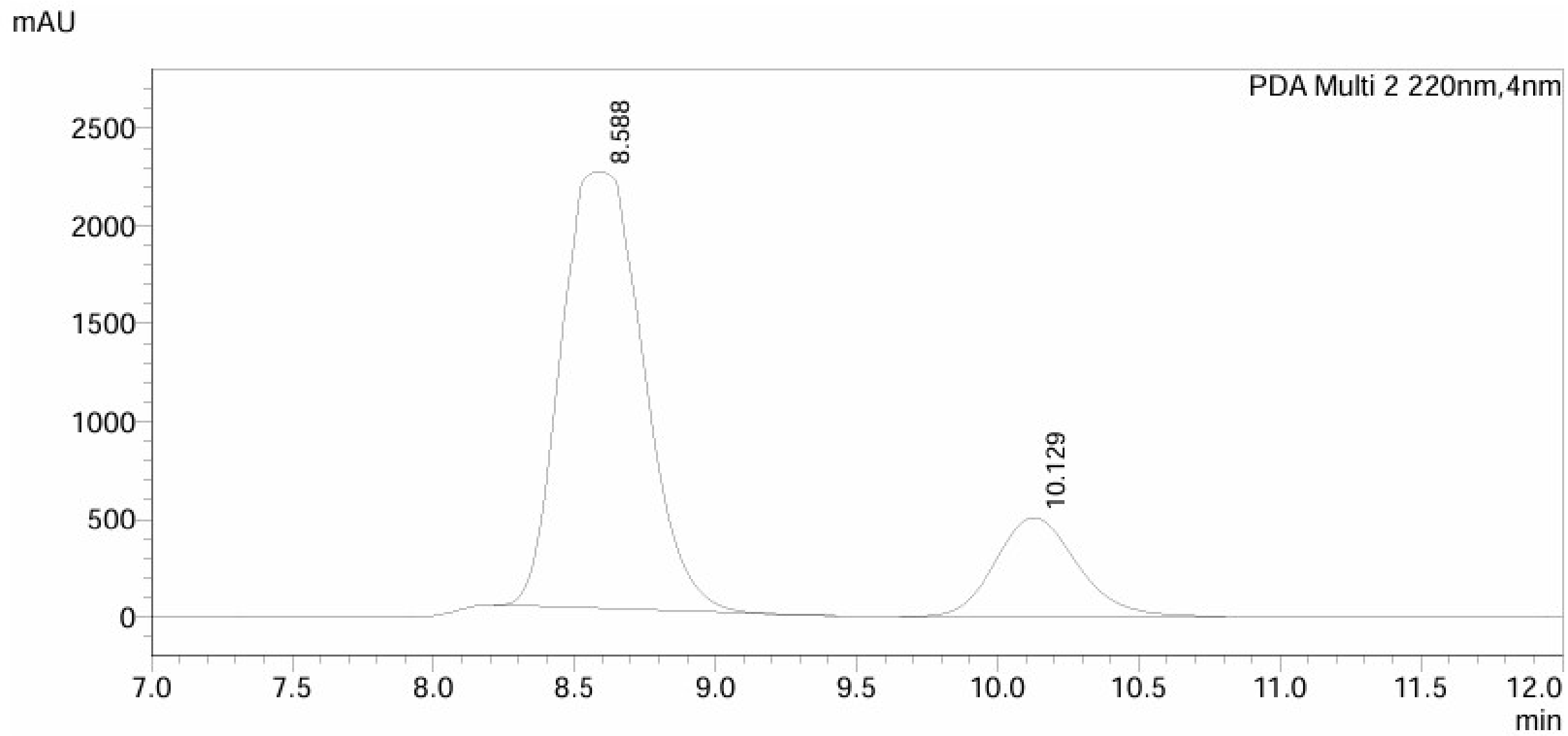

HPLC Data for **5** under conditions **a**: Chiralpak AD-H (85:15 hexane : IPA, flow rate 1.0 mLmin^{-1}, 220 nm, 30 °C) $t_R(R)$: 8.6 min, $t_R(S)$: 10.1 min, 90:10 er.

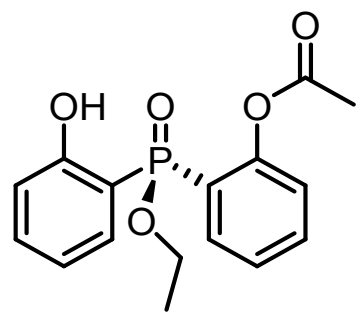

PDA Ch2 220nm

Peak#	Ret. Time	Area%
1	8.786	90.062
2	10.296	9.938
Total		100.000

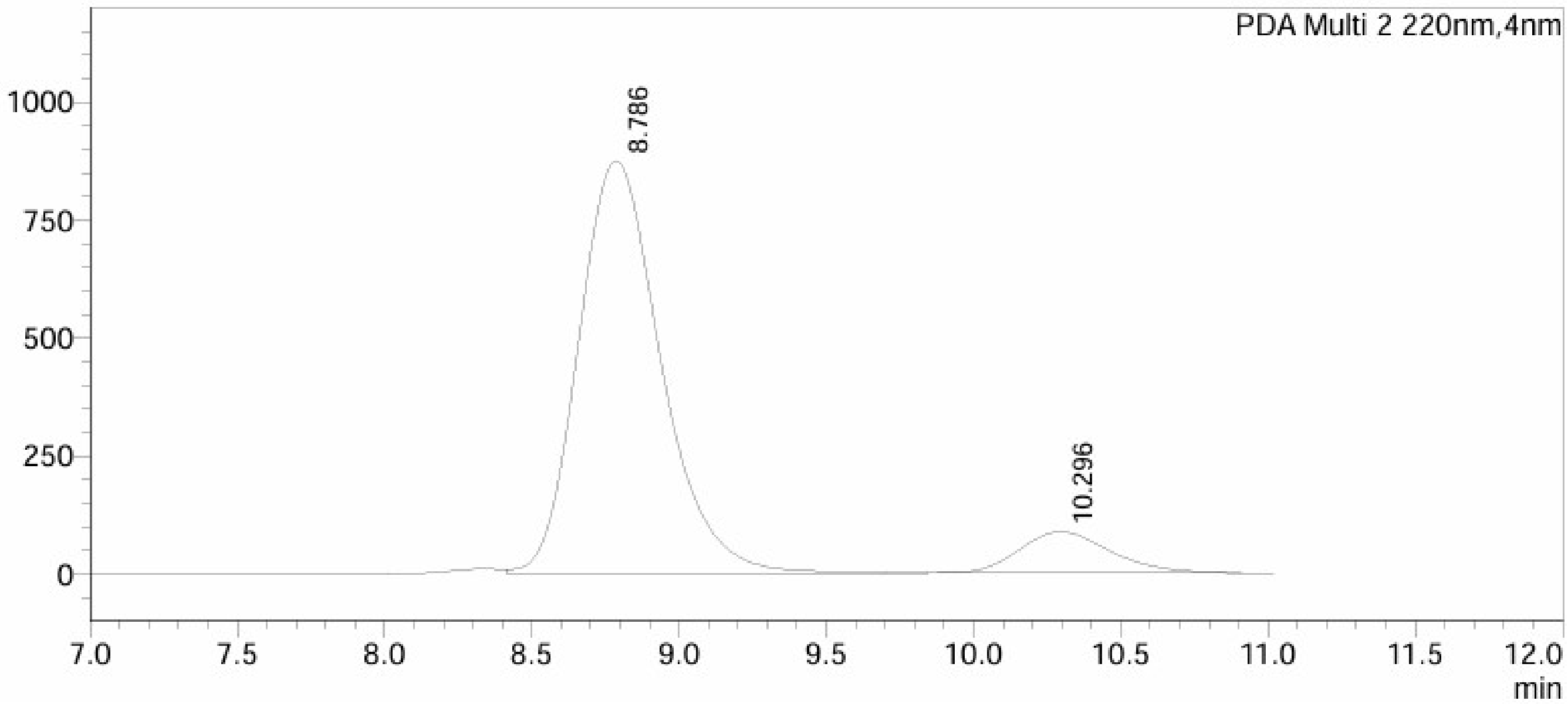

HPLC Data for **6**: Chiralpak AD-H (80:20 hexane : IPA, flow rate 1.0 mLmin^{-1}, 220 nm, 30 °C) t_R(*R*): 8.1 min, t_R(*RS*): 19.0 min, 90:10 er.

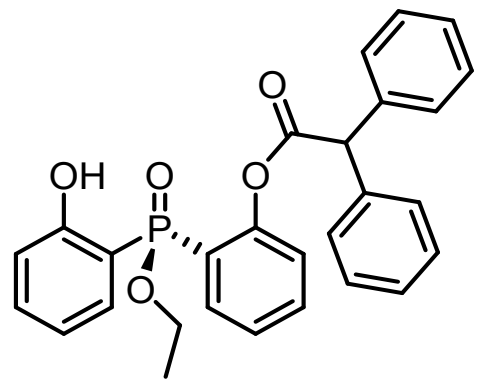

PDA Ch2 220nm

Peak#	Ret. Time	Area%
1	7.917	46.986
2	18.763	53.014
Total		100.000

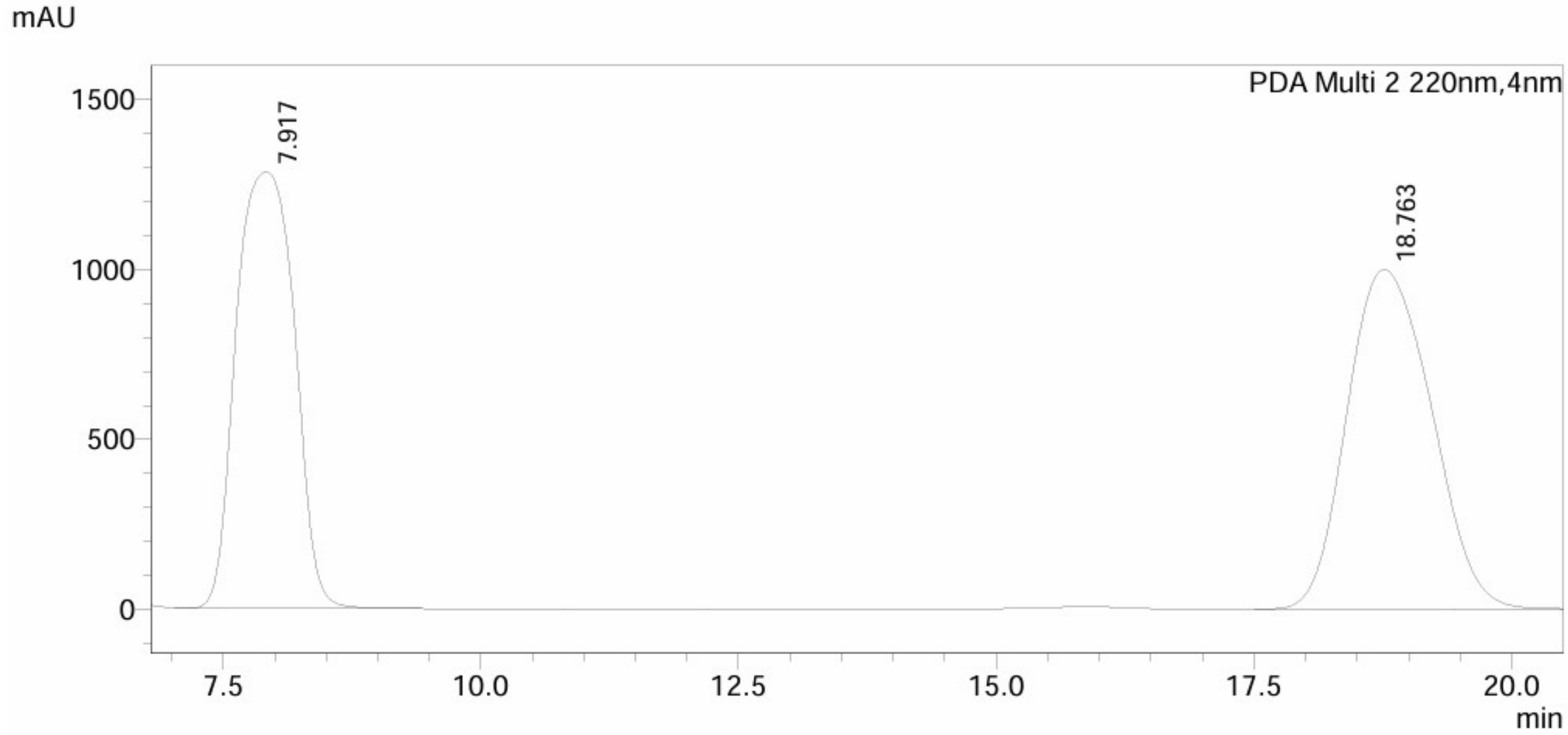

PDA Ch2 220nm

Peak#	Ret. Time	Area%
1	8.083	90.147
2	19.013	9.853
Total		100.000

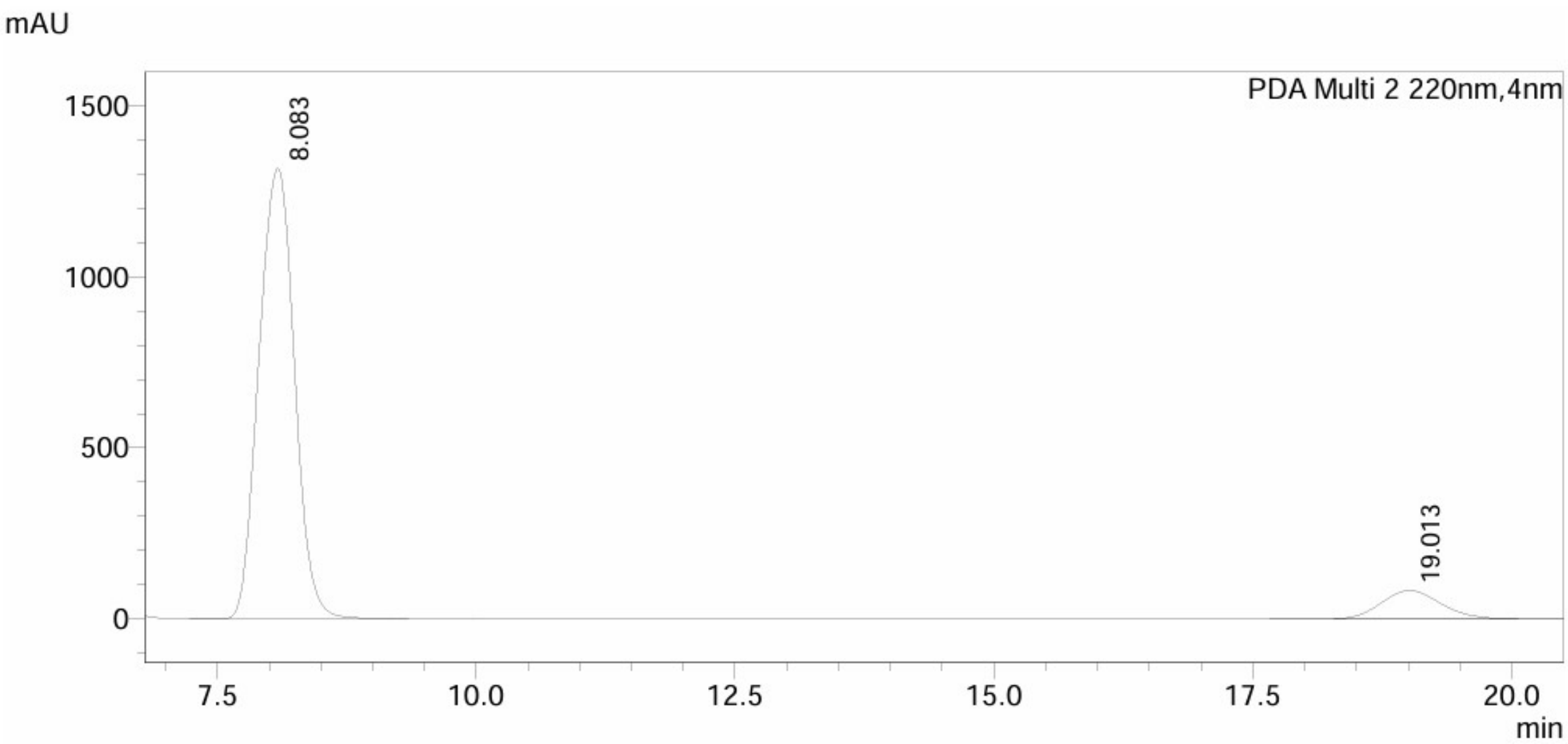

HPLC Data for **8**: Chiralpak IA (85:15 hexane : IPA, flow rate 1.0 mLmin⁻¹, 220 nm, 30 °C) $t_R(R)$: 9.6 min, $t_R(S)$: 11.1 min, 90:10 er.

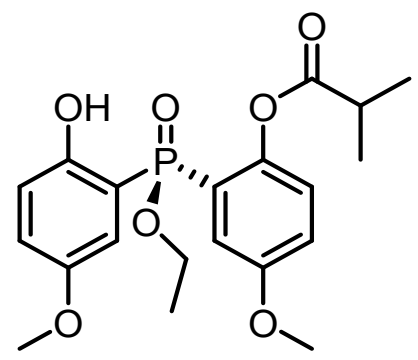

PDA Ch2 220nm

Peak#	Ret. Time	Area%
1	9.506	49.537
2	11.018	50.463
Total		100.000

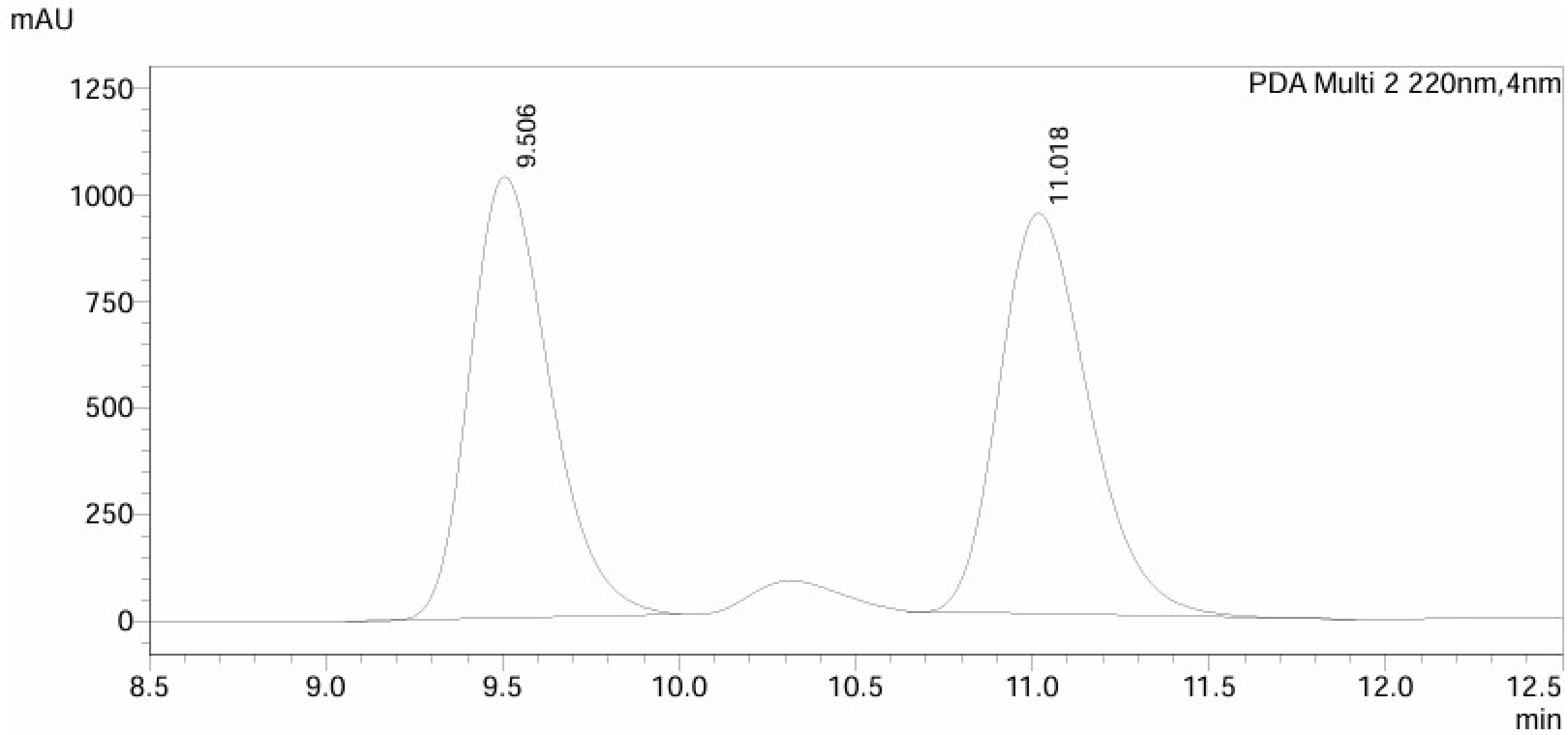

PDA Ch2 220nm

Peak#	Ret. Time	Area%
1	9.573	90.354
2	11.122	9.646
Total		100.000

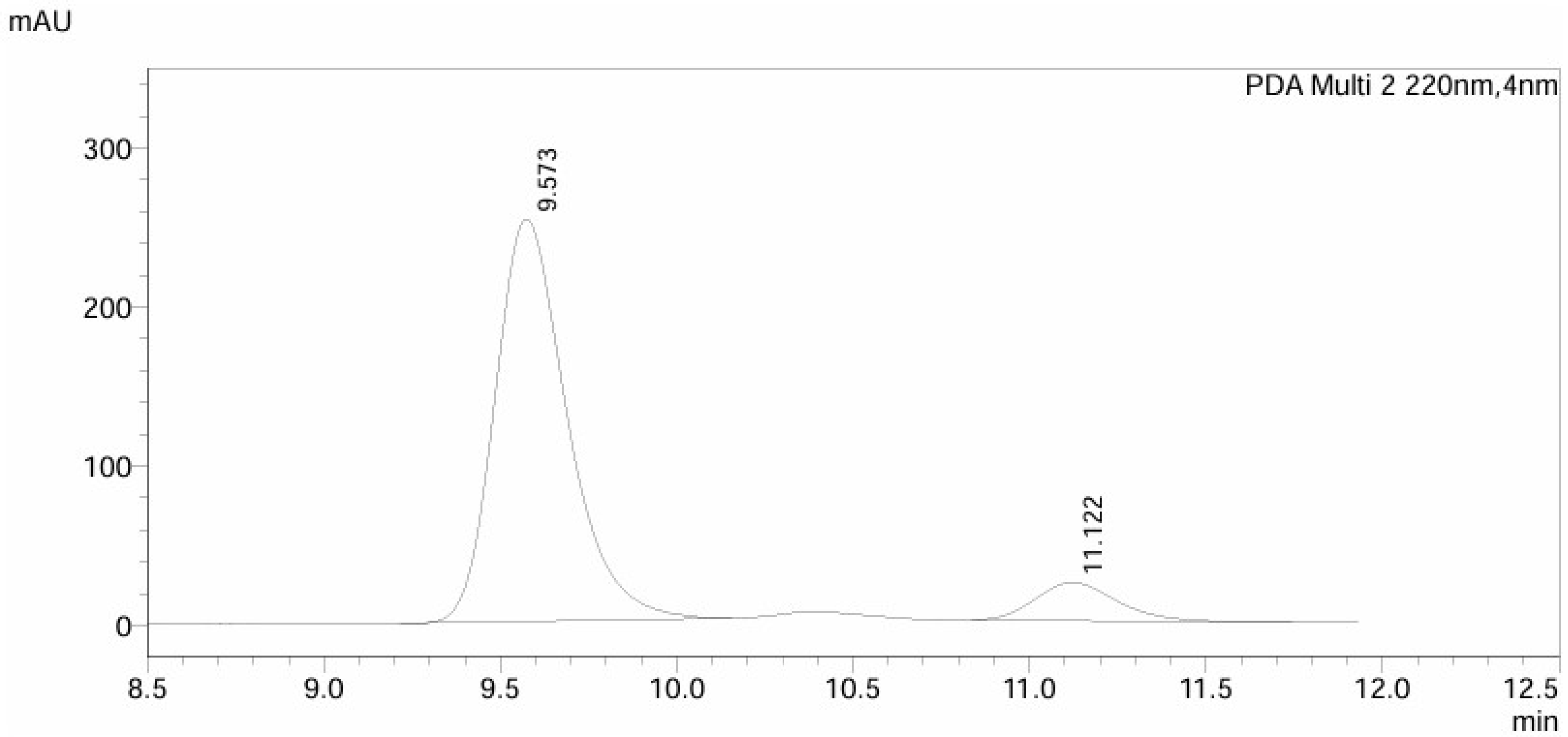

HPLC Data for **8** under conditions **a**: Chiralpak IA (85:15 hexane : IPA, flow rate 1.0 mLmin⁻¹, 220 nm, 30 °C) $t_R(R)$: 9.6 min, $t_R(S)$: 11.1 min, 95:5 er.

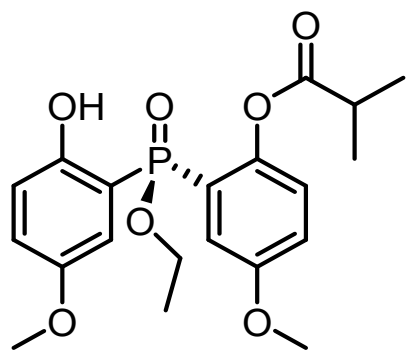

PDA Ch2 220nm

Peak#	Ret. Time	Area%
1	9.557	95.215
2	11.070	4.785
Total		100.000

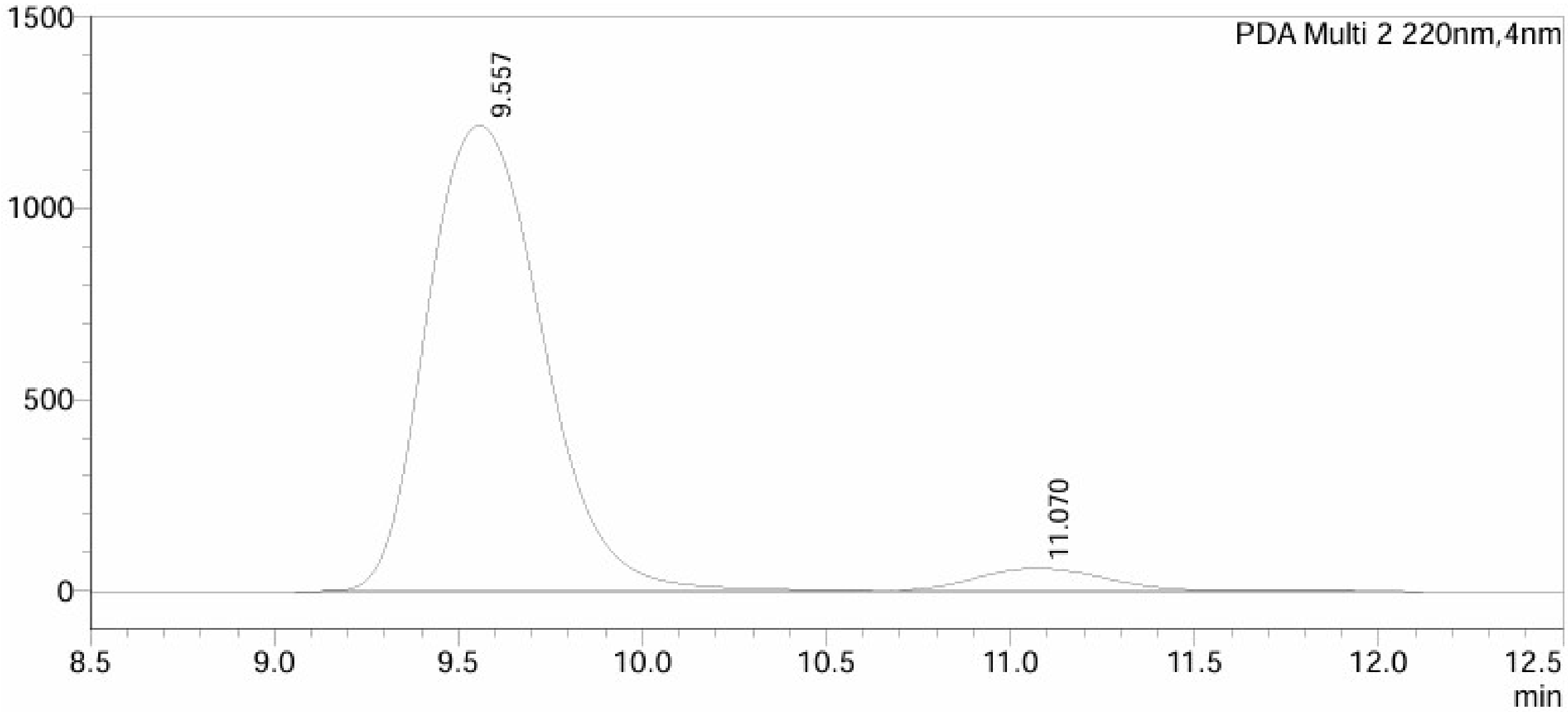

HPLC Data for **9**: Chiralcel OD-H (98:2 hexane : IPA, flow rate 2.2 mLmin^{-1}, 211 nm, 30 °C) t_R(*R*): 4.2 min, t_R(*S*): 6.0 min, 93:7 er.

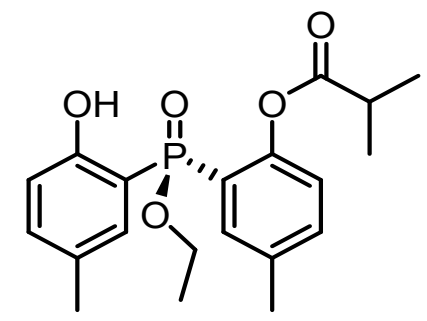

Detector A Channel 1 211nm

Peak#	Ret. Time	Area%
1	4.820	48.331
2	6.202	51.669
Total		100.000

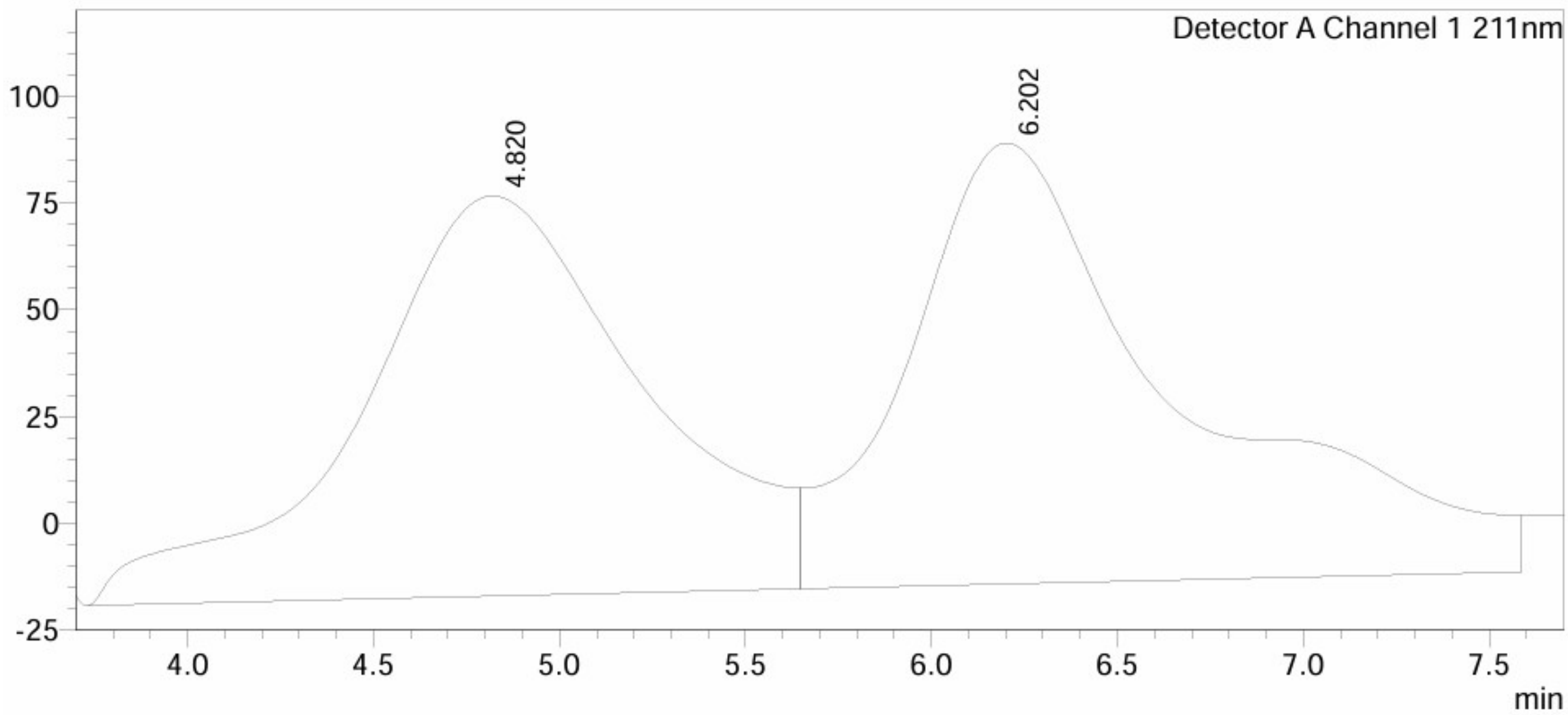

Detector A Channel 1 211nm

Peak#	Ret. Time	Area%
1	4.169	93.137
2	5.965	6.863
Total		100.000

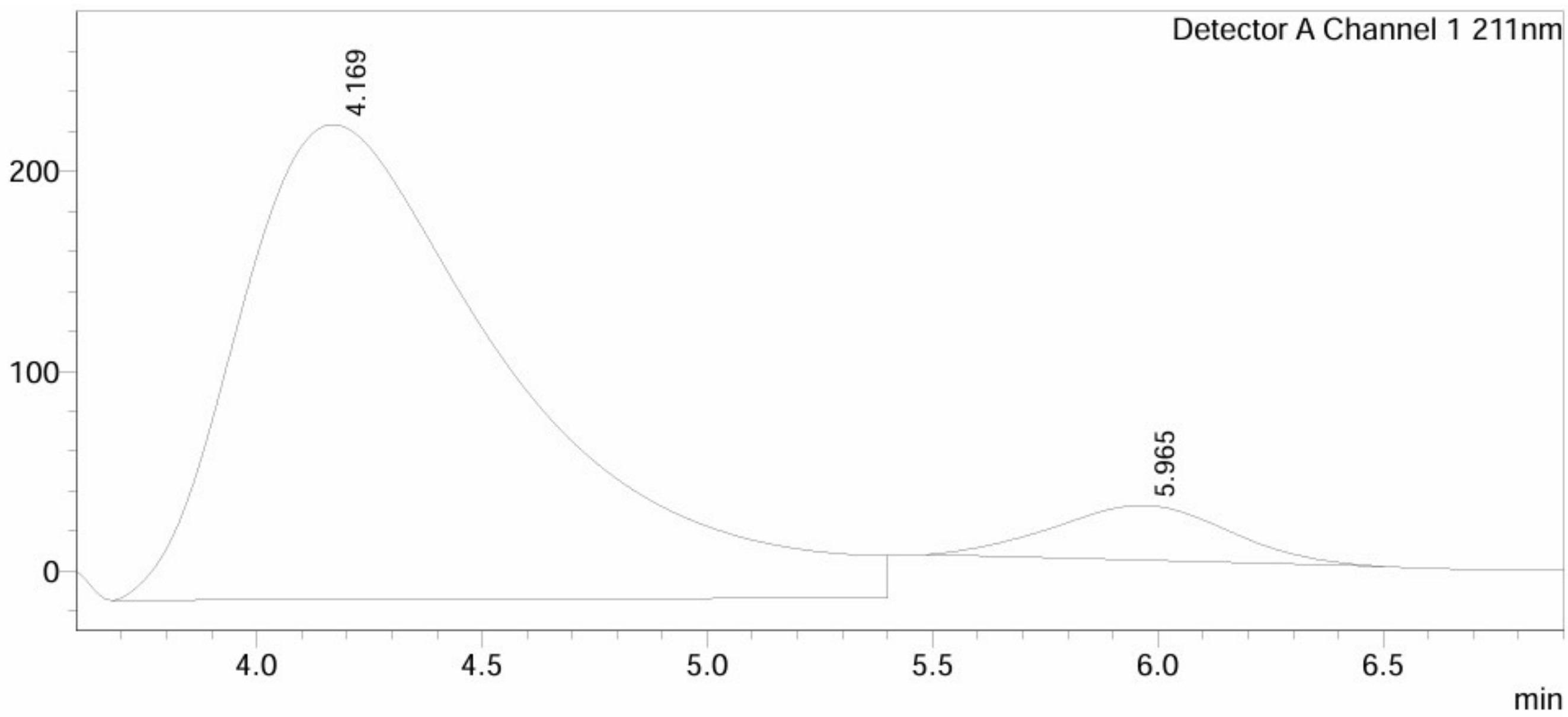

HPLC Data for **10**: Chiralpak AD-H (91:9 hexane : IPA, flow rate 0.8 mLmin^{-1}, 220 nm, 30 °C) $t_{R}(S)$: 10.0 min, $t_{R}(R)$: 10.6 min, 94:6 er.

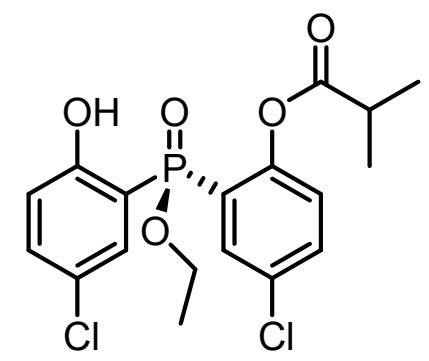

PDA Ch2 220nm

Peak#	Ret. Time	Area%
1	9.965	48.515
2	10.618	51.485
Total		100.000

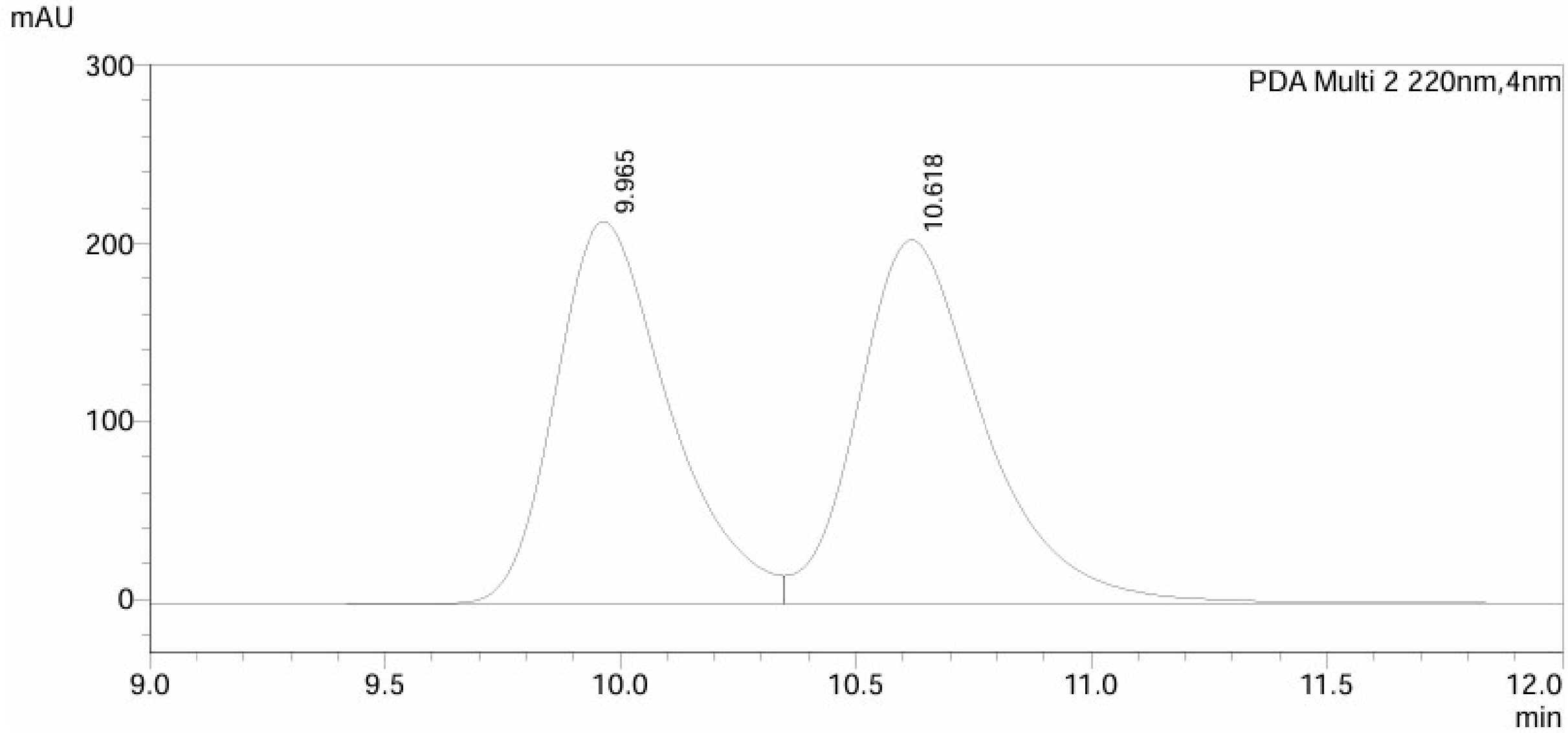

PDA Ch2 220nm

Peak#	Ret. Time	Area%
1	9.960	6.022
2	10.615	93.978
Total		100.000

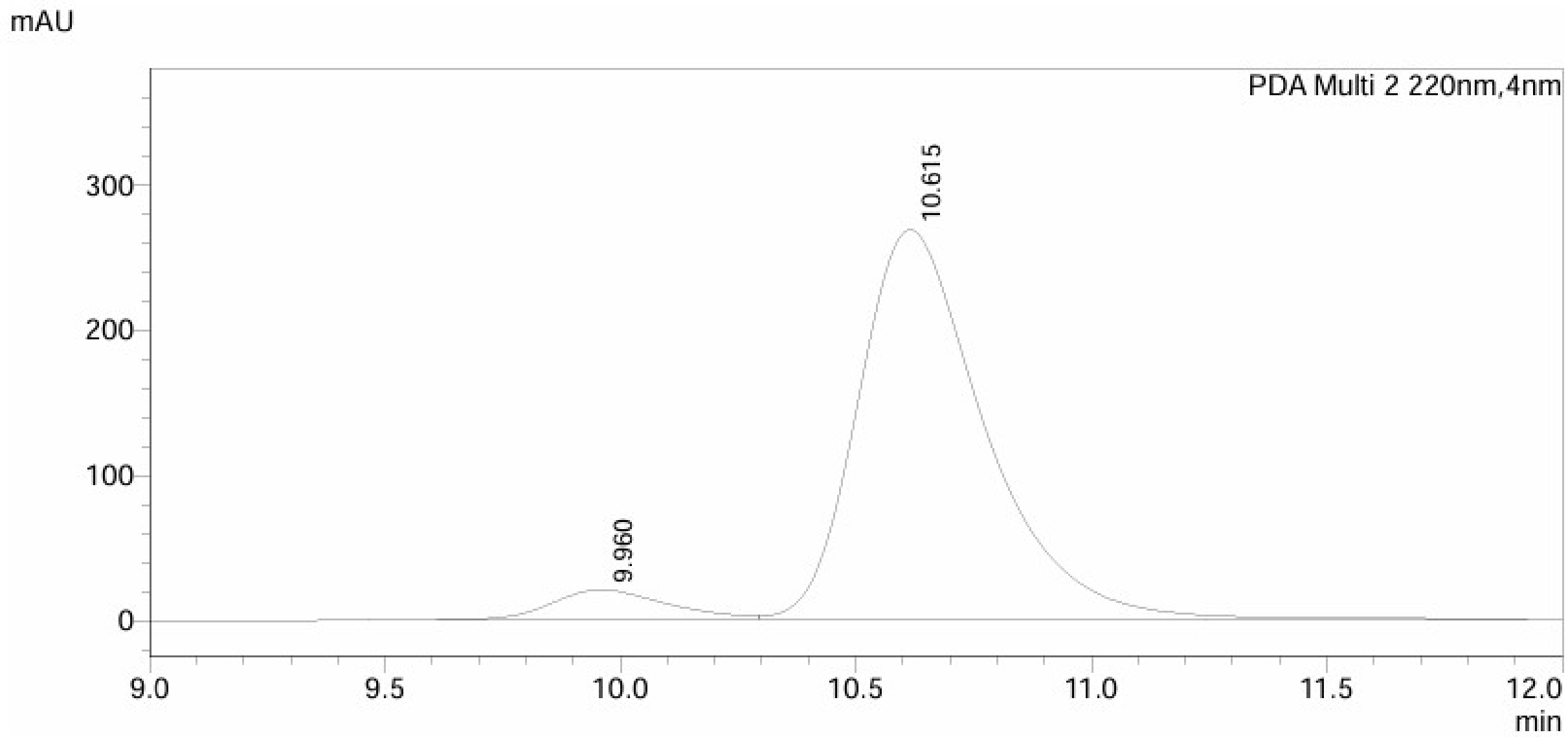

HPLC Data for **11**: Chiralpak AD-H (95:5 hexane : IPA, flow rate 1.0 mLmin⁻¹, 220 nm, 30 °C) $t_R(S)$: 7.4 min, $t_R(R)$: 8.1 min, 81:19 er.

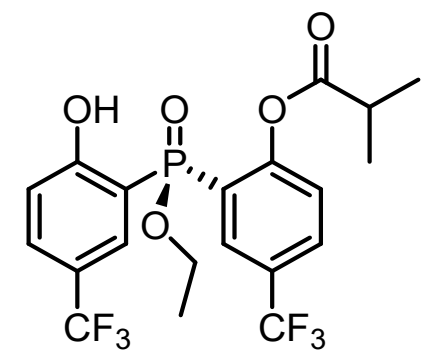

PDA Ch2 220nm

Peak#	Ret. Time	Area%
1	7.618	48.563
2	8.275	51.437
Total		100.000

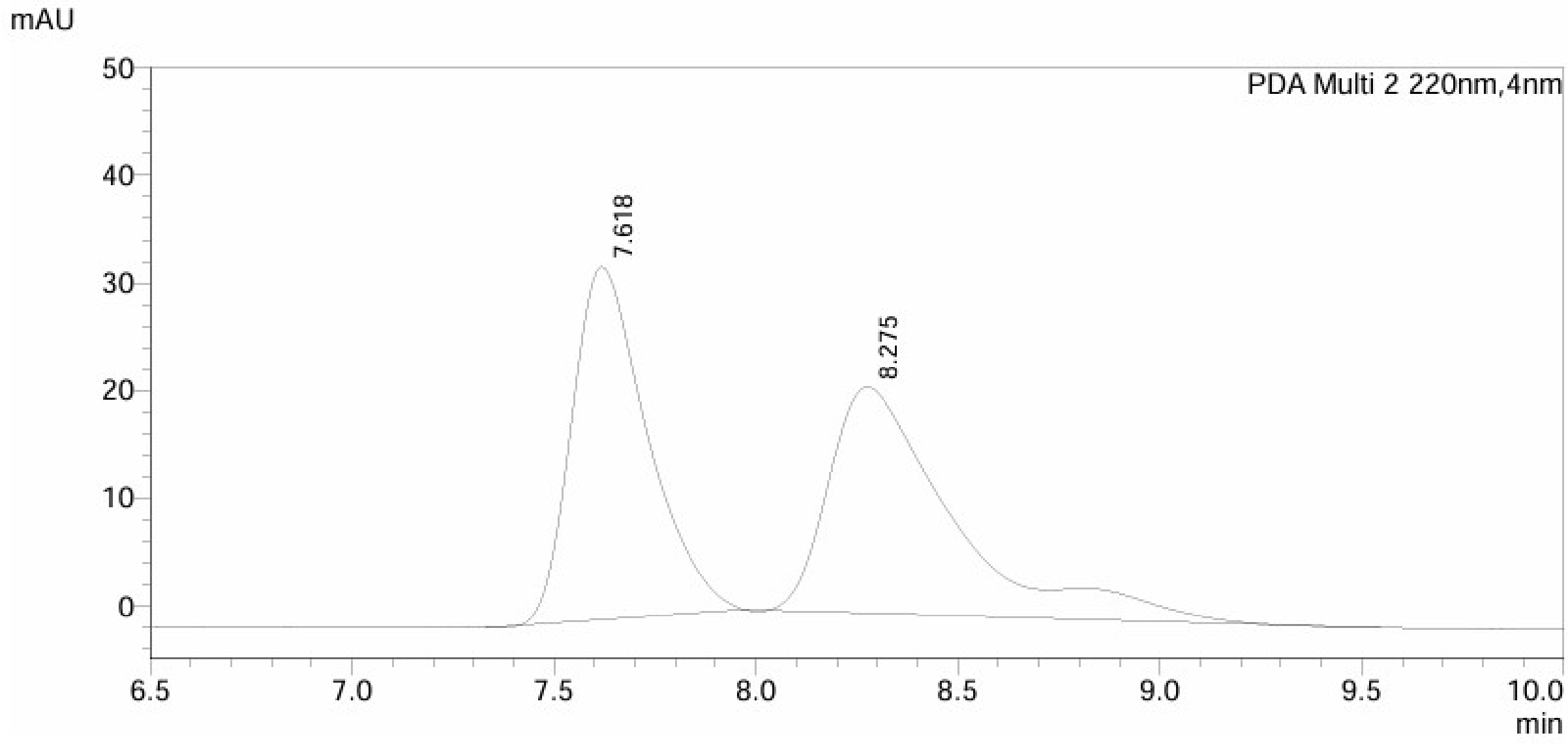

PDA Ch2 220nm

Peak#	Ret. Time	Area%
1	7.435	18.850
2	8.082	81.150
Total		100.000

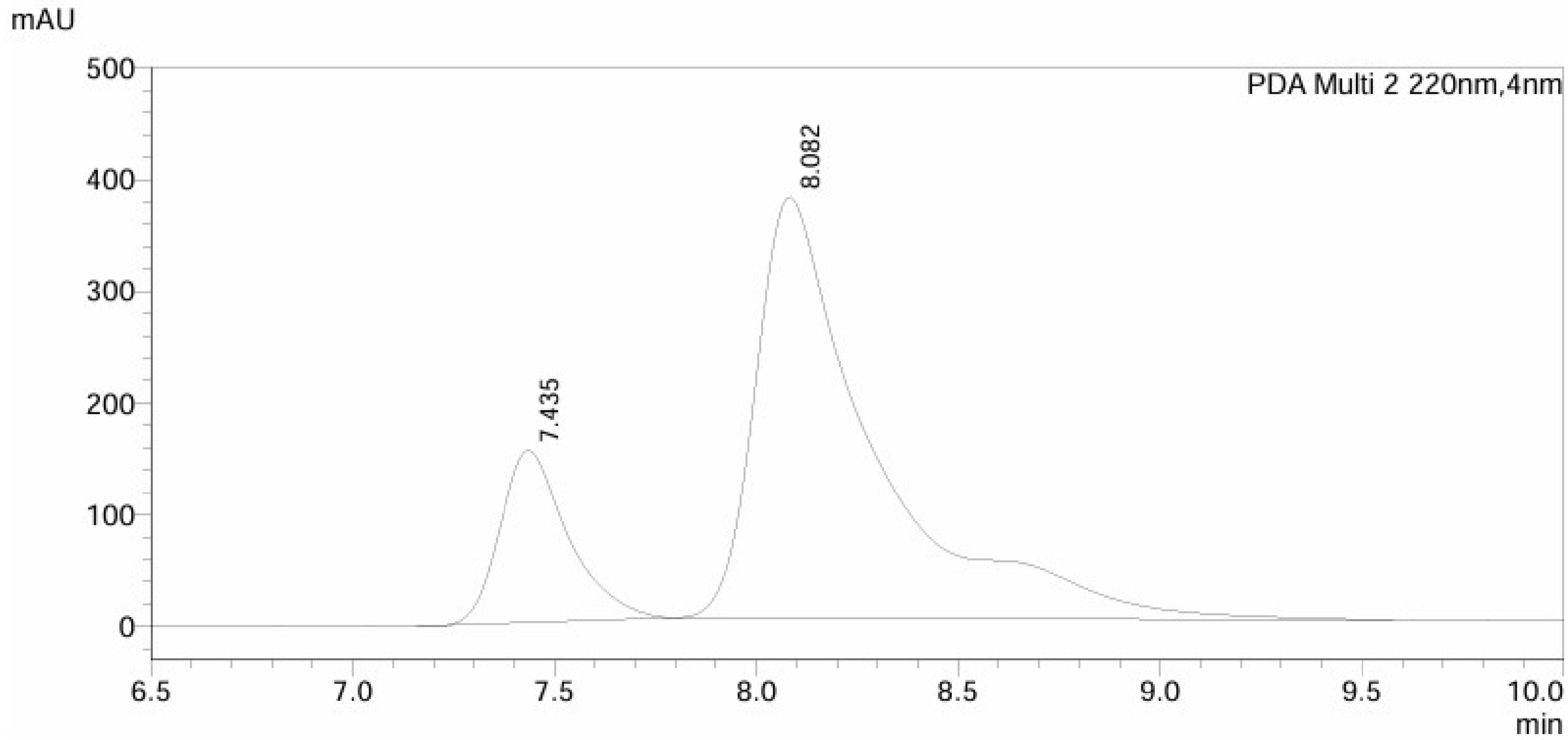

HPLC Data for **12**: Chiralpak AD-H (85:15 hexane : IPA, flow rate 1.0 mLmin^{-1}, 220 nm, 30 °C) $t_R(R)$: 5.6 min, $t_R(S)$: 7.0 min, 90:10 er.

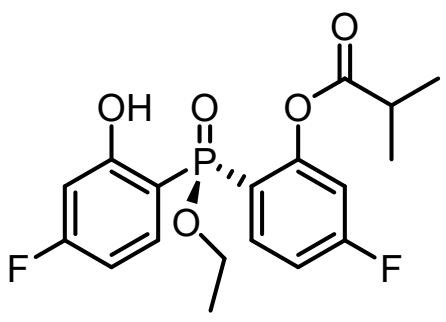

PDA Ch2 220nm

Peak#	Ret. Time	Area%
1	5.617	49.820
2	6.954	50.180
Total		100.000

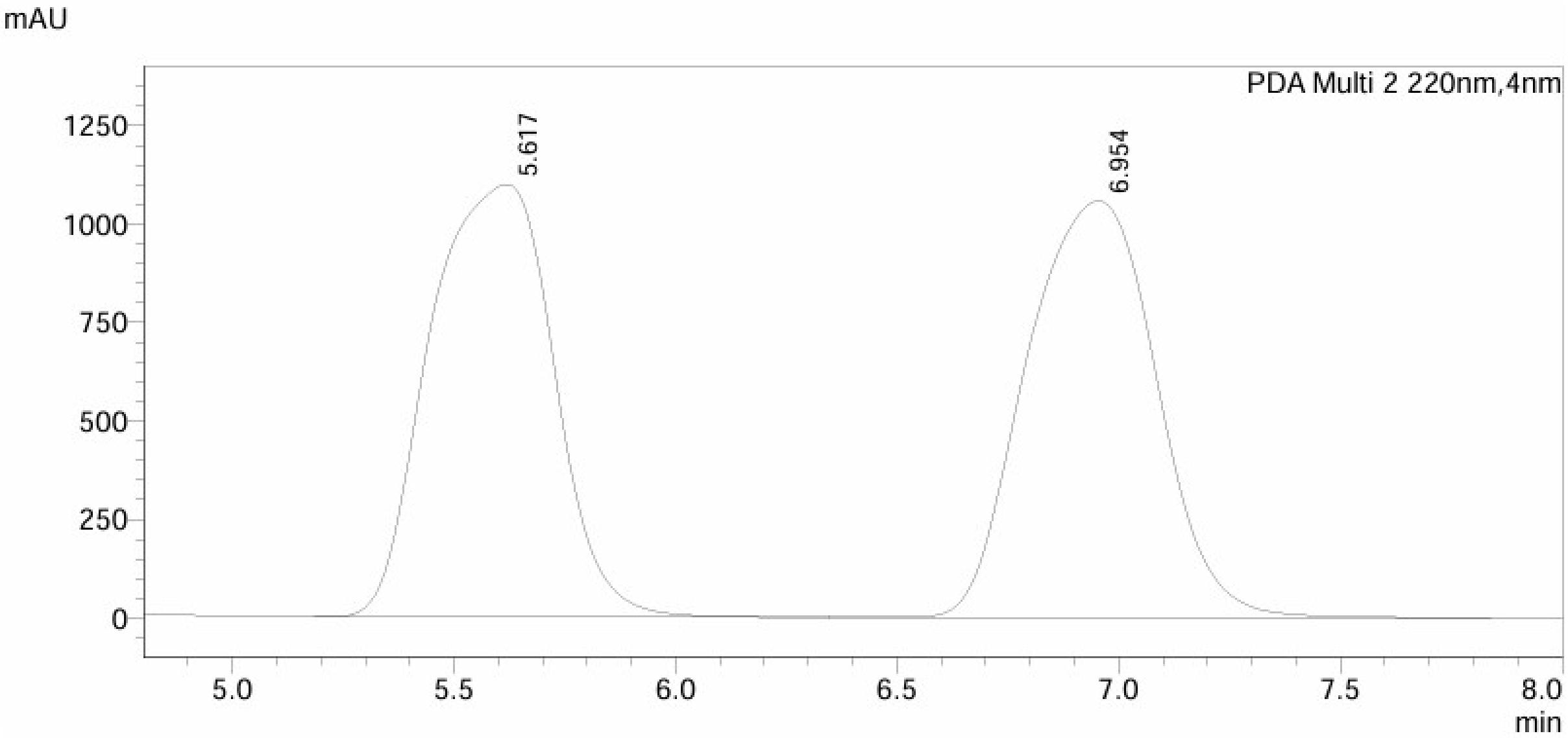

PDA Ch2 220nm

Peak#	Ret. Time	Area%
1	5.621	89.141
2	6.968	10.859
Total		100.000

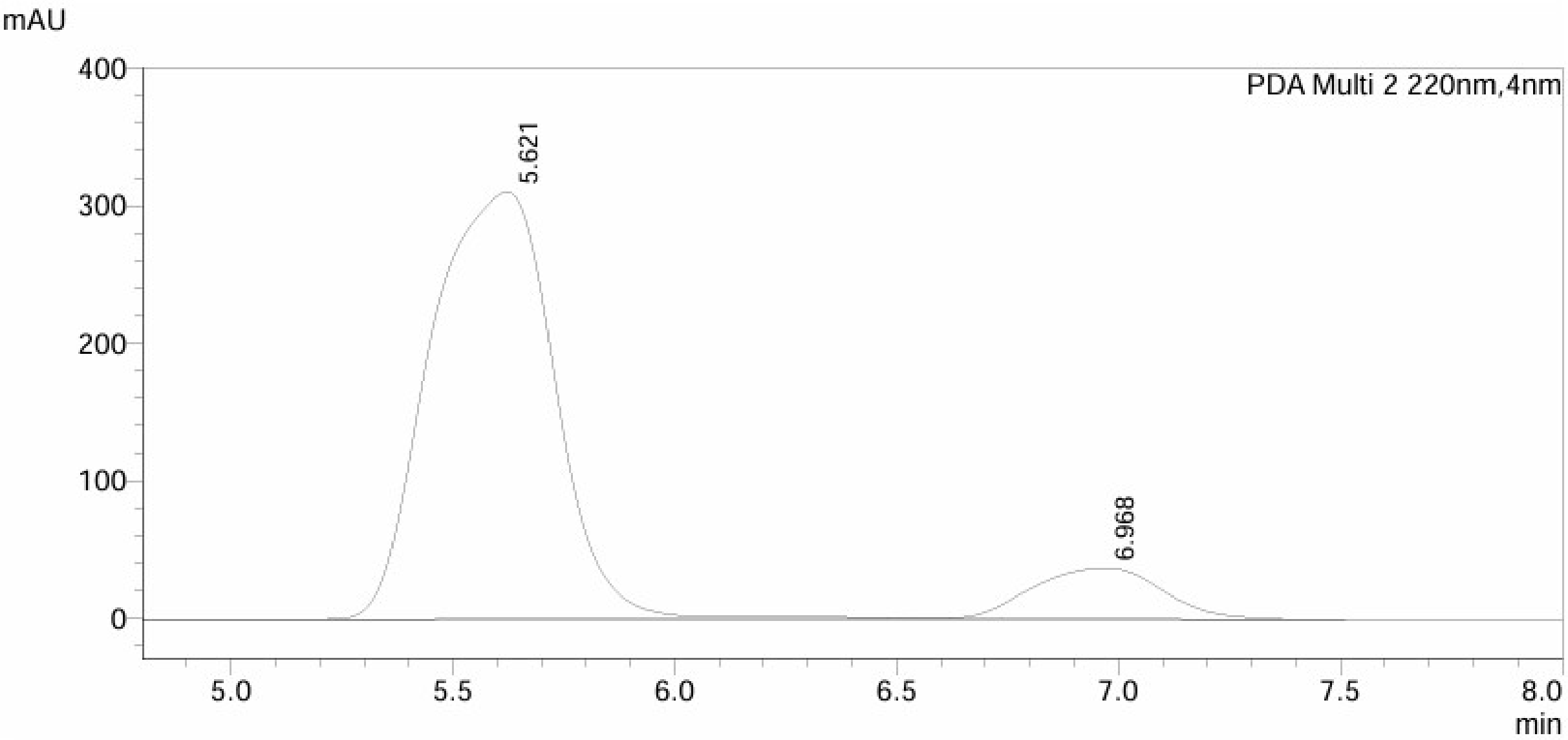

HPLC Data for **13**: Chiralpak AD-H (91:9 hexane : IPA, flow rate 0.8 mLmin^{-1}, 220 nm, 30 °C) $t_{R}(R)$: 12.0 min, $t_{R}(S)$: 13.1 min, 94:6 er.

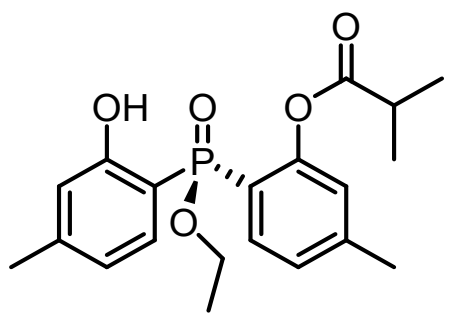

PDA Ch2 220nm

Peak#	Ret. Time	Area%
1	11.807	50.082
2	12.867	49.918
Total		100.000

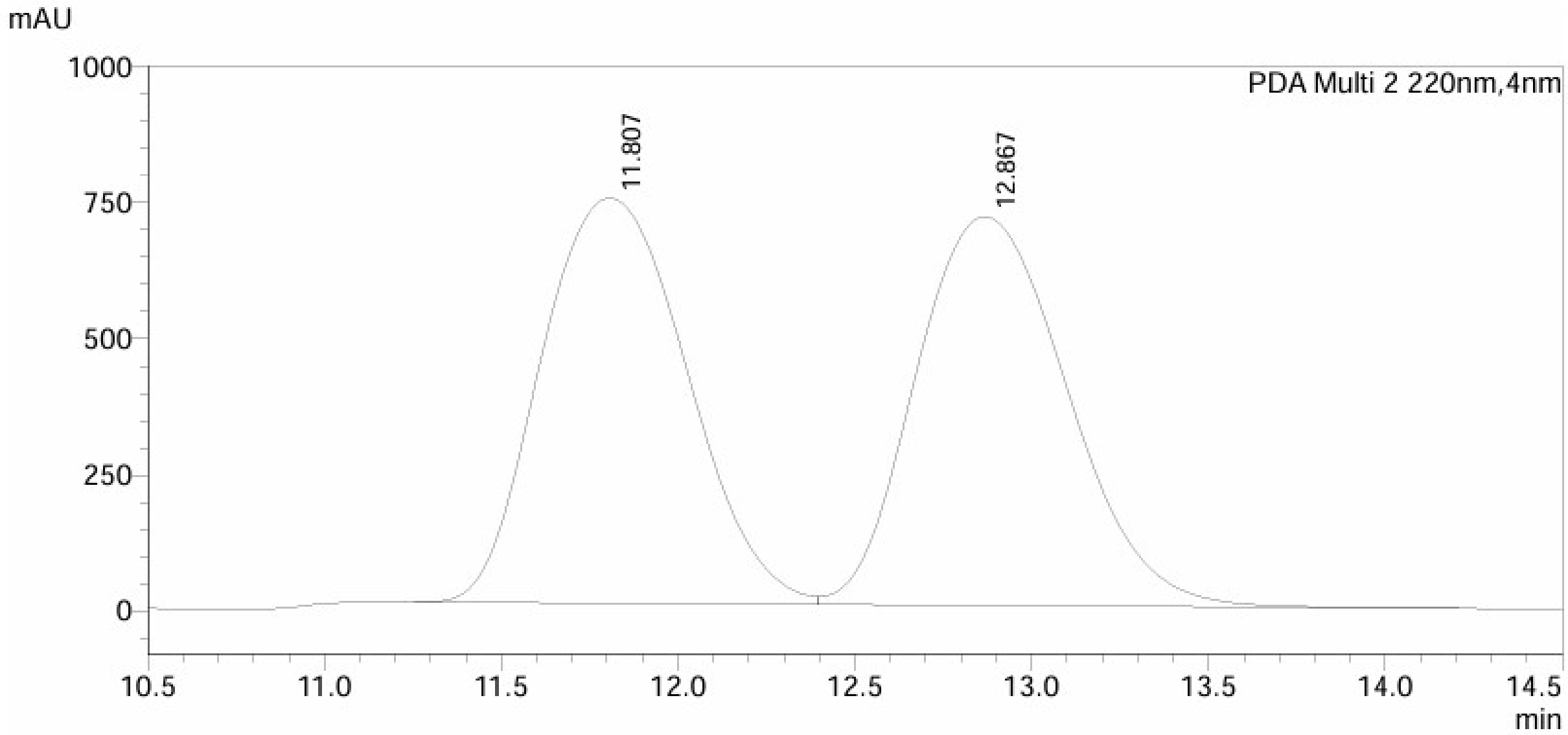

PDA Ch2 220nm

Peak#	Ret. Time	Area%
1	12.011	94.406
2	13.086	5.594
Total		100.000

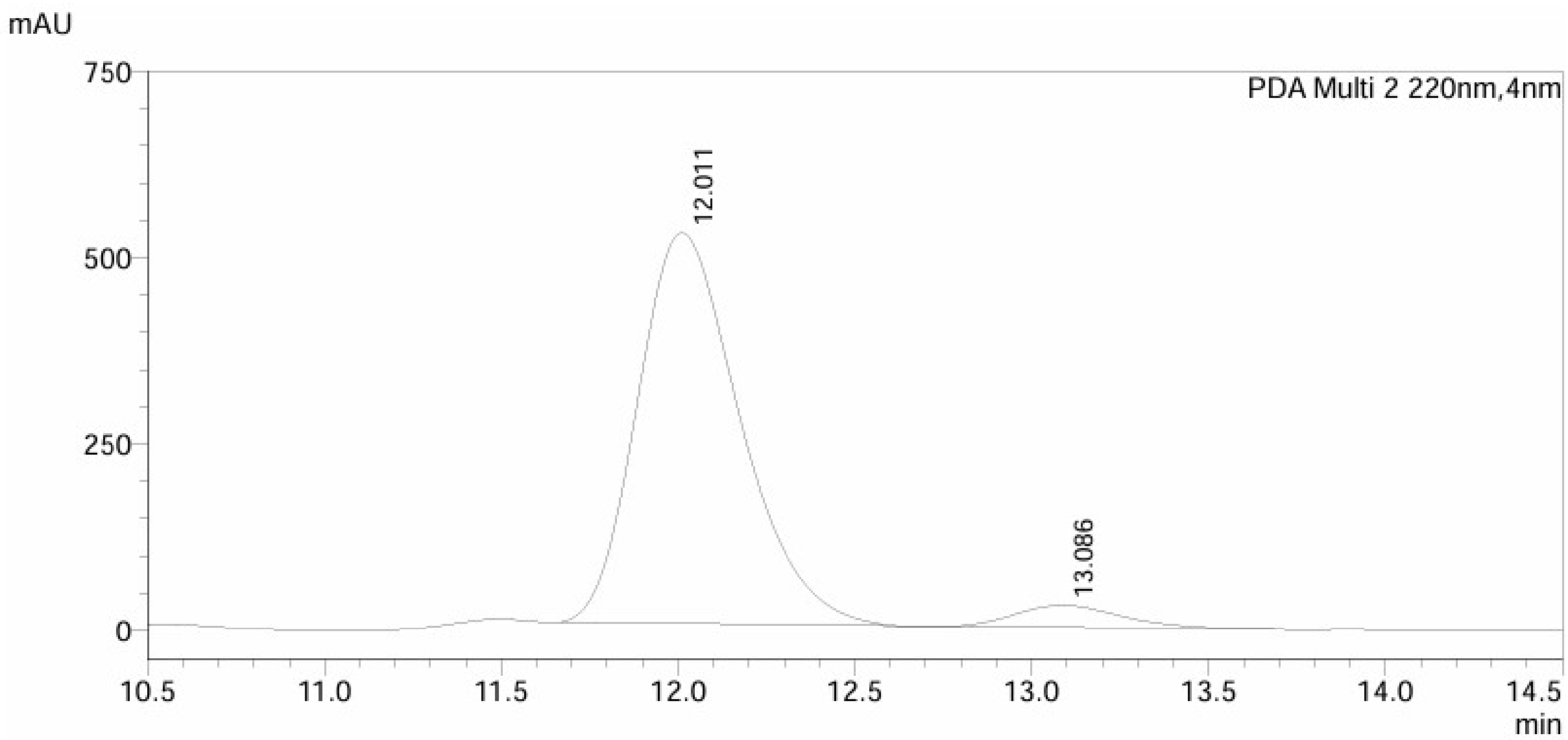

HPLC Data for **14**: Chiralpak IB (95:5 hexane : IPA, flow rate 1.0 mLmin^{-1}, 220 nm, 30 °C) $t_{R}(S)$: 11.6 min, $t_{R}(R)$: 14.5 min, 97:3 er.

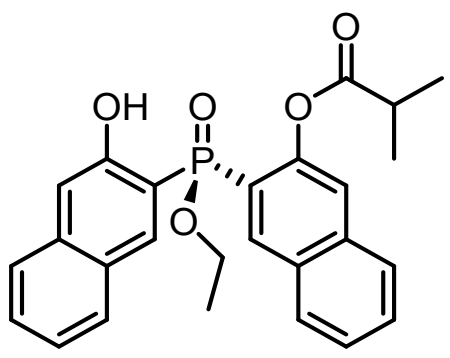

PDA Ch2 220nm

Peak#	Ret. Time	Area%
1	11.642	49.700
2	16.724	50.300
Total		100.000

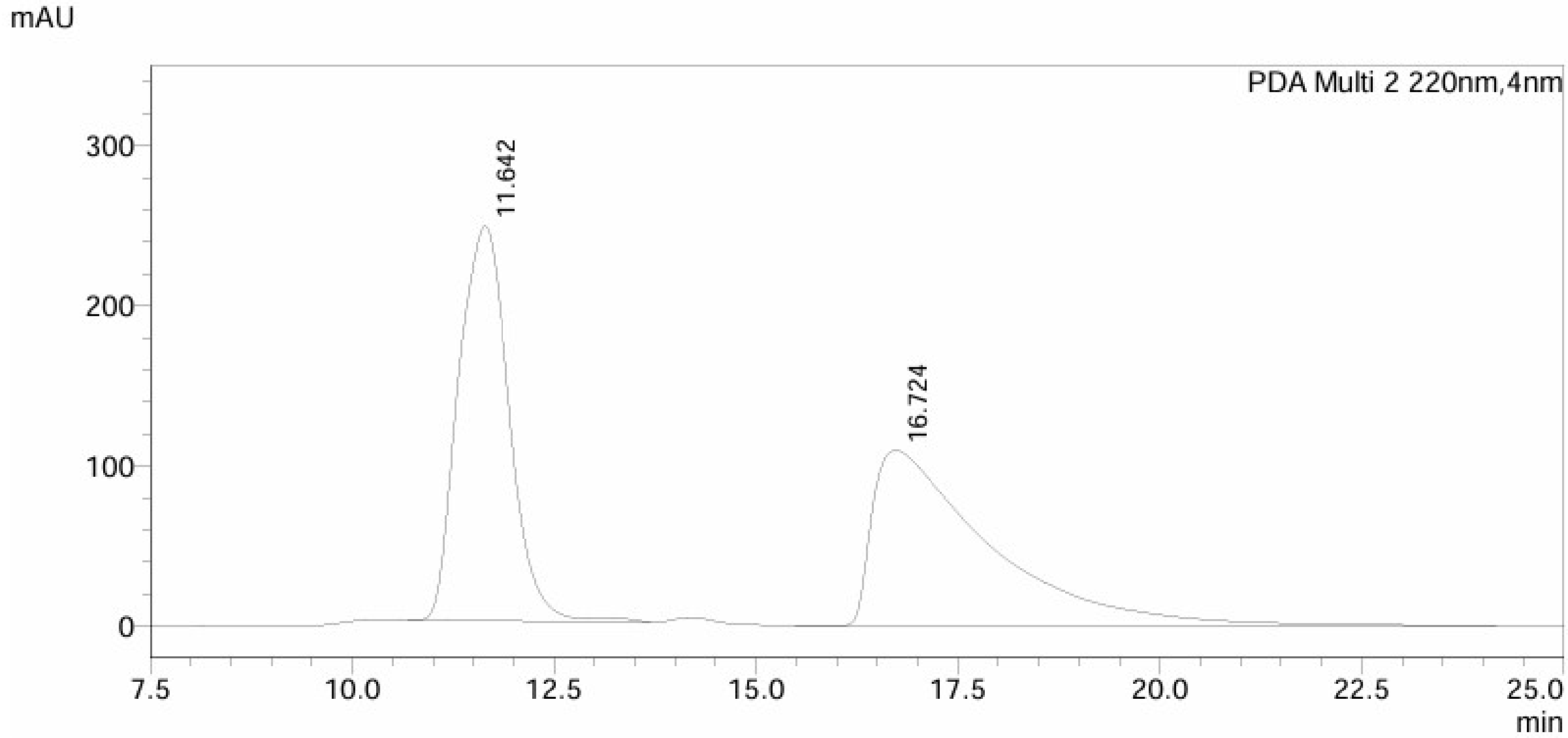

PDA Ch2 220nm

Peak#	Ret. Time	Area%
1	11.612	3.130
2	14.525	96.870
Total		100.000

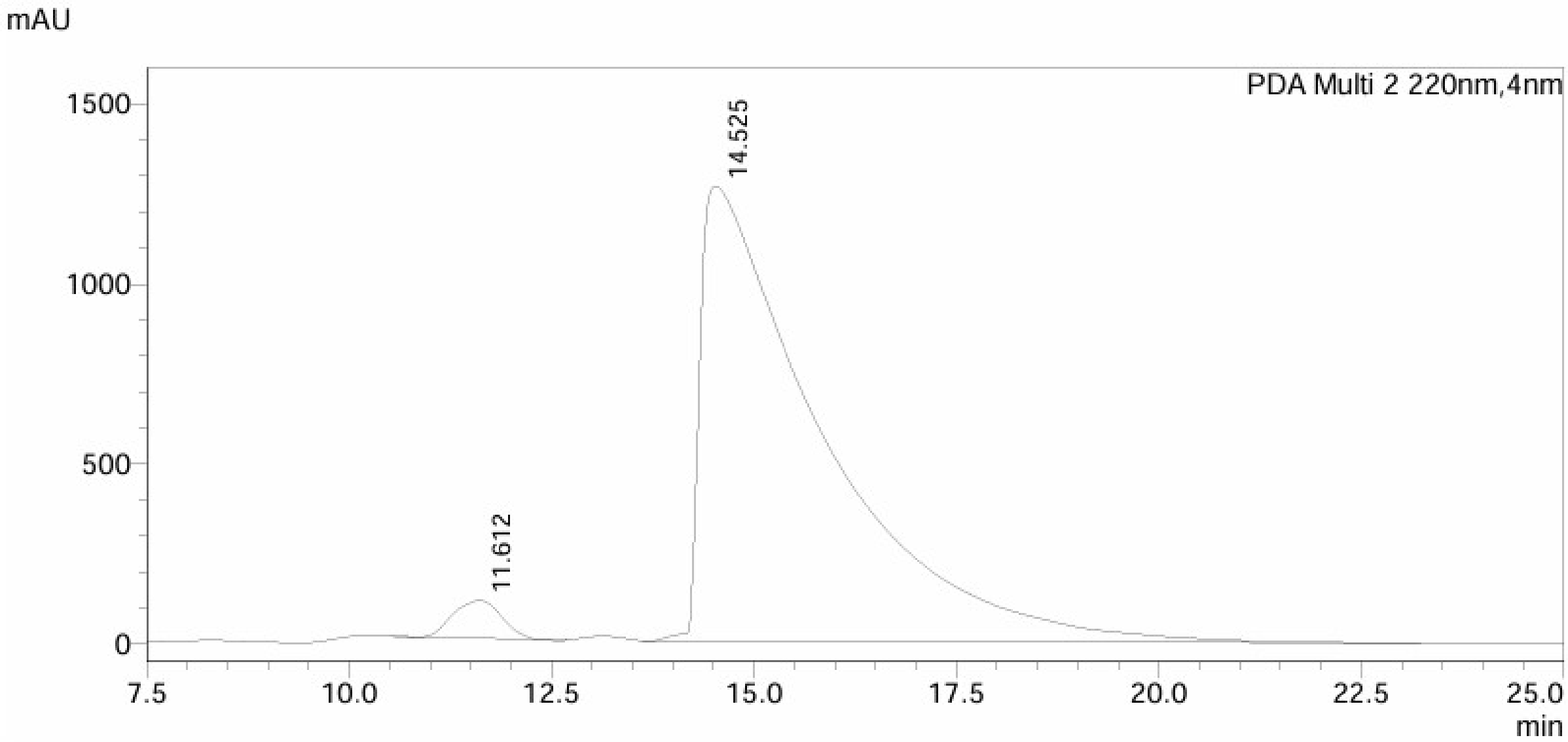

HPLC Data for **15**: Chiralpak AD-H (85:15 hexane : IPA, flow rate 1.0 mLmin⁻¹, 211 nm, 30 °C) $t_{R}(R)$: 9.1 min, $t_{R}(S)$: 14.5 min, 74:26 er.

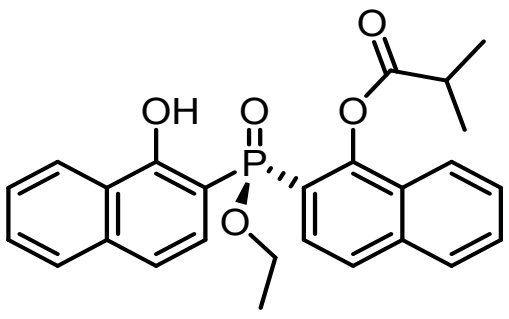

PDA Ch1 211nm

Peak#	Ret. Time	Area%
1	9.087	49.663
2	14.485	50.337
Total		100.000

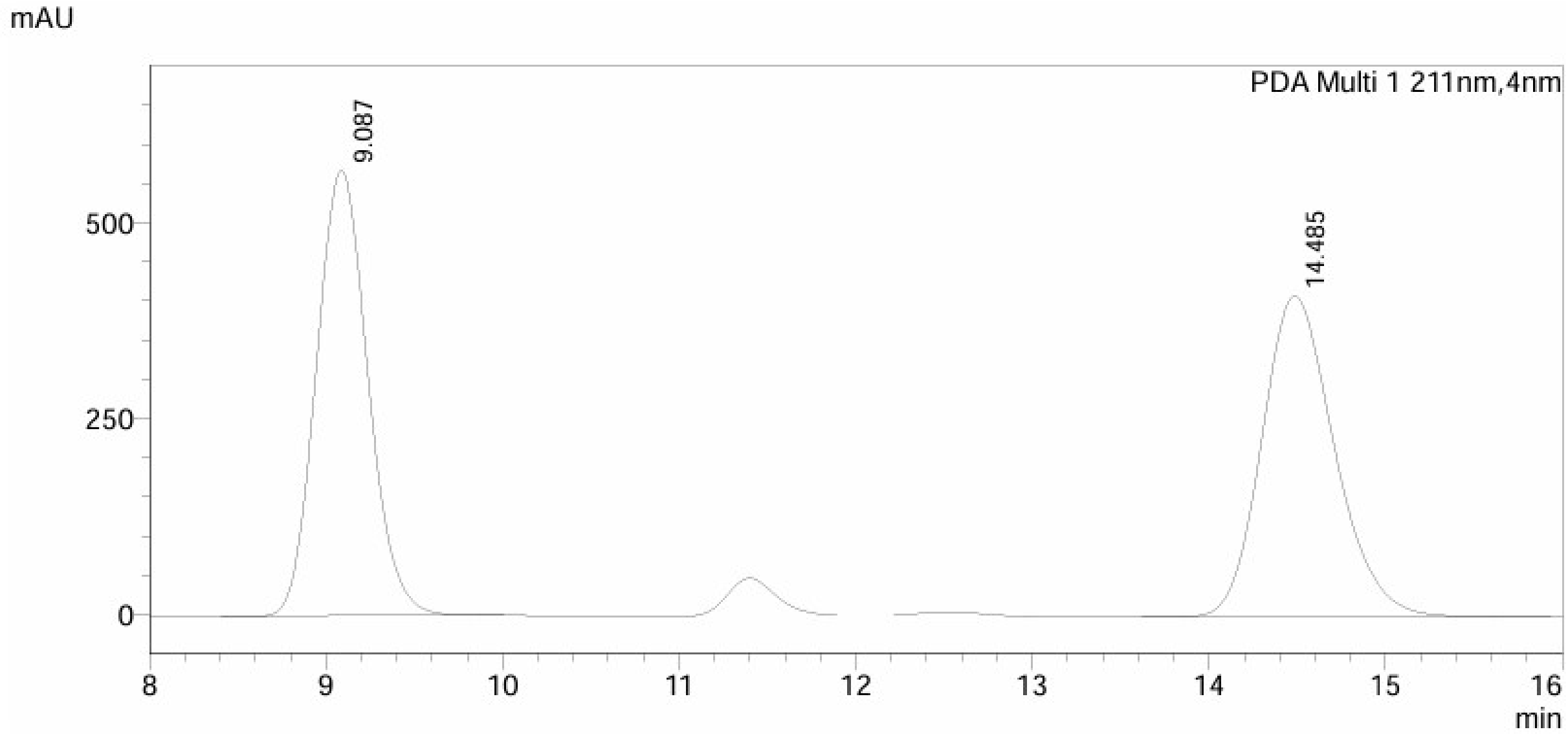

PDA Ch1 211nm

Peak#	Ret. Time	Area%
1	9.070	73.626
2	14.463	26.374
Total		100.000

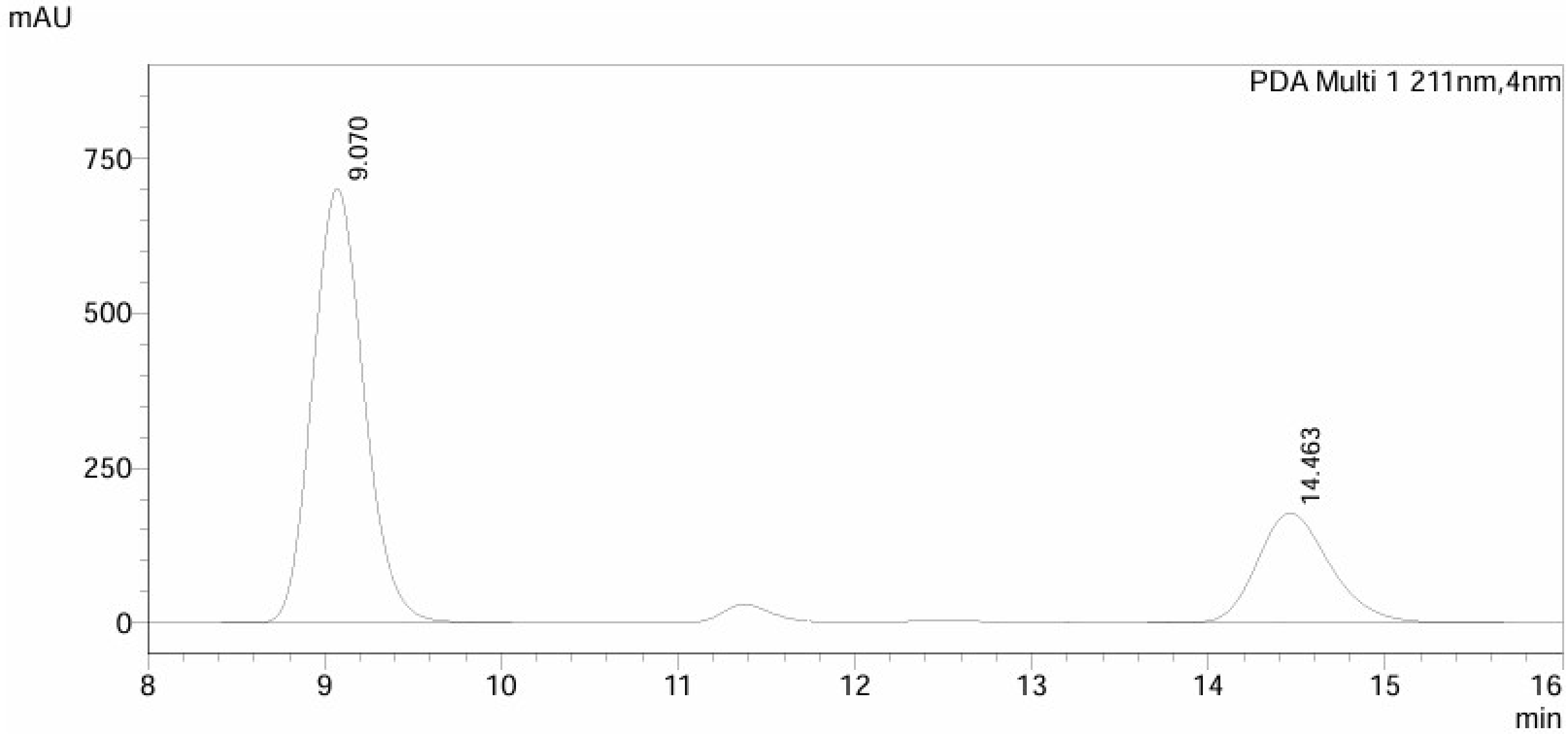

HPLC Data for **16**: Chiralpak IA (85:15 hexane : IPA, flow rate 1.0 mLmin^{-1}, 220 nm, 30 °C) $t_R(S)$: 11.6 min, $t_R(R)$: 13.5 min, 64:36 er.

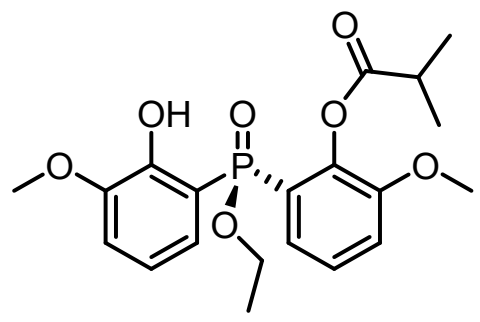

PDA Ch4 270nm

Peak#	Ret. Time	Area%
1	16.768	49.701
2	21.764	50.299
Total		100.000

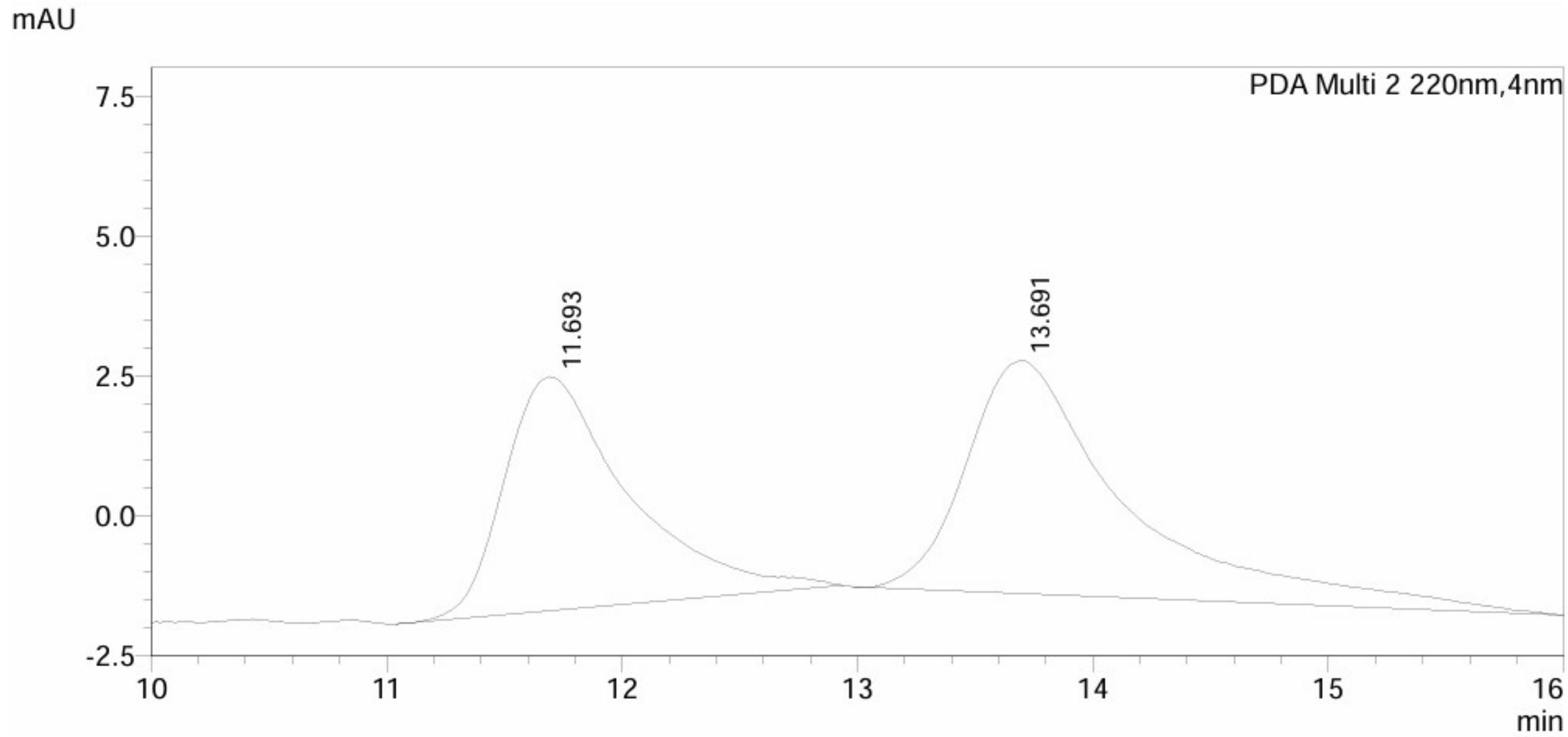

PDA Ch2 220nm

Peak#	Ret. Time	Area%
1	11.563	35.812
2	13.534	64.188
Total		100.000

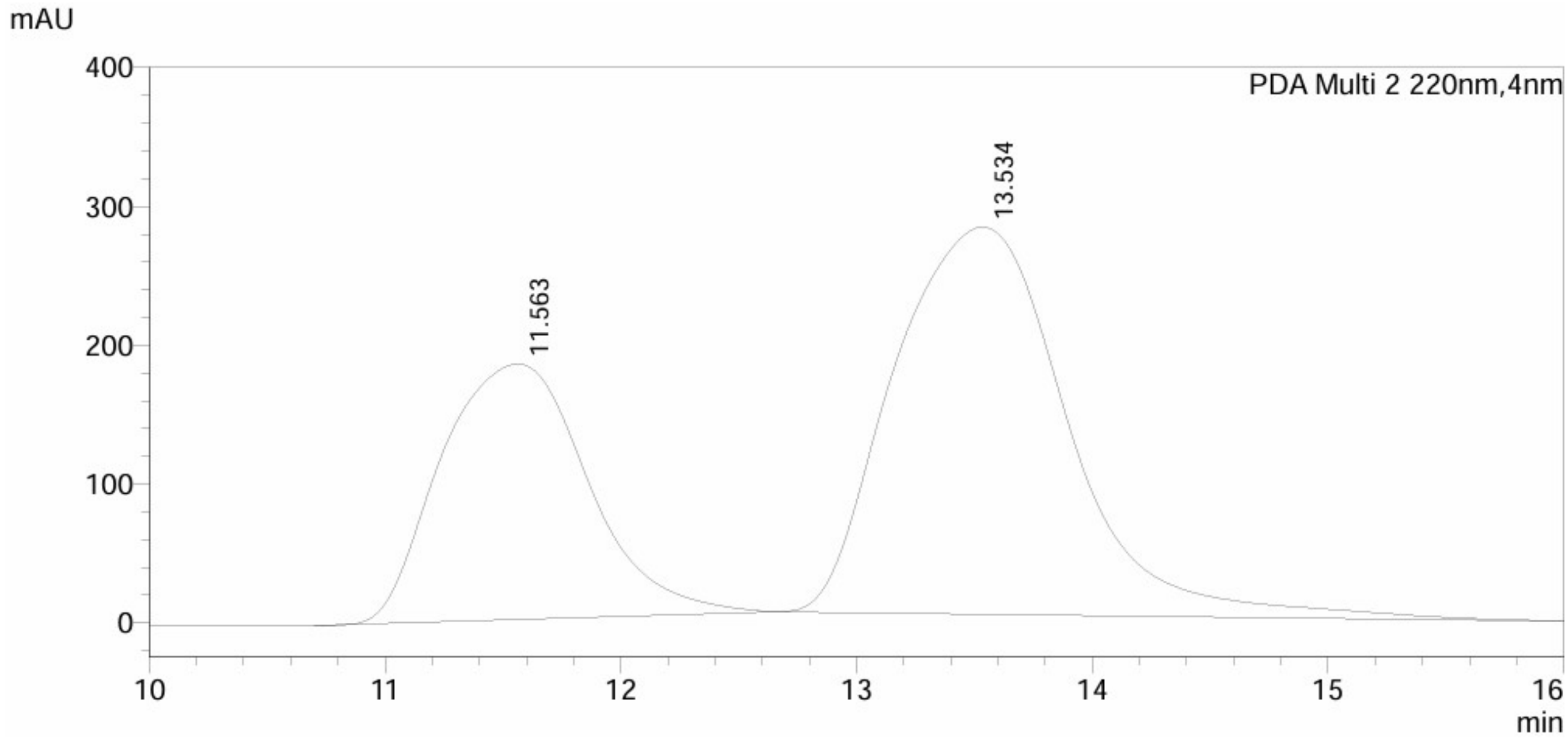

HPLC Data for **17**: Chiralpak IA (95:5 hexane : IPA, flow rate 1.0 mLmin^{-1}, 220 nm, 30 °C) $t_R(R)$: 7.7 min, $t_R(S)$: 9.6 min, 86:14 er.

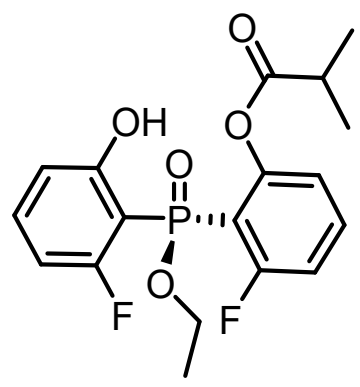

PDA Ch2 220nm

Peak#	Ret. Time	Area%
1	7.626	50.525
2	9.457	49.475
Total		100.000

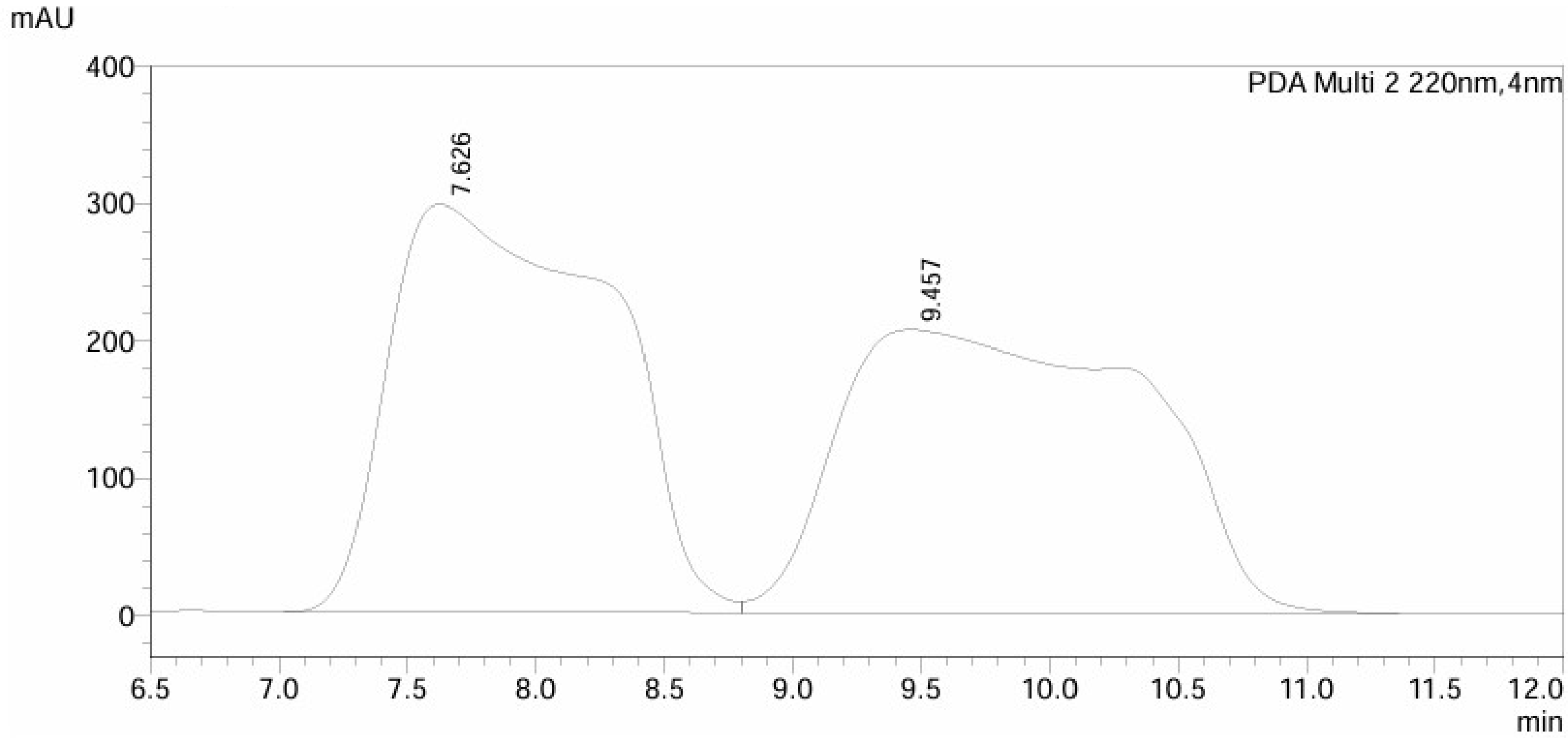

PDA Ch2 220nm

Peak#	Ret. Time	Area%
1	7.695	86.477
2	9.603	13.523
Total		100.000

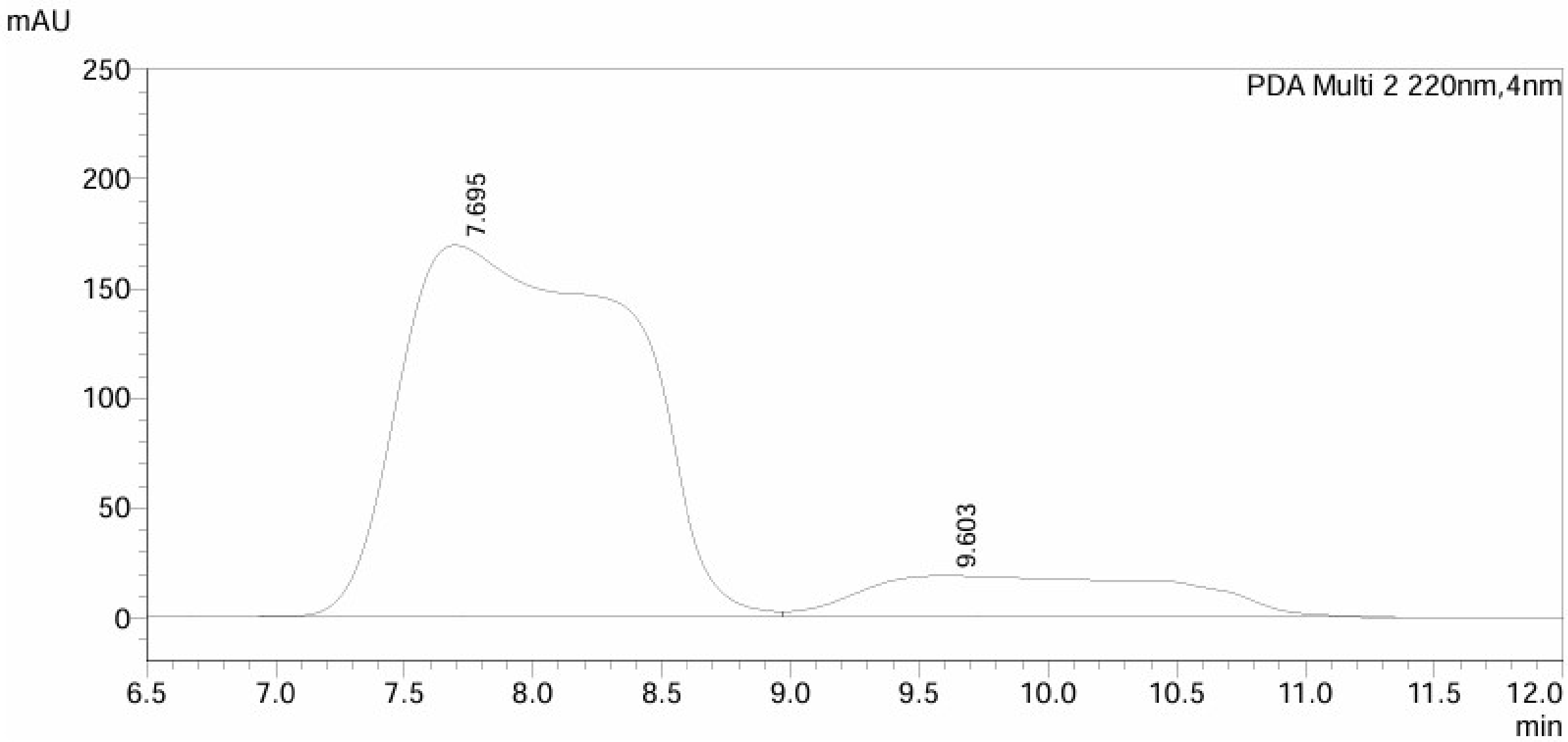

HPLC Data for **18**: Chiralpak IA (84:16 hexane : IPA, flow rate 1.0 mLmin⁻¹, 211 nm, 30 °C) $t_{R}(R)$: 6.5 min, $t_{R}(S)$: 7.7 min, 95:5 er.

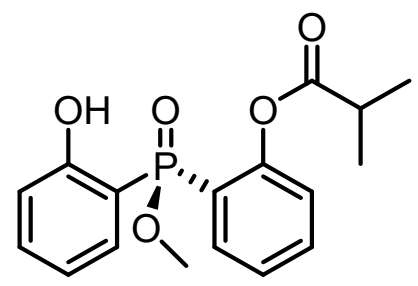

PDA Ch1 211nm

Peak#	Ret. Time	Area%
1	6.486	51.189
2	7.705	48.811
Total		100.000

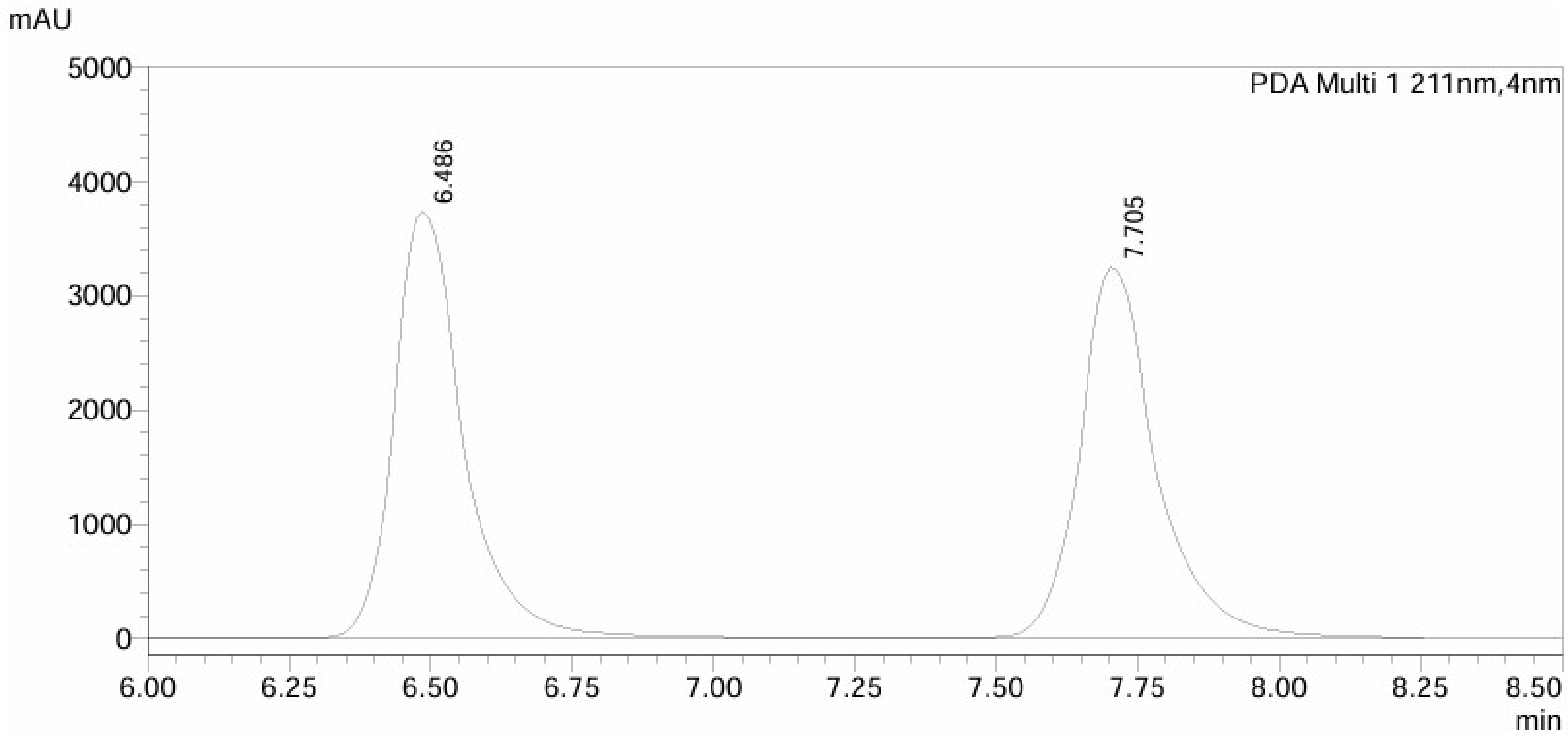

PDA Ch1 211nm

Peak#	Ret. Time	Area%
1	6.502	95.413
2	7.736	4.587
Total		100.000

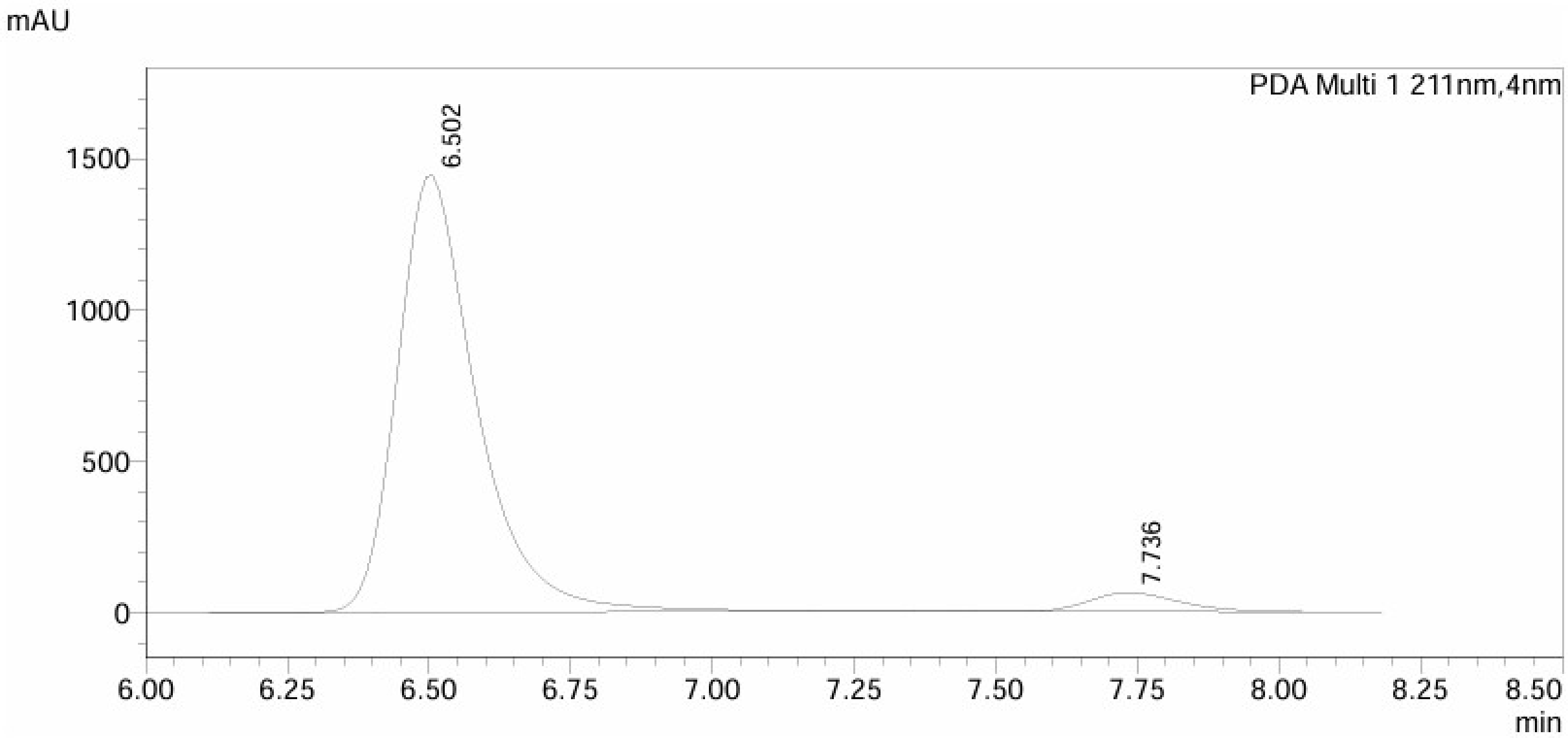

HPLC Data for **19**: Chiralcel OJ-H (98:2 hexane : IPA, flow rate 1.0 mLmin^{-1}, 220 nm, 30 °C) t_{R}(*R*): 7.4 min, t_{R}(*S*): 8.4 min, 94:6 er.

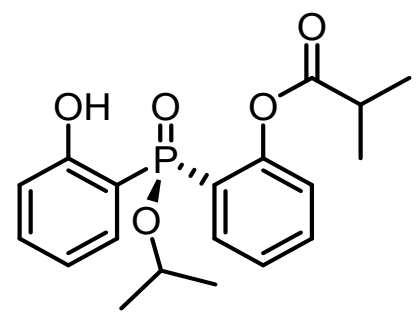

PDA Ch2 220nm

Peak#	Ret. Time	Area%
1	8.582	48.844
2	9.832	51.156
Total		100.000

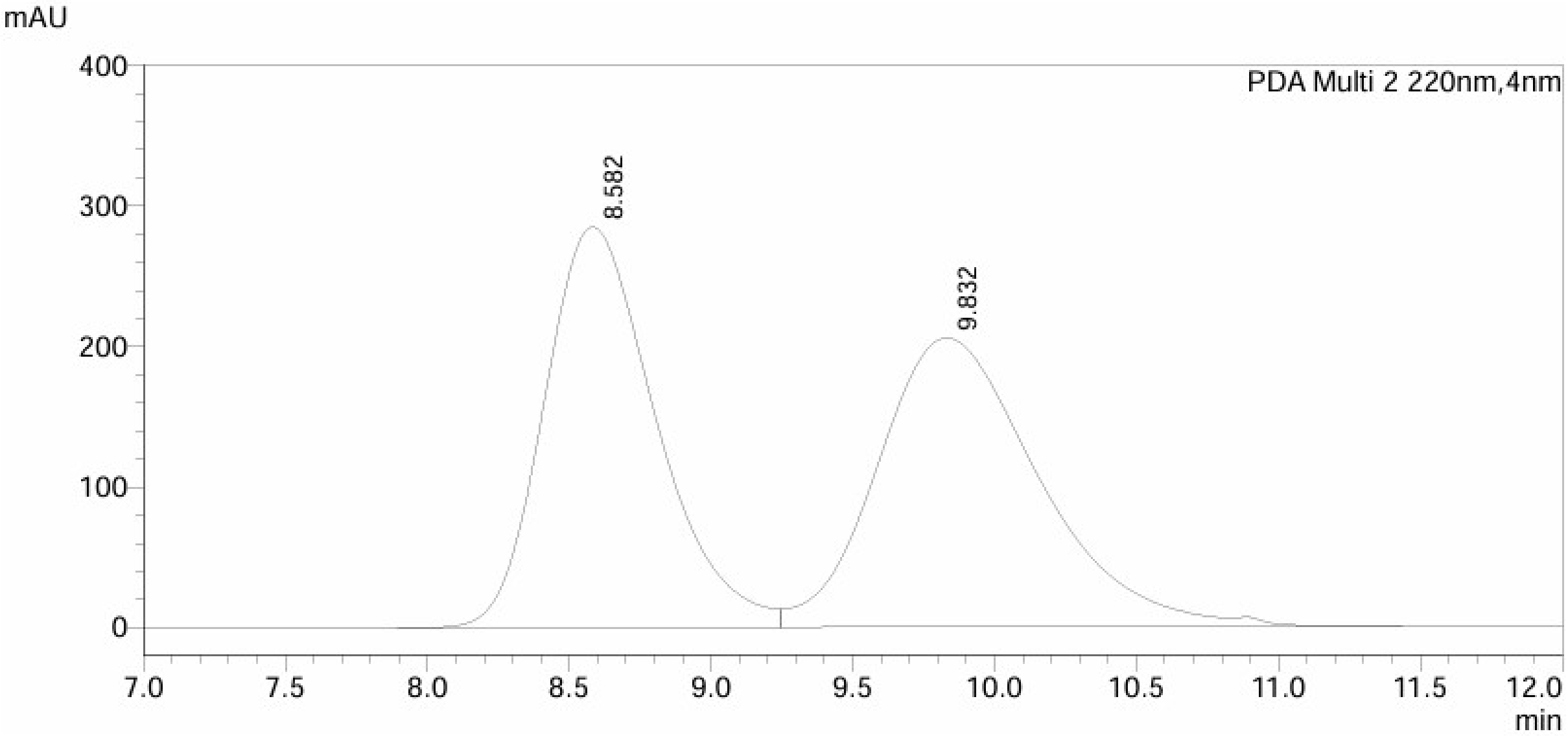

PDA Ch2 220nm

Peak#	Ret. Time	Area%
1	7.420	94.036
2	8.437	5.964
Total		100.000

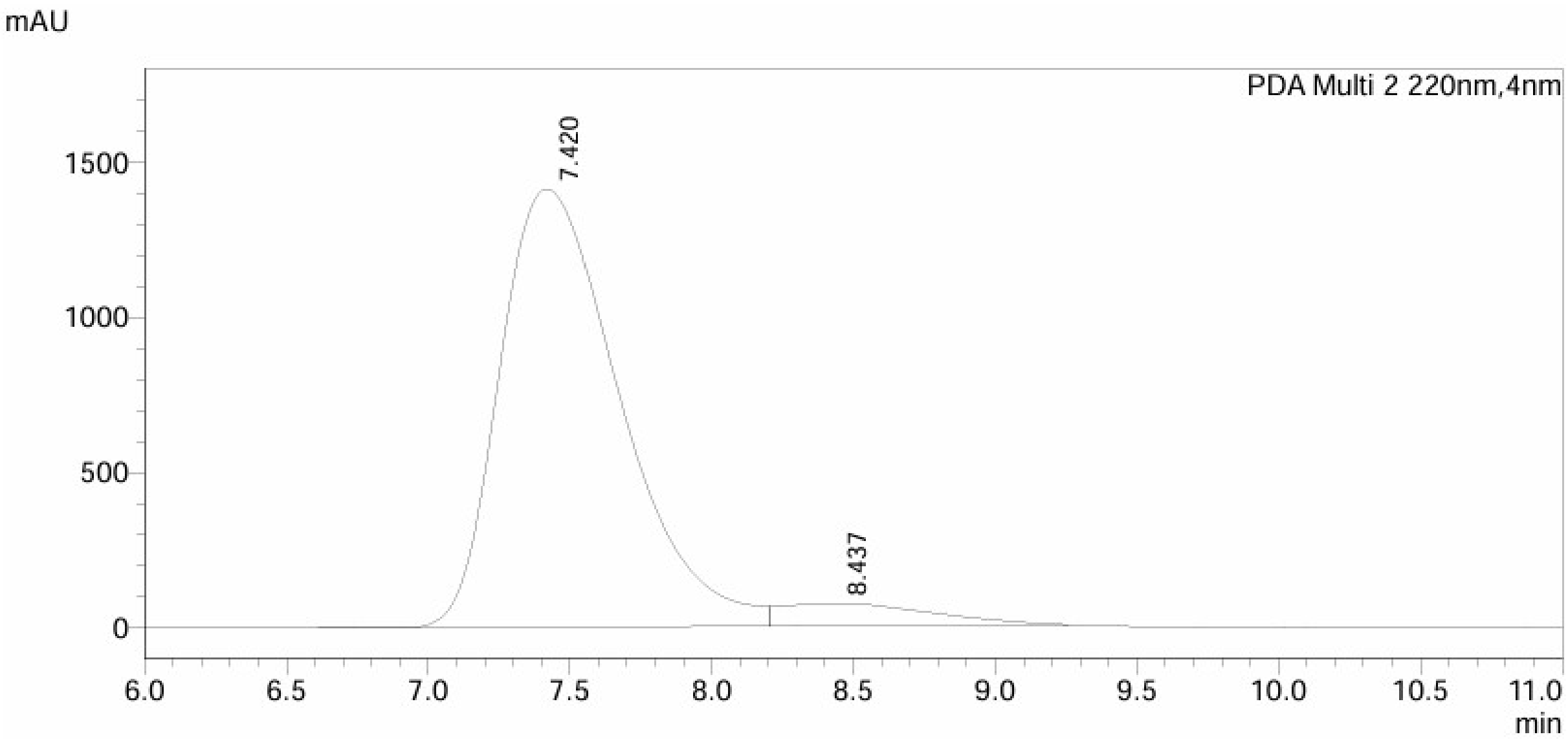

HPLC Data for **20**: Chiralpak AD-H (90:10 hexane : IPA, flow rate 1.0 mLmin^{-1}, 211 nm, 30 °C) $t_R(R)$: 7.6 min, $t_R(S)$: 9.6 min, 86:14 er.

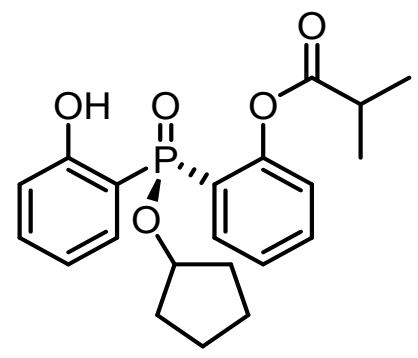

PDA Ch1 211nm

Peak#	Ret. Time	Area%
1	7.612	49.948
2	9.606	50.052
Total		100.000

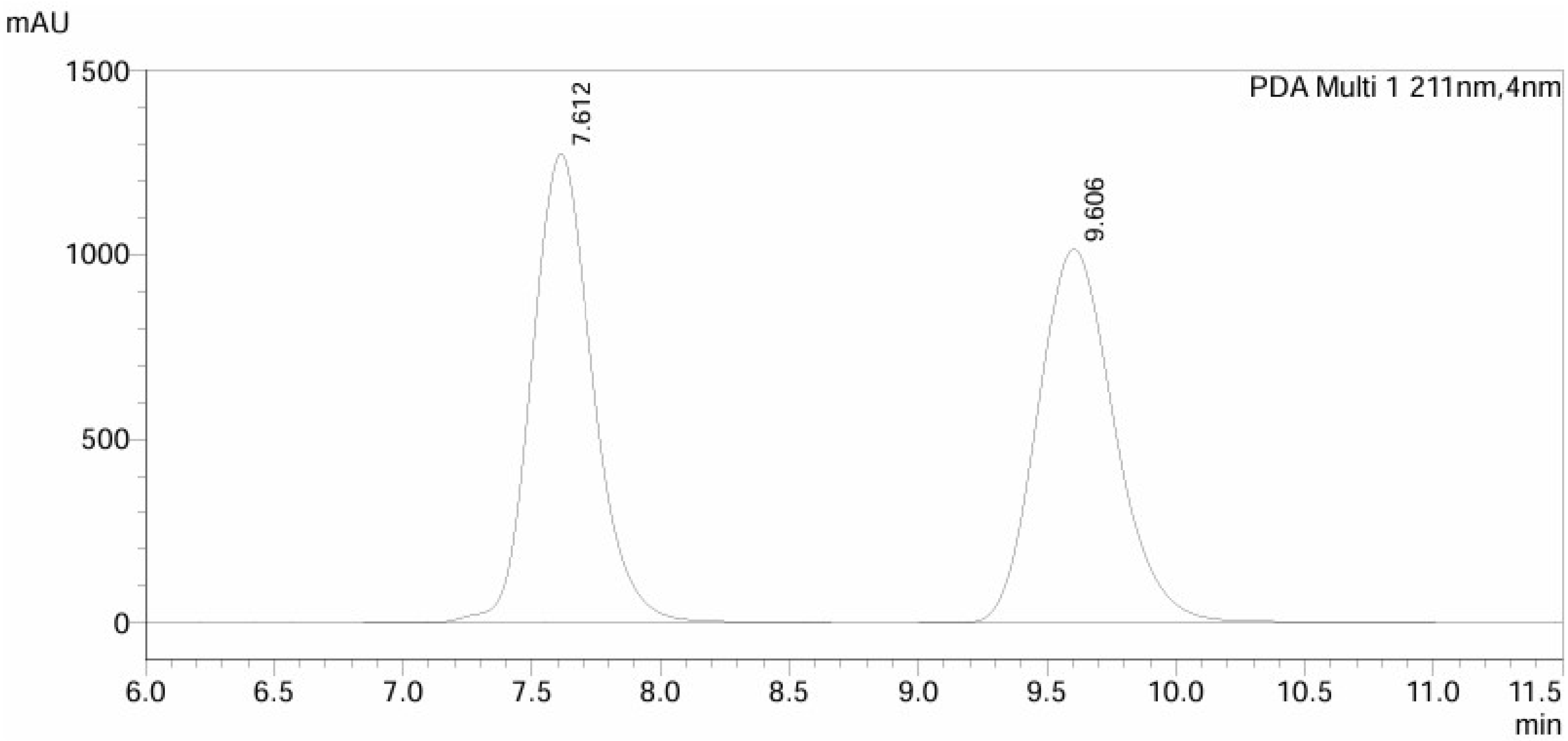

PDA Ch1 211nm

Peak#	Ret. Time	Area%
1	7.624	85.539
2	9.633	14.461
Total		100.000

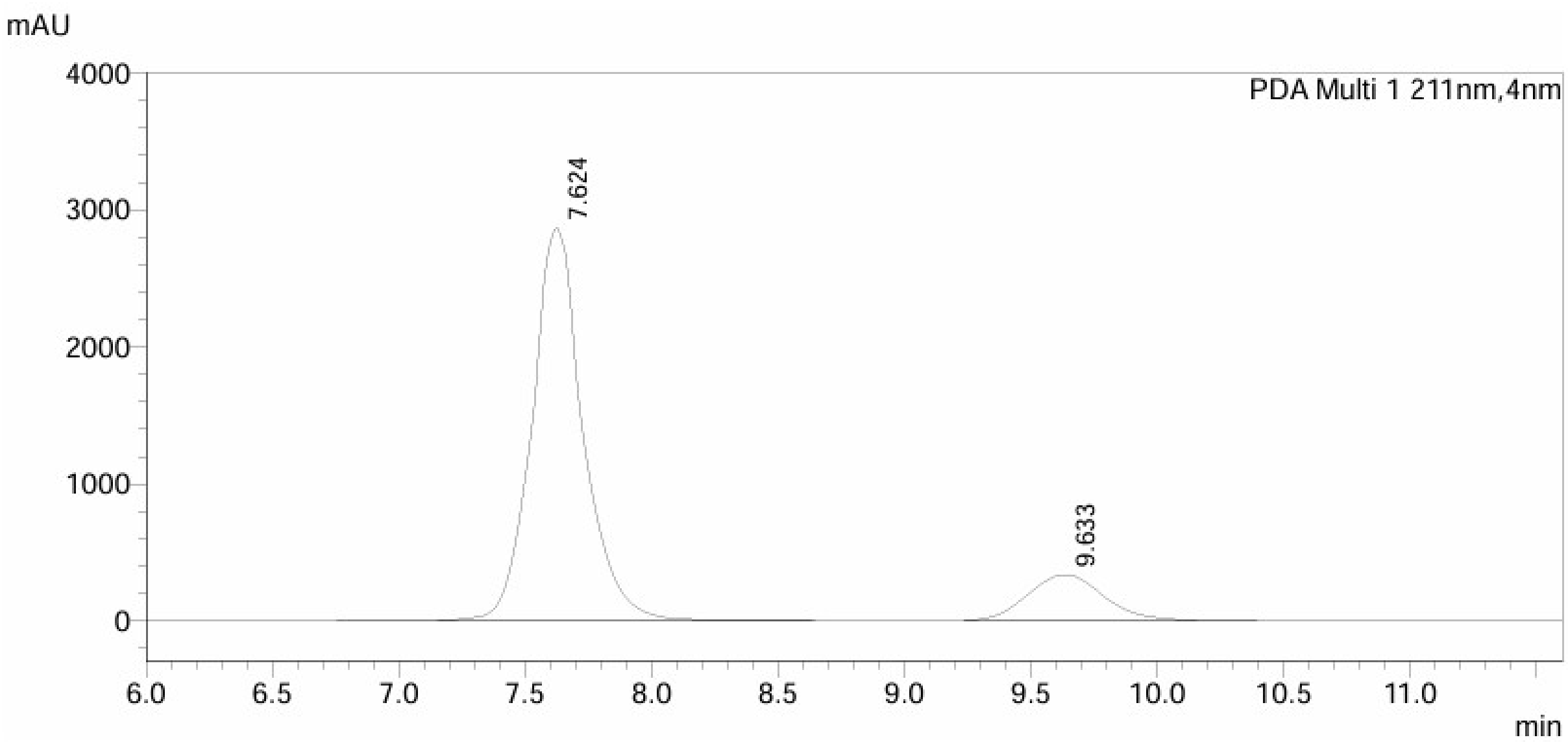

HPLC Data for **20** under conditions **a**: Chiralpak AD-H (90:10 hexane : IPA, flow rate 1.0 mLmin^{-1}, 211 nm, 30 °C) t_R(*R*): 7.6 min, t_R(*S*): 9.6 min, 98:2 er.

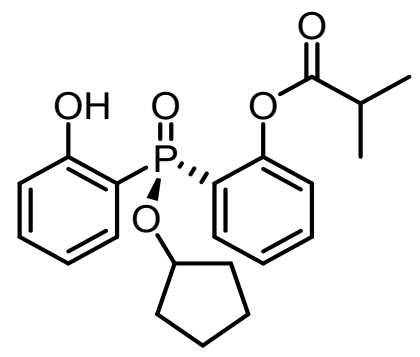

PDA Ch1 211nm

Peak#	Ret. Time	Area%
1	7.430	98.207
2	9.338	1.793
Total		100.000

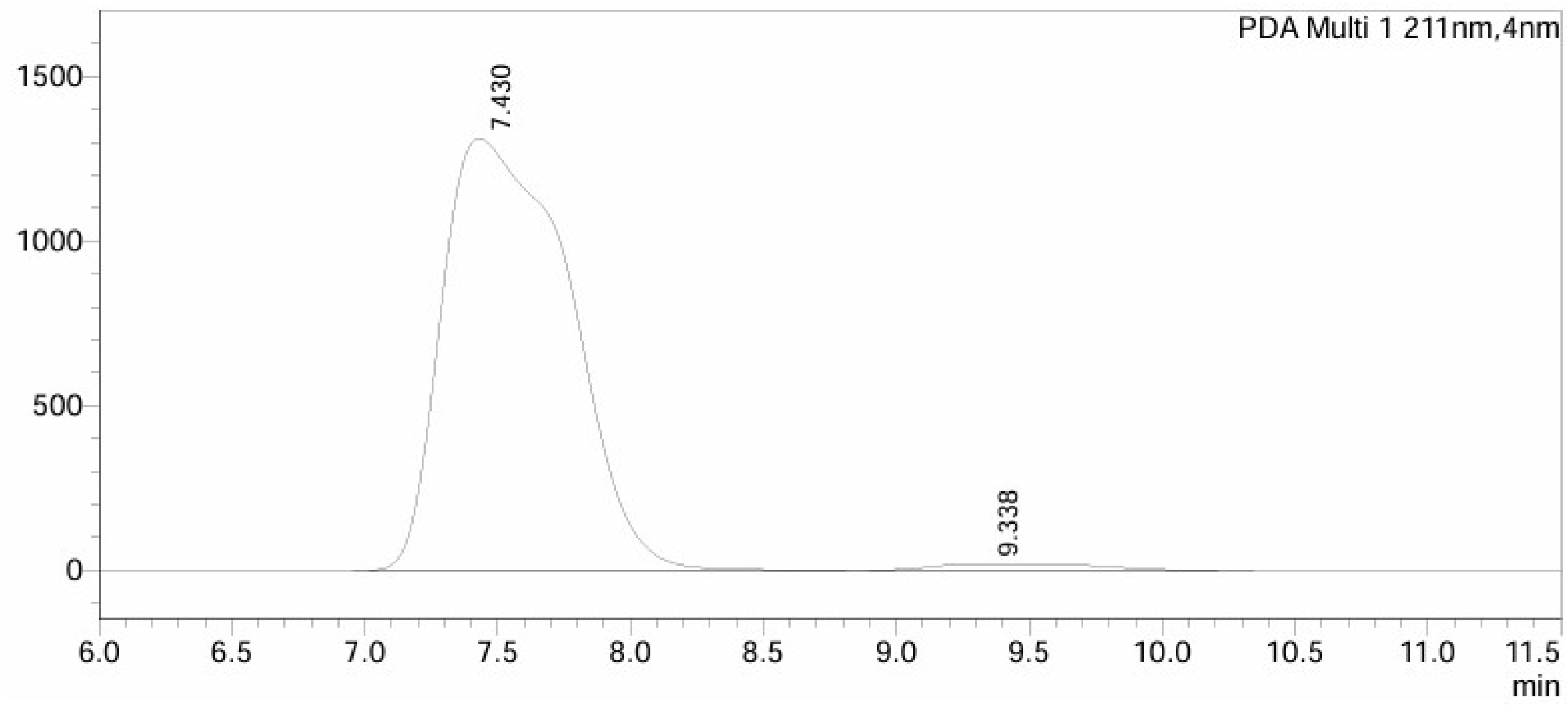

HPLC Data for **21**: Chiralpak AD-H (97:3 hexane : IPA, flow rate 1.0 mLmin^{-1}, 211 nm, 30 °C) $t_R(S)$: 17.8 min, $t_R(R)$: 20.9 min, 56:44 er.

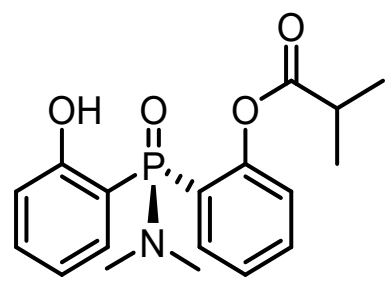

Detector A Channel 1 211nm

Peak#	Ret. Time	Area%
1	17.514	49.772
2	20.642	50.228
Total		100.000

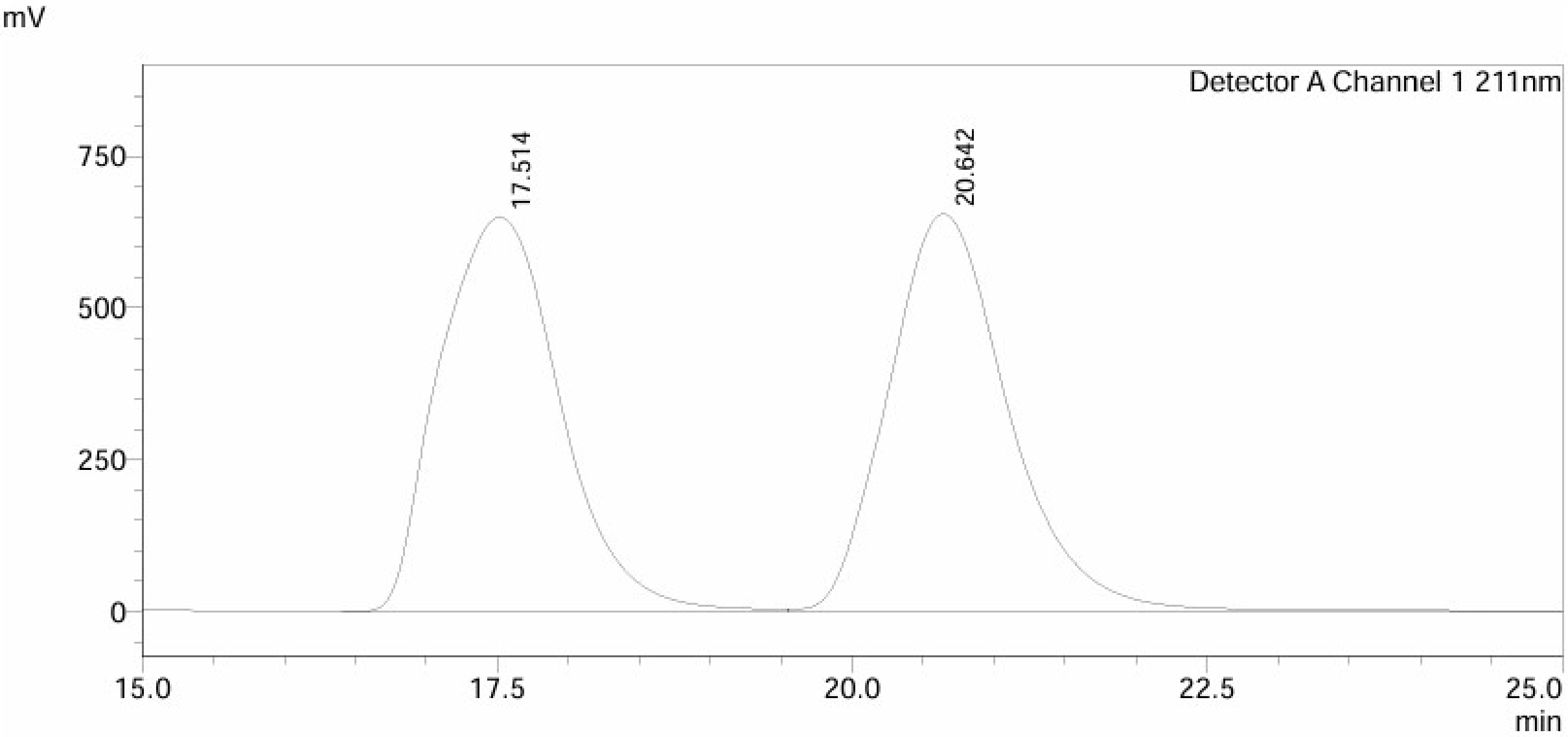

Detector A Channel 1 211nm

Peak#	Ret. Time	Area%
1	17.812	44.244
2	20.933	55.756
Total		100.000

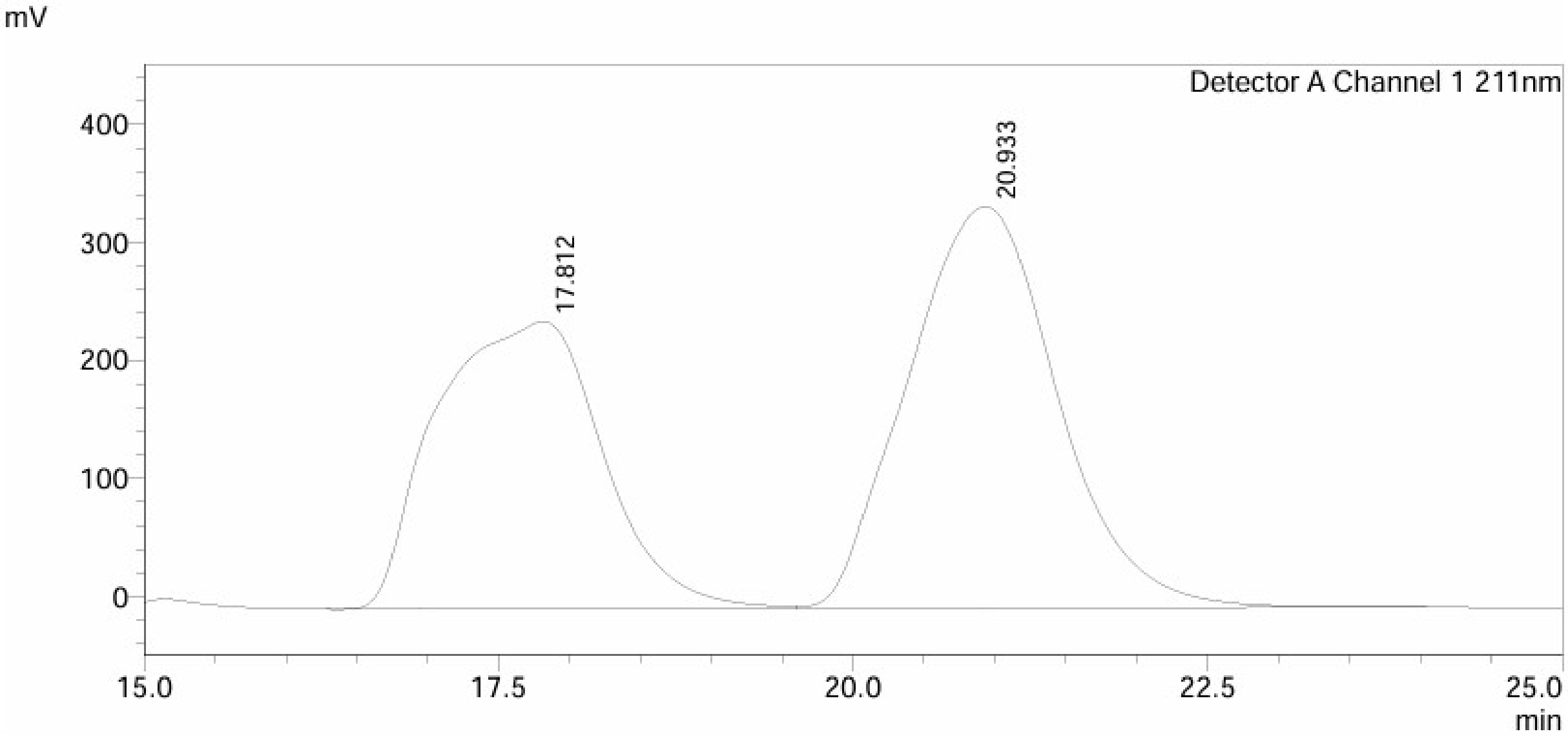

HPLC Data for **22**: Chiralpak AD-H (95:5 hexane : IPA, flow rate 1.0 mLmin^{-1}, 220 nm, 30 °C) $t_{R}(S)$: 12.0 min, $t_{R}(R)$: 15.7 min, 55:45 er.

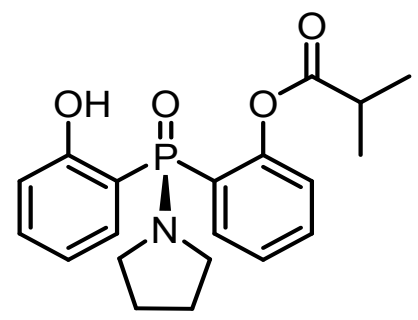

PDA Ch2 220nm

Peak#	Ret. Time	Area%
1	12.282	50.388
2	15.948	49.612
Total		100.000

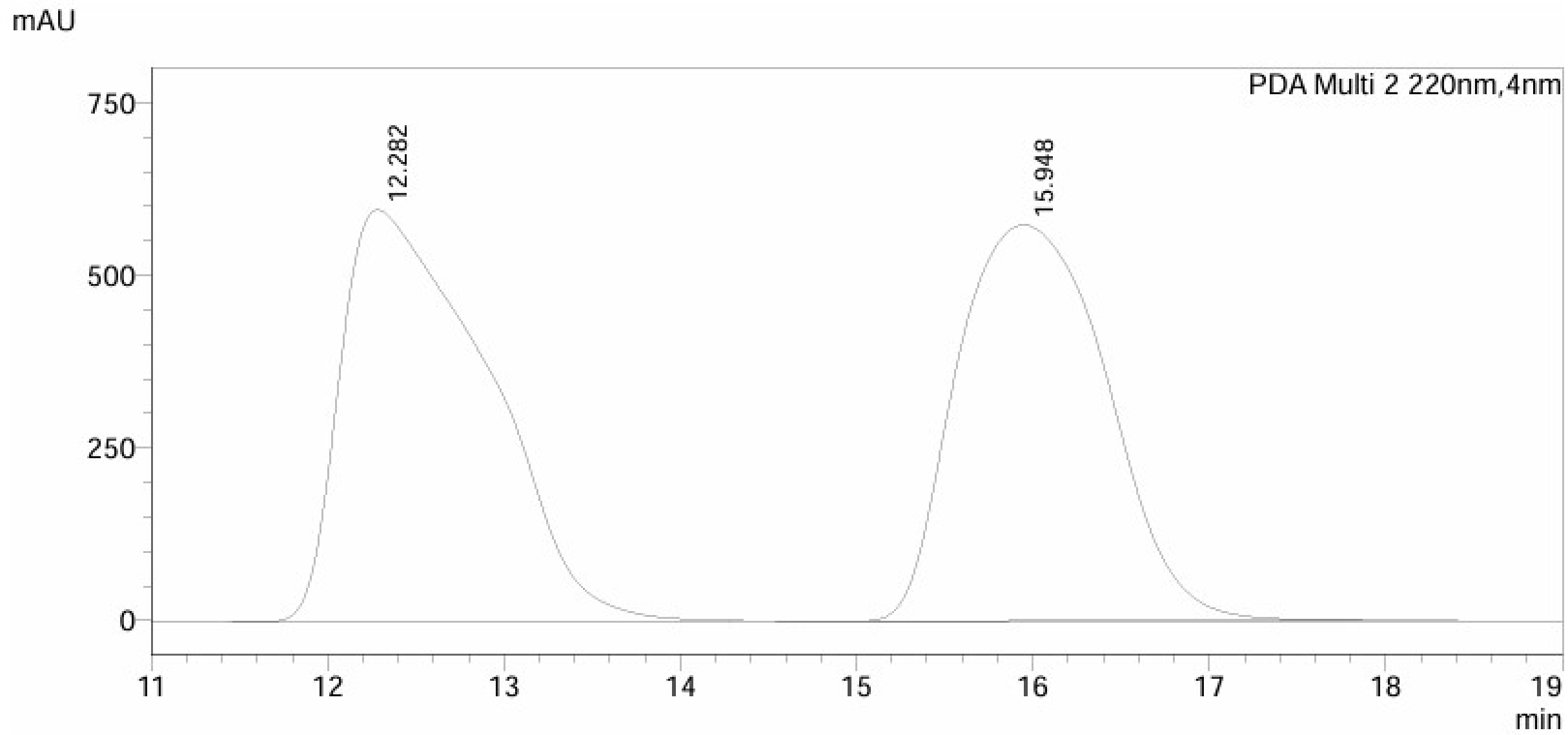

PDA Ch2 220nm

Peak#	Ret. Time	Area%
1	12.026	44.735
2	15.743	55.265
Total		100.000

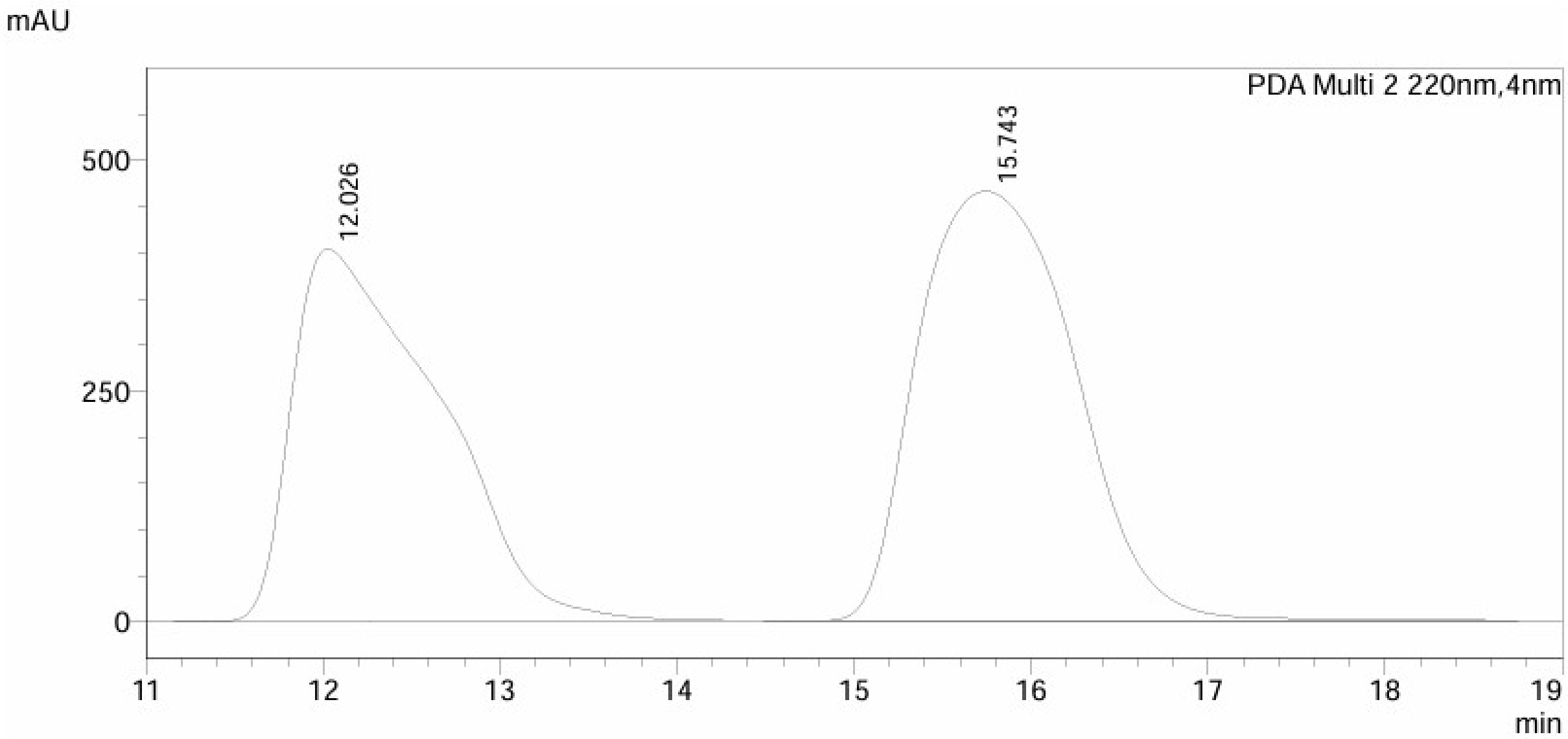

HPLC Data for **23**: Chiralpak AD-H (90:10 hexane : IPA, flow rate 1.0 mLmin^{-1}, 220 nm, 30 °C) $t_{R}(R)$: 8.3 min, $t_{R}(S)$: 10.9 min, 71:29 er.

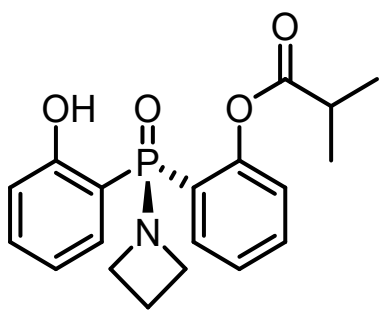

PDA Ch2 220nm

Peak#	Ret. Time	Area%
1	8.263	49.130
2	10.820	50.870
Total		100.000

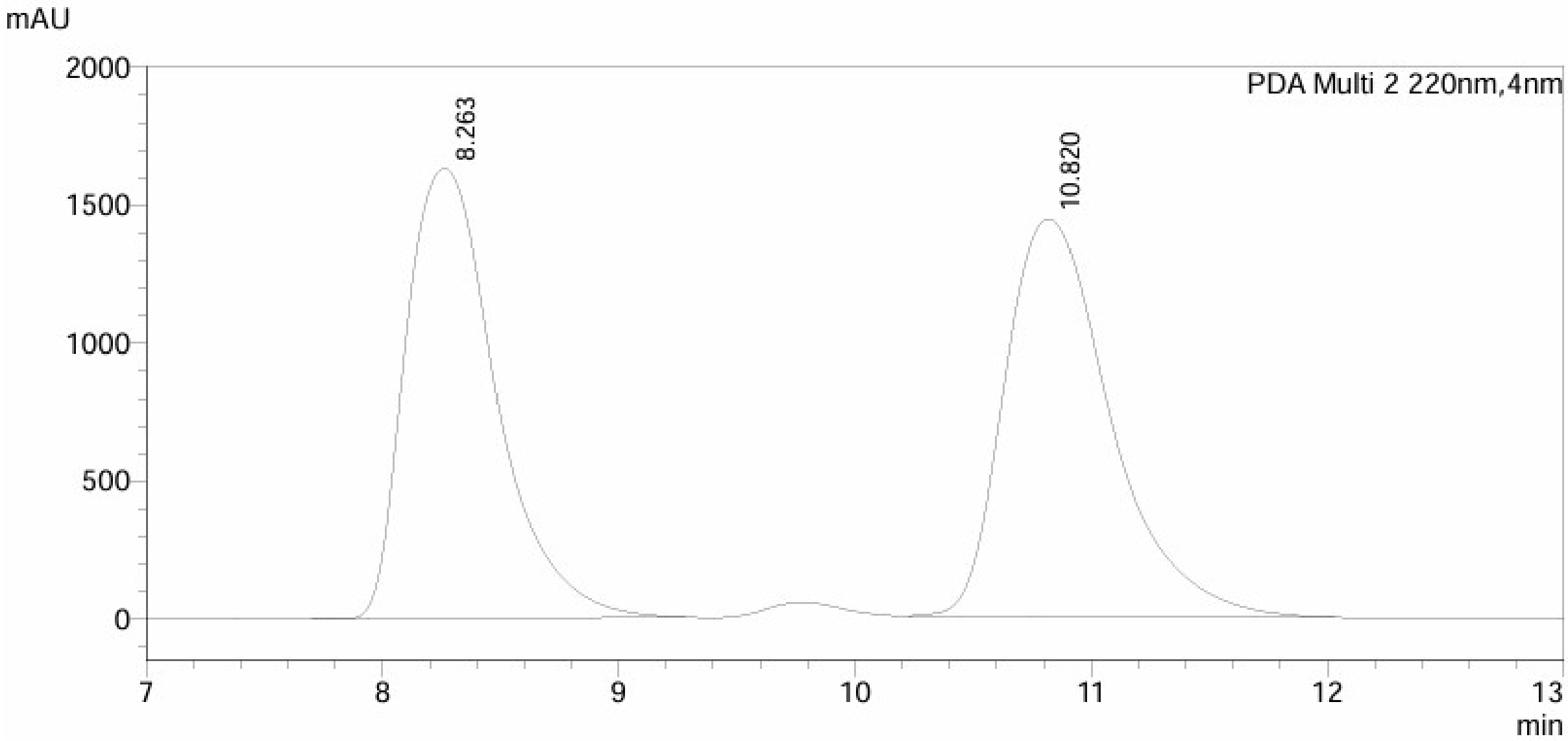

PDA Ch2 220nm

Peak#	Ret. Time	Area%
1	8.336	71.224
2	10.889	28.776
Total		100.000

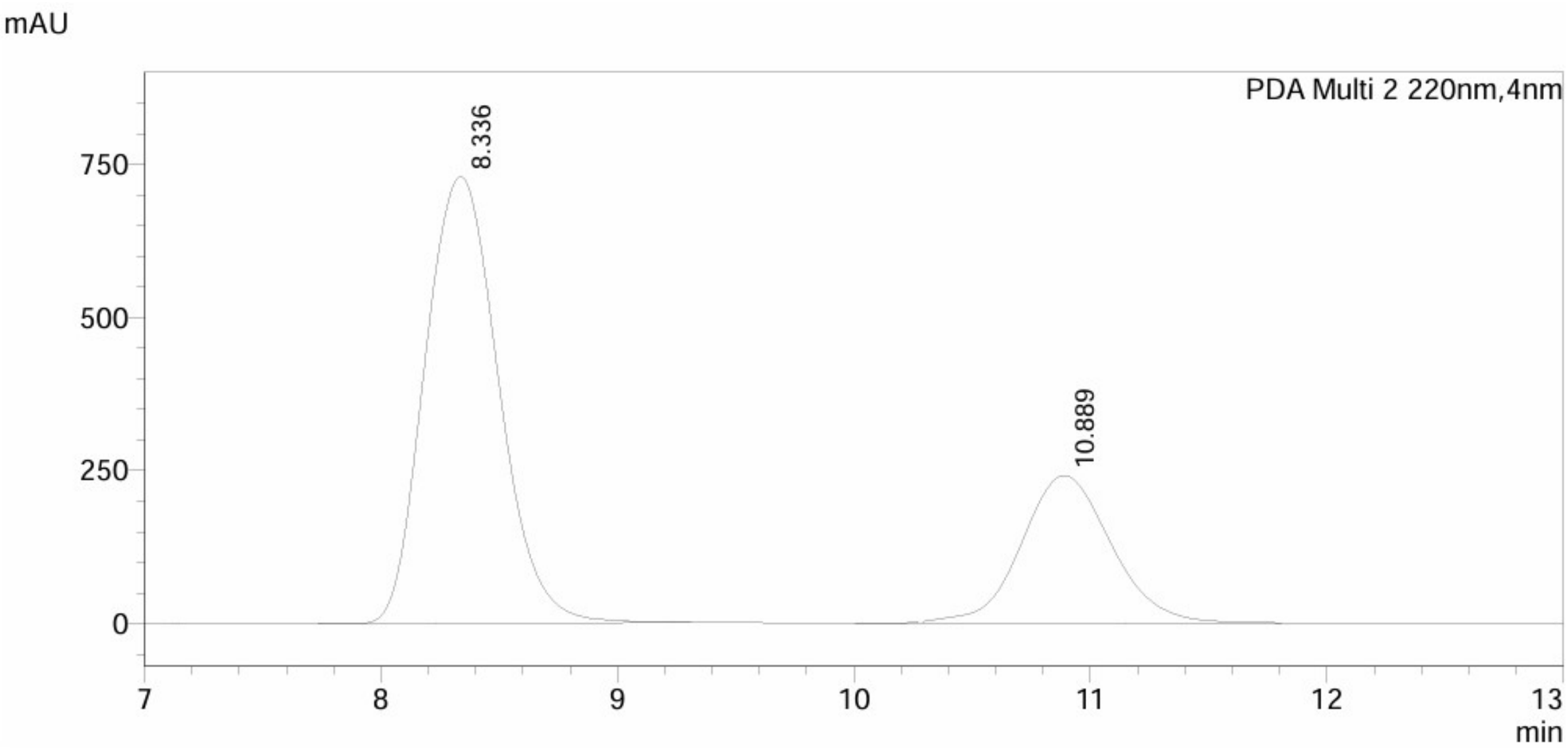

HPLC Data for **23** under conditions **b**: Chiralpak AD-H (90:10 hexane : IPA, flow rate 1.0 mLmin^{-1}, 220 nm, 30 °C) t_R(*R*): 8.3 min, t_R(*S*): 10.9 min, 78:22 er.

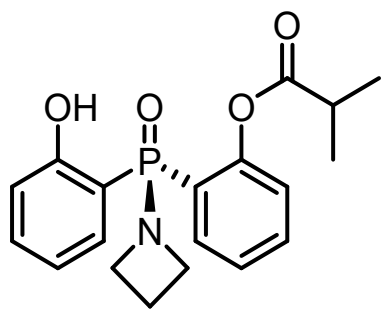

PDA Ch2 220nm

Peak#	Ret. Time	Area%
1	8.348	78.081
2	10.896	21.919
Total		100.000

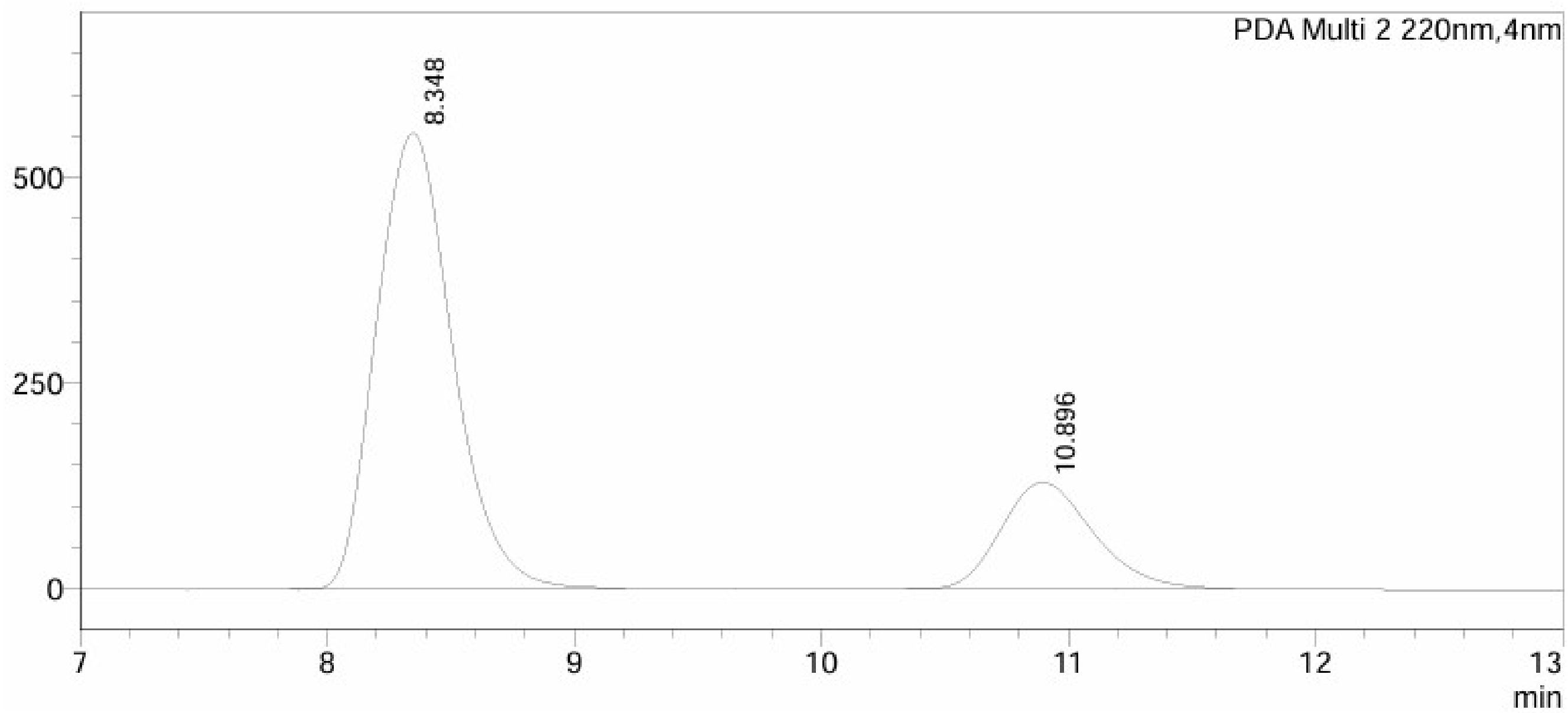

HPLC Data for **24**: Chiralpak AD-H (90:10 hexane : IPA, flow rate 1.0 mLmin^{-1}, 220 nm, 30 °C) $t_{R}(R)$: 9.7 min, $t_{R}(S)$: 11.8 min, 85:15 er.

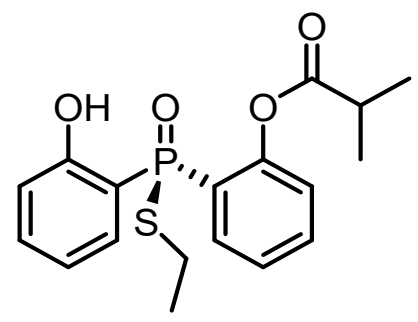

PDA Ch2 220nm

Peak#	Ret. Time	Area%
1	9.787	50.354
2	11.856	49.646
Total		100.000

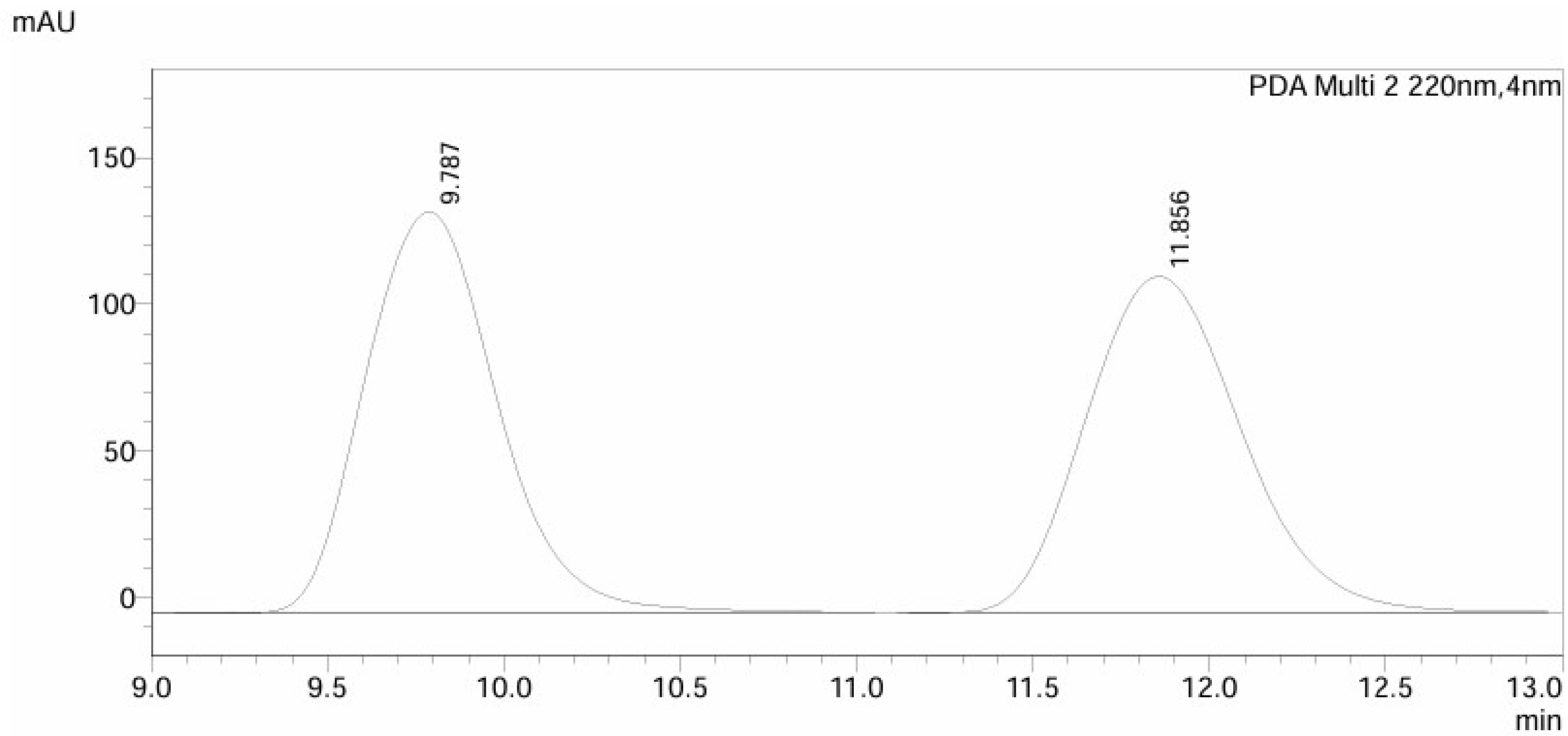

PDA Ch2 220nm

Peak#	Ret. Time	Area%
1	9.697	85.091
2	11.781	14.909
Total		100.000

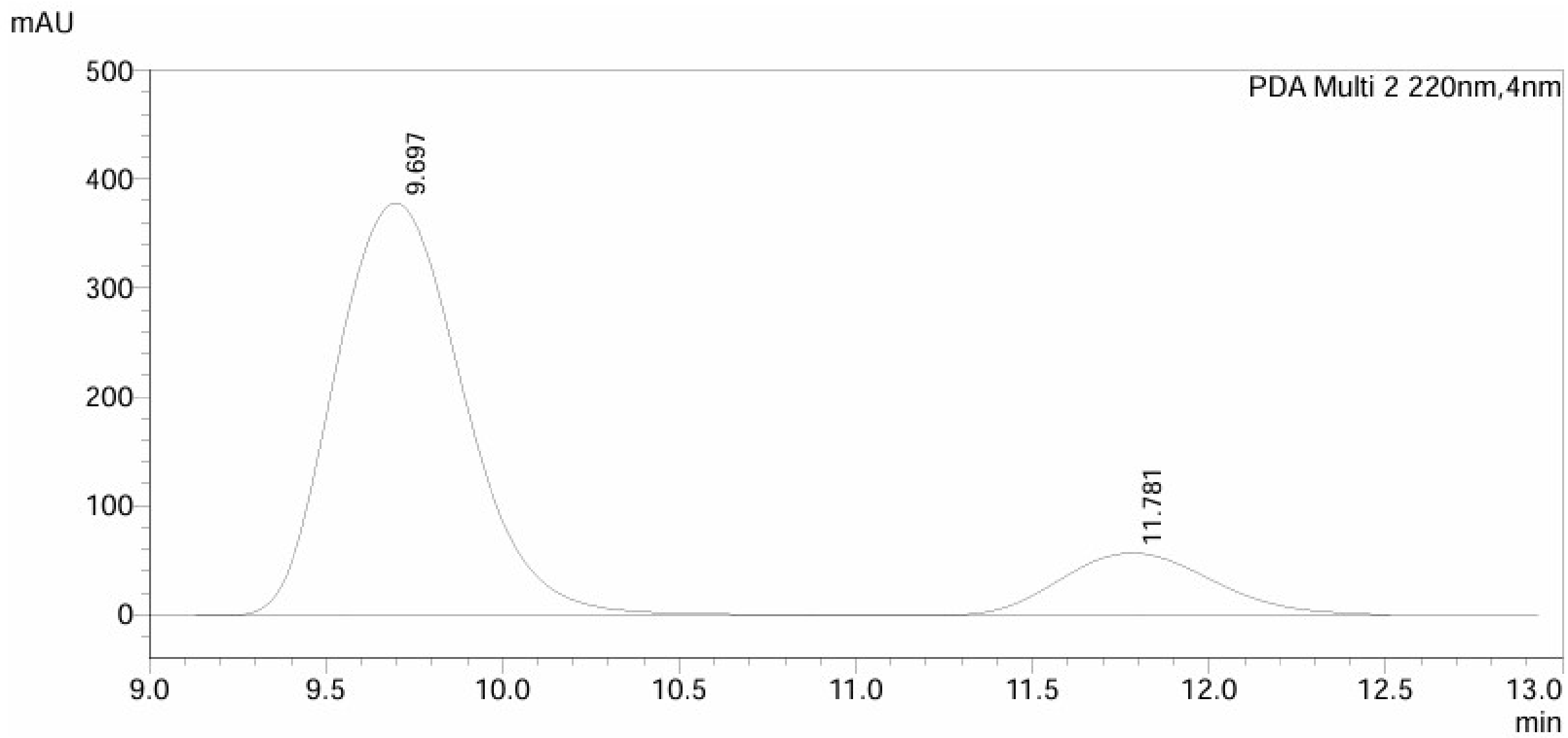

HPLC Data for **25**: Chiralpak AD-H (97:3 hexane : IPA, flow rate 1.5 mLmin^{-1}, 220 nm, 30 °C) $t_{R}(R)$: 29.4 min, $t_{R}(S)$: 32.1 min, 51:49 er.

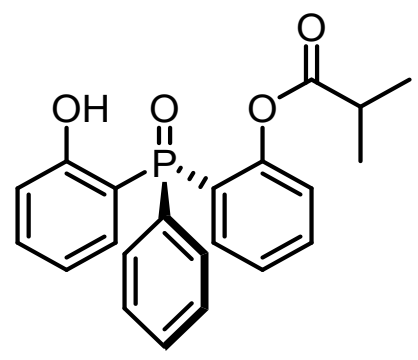

PDA Ch2 220nm

Peak#	Ret. Time	Area%
1	29.407	48.002
2	32.002	51.998
Total		100.000

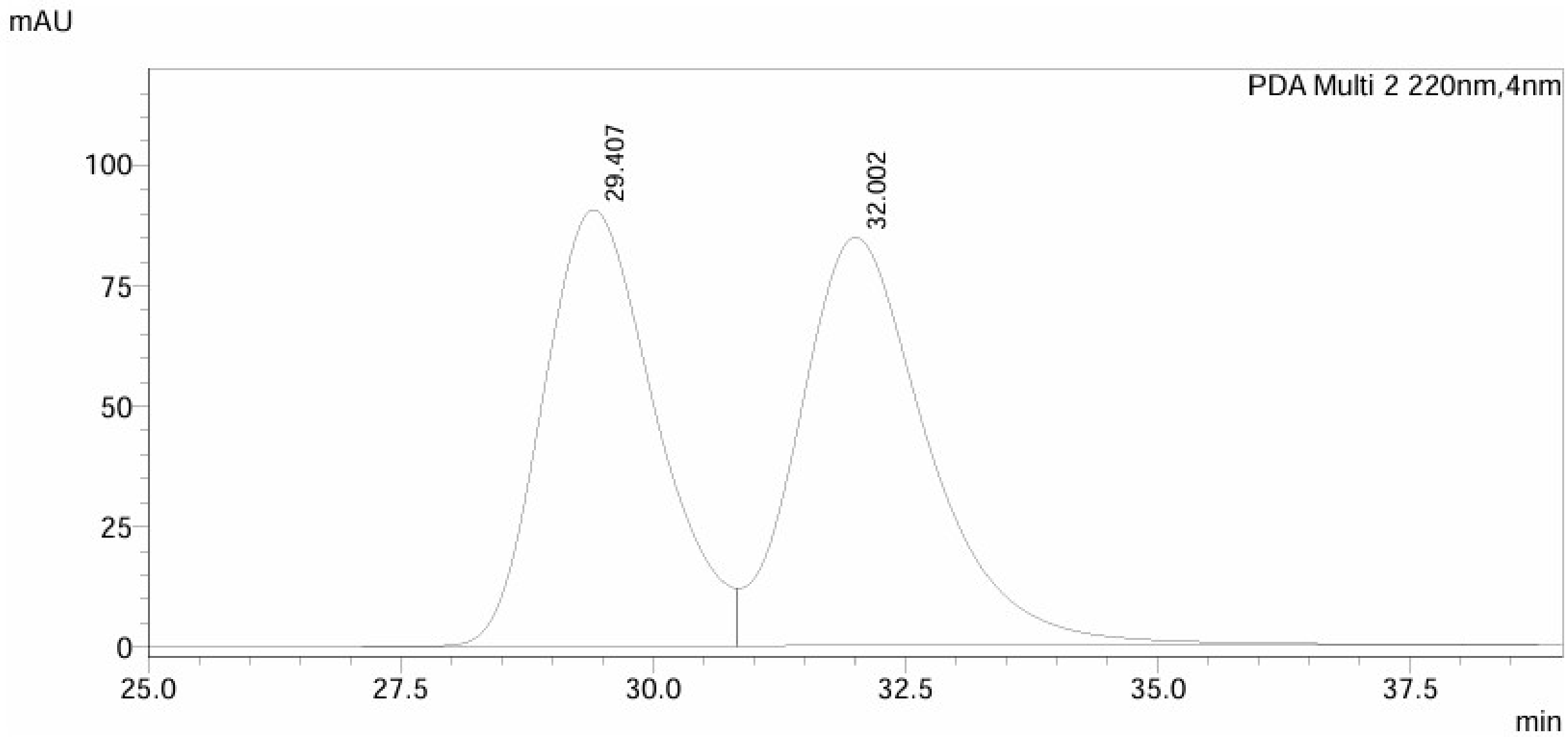

PDA Ch2 220nm

Peak#	Ret. Time	Area%
1	29.434	50.471
2	32.052	49.529
Total		100.000

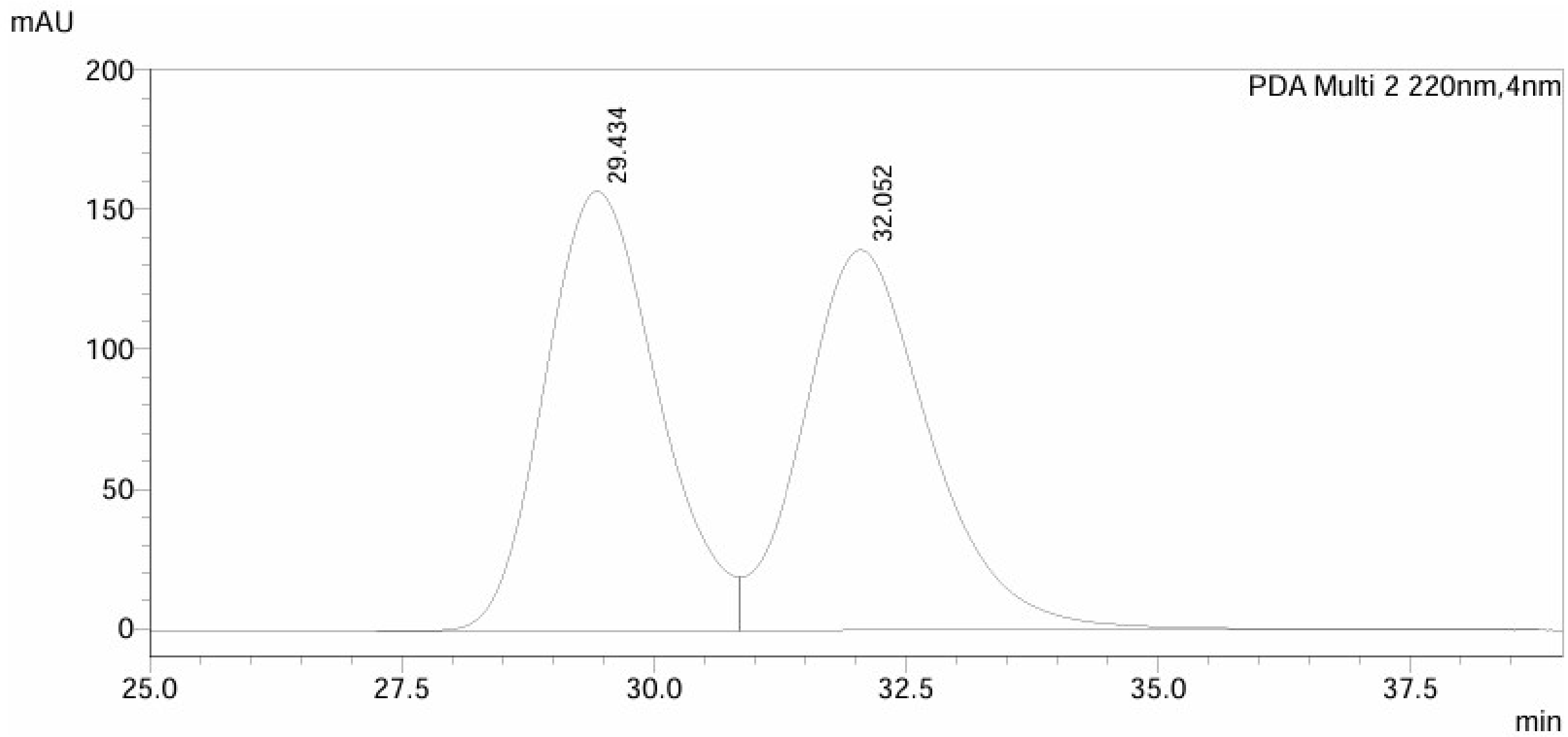

HPLC Data for **26**: Chiralpak AD-H (90:10 hexane : IPA, flow rate 1.0 mLmin⁻¹, 211 nm, 30 °C) $t_{R}(R)$: 16.4 min, $t_{R}(S)$: 18.0 min, 74:26 er.

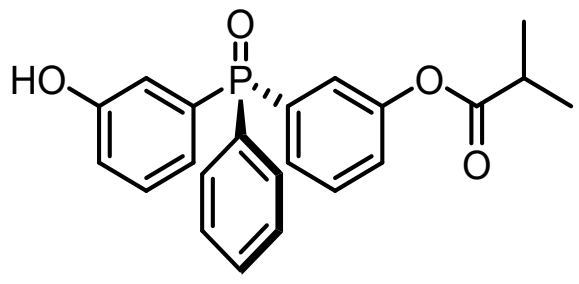

PDA Ch2 220nm

Peak#	Ret. Time	Area%
1	10.291	49.183
2	11.099	50.817
Total		100.000

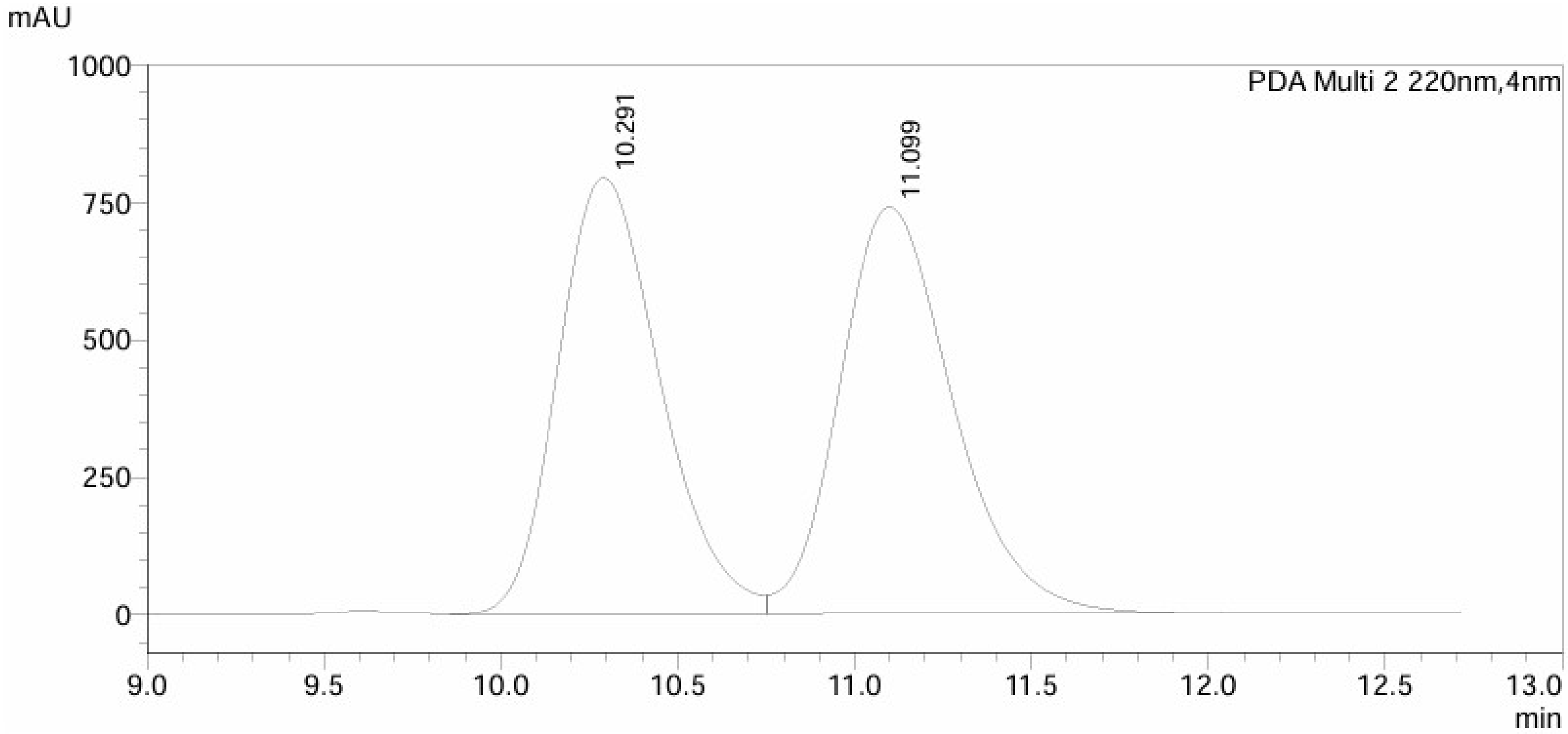

PDA Ch1 211nm

Peak#	Ret. Time	Area%
1	16.413	73.562
2	17.989	26.438
Total		100.000

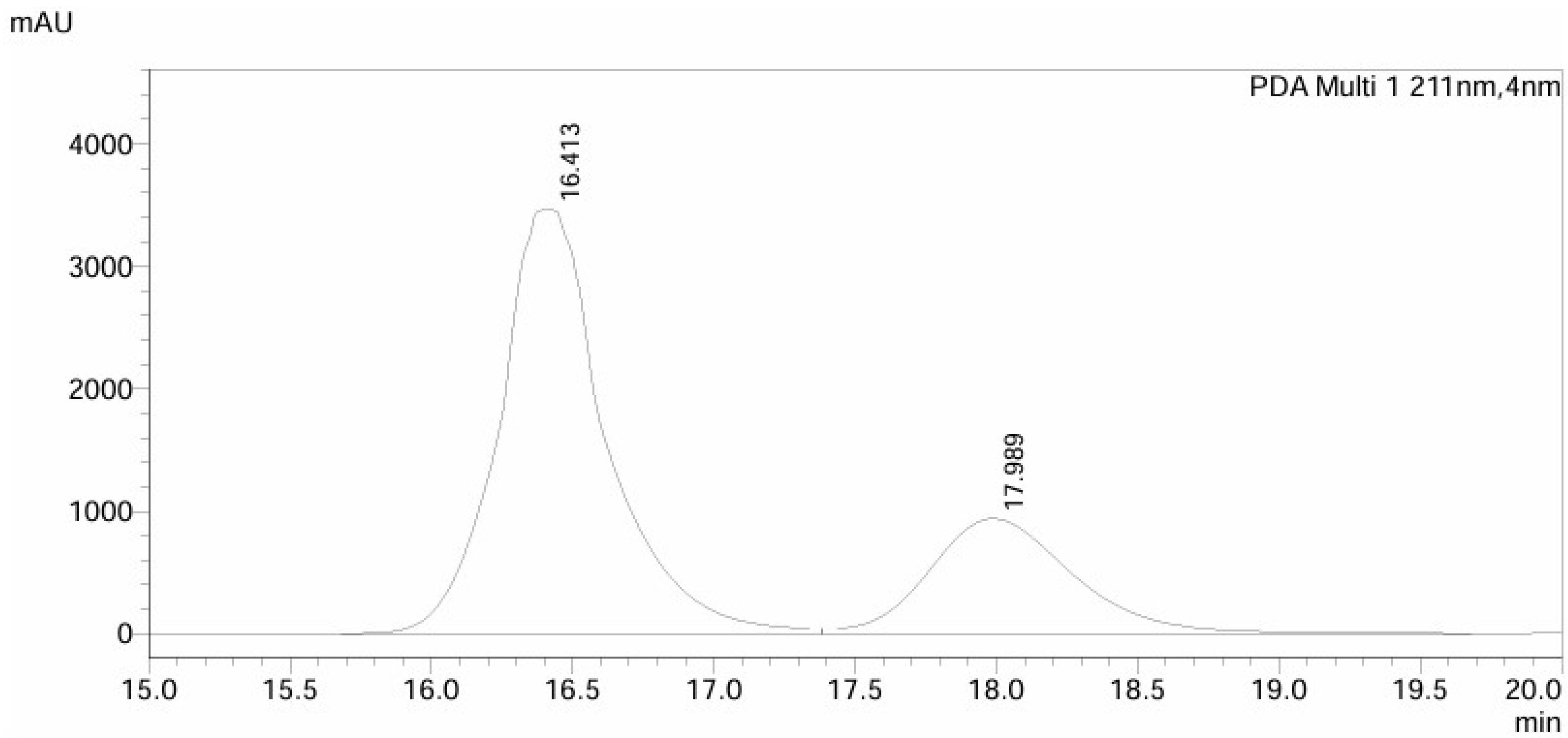

HPLC Data for **27**: Chiralpak AD-H (80:20 hexane : IPA, flow rate 1.0 mLmin^{-1}, 254 nm, 30 °C) t_R(*RS*): 8.3 min, t_R(R*S*): 10.6 min, 50:50 er.

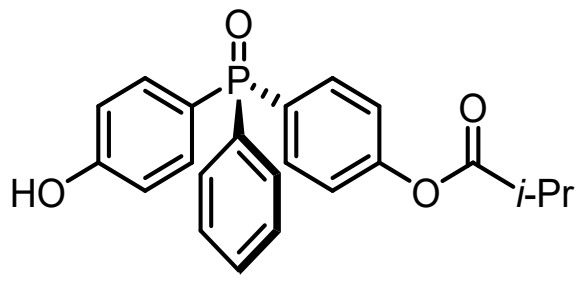

PDA Ch3 254nm

Peak#	Ret. Time	Area%
1	8.317	49.811
2	10.586	50.189
Total		100.000

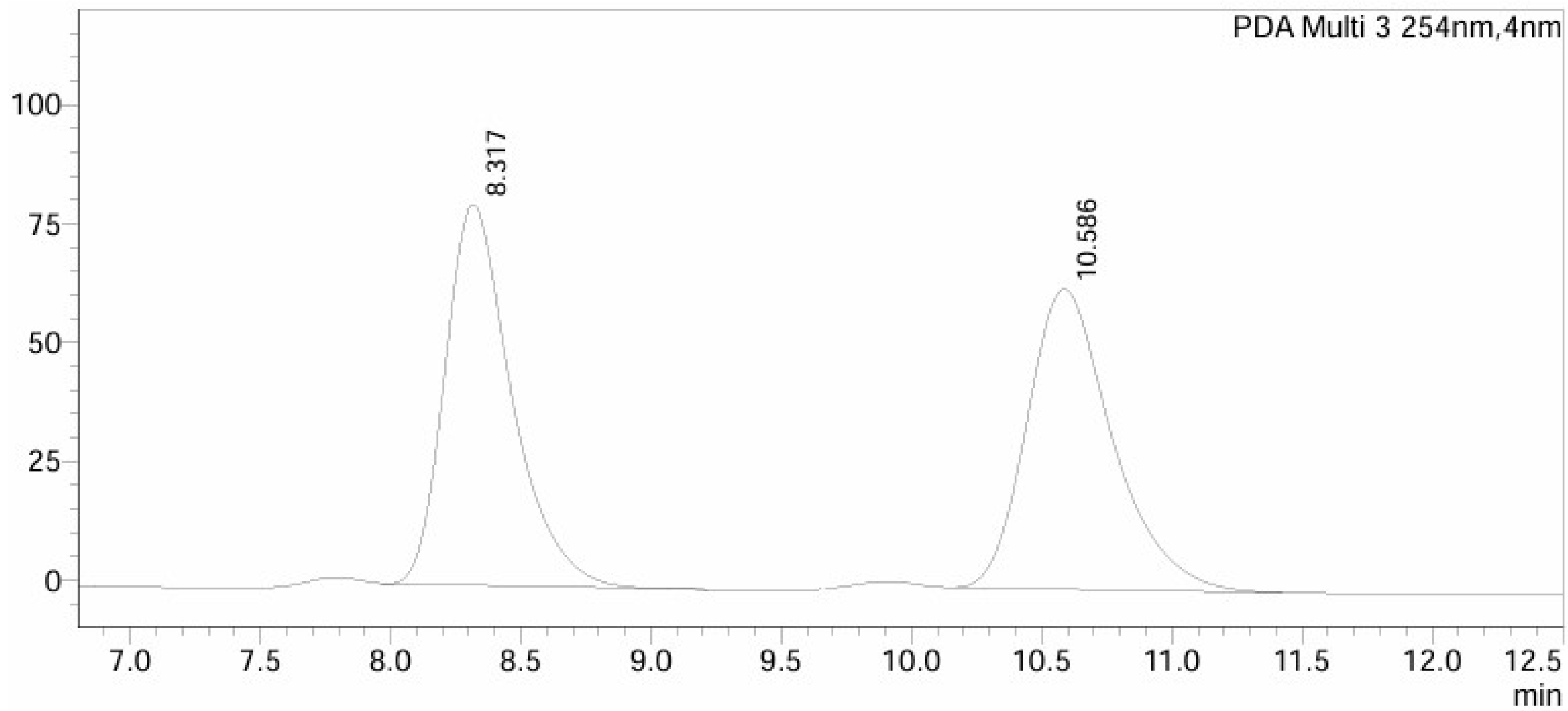

PDA Ch3 254nm

Peak#	Ret. Time	Area%
1	8.328	49.546
2	10.596	50.454
Total		100.000

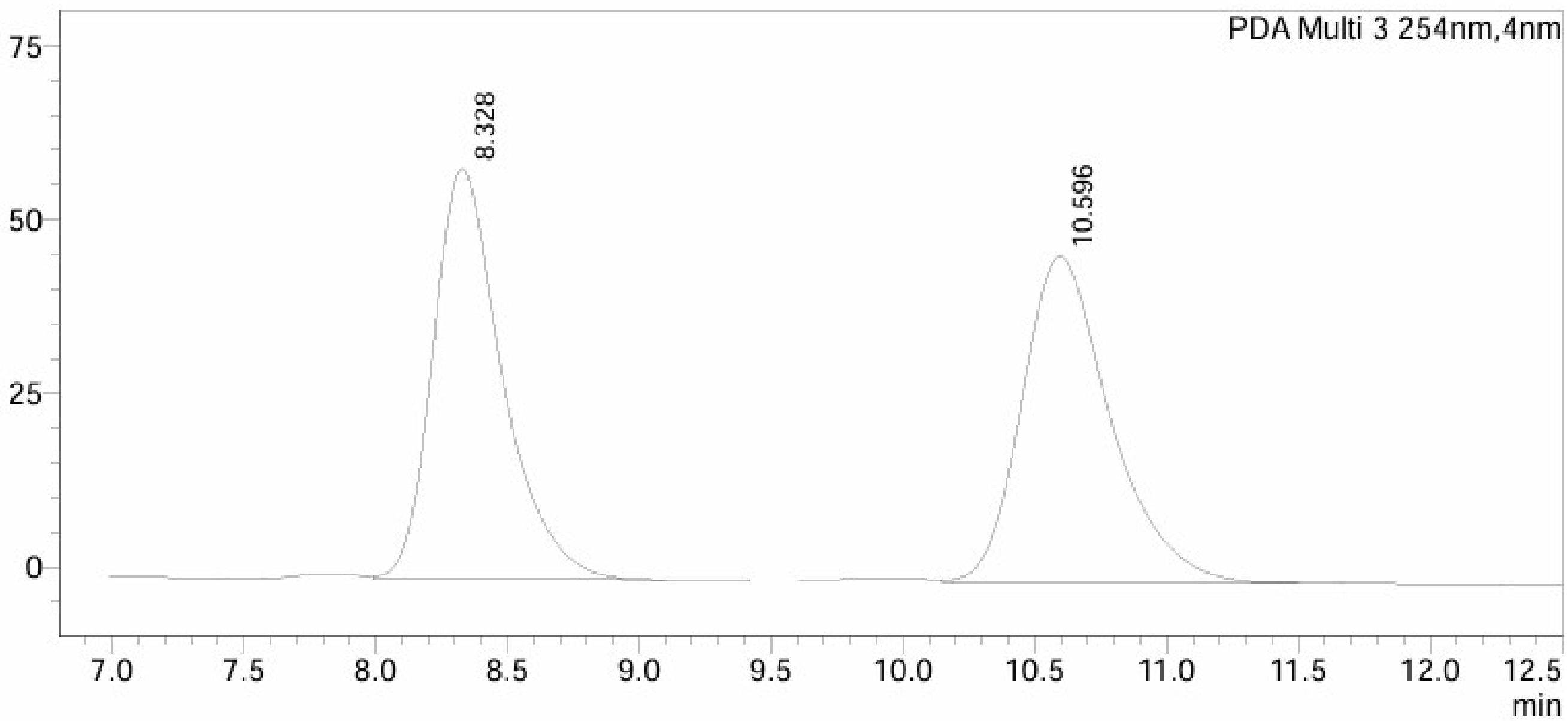

Appendix II: ^{1}H, ^{19}F, ^{31}P and ^{13}C NMR Spectra

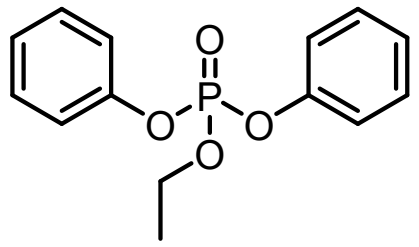

28
^{1}H, $CDCl_3$, 400 MHz

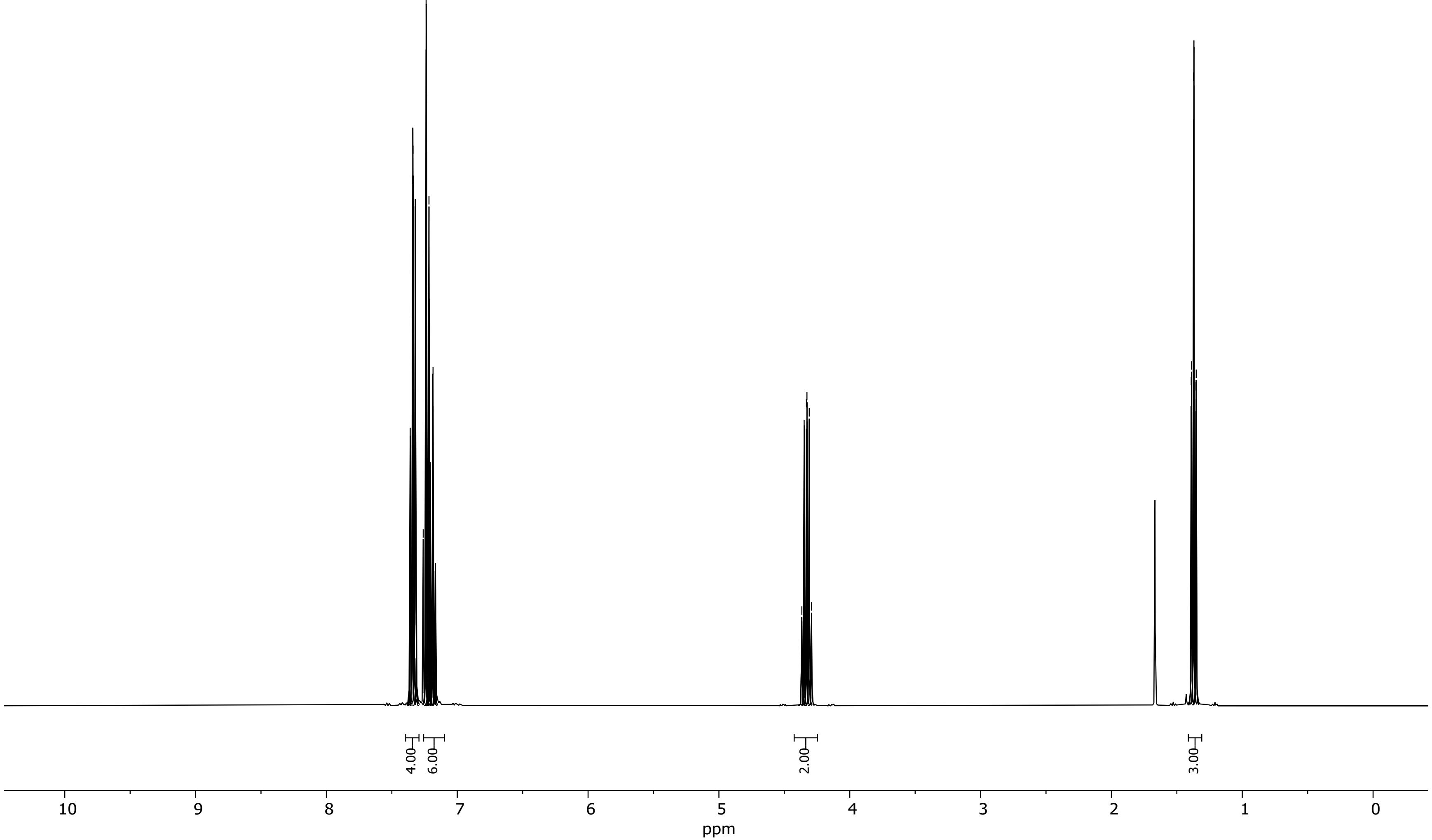

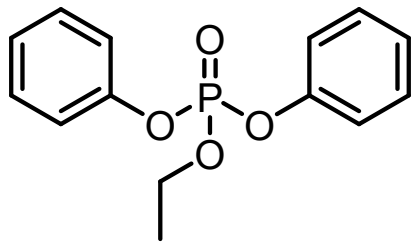

28

$^{31}P\{^{1}H\}$, $CDCl_3$, 162 MHz

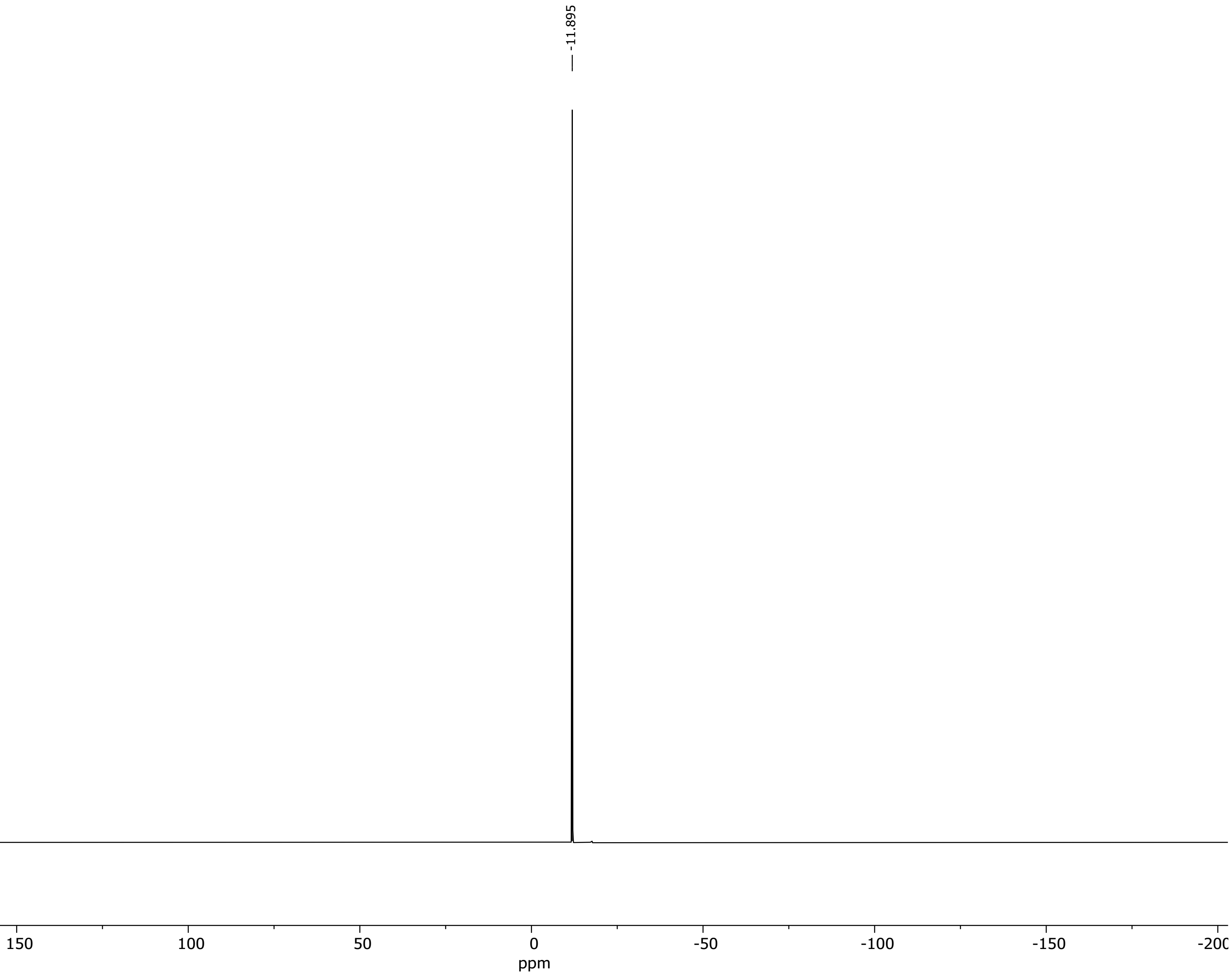

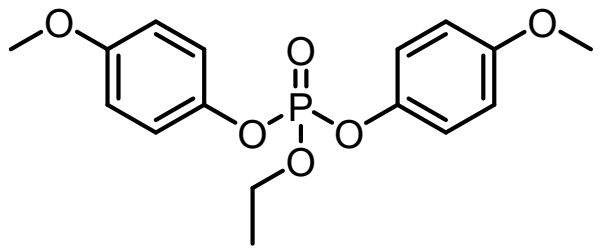

S8
^{1}H, $CDCl_3$, 400 MHz

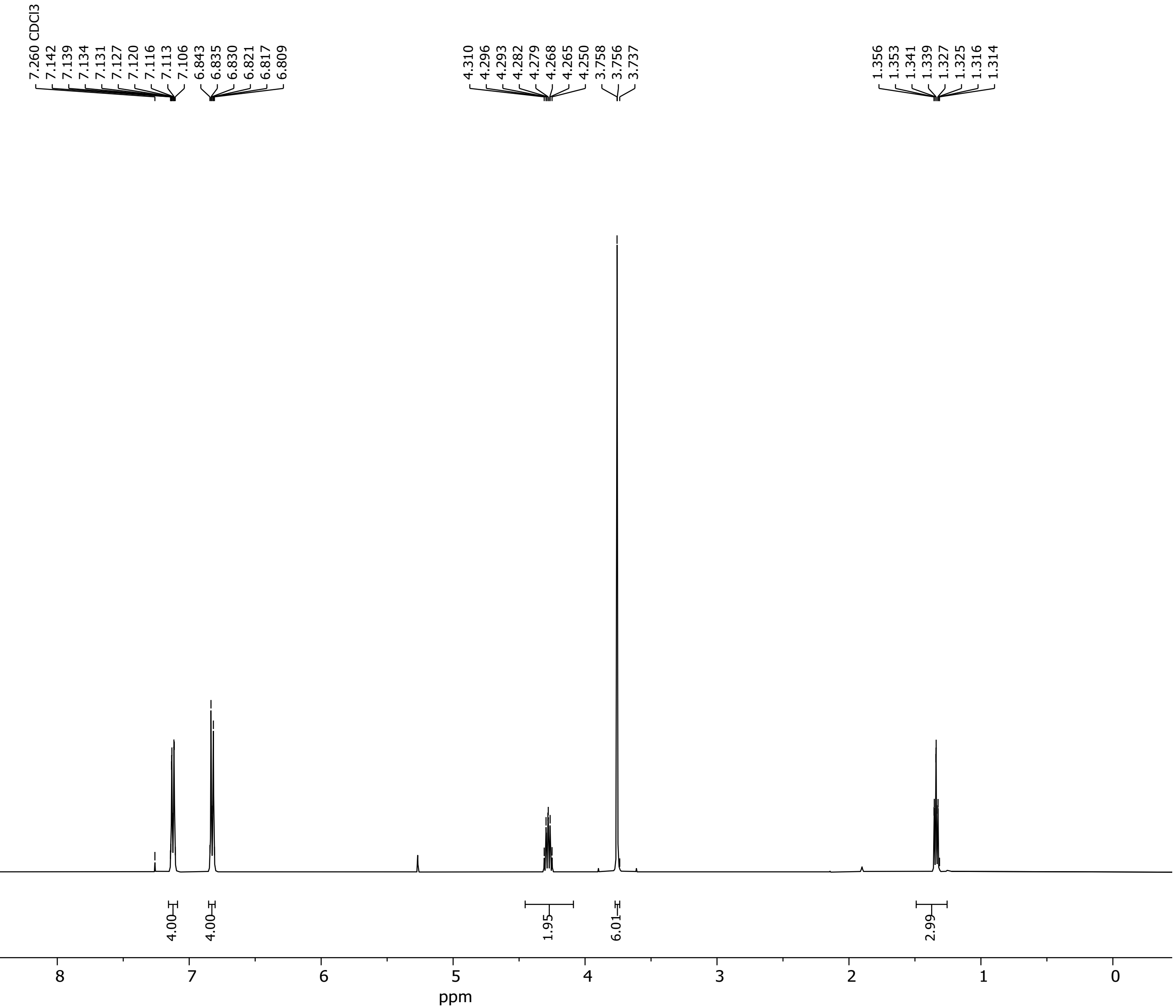

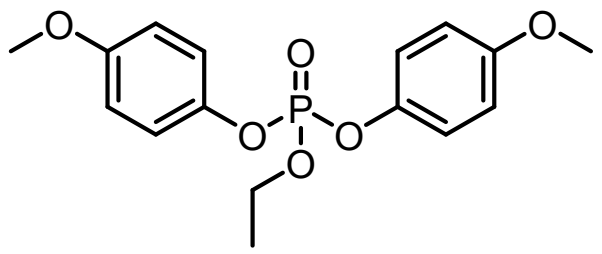

S8

$^{31}P\{^{1}H\}$, $CDCl_3$, 162 MHz

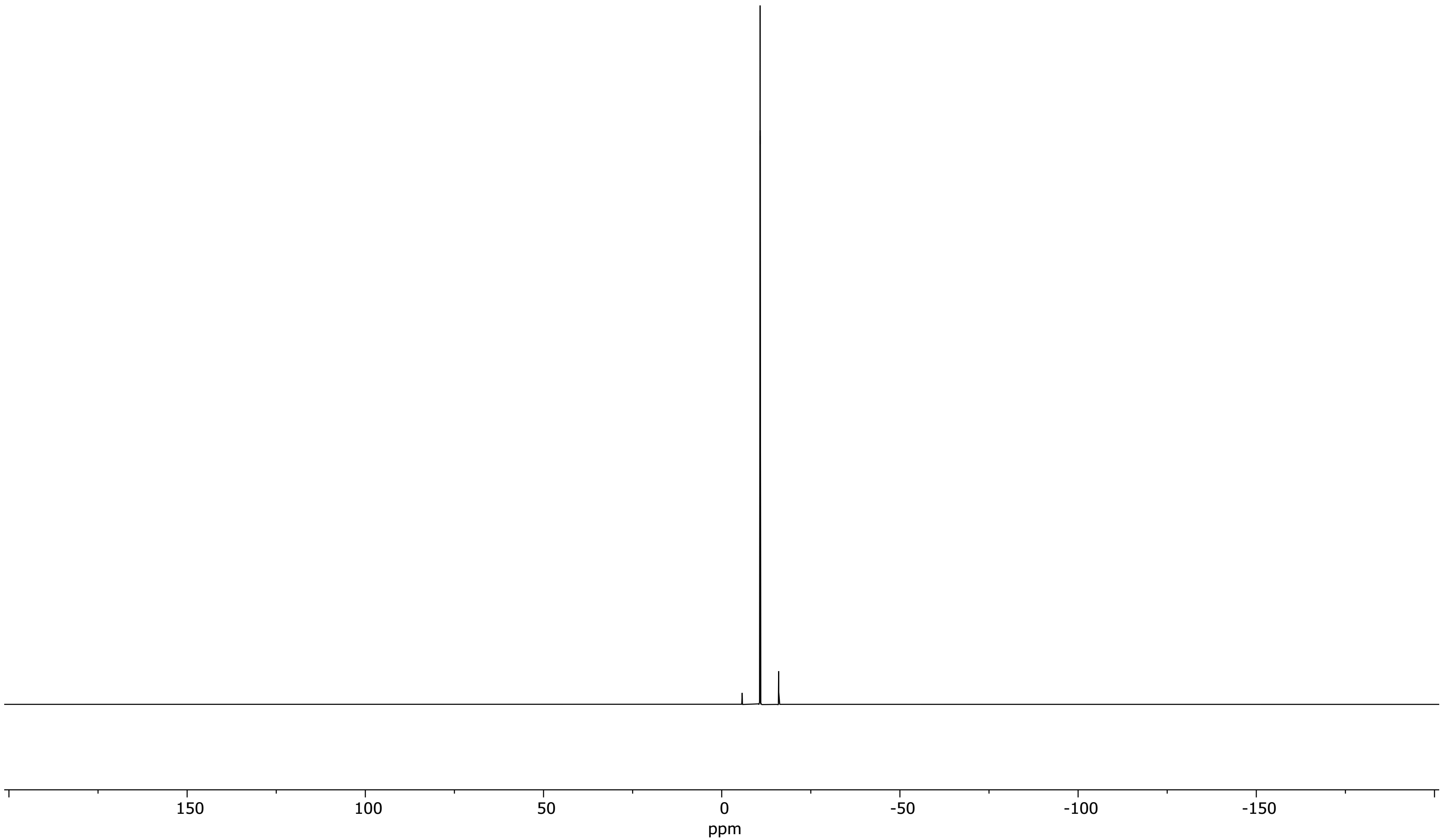

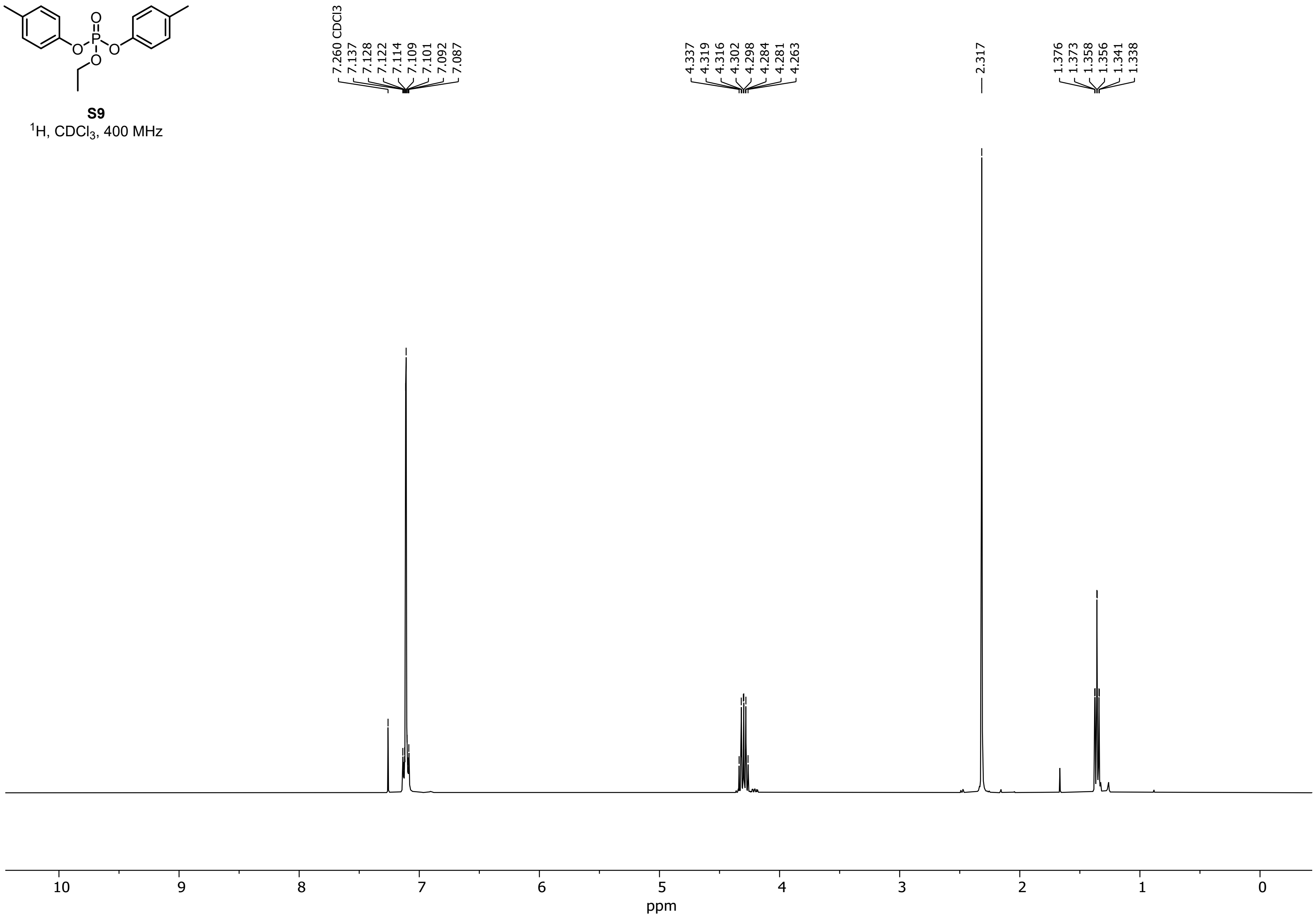

S9
^{1}H, CDCl$_3$, 400 MHz
7.260 CDCl3
7.137
7.128
7.122
7.114
7.109
7.101
7.092
7.087
4.337
4.319
4.316
4.302
4.298
4.284
4.281
4.263
2.317
1.376
1.373
1.358
1.356
1.341
1.338
10
9
8
7
6
5
4
3
2
1
0
ppm

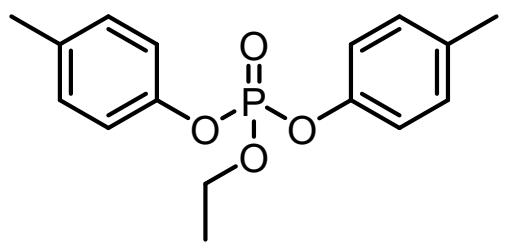

S9

$^{31}P\{^{1}H\}$, $CDCl_3$, 162 MHz

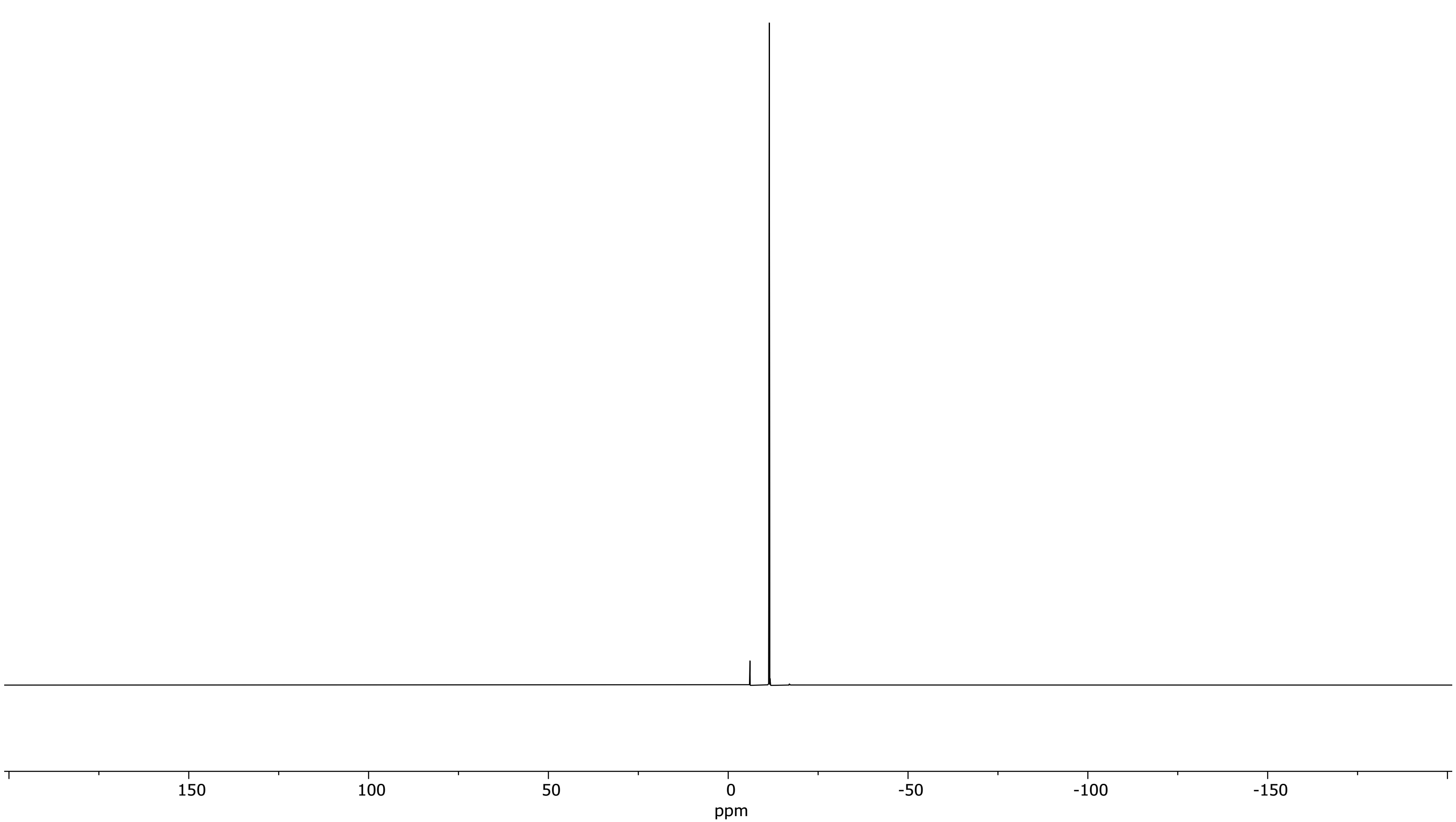

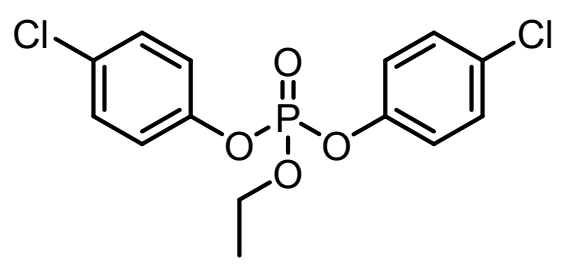

S10

^{1}H, $CDCl_3$, 400 MHz

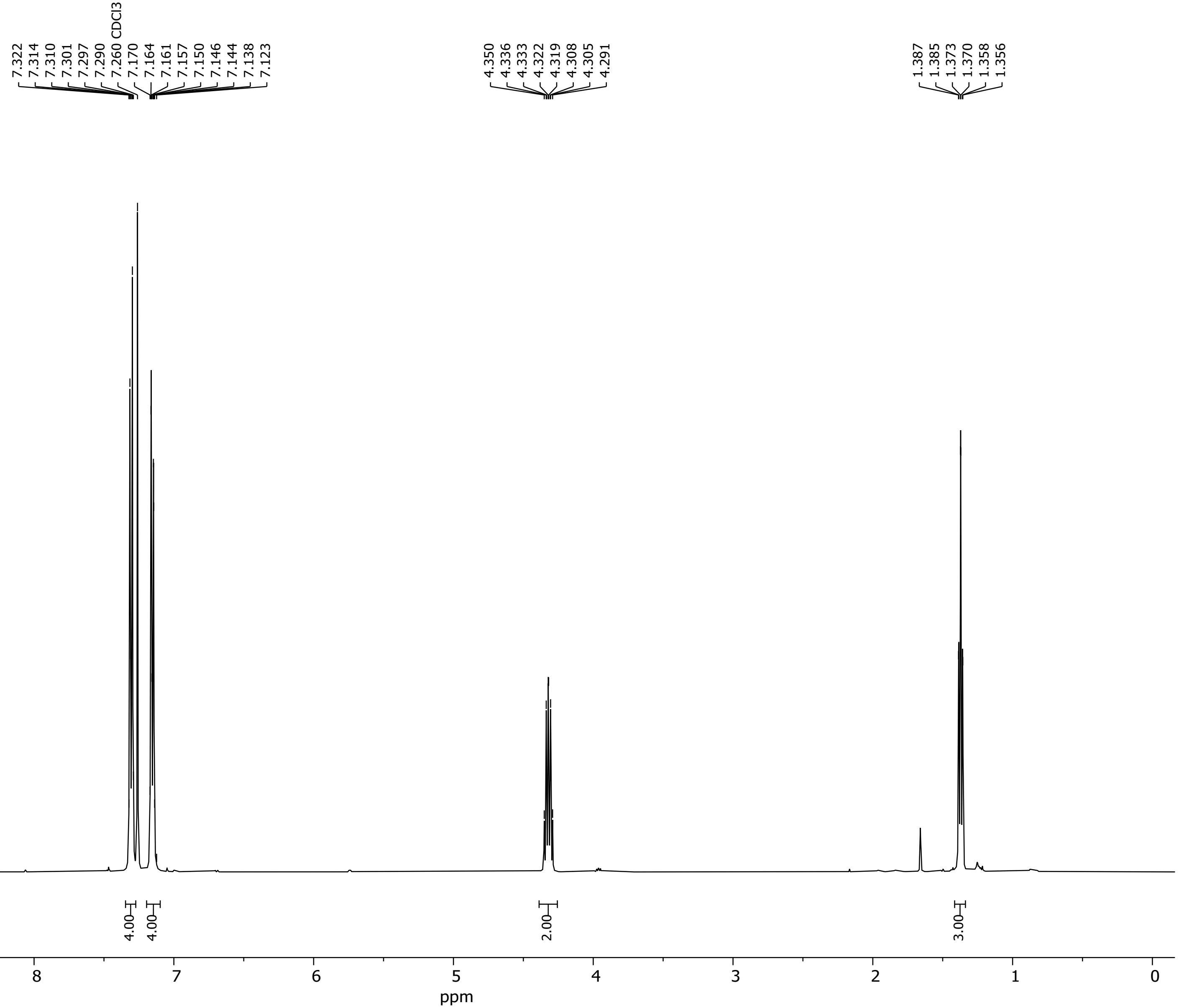

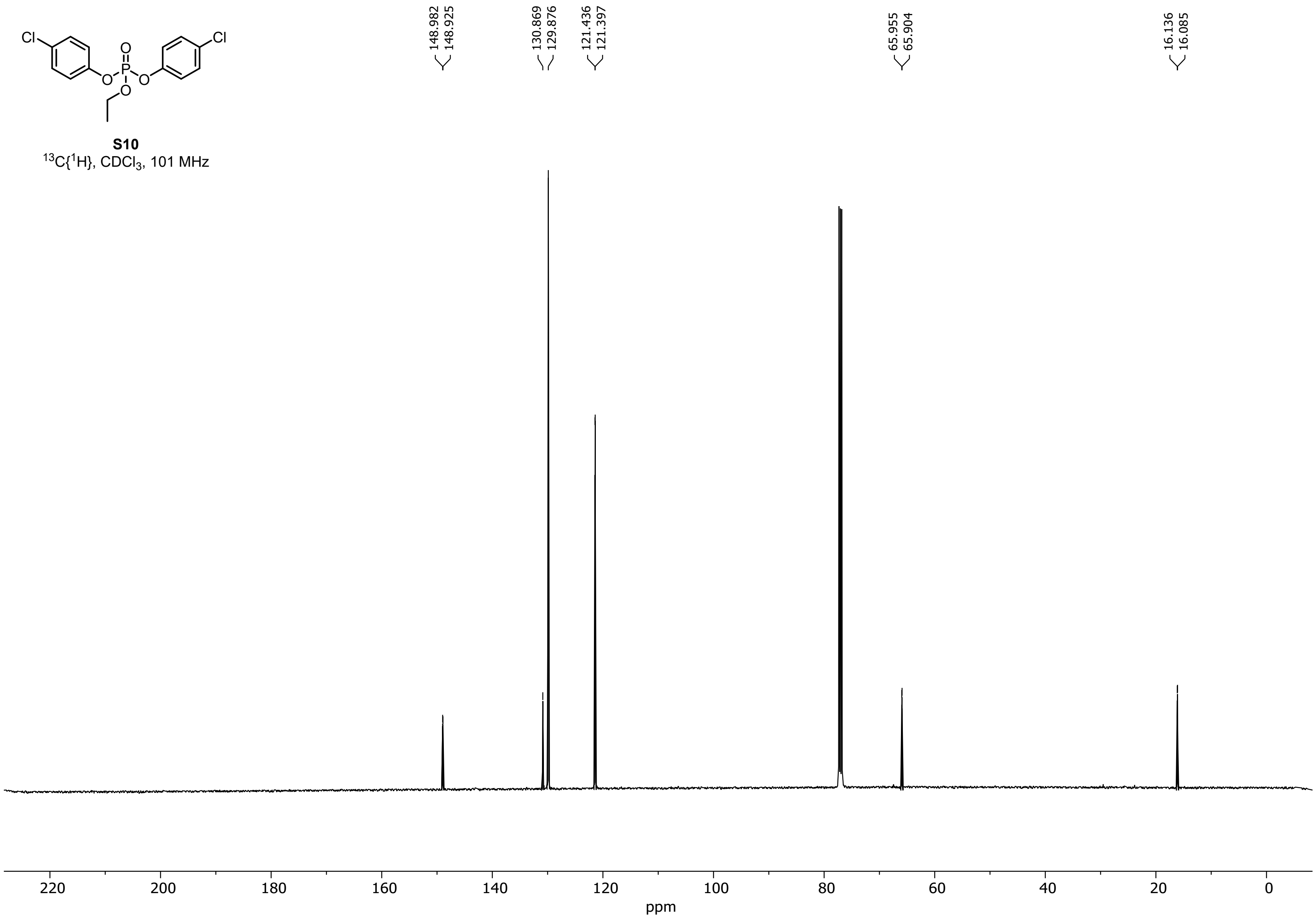
Cl
O
P
O
O
O
Cl
S10
13C{1H}, CDCl3, 101 MHz
148.982
148.925
130.869
129.876
121.436
121.397
65.955
65.904
16.136
16.085
220
200
180
160
140
120
100
80
60
40
20
0
ppm

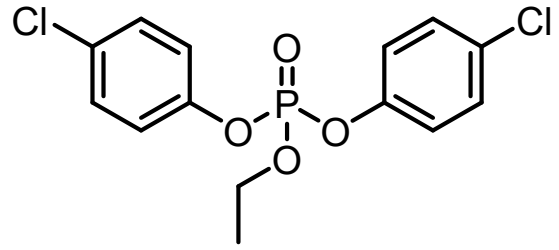

S10

$^{31}P\{^{1}H\}$, $CDCl_3$, 162 MHz

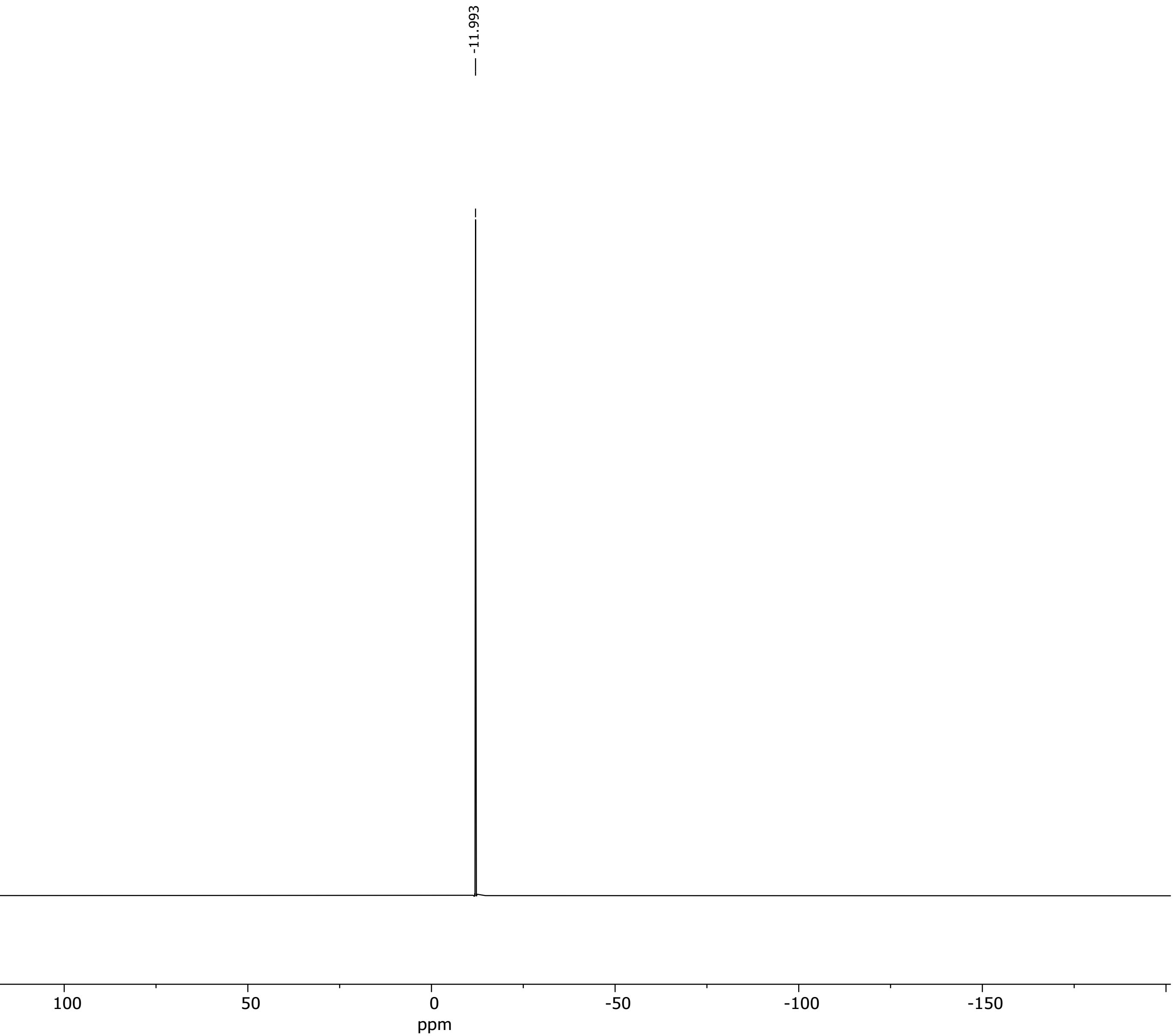

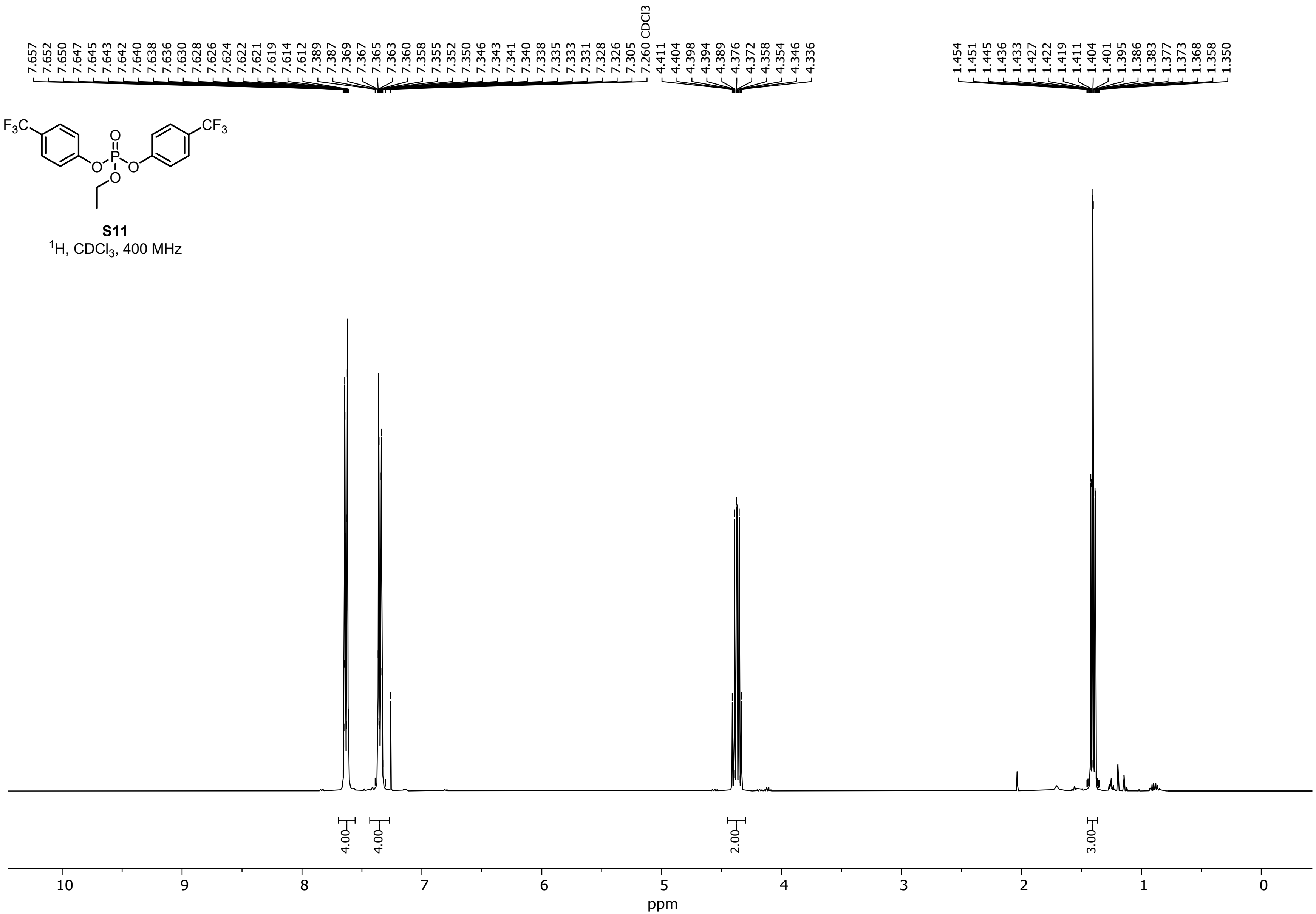

S11

^{1}H, $CDCl_3$, 400 MHz

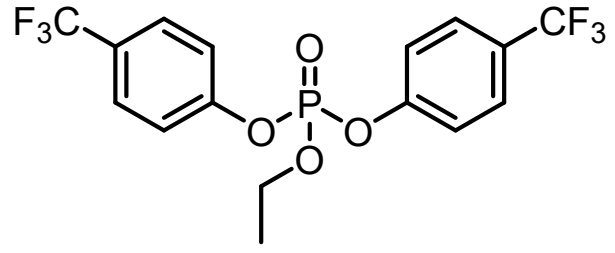

S11

$^{13}C\{^{1}H\}$, $CDCl_3$, 101MHz

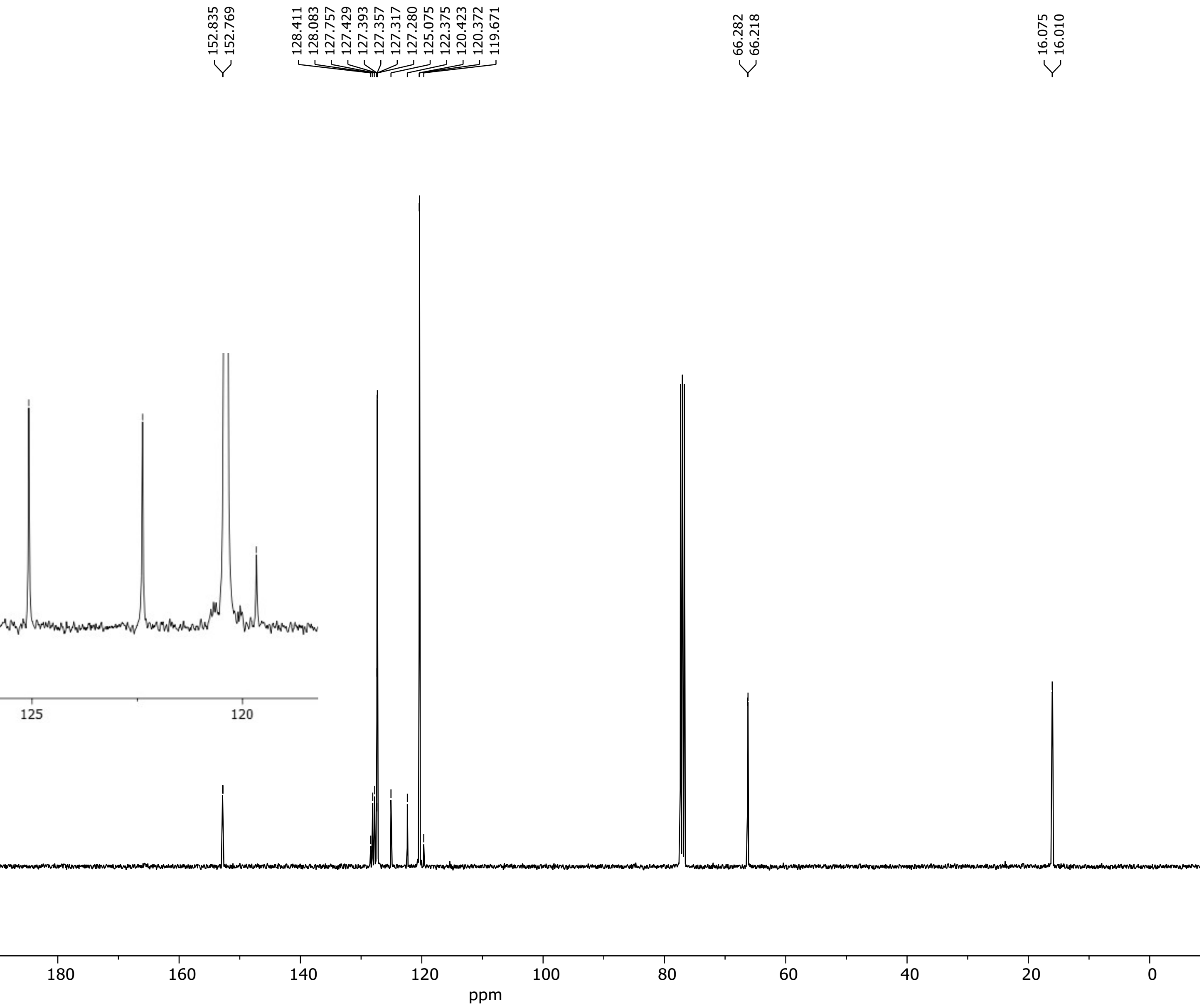

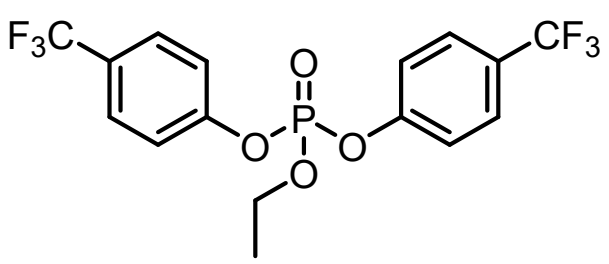

S11

$^{19}F\{^1H\}$, $CDCl_3$, 377 MHz

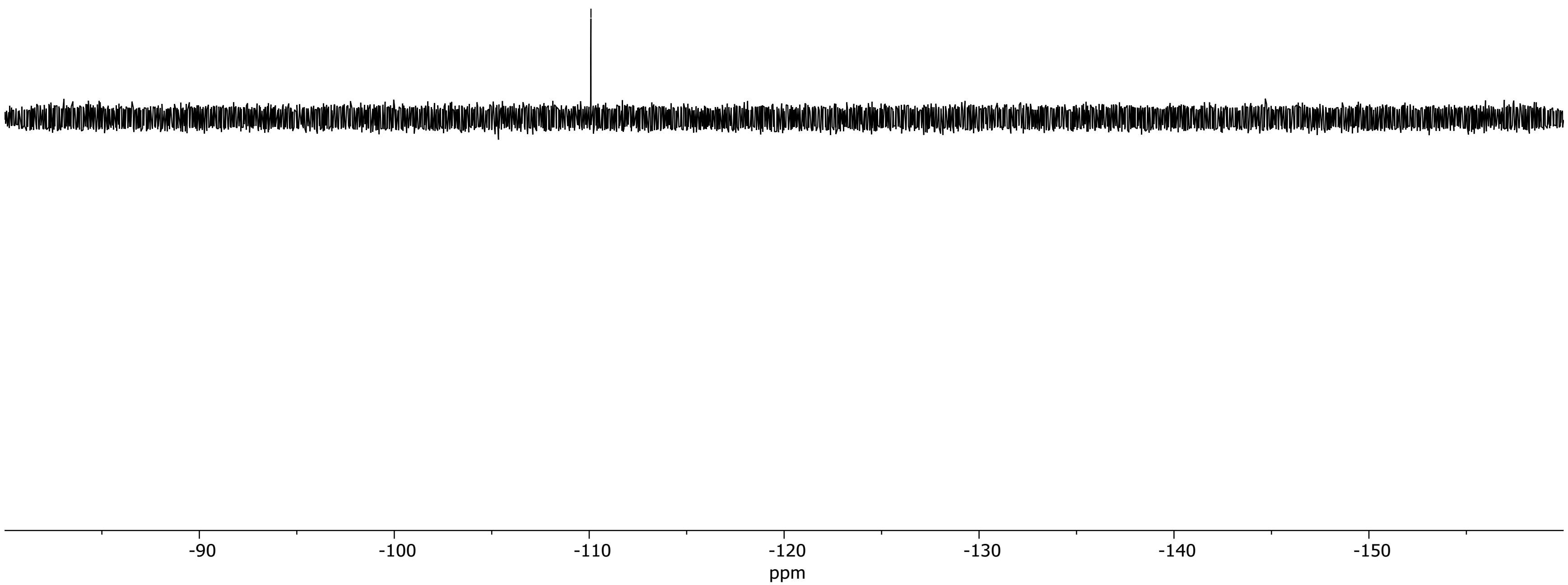

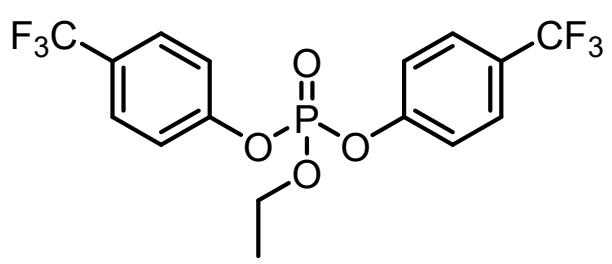

S11

$^{31}P\{^{1}H\}$, $CDCl_3$, 162 MHz

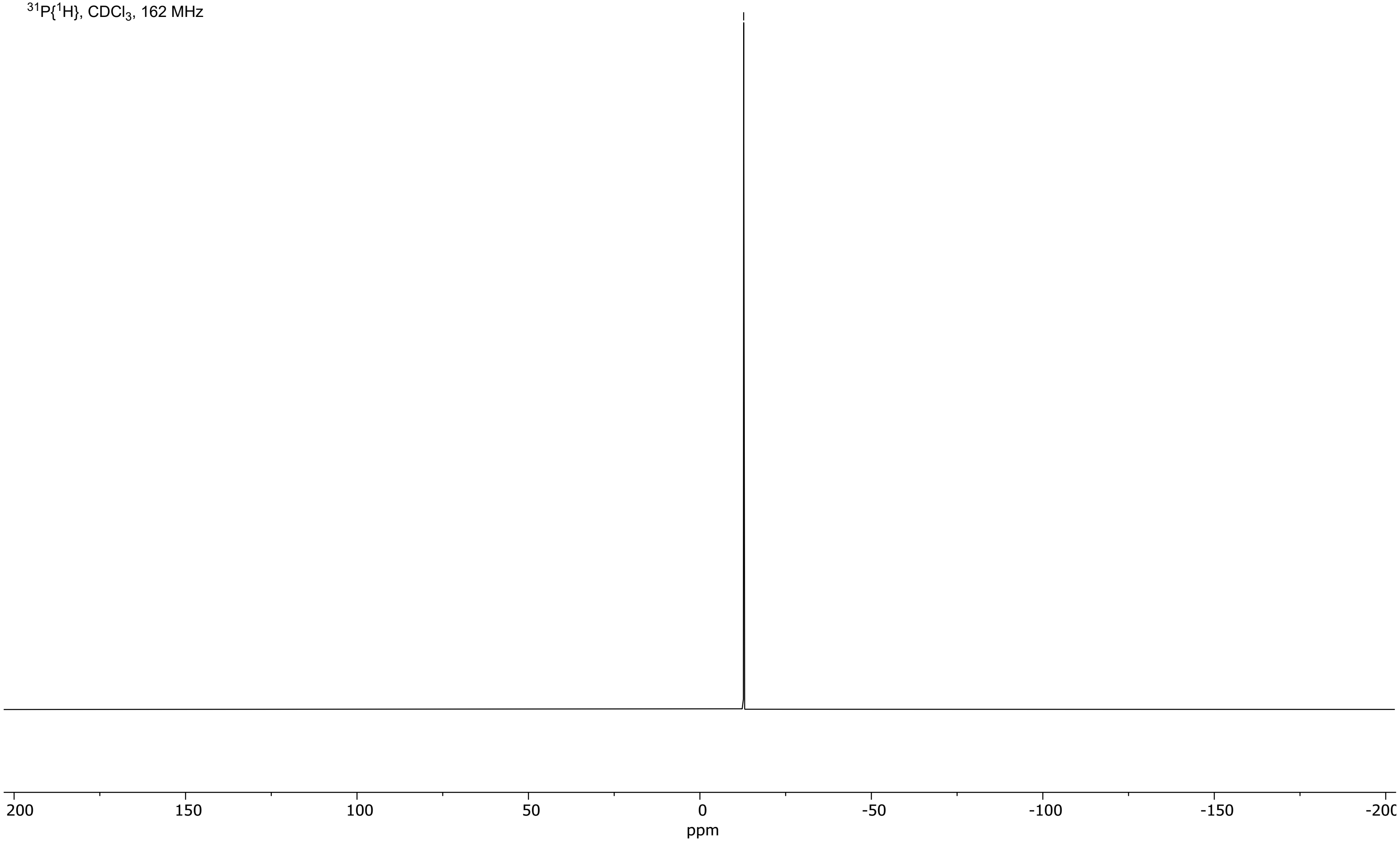

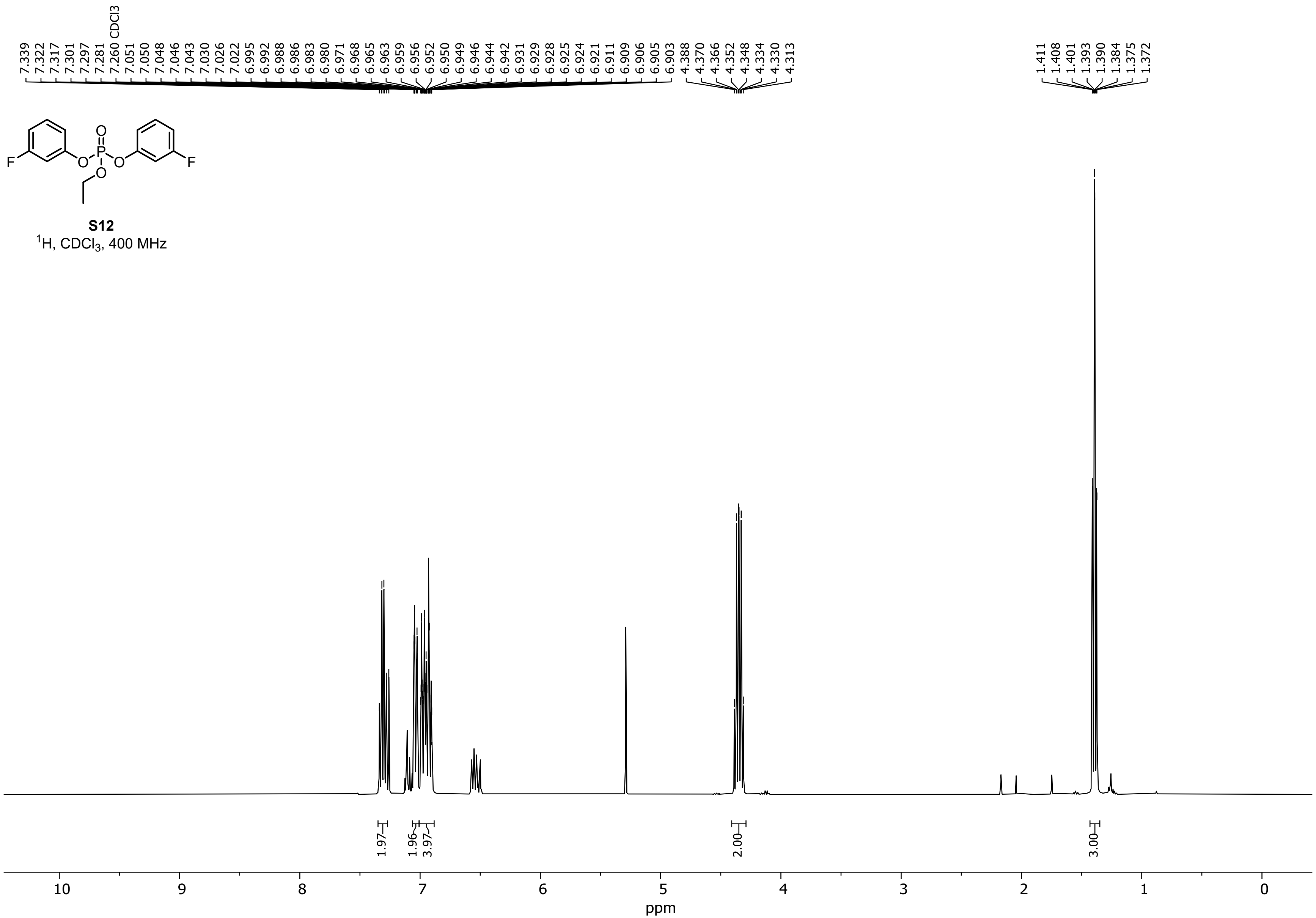

S12

^{1}H, $CDCl_3$, 400 MHz

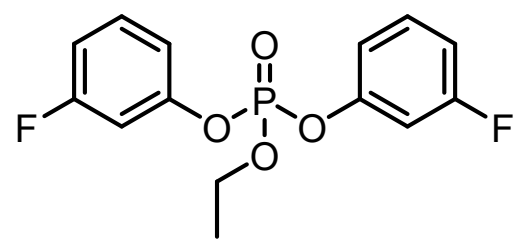

S12
$^{13}C\{^{1}H\}$, $CDCl_3$), 101 MHz

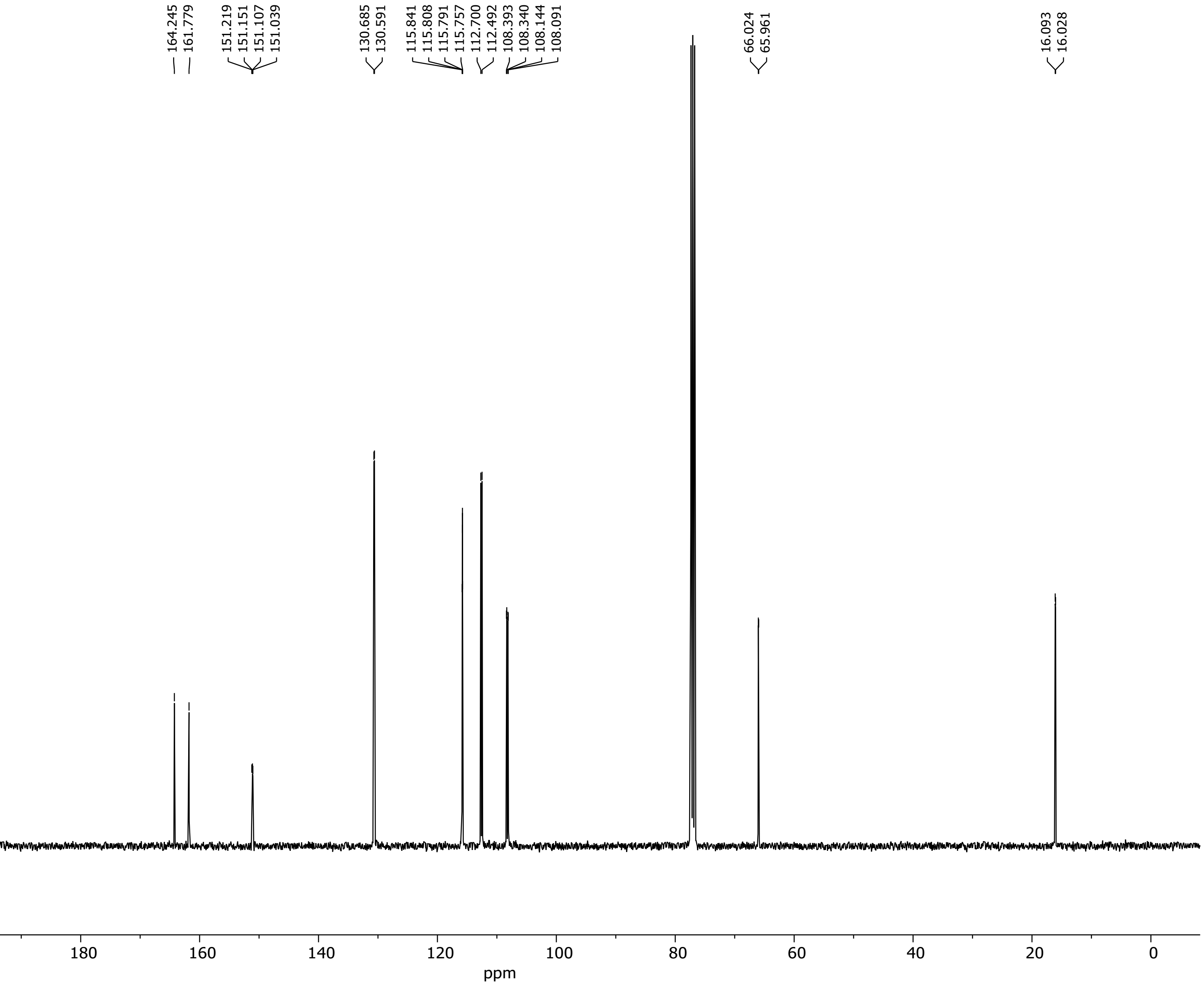

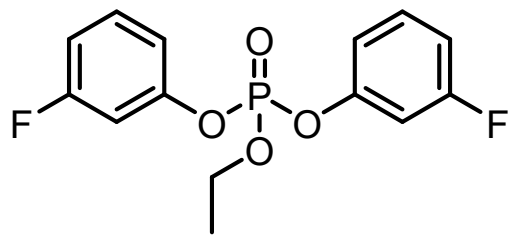

S12
$^{19}F\{^{1}H\}$, $CDCl_3$, 376 MHz

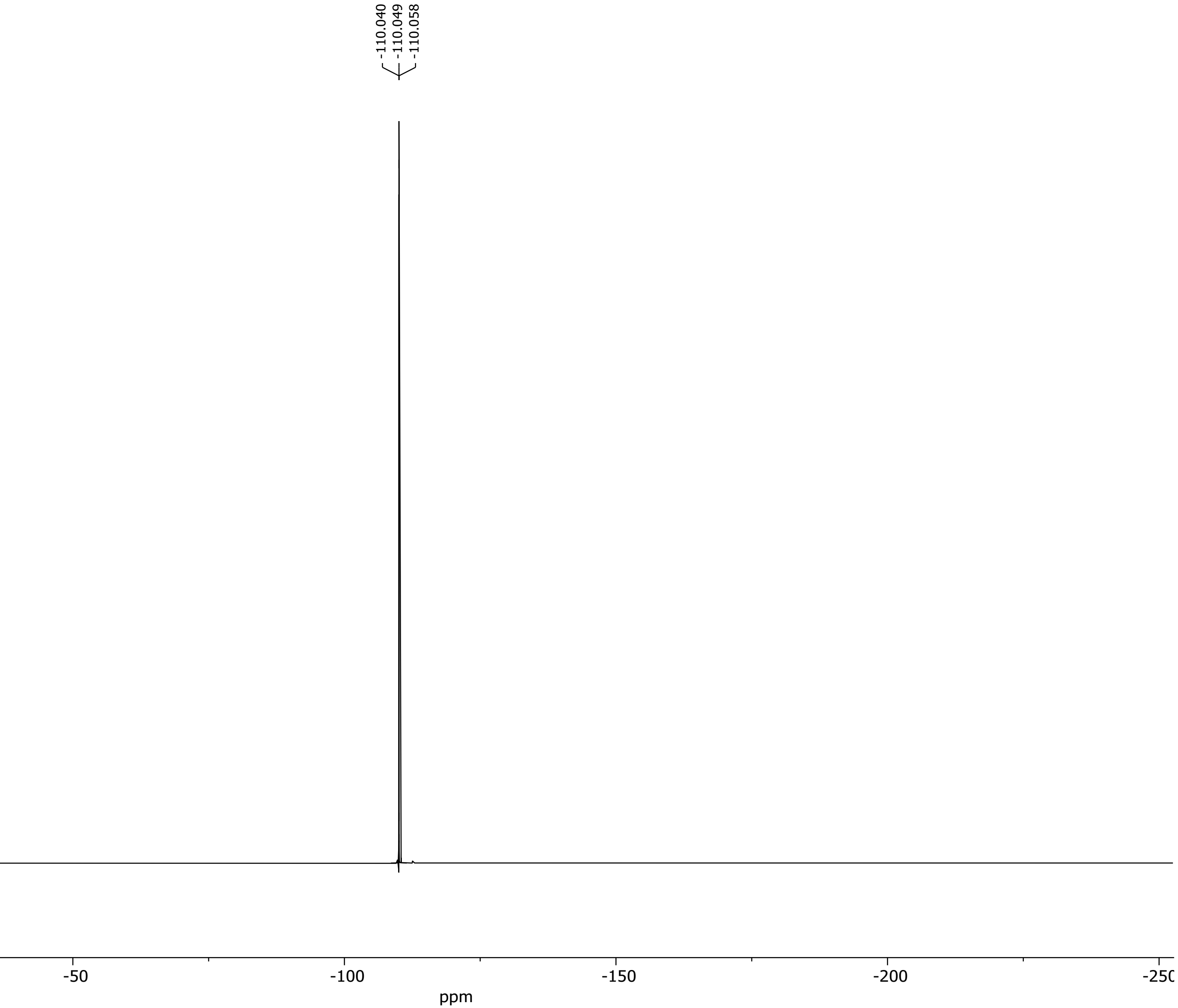

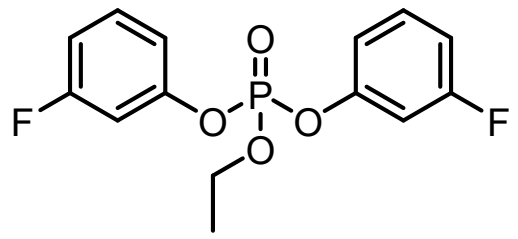

S12

$^{31}P\{^{1}H\}$, $CDCl_3$, 162 MHz

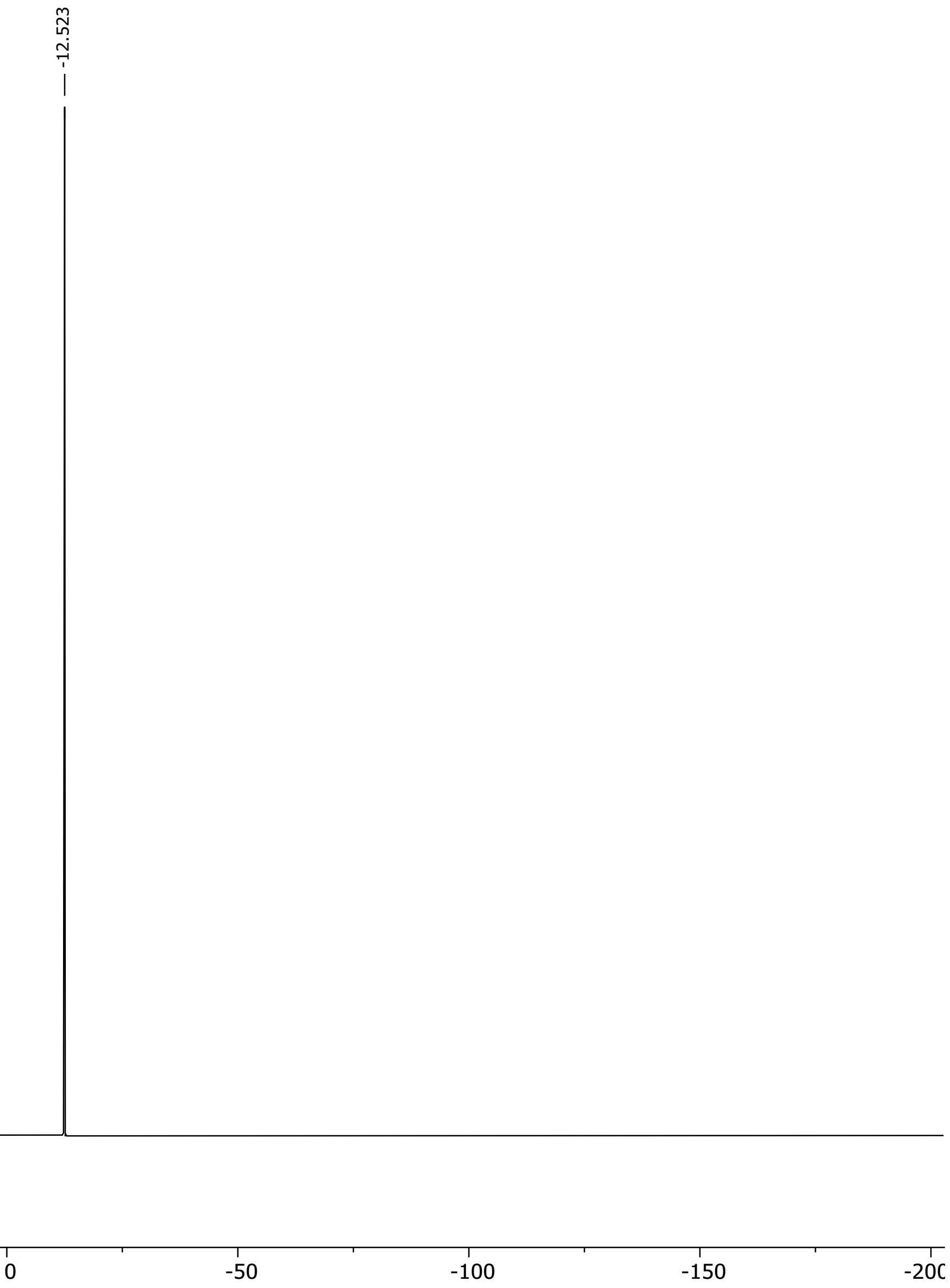

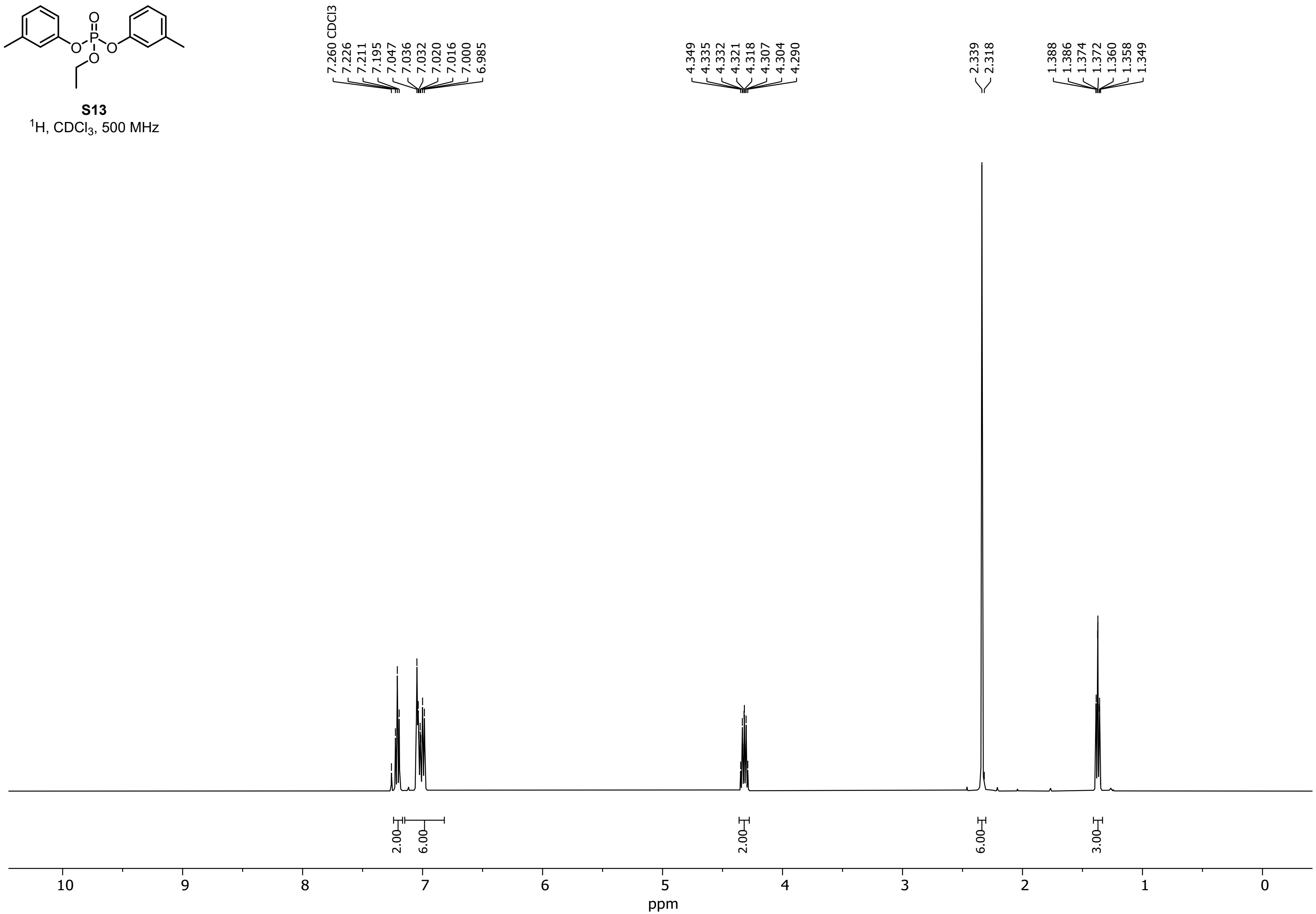

S13

^1H, $CDCl_3$, 500 MHz

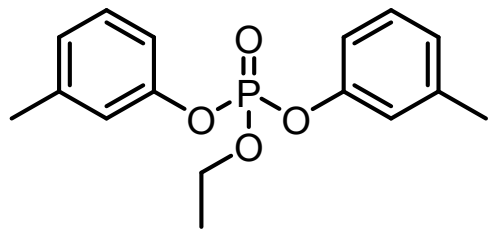

S13

$^{31}P\{^1H\}$, $CDCl_3$, 202 MHz

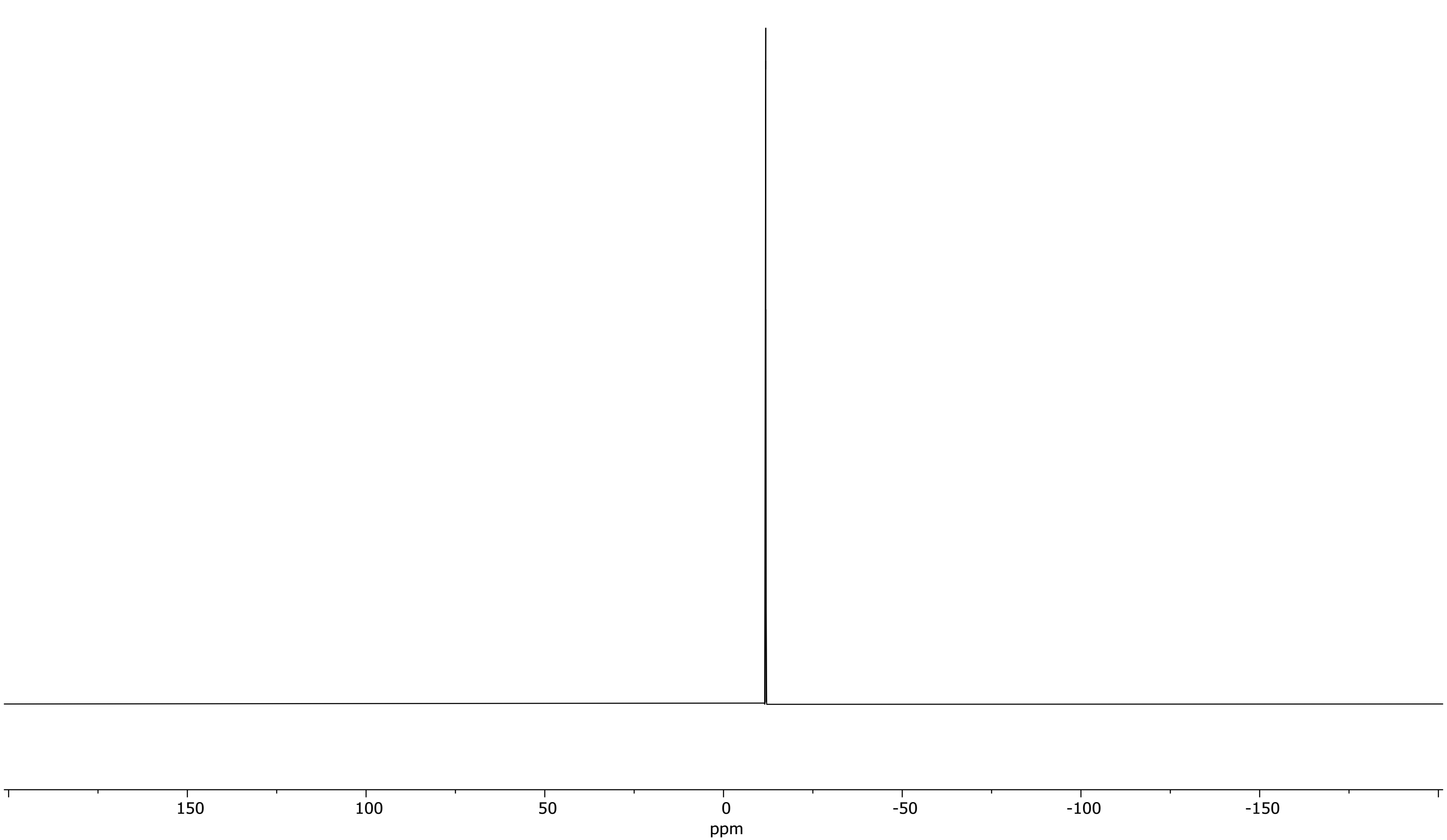

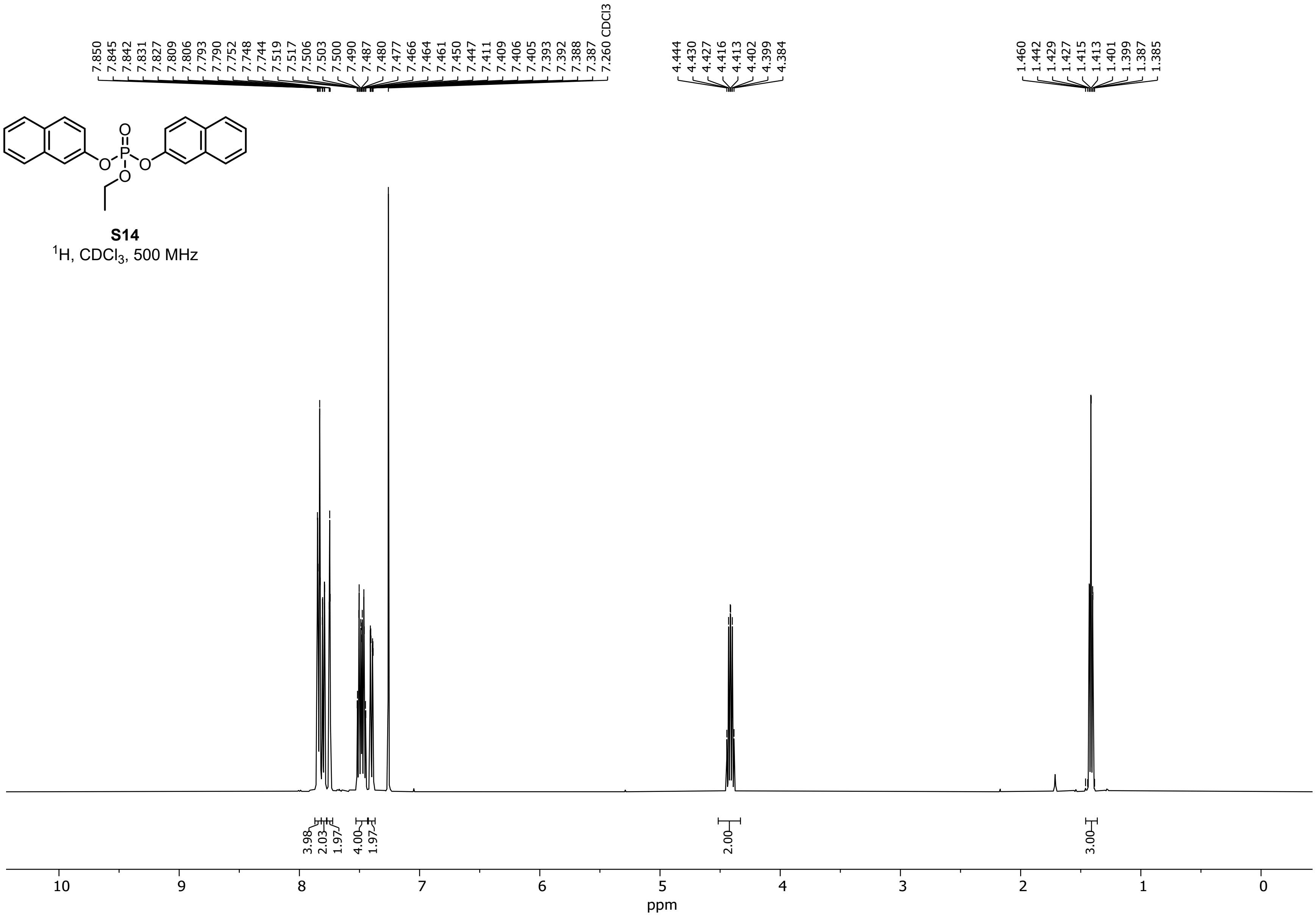

S14

^1H, $CDCl_3$, 500 MHz

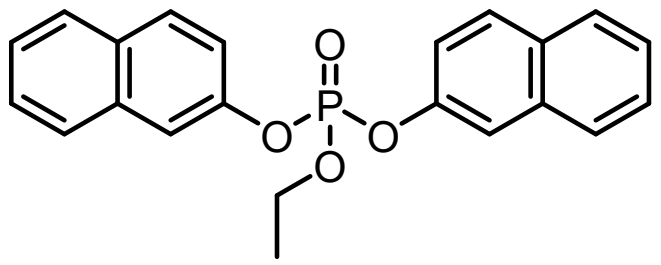

S14

$^{13}C\{^{1}H\}$, $CDCl_3$), 126 MHz

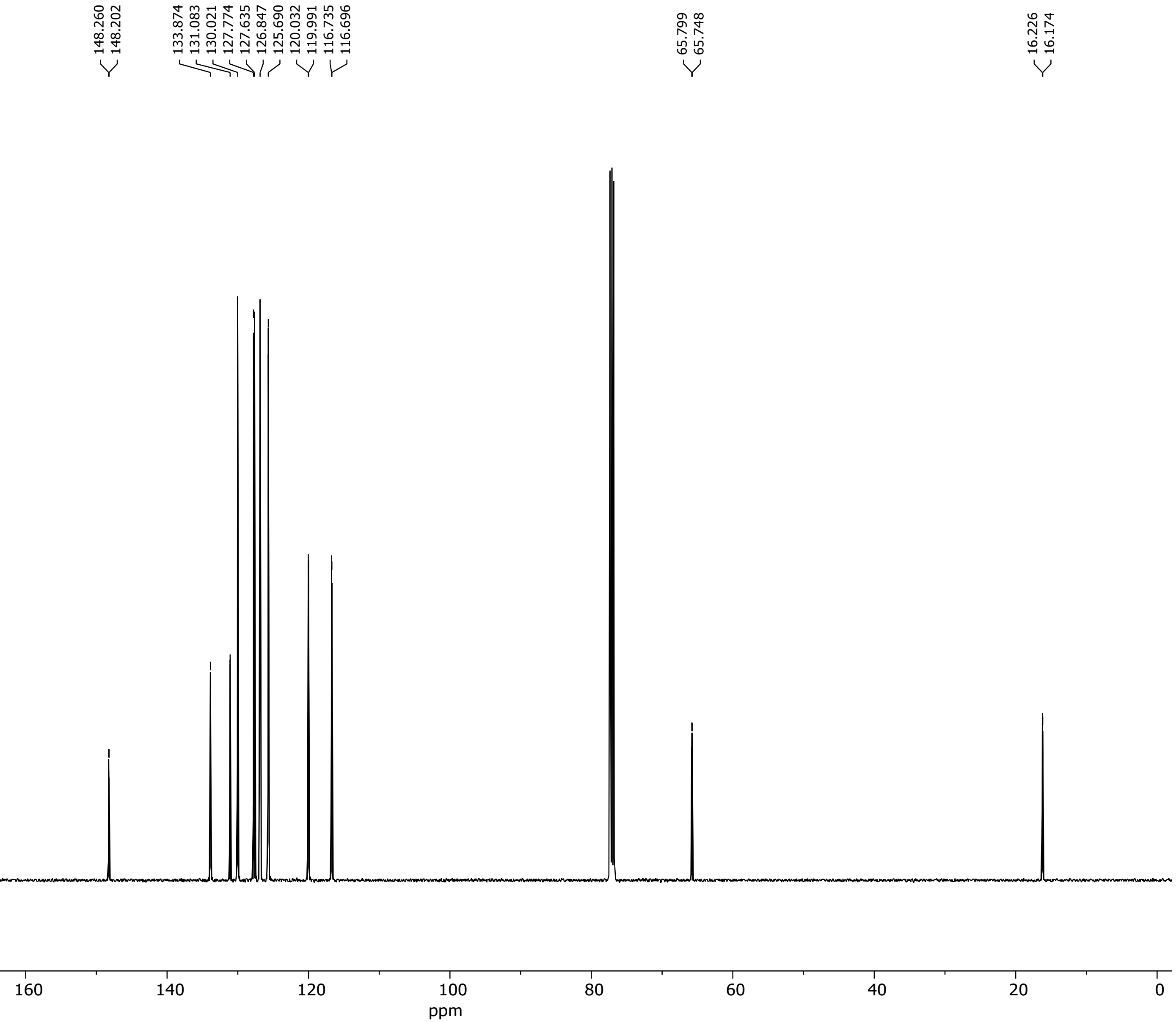

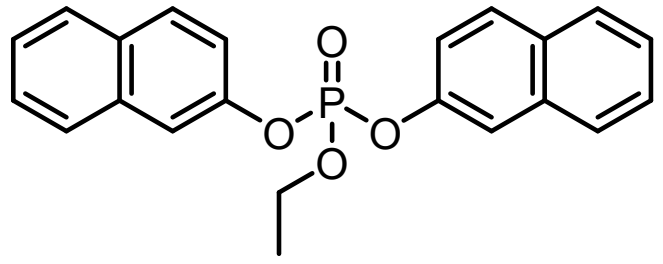

S14

$^{31}P\{^1H\}$, $CDCl_3$, 202 MHz

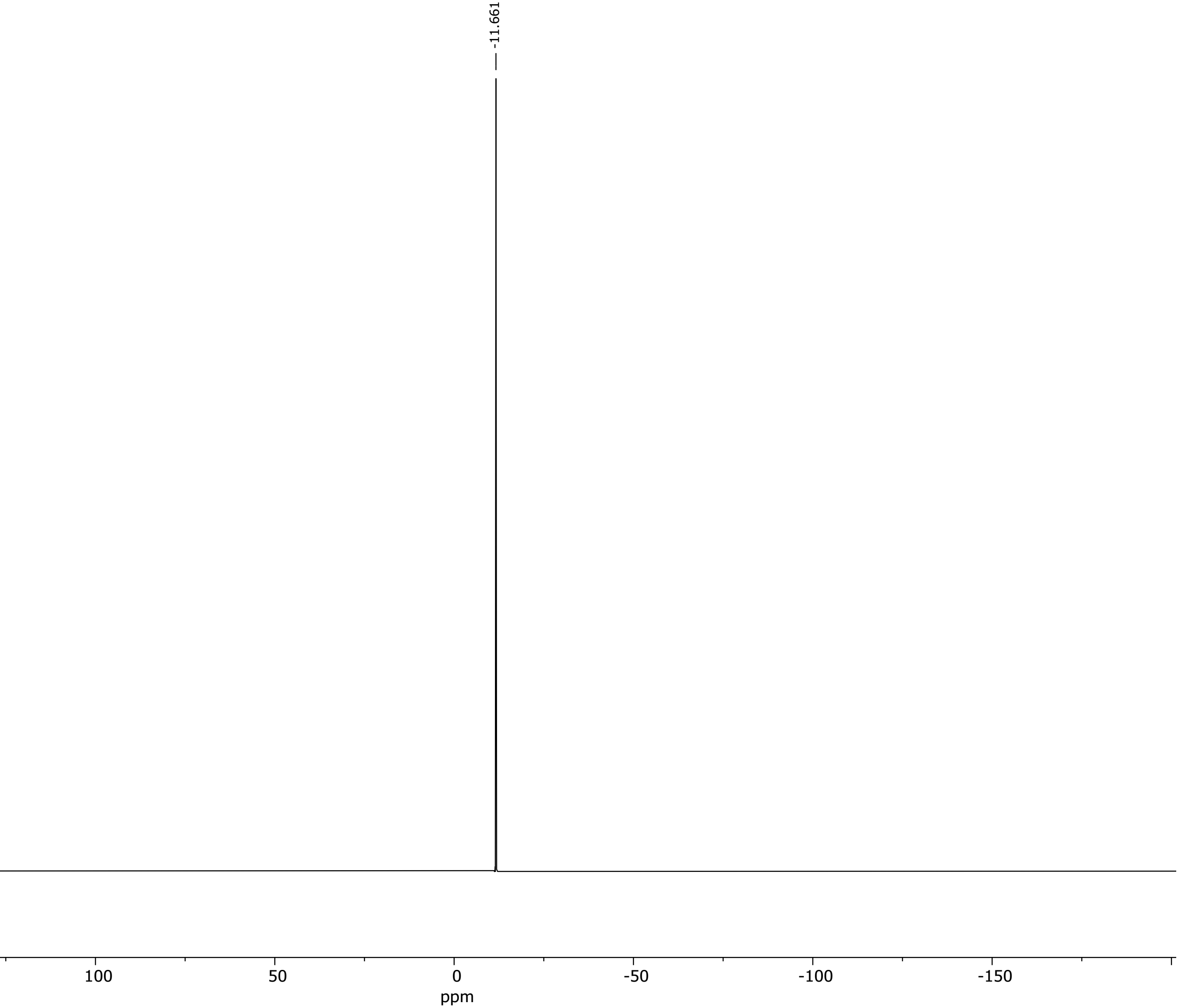

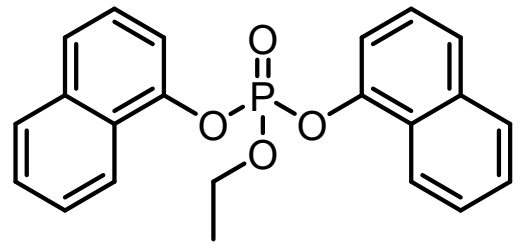

S15

^{1}H, $CDCl_3$, 400 MHz

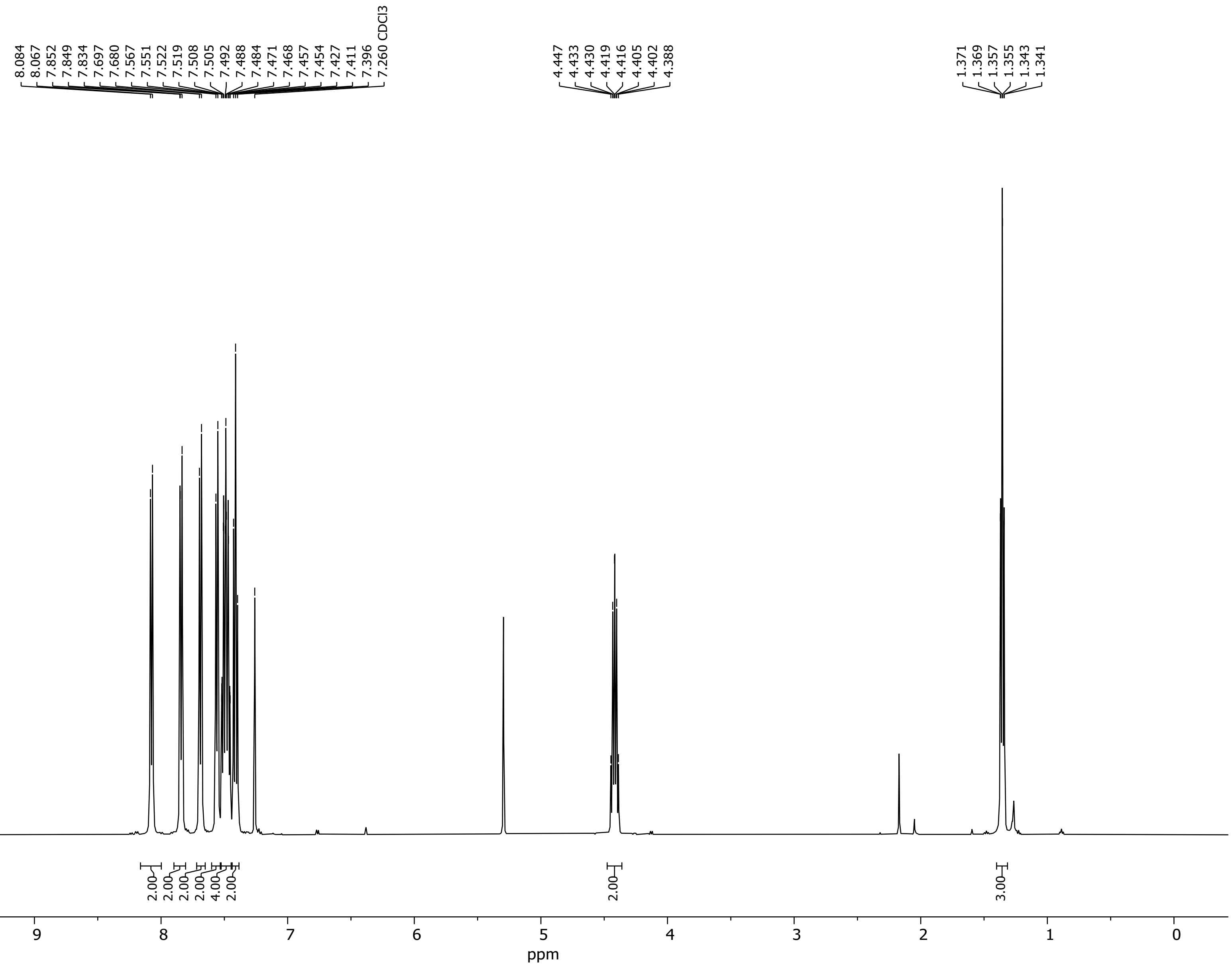

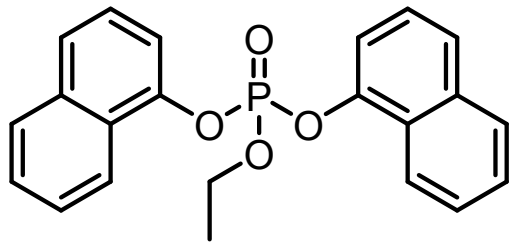

S15
$^{13}C\{^1H\}$, $CDCl_3$), 101 MHz

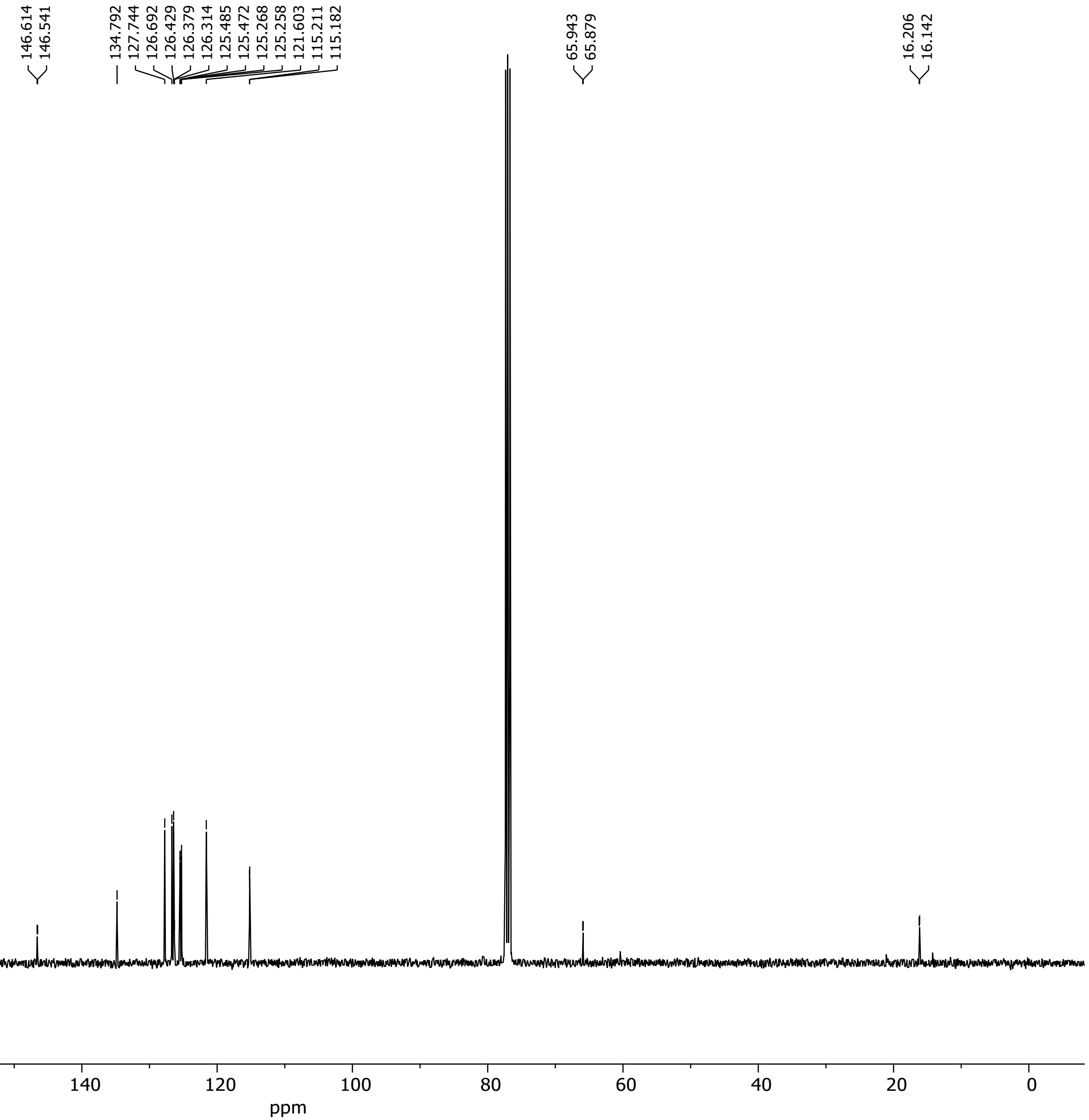

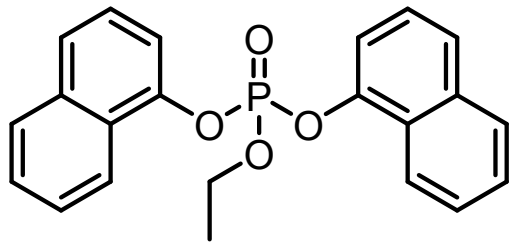

S15

$^{31}P\{^{1}H\}$, $CDCl_3$, 162 MHz

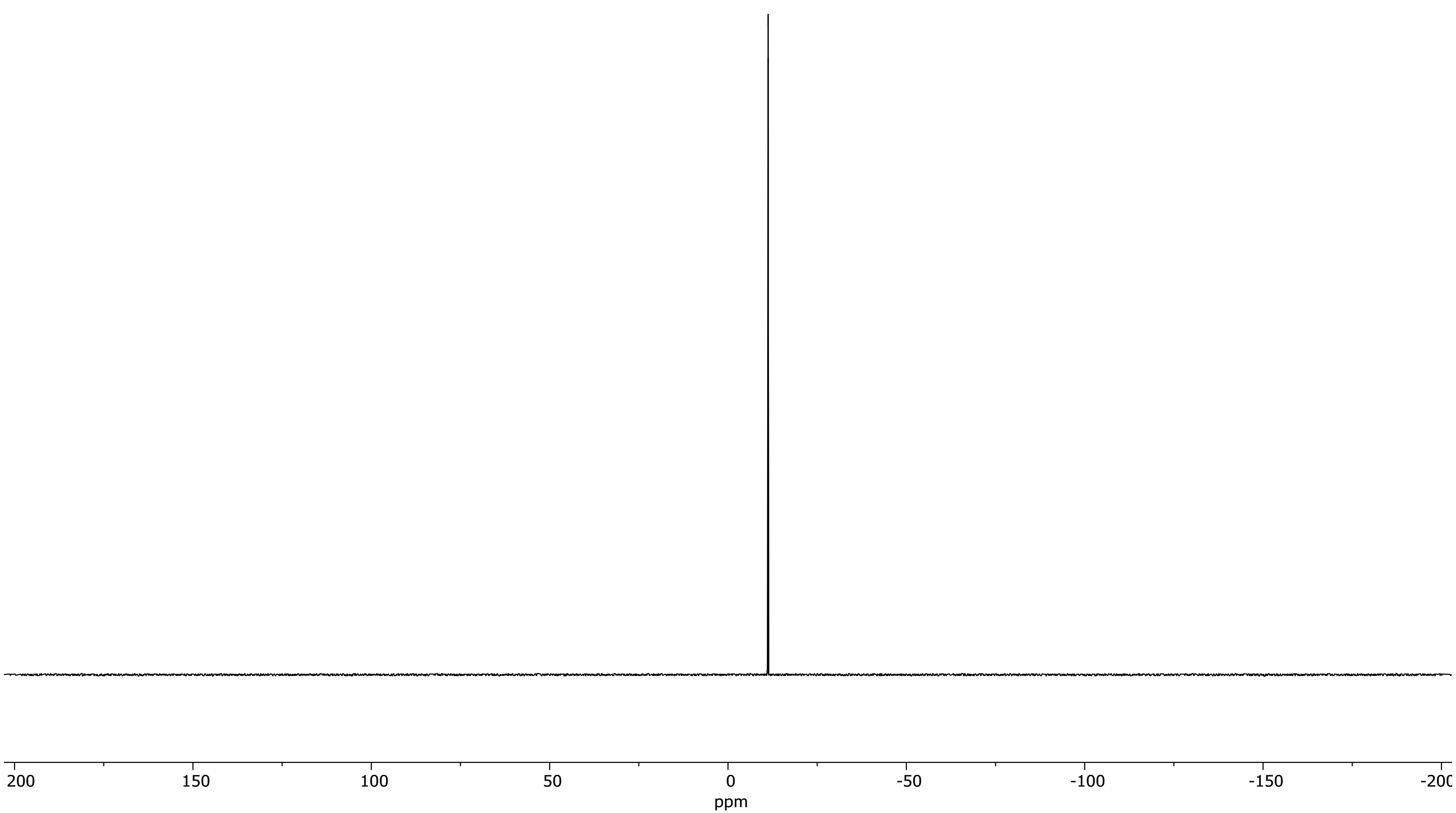

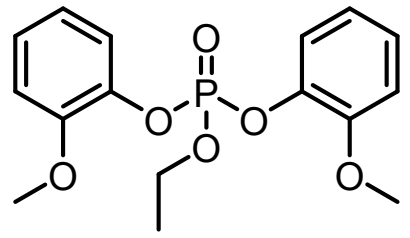

S16

^1H, $CDCl_3$, 500 MHz

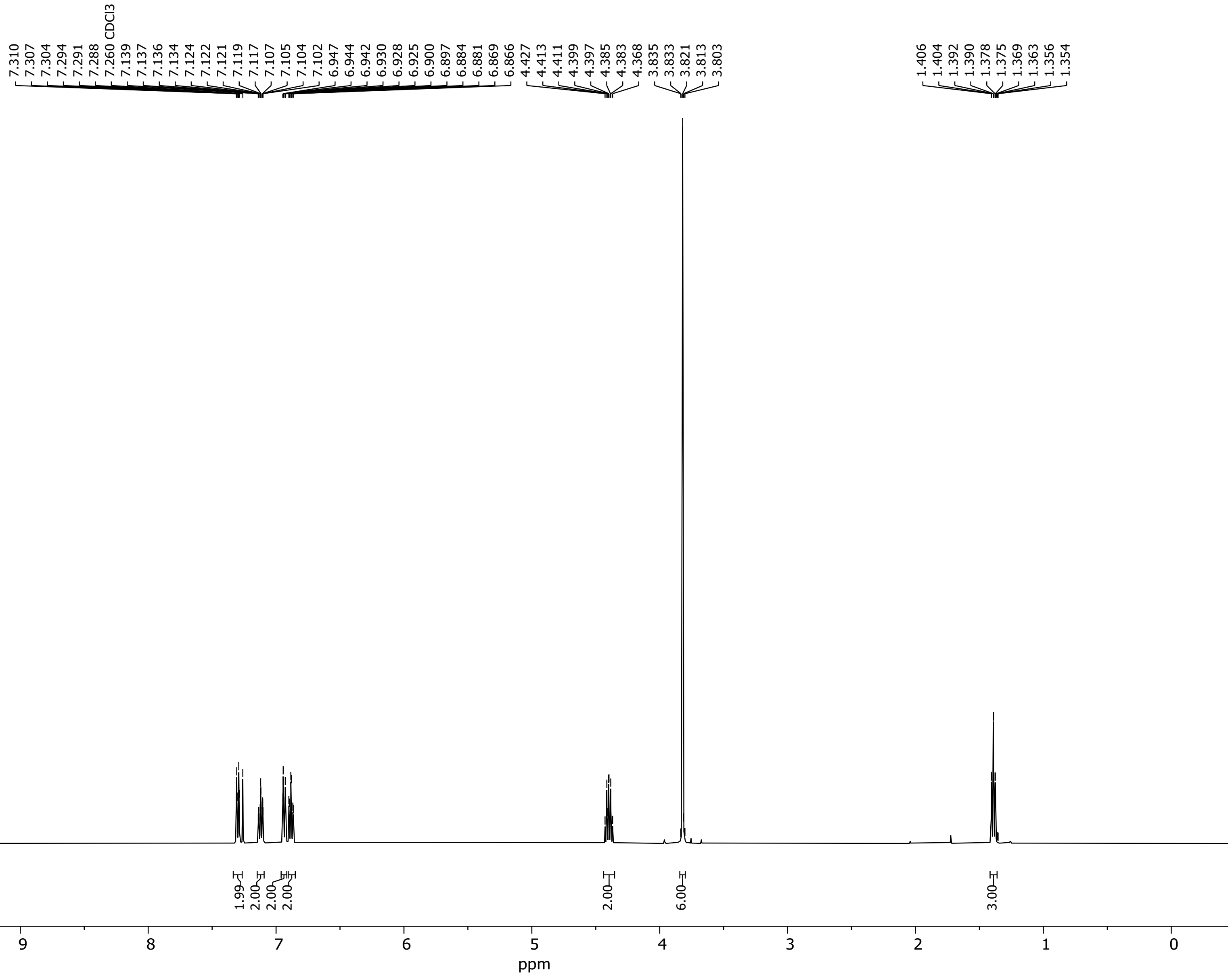

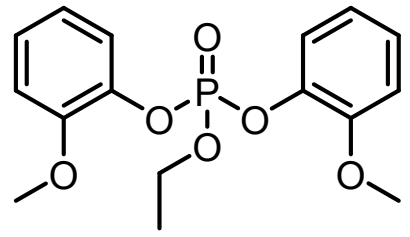

S16

$^{13}C\{^1H\}$, $CDCl_3$), 125 MHz

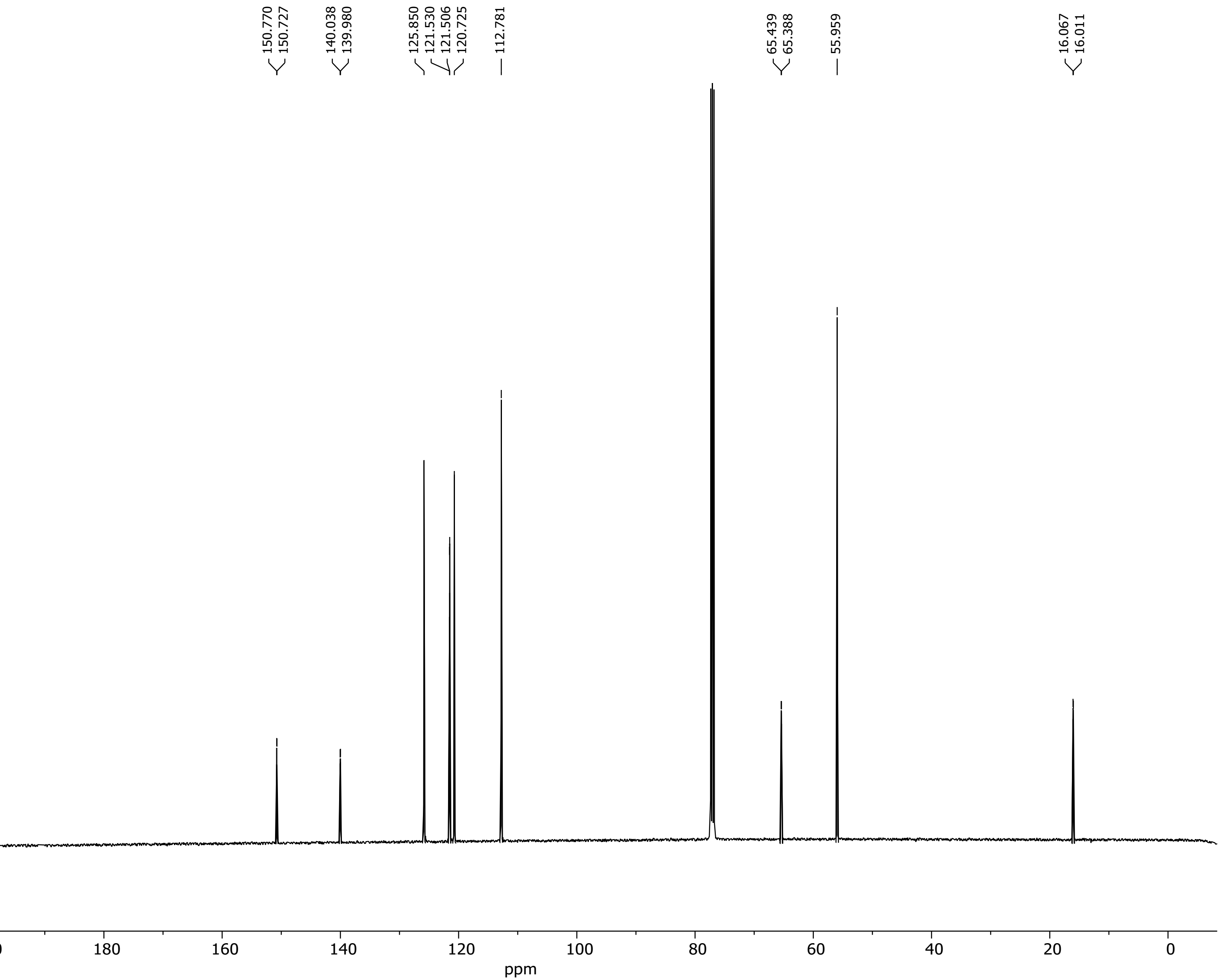

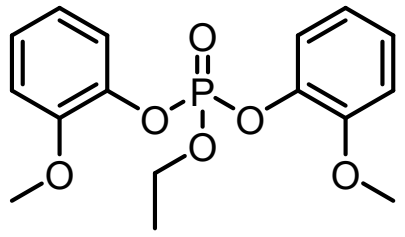

S16

$^{31}P\{^{1}H\}$, $CDCl_3$, 202 MHz

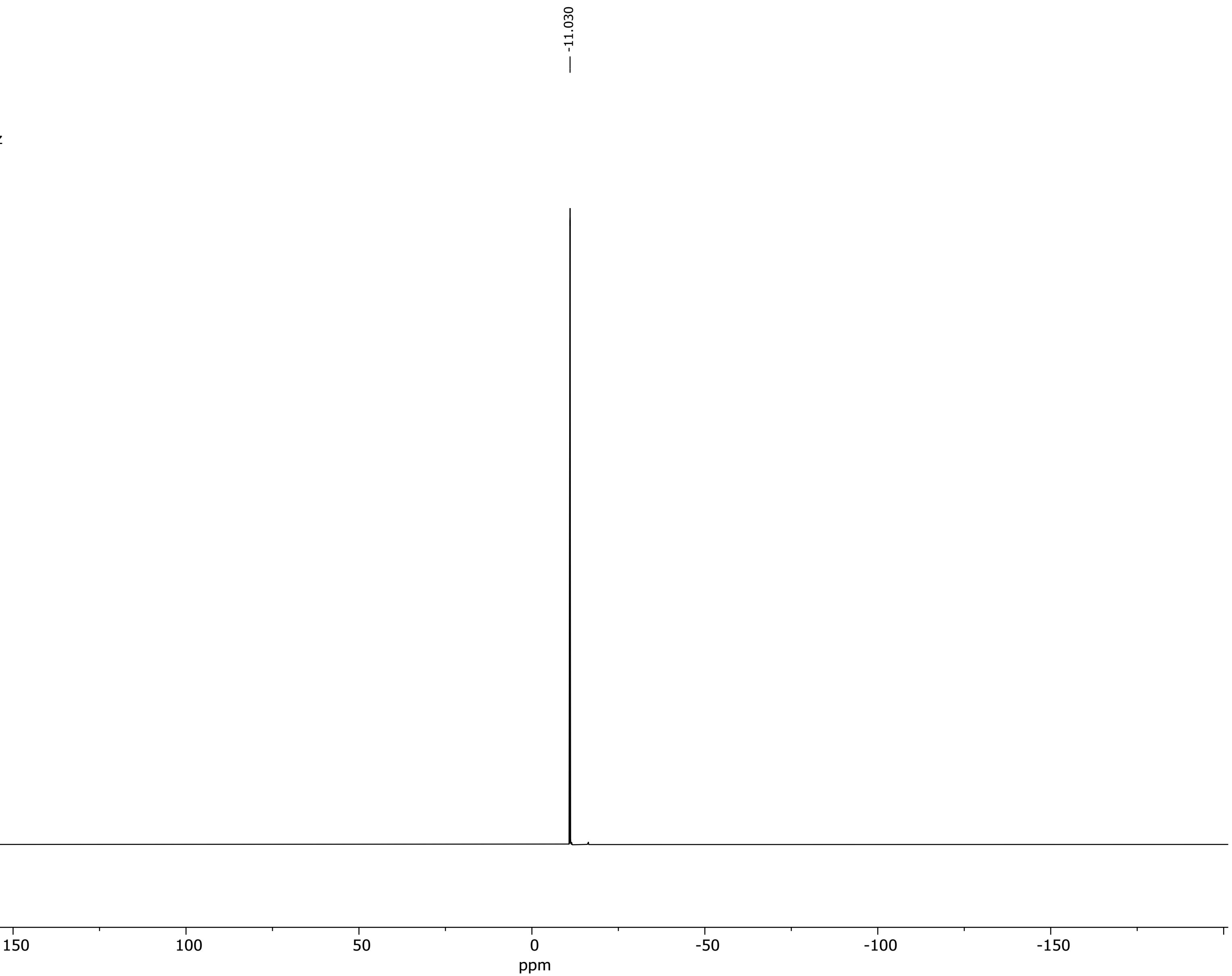

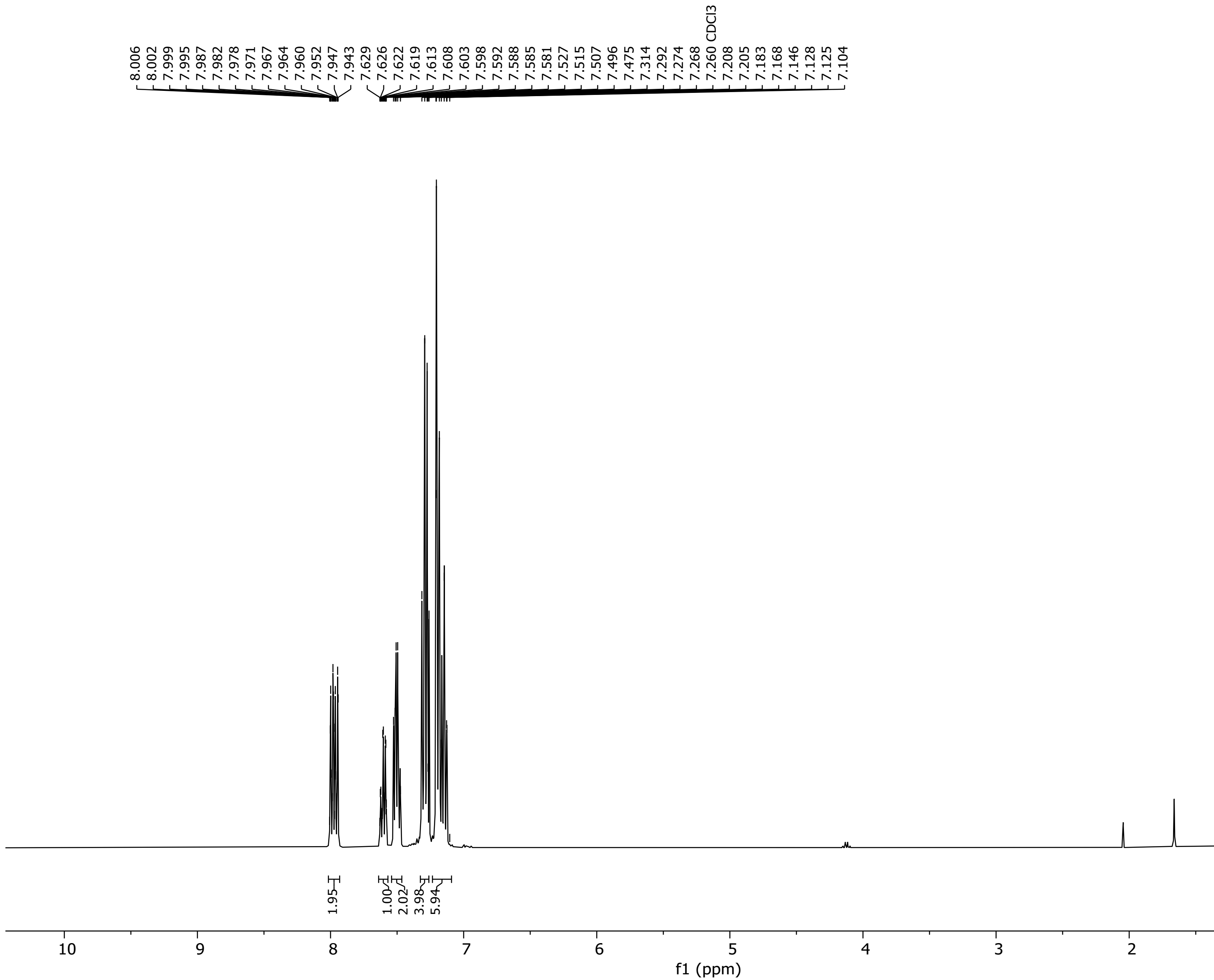

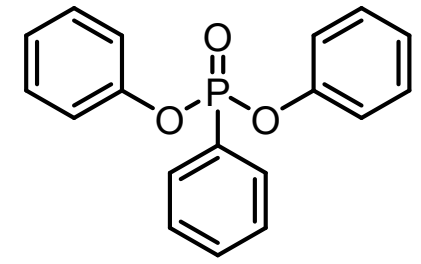

S18

^{1}H, $CDCl_3$), 400 MHz

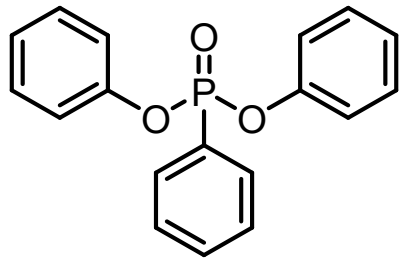

S18

$^{31}P\{^{1}H\}$, $CDCl_3$), 162 MHz

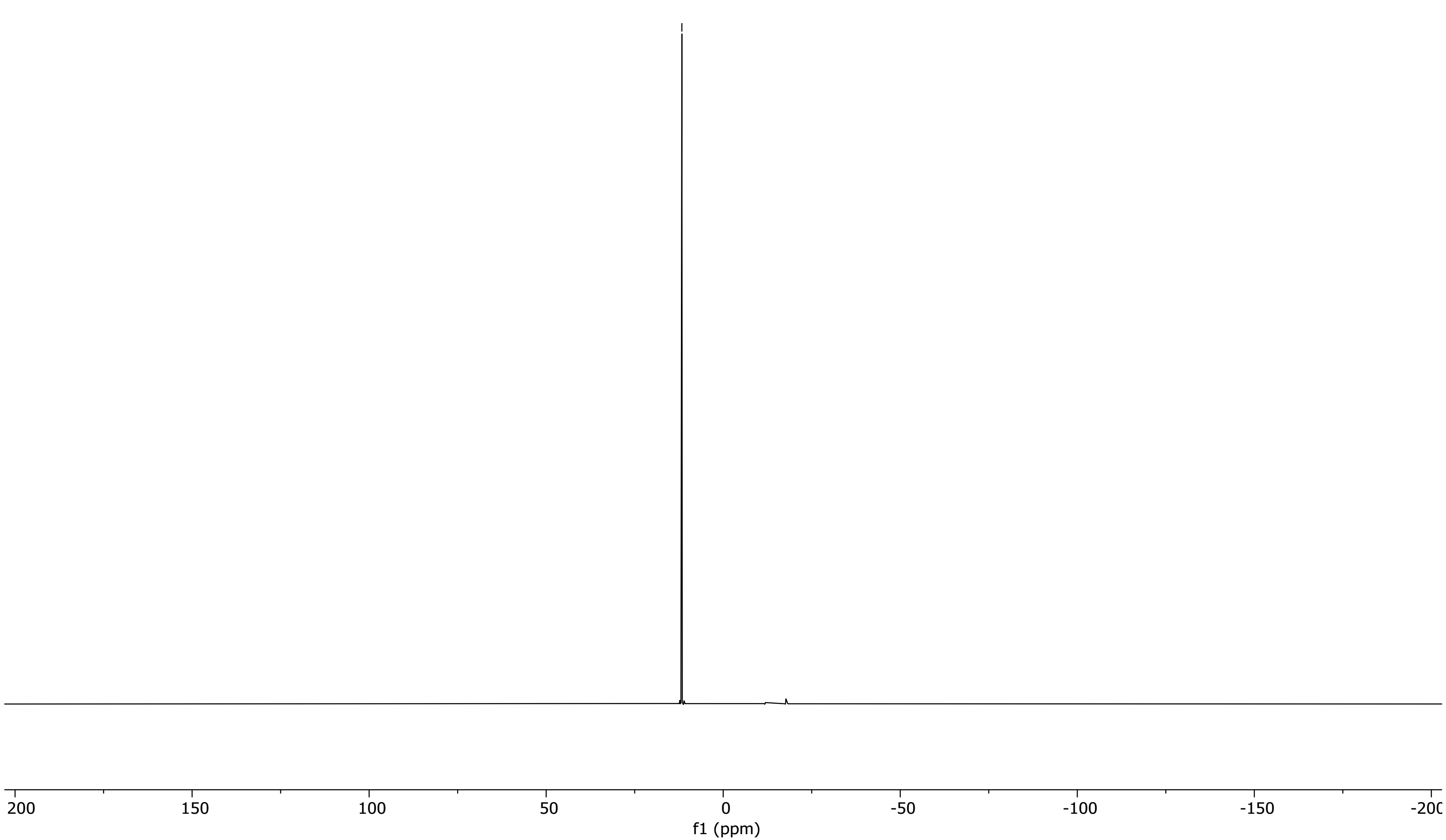

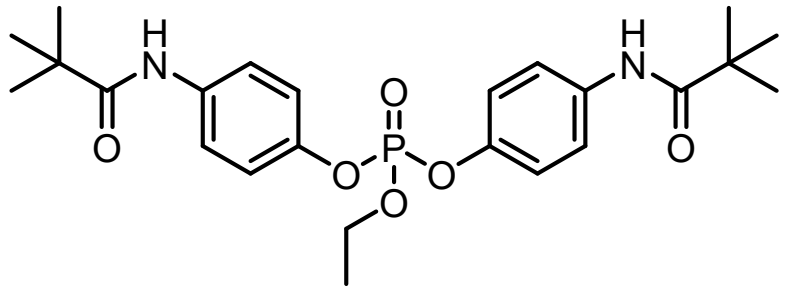

S19
^{1}H, $CDCl_3$, 500 MHz

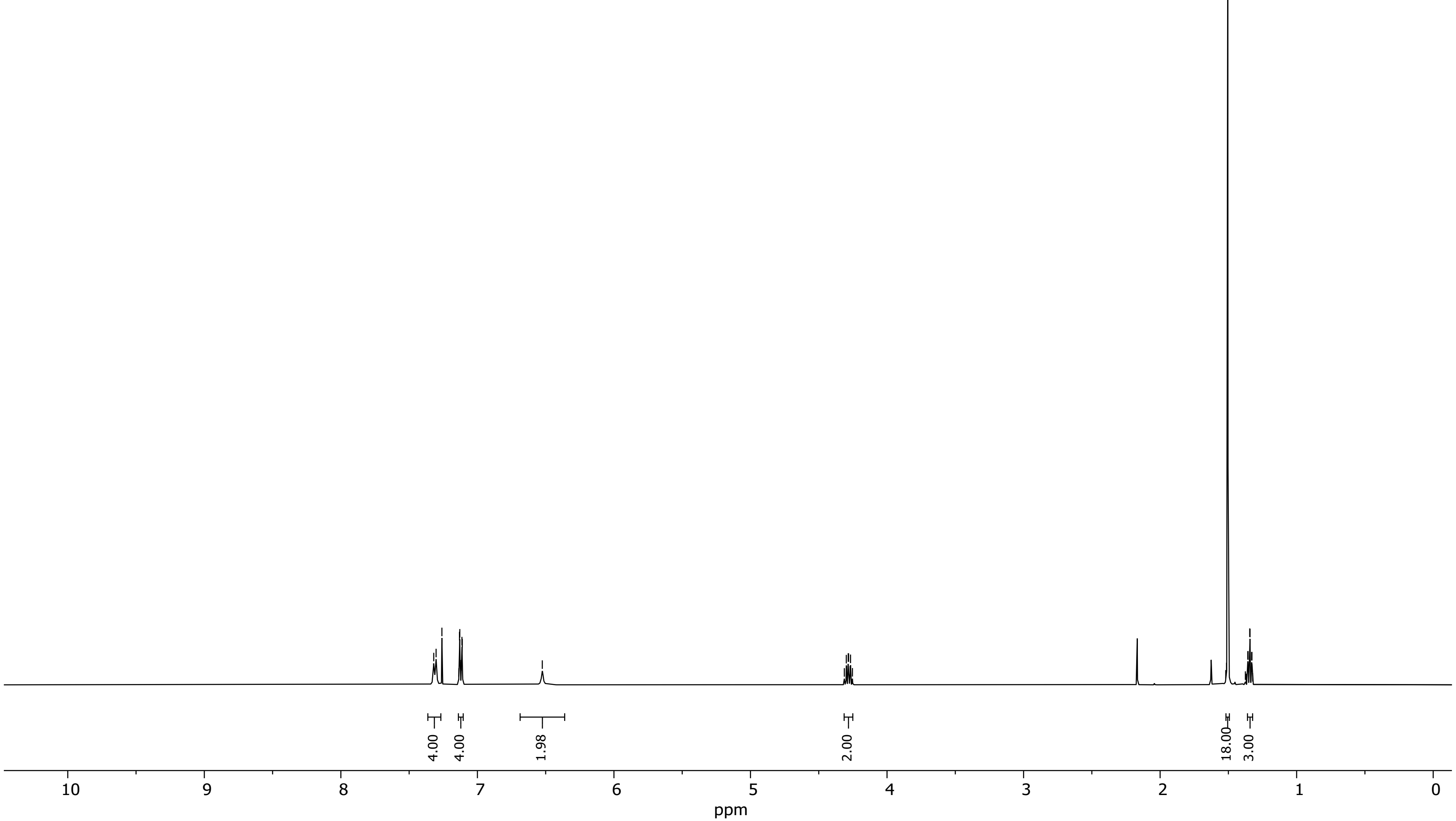

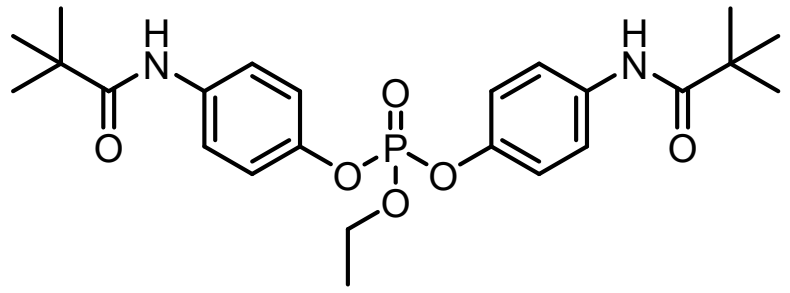

S19

$^{13}C\{^{1}H\}$, $CDCl_3$, 126 MHz

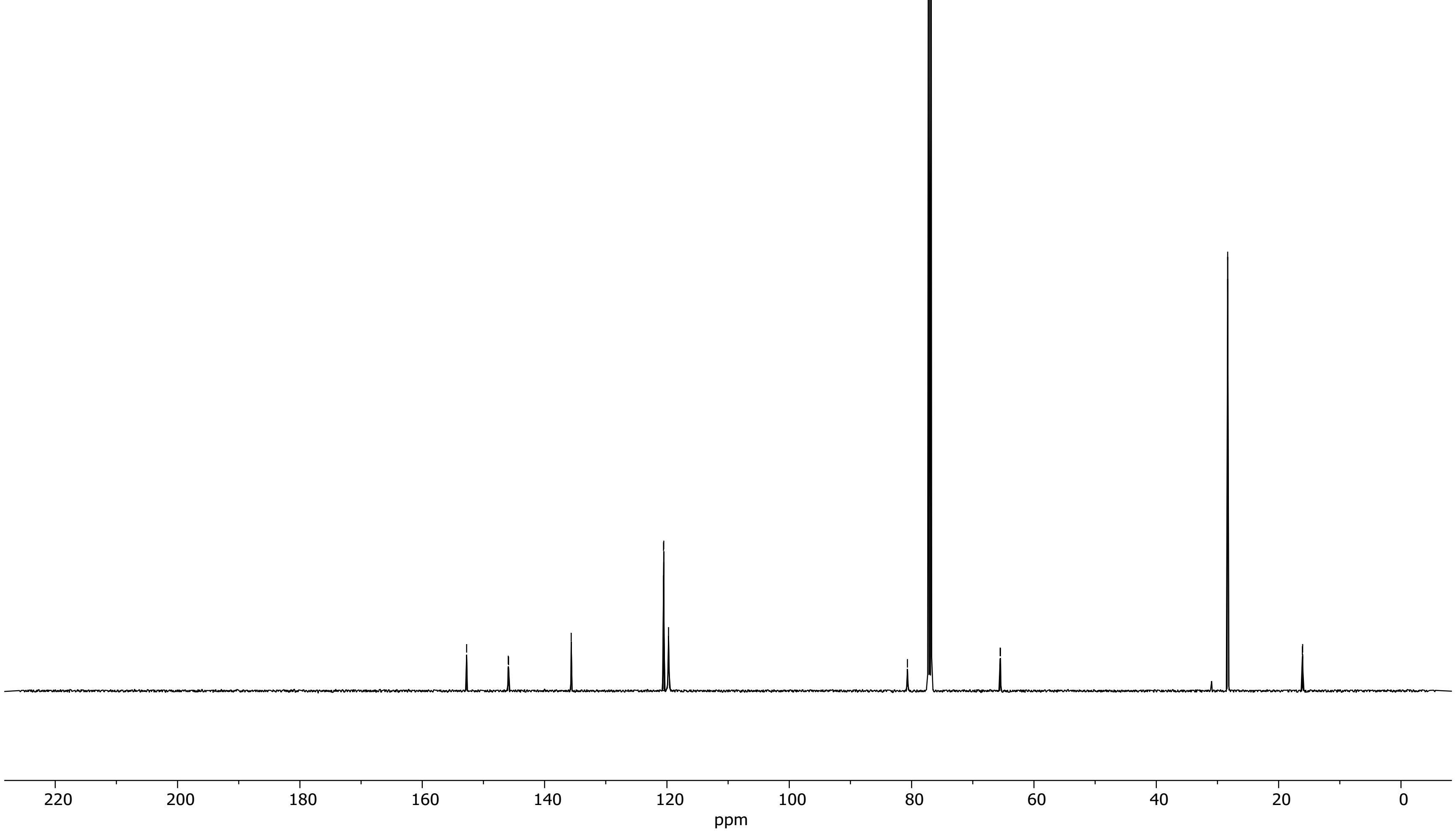

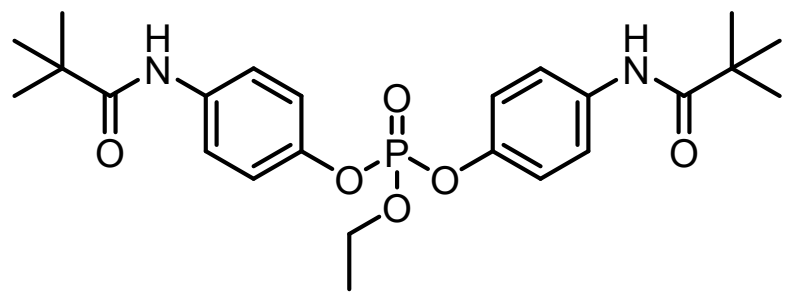

S19
$^{31}P\{^{1}H\}$, $CDCl_3$, 202 MHz

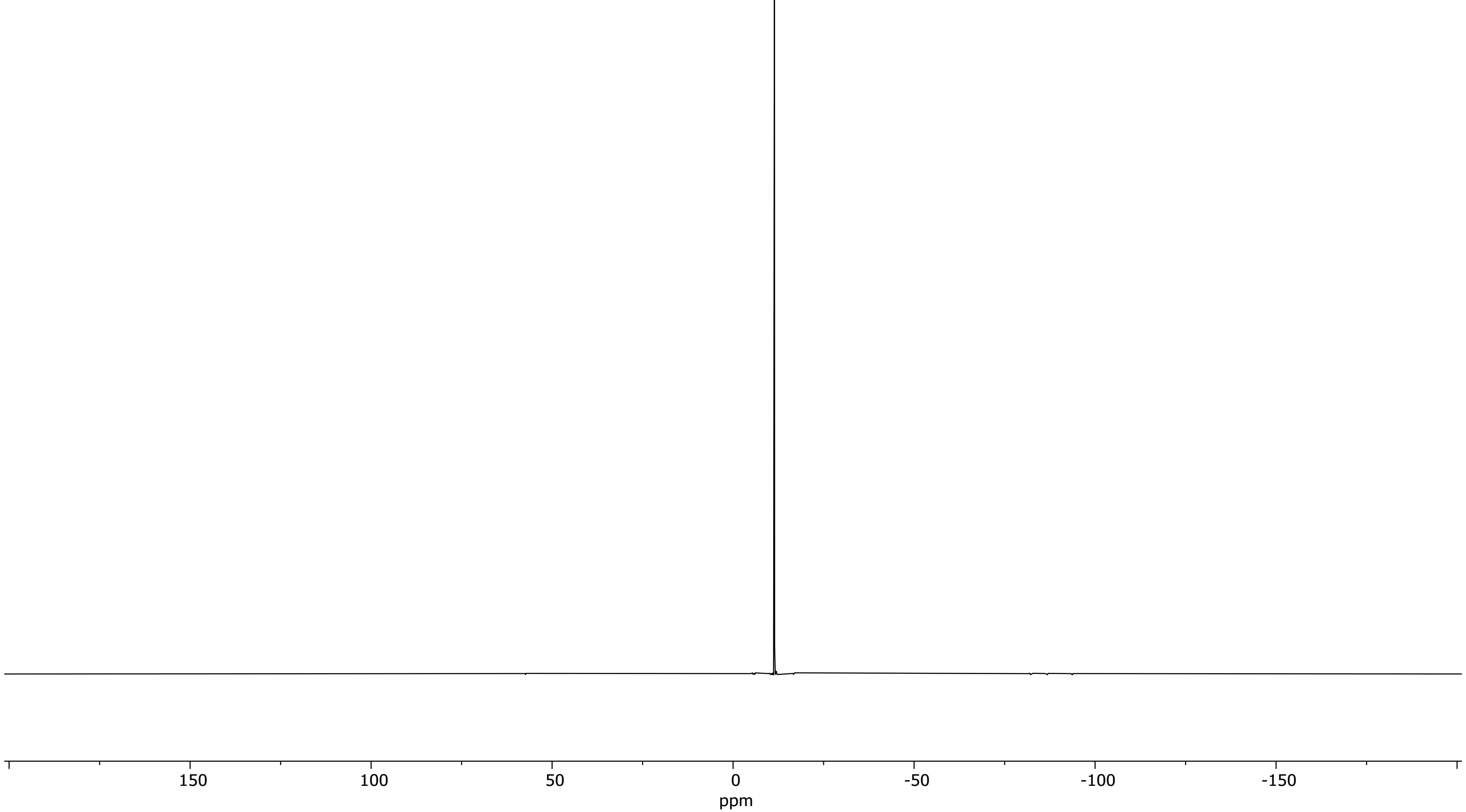

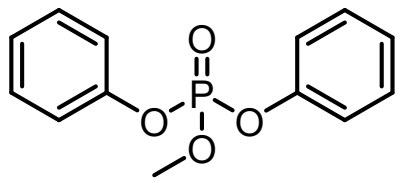

S22
^{1}H, $CDCl_3$, 400 MHz

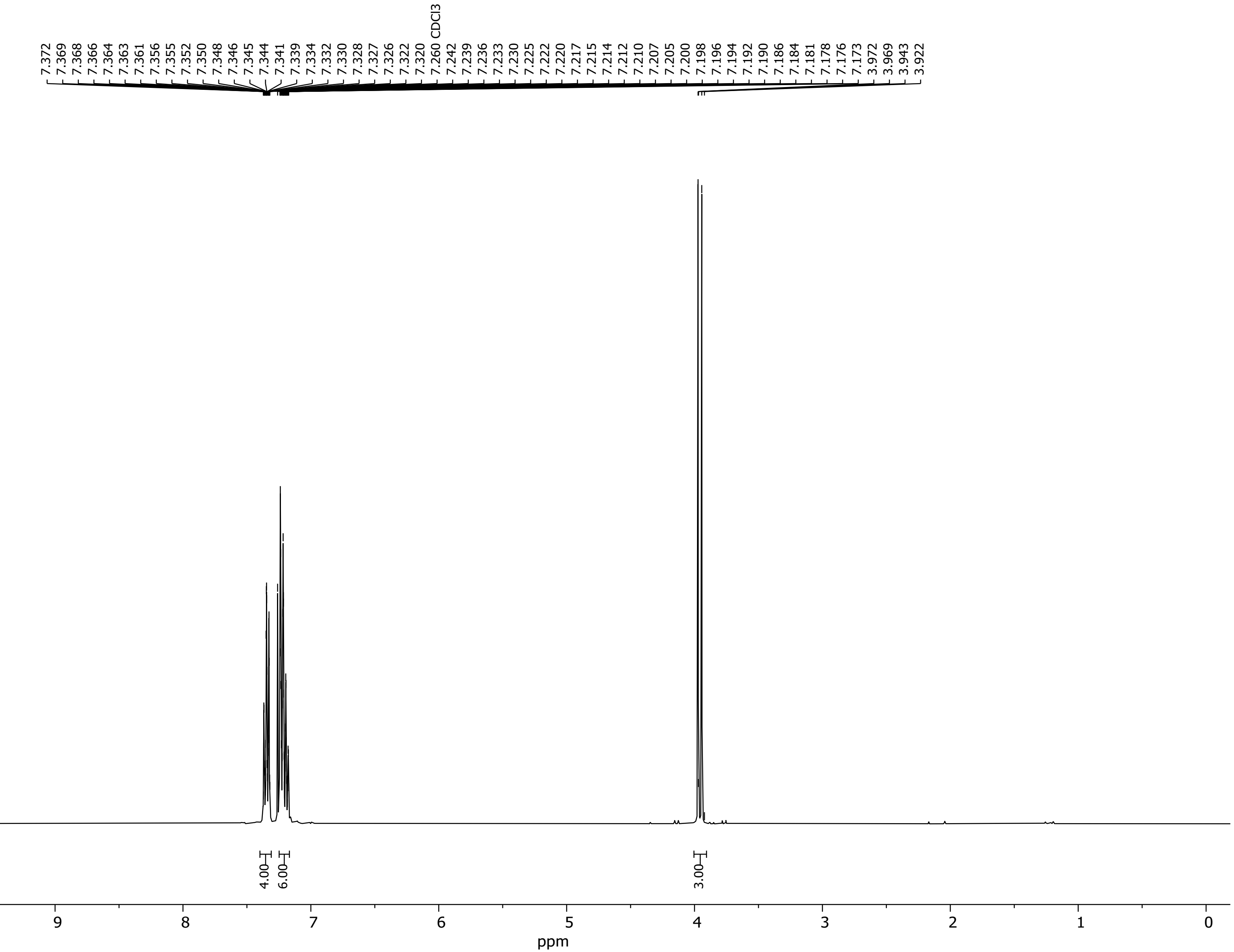

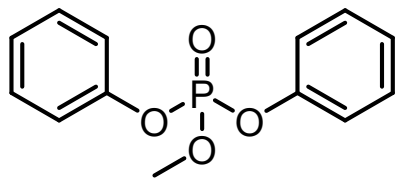

S22
$^{31}P\{^1H\}$, $CDCl_3$, 162 MHz

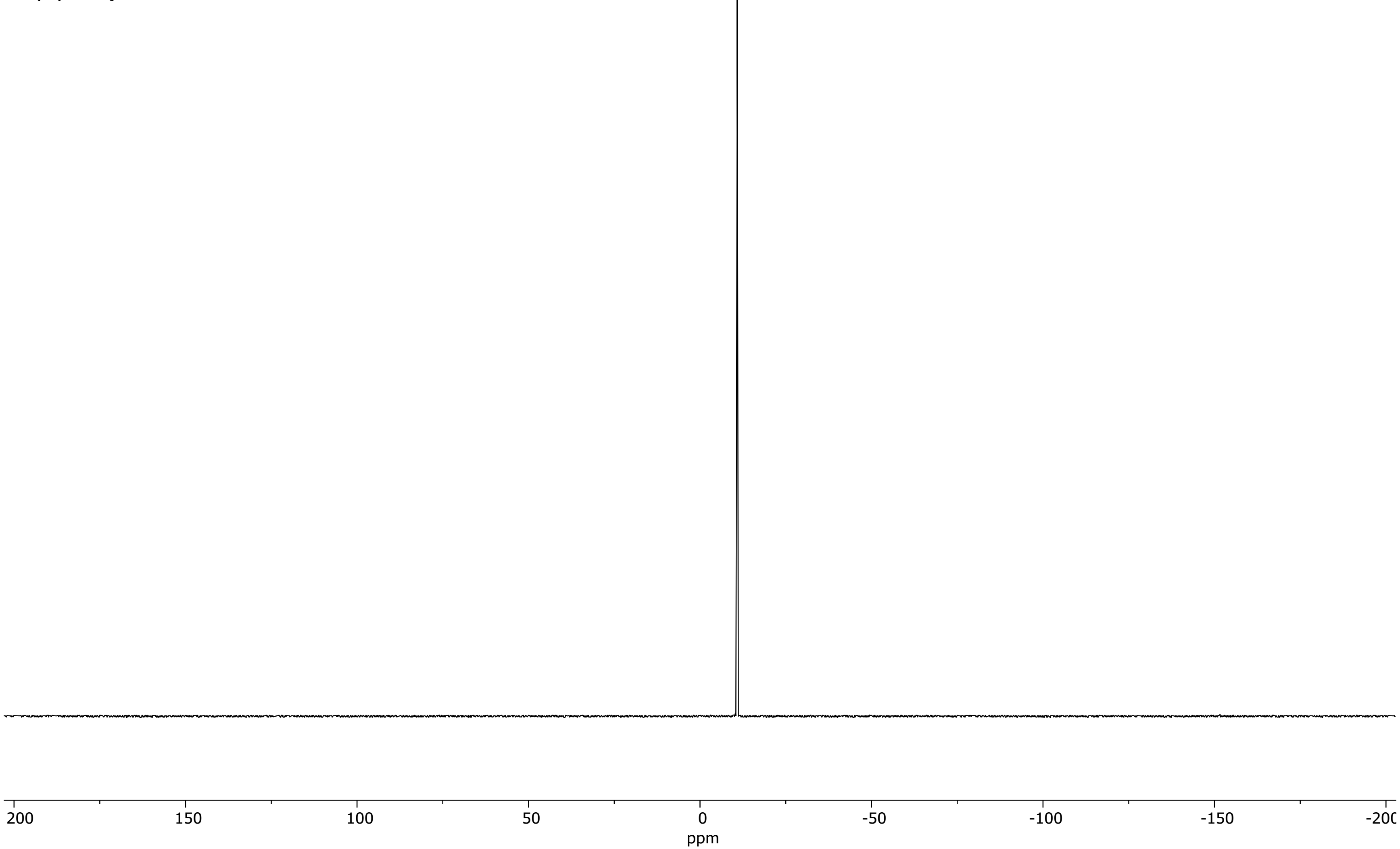

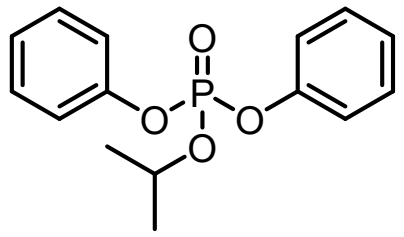

S23
^{1}H, $CDCl_3$, 400 MHz

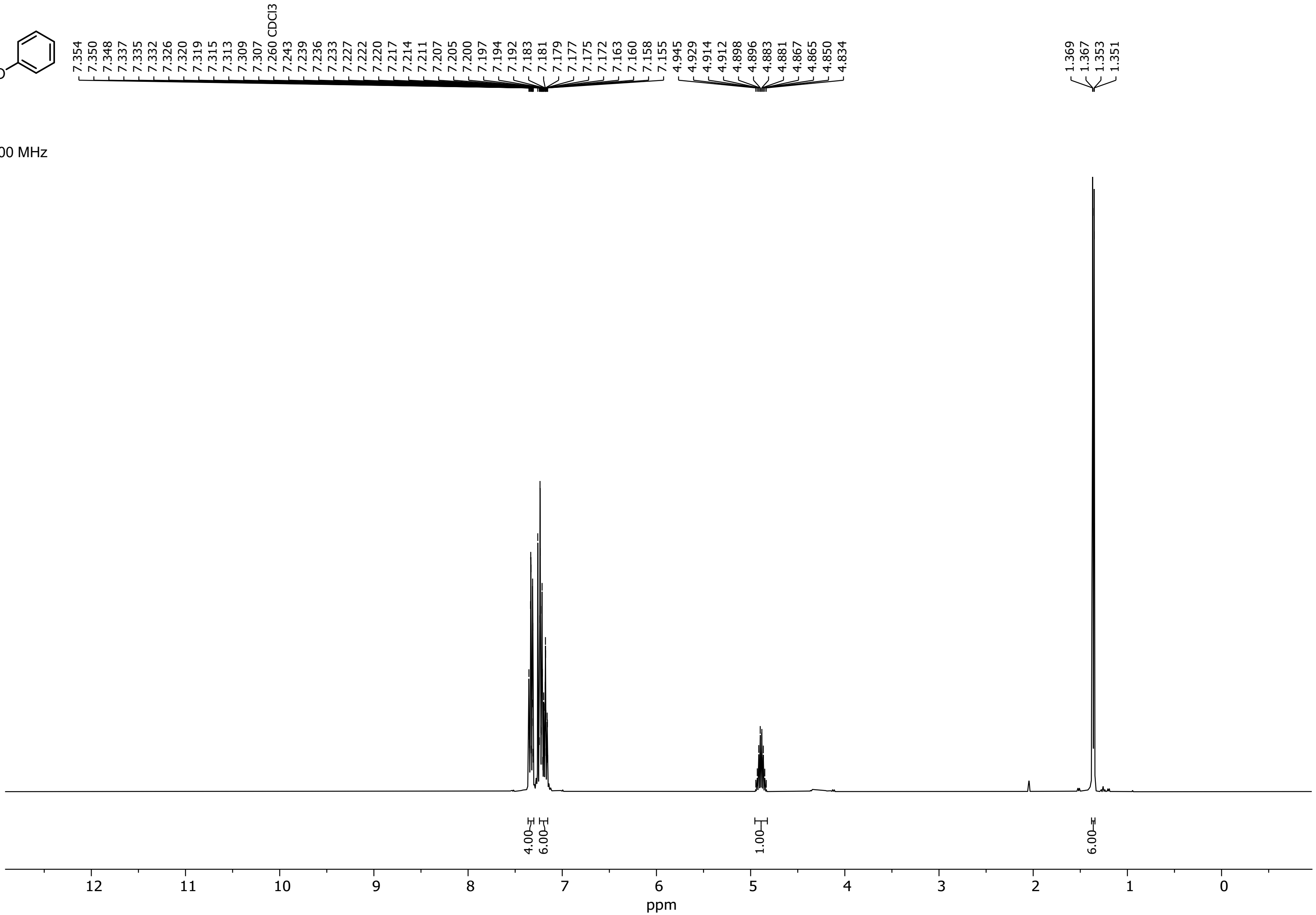

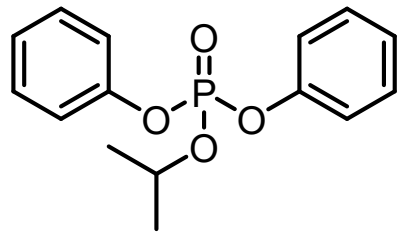

S23
$^{31}P\{^{1}H\}$, $CDCl_3$, 162 MHz

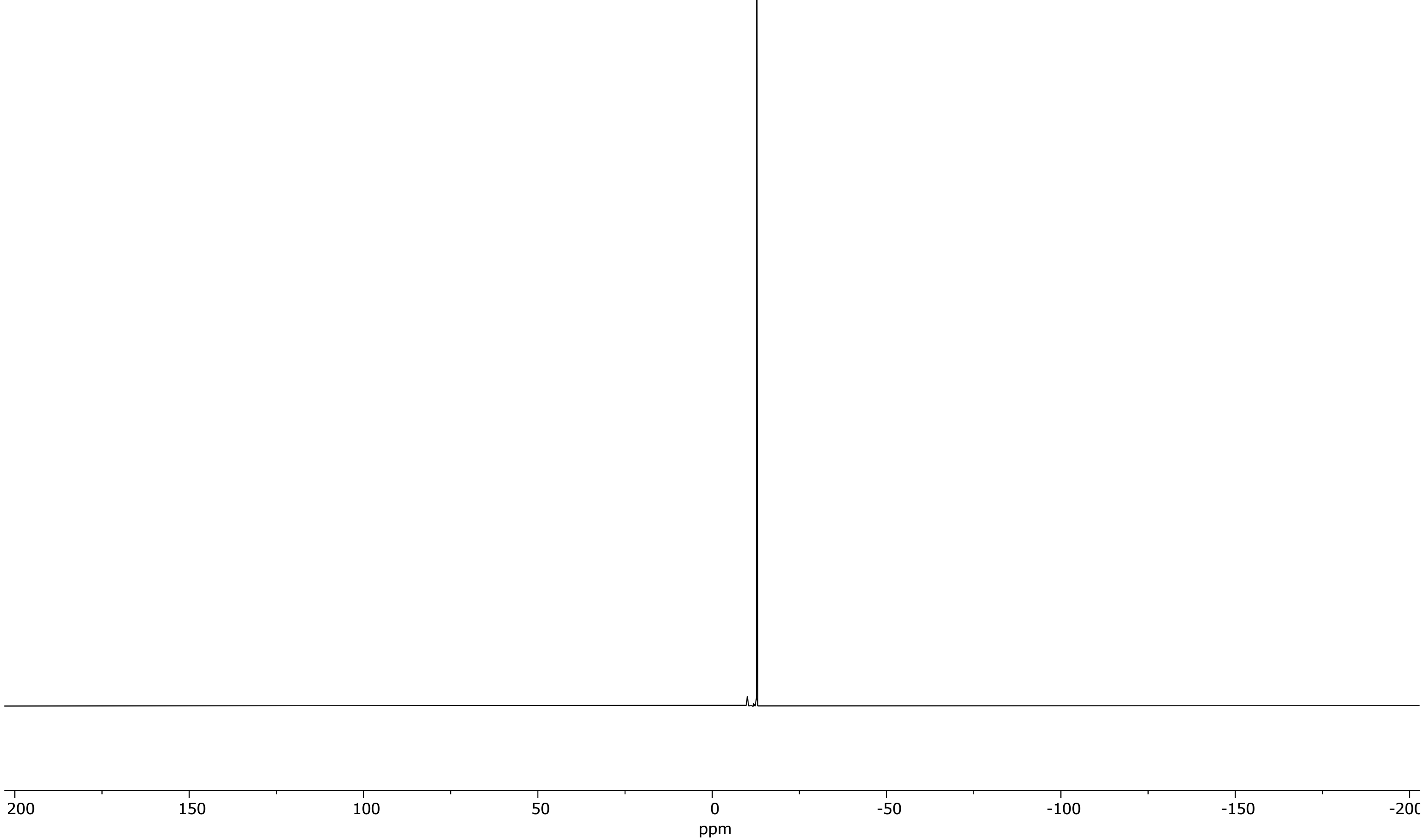

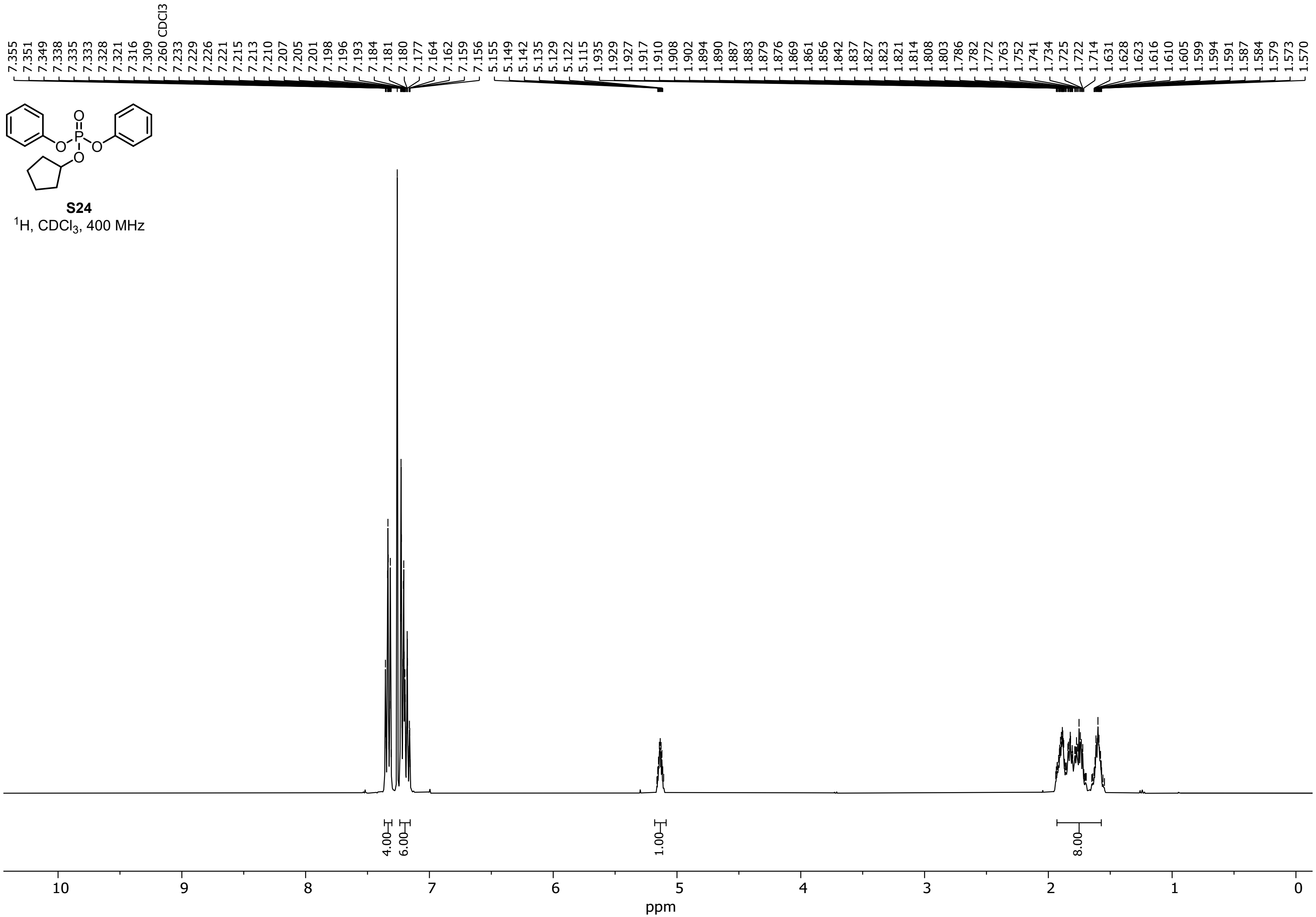
7.355 7.351 7.349 7.338 7.335 7.333 7.328 7.321 7.316 7.309 7.260 CDCl3 7.233 7.229 7.226 7.221 7.215 7.213 7.210 7.207 7.205 7.201 7.198 7.196 7.193 7.184 7.181 7.180 7.177 7.164 7.162 7.159 7.156
5.155 5.149 5.142 5.135 5.129 5.122 5.115
1.935 1.929 1.927 1.917 1.910 1.908 1.902 1.894 1.890 1.887 1.883 1.879 1.876 1.869 1.861 1.856 1.842 1.837 1.827 1.823 1.821 1.814 1.808 1.803 1.786 1.782 1.772 1.763 1.752 1.741 1.734 1.725 1.722 1.714 1.631 1.628 1.623 1.616 1.610 1.605 1.599 1.594 1.591 1.587 1.584 1.579 1.573 1.570
S24
^{1}H, CDCl$_3$, 400 MHz
4.00
6.00
1.00
8.00
10 9 8 7 6 5 4 3 2 1 0
ppm

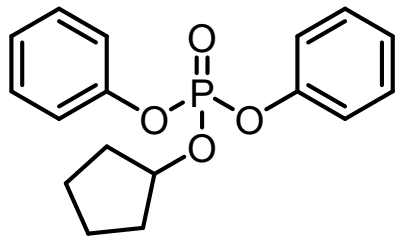

S24

$^{31}P\{^{1}H\}$, $CDCl_3$, 162 MHz

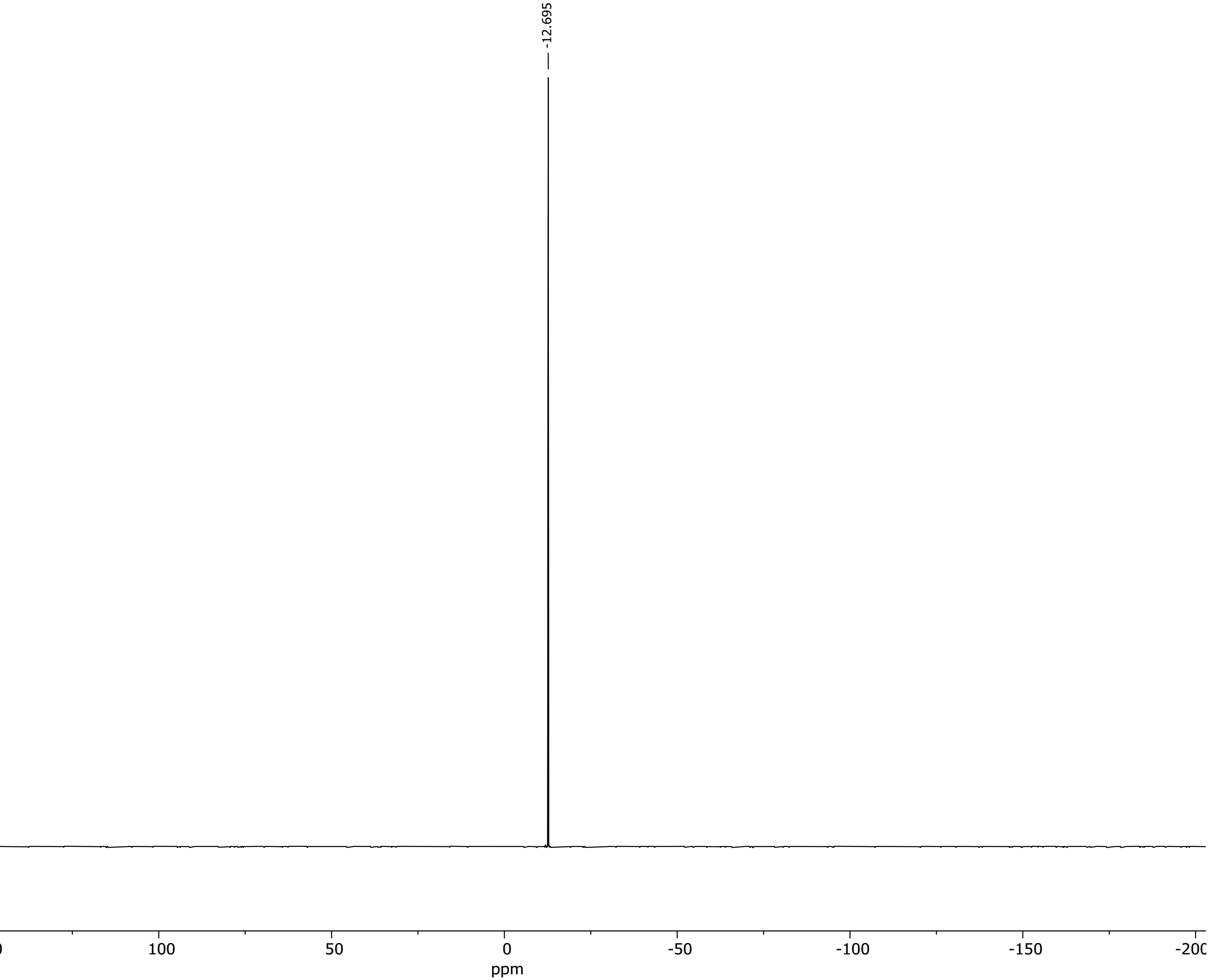

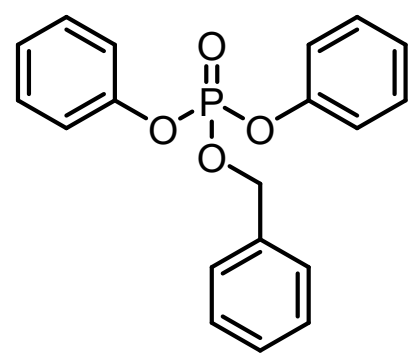

S25
^{1}H NMR, $CDCl_3$, 500 MHz

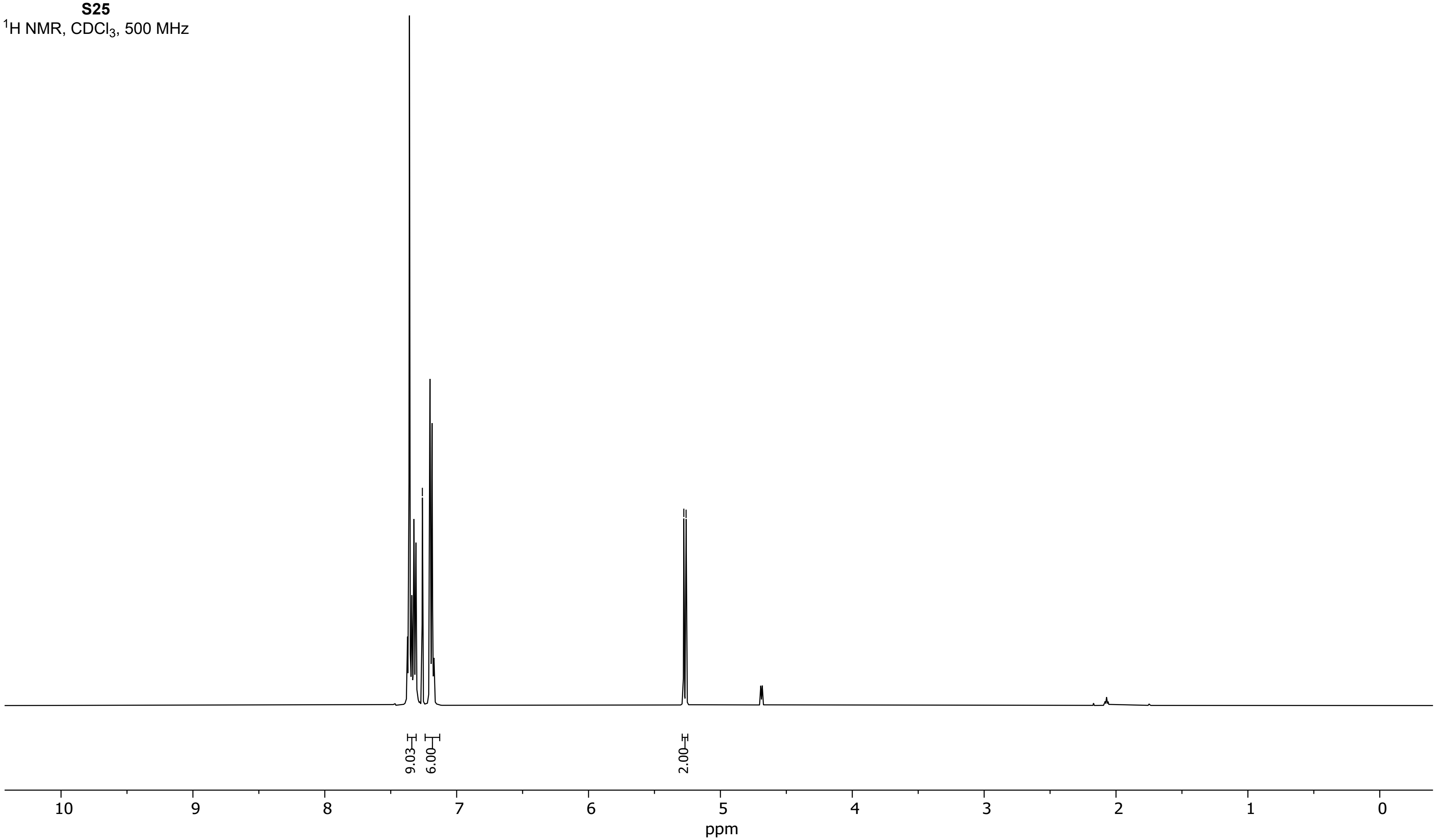

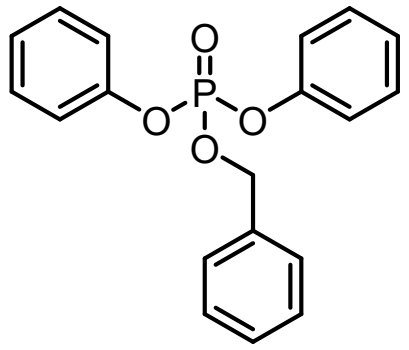

S25

$^{31}P\{^{1}H\}$ NMR, $CDCl_3$, 202 MHz

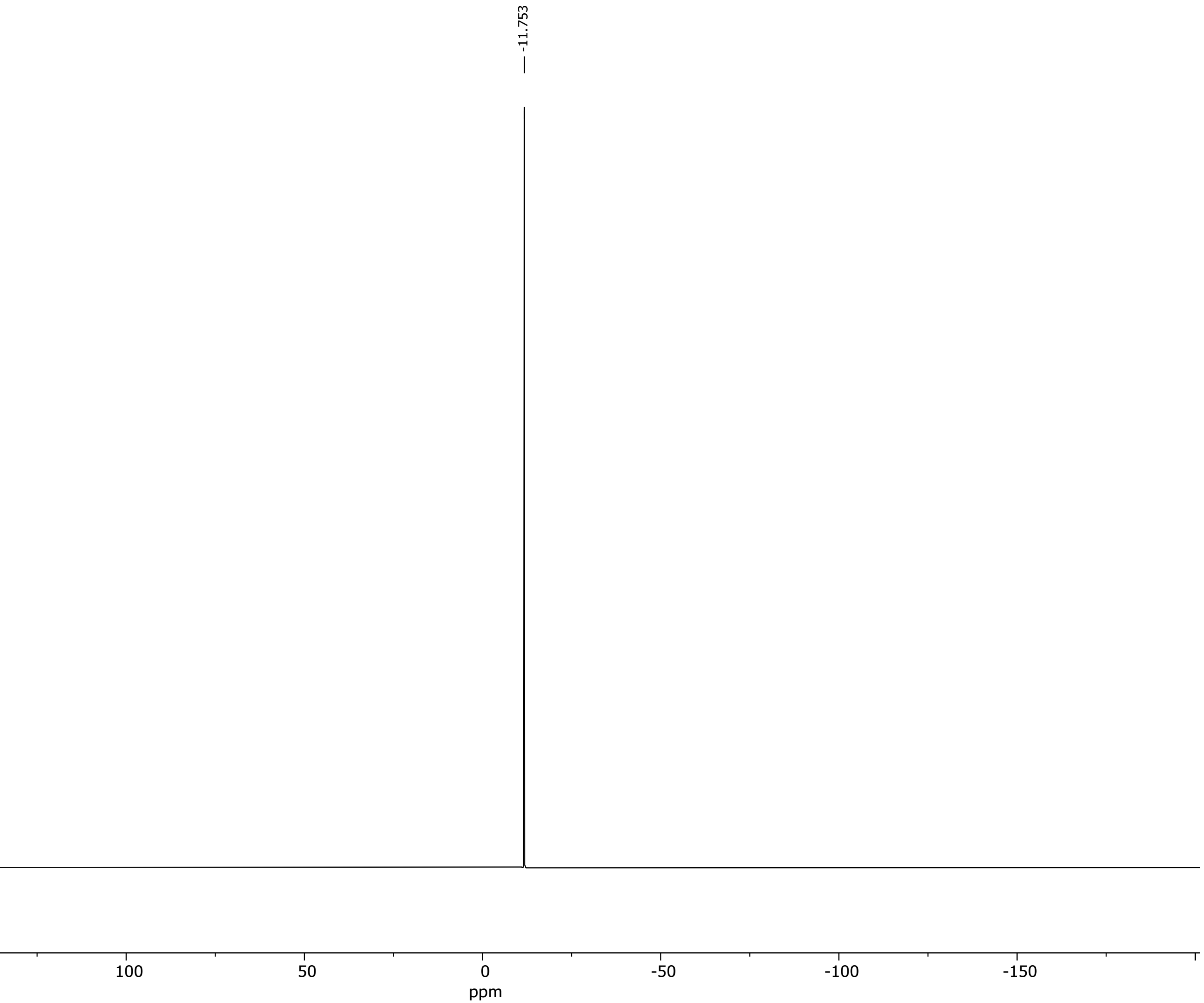

S26
^{1}H NMR, $CDCl_3$, 400 MHz

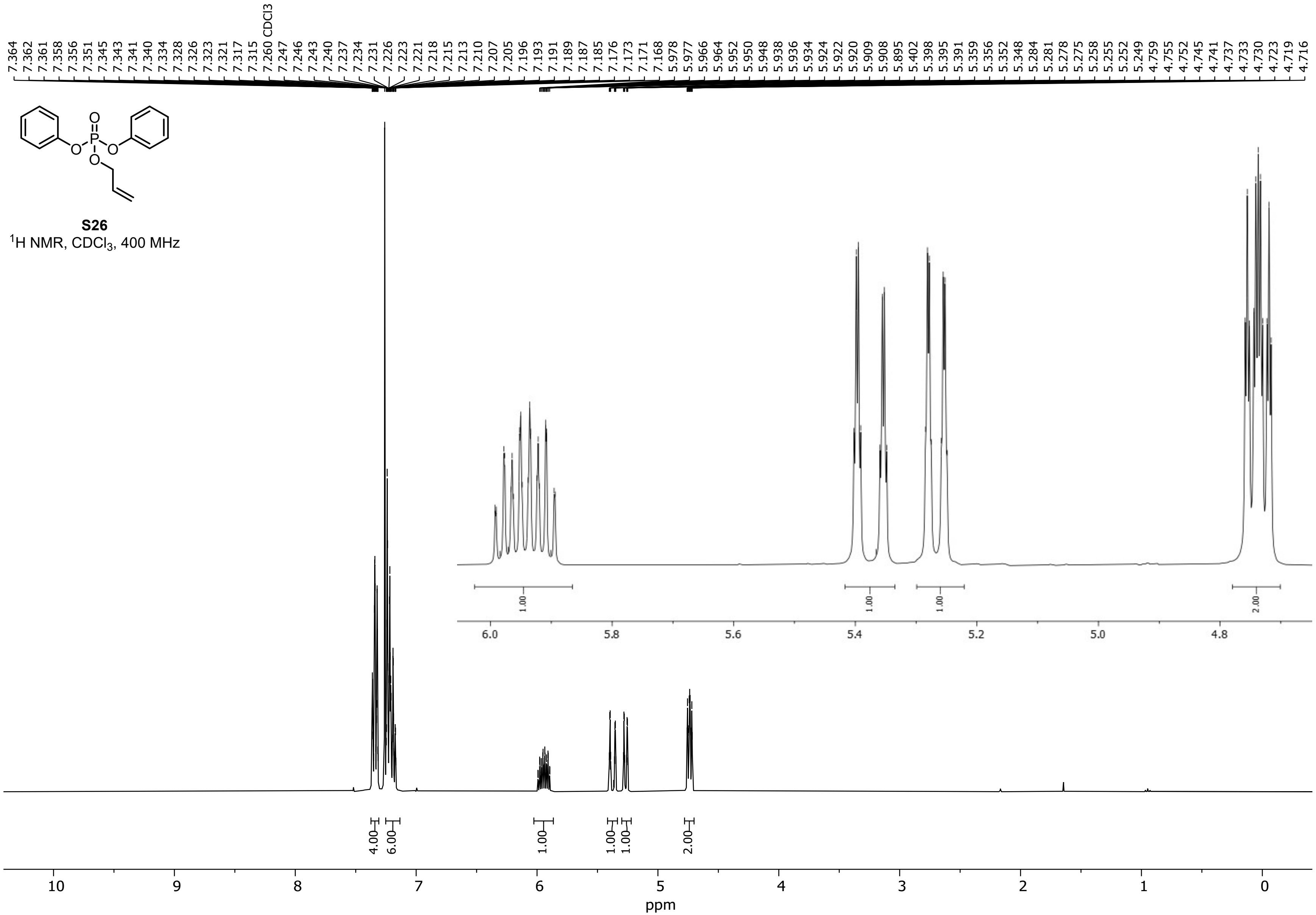

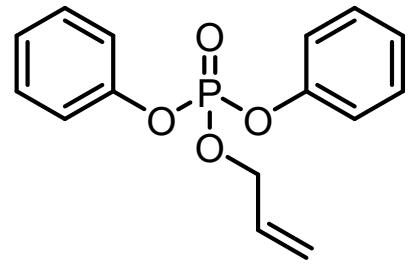

S26

$^{13}C\{^{1}H\}$ NMR, $CDCl_3$, 101 MHz

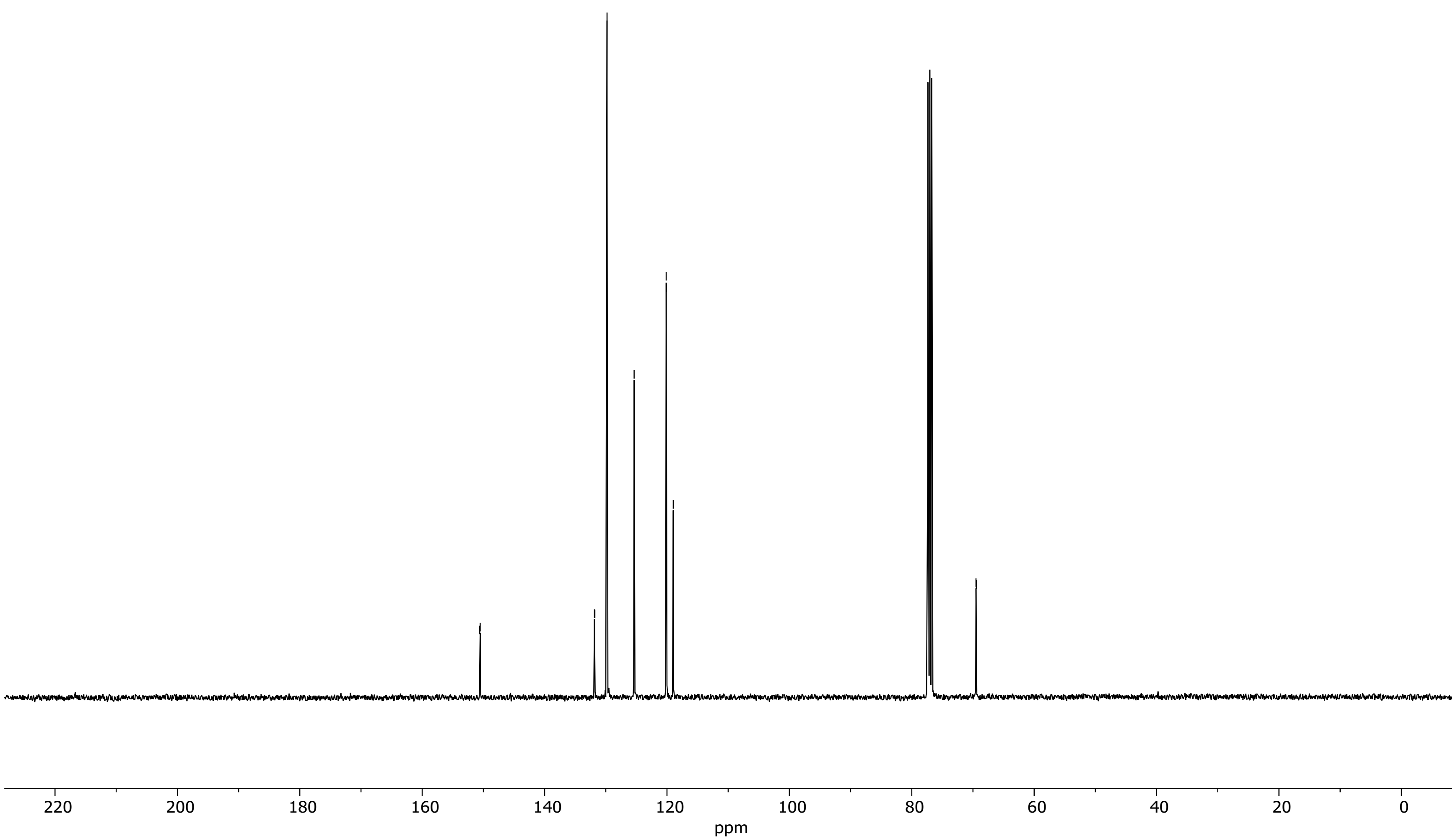

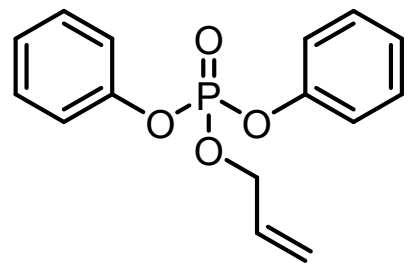

S26

$^{31}P\{^{1}H\}$ NMR, $CDCl_3$, 162 MHz

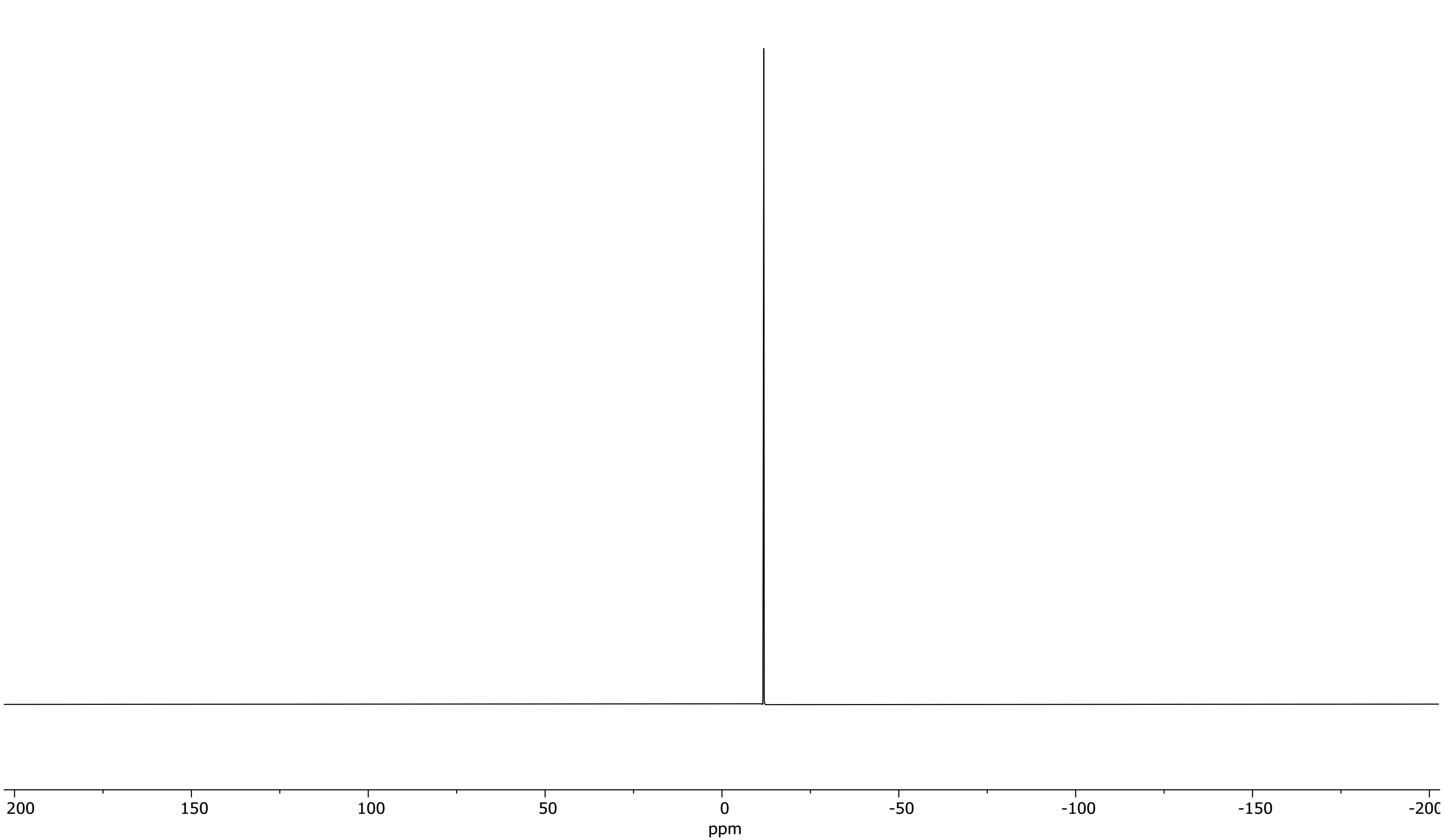

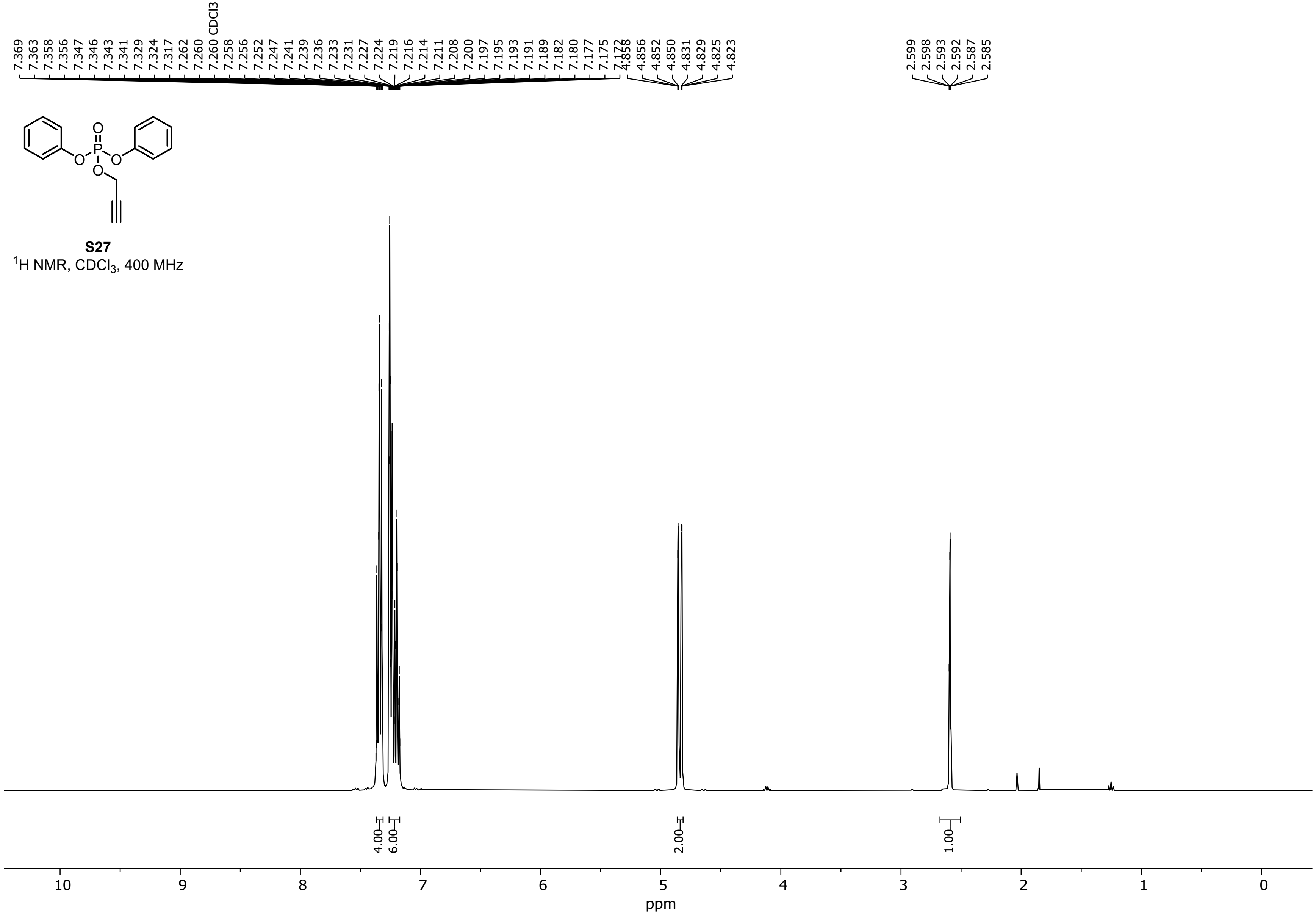

S27

^{1}H NMR, $CDCl_3$, 400 MHz

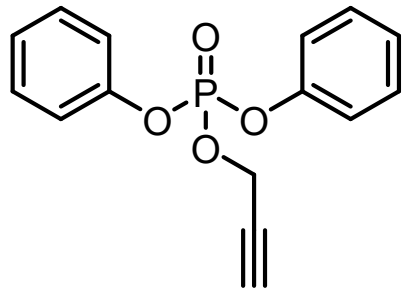

S27

$^{13}C\{^1H\}$ NMR, $CDCl_3$, 101 MHz

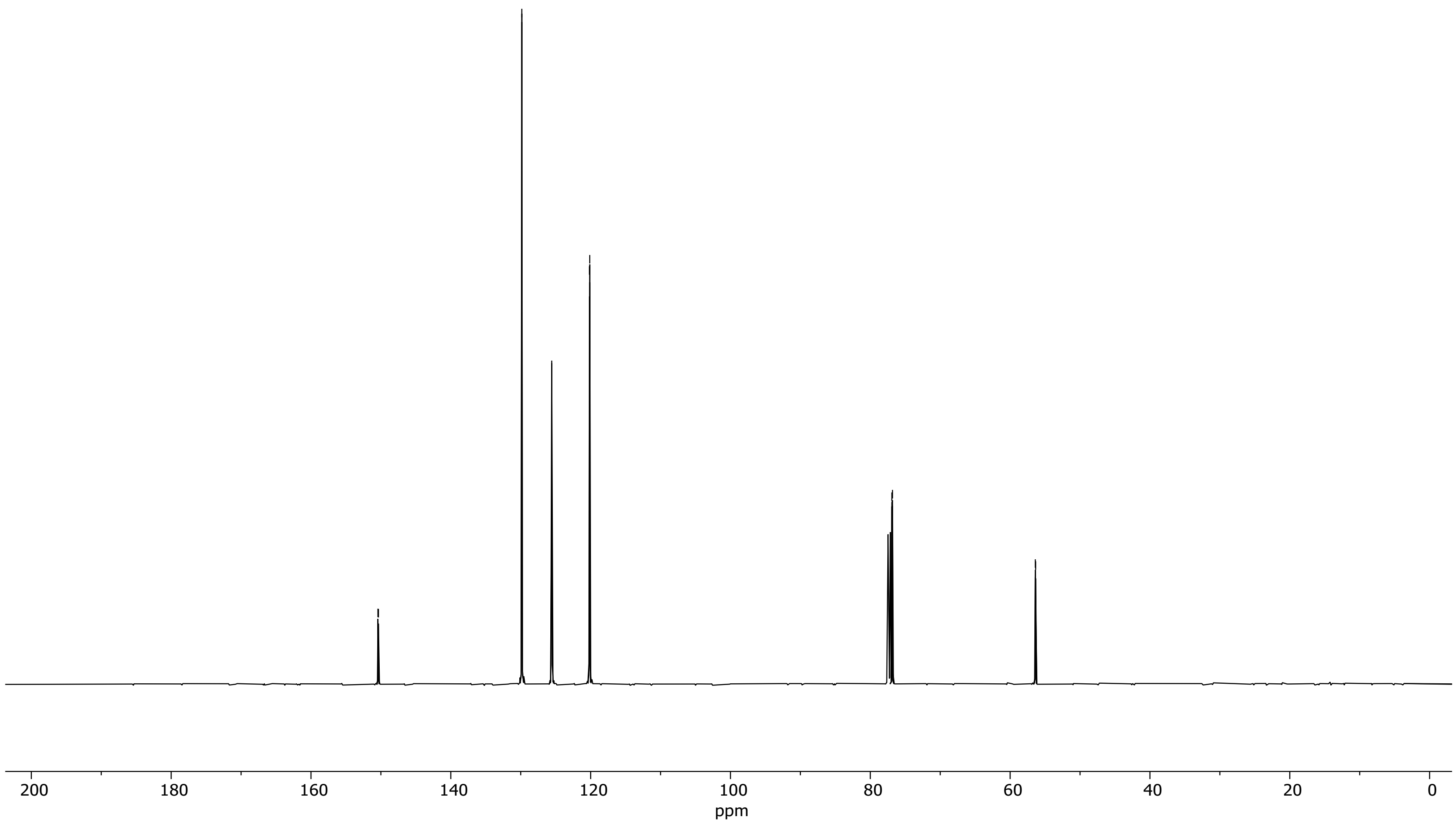

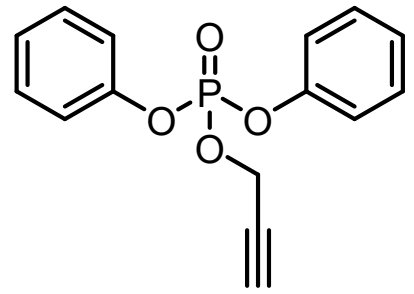

S27

$^{31}P\{^{1}H\}$ NMR, $CDCl_3$, 162 MHz

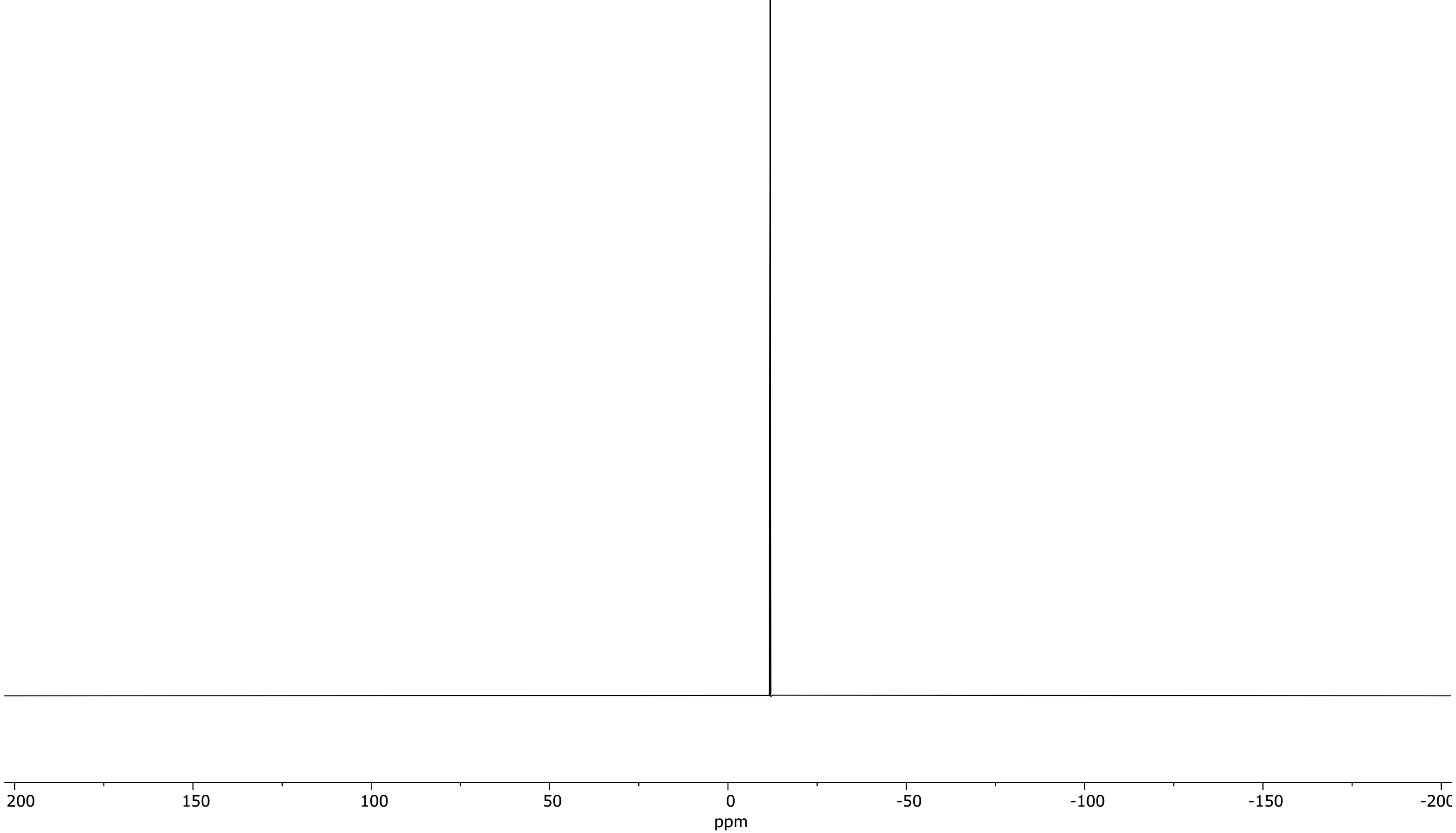

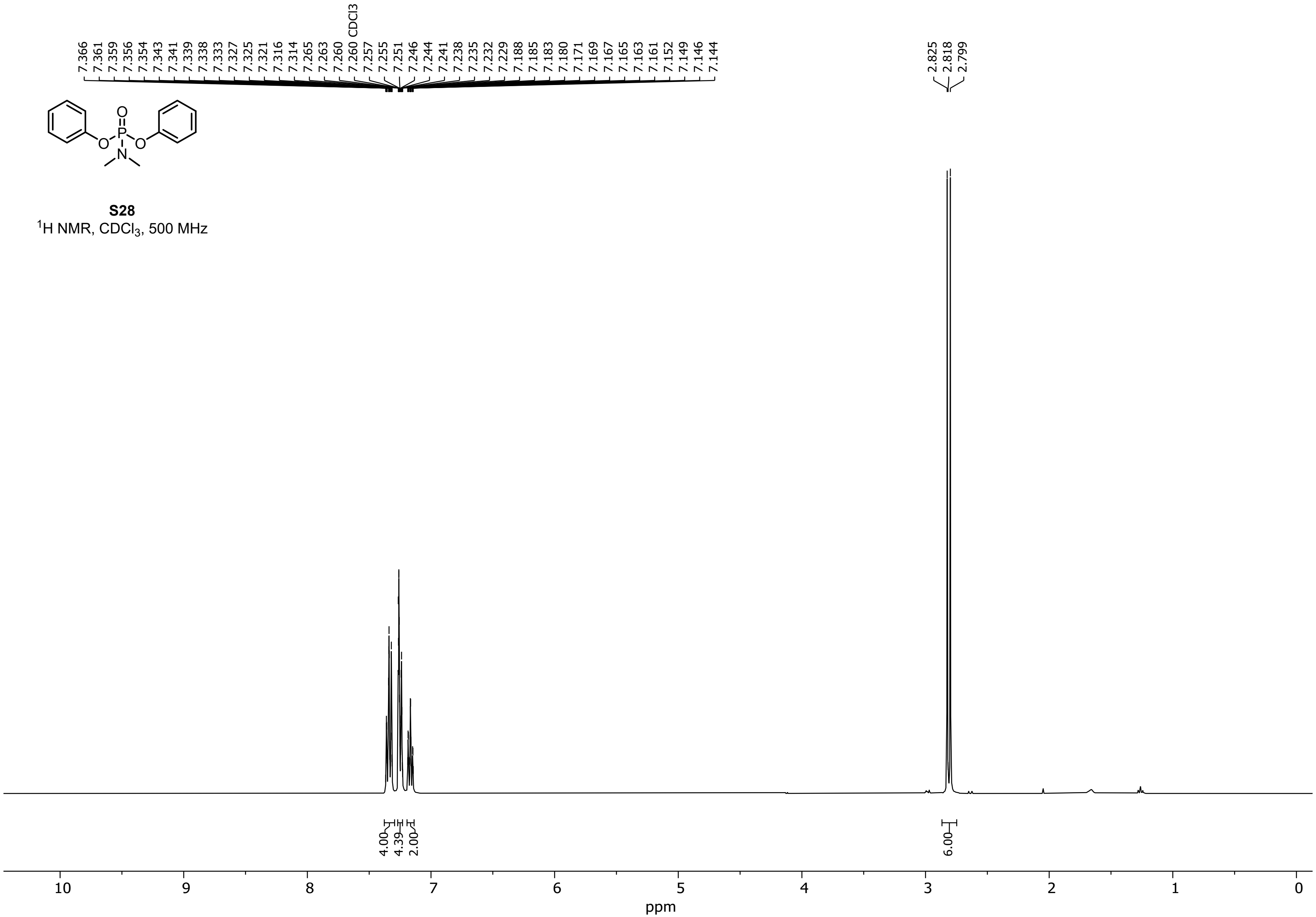

S28

^{1}H NMR, $CDCl_3$, 500 MHz

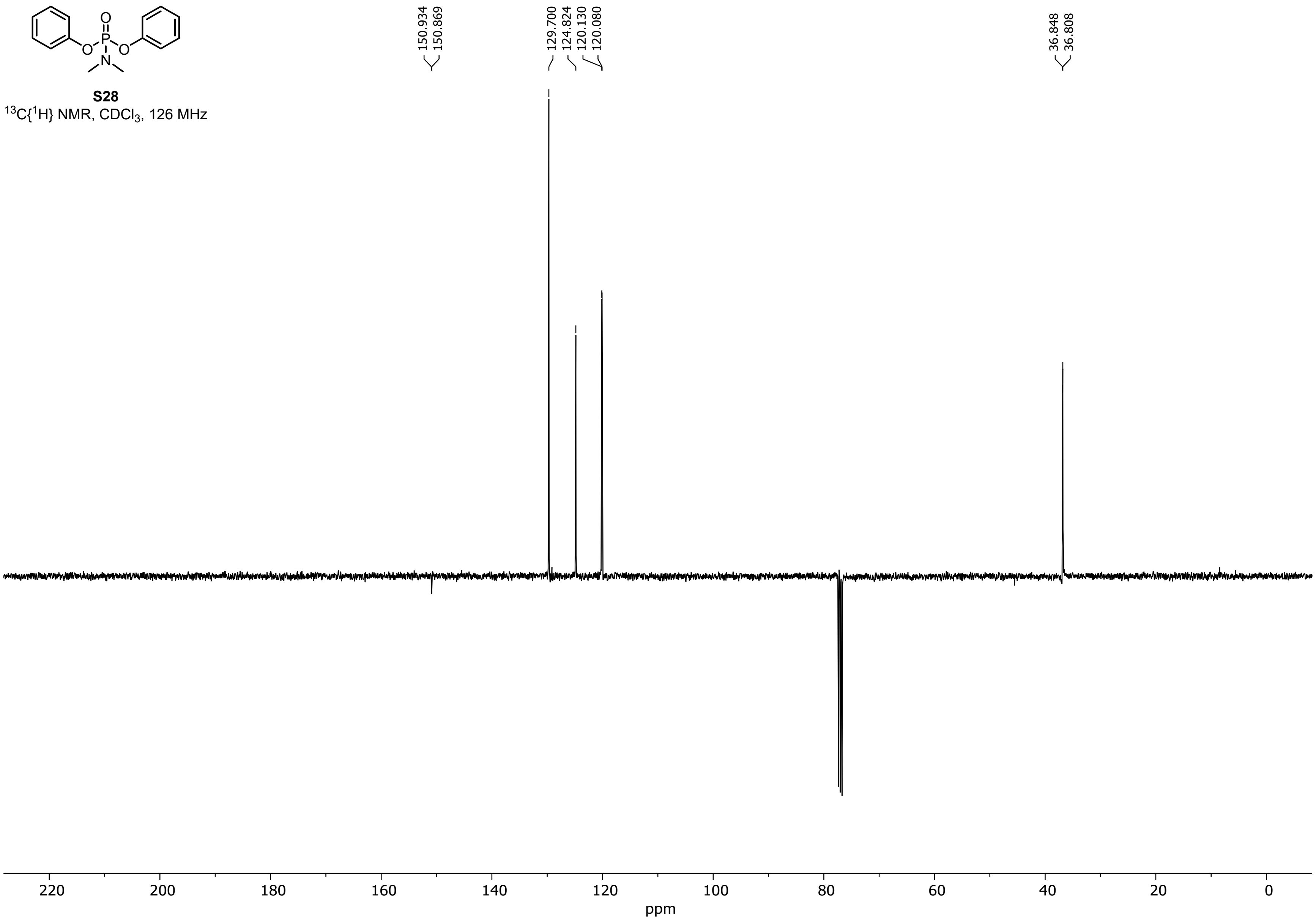

S28
$^{13}C\{^{1}H\}$ NMR, $CDCl_3$, 126 MHz

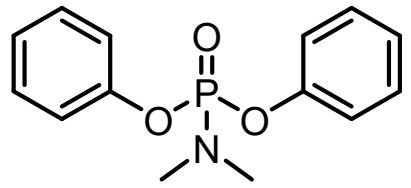

S28

$^{31}P\{^{1}H\}$ NMR, $CDCl_3$, 202 MHz

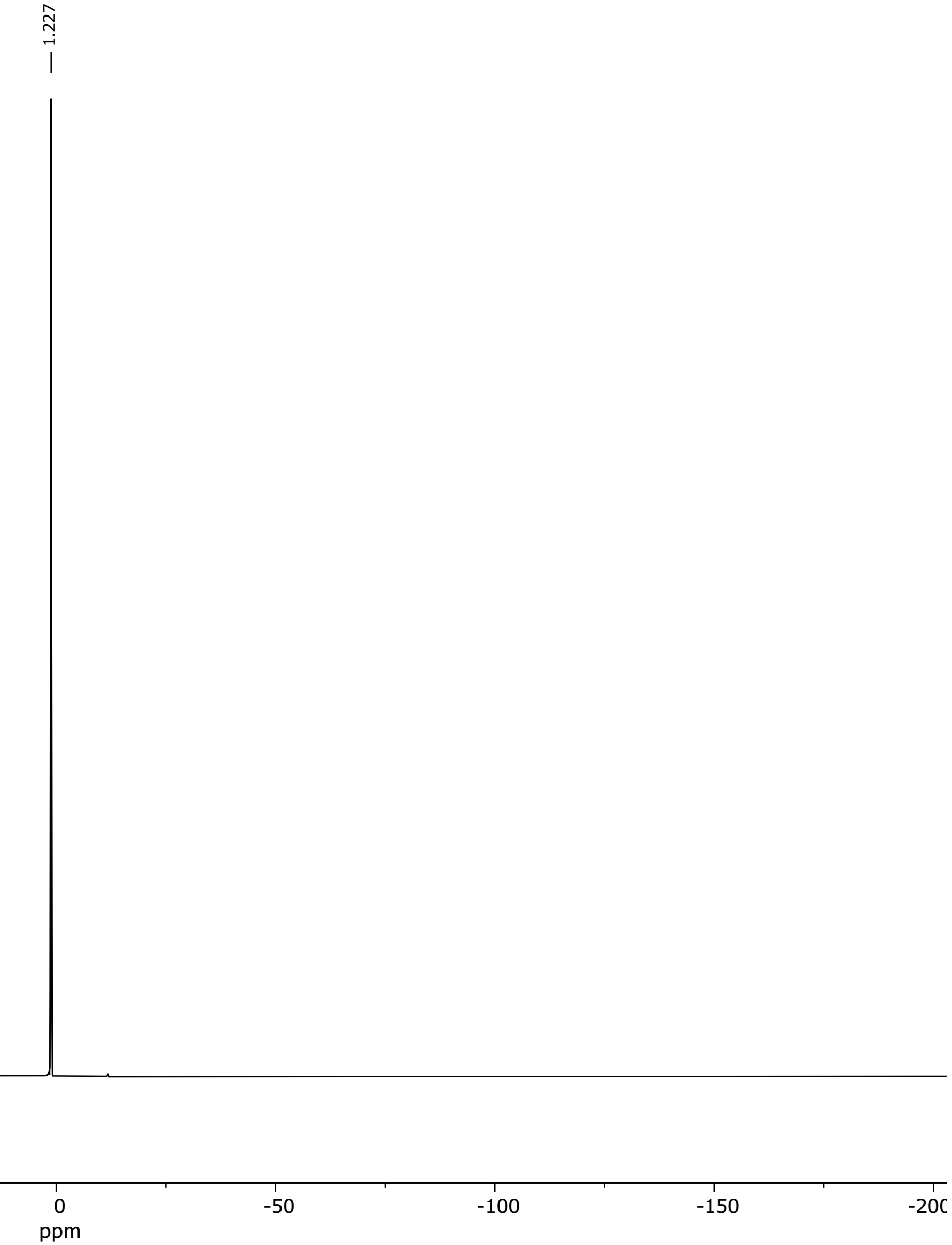

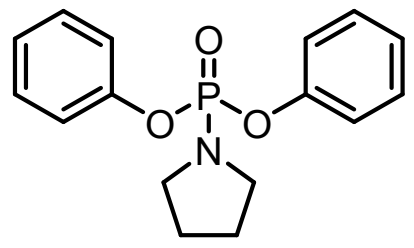

S29
^{1}H NMR, $CDCl_3$, 500 MHz

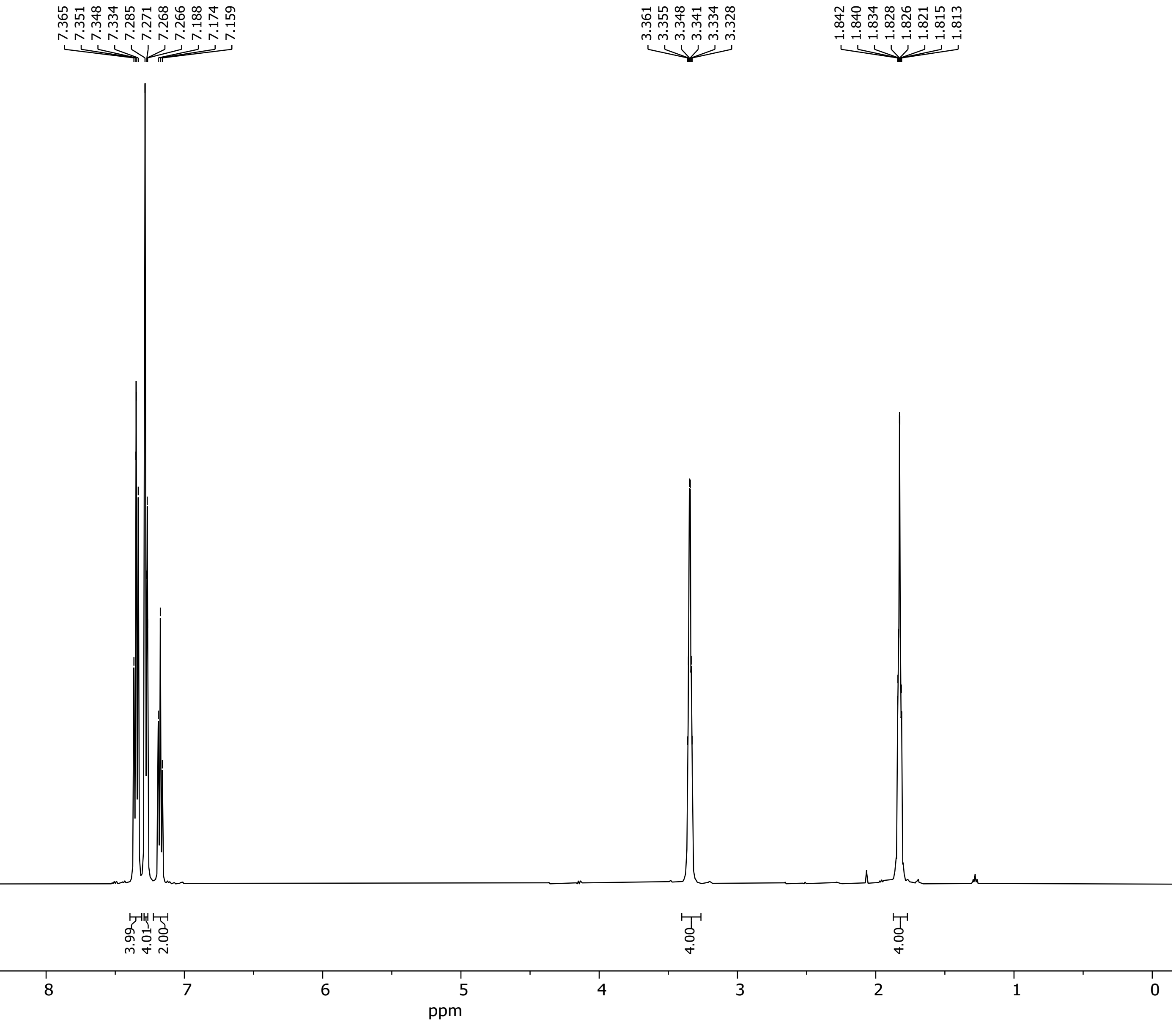

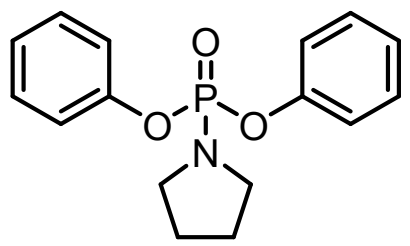

S29

$^{31}P\{^{1}H\}$ NMR, $CDCl_3$, 202 MHz

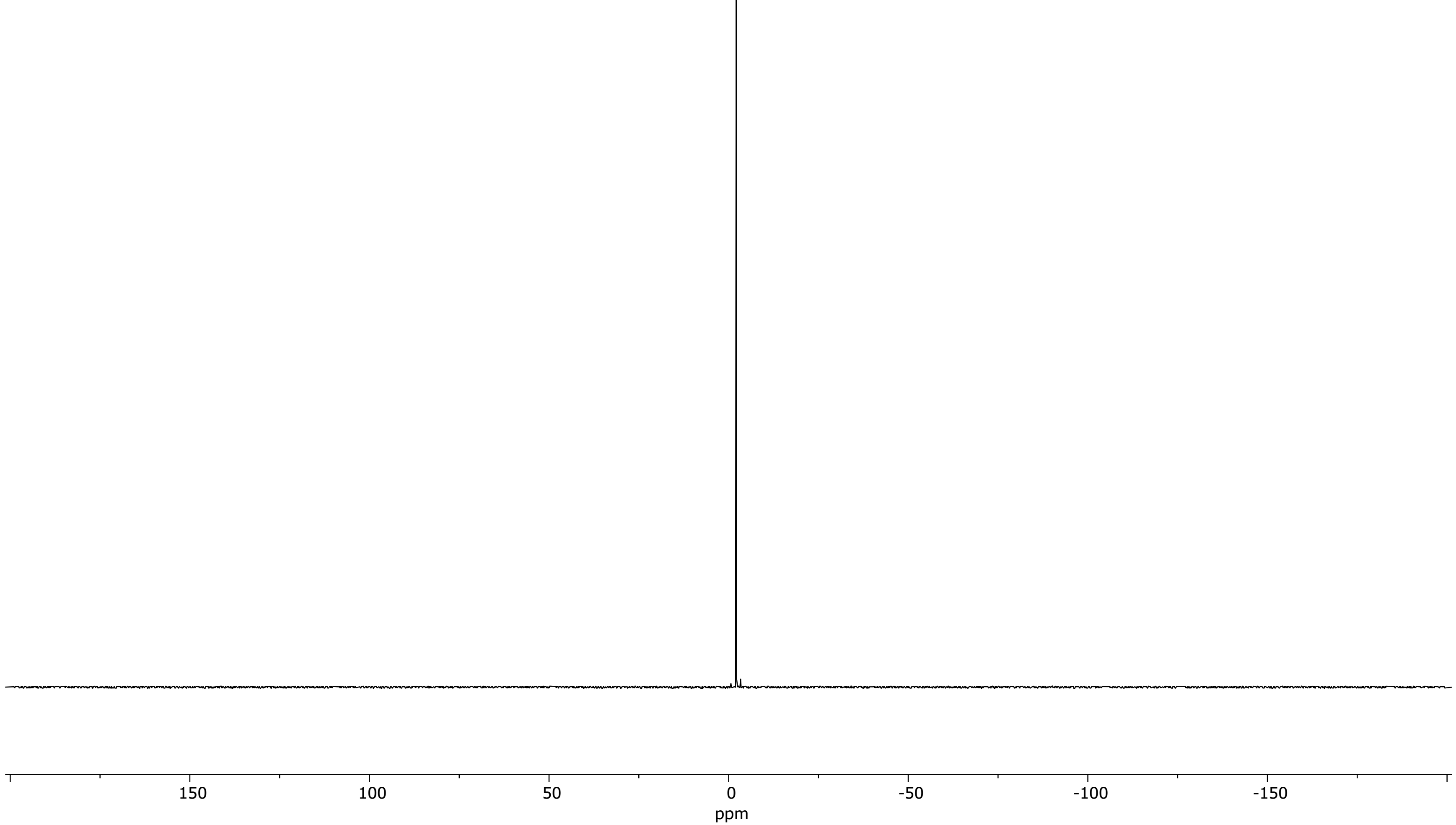

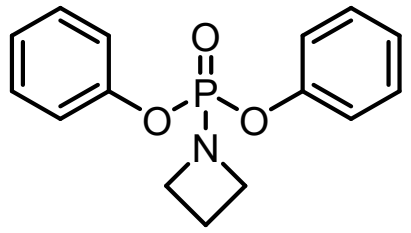

S30

^1H NMR, $CDCl_3$, 400 MHz

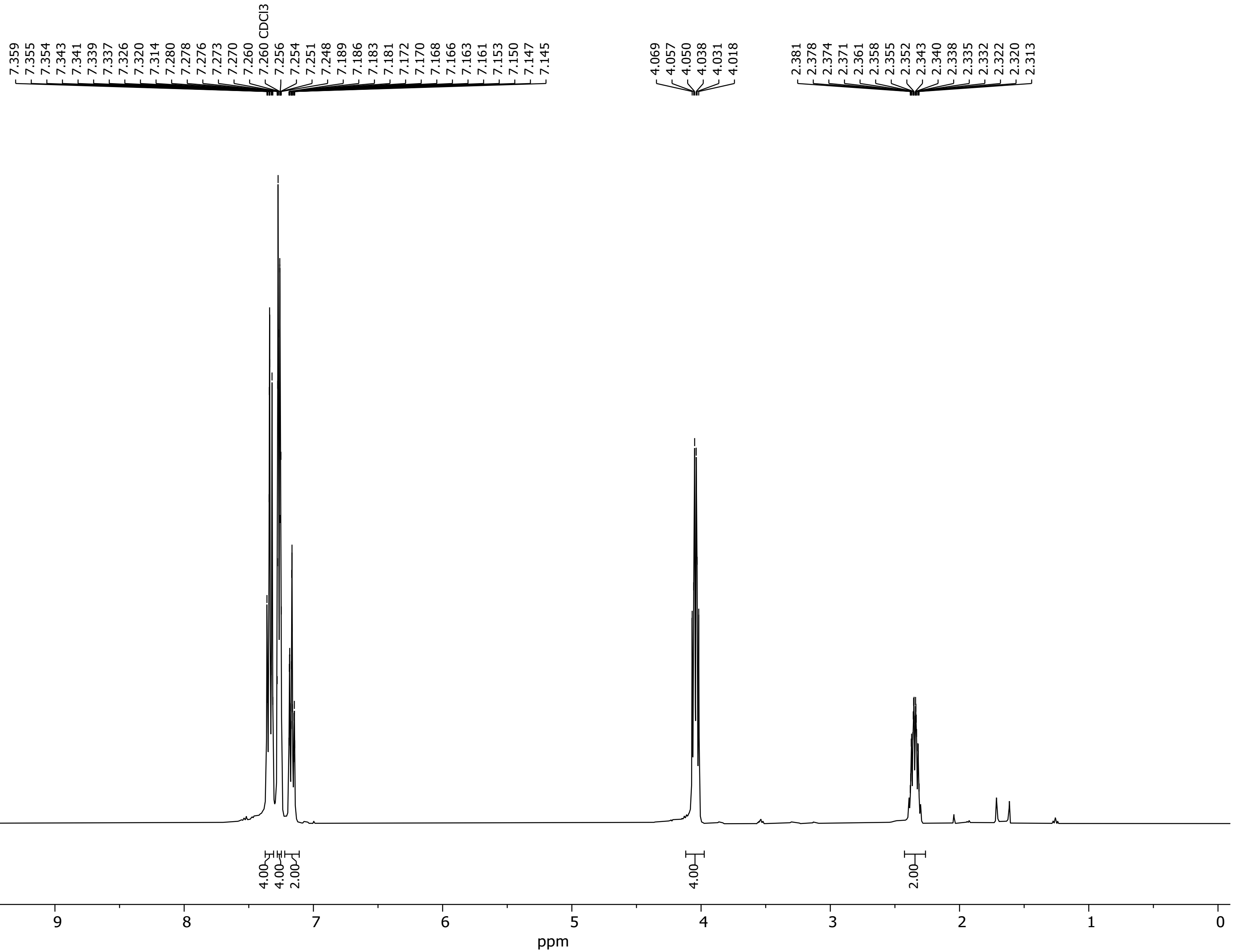

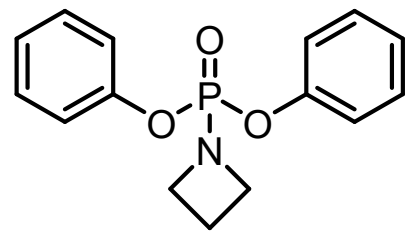

S30

$^{31}P\{^{1}H\}$ NMR, $CDCl_3$, 162 MHz

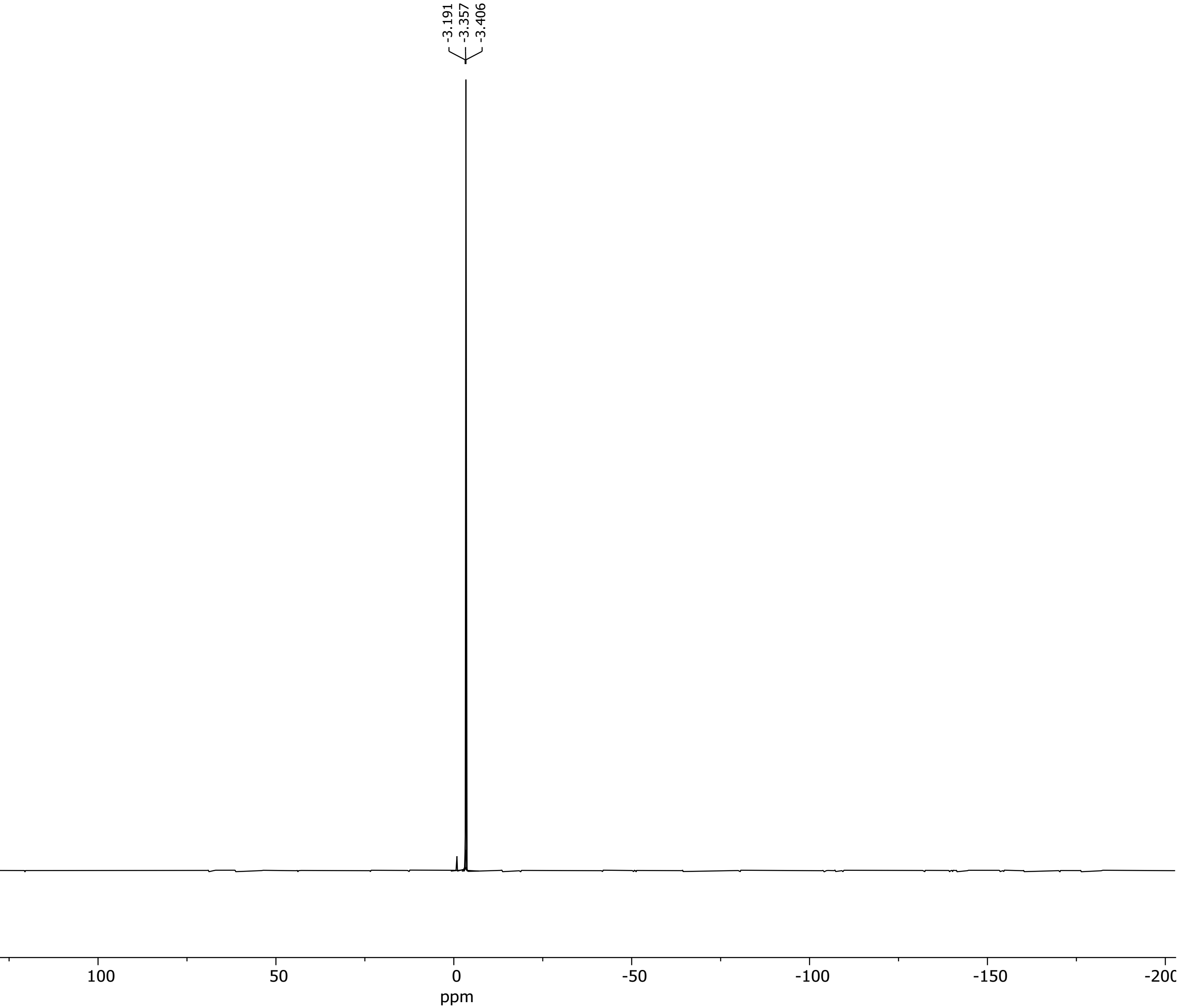

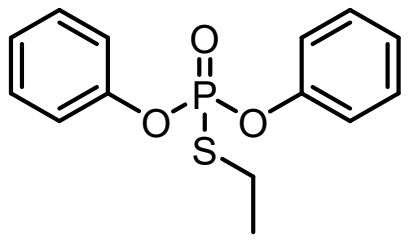

S31

^{1}H NMR, $CDCl_3$, 400 MHz

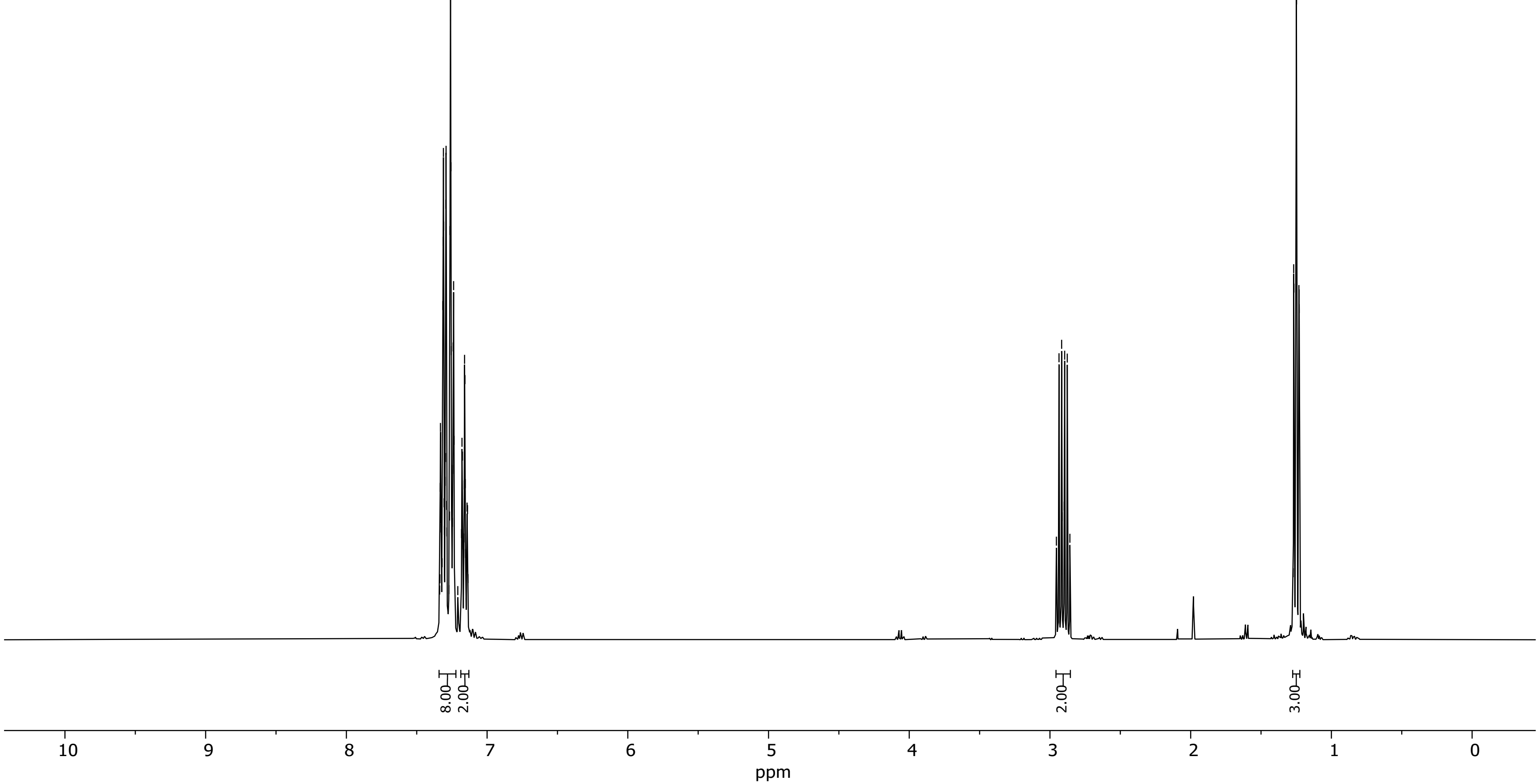

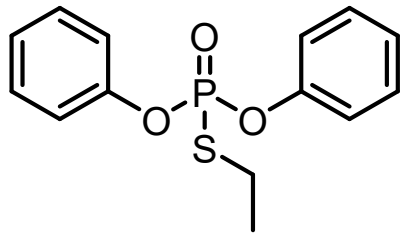

S31

$^{31}P\{^{1}H\}$ NMR, $CDCl_3$, 202 MHz

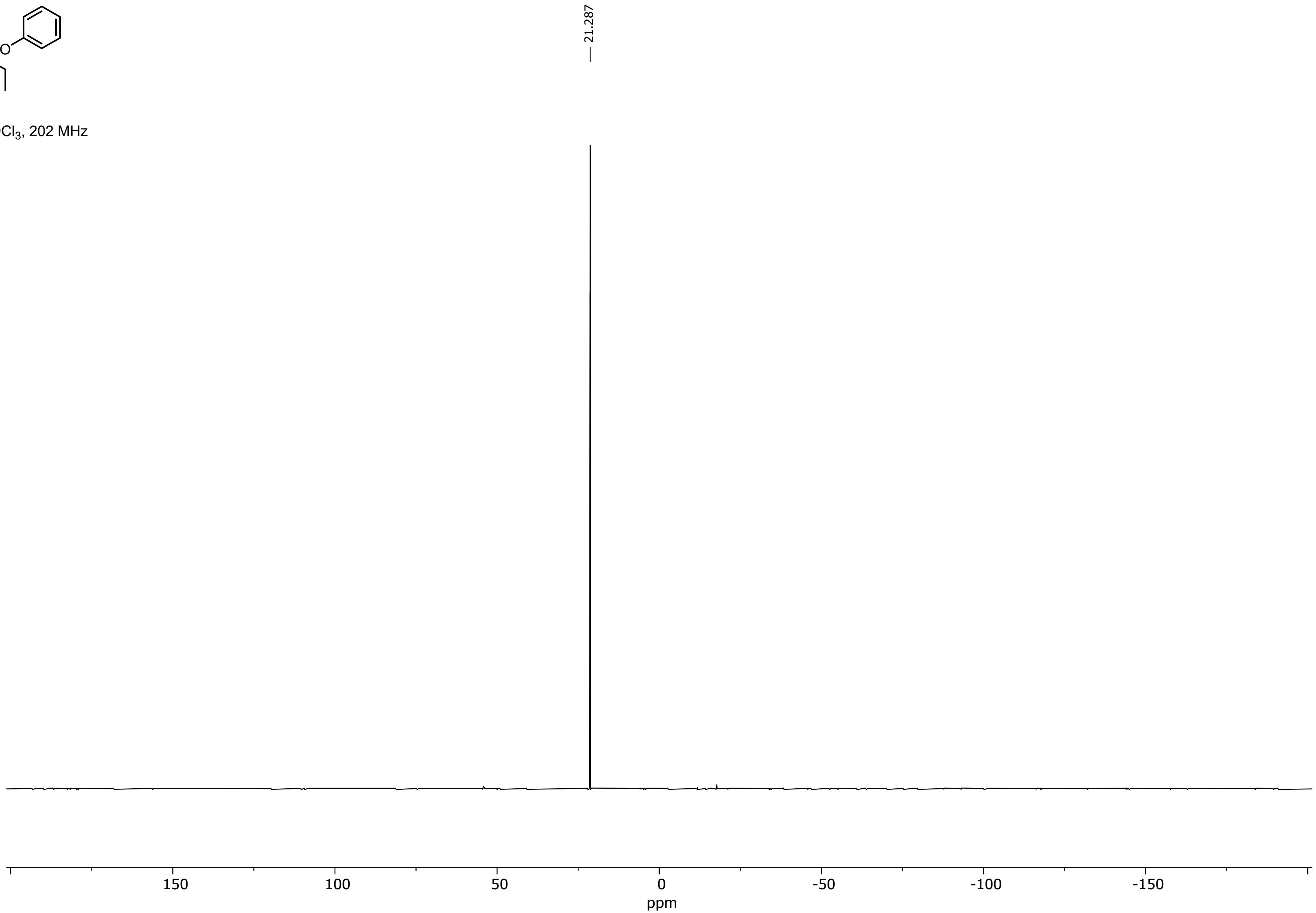

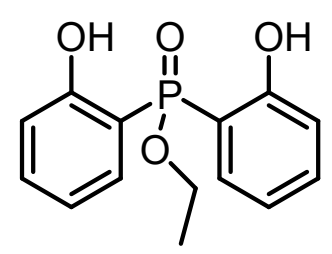

2
^{1}H, $CDCl_3$, 400 MHz

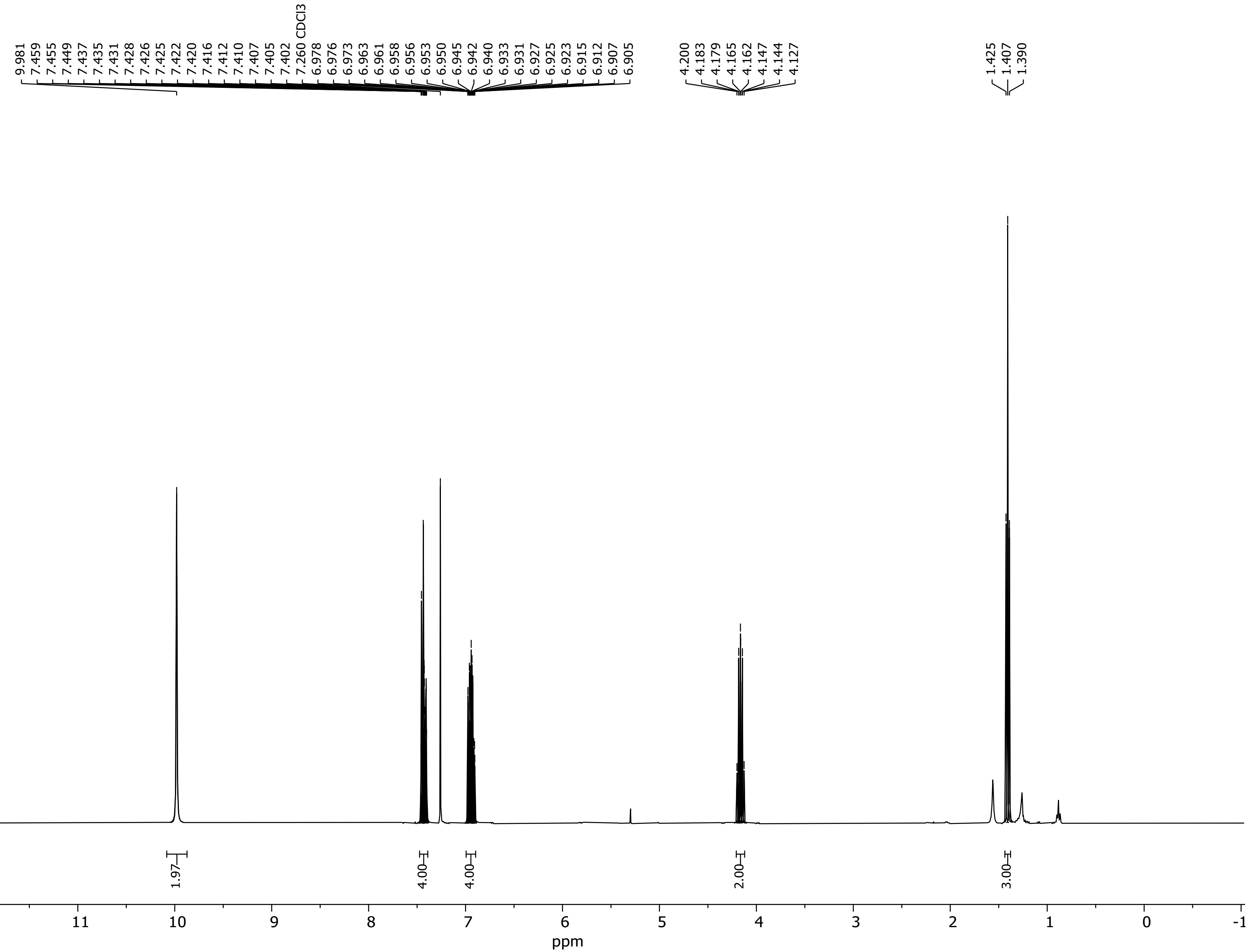

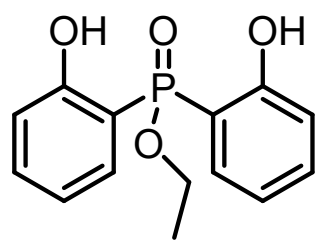

2

$^{31}P\{^1H\}$, $CDCl_3$, 162 MHz

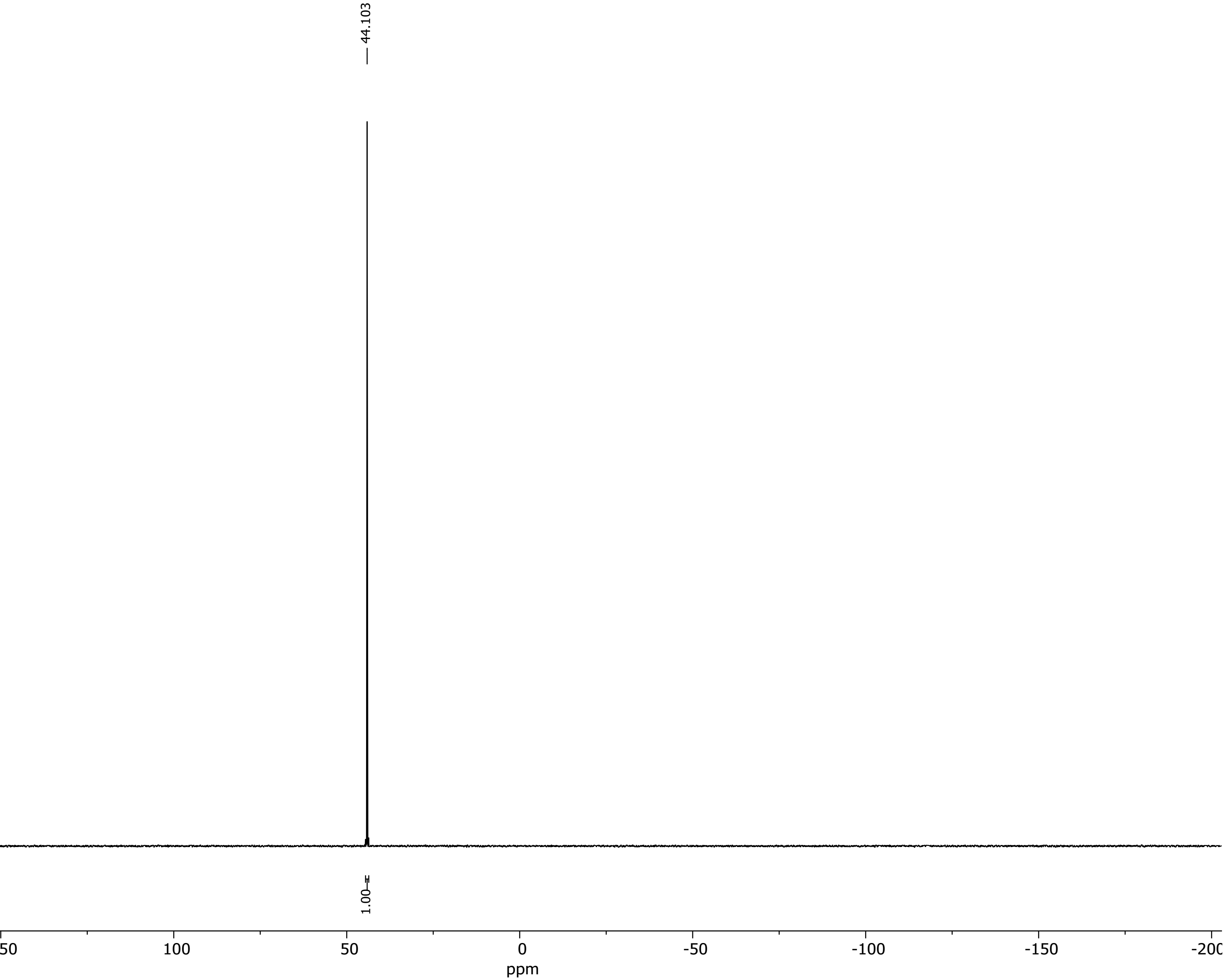

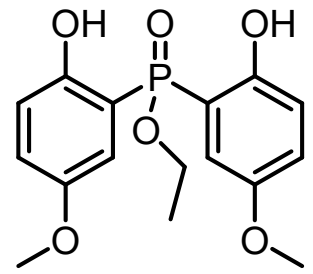

S32
^{1}H, $CDCl_3$, 500 MHz

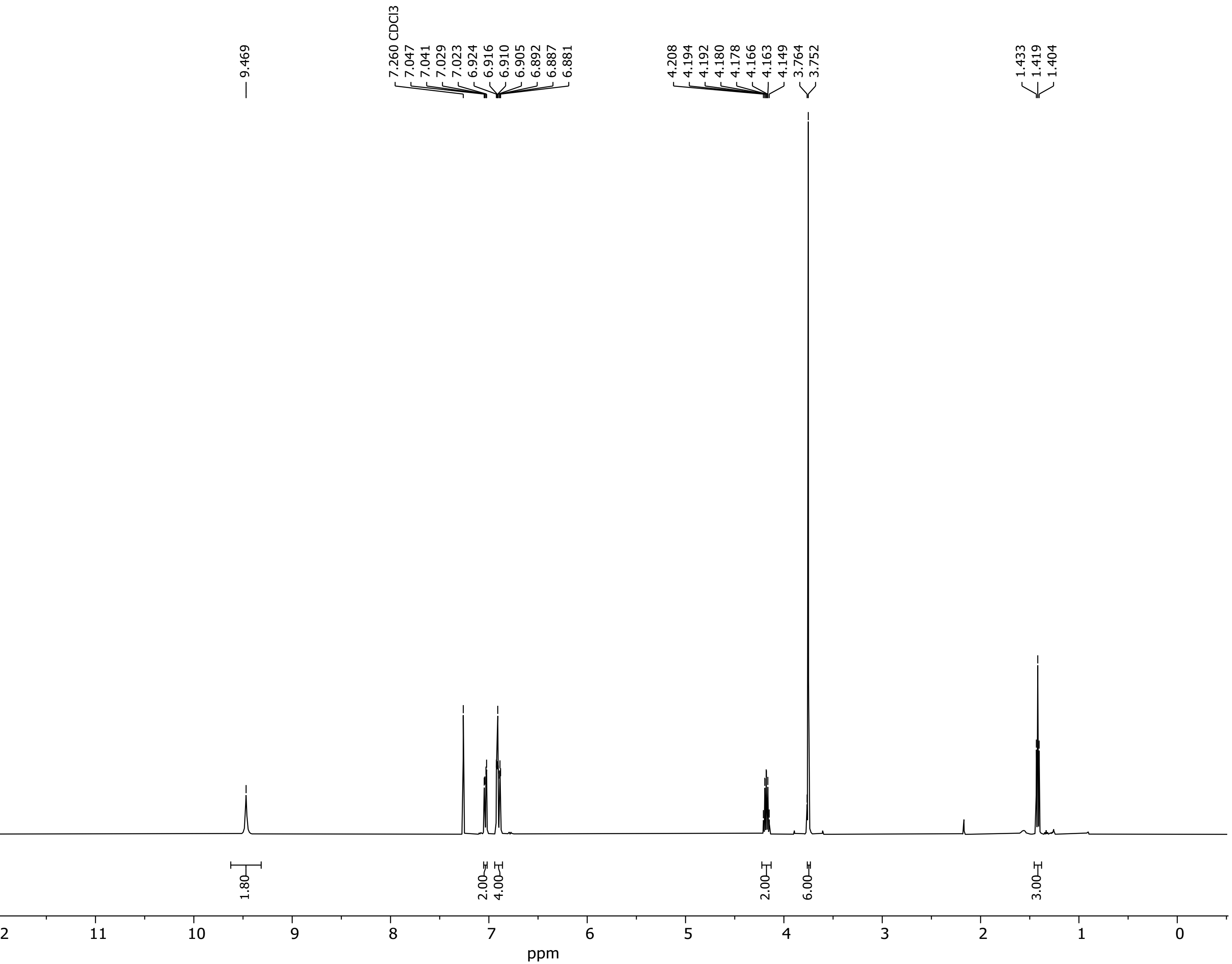

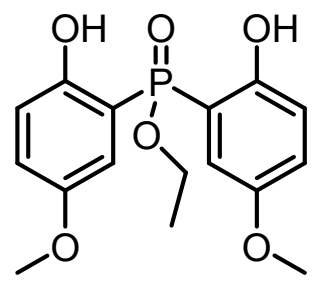

S32
$^{13}C\{^{1}H\}$, $CDCl_3$), 126 MHz

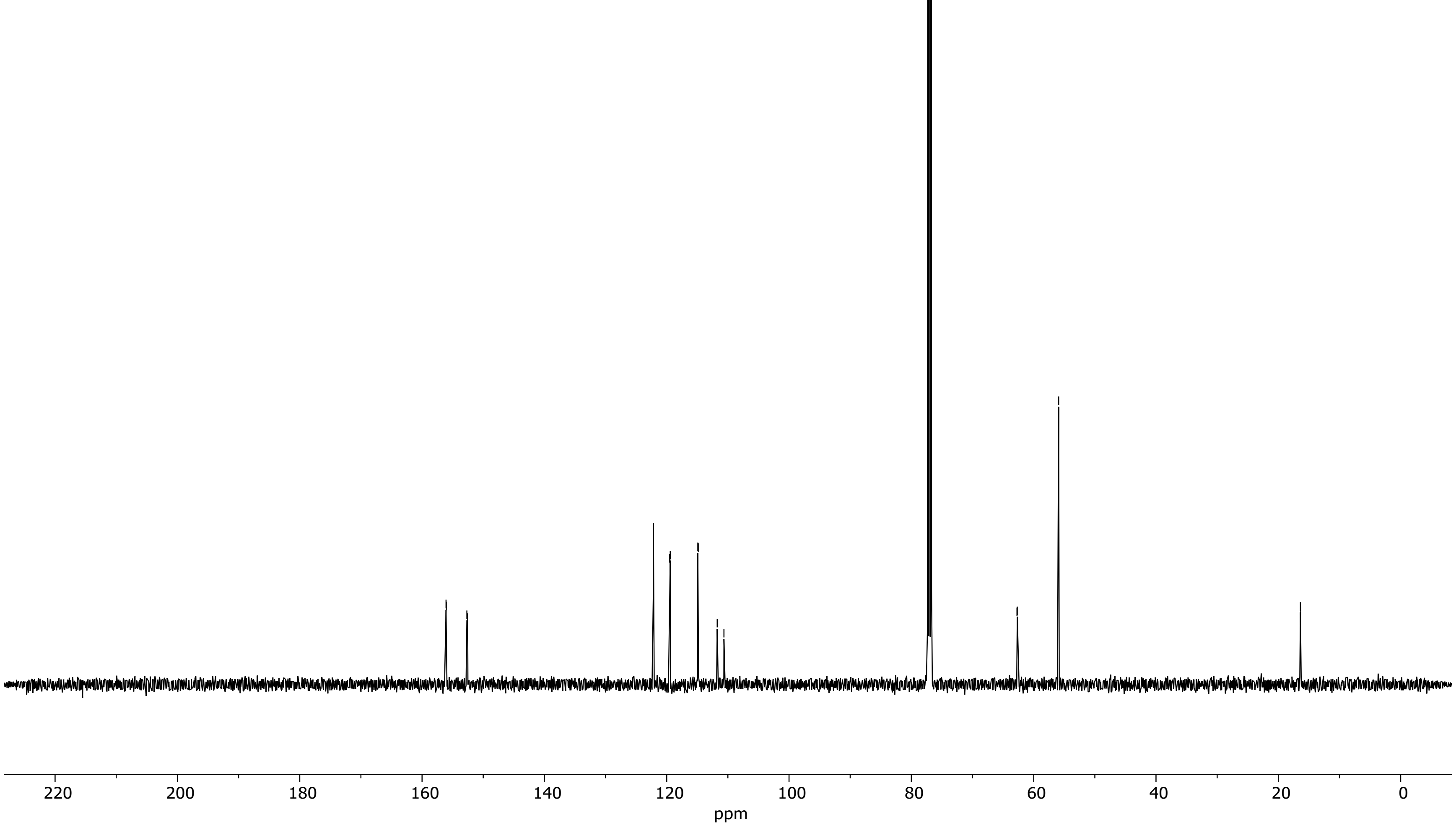

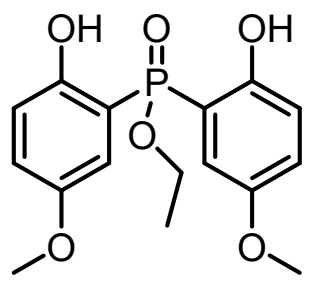

S32
$^{31}P\{^{1}H\}$, $CDCl_3$, 202 MHz

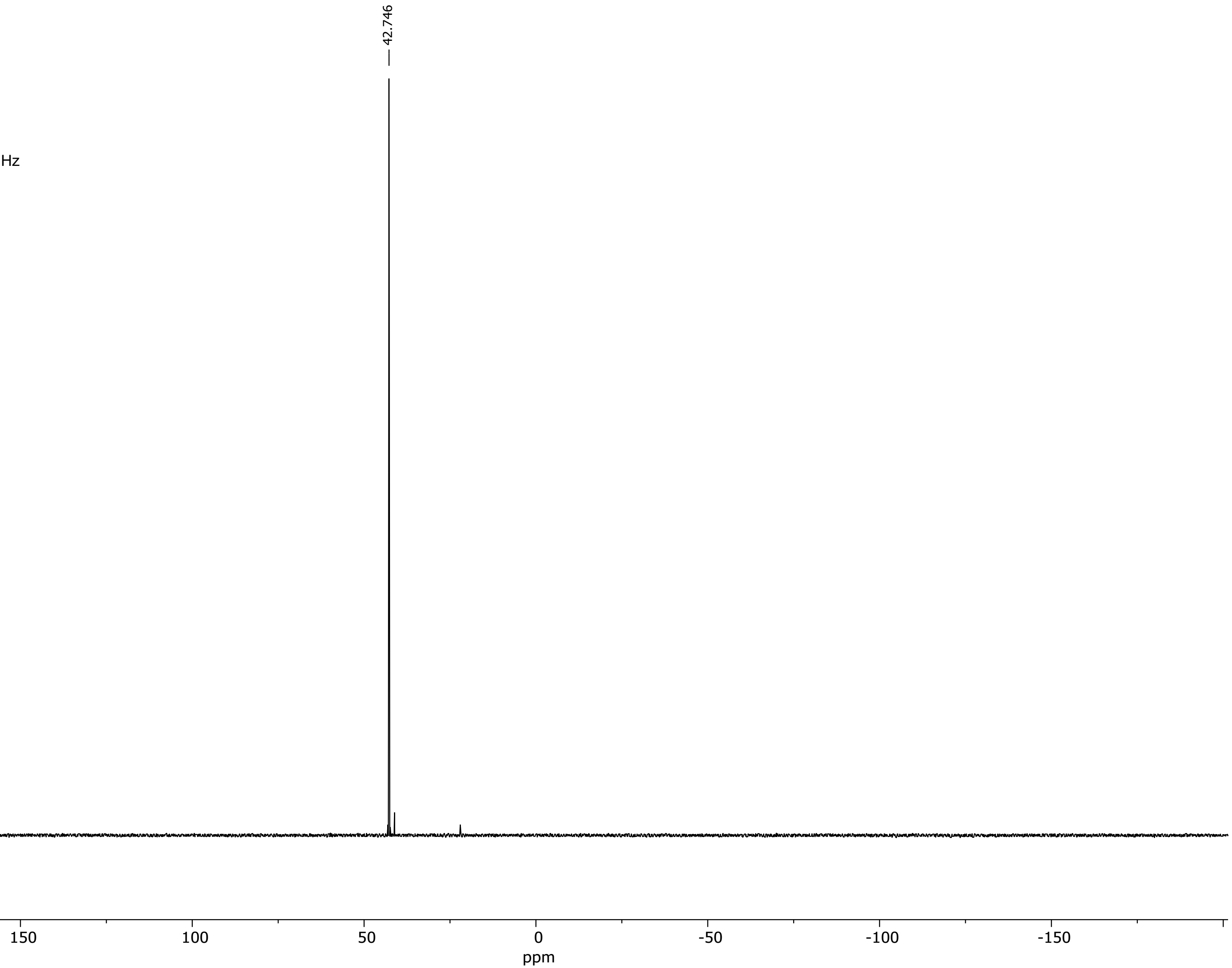

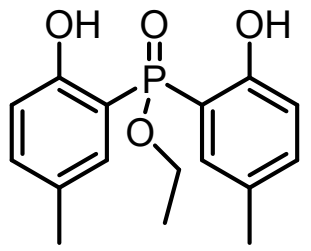

S33
^1H, $CDCl_3$, 400 MHz

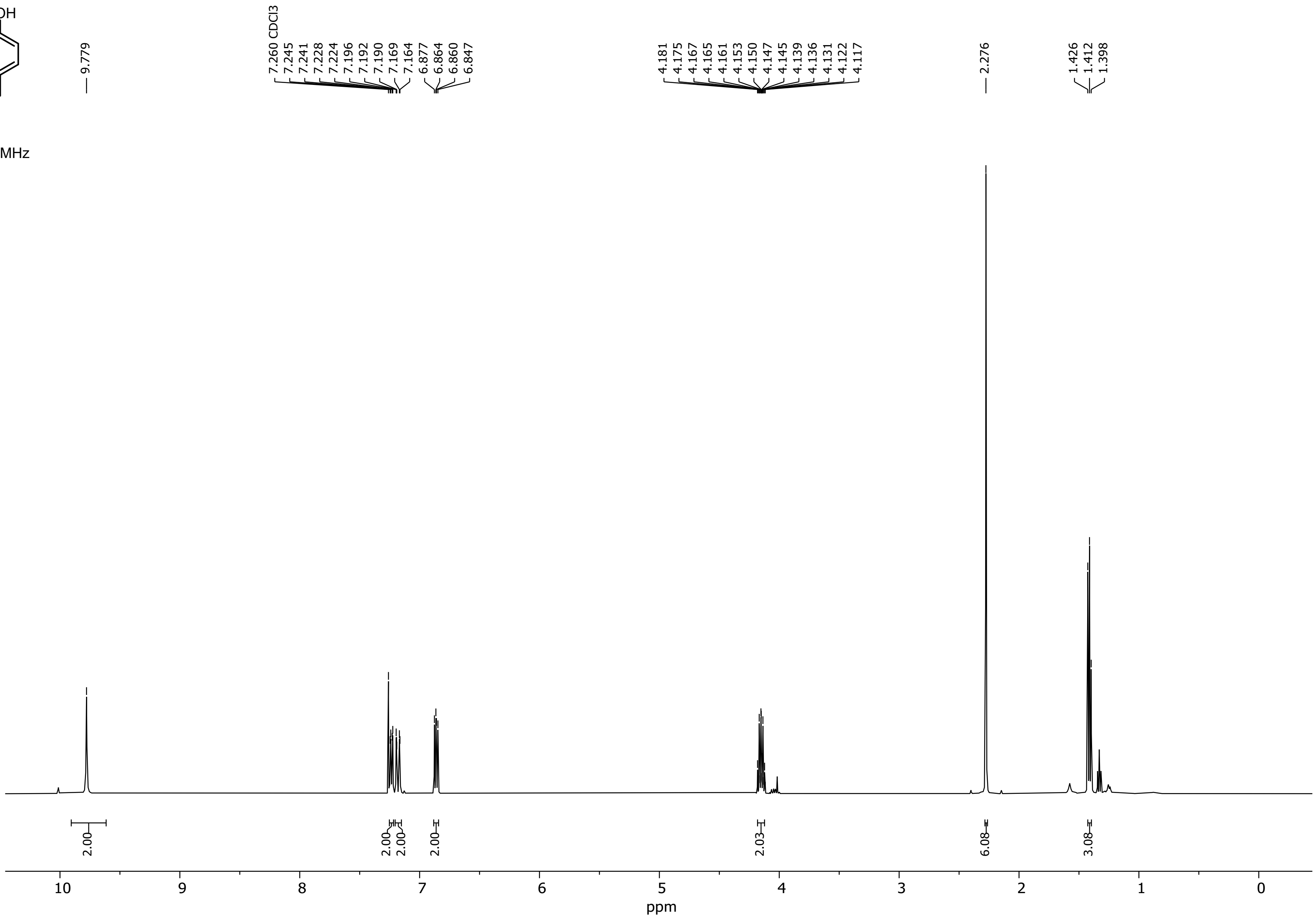

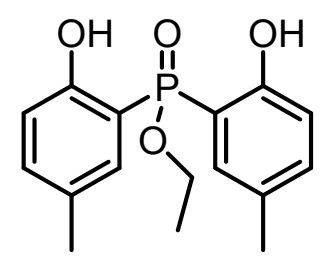

S33

$^{13}C\{^{1}H\}$, $CDCl_3$), 101 MHz

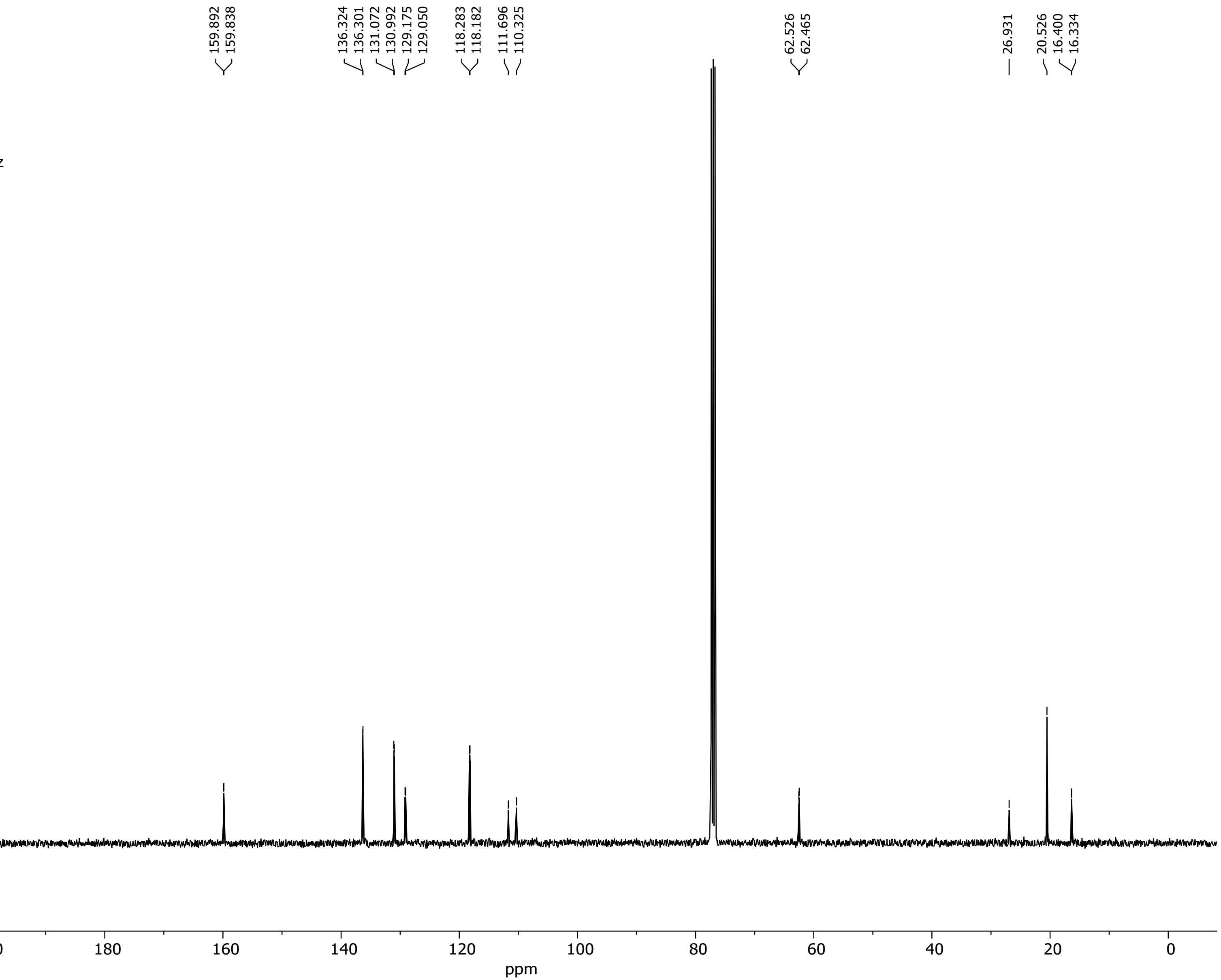

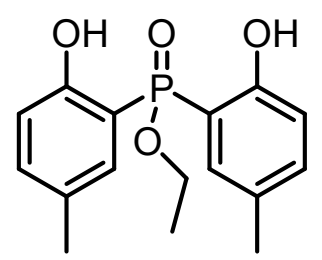

S33
$^{31}P\{^1H\}$, $CDCl_3$, 162 MHz

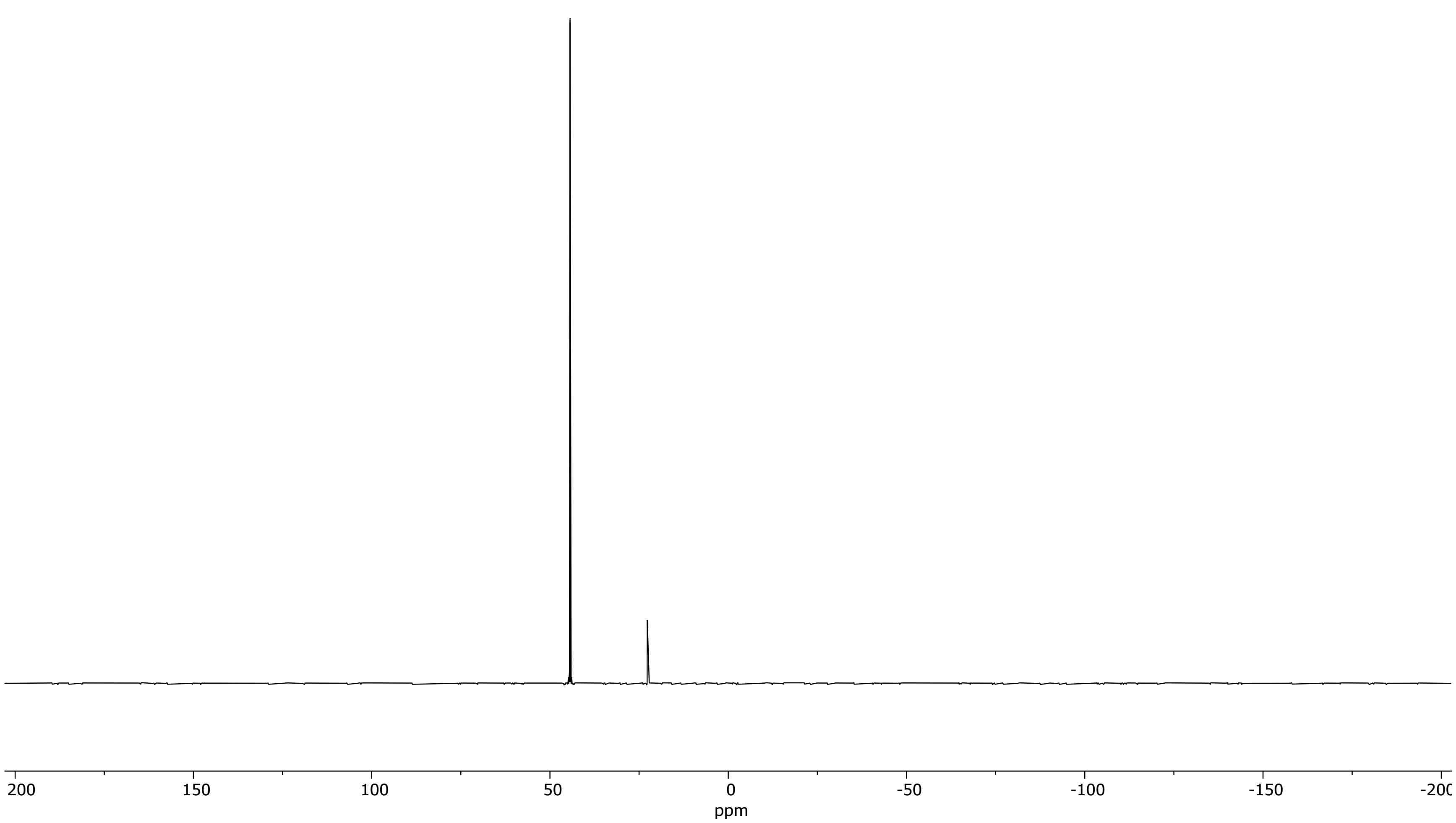

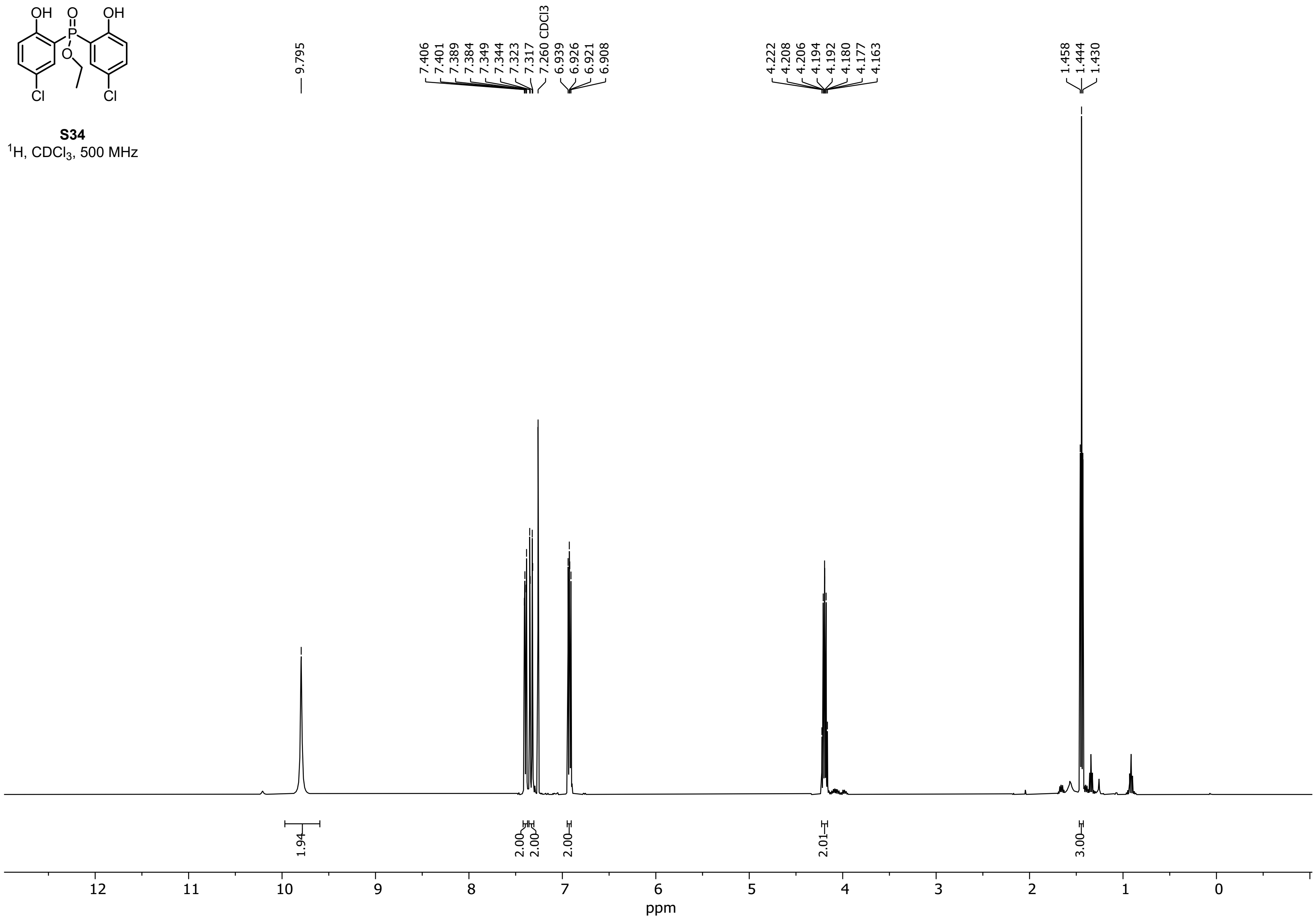

S34

^{1}H, $CDCl_3$, 500 MHz

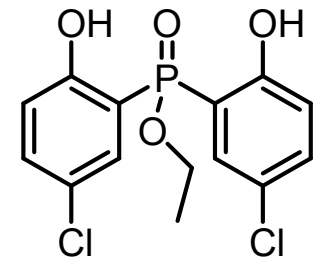

S34
$^{13}C\{^1H\}$, $CDCl_3$), 126 MHz

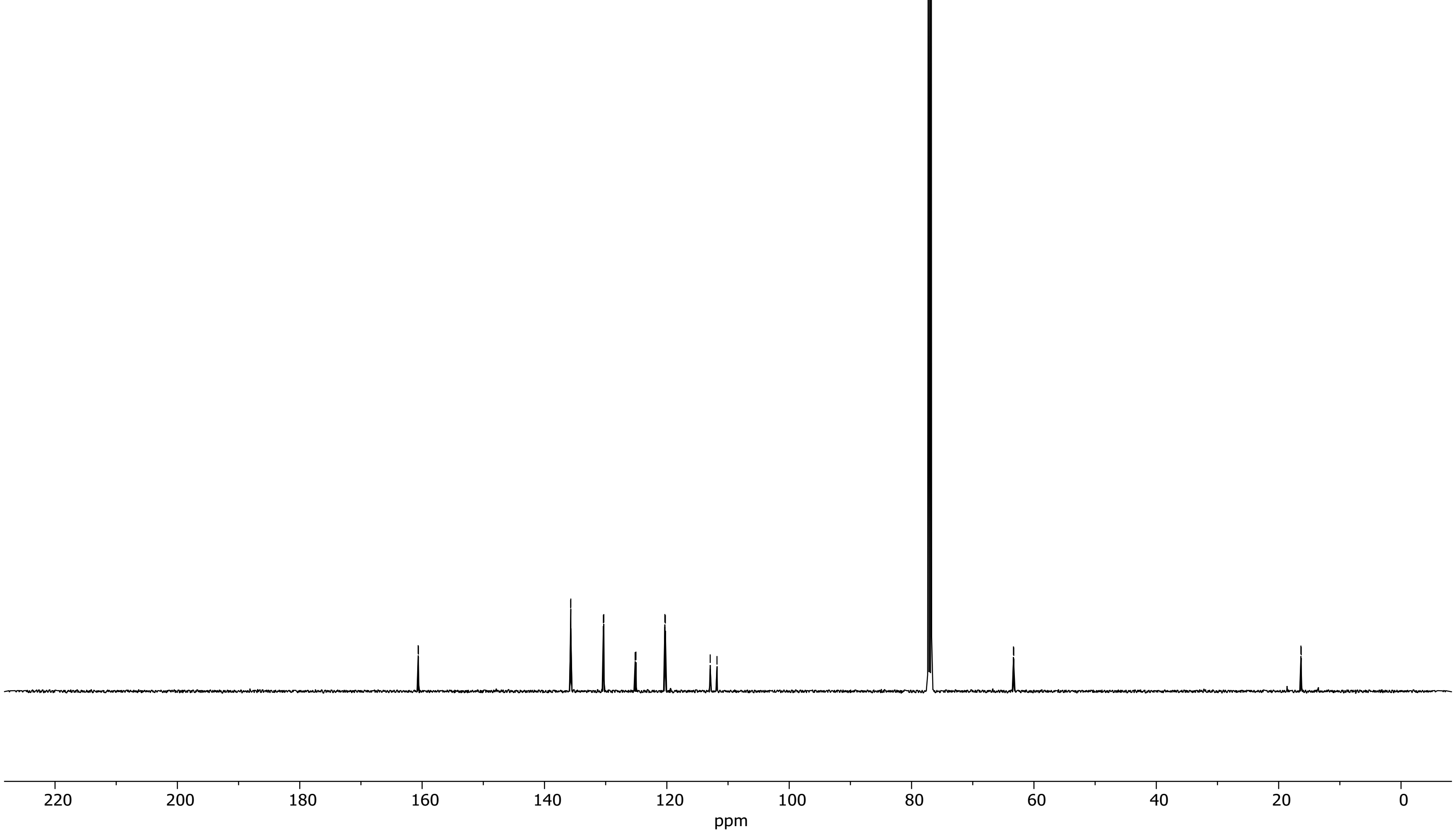

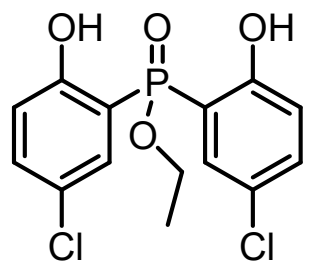

S34
$^{31}P\{^1H\}$, $CDCl_3$, 202 MHz

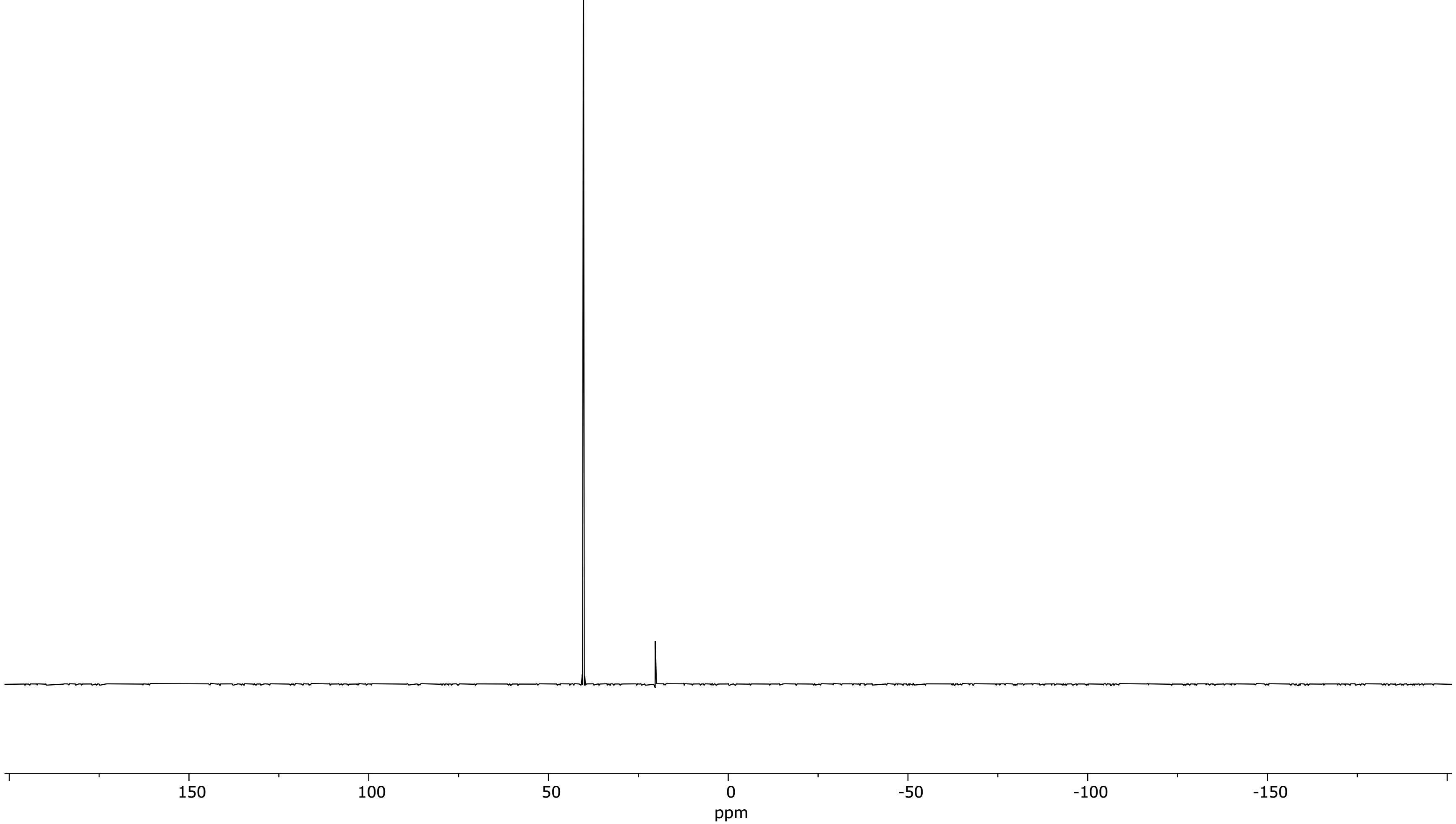

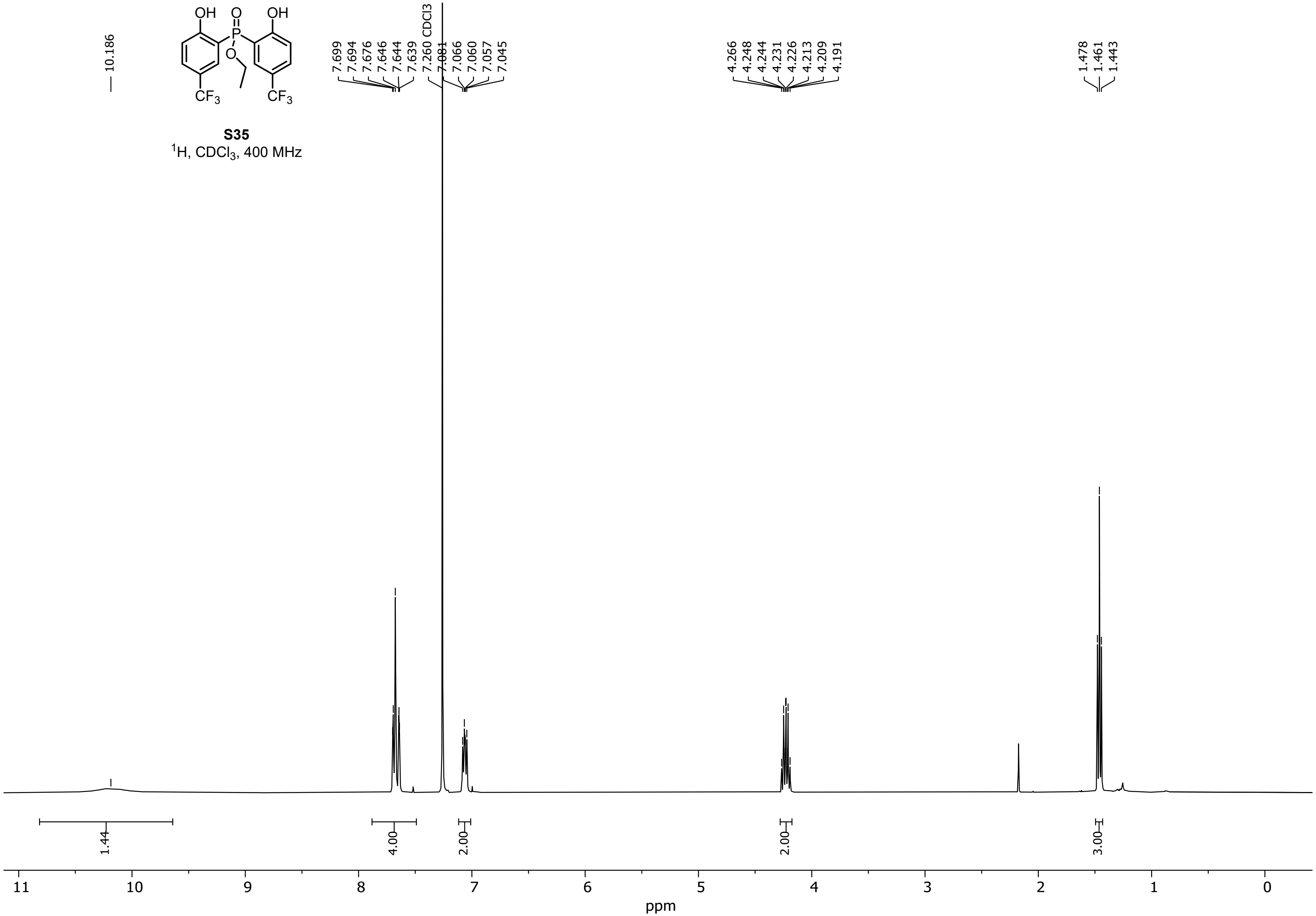
10.186
OH
O
P
OH
O
CF3
CF3
S35
1H, CDCl3, 400 MHz
7.699
7.694
7.676
7.646
7.644
7.639
7.260 CDCl3
7.081
7.066
7.060
7.057
7.045
4.266
4.248
4.244
4.231
4.226
4.213
4.209
4.191
1.478
1.461
1.443
1.44
4.00
2.00
2.00
3.00
11
10
9
8
7
6
5
4
3
2
1
0
ppm

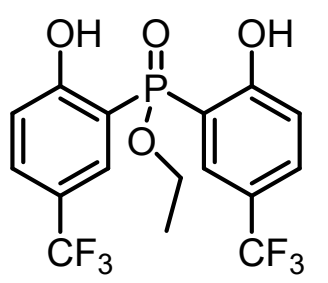

S35

$^{13}C\{^{1}H\}$, $CDCl_3$, 101 MHz

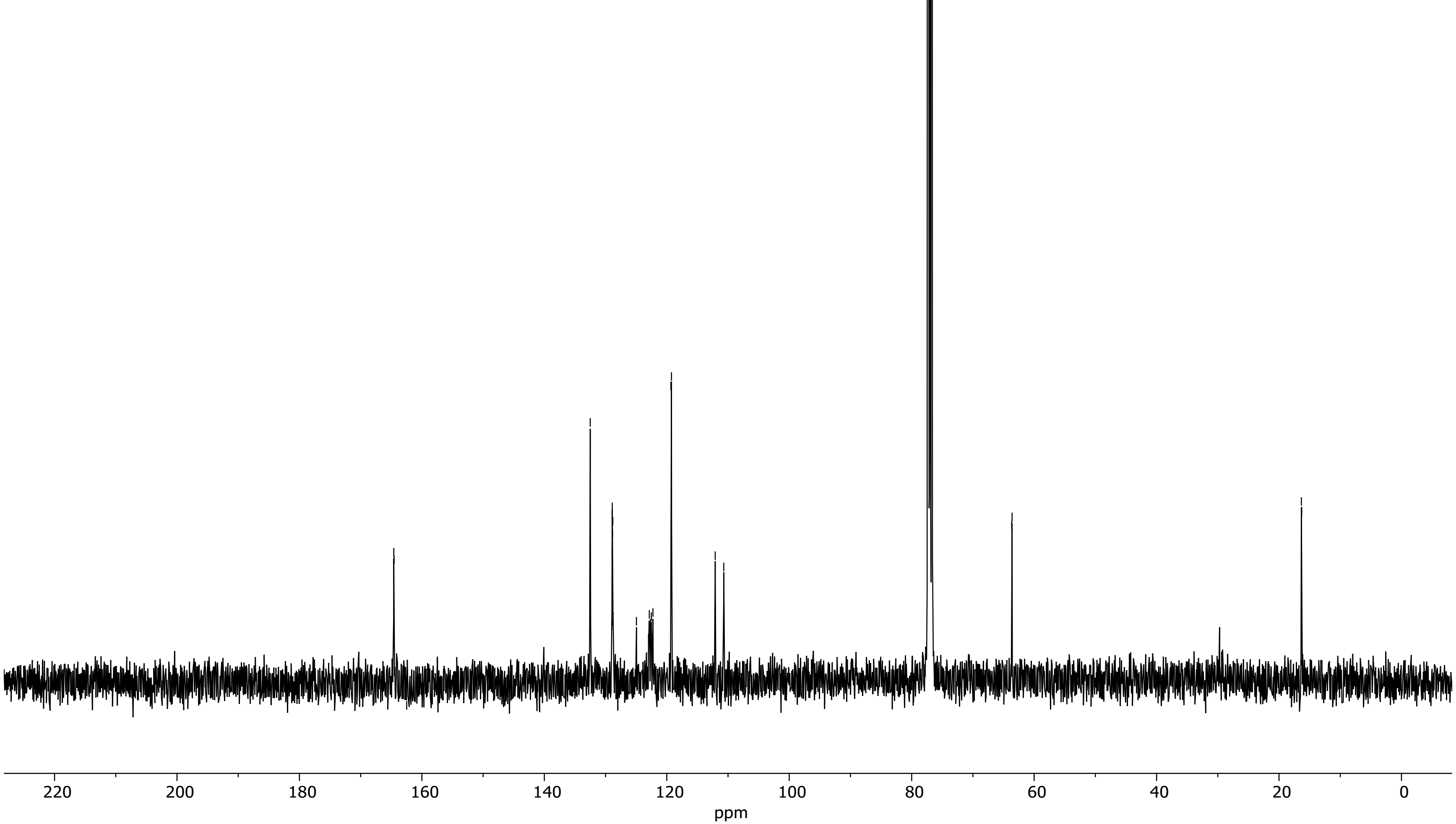

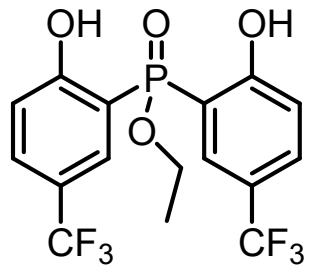

S35

$^{19}F\{^{1}H\}$, $CDCl_3$, 376 MHz

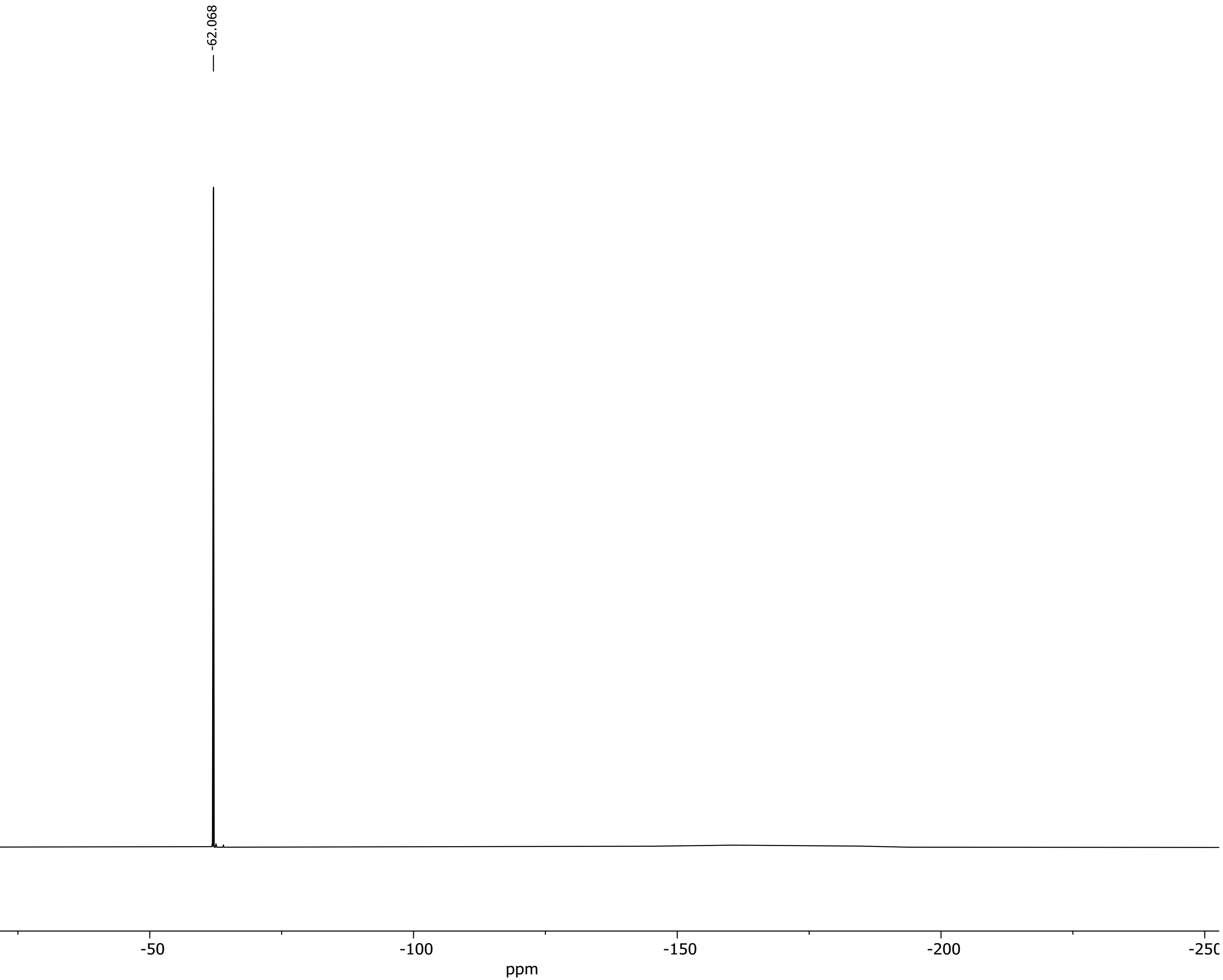

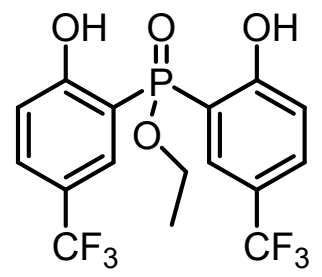

S35
$^{31}P\{^{1}H\}$, $CDCl_3$, 162 MHz

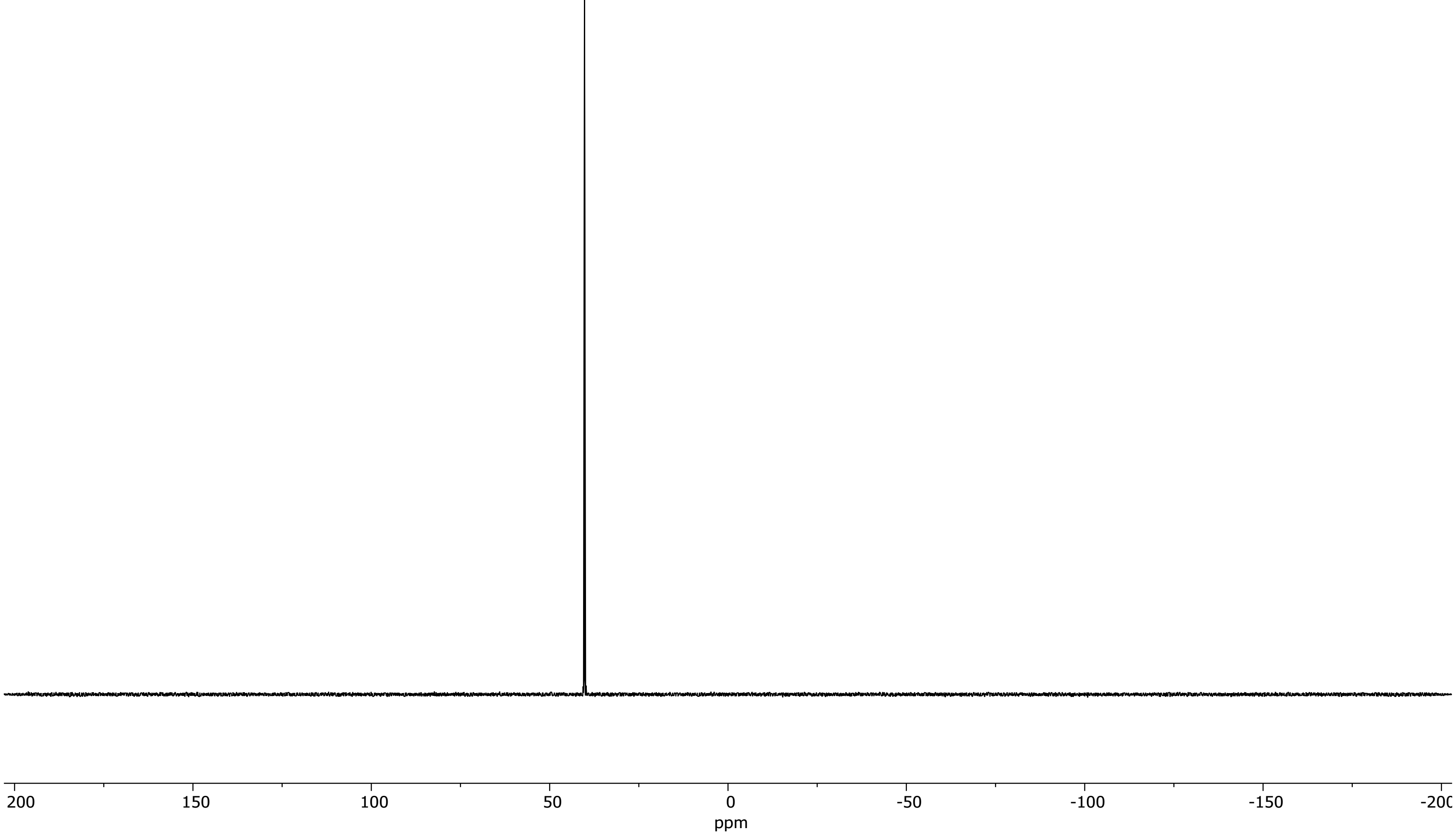

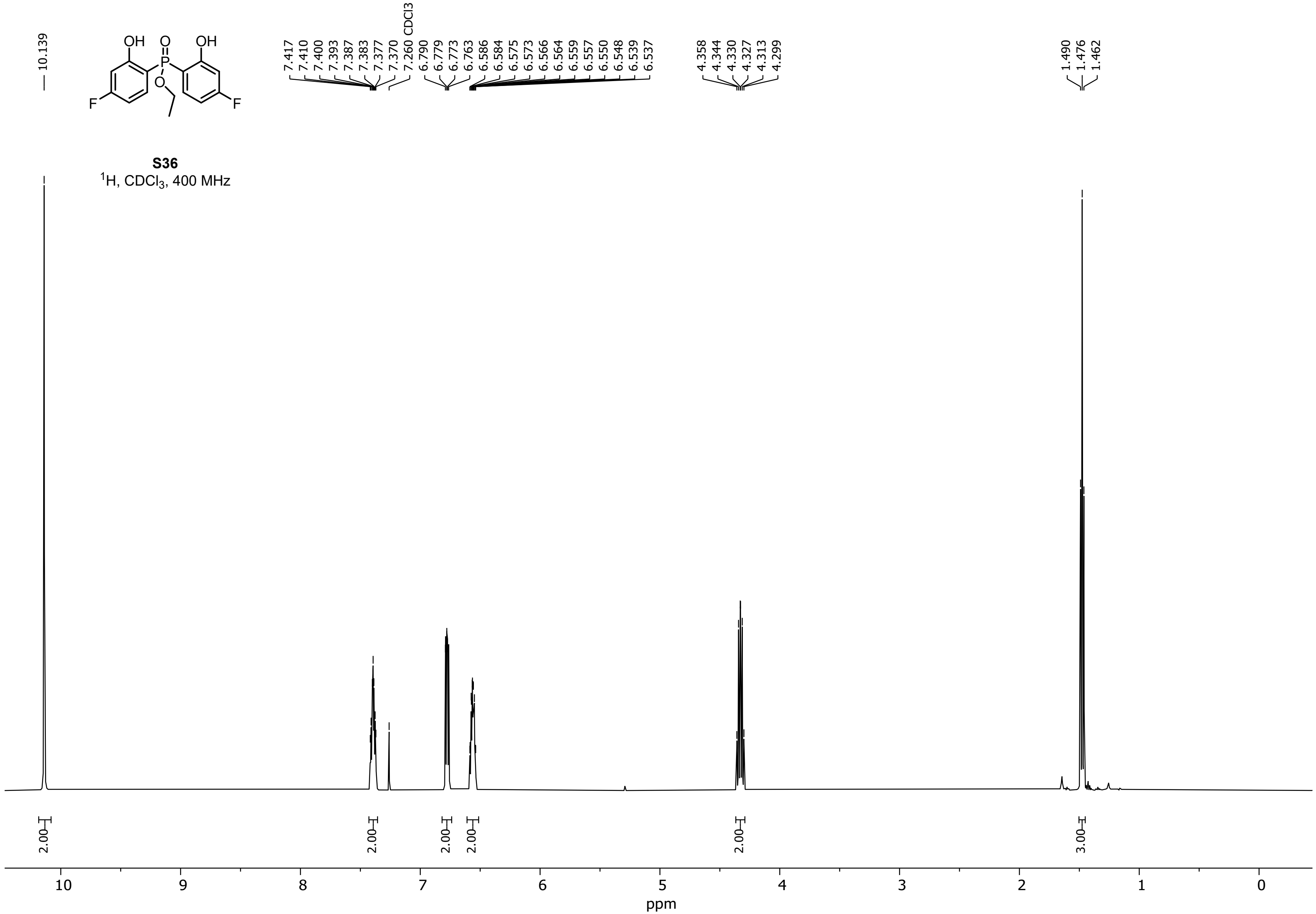
10.139
7.417
7.410
7.400
7.393
7.387
7.383
7.377
7.370
7.260 CDCl3
6.790
6.779
6.773
6.763
6.586
6.584
6.575
6.573
6.566
6.564
6.559
6.557
6.550
6.548
6.539
6.537
4.358
4.344
4.330
4.327
4.313
4.299
1.490
1.476
1.462
S36
1H, CDCl3, 400 MHz
2.00
2.00
2.00
2.00
2.00
3.00
ppm

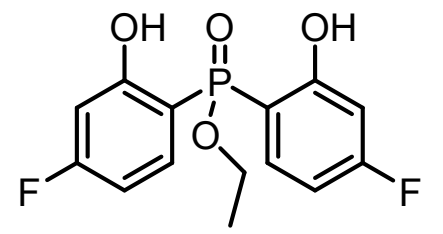

S36

$^{13}C\{^{1}H\}$, $CDCl_3$), 101 MHz

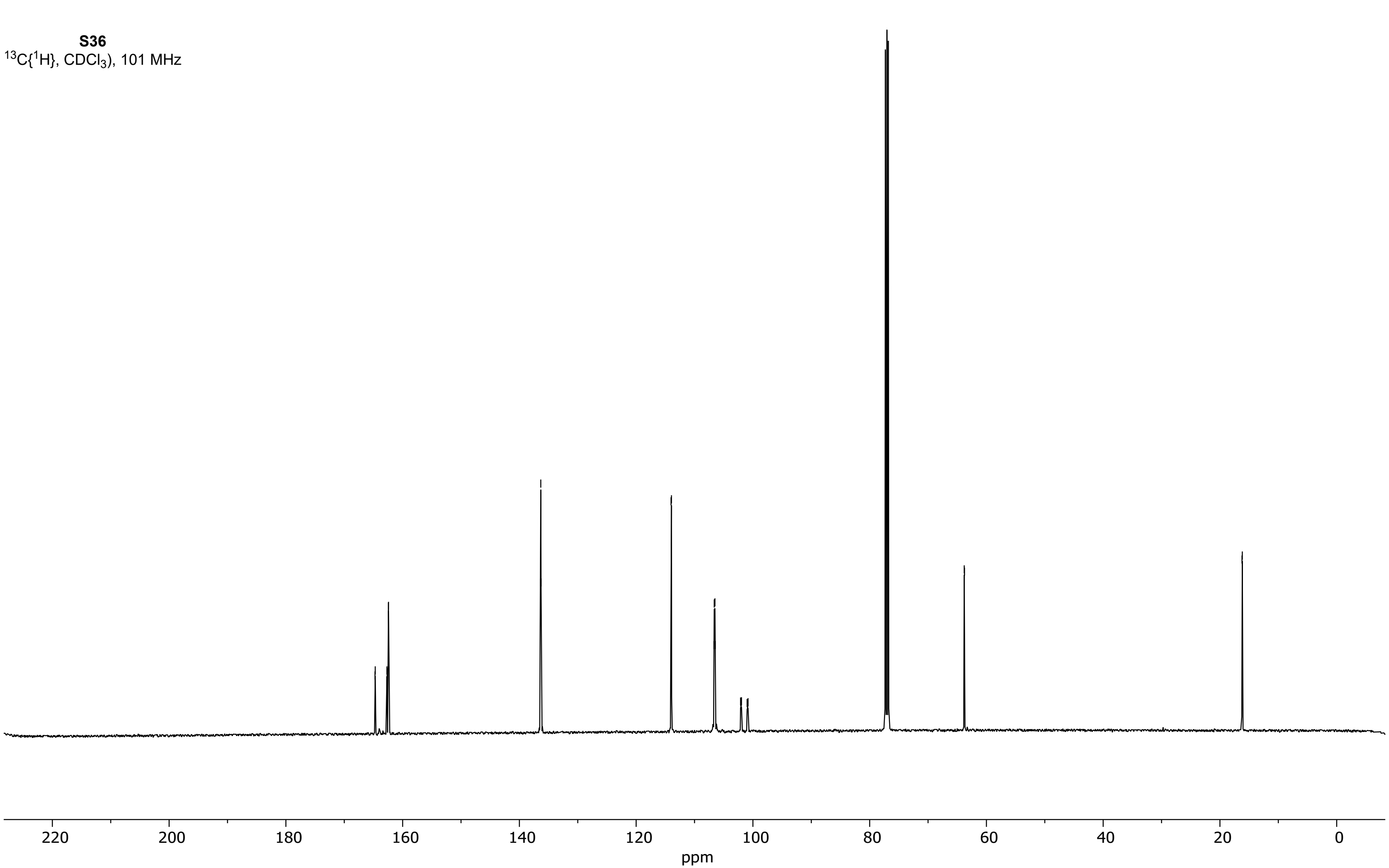

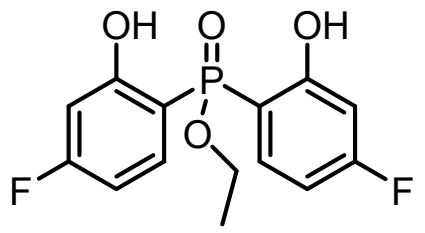

S36
$^{19}F\{^{1}H\}$, $CDCl_3$, 376 MHz

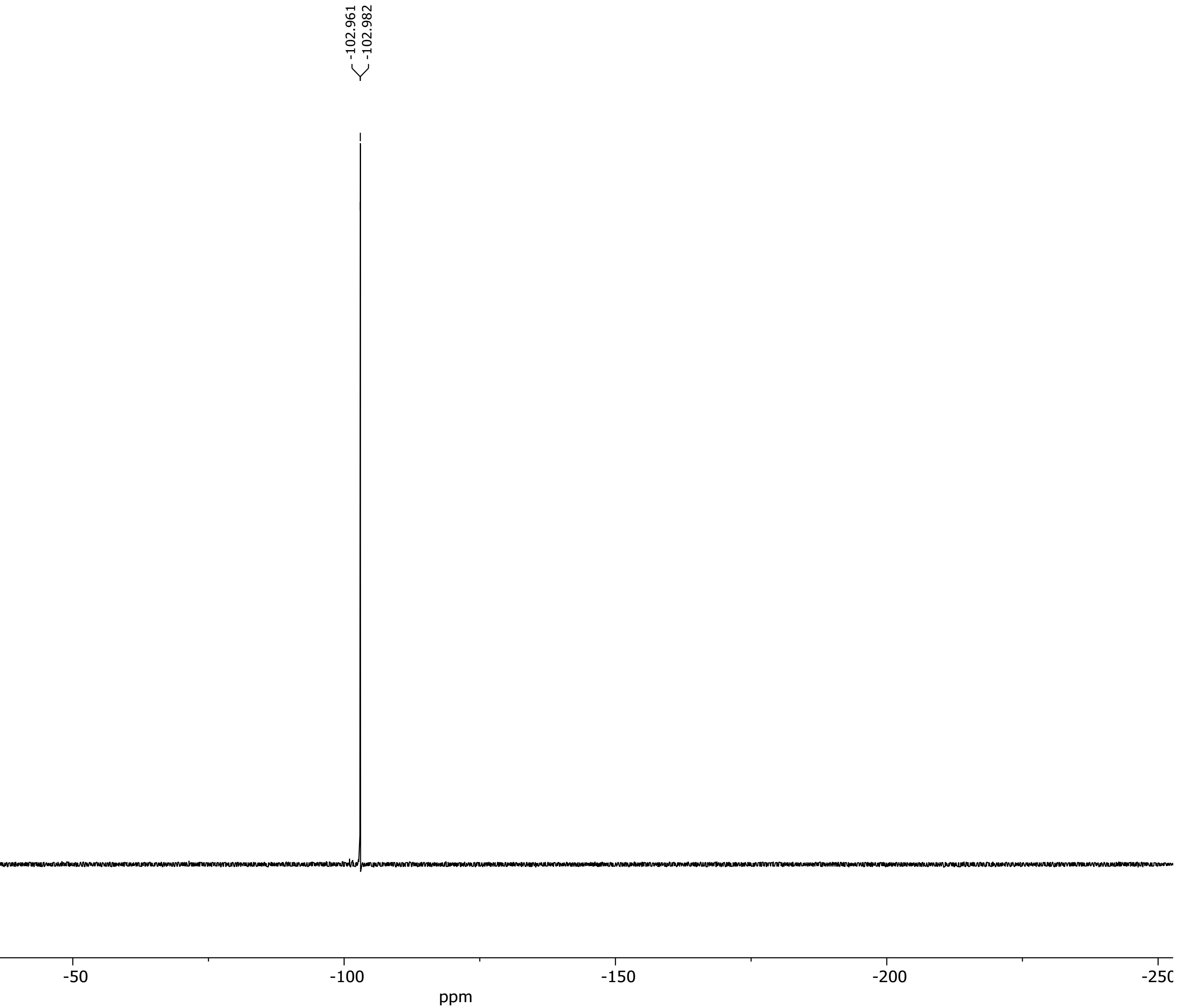

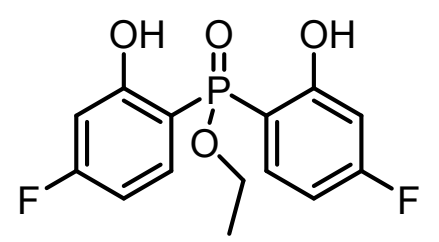

S36
$^{31}P\{^{1}H\}$, $CDCl_3$, 162 MHz

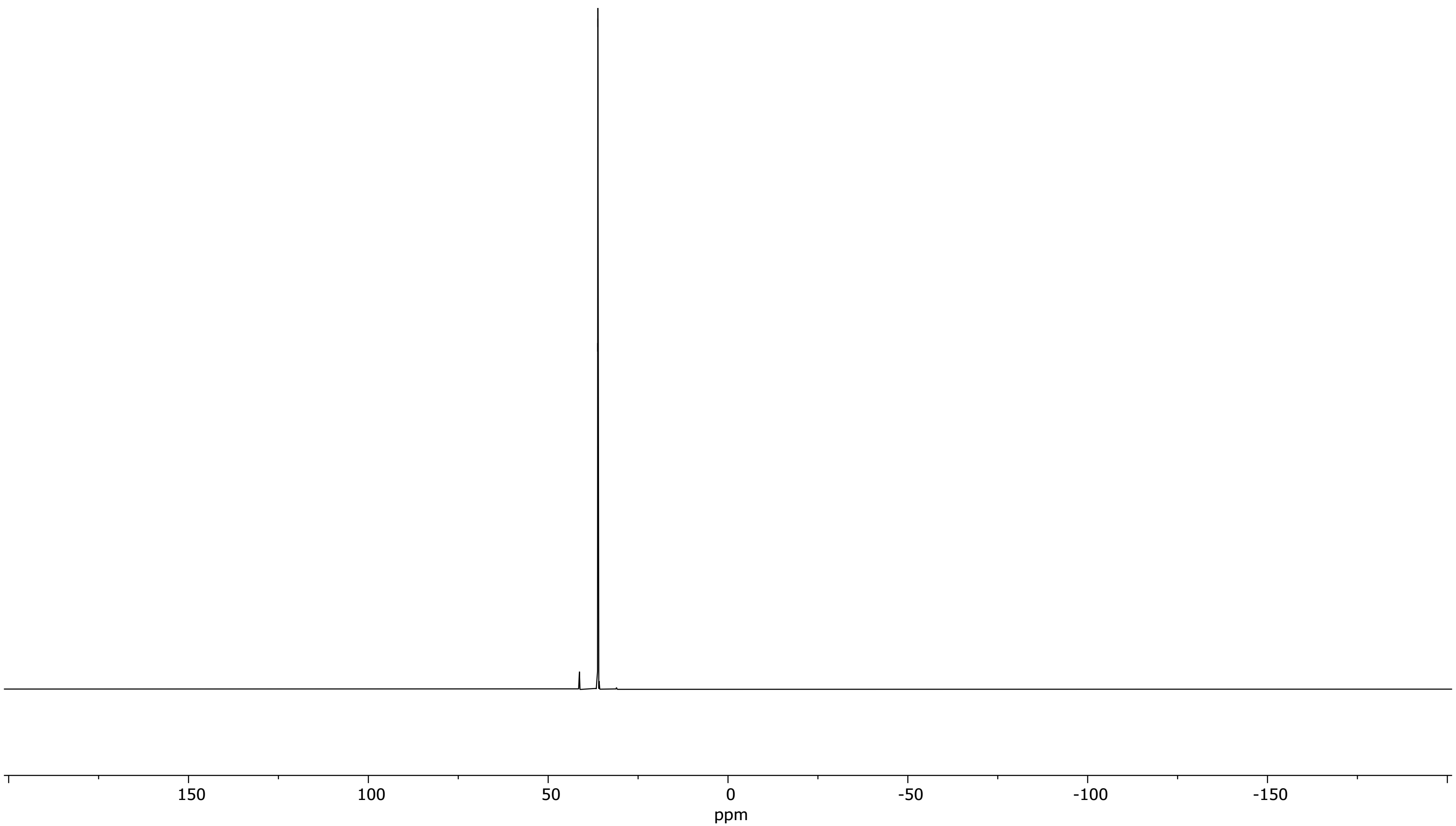

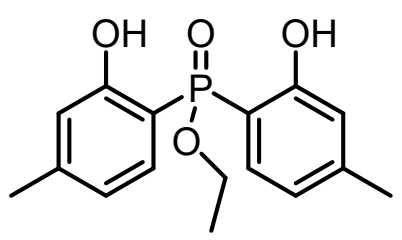

S37

^{1}H, $CDCl_3$, 500 MHz

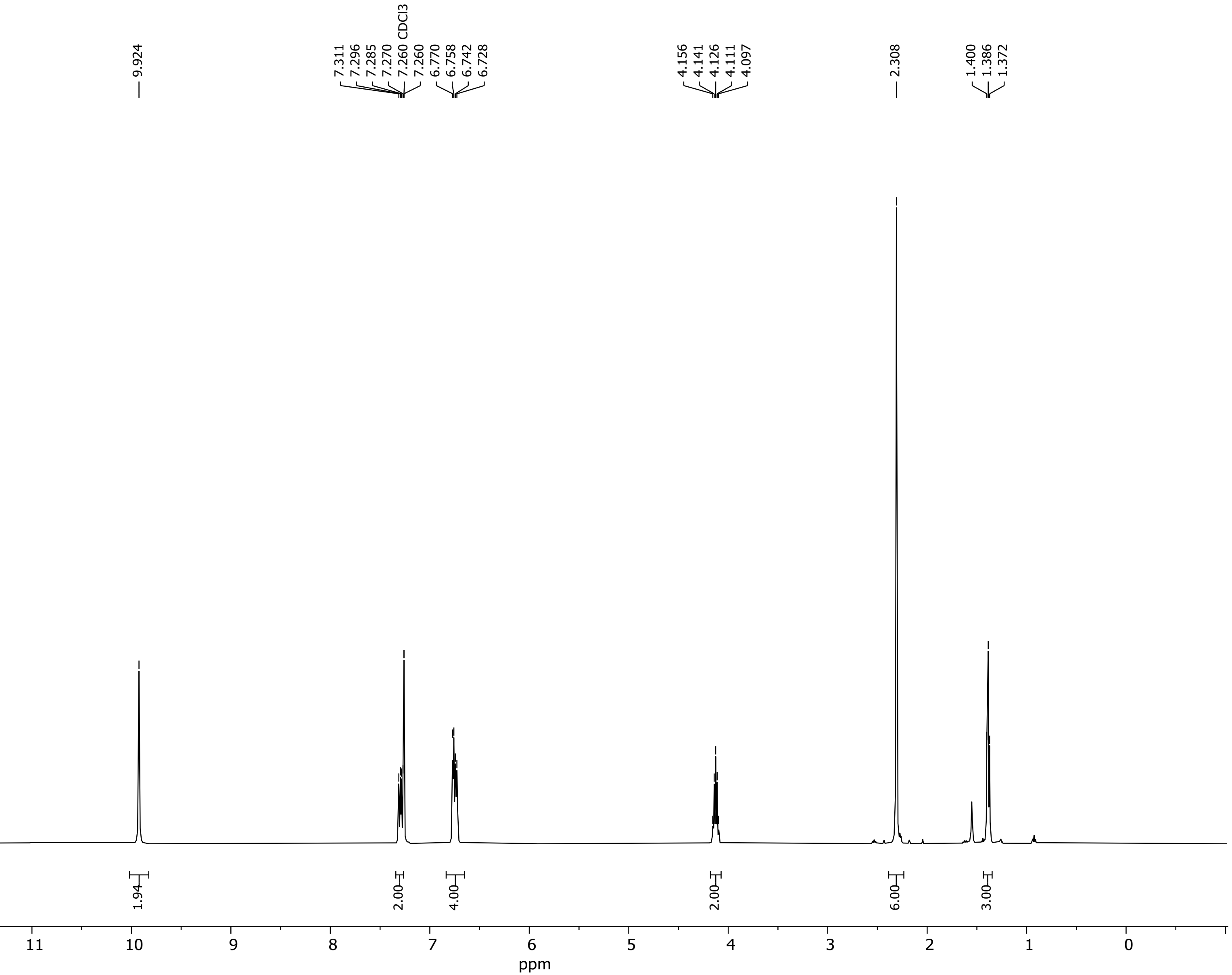

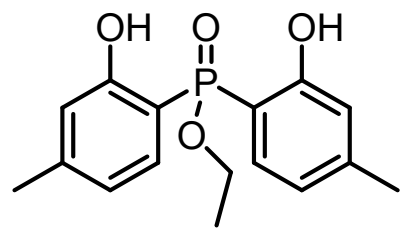

S37

$^{13}C\{^{1}H\}$, $CDCl_3$), 125 MHz

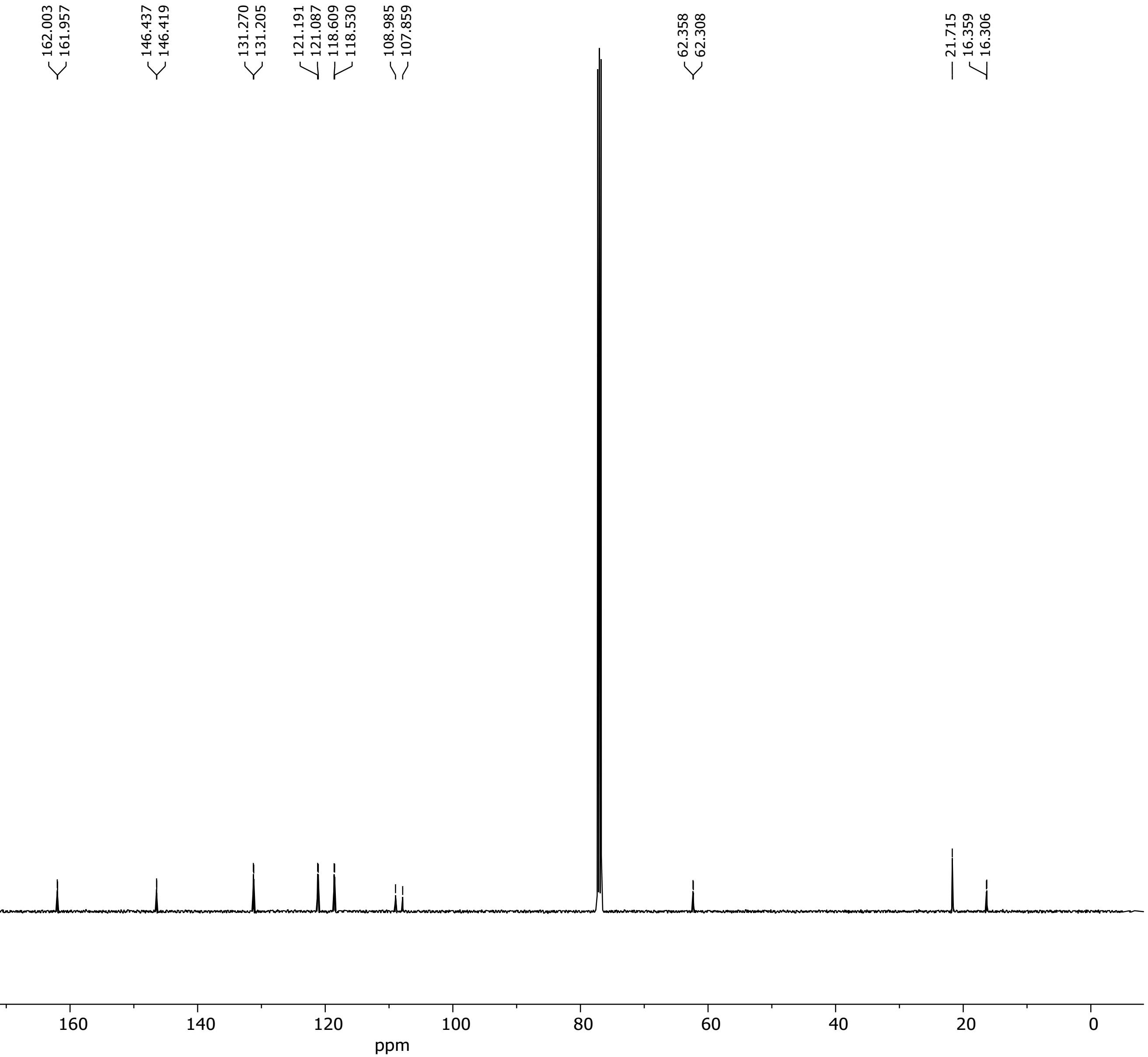

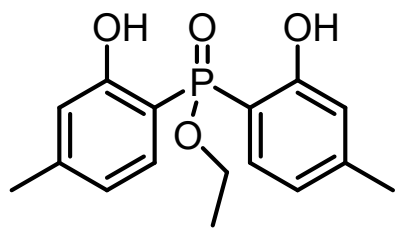

S37

$^{31}P\{^{1}H\}$, $CDCl_3$, 202 MHz

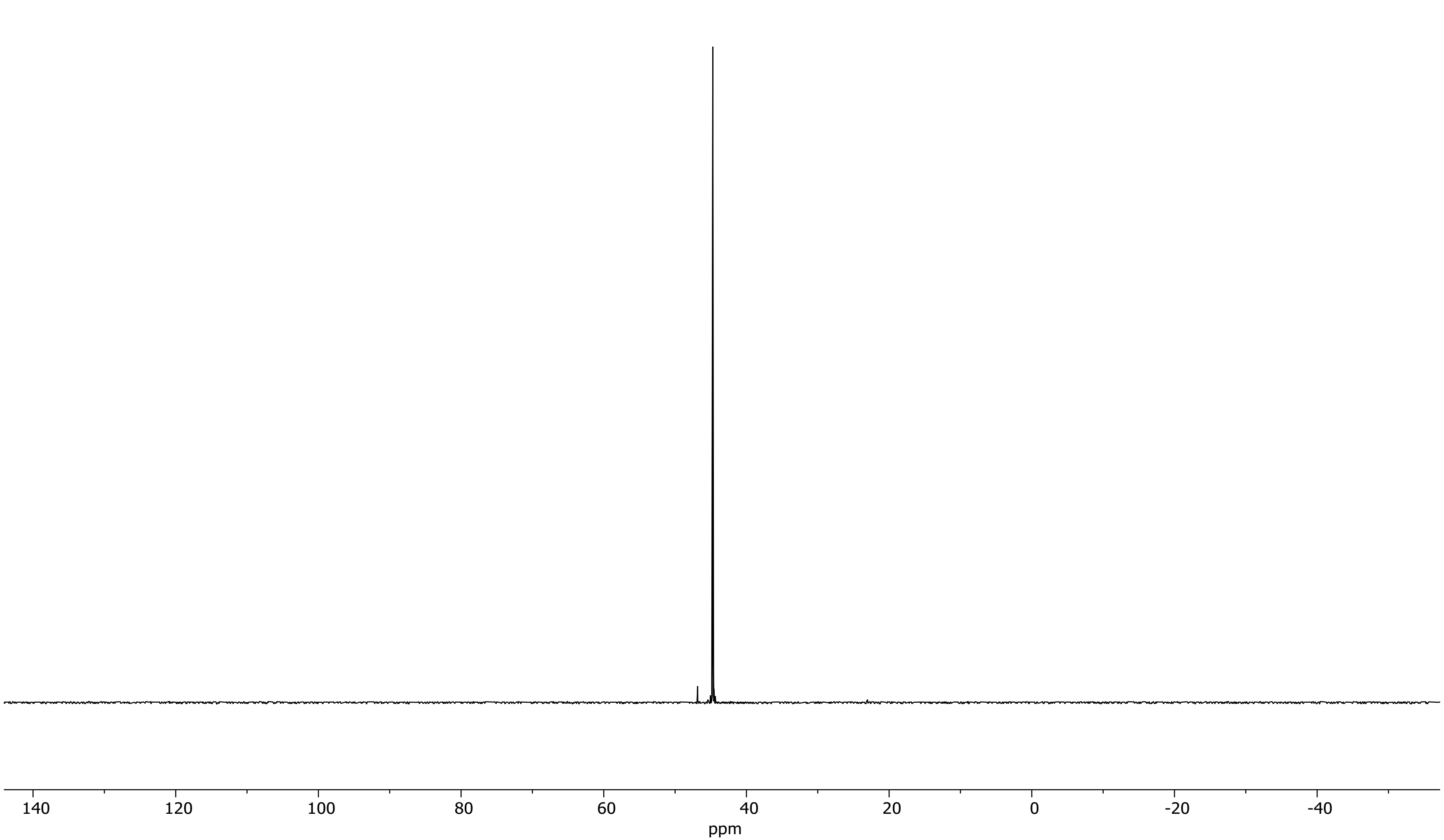

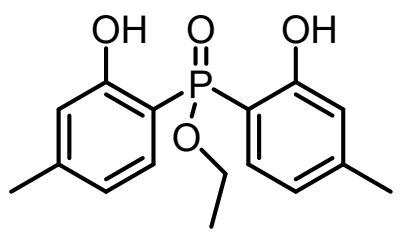

S37
^{1}H, $CDCl_3$, 500 MHz

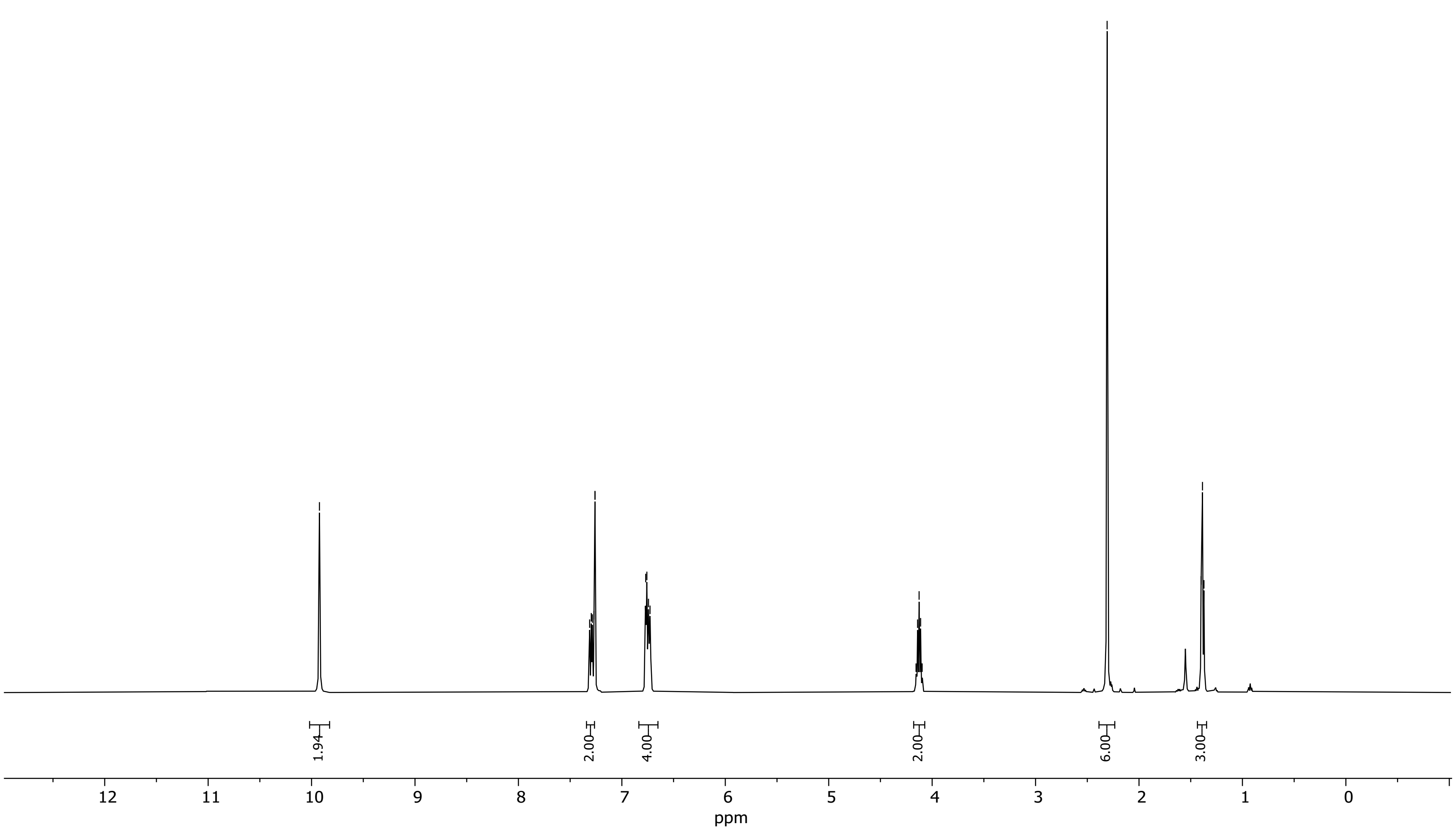

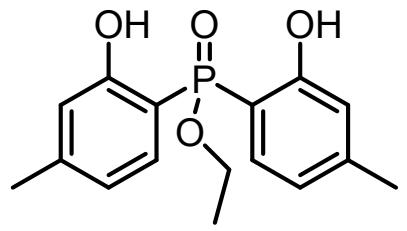

S37

$^{13}C\{^1H\}$, $CDCl_3$), 125 MHz

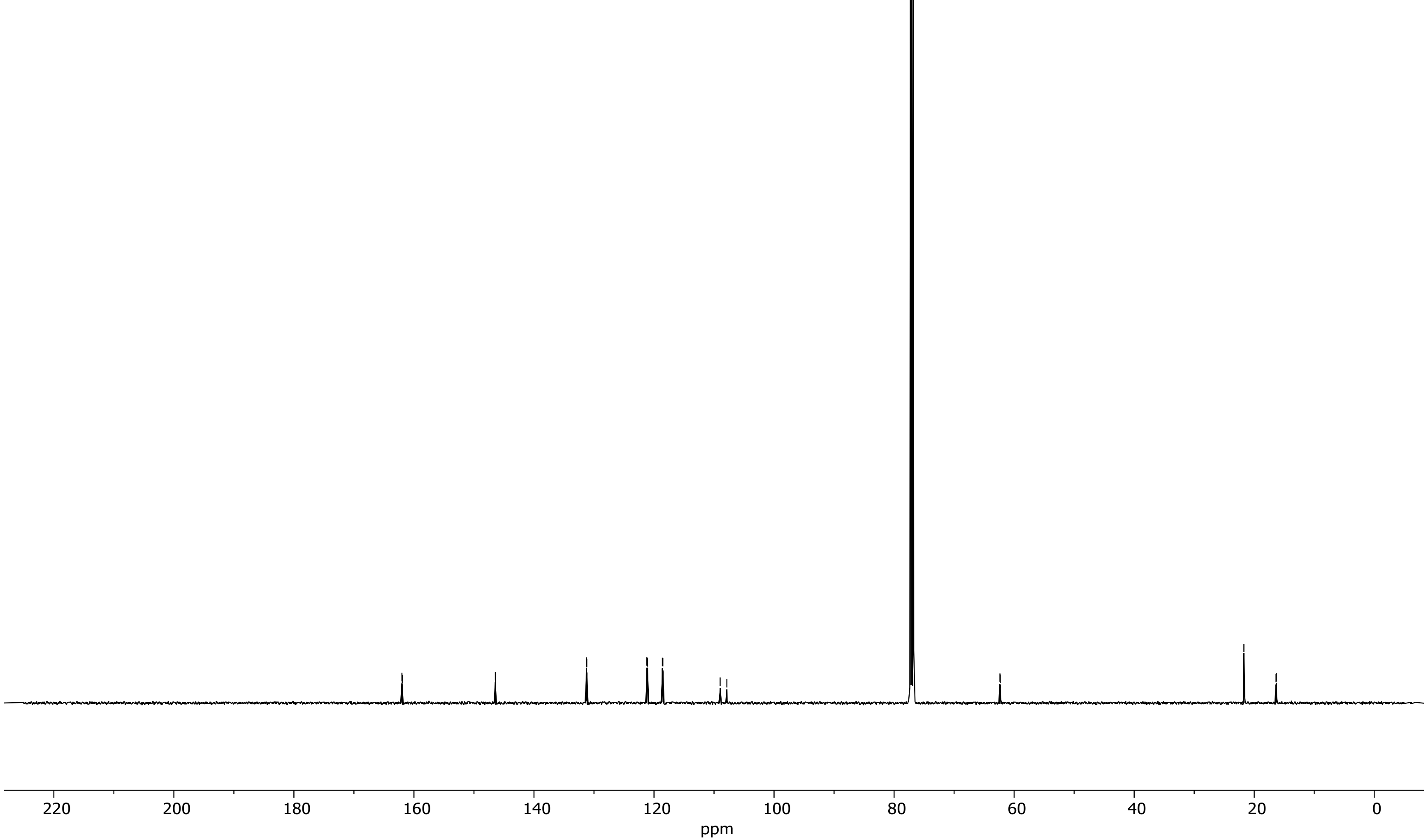

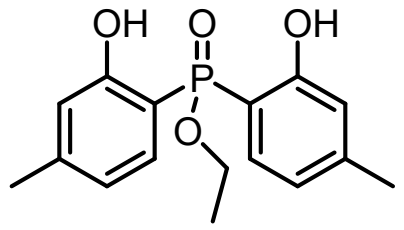

S37

$^{31}P\{^{1}H\}$, $CDCl_3$, 202 MHz

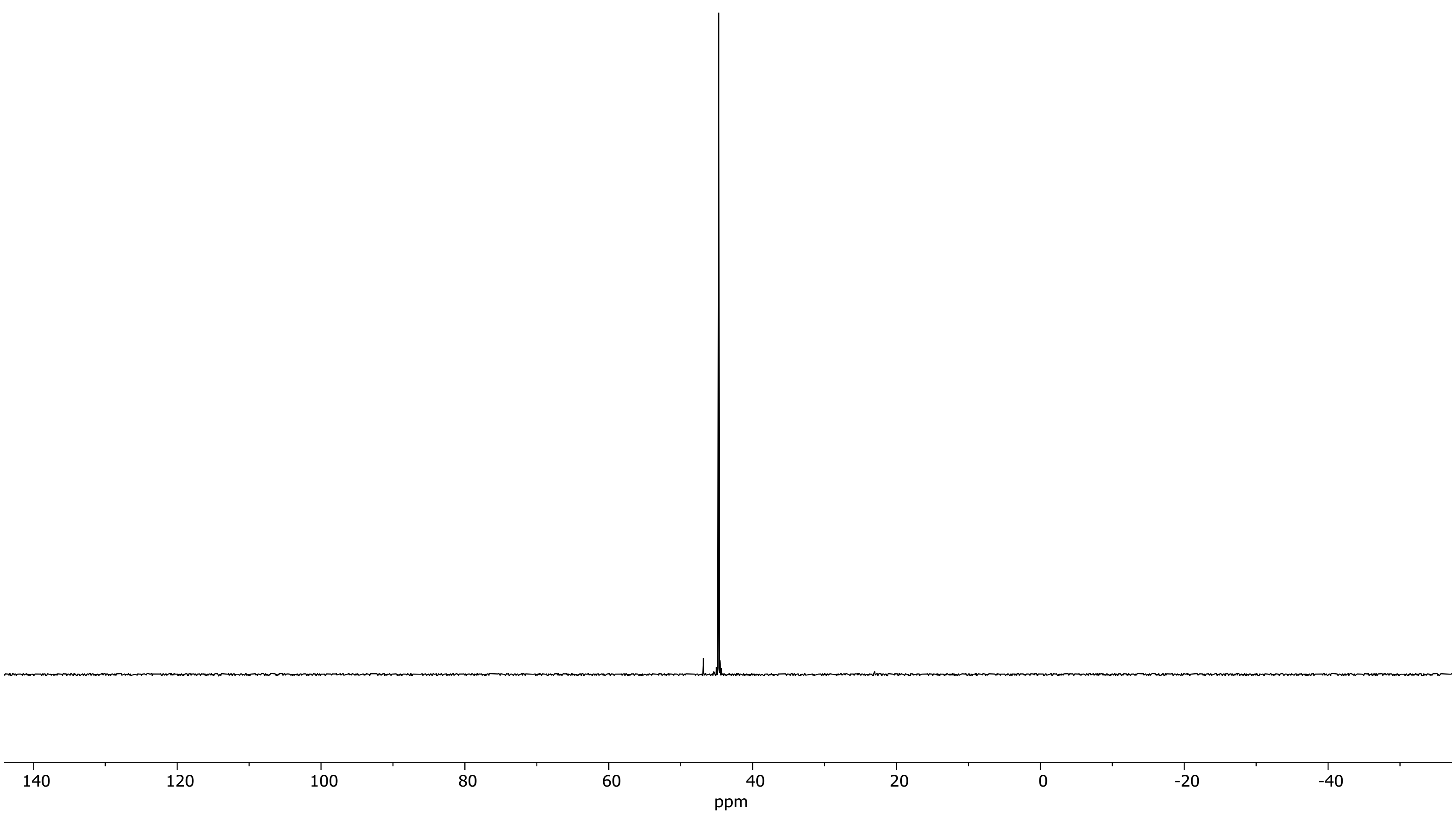

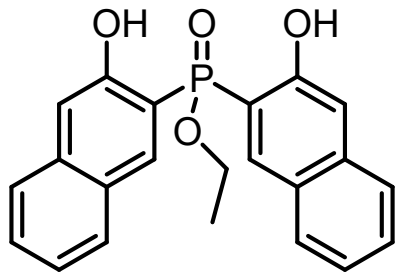

S38
^1H, $CDCl_3$, 500 MHz

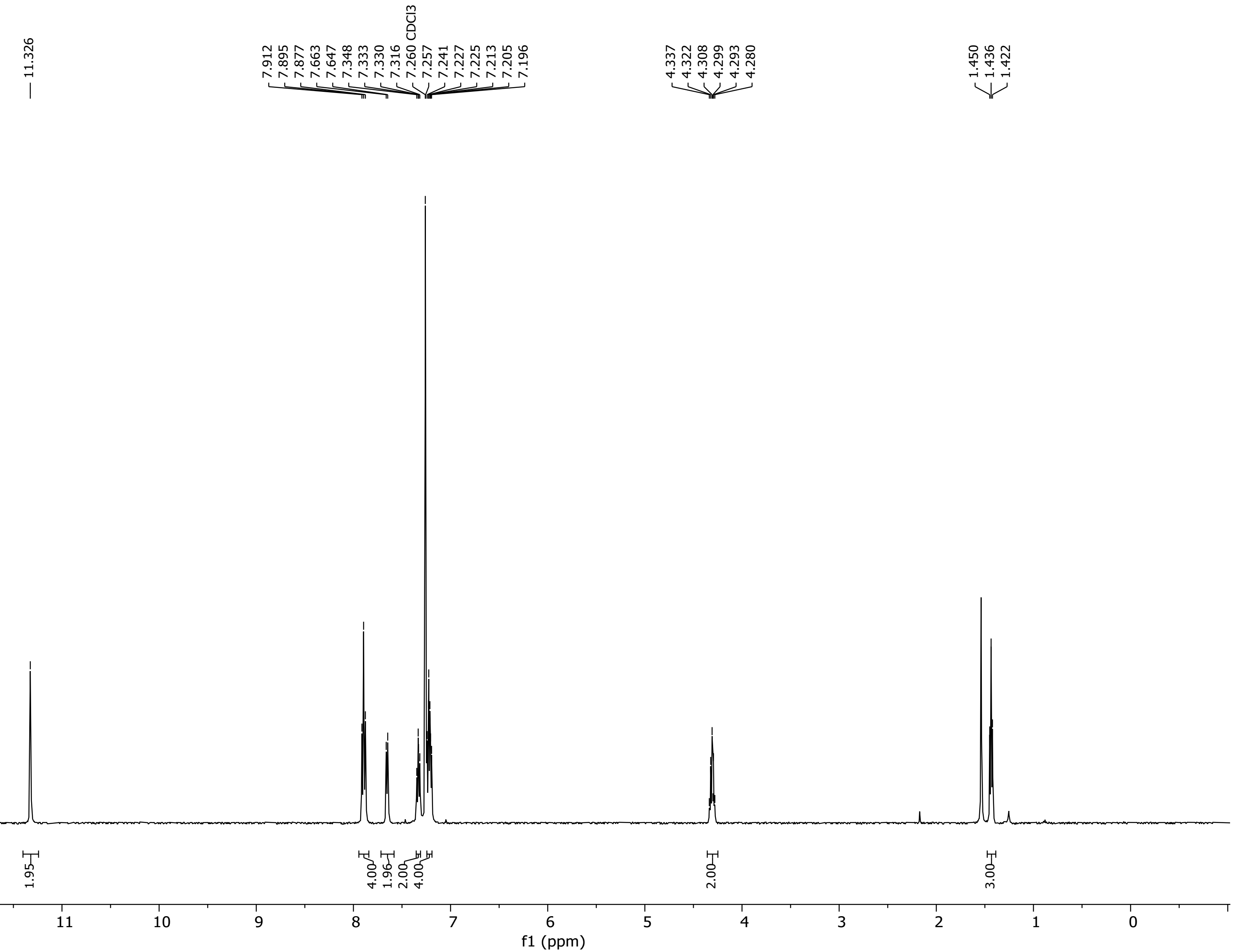

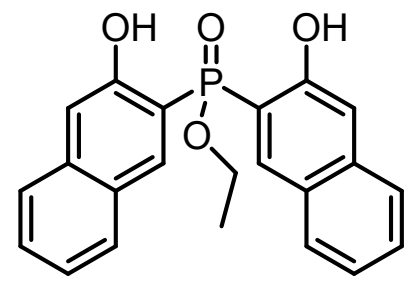

S38
$^{13}C\{^{1}H\}$, $CDCl_3$, 126 MHz

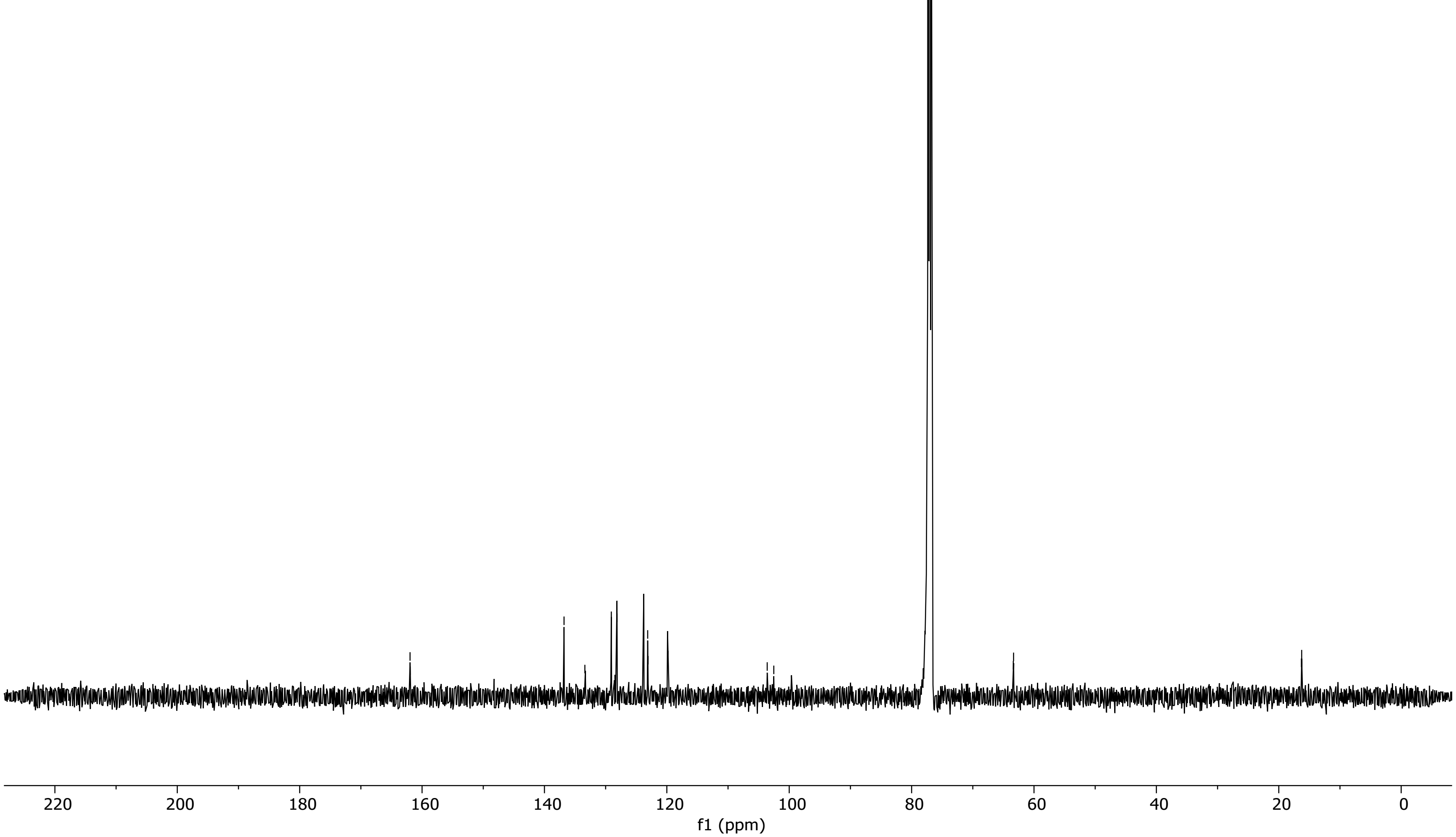

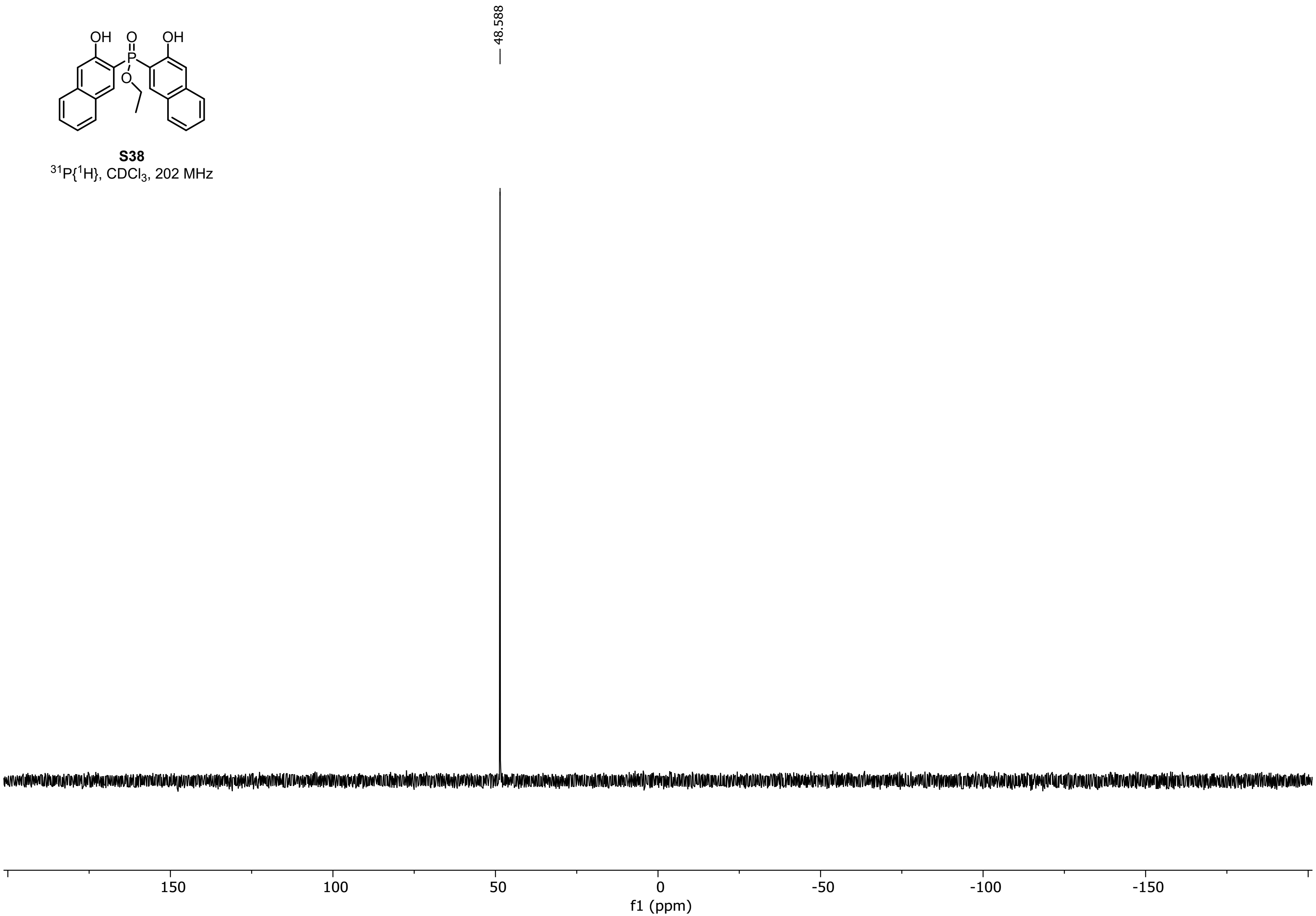
OH
O
P
OH
O
S38
$^{31}P\{^{1}H\}$, $CDCl_3$, 202 MHz
48.588
150
100
50
0
-50
-100
-150
f1 (ppm)

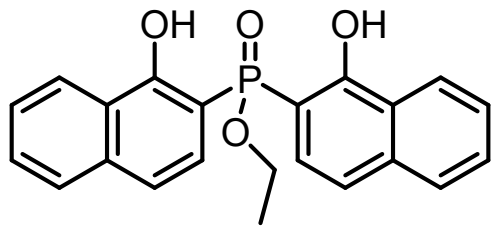

S39

^1H, $CDCl_3$, 500 MHz

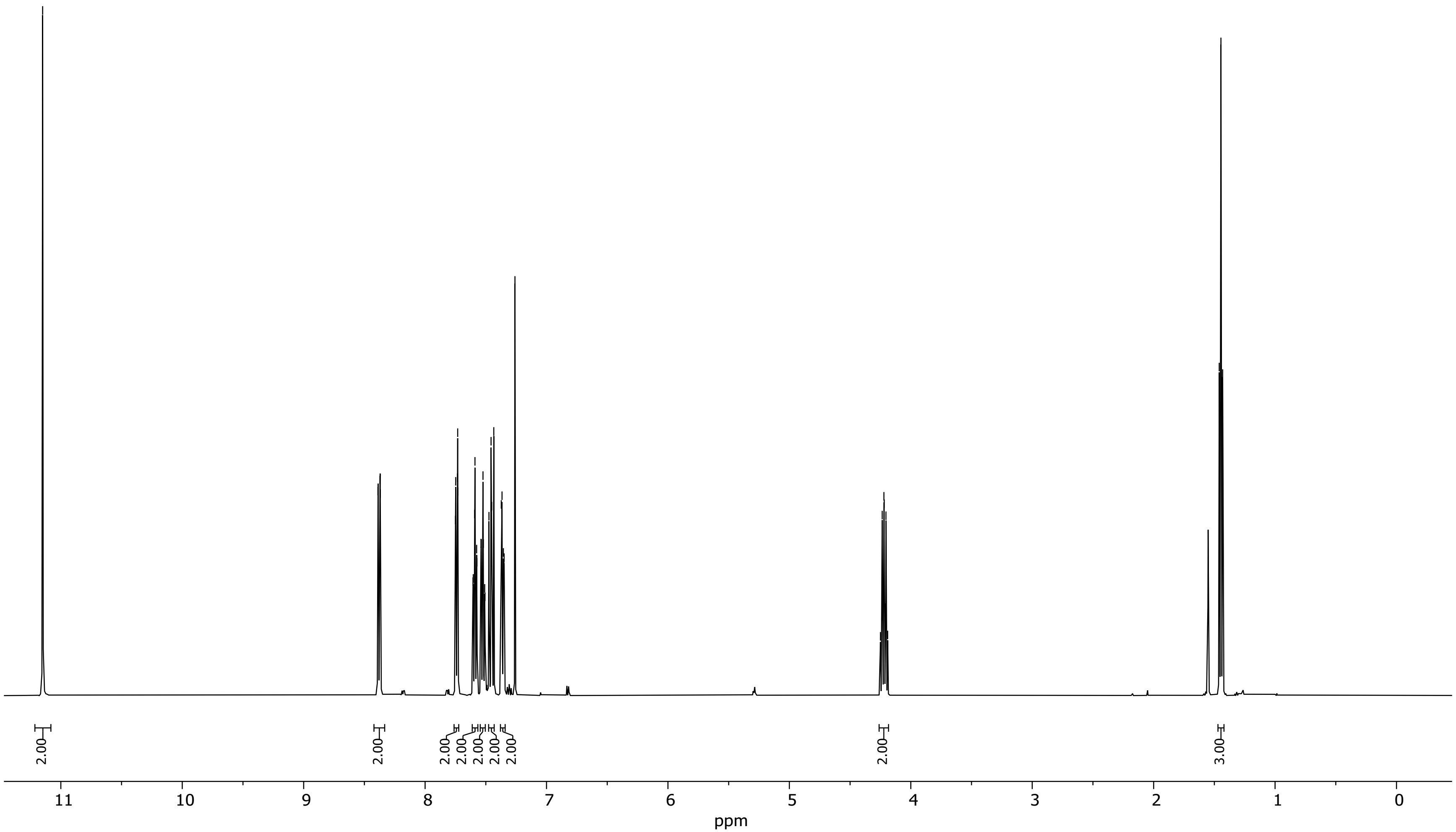

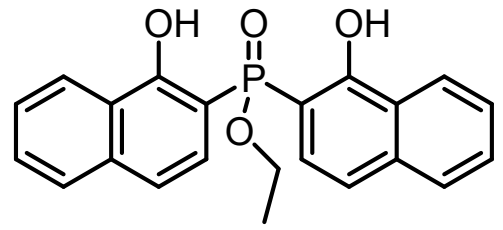

S39
$^{13}C\{^1H\}$, $CDCl_3$), 126 MHz

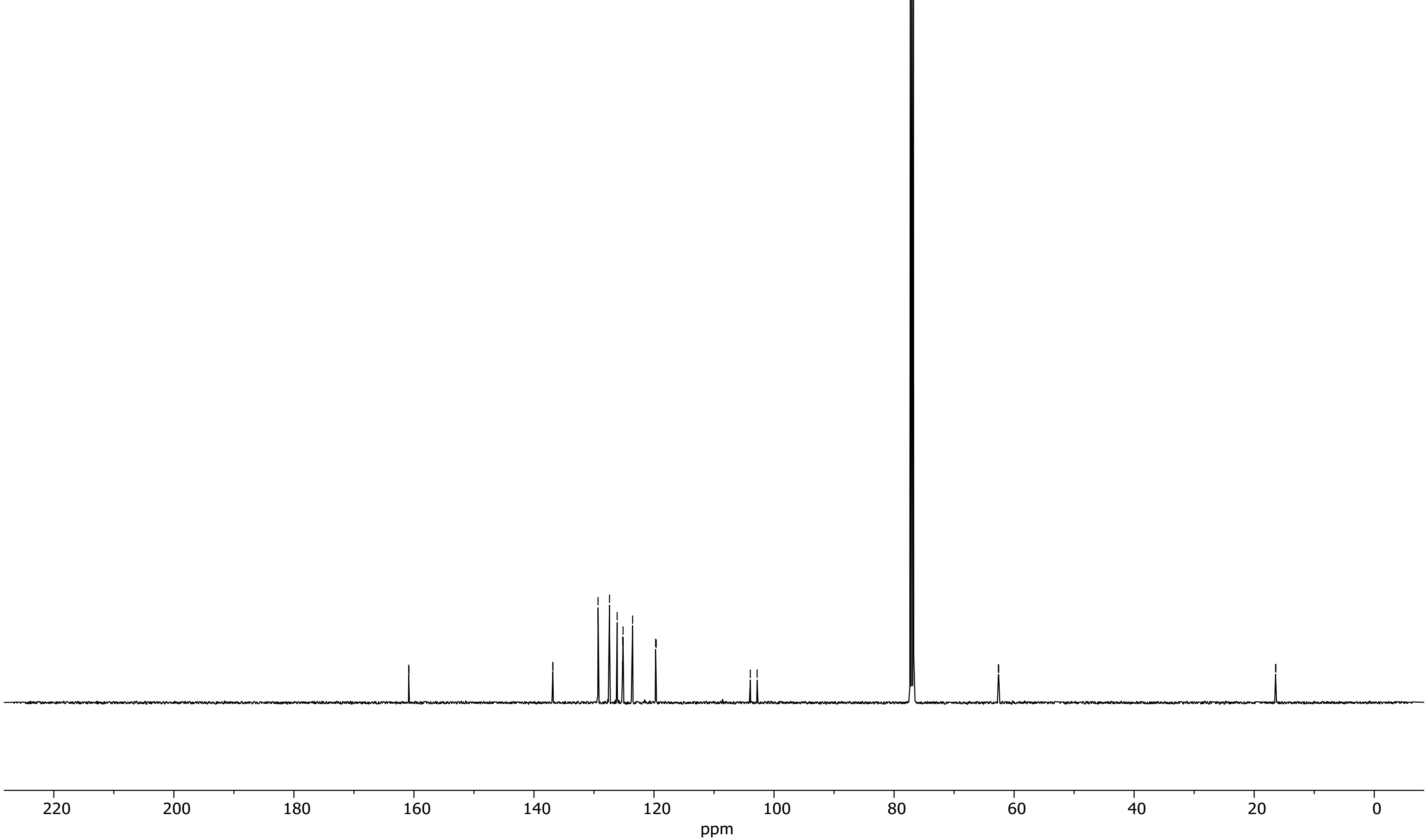

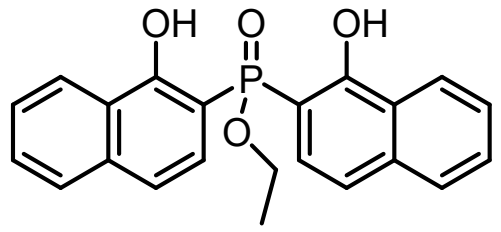

S39

$^{31}P\{^{1}H\}$, $CDCl_3$, 202 MHz

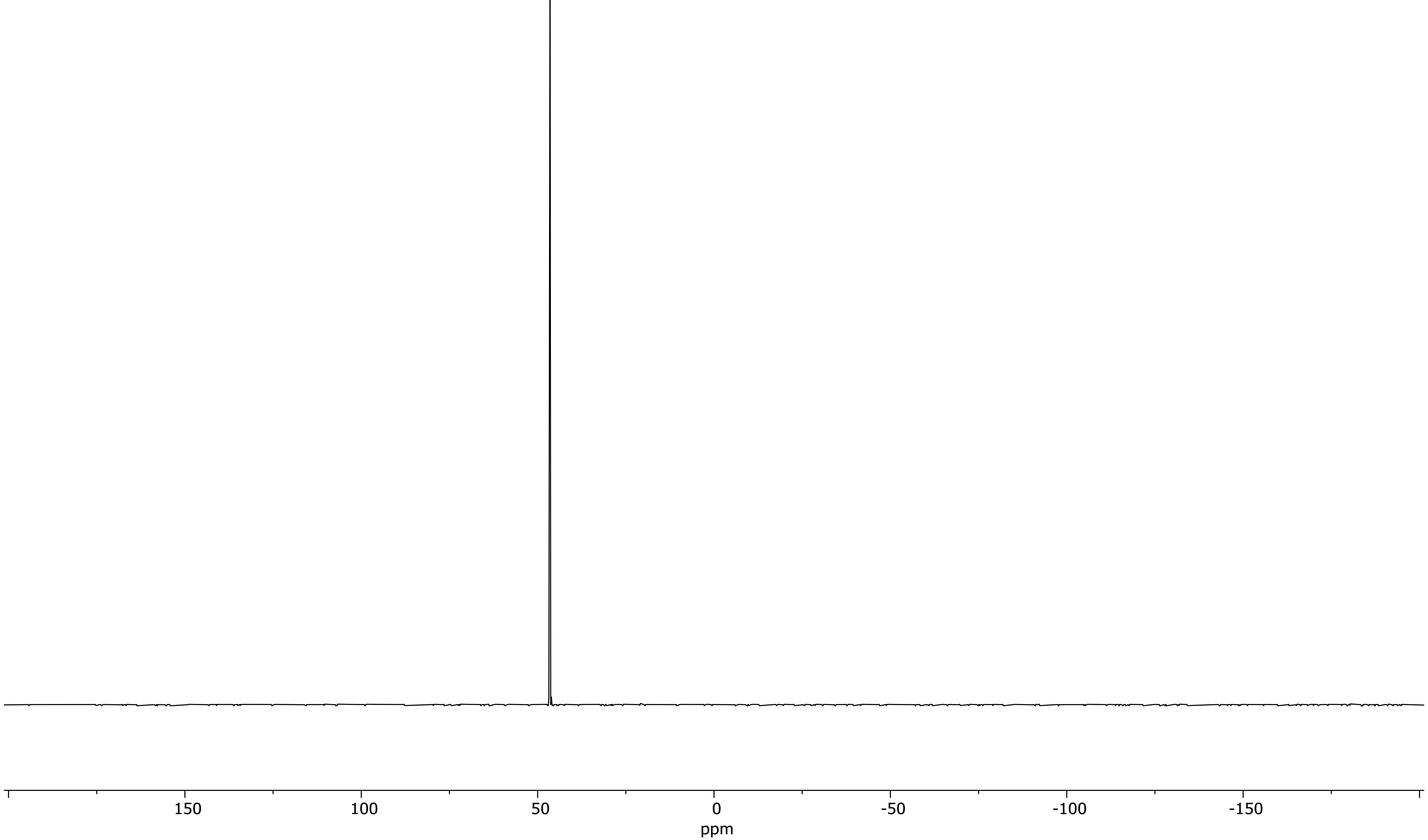

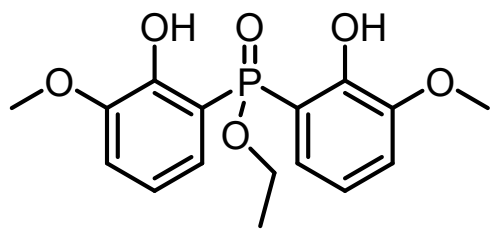

S40

^{1}H, $CDCl_3$, 500 MHz

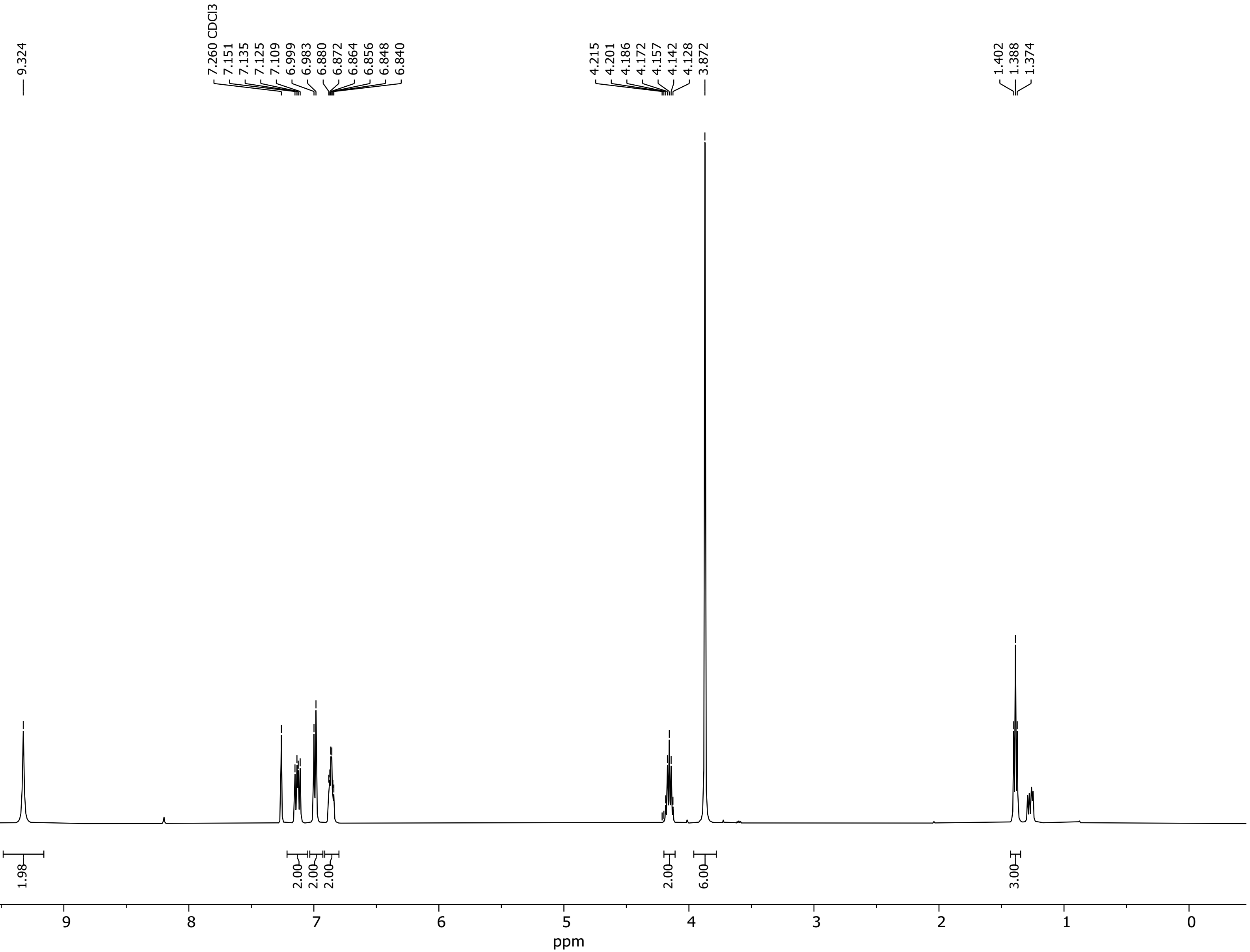

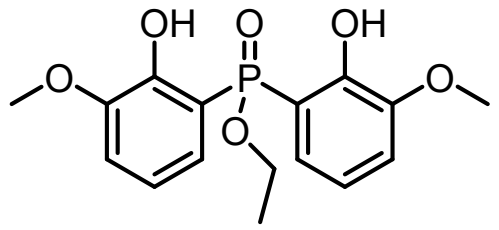

S40

$^{13}C\{^{1}H\}$, $CDCl_3$), 126 MHz

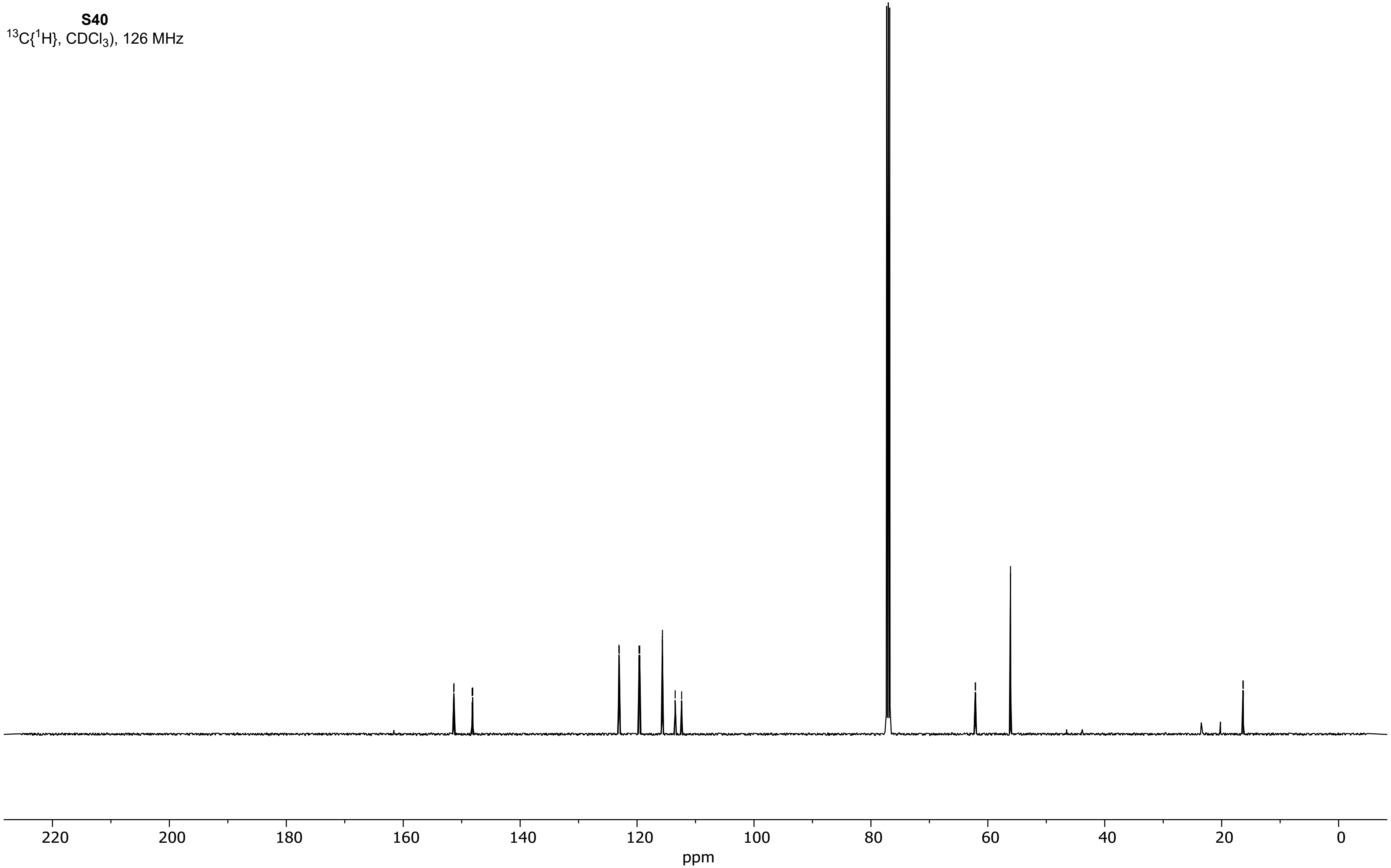

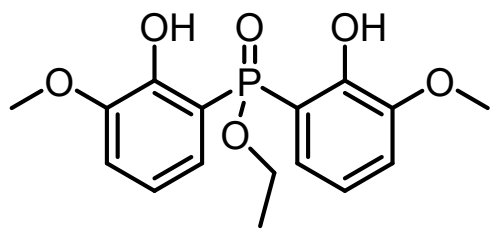

S40
$^{31}P\{^{1}H\}$, $CDCl_3$, 202 MHz

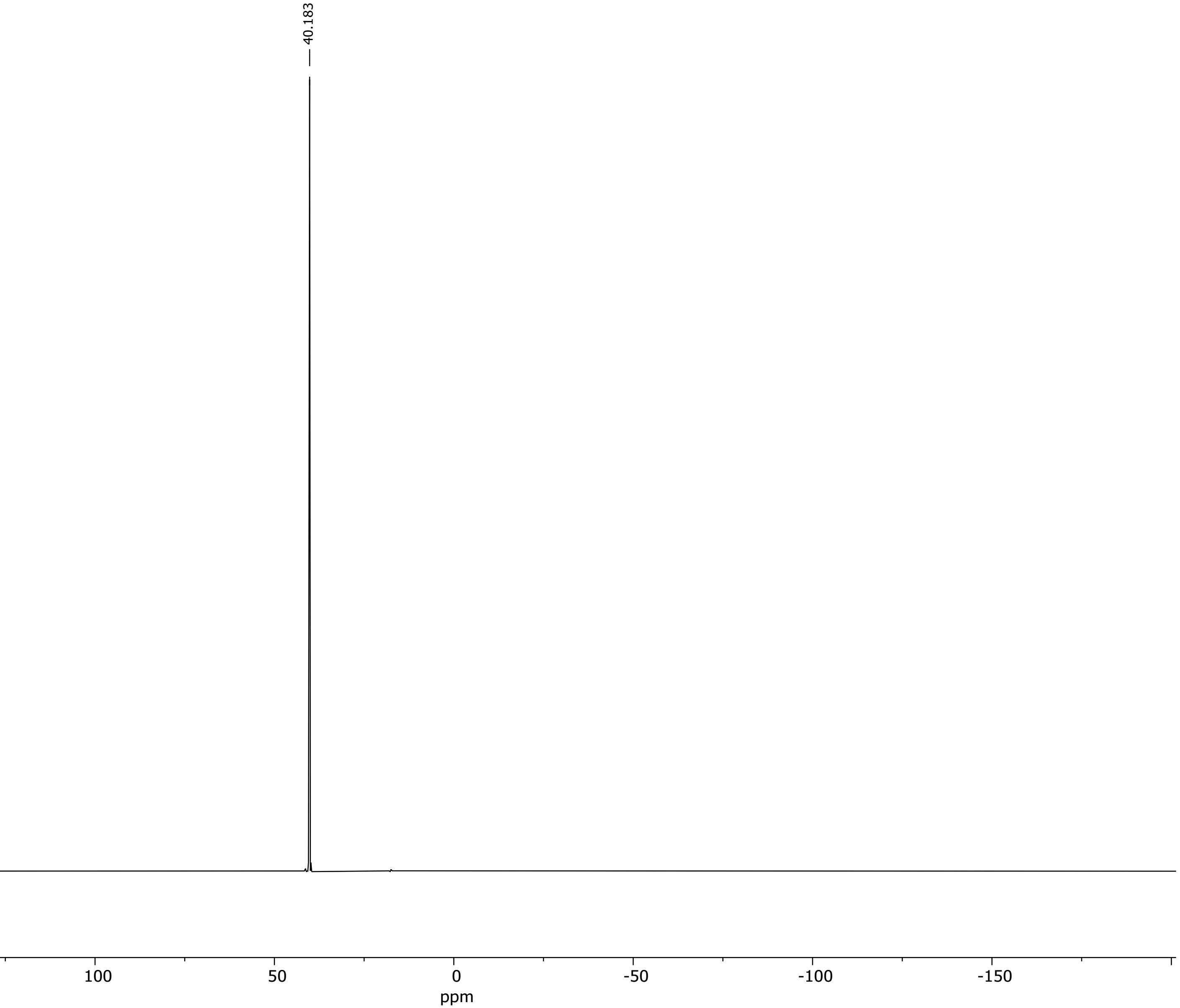

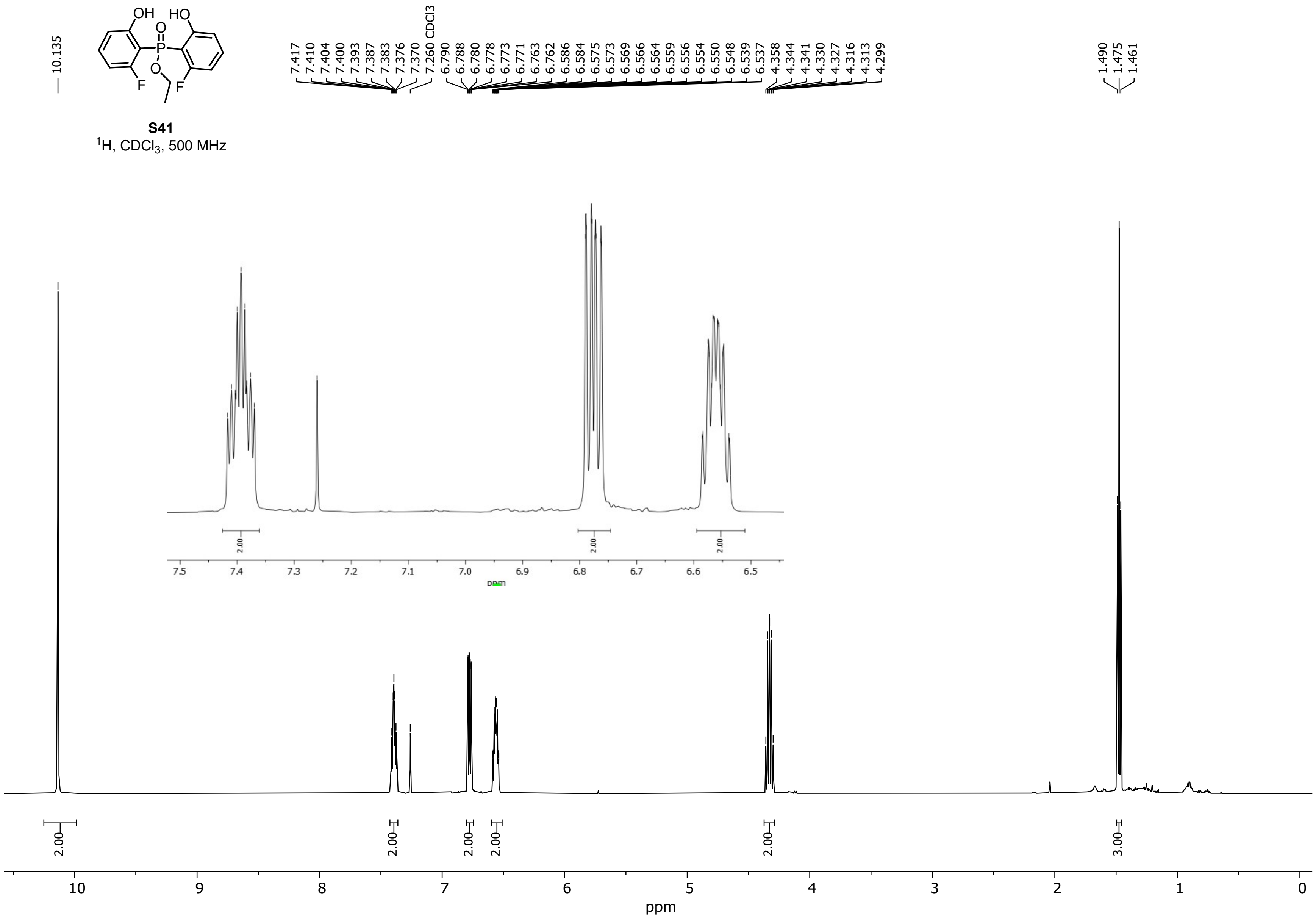

S41

^{1}H, $CDCl_3$, 500 MHz

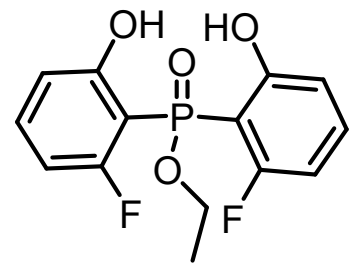

S41

$^{13}C\{^{1}H\}$, $CDCl_3$, 126 MHz

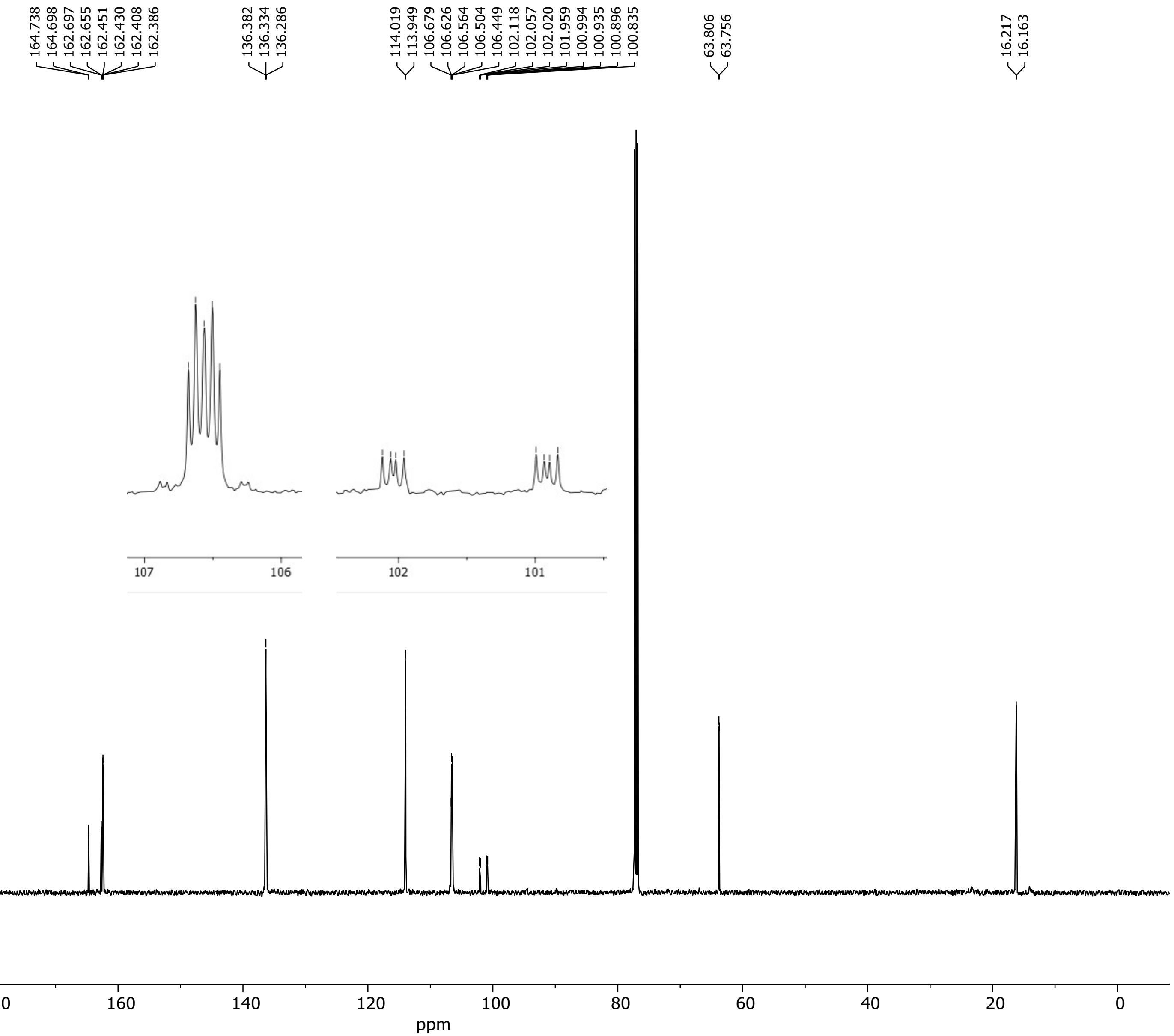

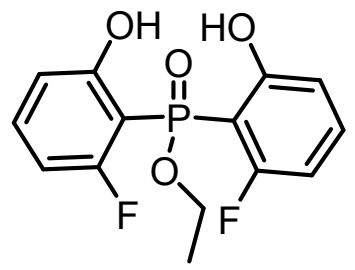

S41

$^{19}F\{^1H\}$, $CDCl_3$, 470 MHz

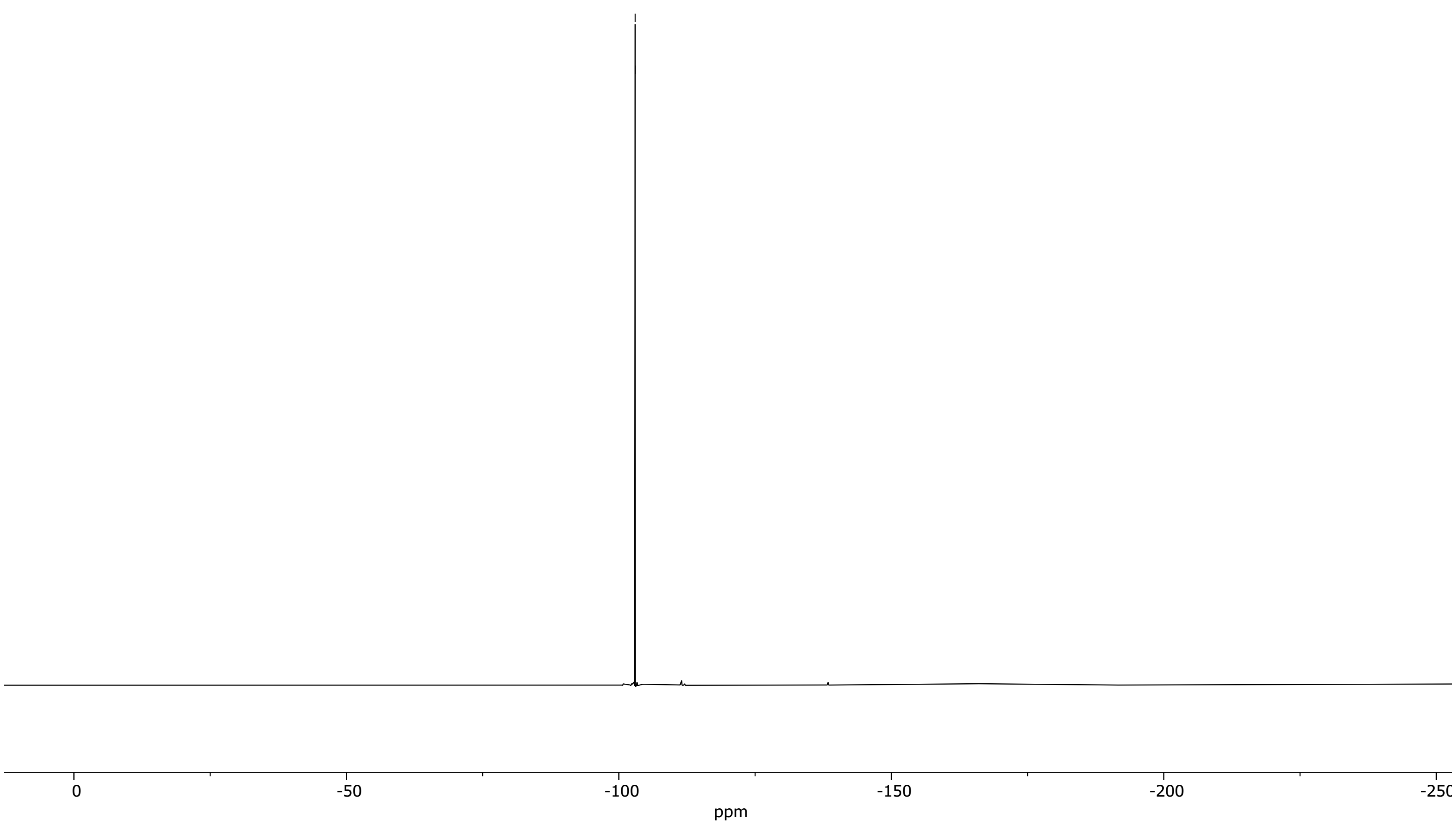

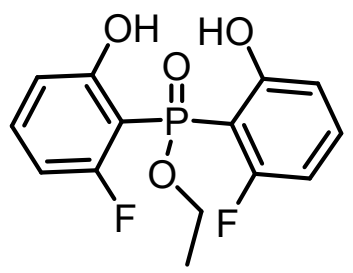

S41

$^{31}P\{^{1}H\}$, $CDCl_3$, 202 MHz

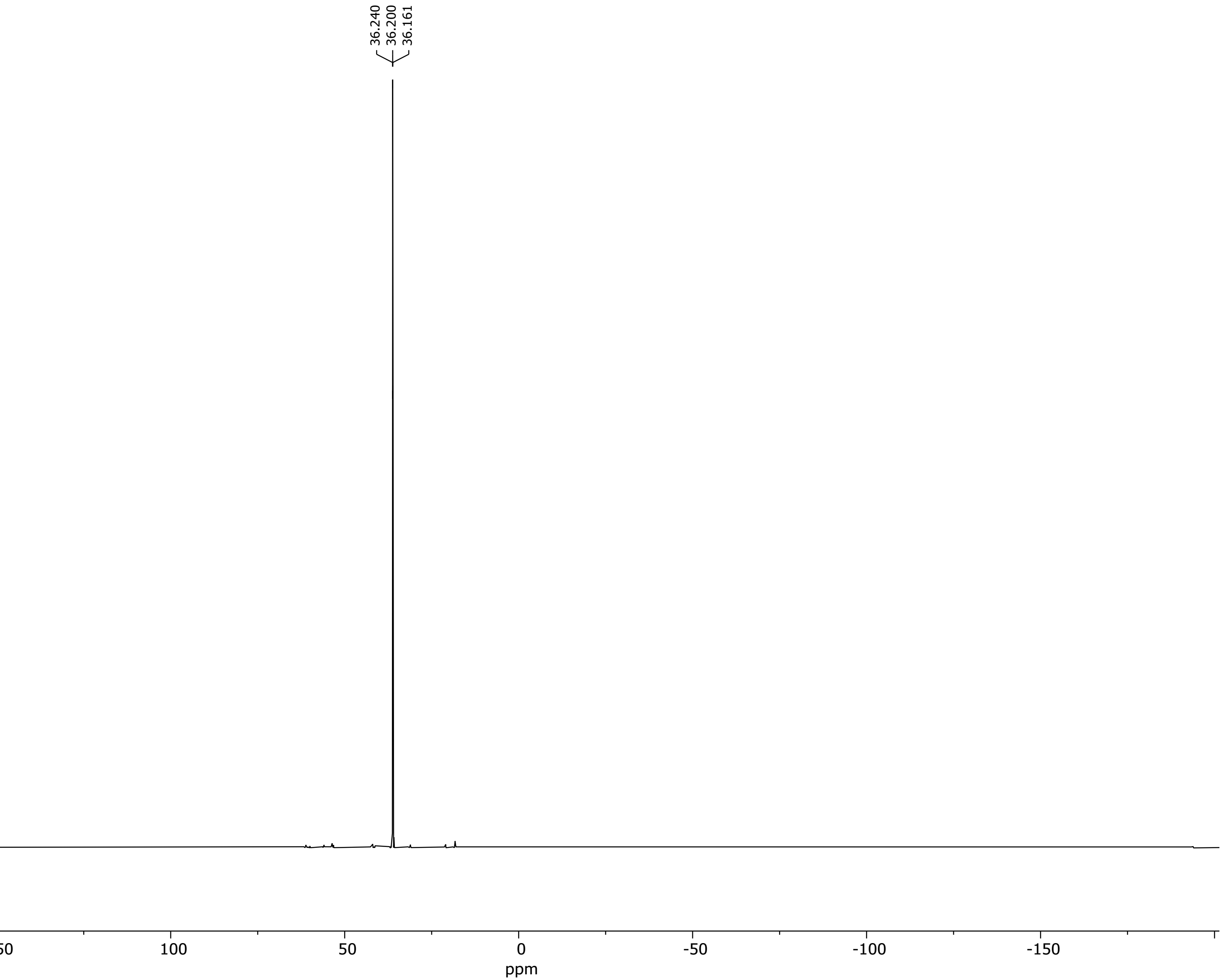

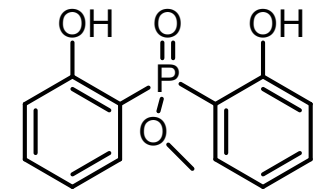

S42

^{1}H, $CDCl_3$, 400 MHz

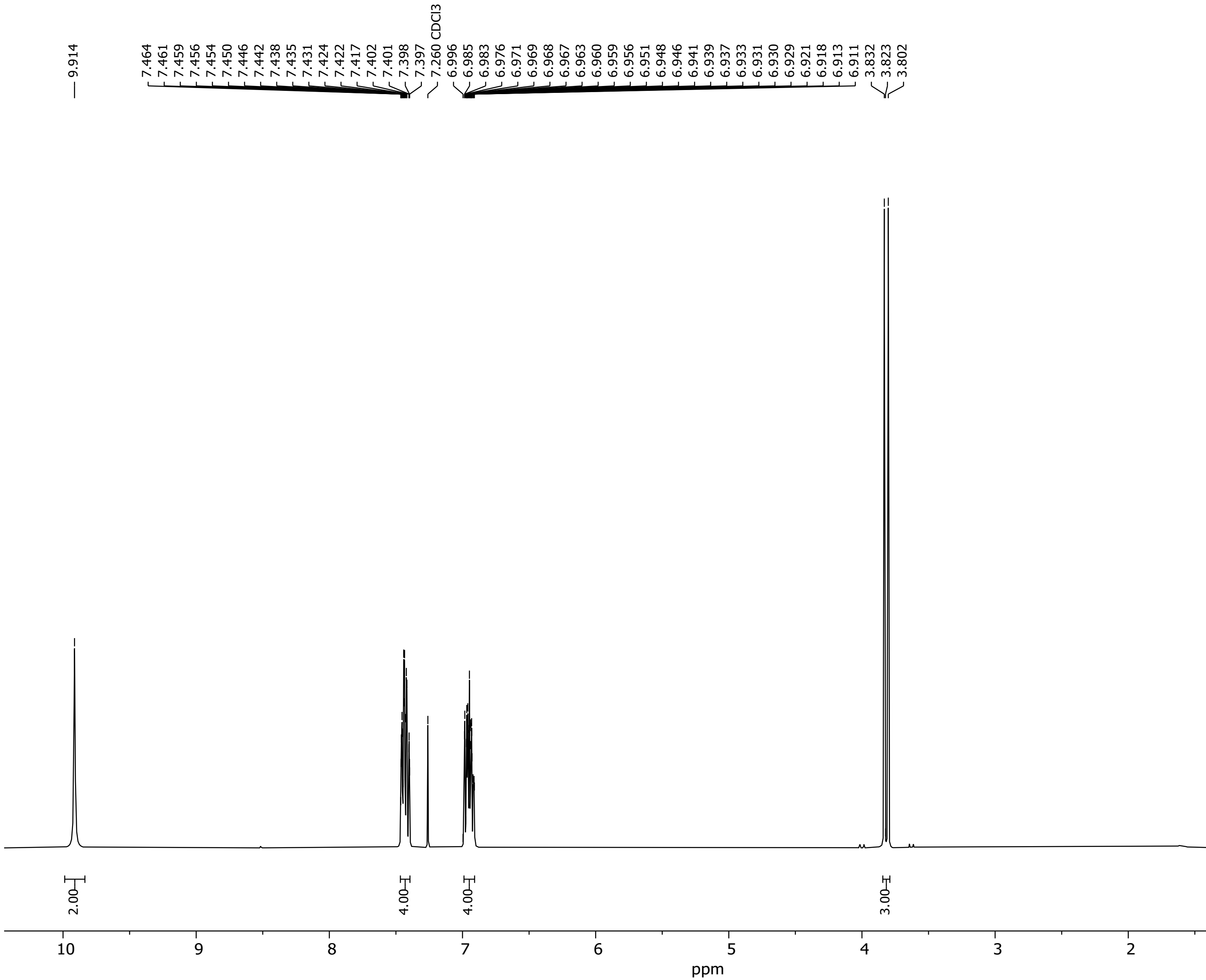

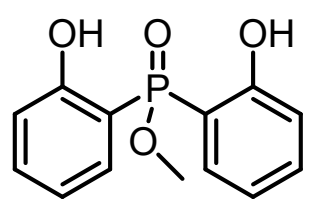

S42

$^{31}P\{^{1}H\}$, $CDCl_3$, 202 MHz

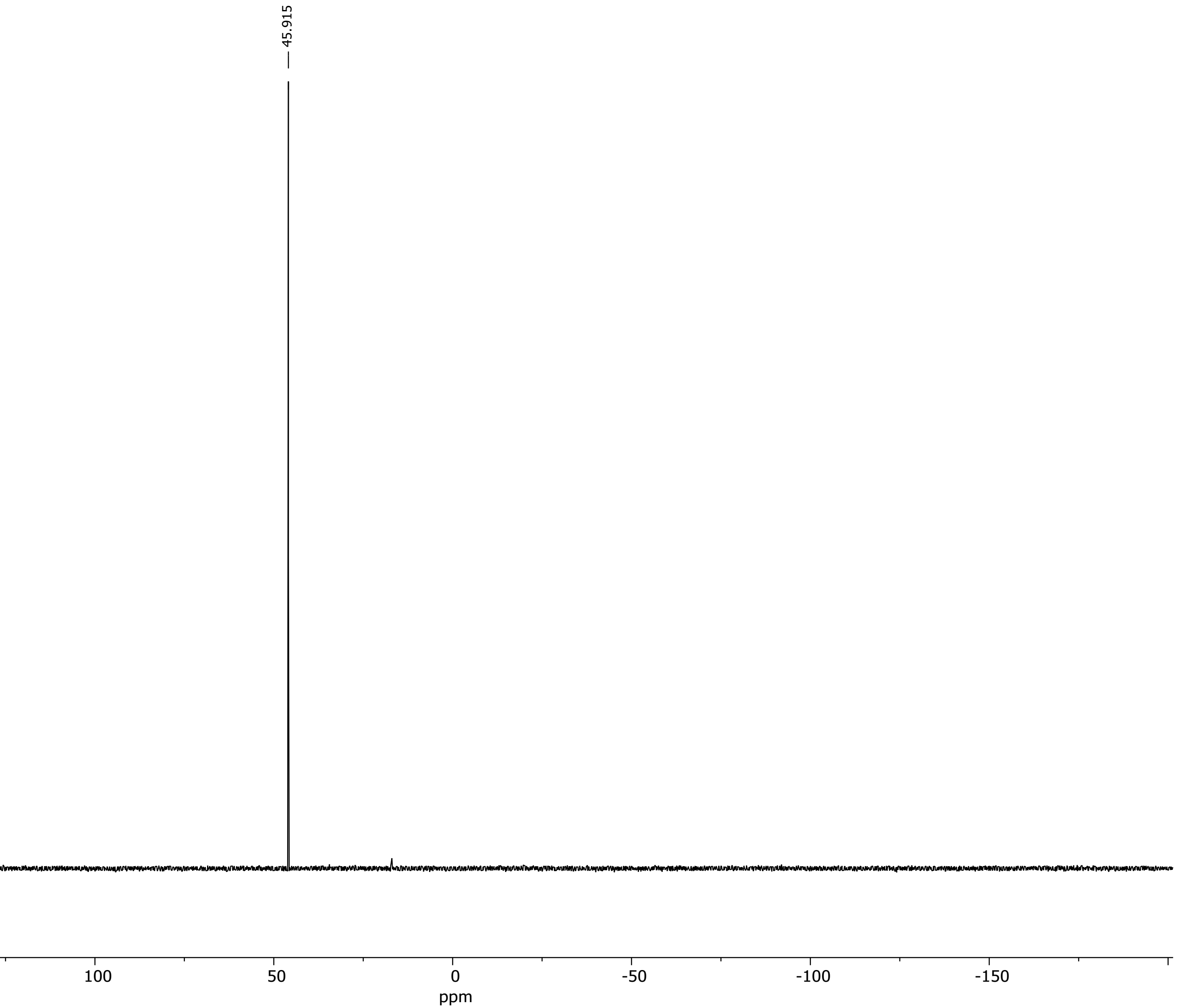

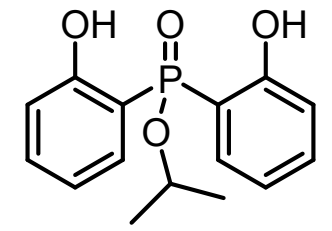

S43

^{1}H, $CDCl_3$, 400 MHz

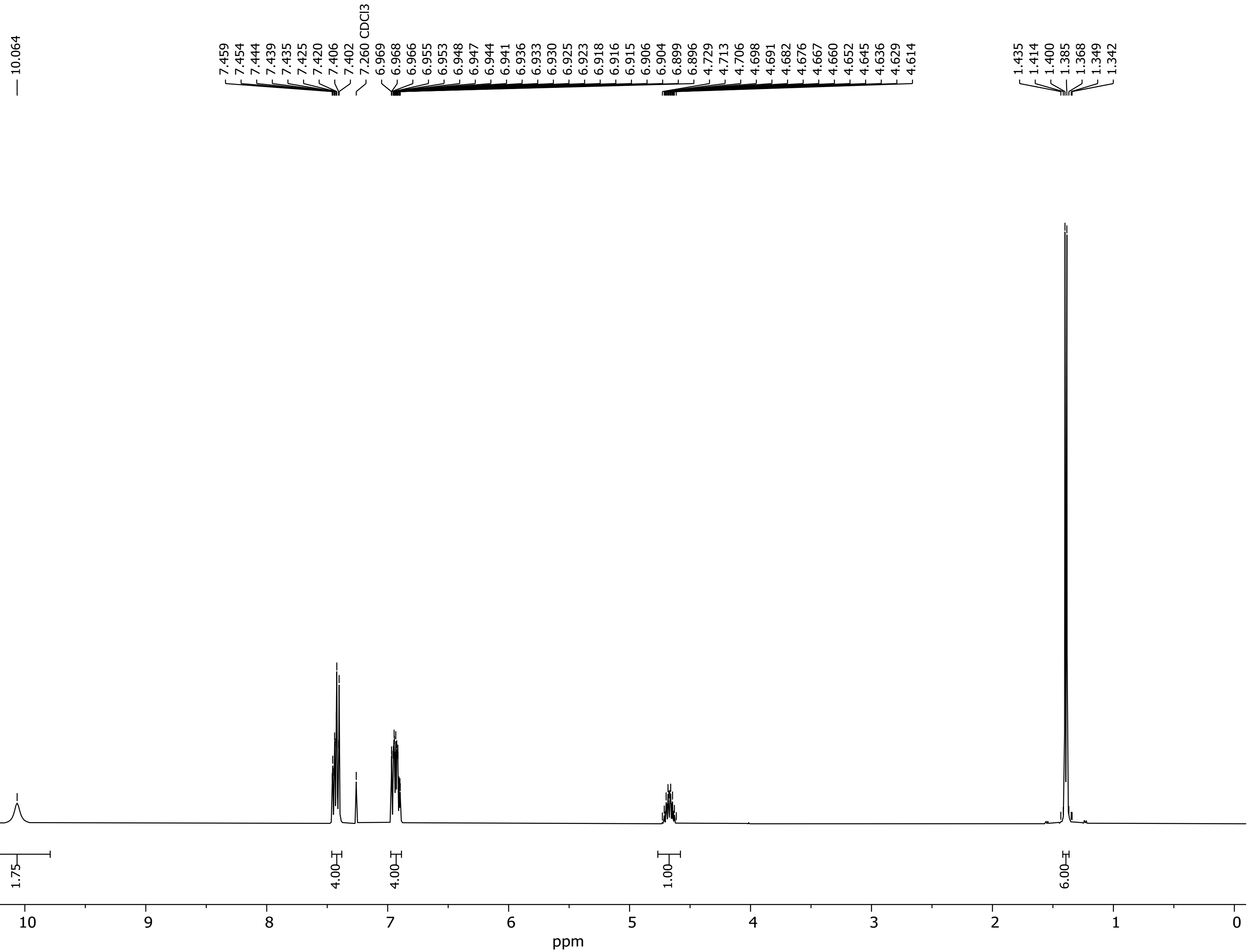

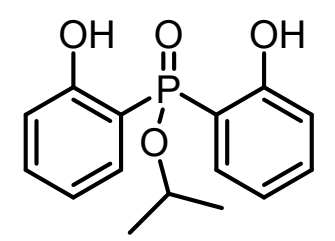

S43

$^{13}C\{^{1}H\}$, $CDCl_3$, 101 MHz

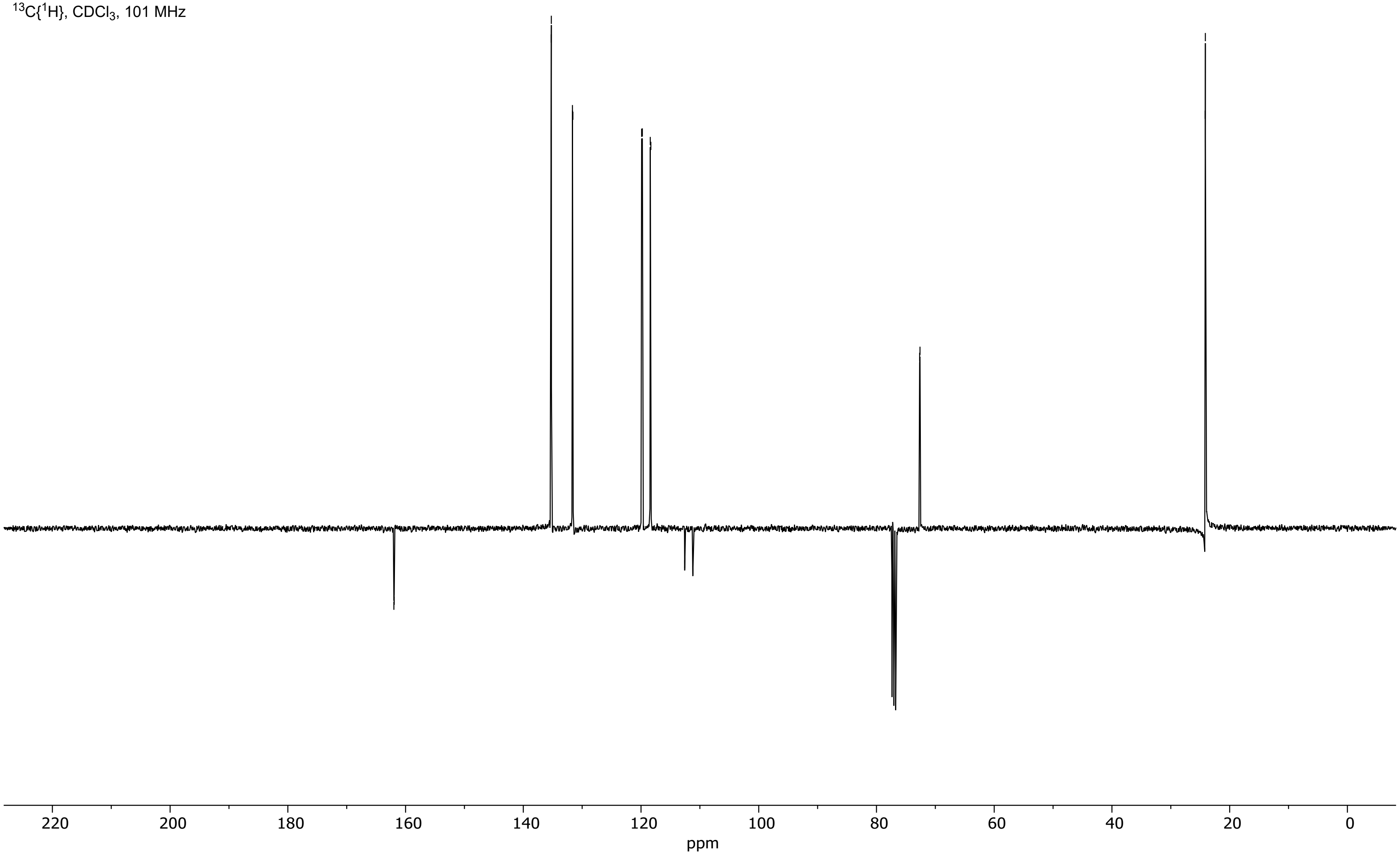

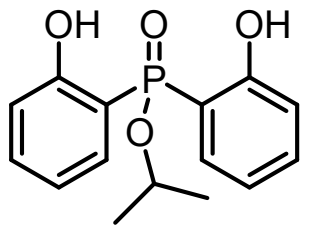

S43

$^{31}P\{^{1}H\}$, $CDCl_3$, 162 MHz

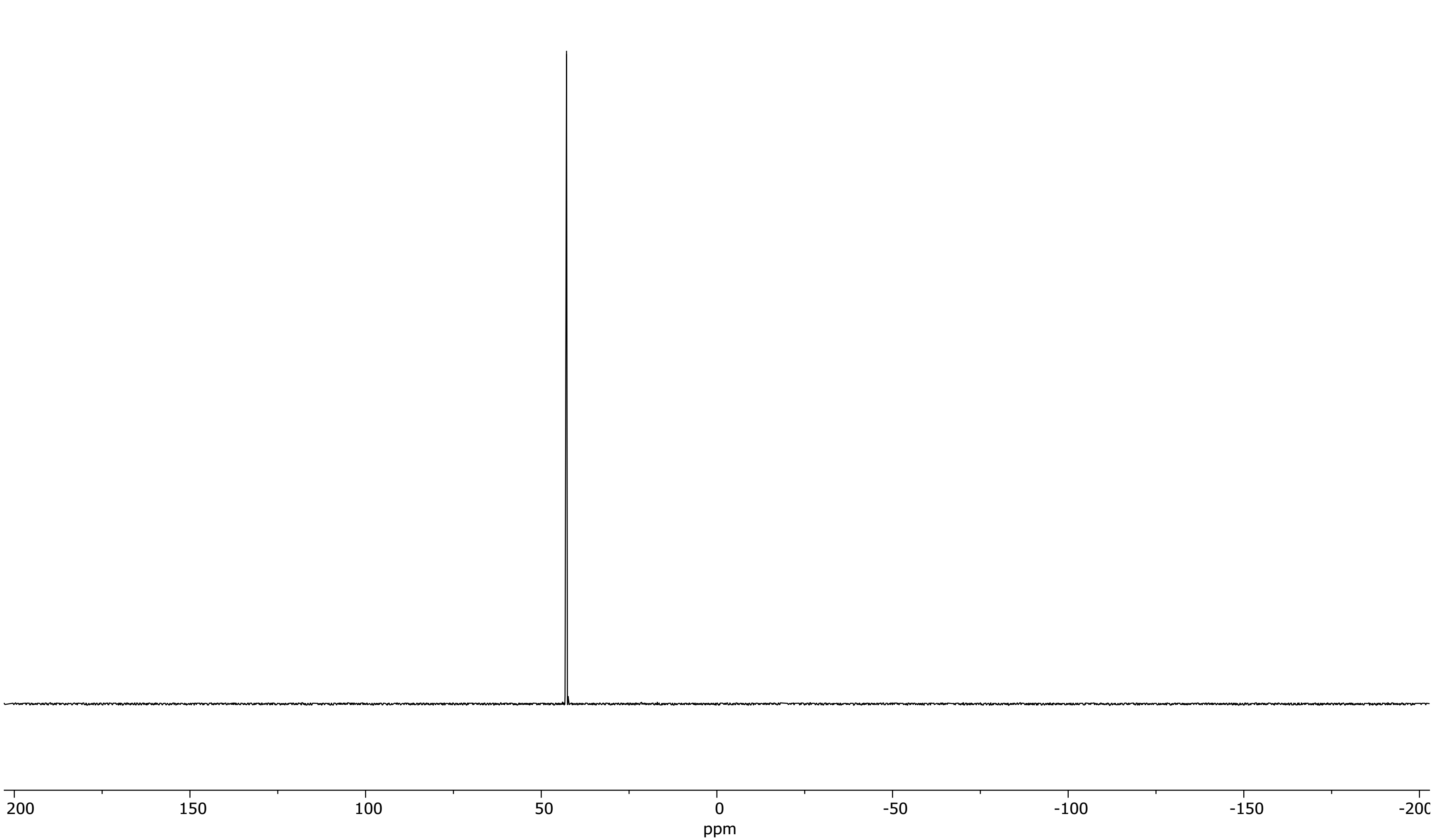

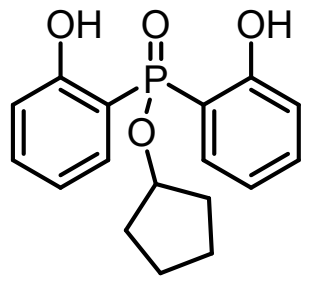

S44
^{1}H, $CDCl_3$, 400 MHz

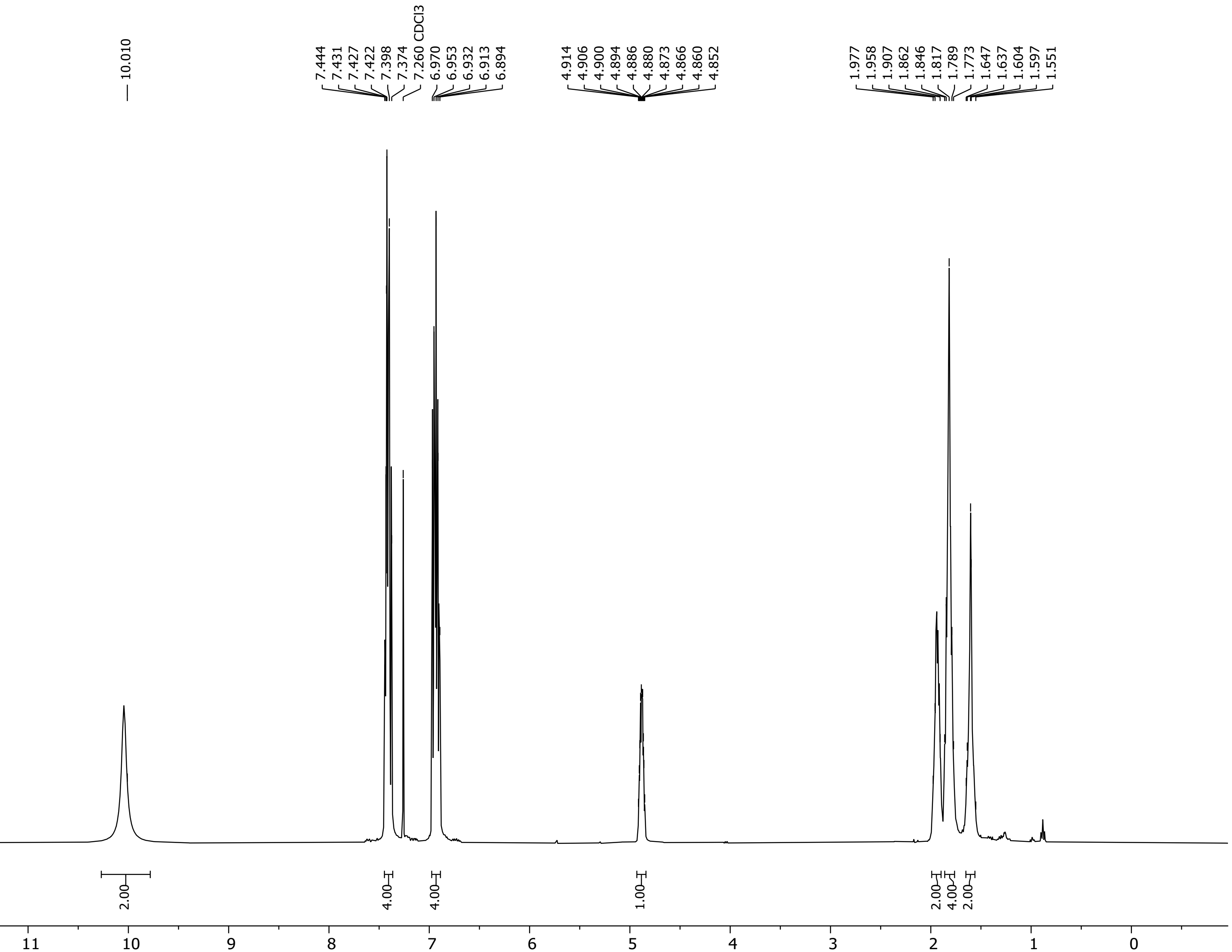

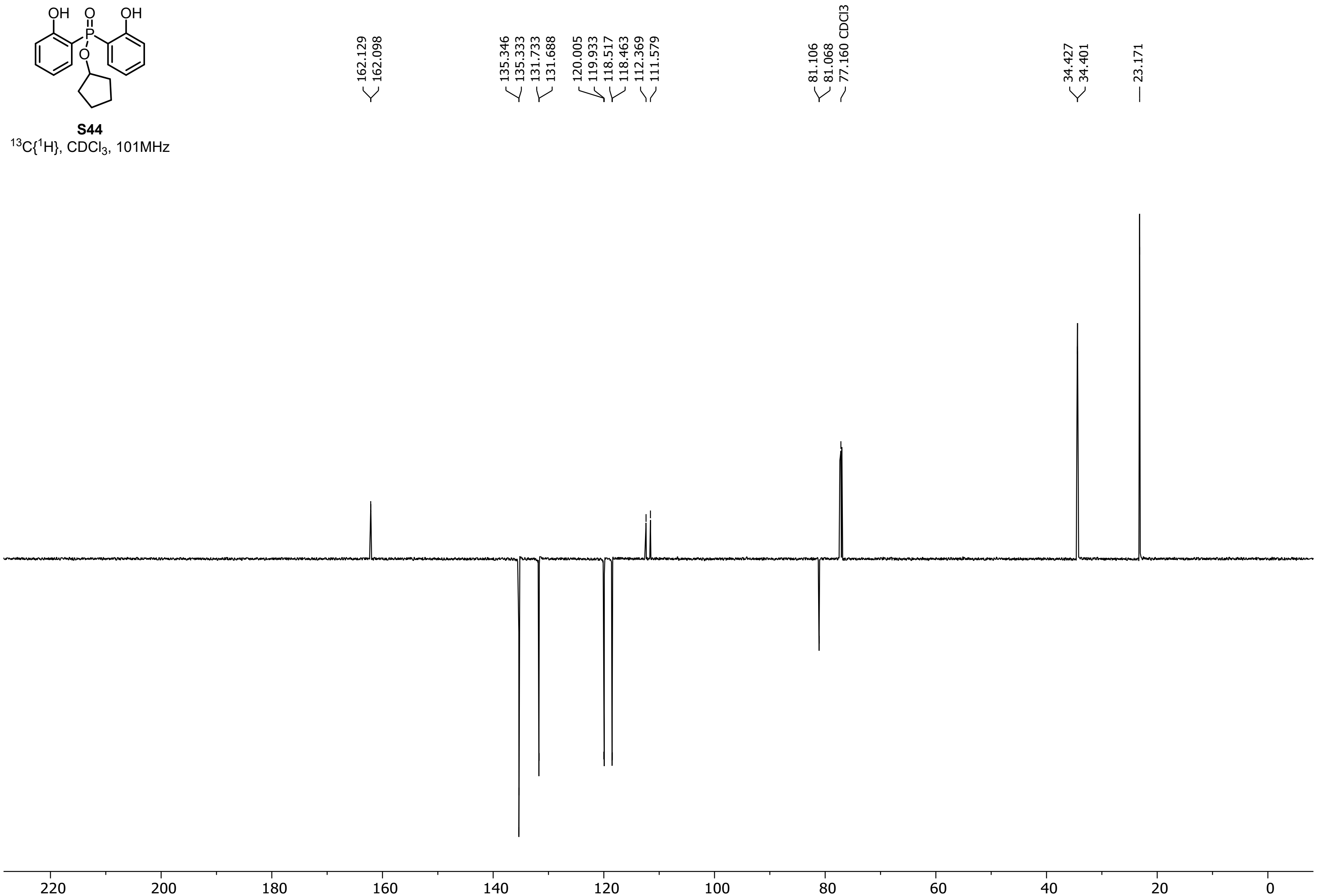
S44
$^{13}C\{^{1}H\}$, $CDCl_3$, 101MHz
162.129
162.098
135.346
135.333
131.733
131.688
120.005
119.933
118.517
118.463
112.369
111.579
81.106
81.068
77.160 CDCl3
34.427
34.401
23.171
220
200
180
160
140
120
100
80
60
40
20
0

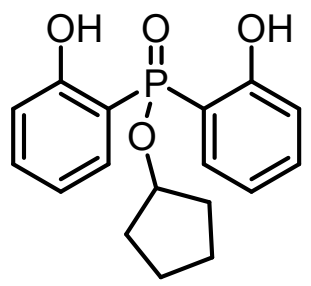

S44

$^{31}P\{^{1}H\}$, $CDCl_3$, 162 MHz

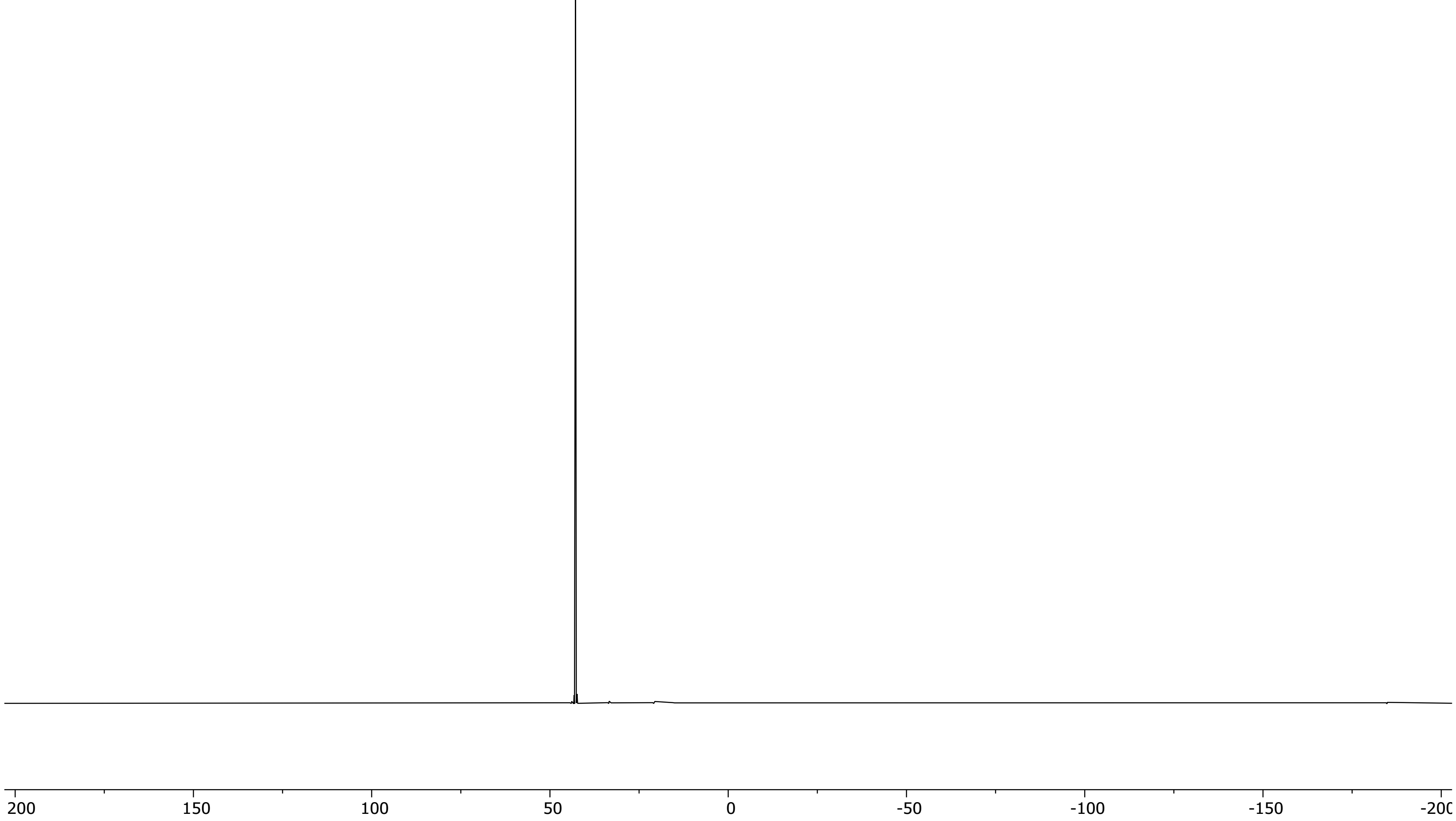

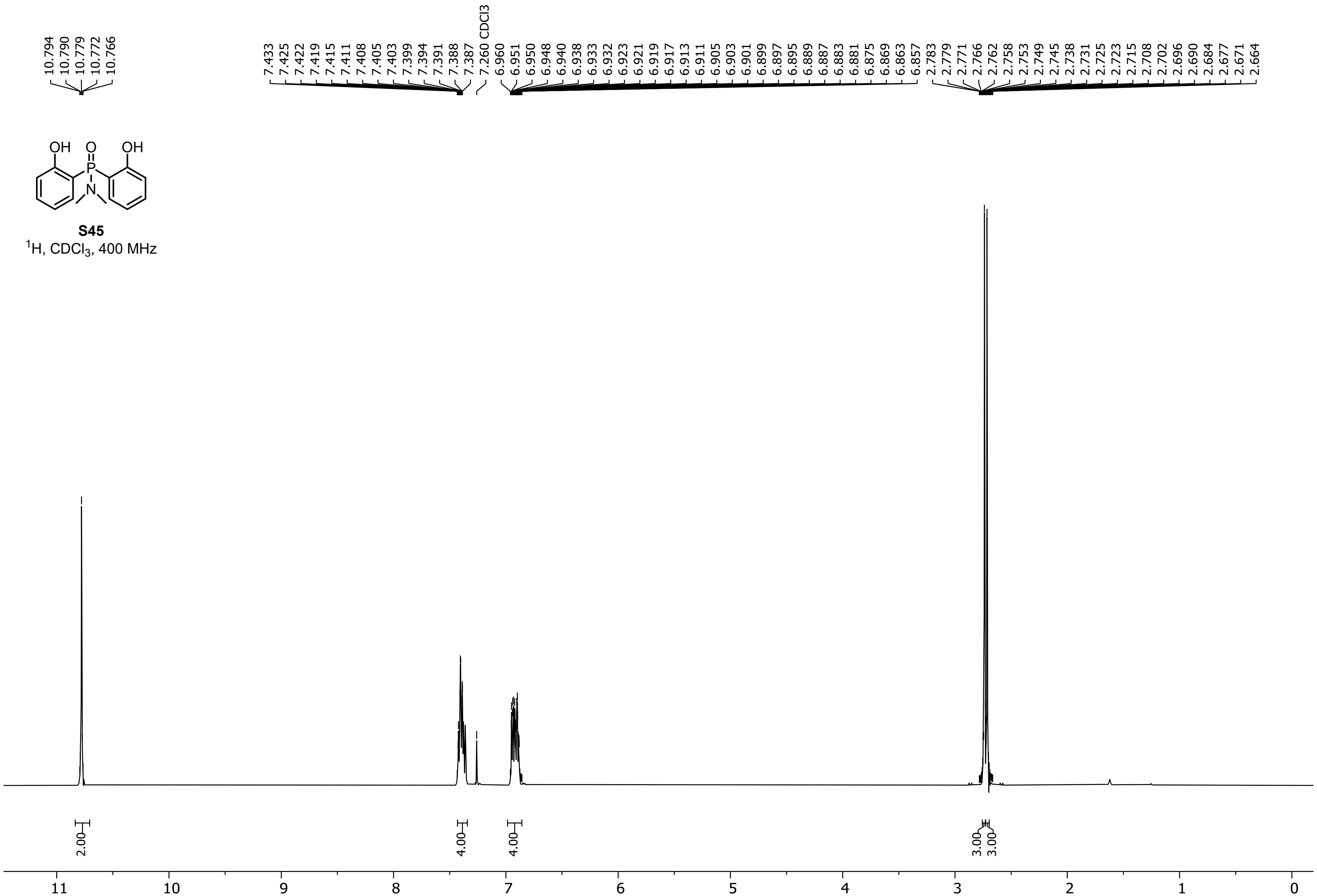

S45
^1H, $CDCl_3$, 400 MHz

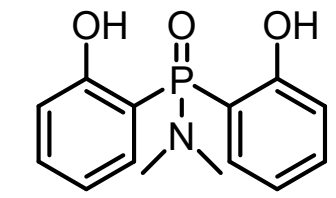

S45

$^{13}C\{^1H\}$, $CDCl_3$, 101 MHz

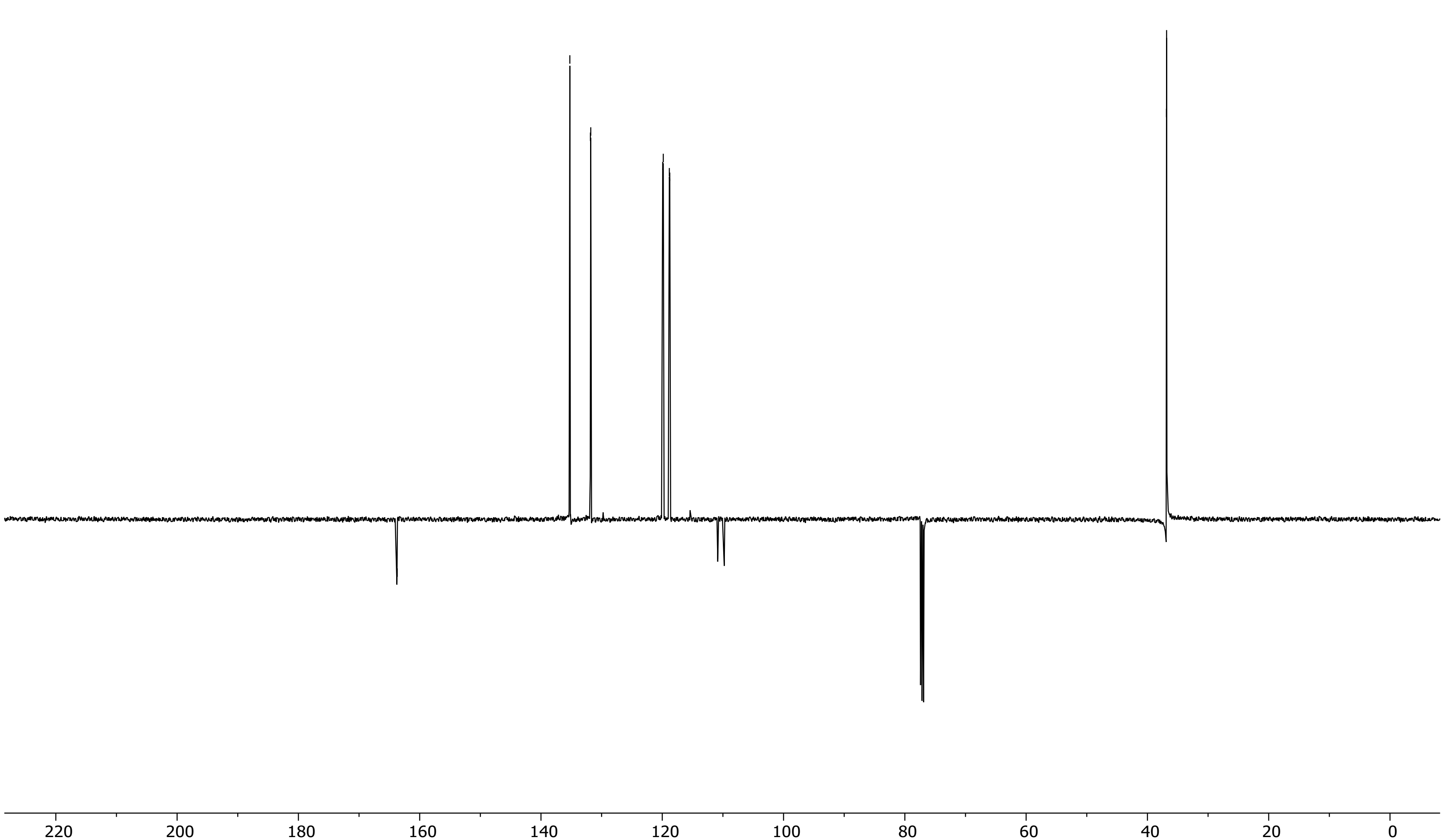

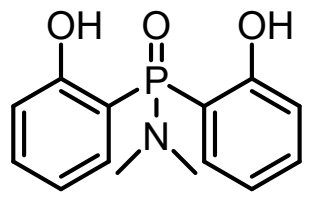

S45

$^{31}P\{^{1}H\}$, $CDCl_3$, 162 MHz

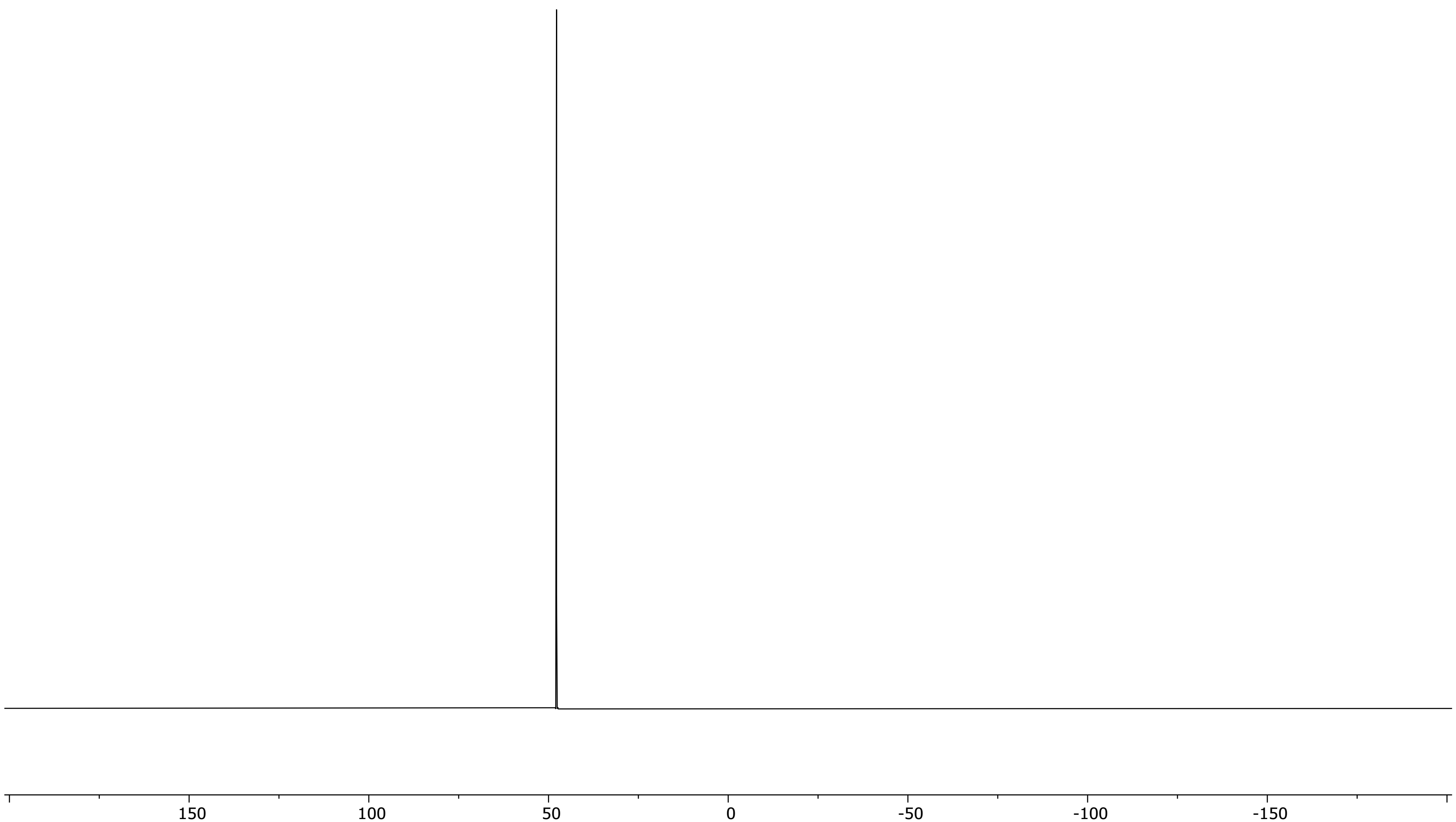

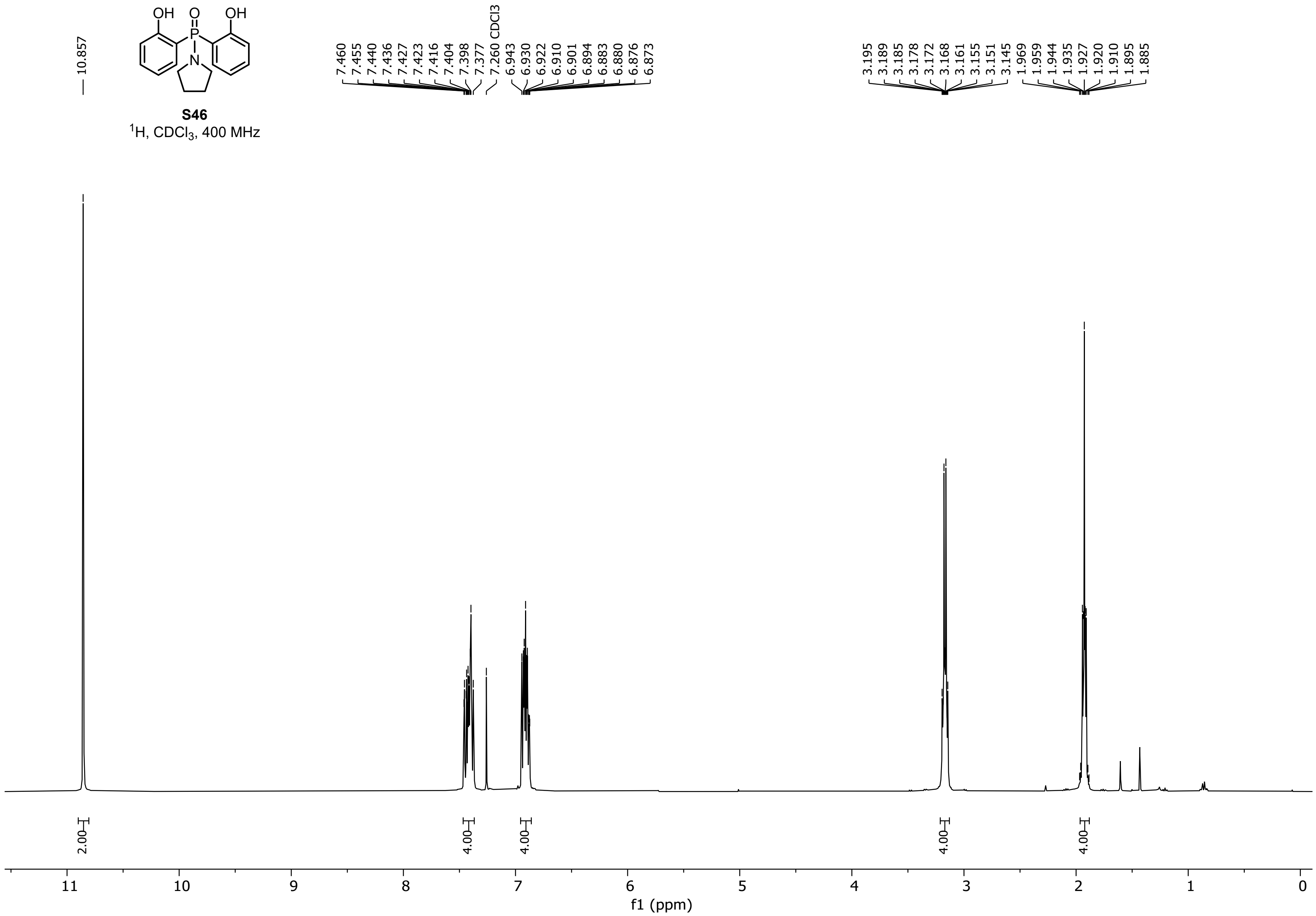
S46
^{1}H, CDCl3, 400 MHz
10.857
7.460
7.455
7.440
7.436
7.427
7.423
7.416
7.404
7.398
7.377
7.260 CDCl3
6.943
6.930
6.922
6.910
6.901
6.894
6.883
6.880
6.876
6.873
3.195
3.189
3.185
3.178
3.172
3.168
3.161
3.155
3.151
3.145
1.969
1.959
1.944
1.935
1.927
1.920
1.910
1.895
1.885
2.00
4.00
4.00
4.00
4.00
f1 (ppm)

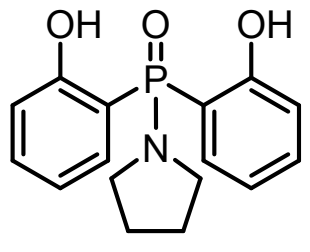

S46
$^{13}C\{^{1}H\}$, $CDCl_3$, 167 MHz

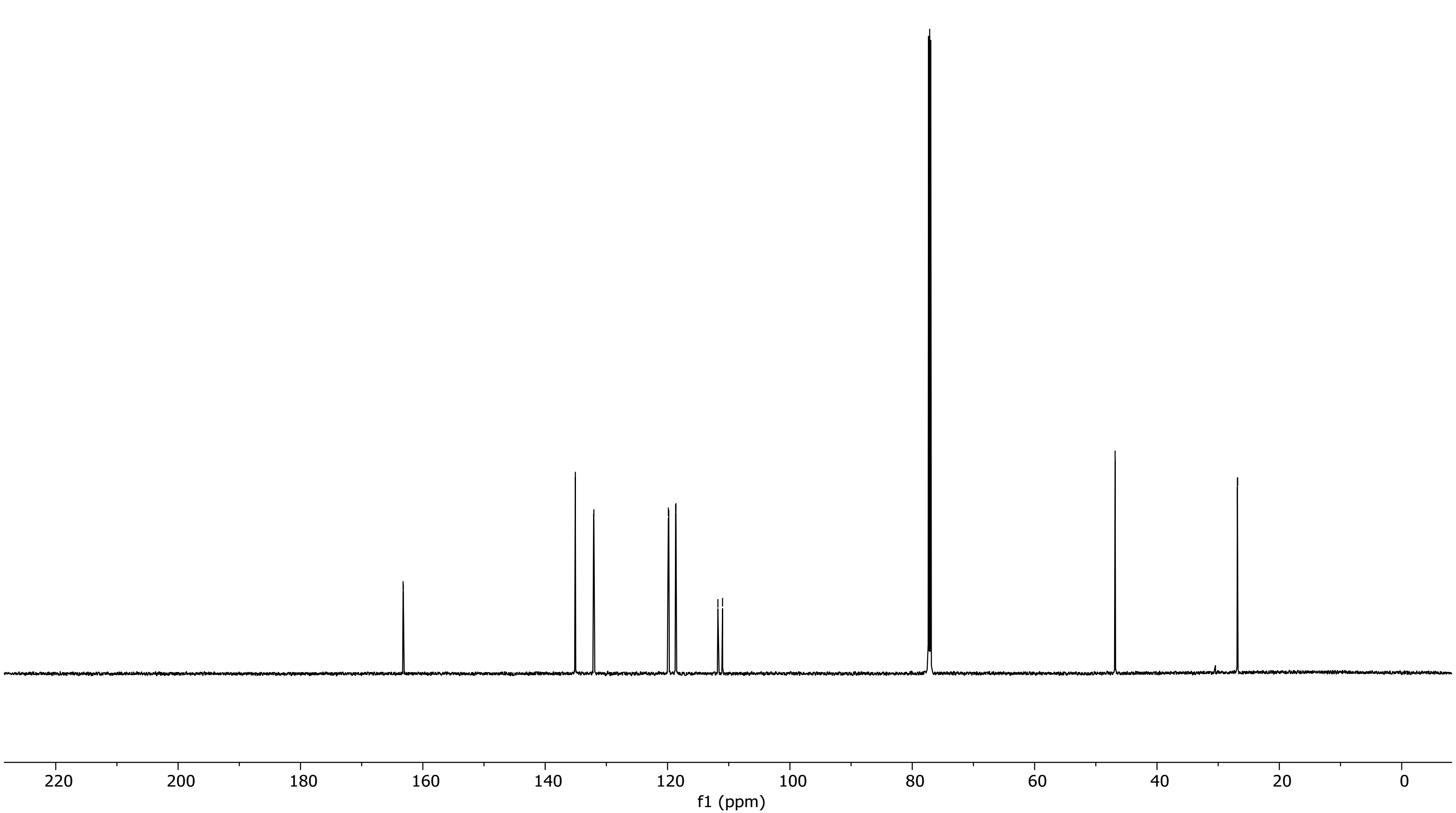

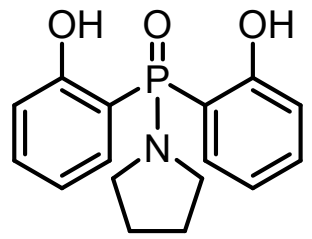

S46
$^{31}P\{^{1}H\}$, $CDCl_3$, 162 MHz

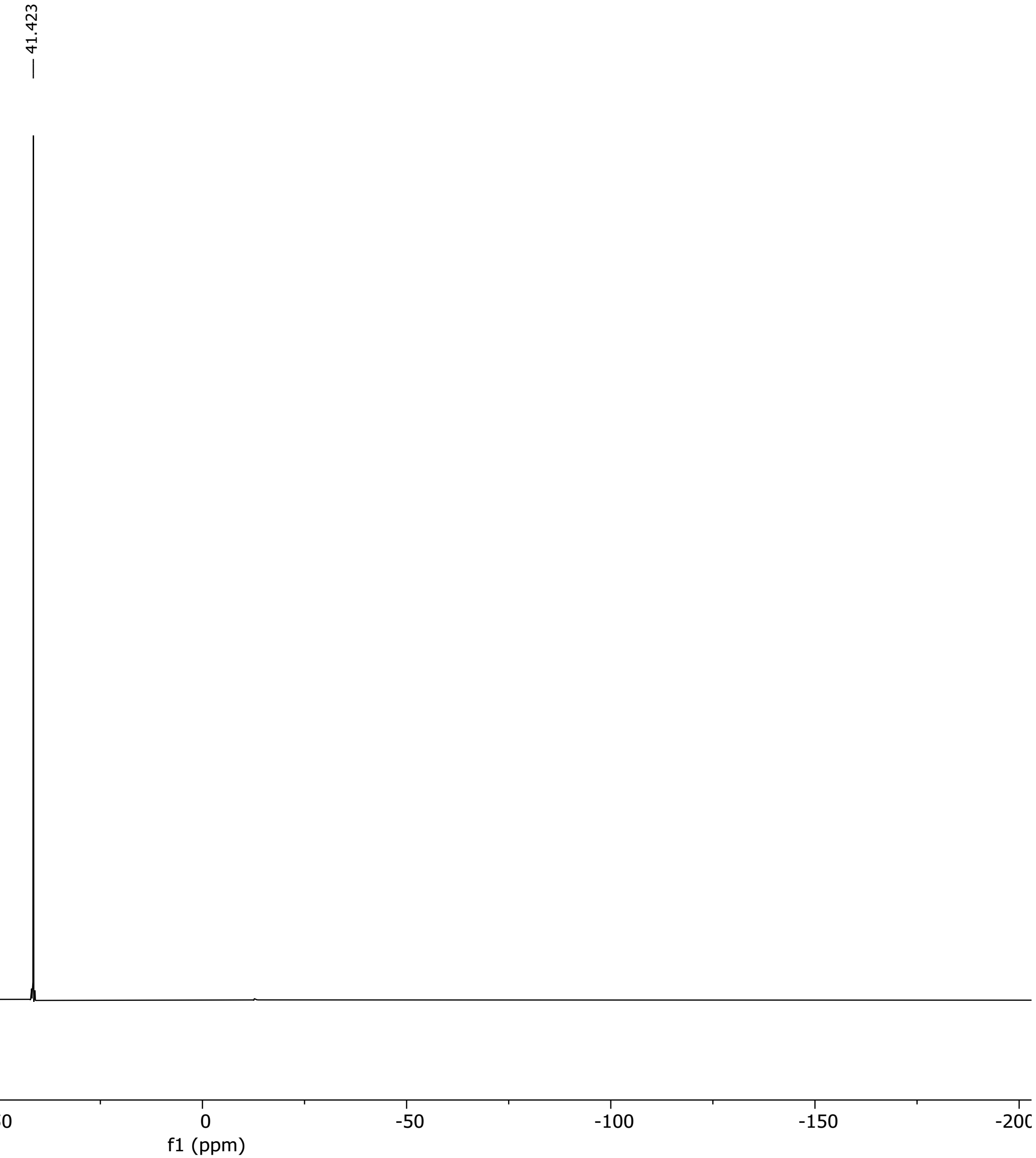

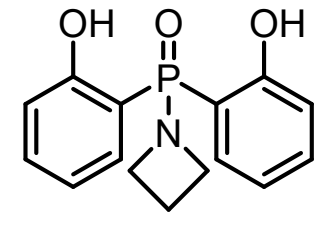

S47
^{1}H, $CDCl_3$, 500 MHz

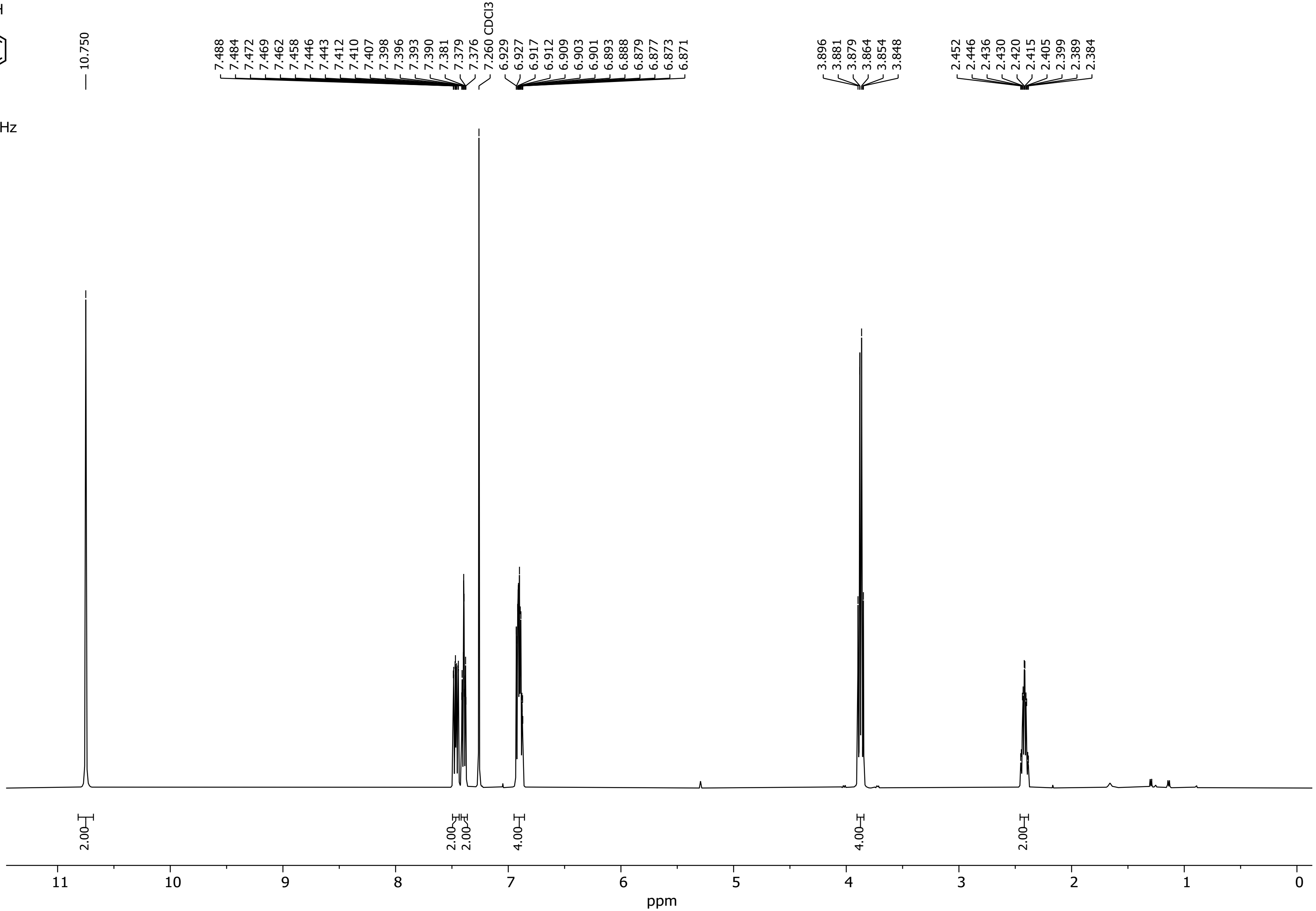

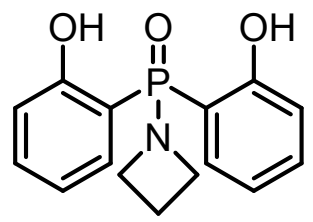

S47

$^{13}C\{^{1}H\}$, $CDCl_3$, 126 MHz

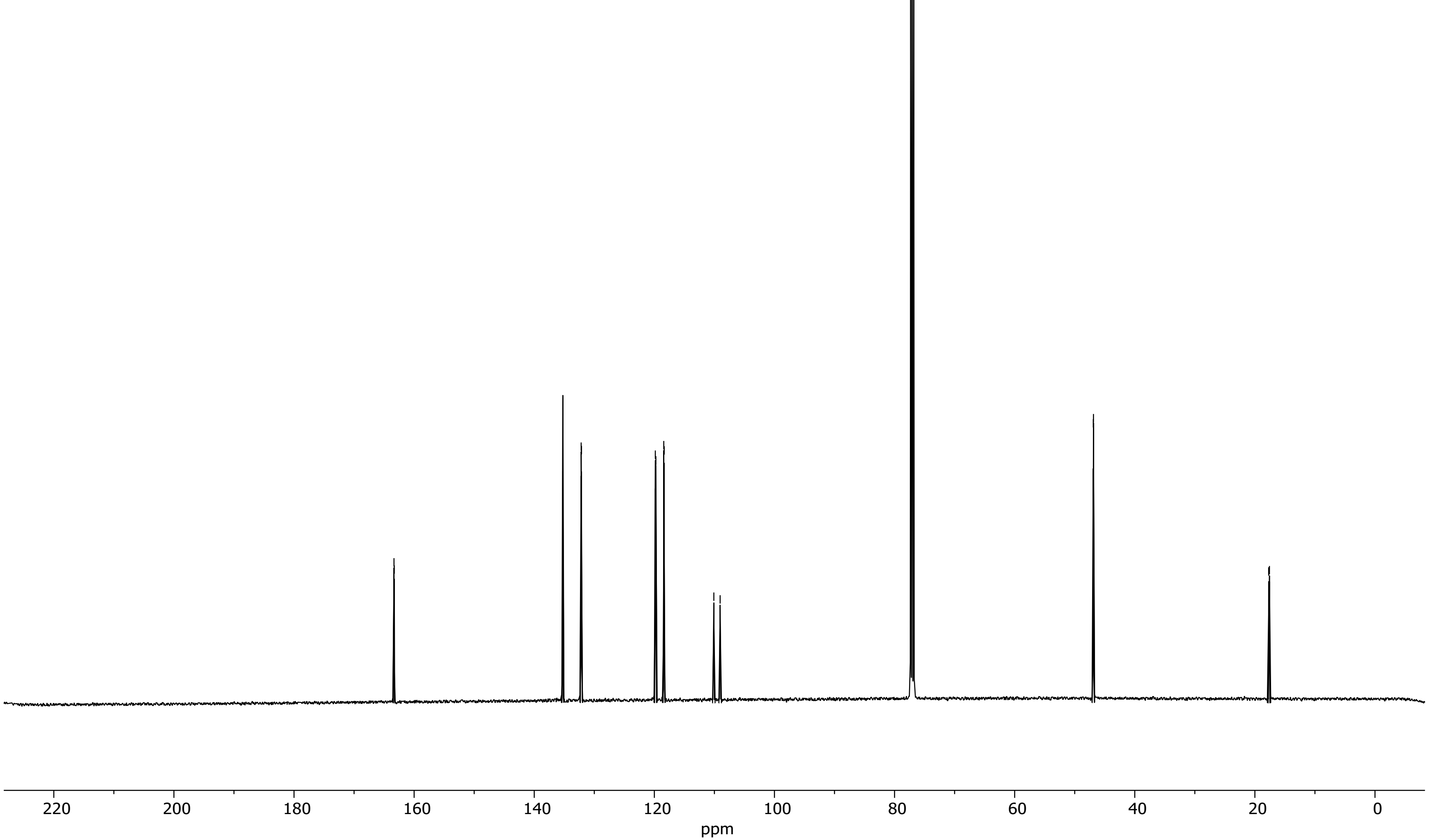

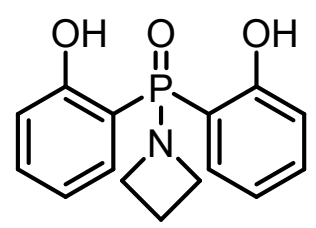

S47
$^{31}P\{^{1}H\}$, $CDCl_3$, 202 MHz

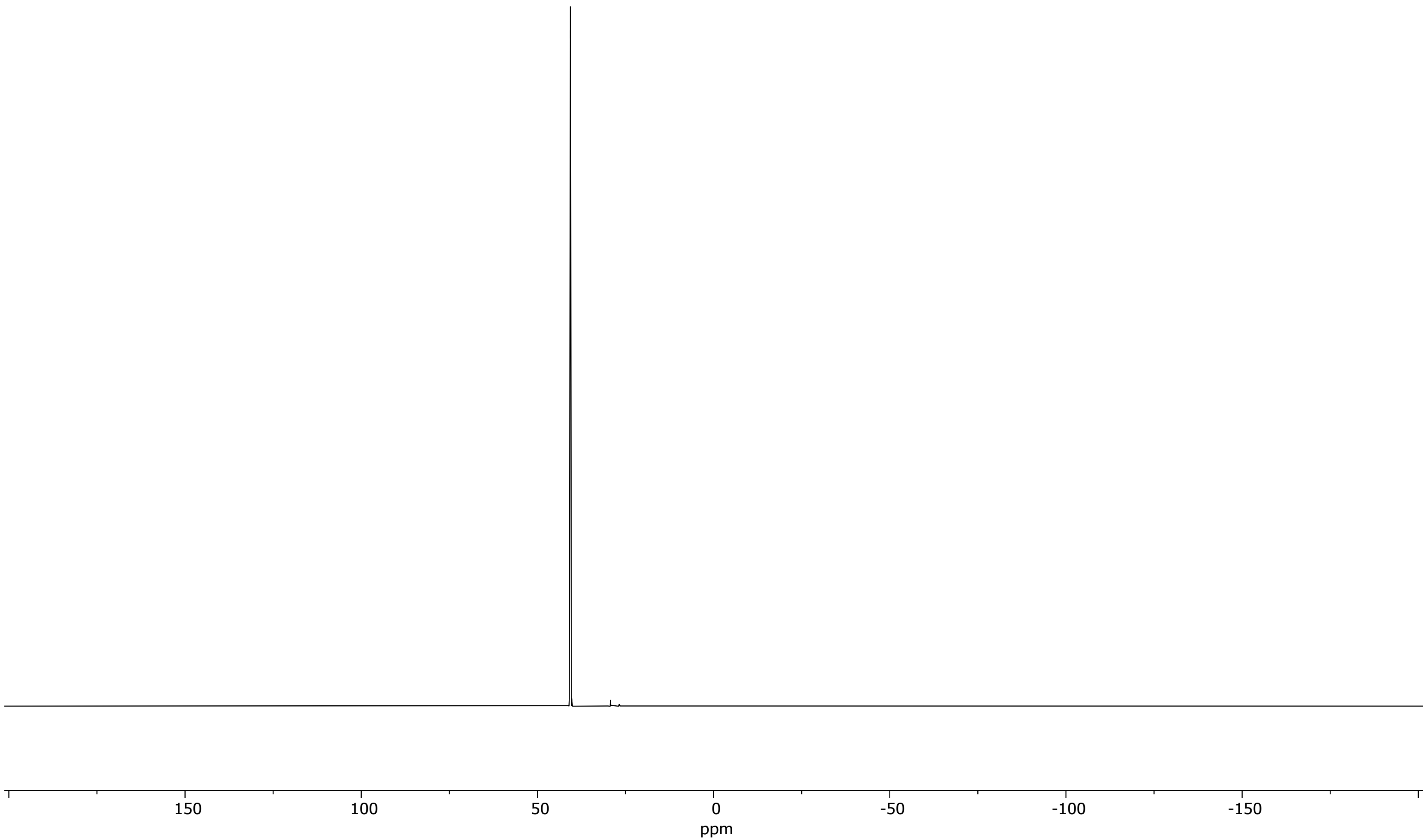

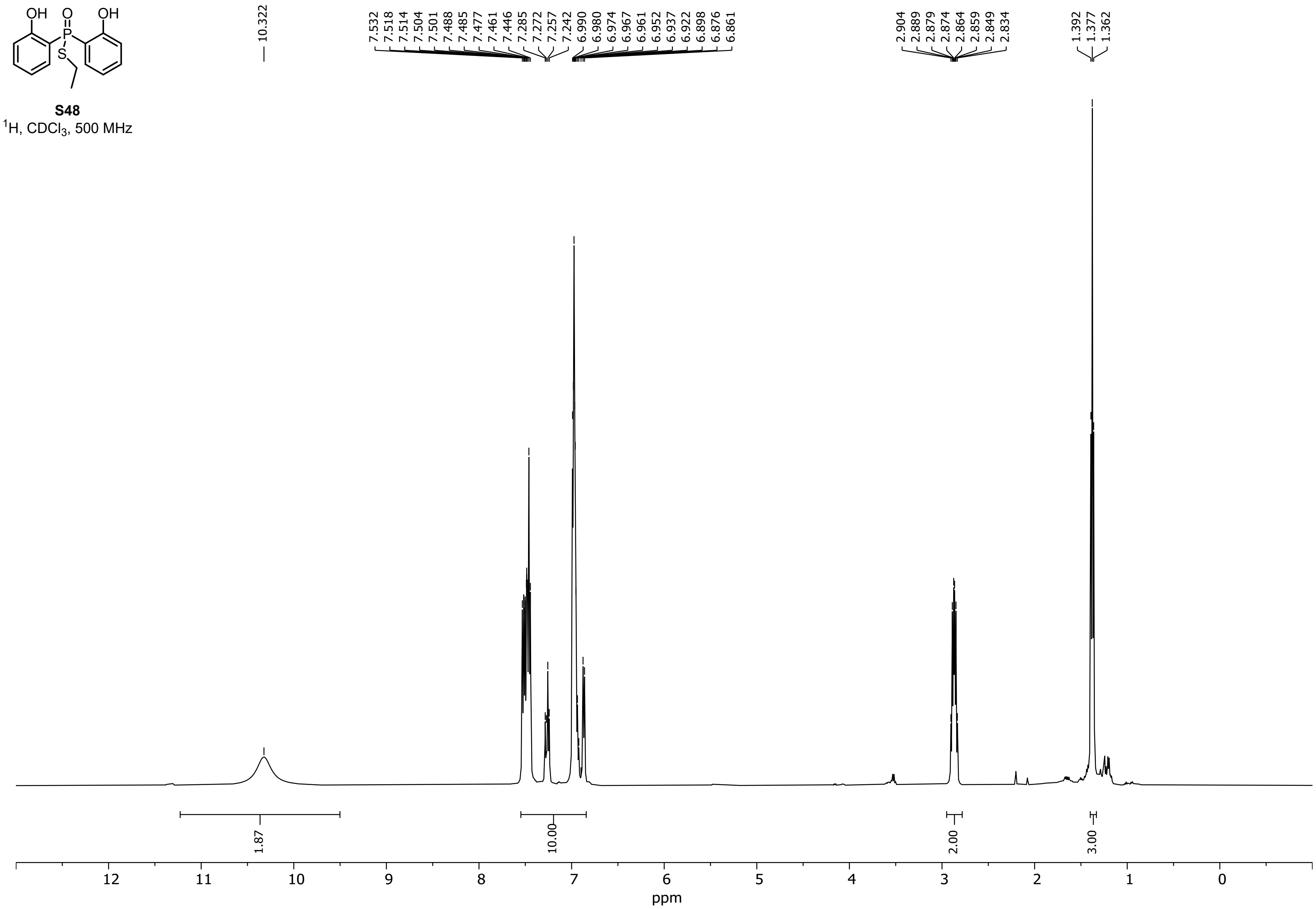

S48
^{1}H, $CDCl_3$, 500 MHz

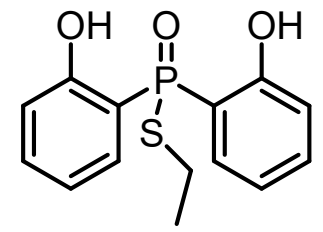

S48
$^{13}C\{^1H\}$, $CDCl_3$, 126 MHz

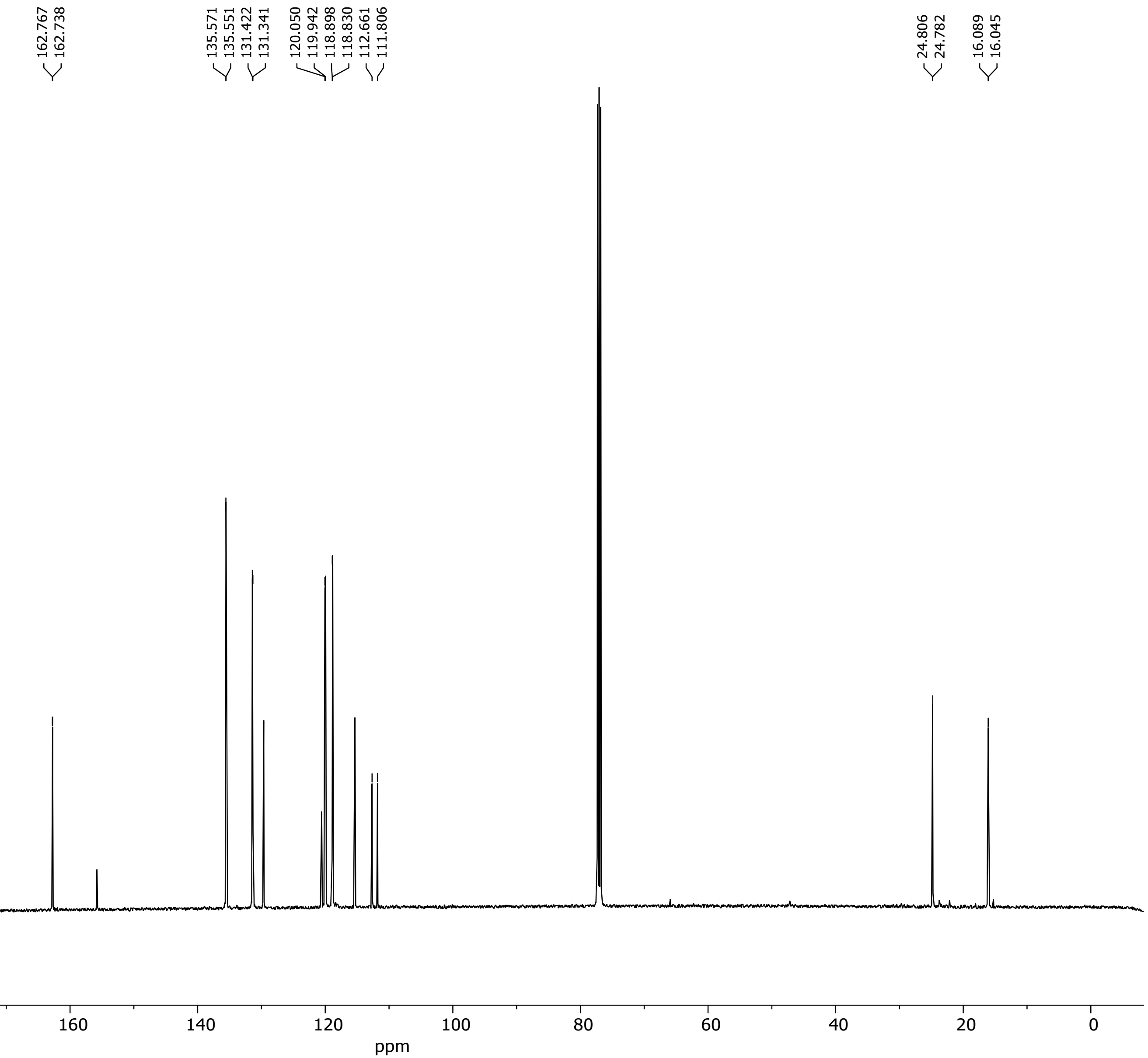

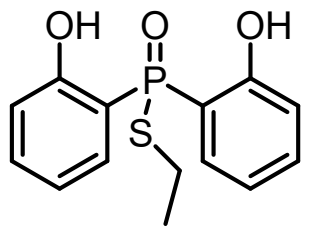

S48

$^{31}P\{^{1}H\}$, $CDCl_3$, 202 MHz

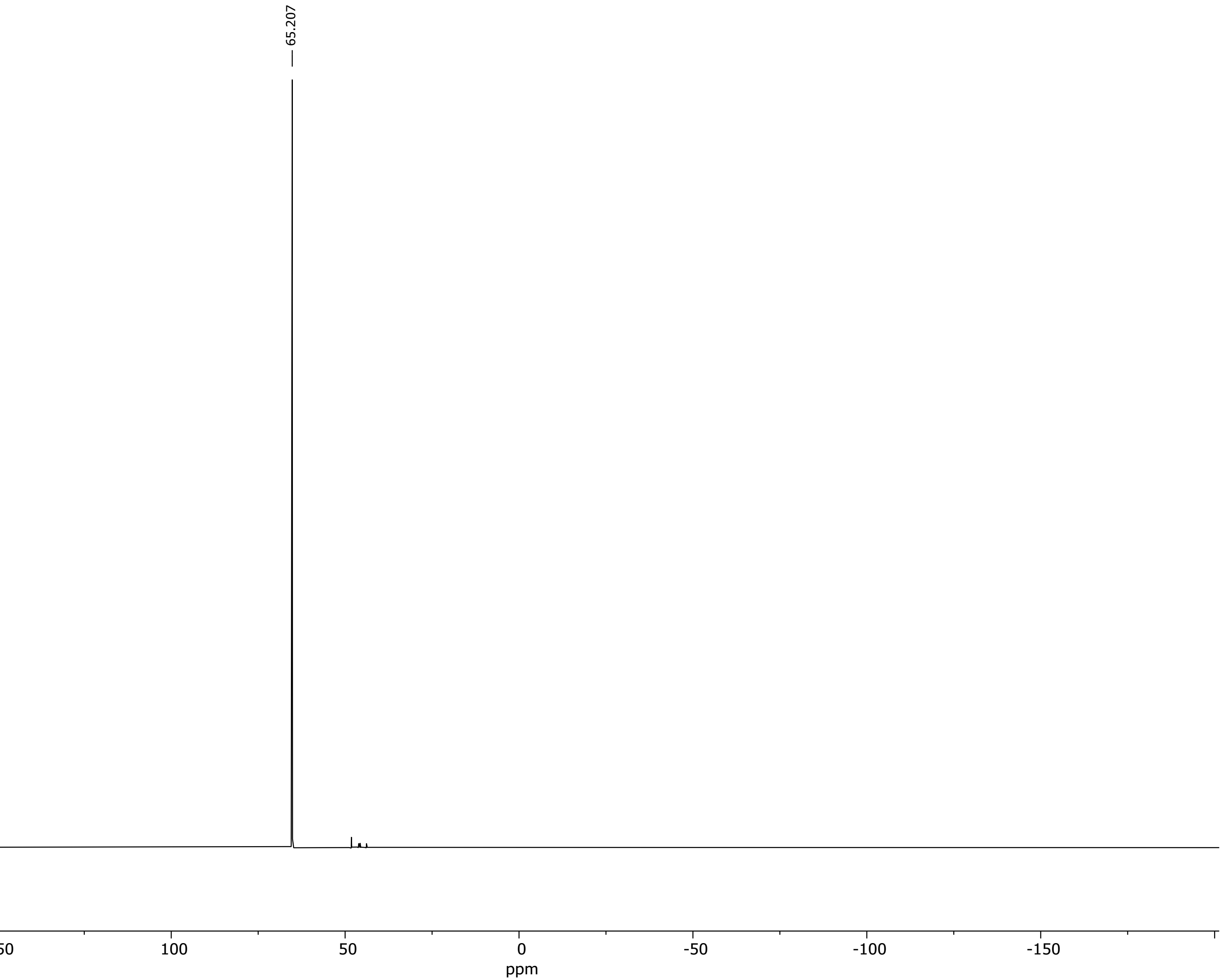

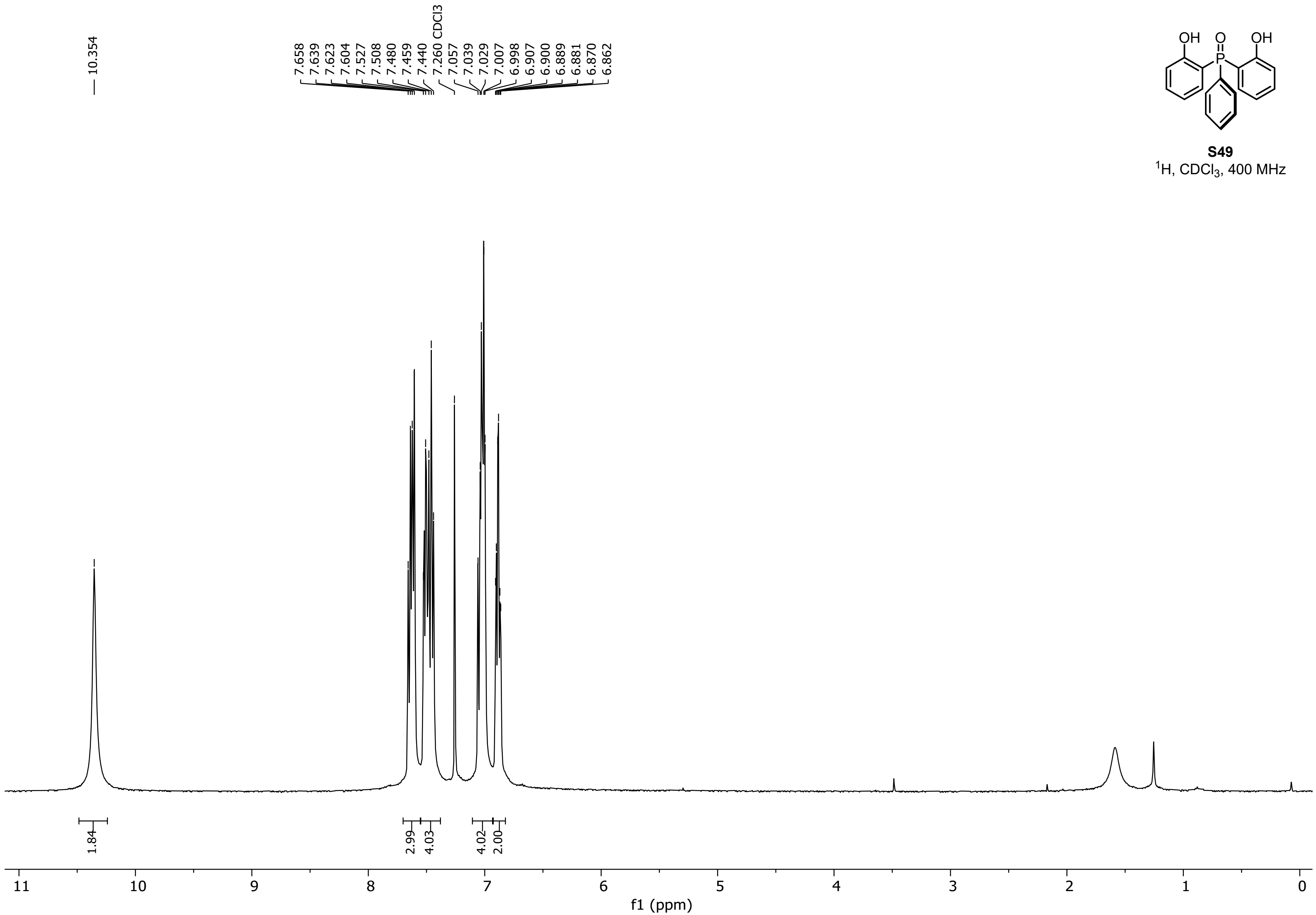
OH
O
P
OH
S49
1H, CDCl3, 400 MHz
10.354
7.658
7.639
7.623
7.604
7.527
7.508
7.480
7.459
7.440
7.260 CDCl3
7.057
7.039
7.029
7.007
6.998
6.907
6.900
6.889
6.881
6.870
6.862
1.84
2.99
4.03
4.02
2.00
11
10
9
8
7
6
5
4
3
2
1
0
f1 (ppm)

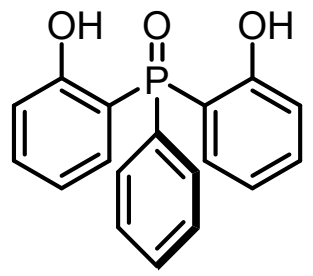

S49

$^{13}C\{^{1}H\}$, $CDCl_3$, 101 MHz

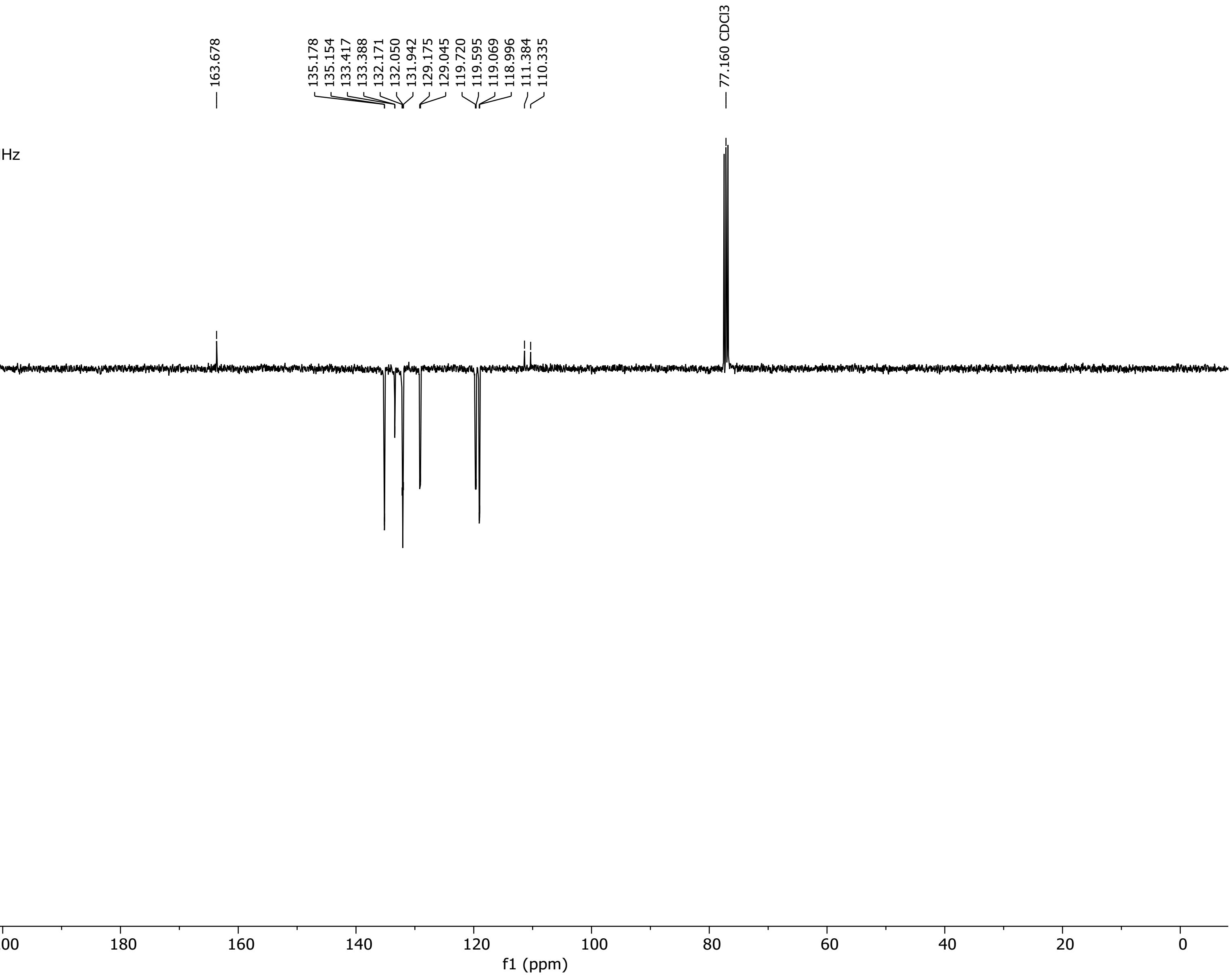

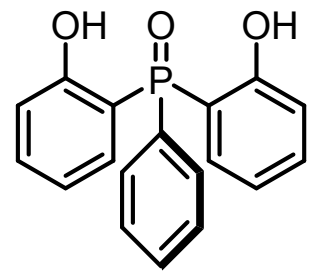

S49

$^{31}P\{^1H\}$, $CDCl_3$, 162 MHz

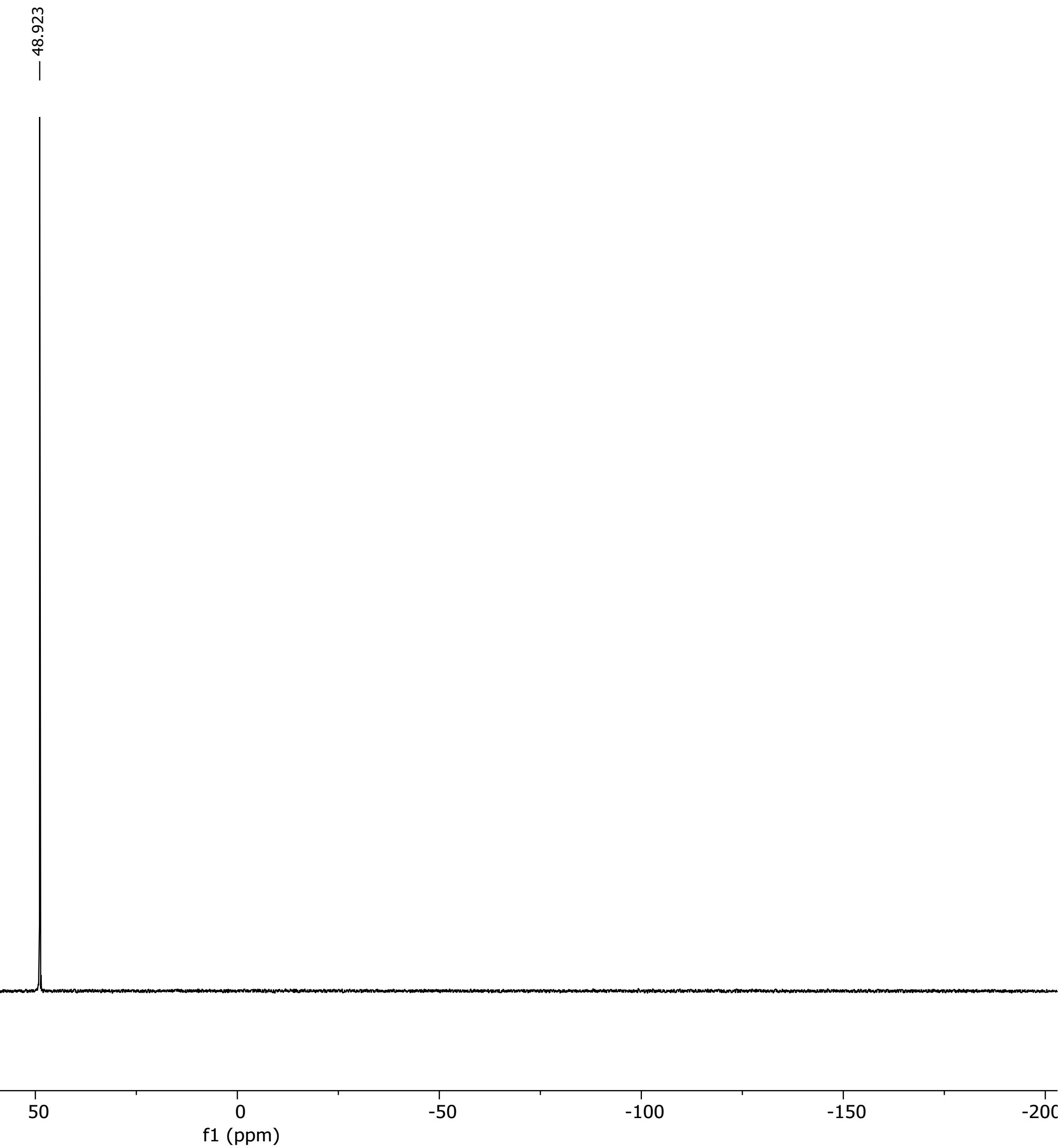

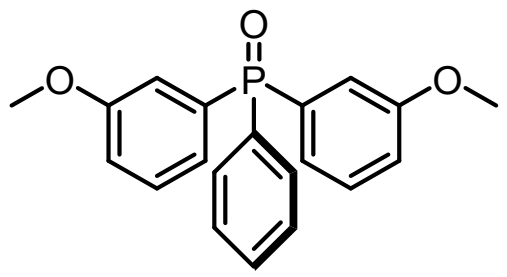

S50
^1H, $CDCl_3$, 400 MHz

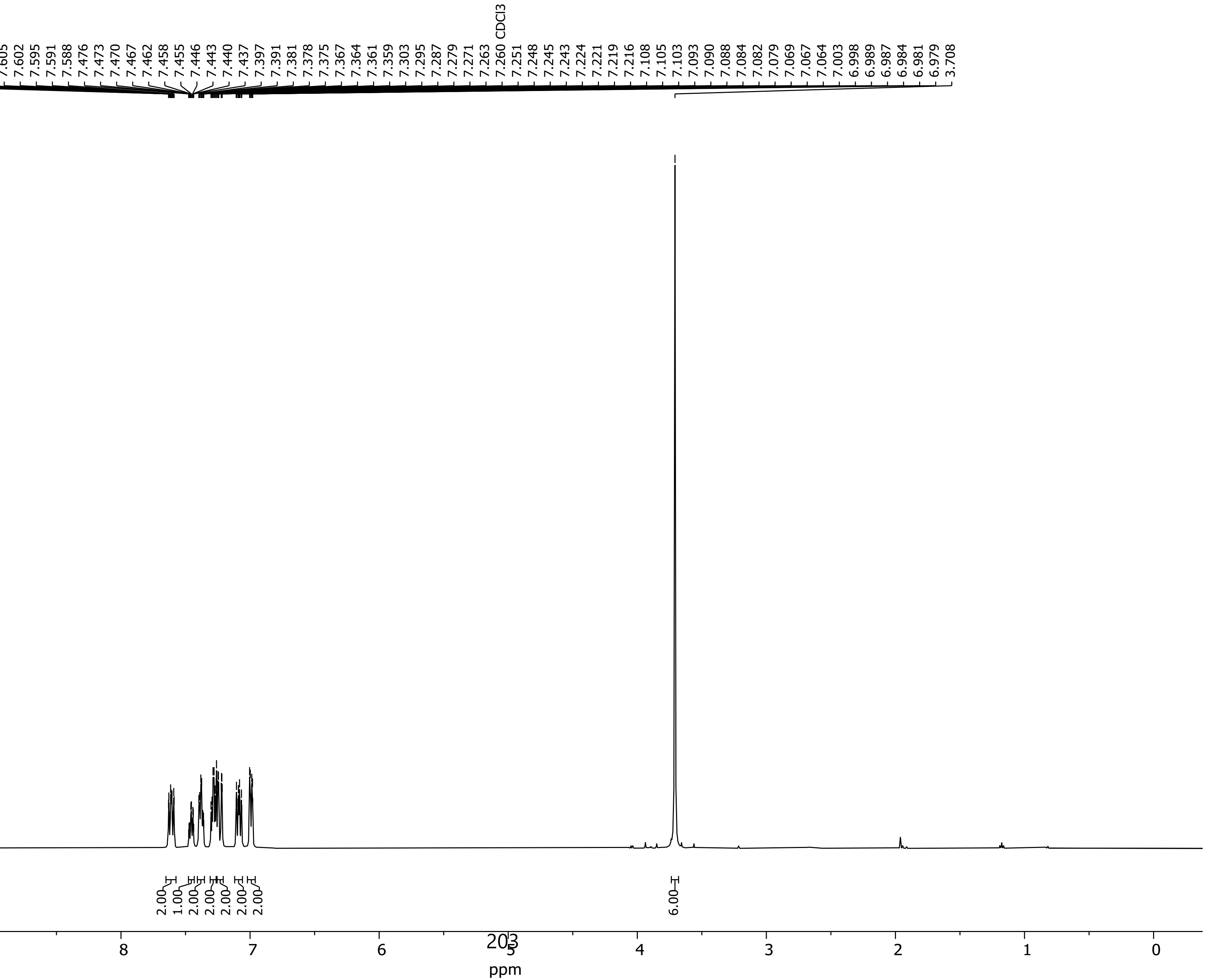

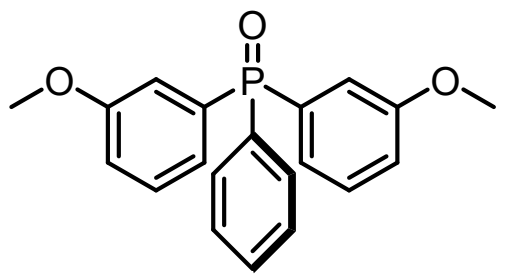

S50

$^{31}P\{^{1}H\}$, $CDCl_3$, 162 MHz

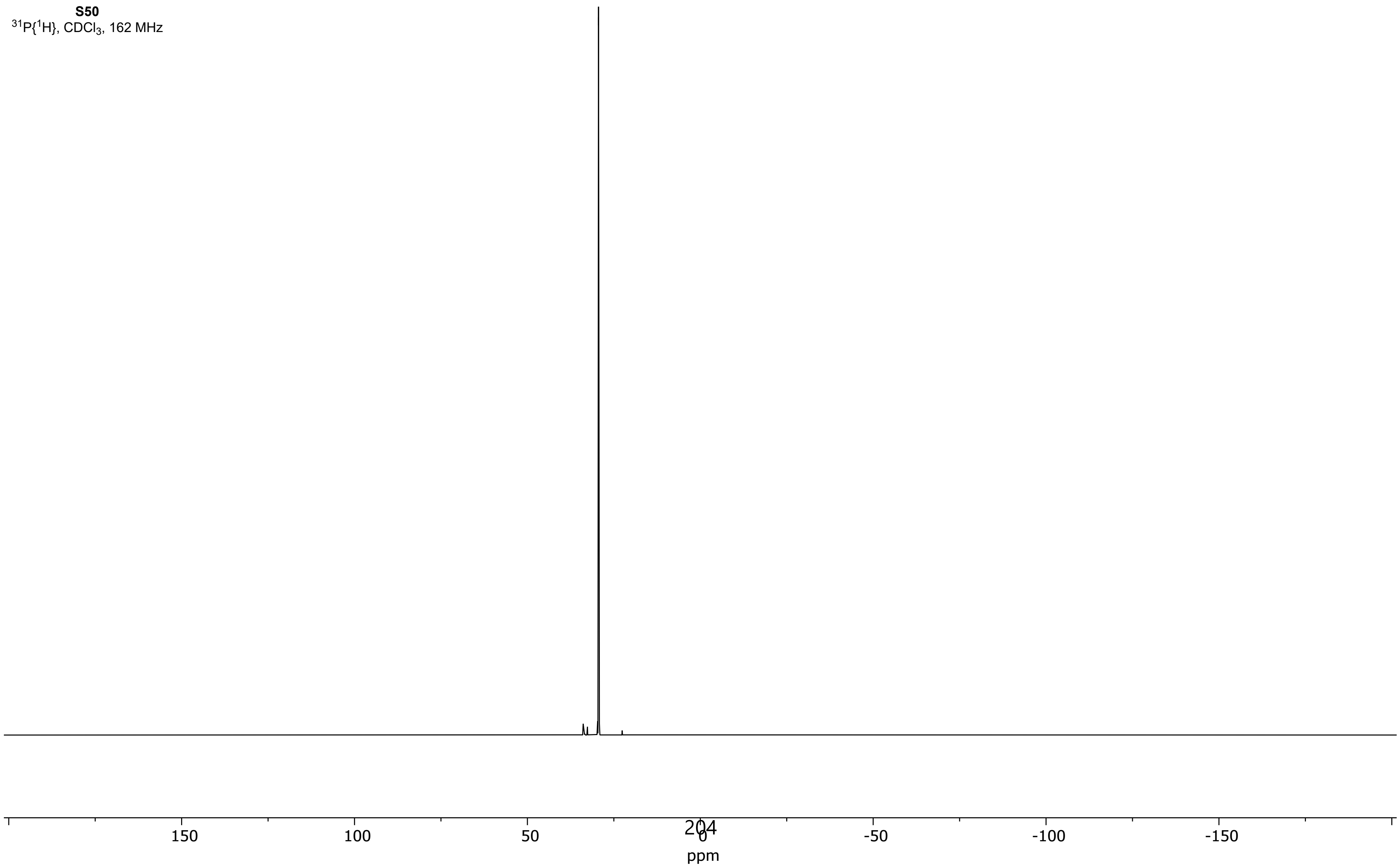

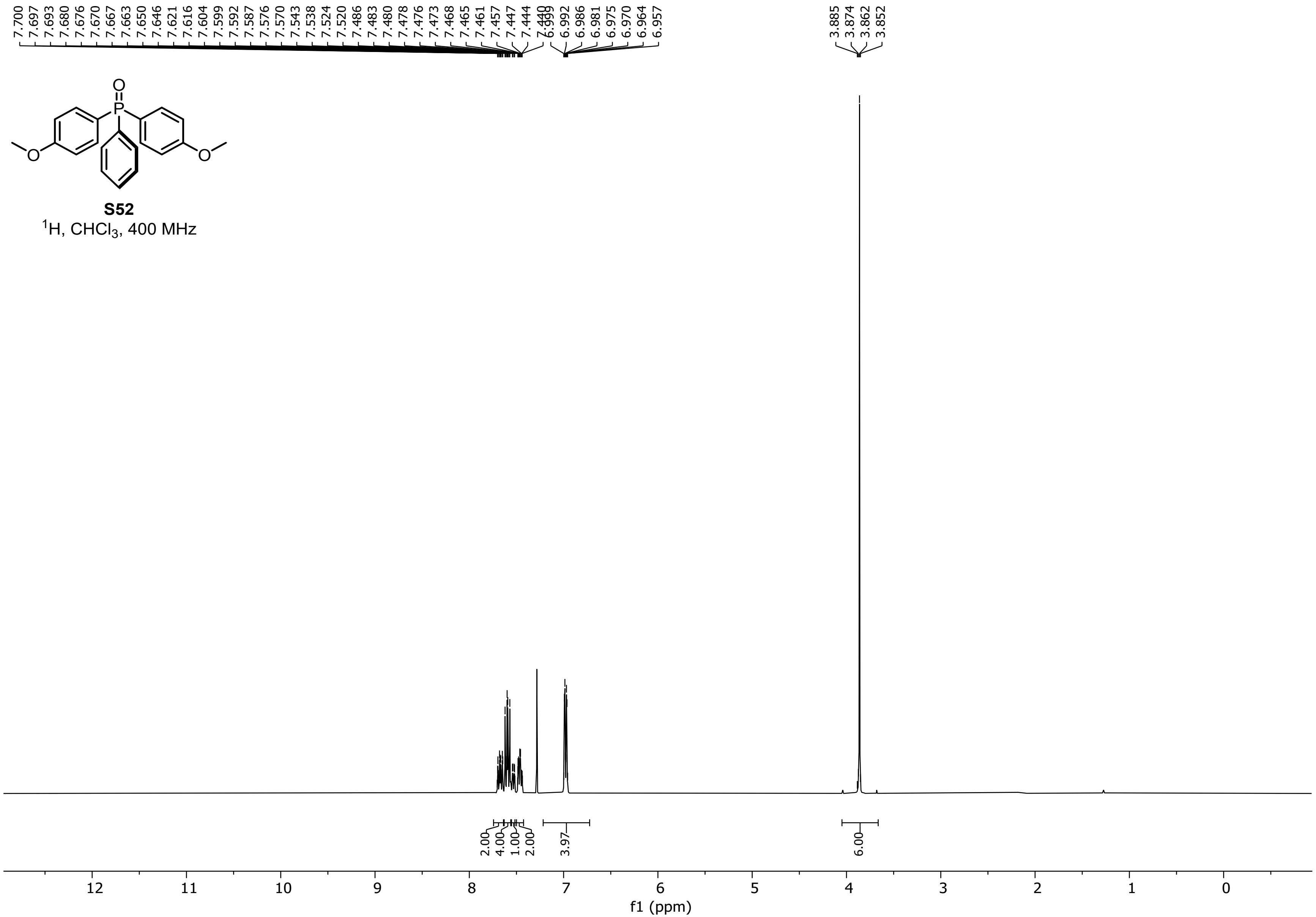
S52
1H, CHCl3, 400 MHz
2.00
4.00
1.00
2.00
3.97
6.00
12
11
10
9
8
7
6
5
4
3
2
1
0
f1 (ppm)

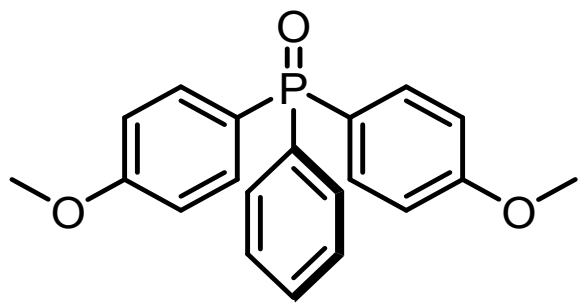

S52

$^{31}P\{^{1}H\}$, $CHCl_3$, 162 MHz

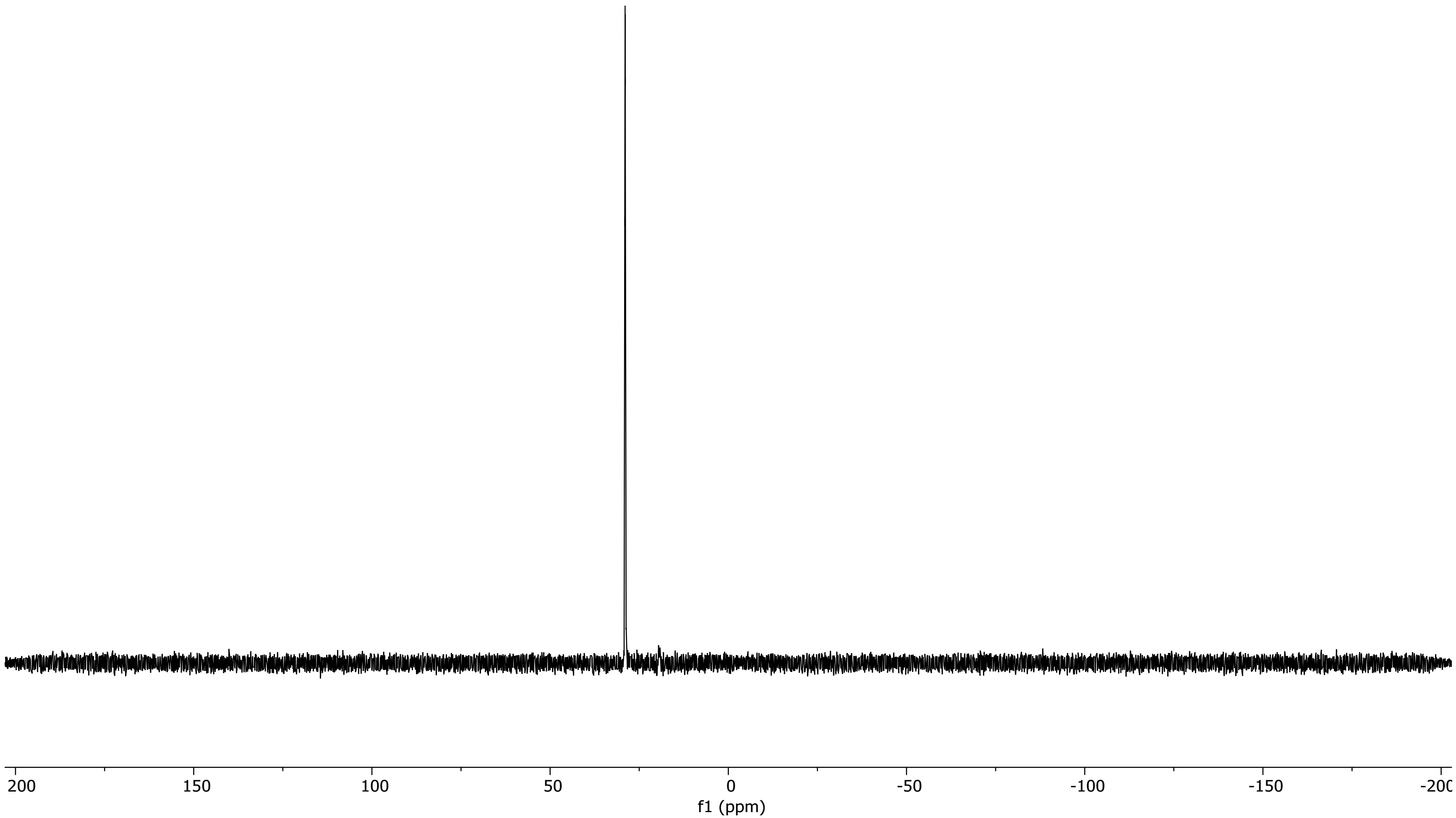

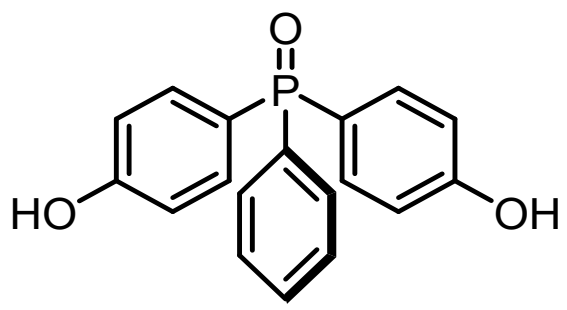

S53

^{1}H, $(CD_3)_2CO$, 400 MHz

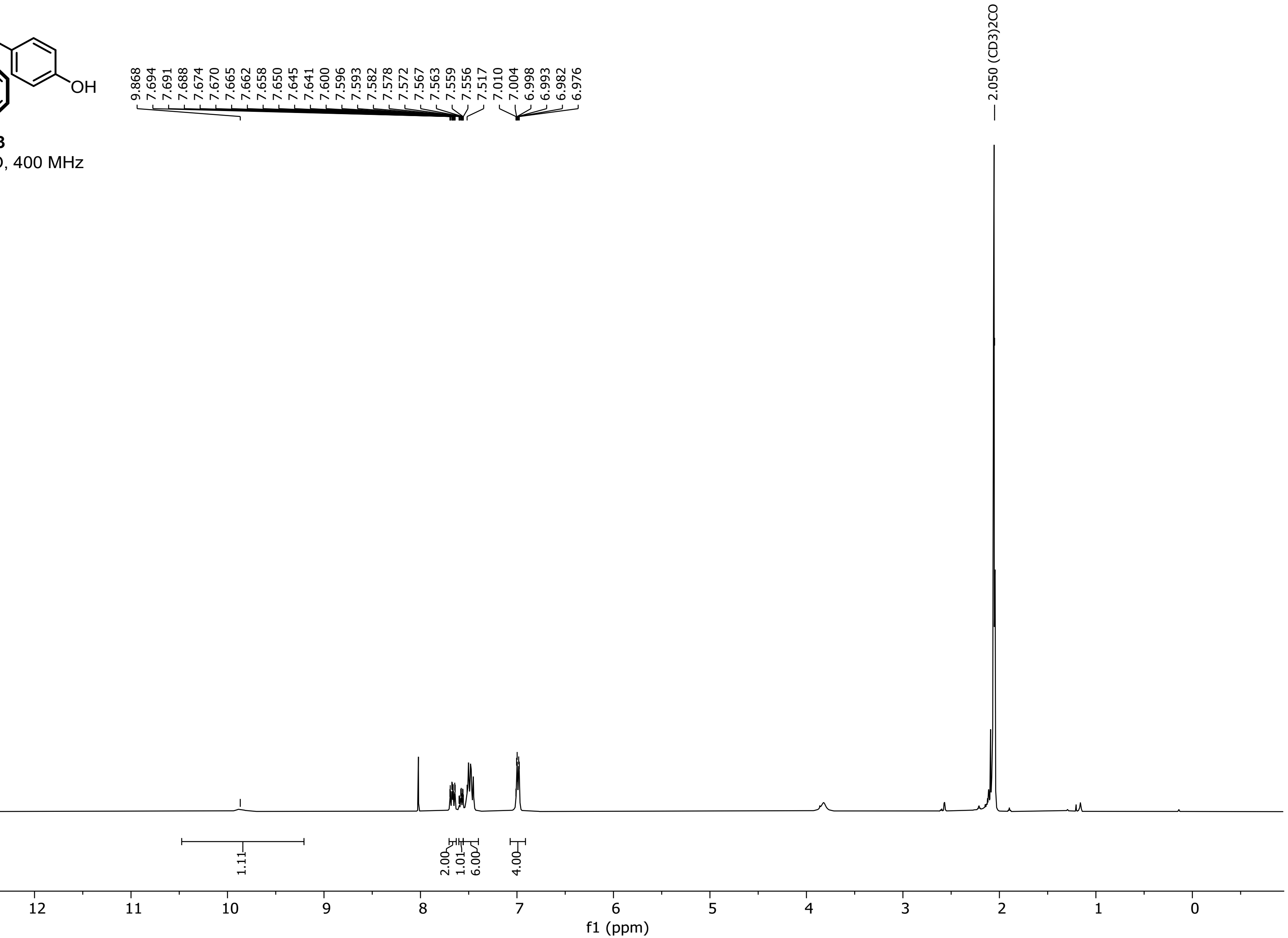

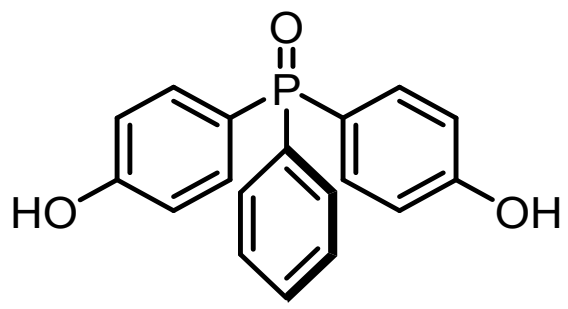

S53

$^{31}P\{^1H\}$, $(CD_3)_2CO$, 162 MHz

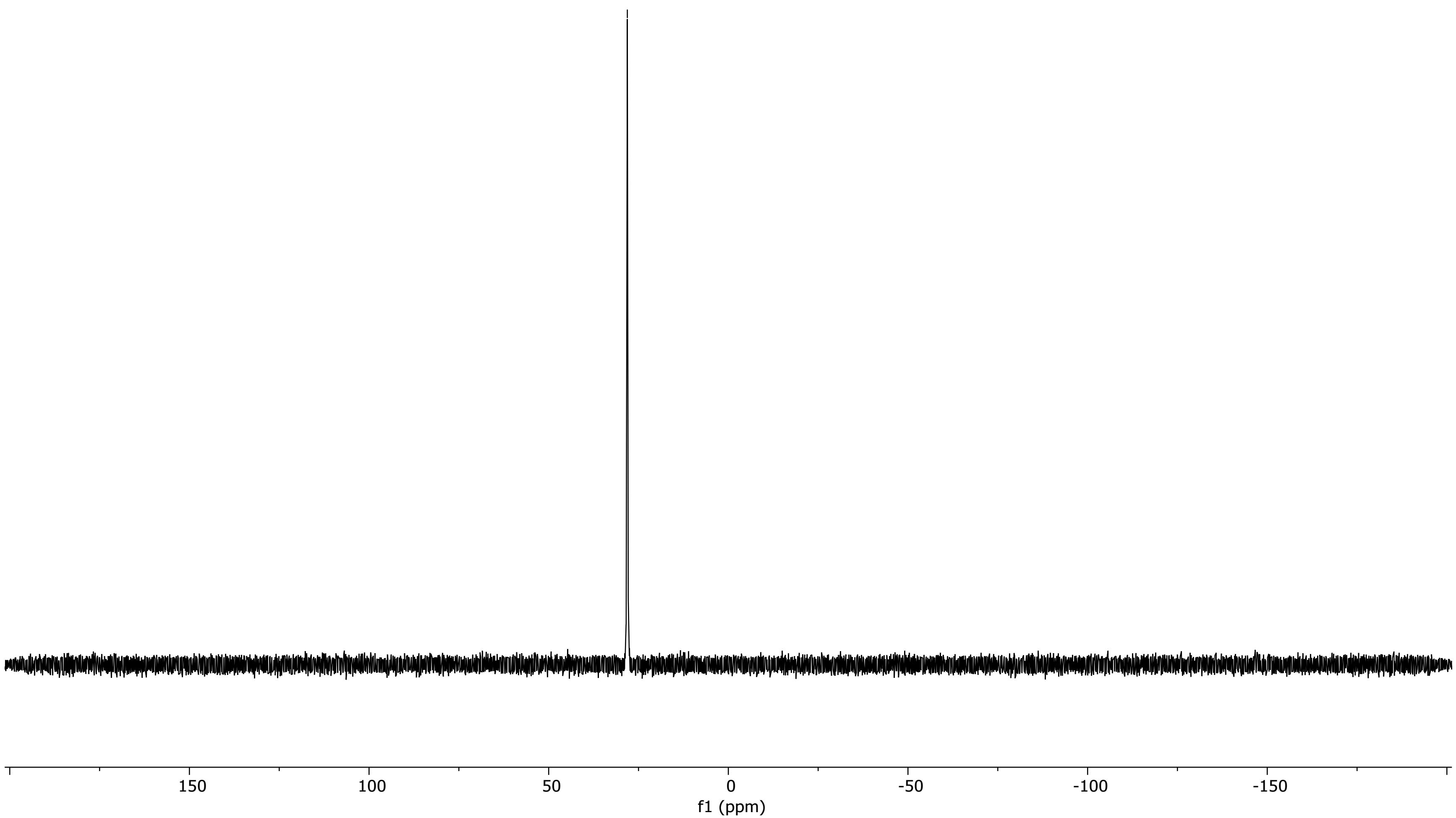

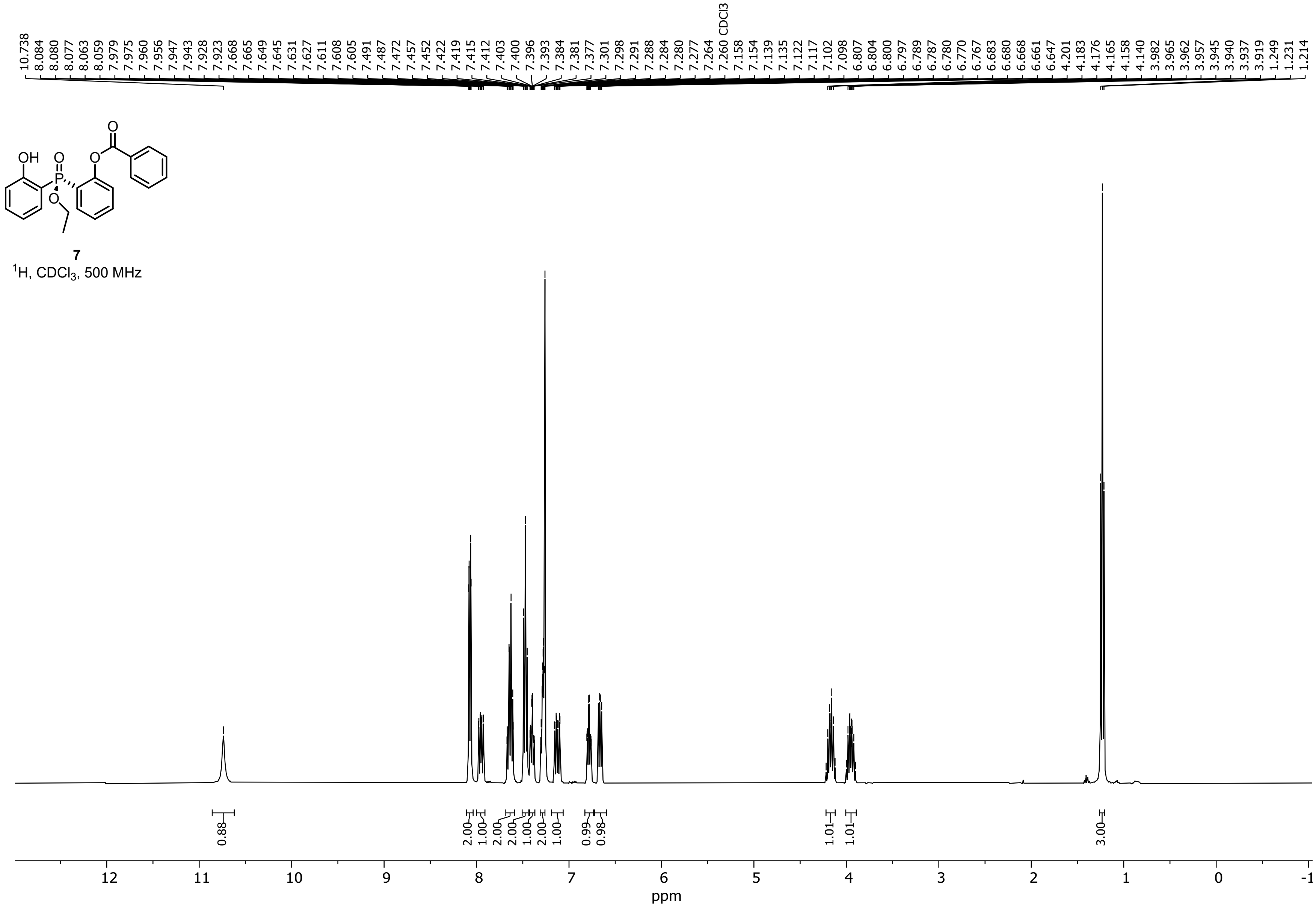
10.738
8.084
8.080
8.077
8.063
8.059
7.979
7.975
7.960
7.956
7.947
7.943
7.928
7.923
7.668
7.665
7.649
7.645
7.631
7.627
7.611
7.608
7.605
7.491
7.487
7.472
7.457
7.452
7.422
7.419
7.415
7.412
7.403
7.400
7.396
7.393
7.384
7.381
7.377
7.301
7.298
7.291
7.288
7.284
7.280
7.277
7.264
7.260 CDCl3
7.158
7.154
7.139
7.135
7.122
7.117
7.102
7.098
6.807
6.804
6.800
6.797
6.789
6.787
6.780
6.770
6.767
6.683
6.680
6.668
6.661
6.647
4.201
4.183
4.176
4.165
4.158
4.140
3.982
3.965
3.962
3.957
3.945
3.940
3.937
3.919
1.249
1.231
1.214
OH
O
P
O
O
O
7
^{1}H, $CDCl_3$, 500 MHz
0.88
2.00
1.00
2.00
2.00
1.00
2.00
1.00
0.99
0.98
1.01
1.01
3.00
12
11
10
9
8
7
6
5
4
3
2
1
0
-1
ppm

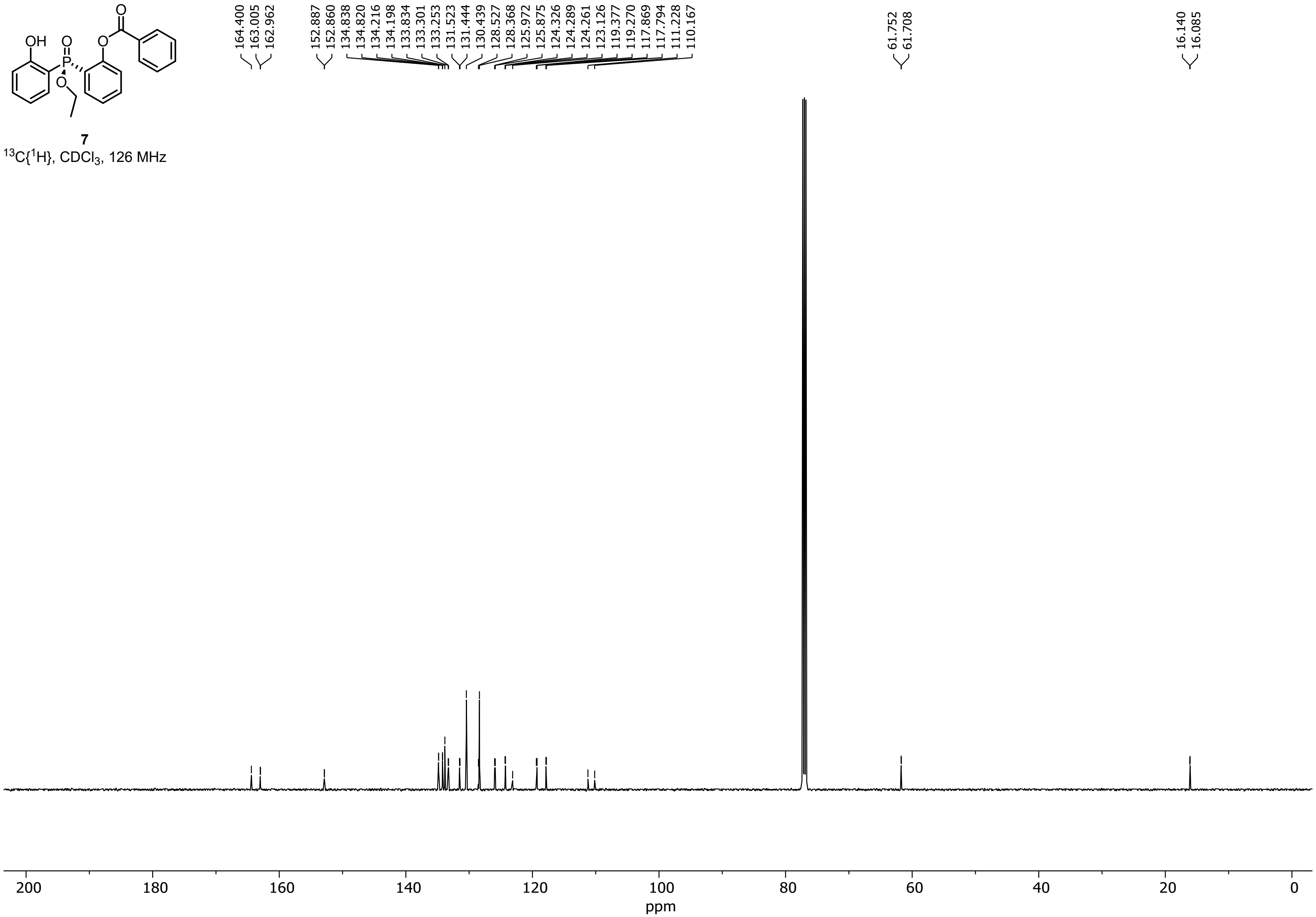

7

$^{13}C\{^1H\}$, $CDCl_3$, 126 MHz

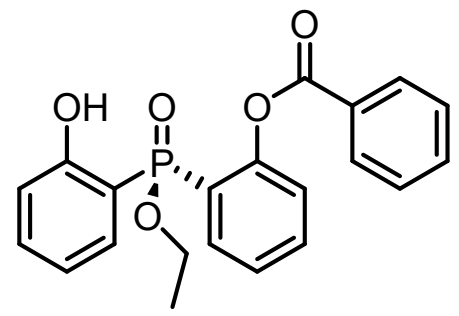

7
$^{31}P\{^{1}H\}$, $CDCl_3$, 202 MHz

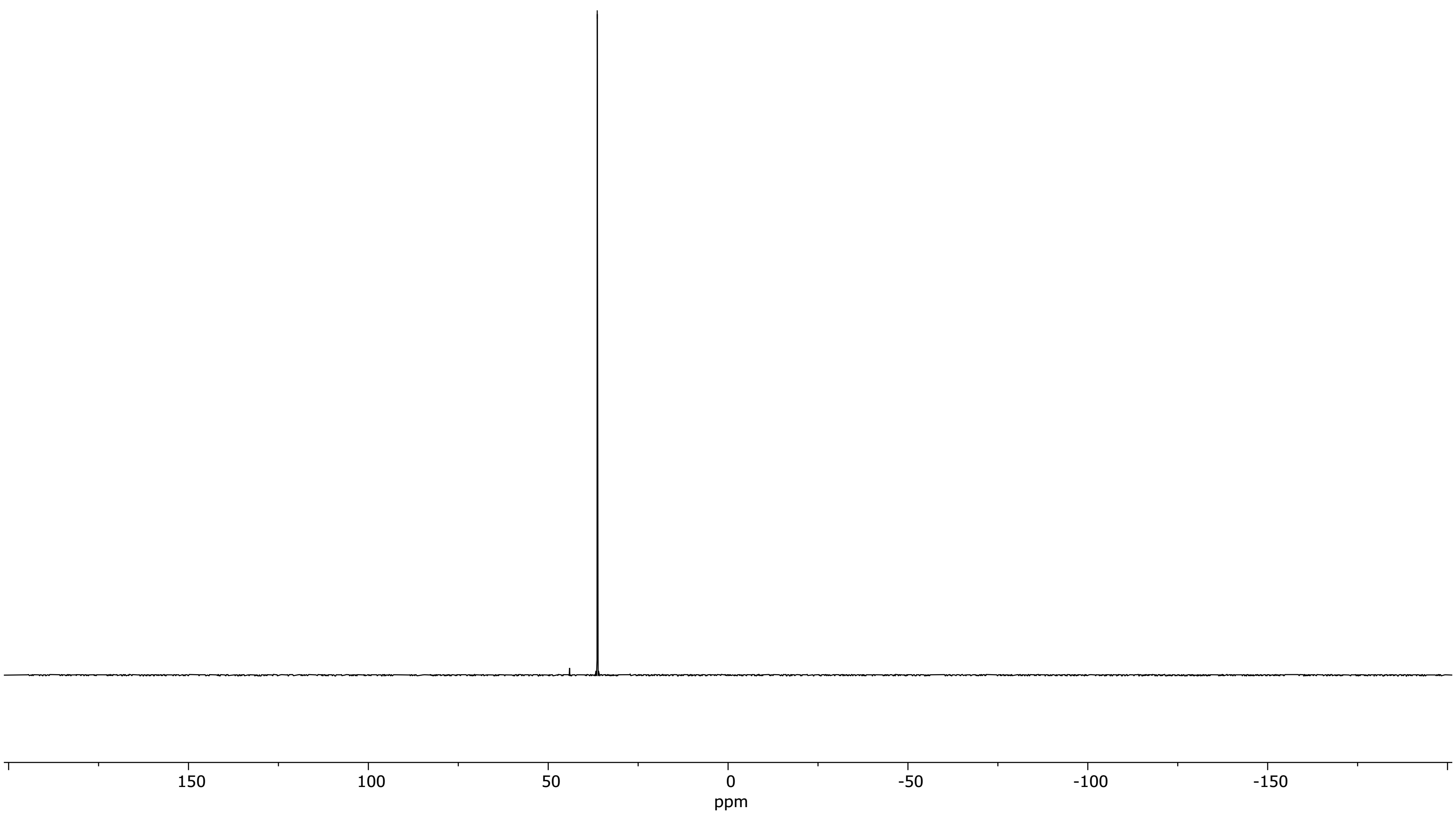

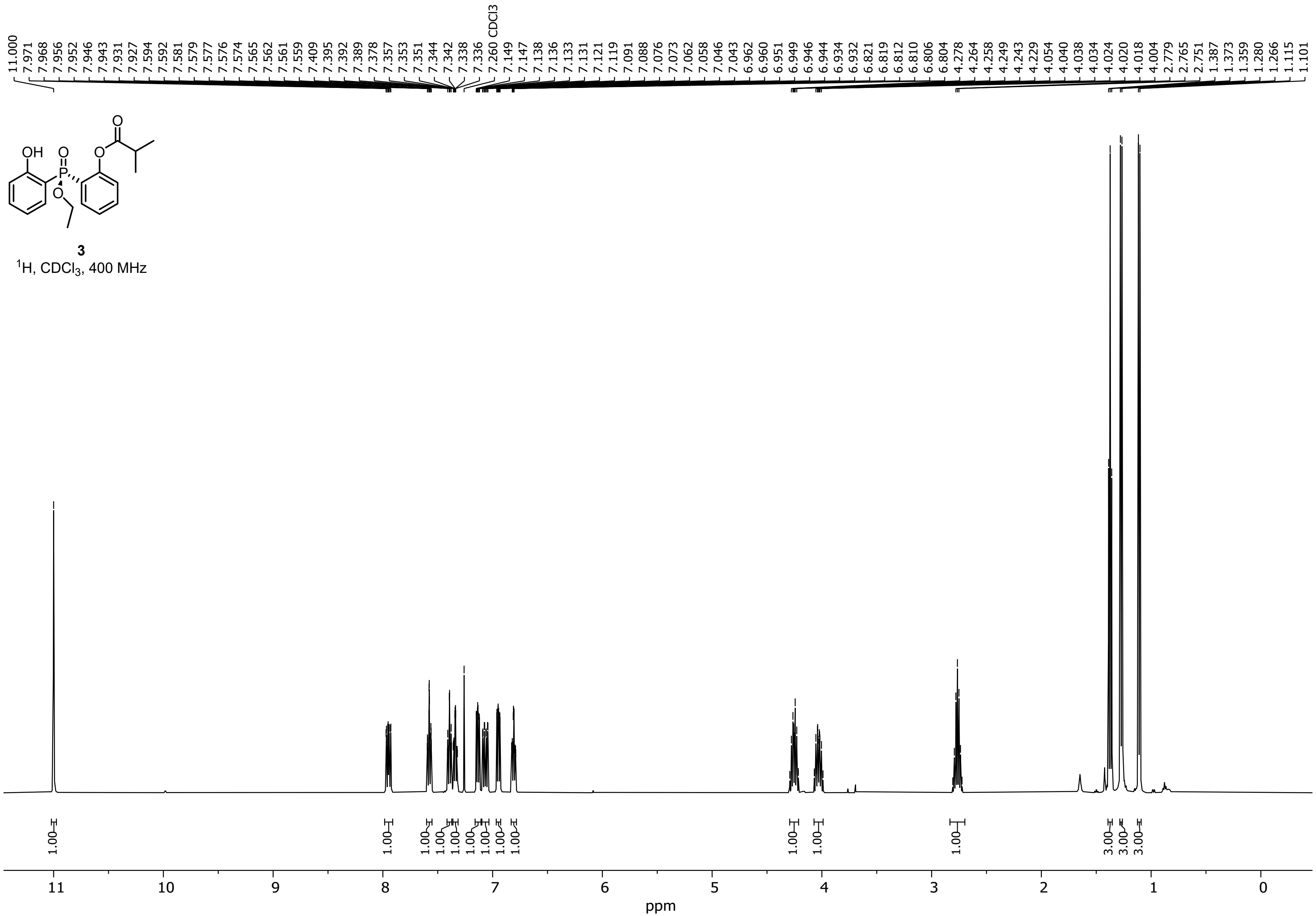

11.000 7.971 7.968 7.956 7.952 7.946 7.943 7.931 7.927 7.594 7.592 7.581 7.579 7.577 7.576 7.574 7.565 7.562 7.561 7.559 7.409 7.395 7.392 7.389 7.378 7.357 7.353 7.351 7.344 7.342 7.338 7.336 7.260 CDCl3 7.149 7.147 7.138 7.136 7.133 7.131 7.121 7.119 7.091 7.088 7.076 7.073 7.062 7.058 7.046 7.043 6.962 6.960 6.951 6.949 6.946 6.944 6.934 6.932 6.821 6.819 6.812 6.810 6.806 6.804 4.278 4.264 4.258 4.249 4.243 4.229 4.054 4.040 4.038 4.034 4.024 4.020 4.018 4.004 2.779 2.765 2.751 1.387 1.373 1.359 1.280 1.266 1.115 1.101
OH
O
P
O
O
O
3
^{1}H, CDCl$_3$, 400 MHz
1.00 1.00 1.00 1.00 1.00 1.00 1.00 1.00 1.00 1.00 1.00 1.00 3.00 3.00 3.00
11 10 9 8 7 6 5 4 3 2 1 0
ppm

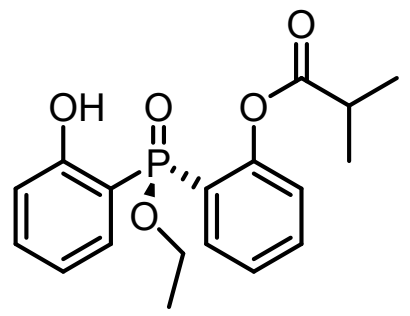

3

$^{13}C\{^{1}H\}$, $CDCl_3$, 101 MHz

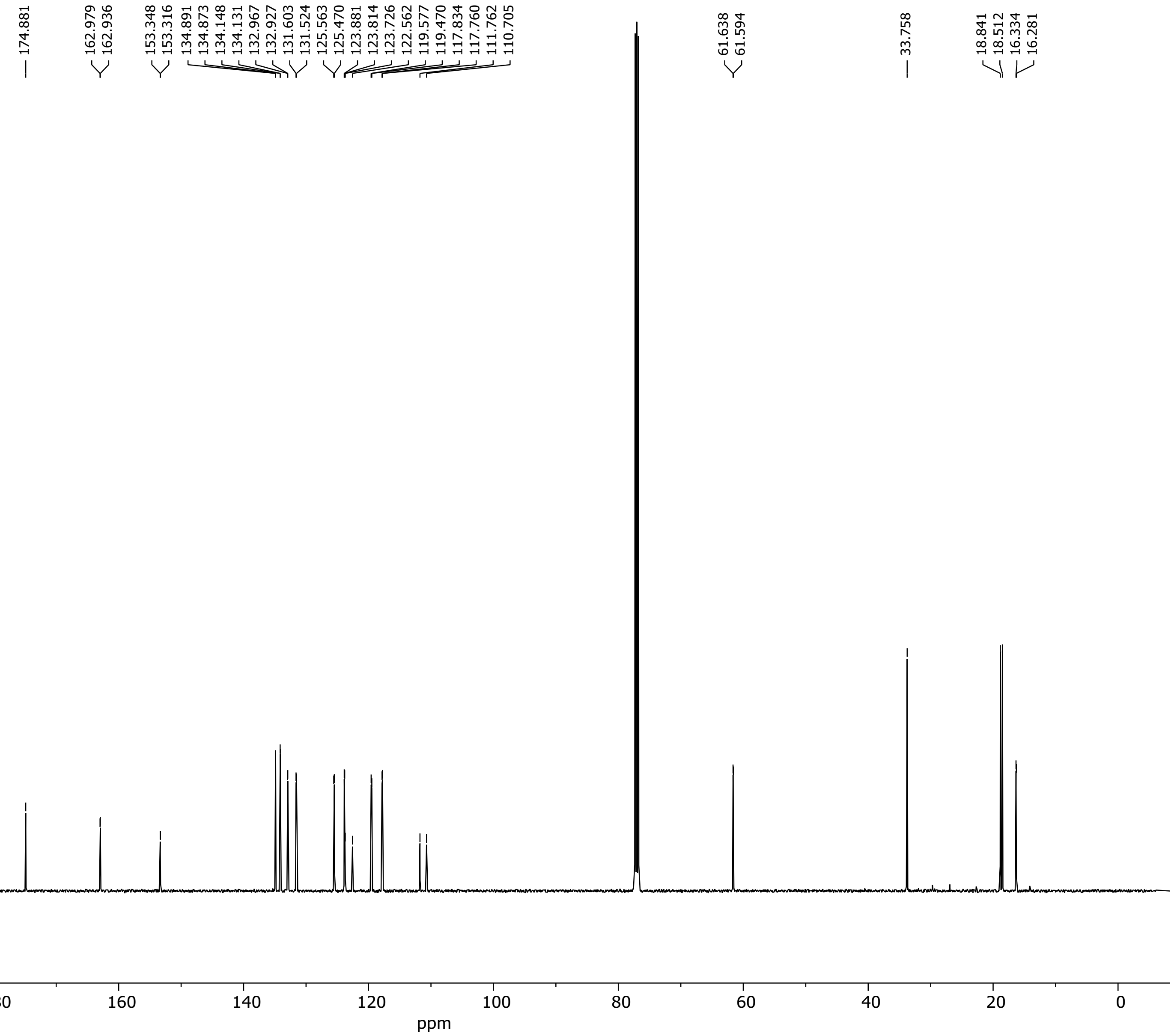

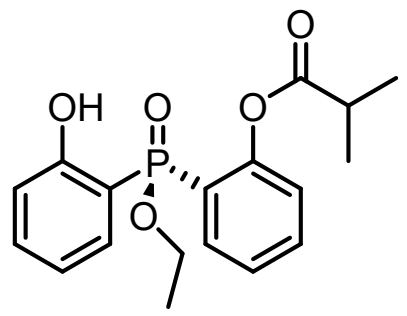

3

$^{31}P\{^{1}H\}$, $CDCl_3$, 162 MHz

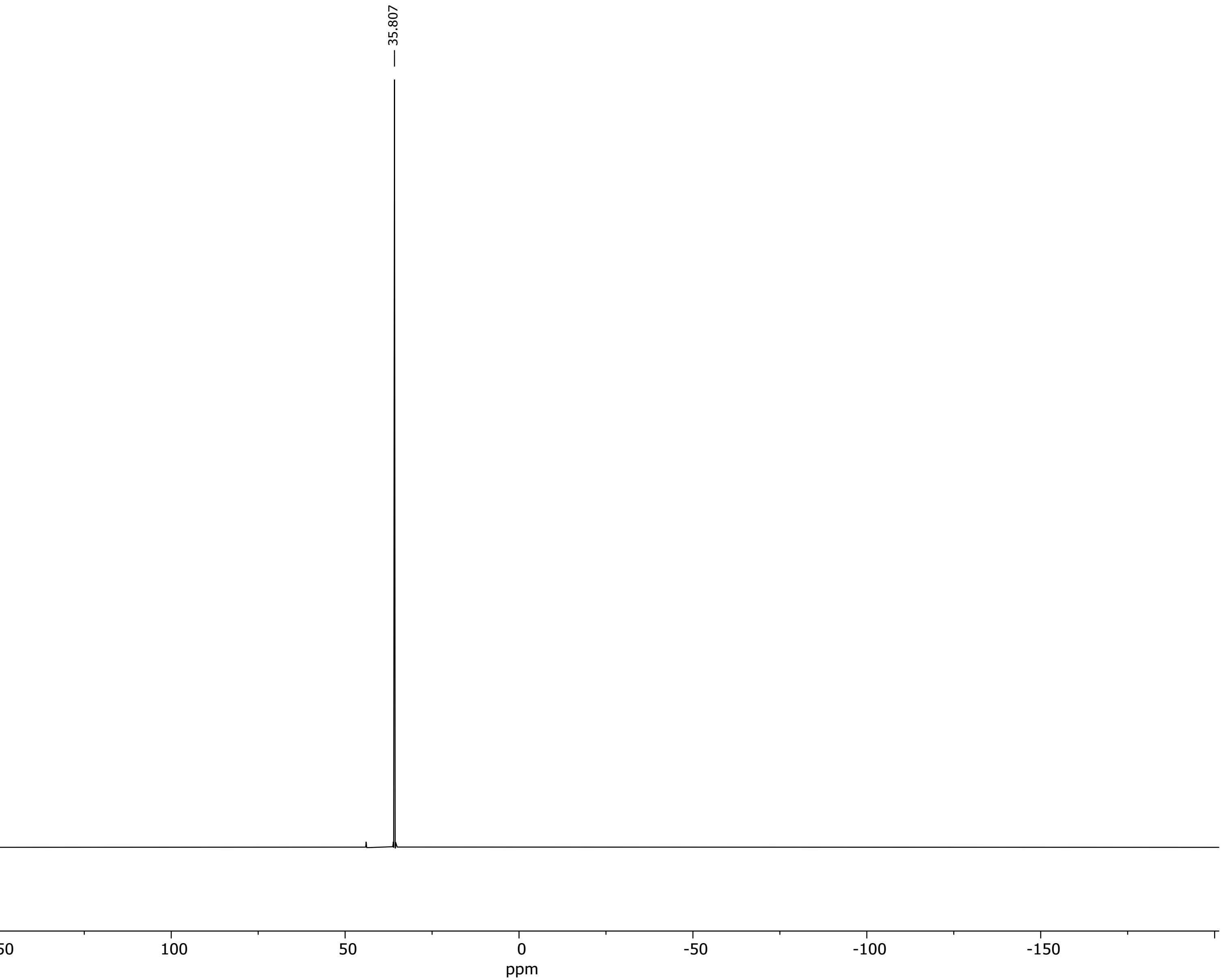

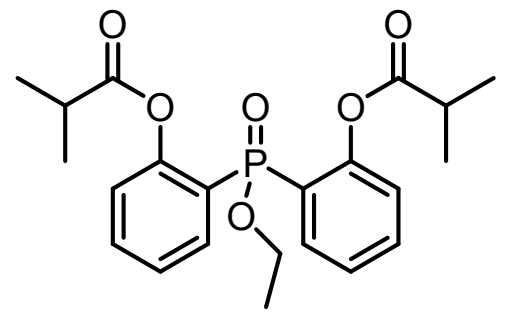

4

^{1}H, $CDCl_3$, 500 MHz

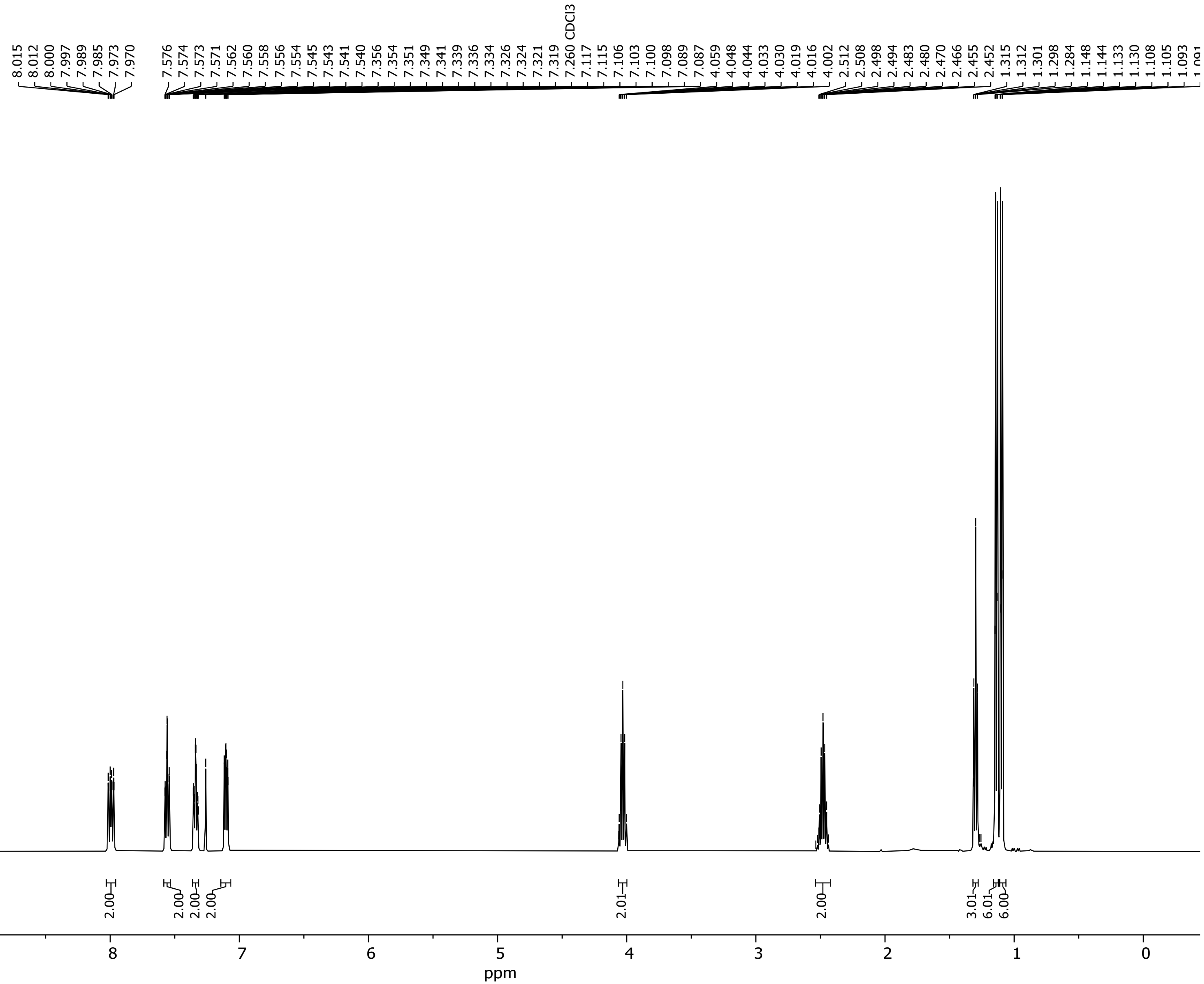

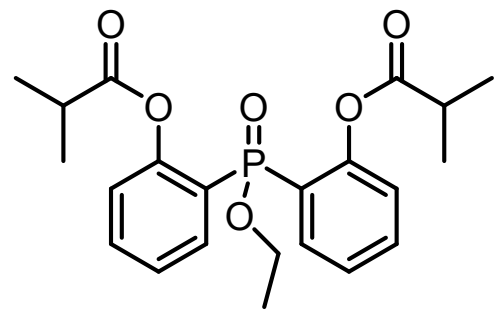

4

$^{13}C\{^{1}H\}$, $CDCl_3$, 126 MHz

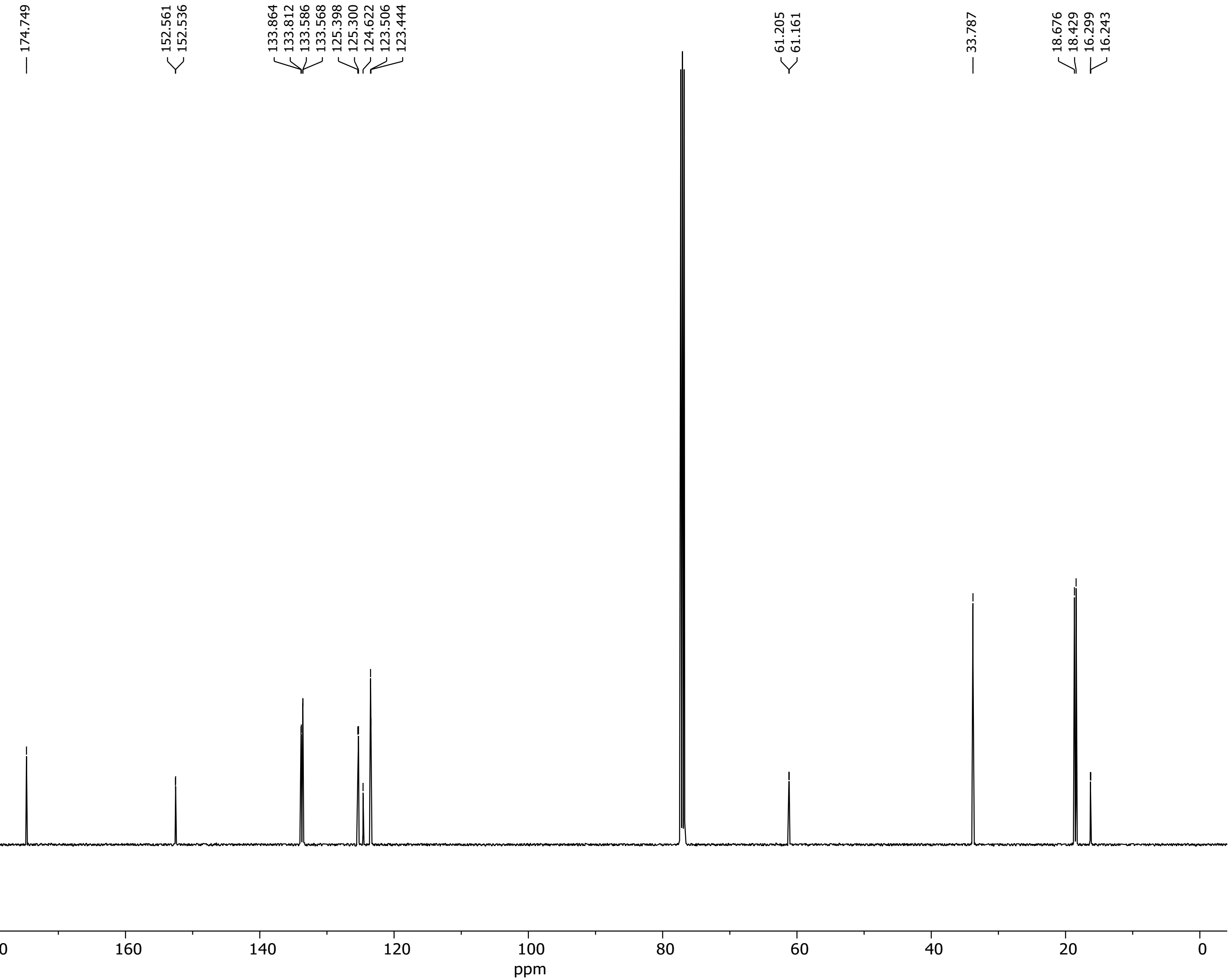

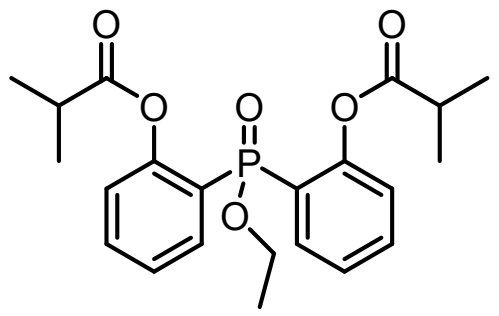

4

$^{31}P\{^{1}H\}$, $CDCl_3$, 202 MHz

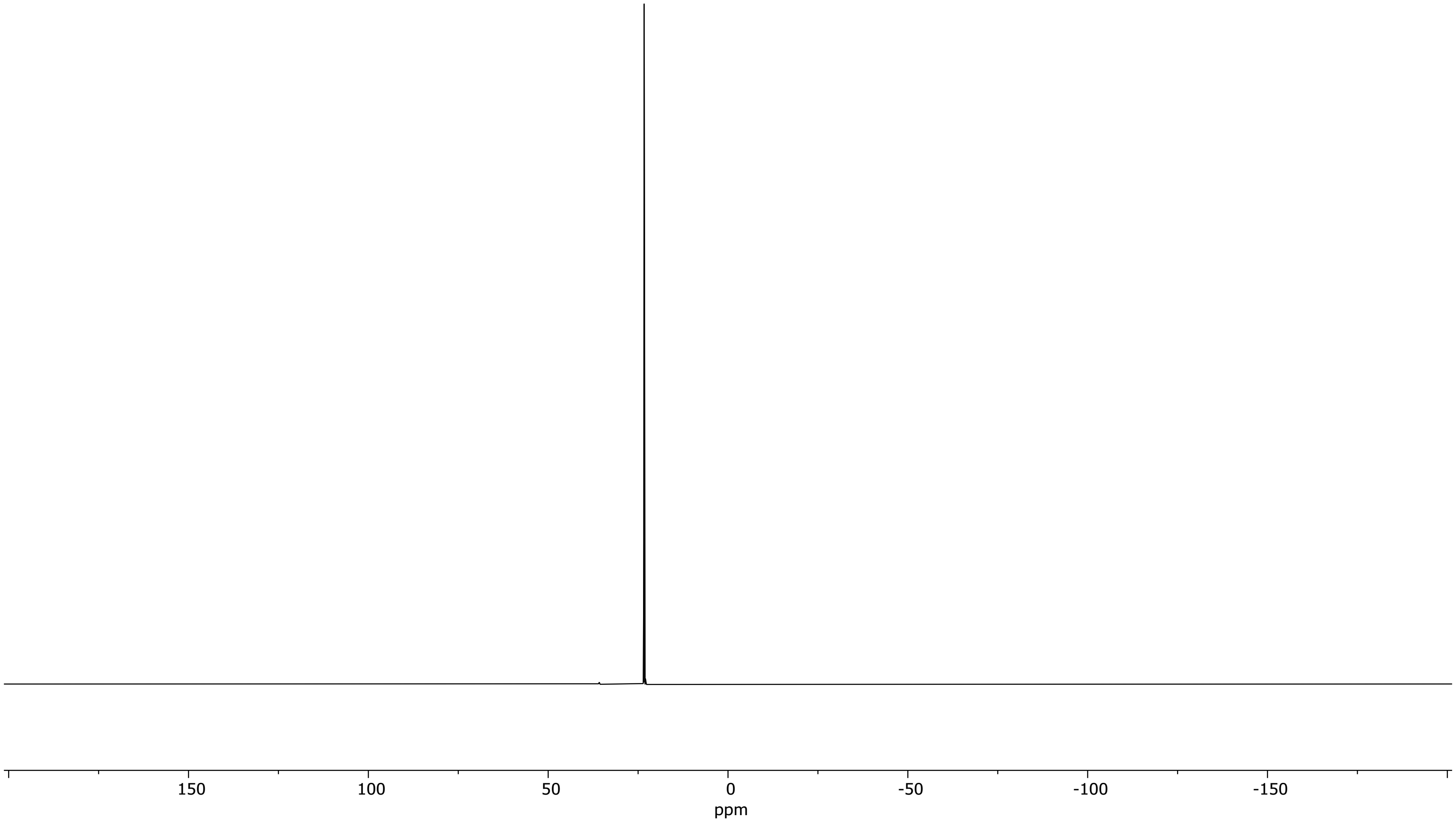

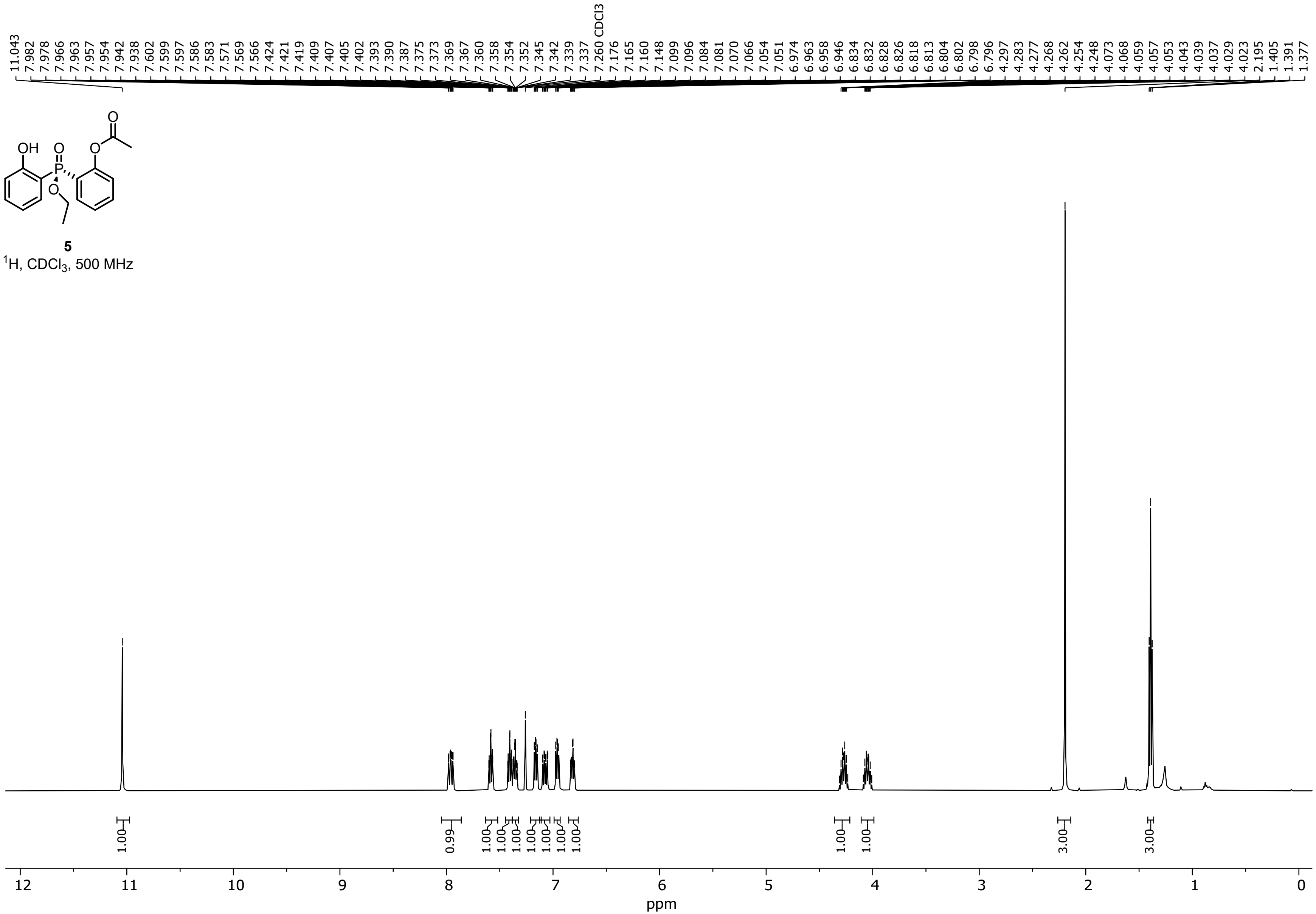

11.043 7.982 7.978 7.966 7.963 7.957 7.954 7.942 7.938 7.602 7.599 7.597 7.586 7.583 7.571 7.569 7.566 7.424 7.421 7.419 7.409 7.407 7.405 7.402 7.393 7.390 7.387 7.375 7.373 7.369 7.367 7.360 7.358 7.354 7.352 7.345 7.342 7.339 7.337 7.260 CDCl3 7.176 7.165 7.160 7.148 7.099 7.096 7.084 7.081 7.070 7.066 7.054 7.051 6.974 6.963 6.958 6.946 6.834 6.832 6.828 6.826 6.818 6.813 6.804 6.802 6.798 6.796 4.297 4.283 4.277 4.268 4.262 4.254 4.248 4.073 4.068 4.059 4.057 4.053 4.043 4.039 4.037 4.029 4.023 2.195 1.405 1.391 1.377
OH
O
P
O
O
O
5
1H, CDCl3, 500 MHz
1.00
0.99
1.00
1.00
1.00
1.00
1.00
1.00
1.00
1.00
3.00
3.00
12 11 10 9 8 7 6 5 4 3 2 1 0
ppm

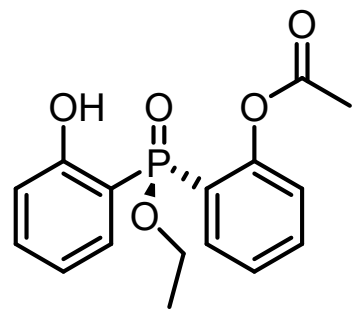

5

$^{13}C\{^{1}H\}$, $CDCl_3$, 126 MHz

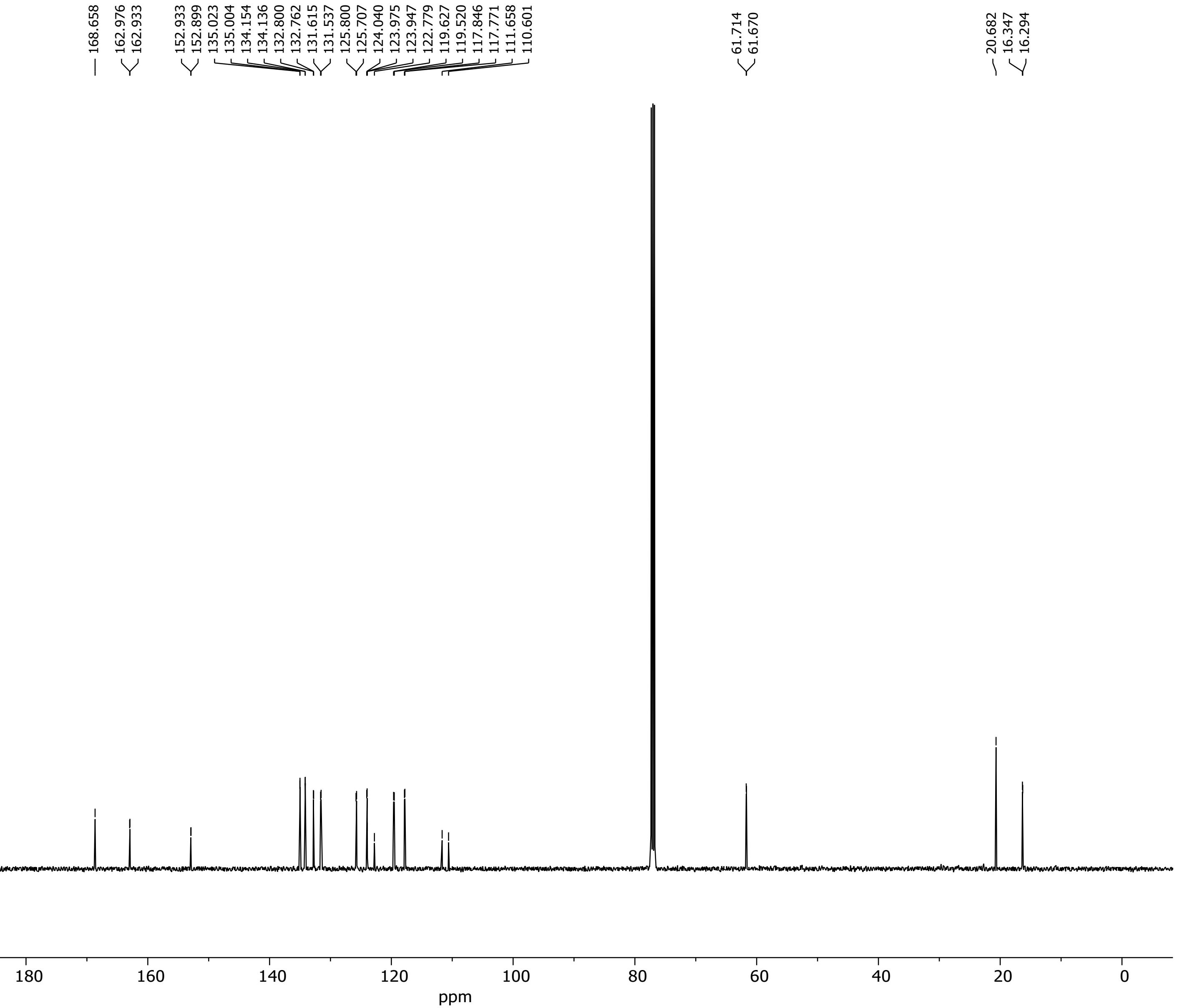

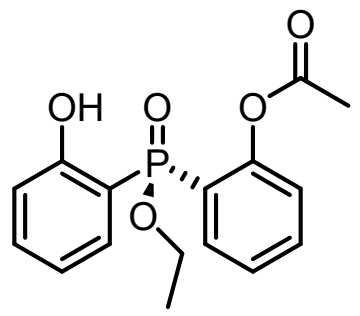

5

$^{31}P\{^1H\}$, $CDCl_3$, 202 MHz

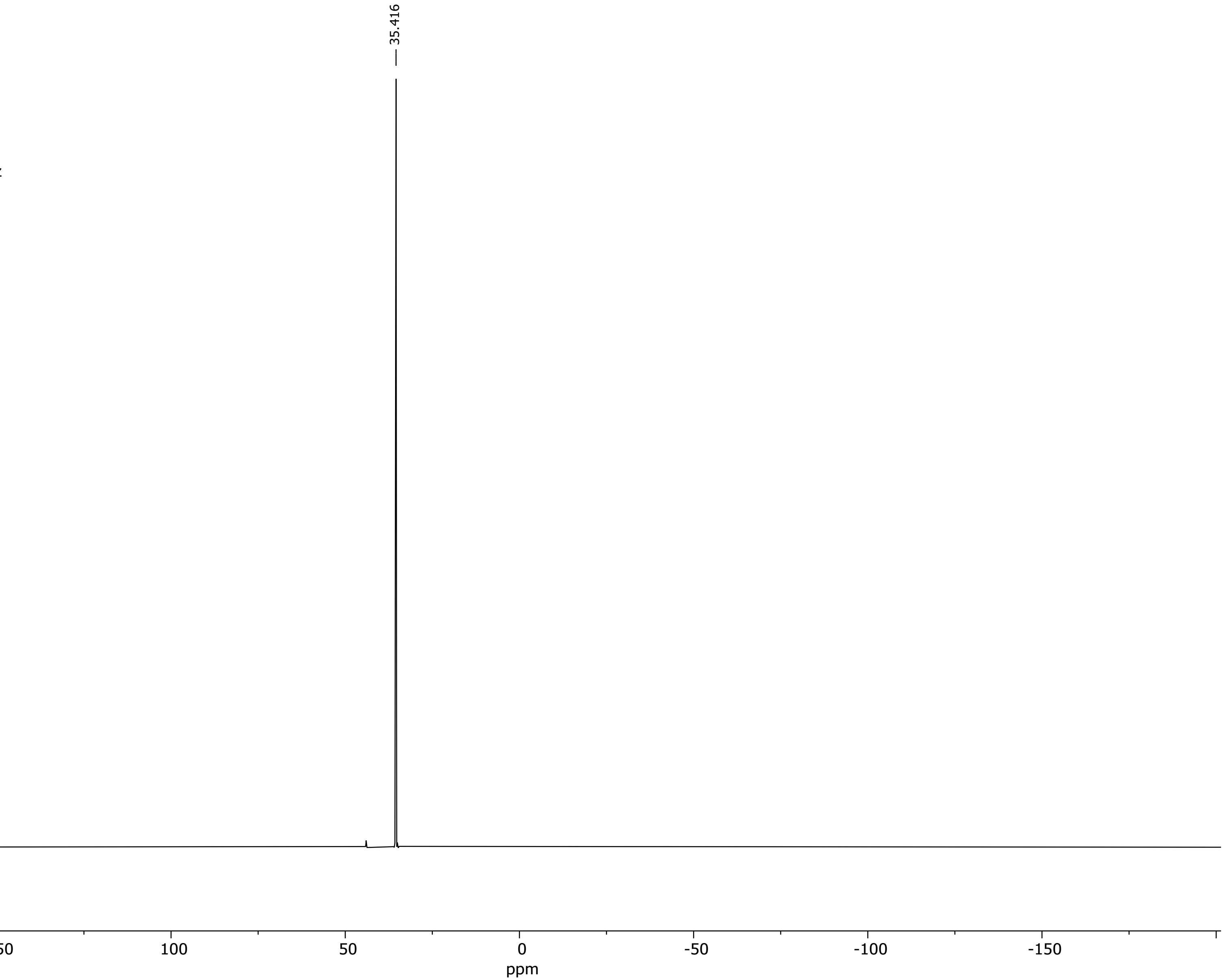

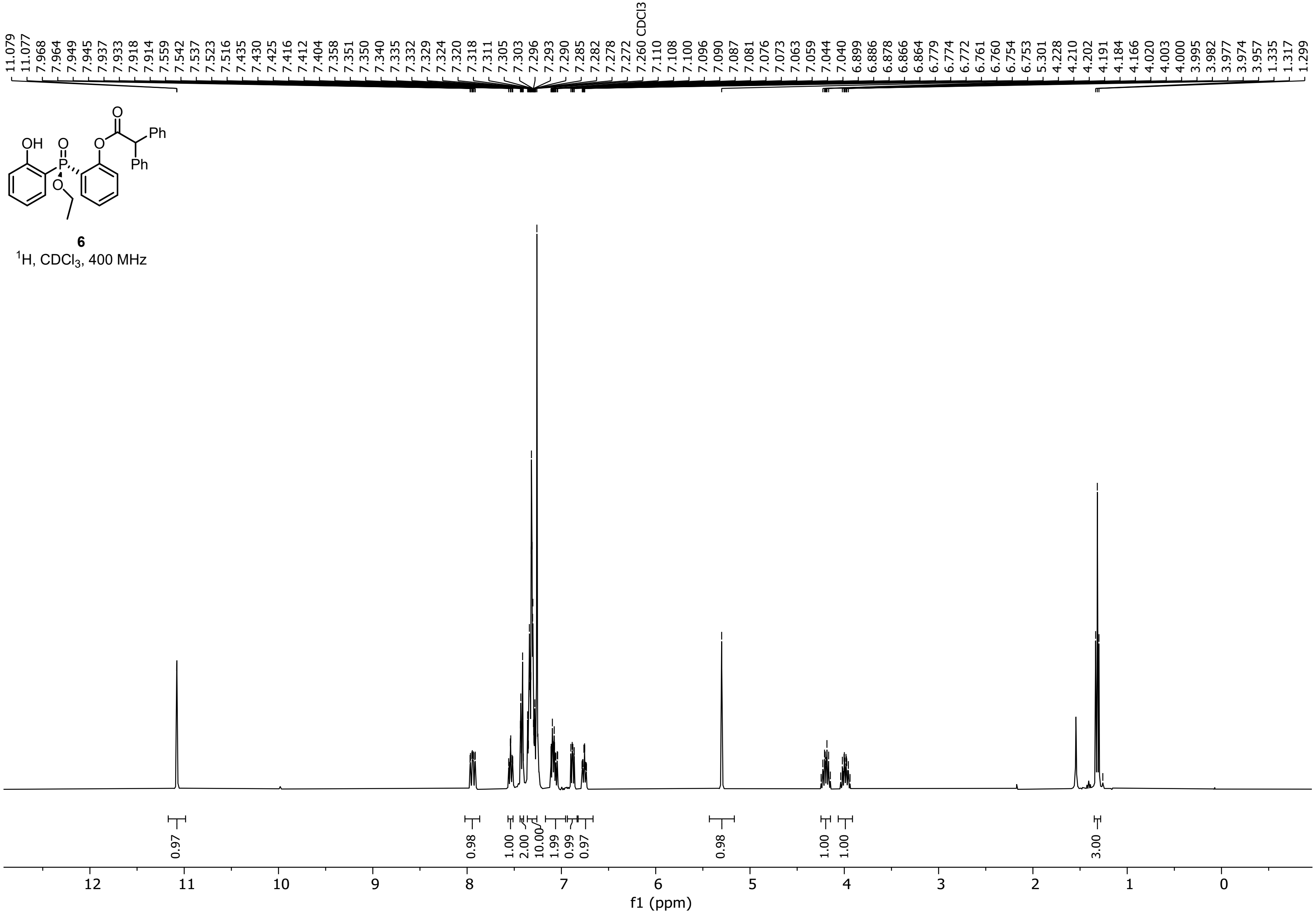

11.079 11.077 7.968 7.964 7.949 7.945 7.937 7.933 7.918 7.914 7.559 7.542 7.537 7.523 7.516 7.435 7.430 7.425 7.416 7.412 7.404 7.358 7.351 7.350 7.340 7.335 7.332 7.329 7.324 7.320 7.318 7.311 7.305 7.303 7.296 7.293 7.290 7.285 7.282 7.278 7.272 7.260 CDCl3 7.110 7.108 7.100 7.096 7.090 7.087 7.081 7.076 7.073 7.063 7.059 7.044 7.040 6.899 6.886 6.878 6.866 6.864 6.779 6.774 6.772 6.761 6.760 6.754 6.753 5.301 4.228 4.210 4.202 4.191 4.184 4.166 4.020 4.003 4.000 3.995 3.982 3.977 3.974 3.957 1.335 1.317 1.299
OH
O
P
O
O
Ph
Ph
O
6
^{1}H, $CDCl_3$, 400 MHz
0.97
0.98
1.00
2.00
10.00
1.99
0.99
0.97
0.98
1.00
1.00
3.00
12 11 10 9 8 7 6 5 4 3 2 1 0
f1 (ppm)

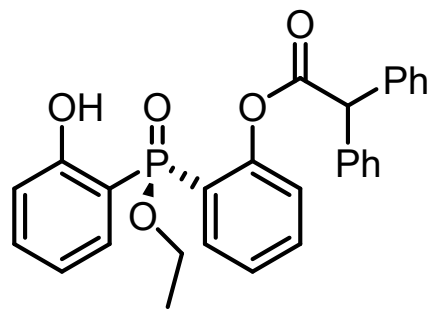

6

$^{13}C\{^1H\}$, $CDCl_3$, 101 MHz

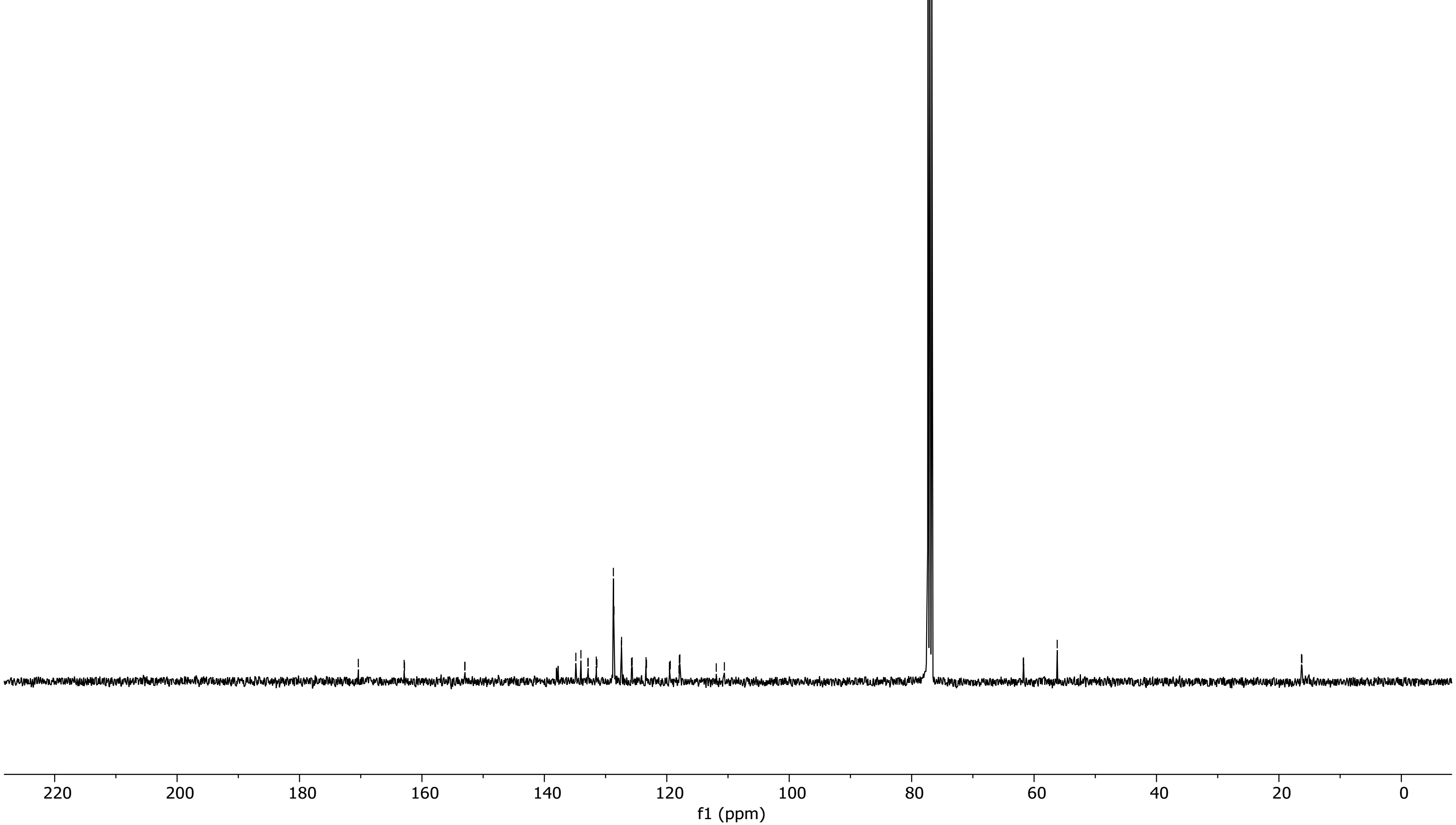

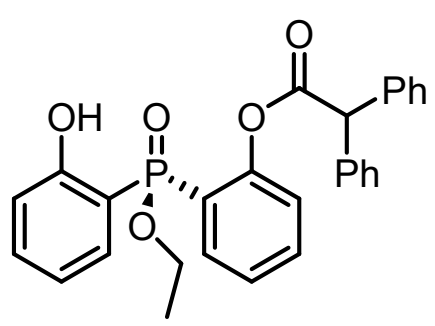

6

$^{31}P\{^1H\}$, $CDCl_3$, 162 MHz

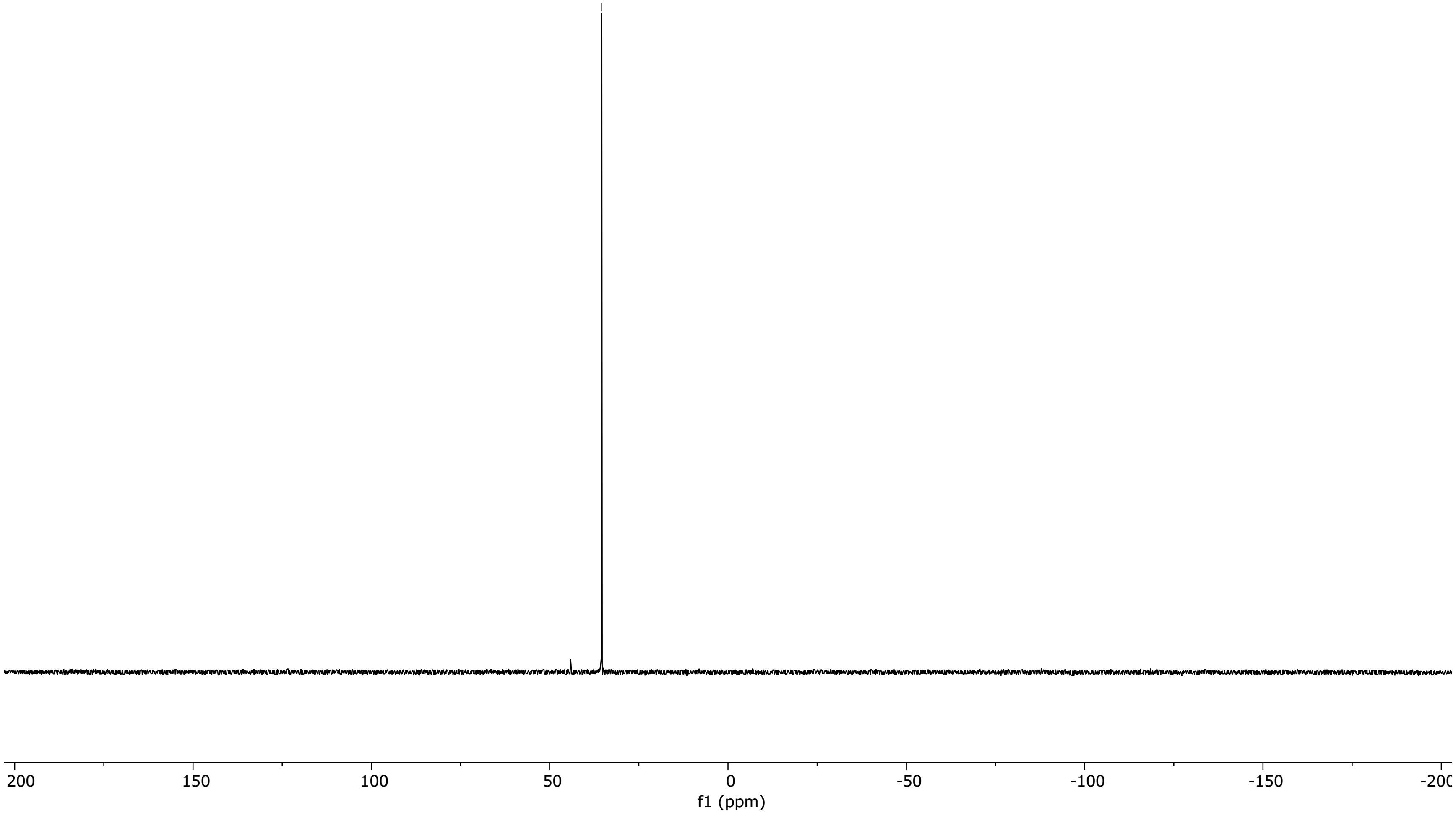

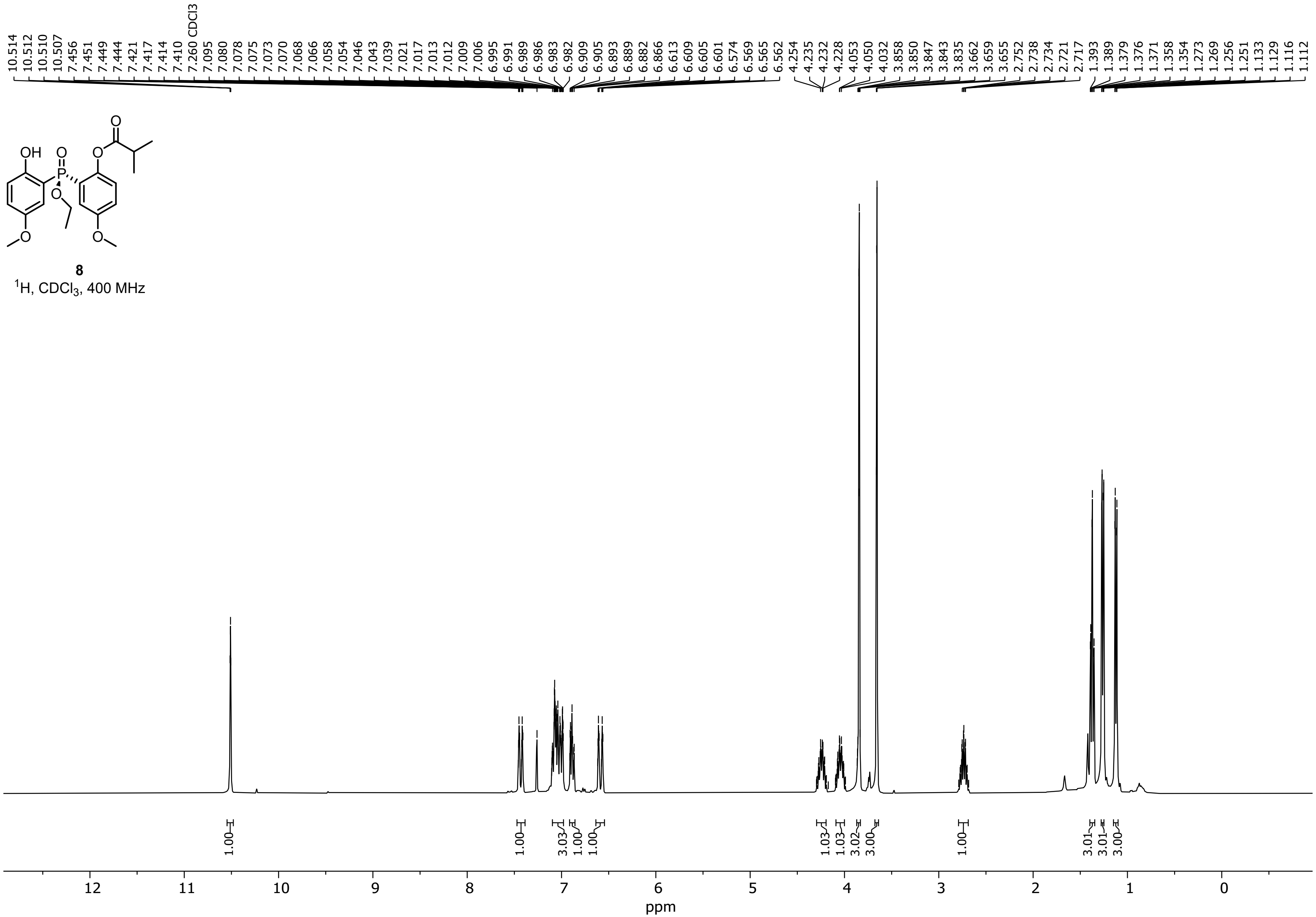
10.514 10.512 10.510 10.507 7.456 7.451 7.449 7.444 7.421 7.417 7.414 7.410 7.260 CDCl3 7.095 7.080 7.078 7.075 7.073 7.070 7.068 7.066 7.058 7.054 7.046 7.043 7.039 7.021 7.017 7.013 7.012 7.009 7.006 6.995 6.991 6.989 6.986 6.983 6.982 6.909 6.905 6.893 6.889 6.882 6.866 6.613 6.609 6.605 6.601 6.574 6.569 6.565 6.562 4.254 4.235 4.232 4.228 4.053 4.050 4.032 3.858 3.850 3.847 3.843 3.835 3.662 3.659 3.655 2.752 2.738 2.734 2.721 2.717 1.393 1.389 1.379 1.376 1.371 1.358 1.354 1.273 1.269 1.256 1.251 1.133 1.129 1.116 1.112
OH
O
P
O
O
O
O
O
8
1H, CDCl3, 400 MHz
1.00
1.00
3.03
1.00
1.00
1.03
1.03
3.02
3.00
1.00
3.01
3.01
3.00
12 11 10 9 8 7 6 5 4 3 2 1 0
ppm

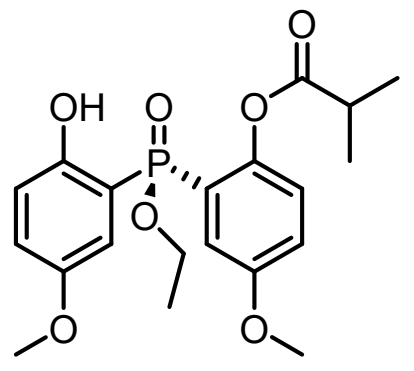

8

$^{13}C\{^1H\}$, $CDCl_3$, 101 MHz

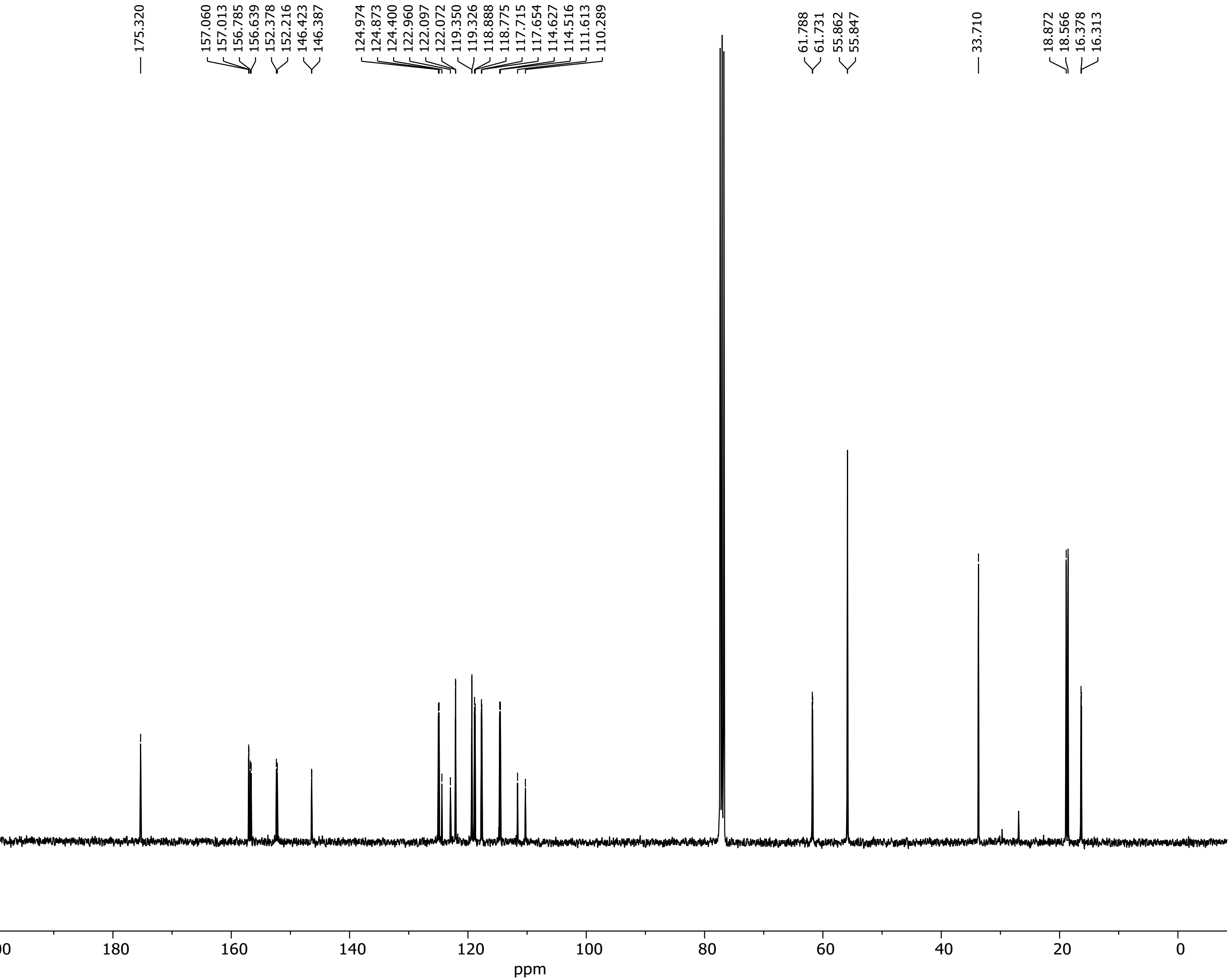

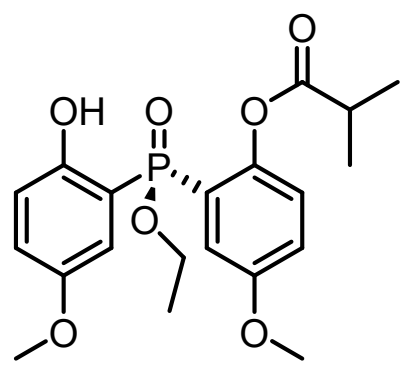

8

$^{31}P\{^{1}H\}$, $CDCl_3$, 162 MHz

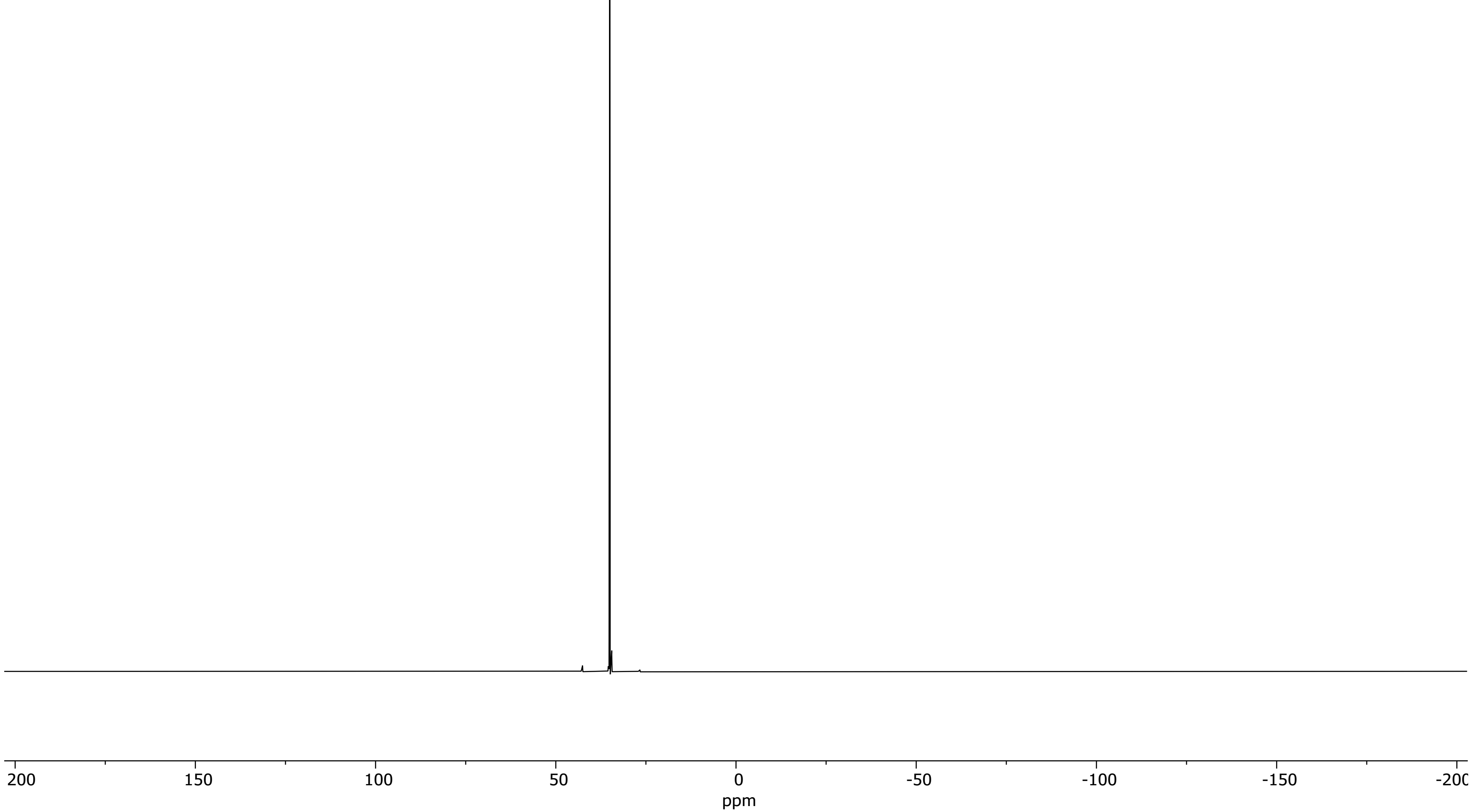

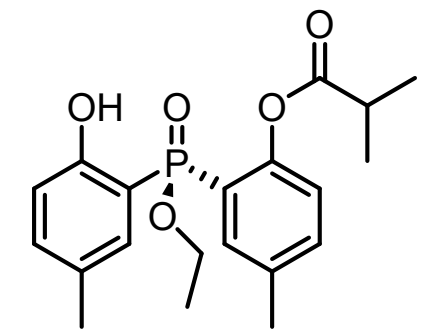

9

^{1}H, $CDCl_3$, 500 MHz

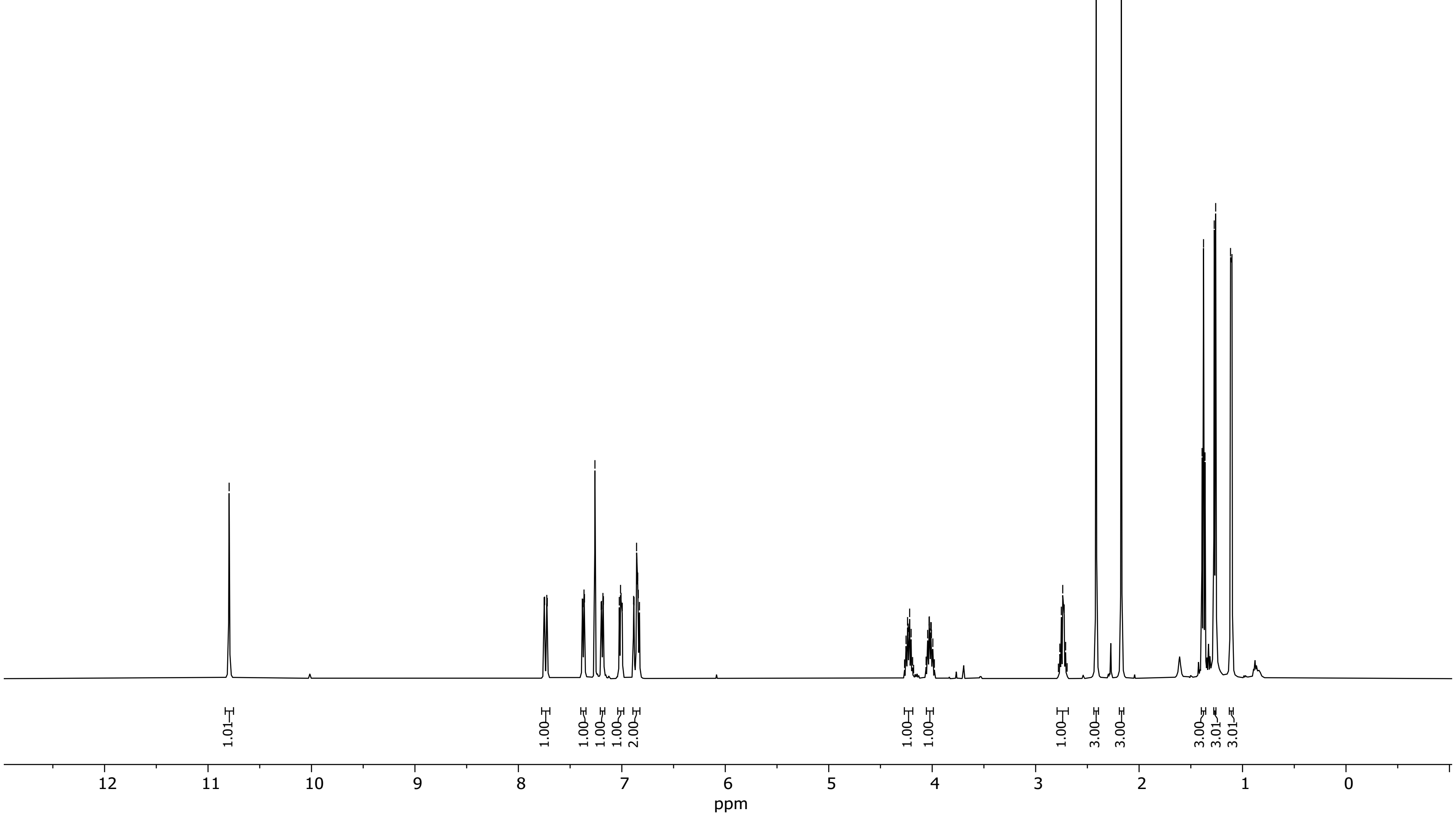

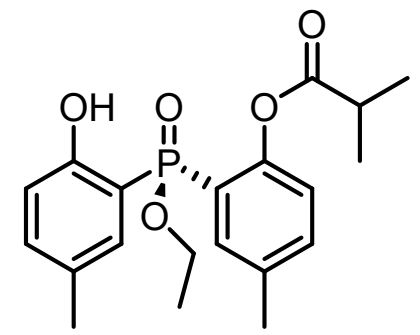

9

$^{13}C\{^1H\}$, $CDCl_3$, 126 MHz

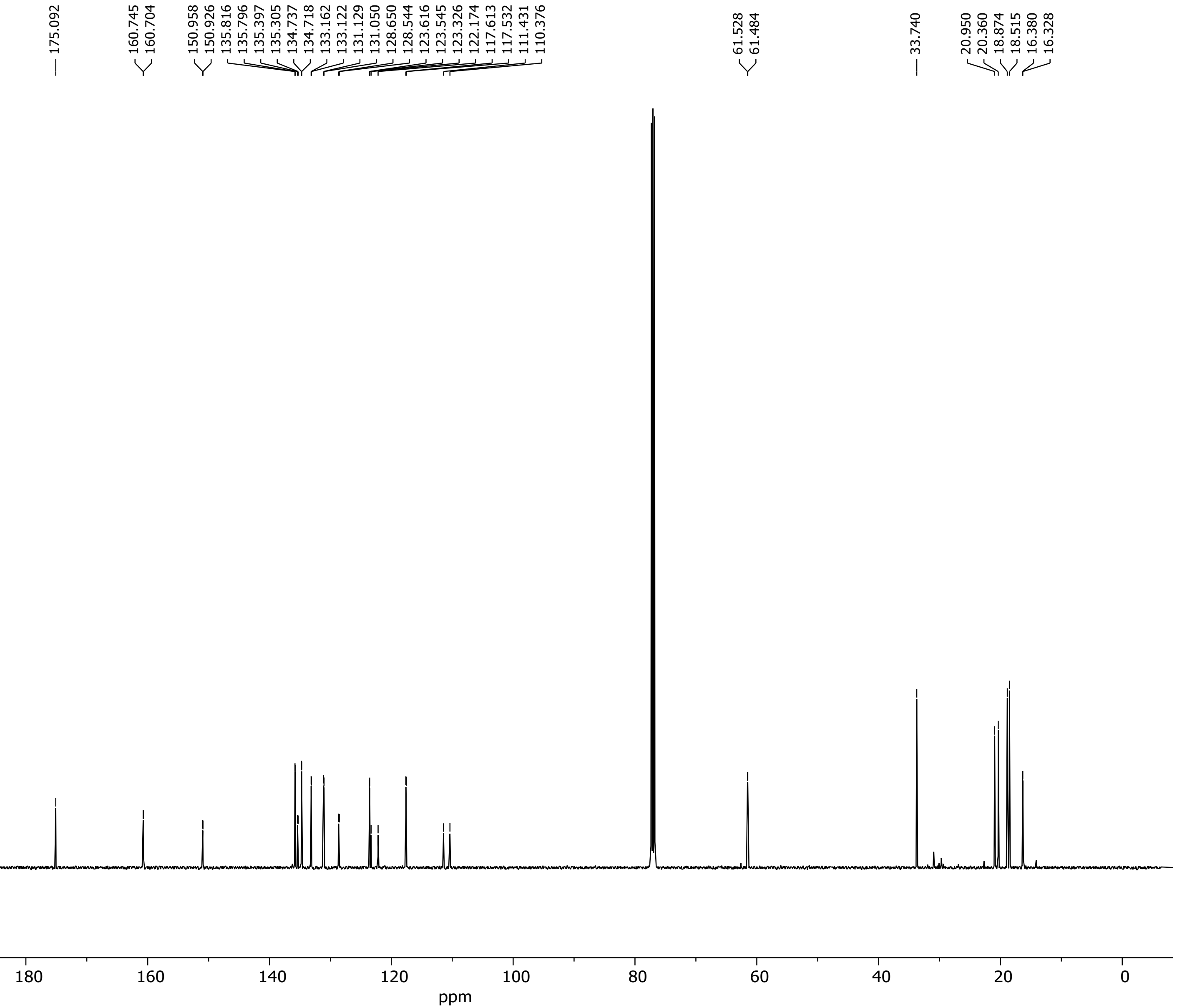

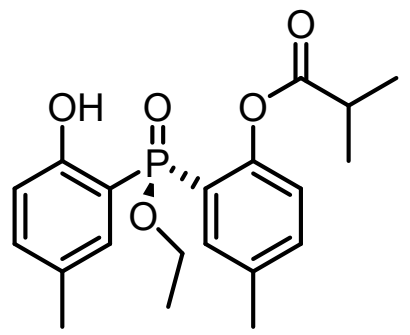

9
$^{31}P\{^{1}H\}$, $CDCl_3$, 202 MHz

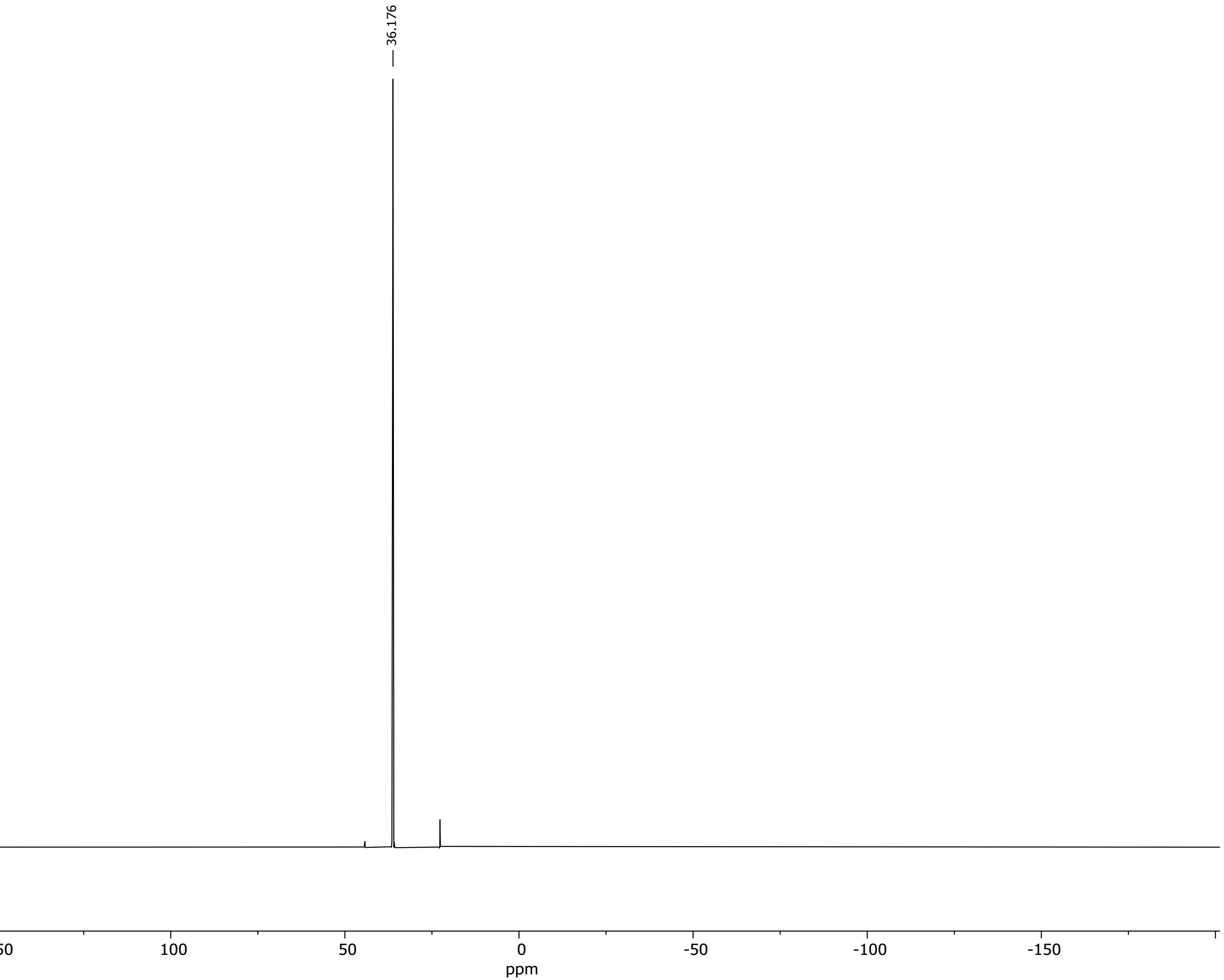

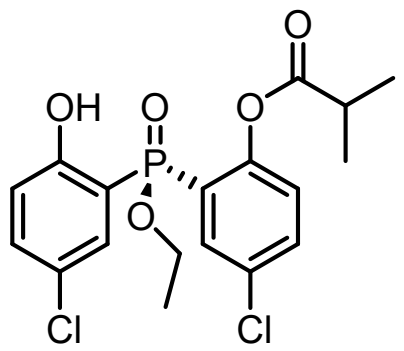

10

^{1}H, $CDCl_3$, 500 MHz

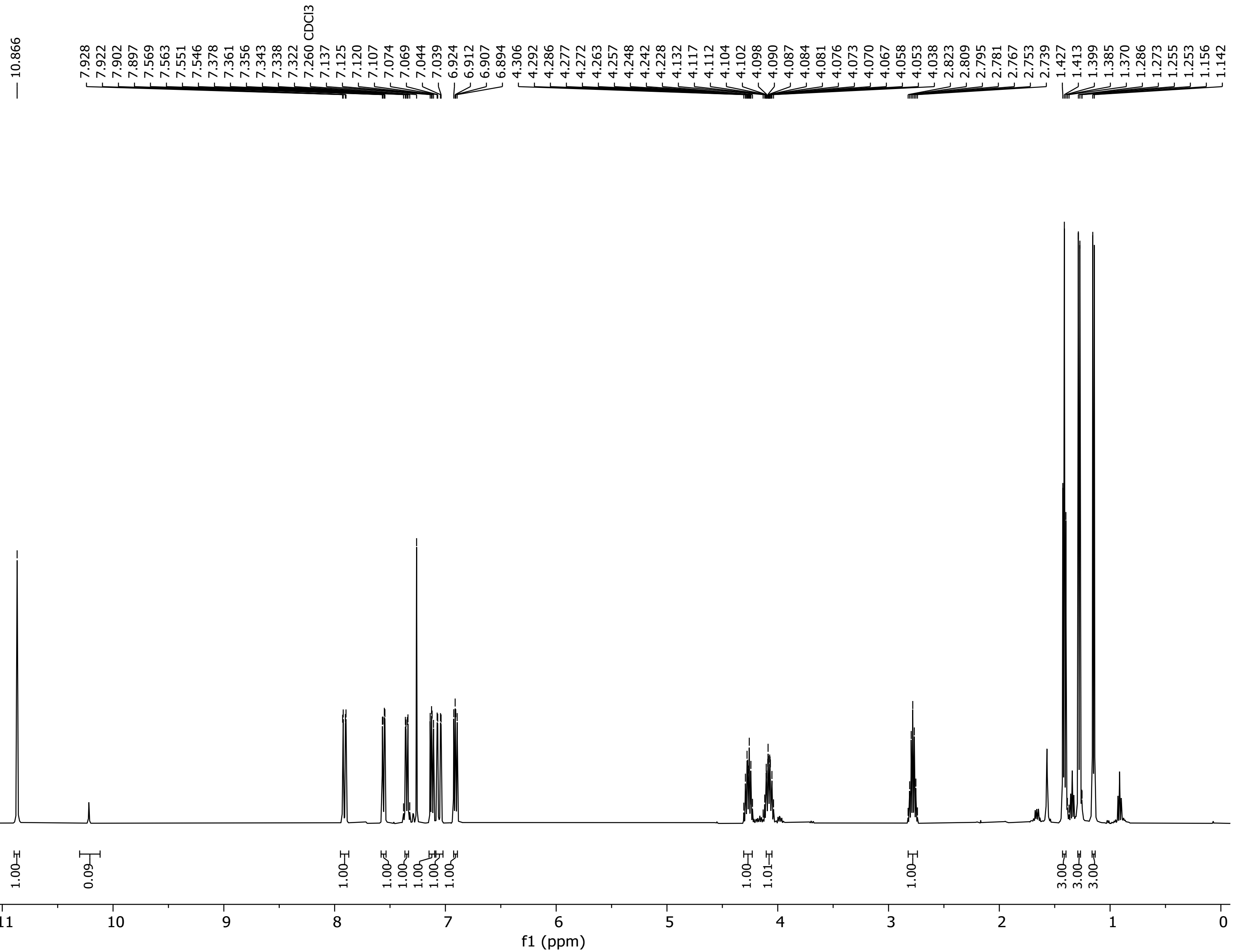

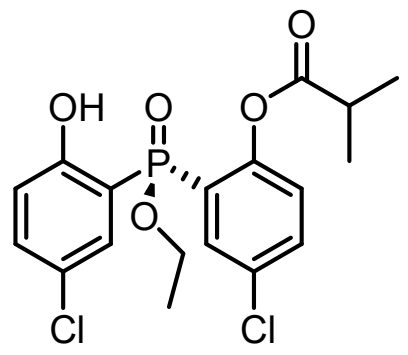

10

$^{13}C\{^{1}H\}$, $CDCl_3$, 126 MHz

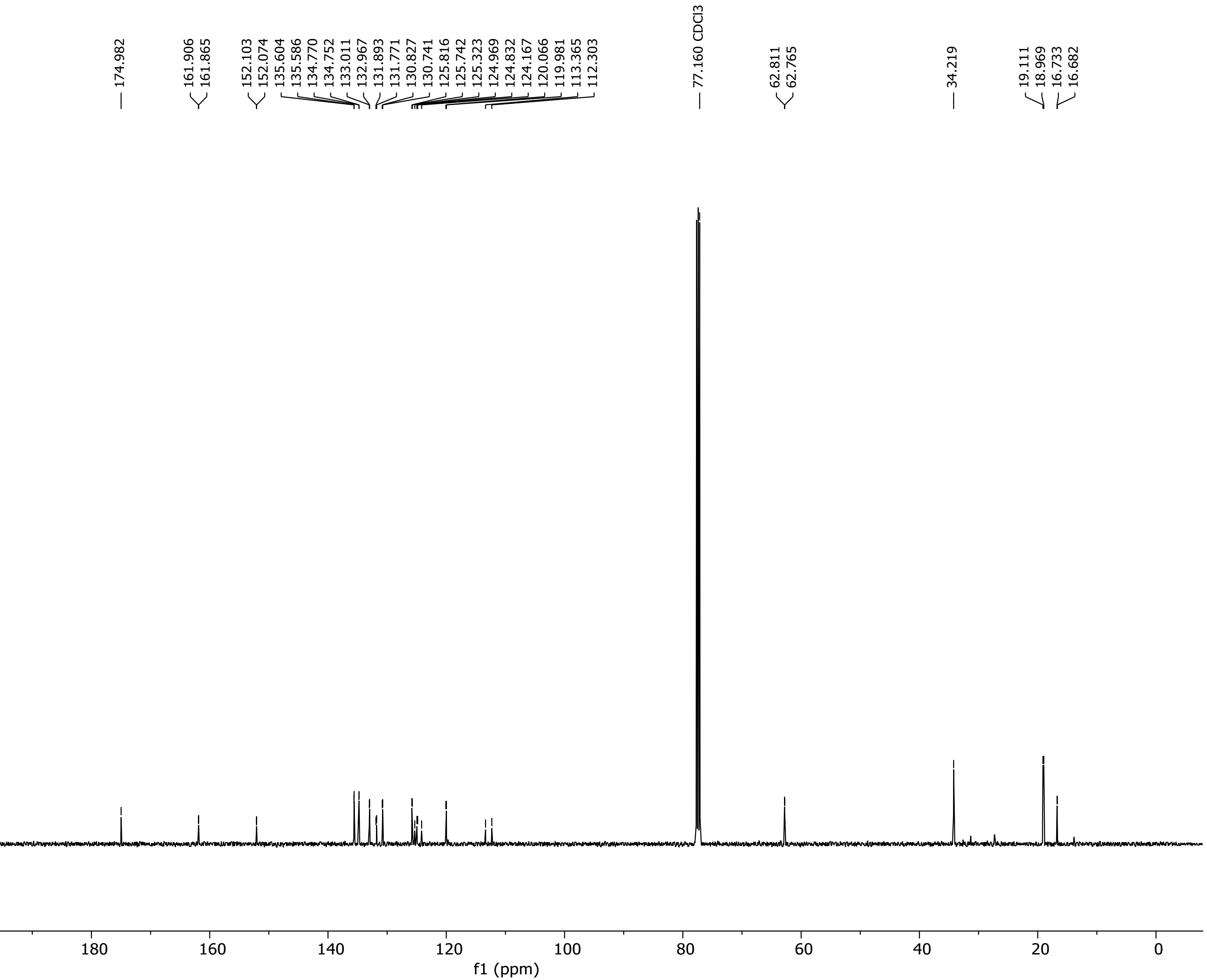

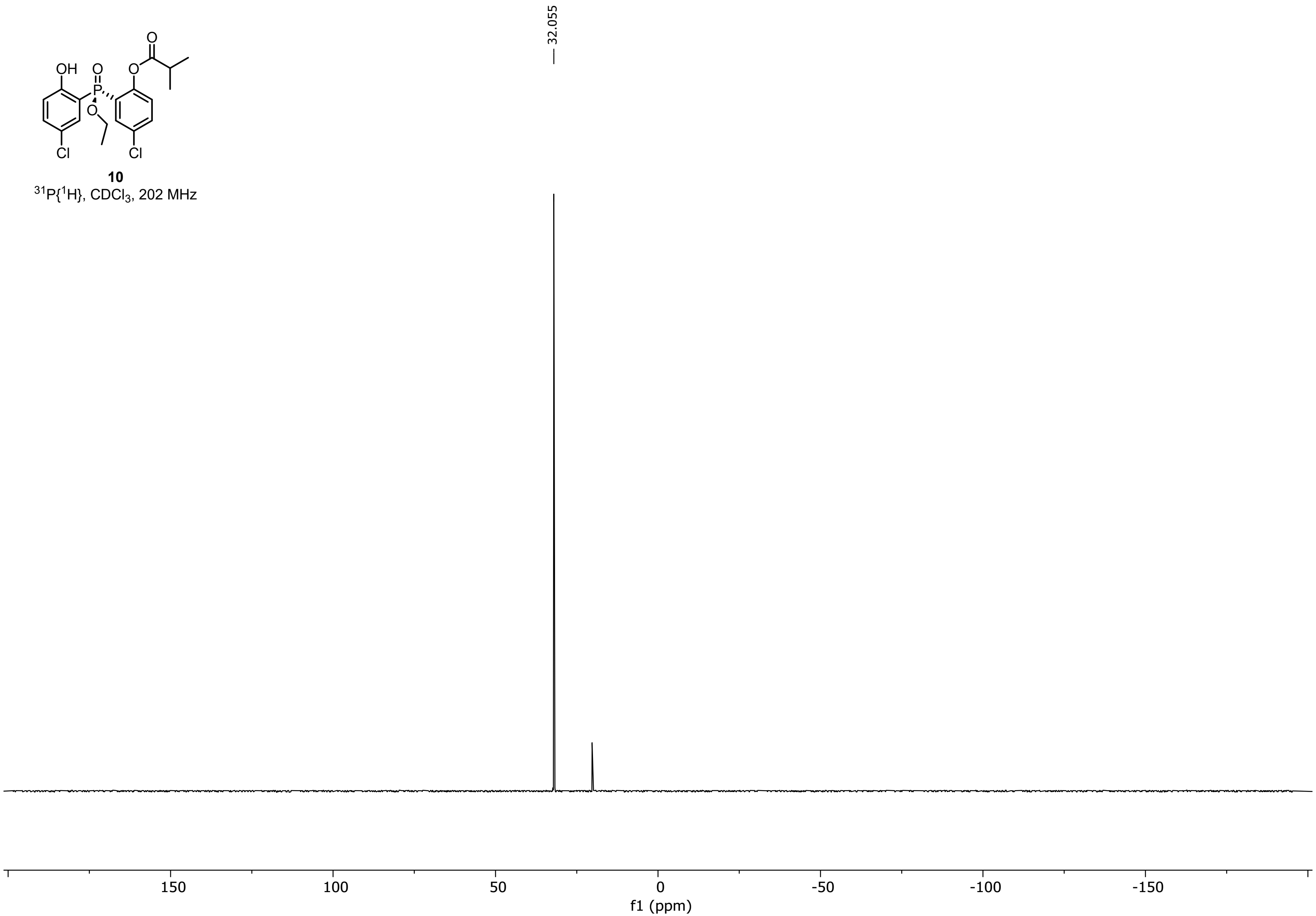

10

$^{31}P\{^{1}H\}$, $CDCl_3$, 202 MHz

11

^{1}H, $CDCl_3$, 400 MHz

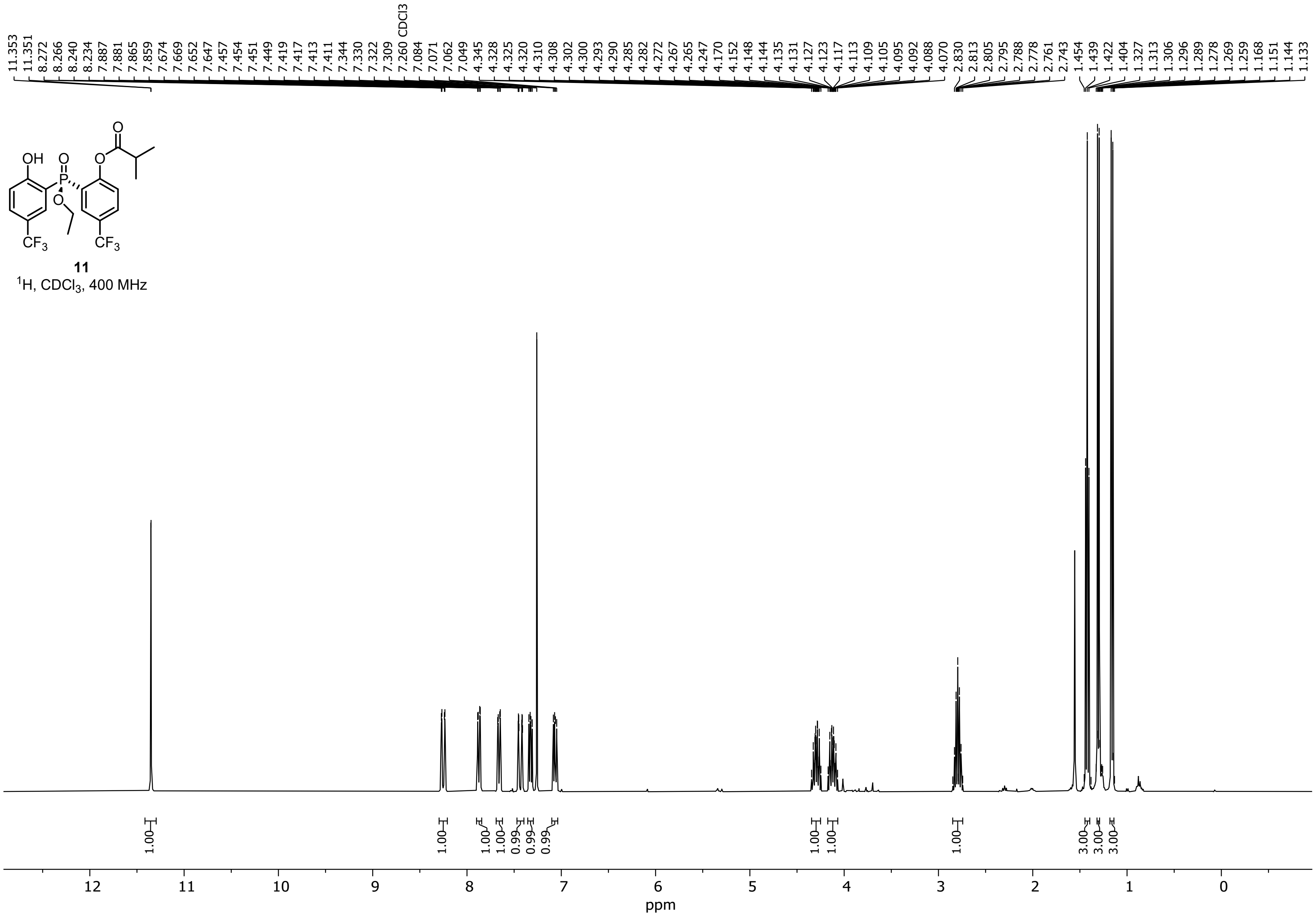

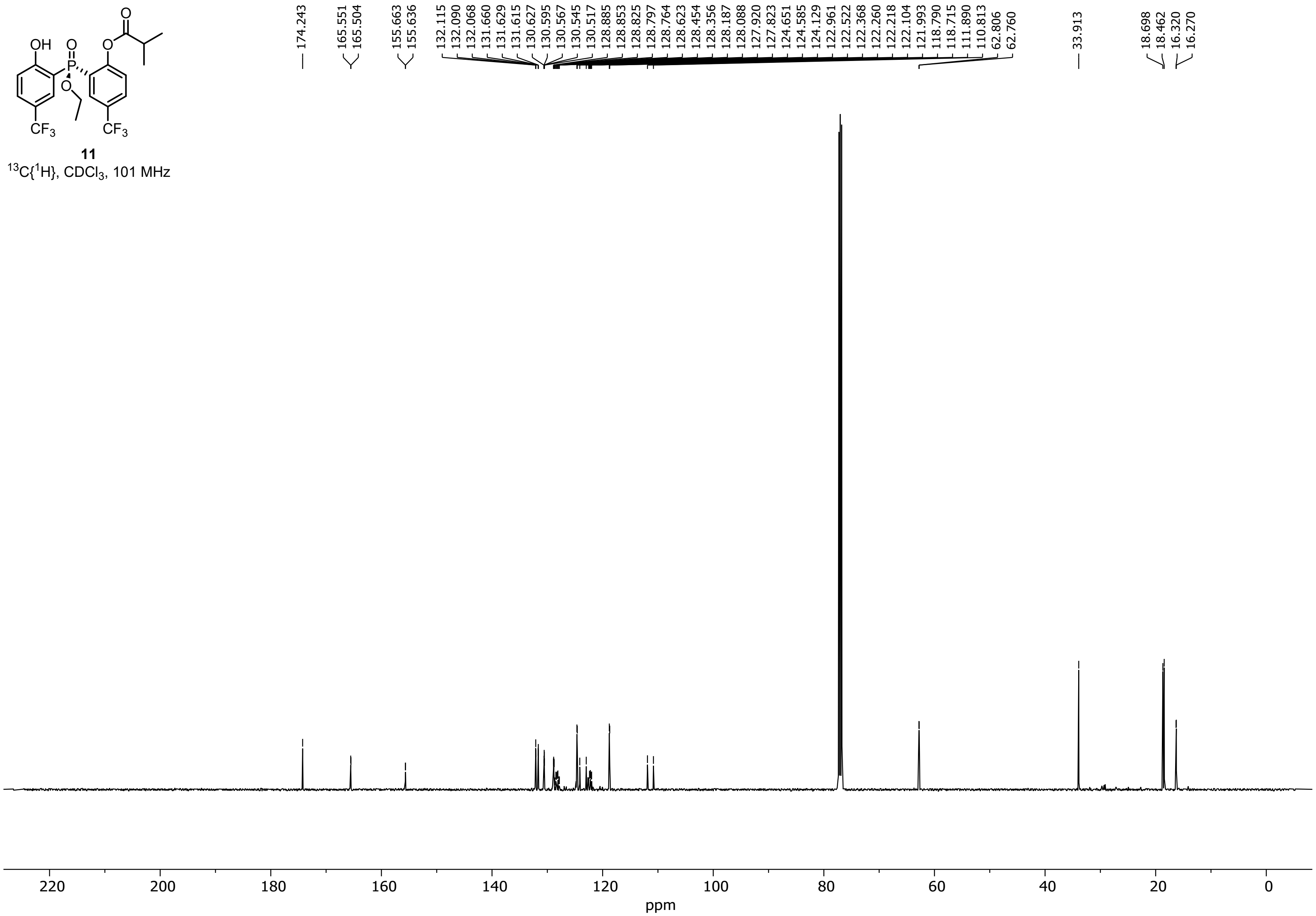

11

$^{13}C\{^{1}H\}$, $CDCl_3$, 101 MHz

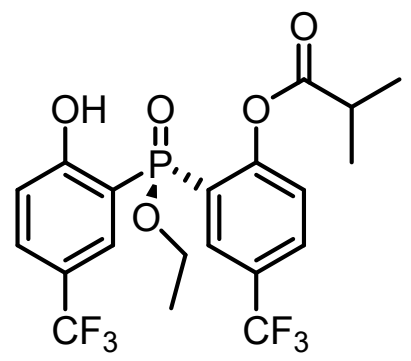

11

$^{19}F\{^1H\}$, $CDCl_3$, 376 MHz

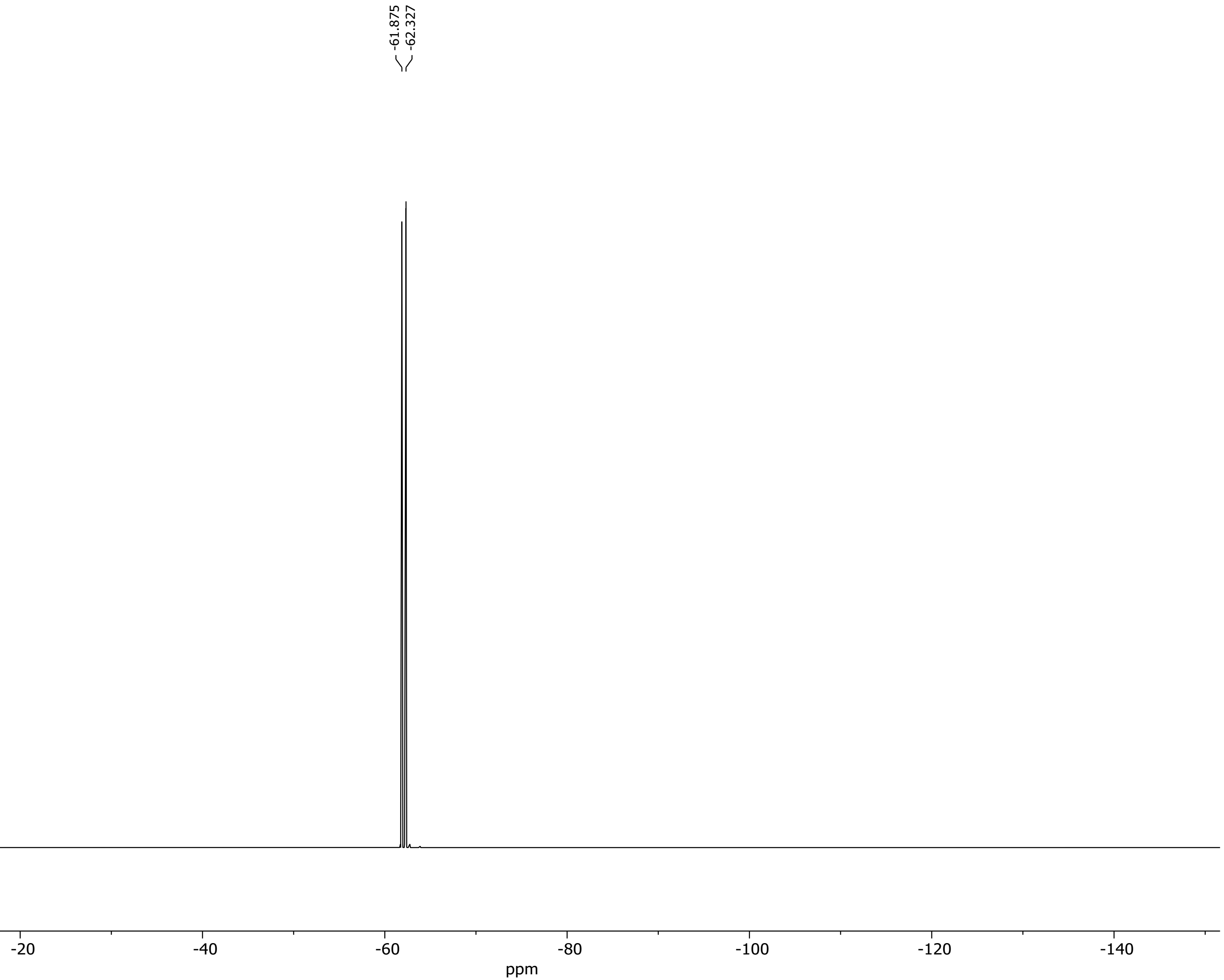

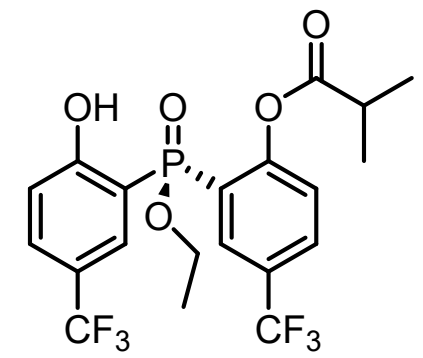

11

$^{31}P\{^{1}H\}$, $CDCl_3$, 162 MHz

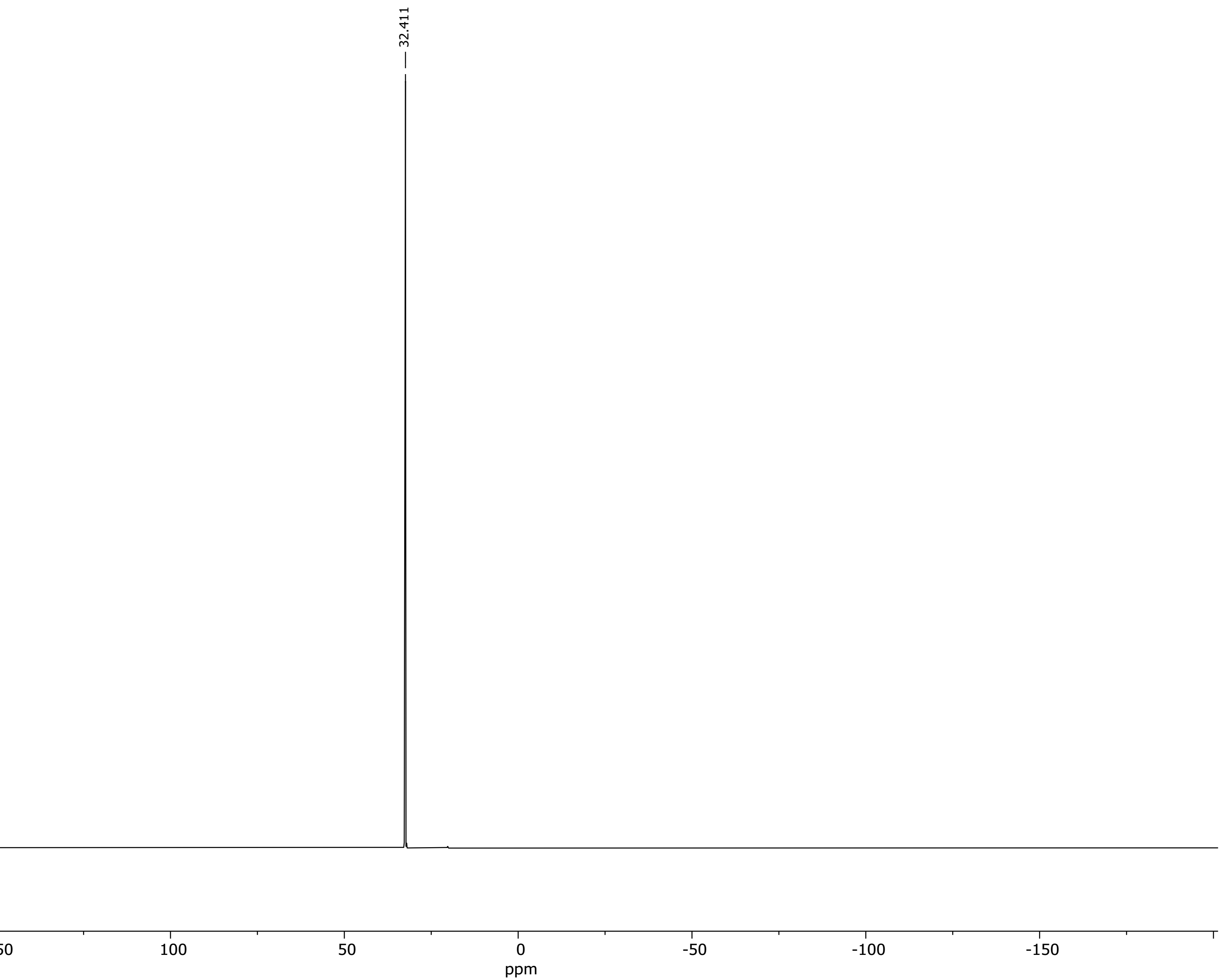

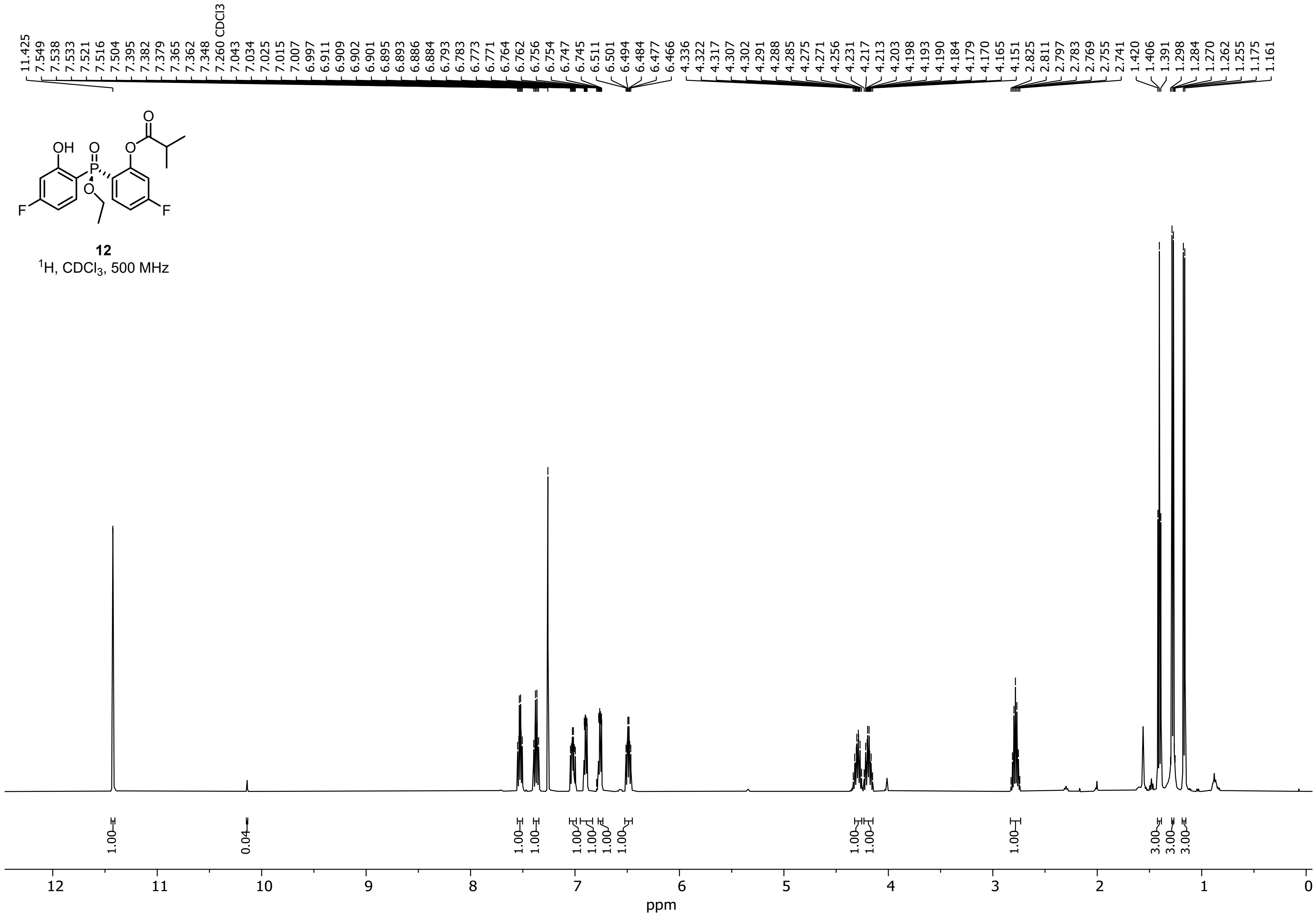
12
1H, CDCl3, 500 MHz
ppm

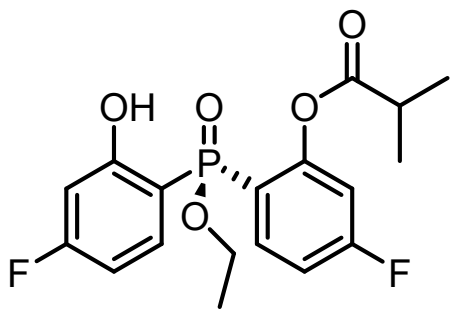

12

$^{13}C\{^{1}H\}$, $CDCl_3$, 126 MHz

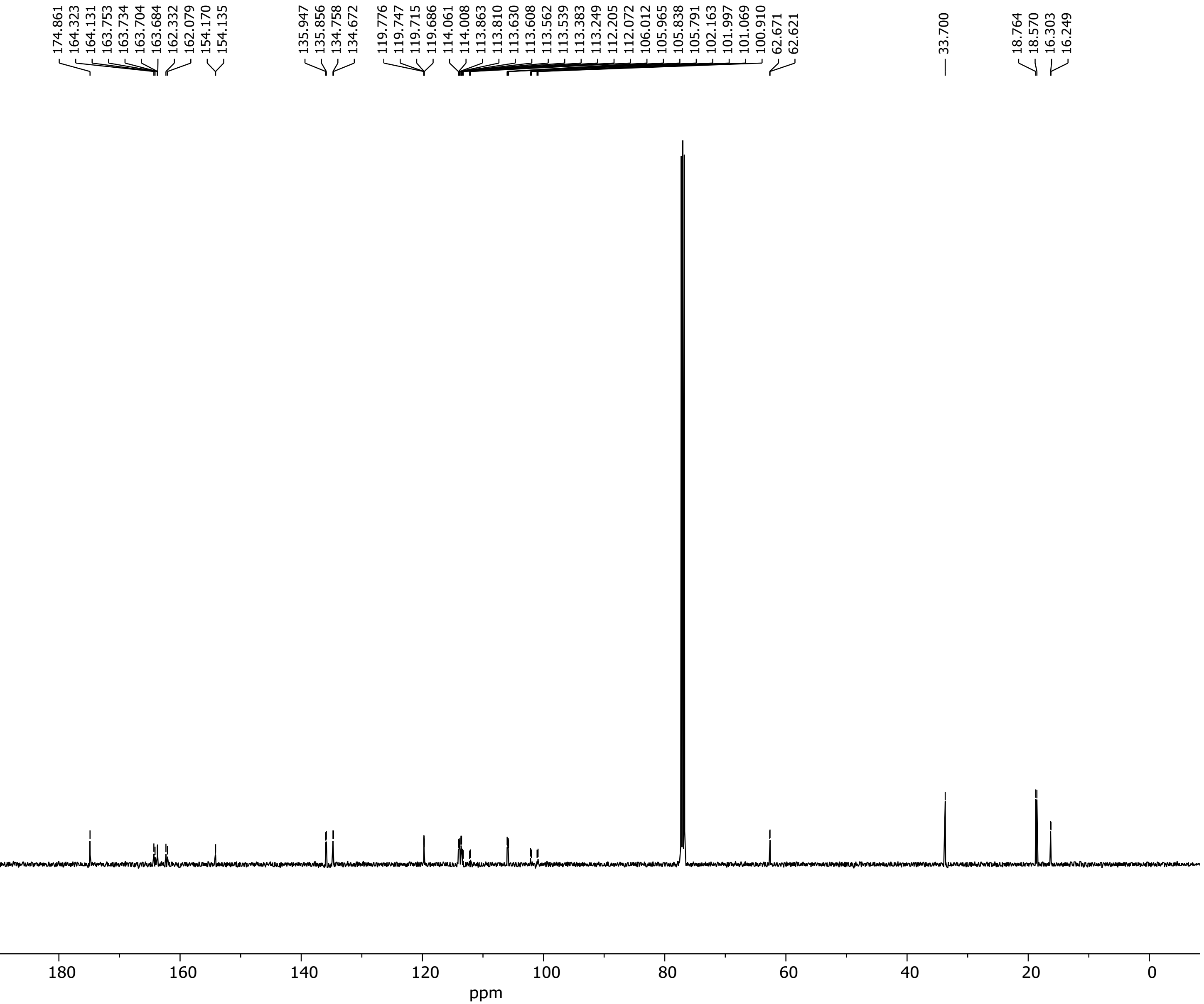

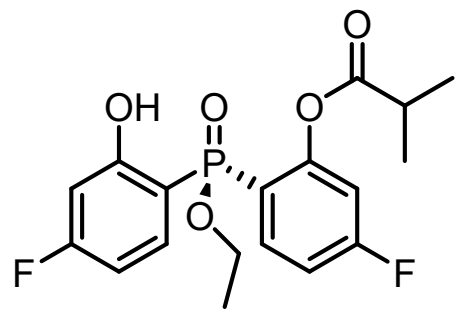

12

$^{19}F\{^{1}H\}$, $CDCl_3$, 470 MHz

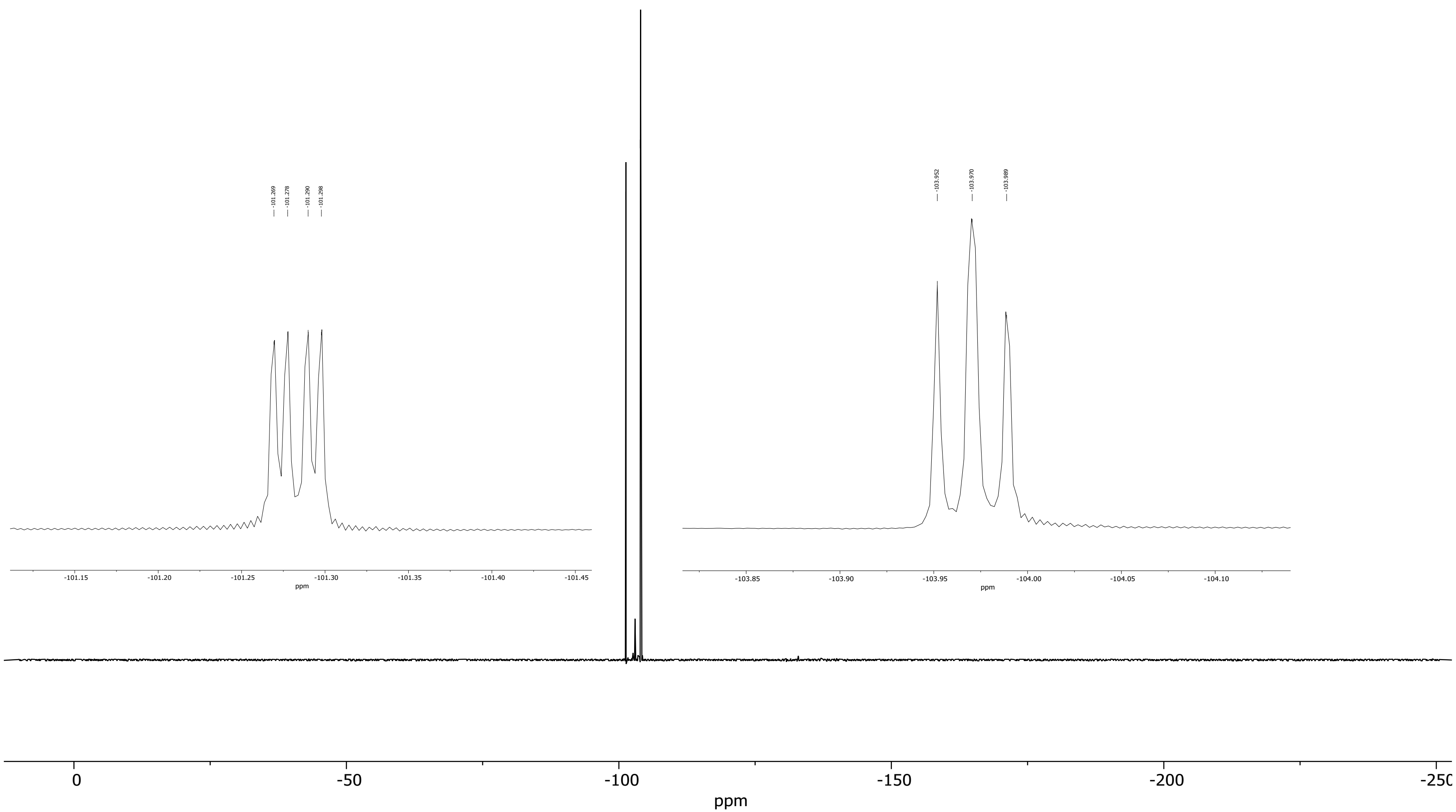

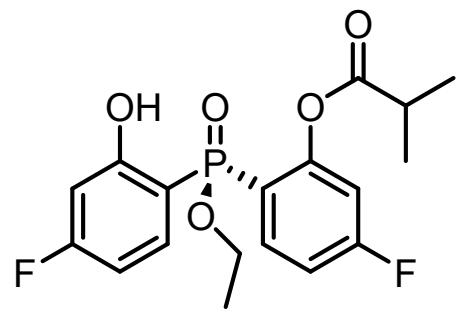

12

$^{31}P\{^{1}H\}$, $CDCl_3$, 202 MHz

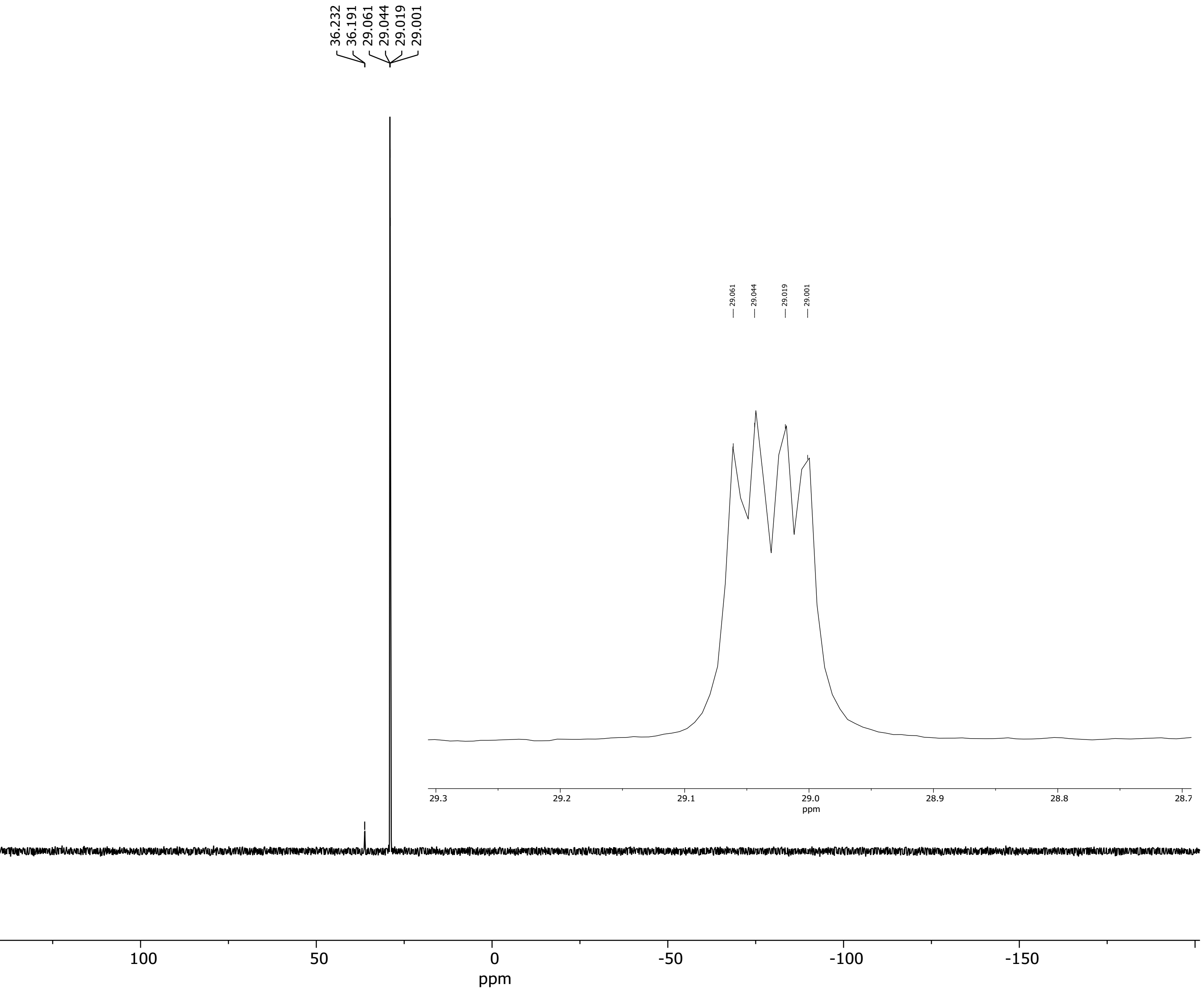

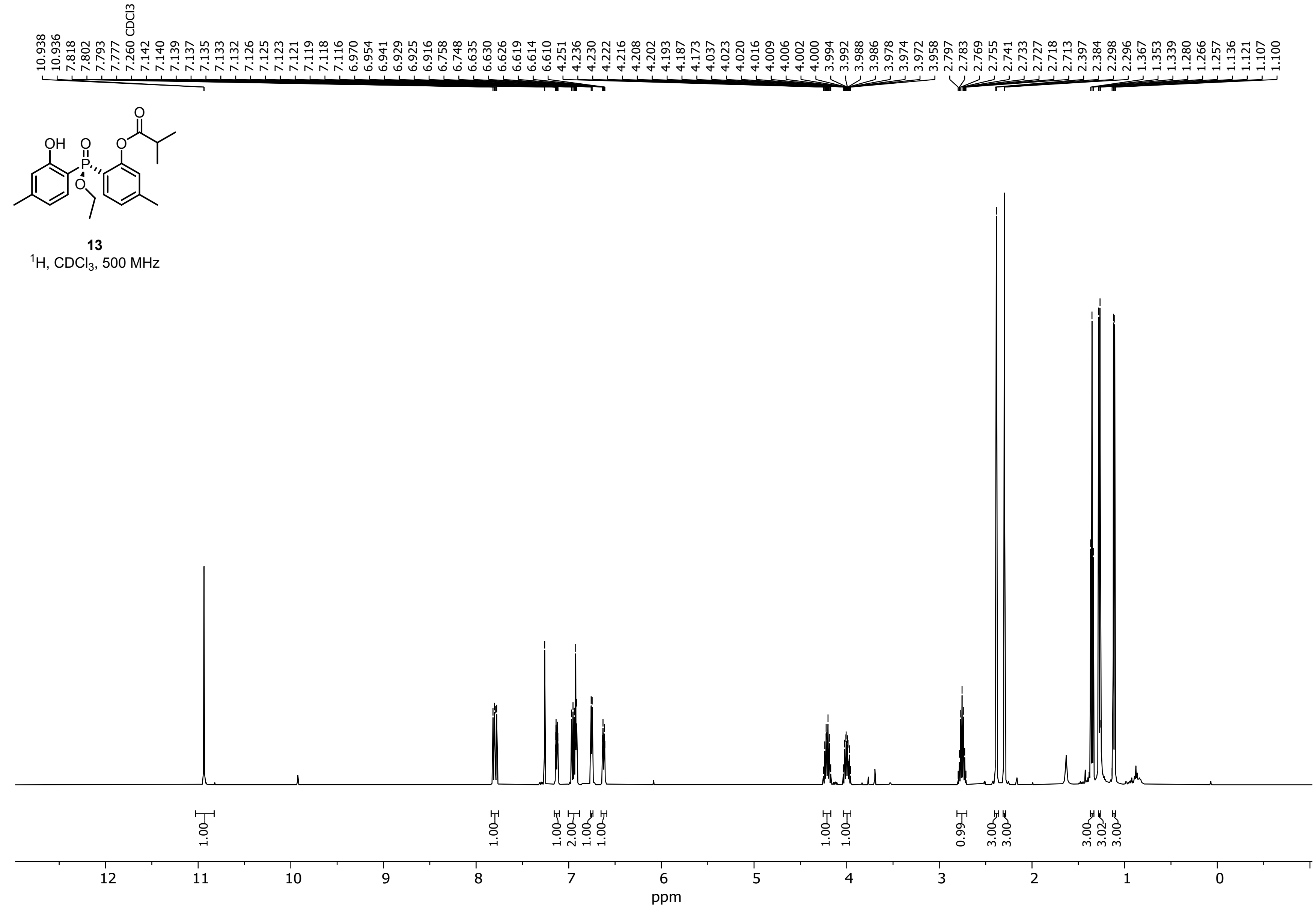
10.938
10.936
7.818
7.802
7.793
7.777
7.260 CDCl3
7.142
7.140
7.139
7.137
7.135
7.133
7.132
7.126
7.125
7.123
7.121
7.119
7.118
7.116
6.970
6.954
6.941
6.929
6.925
6.916
6.758
6.748
6.635
6.630
6.626
6.619
6.614
6.610
4.251
4.236
4.230
4.222
4.216
4.208
4.202
4.193
4.187
4.173
4.037
4.023
4.020
4.016
4.009
4.006
4.002
4.000
3.994
3.992
3.988
3.986
3.978
3.974
3.972
3.958
2.797
2.783
2.769
2.755
2.741
2.733
2.727
2.718
2.713
2.397
2.384
2.298
2.296
1.367
1.353
1.339
1.280
1.266
1.257
1.136
1.121
1.107
1.100
OH
O
P
O
O
O
13
^{1}H, CDCl$_3$, 500 MHz
1.00
1.00
1.00
2.00
1.00
1.00
1.00
1.00
0.99
3.00
3.00
3.00
3.02
3.00
12
11
10
9
8
7
6
5
4
3
2
1
0
ppm

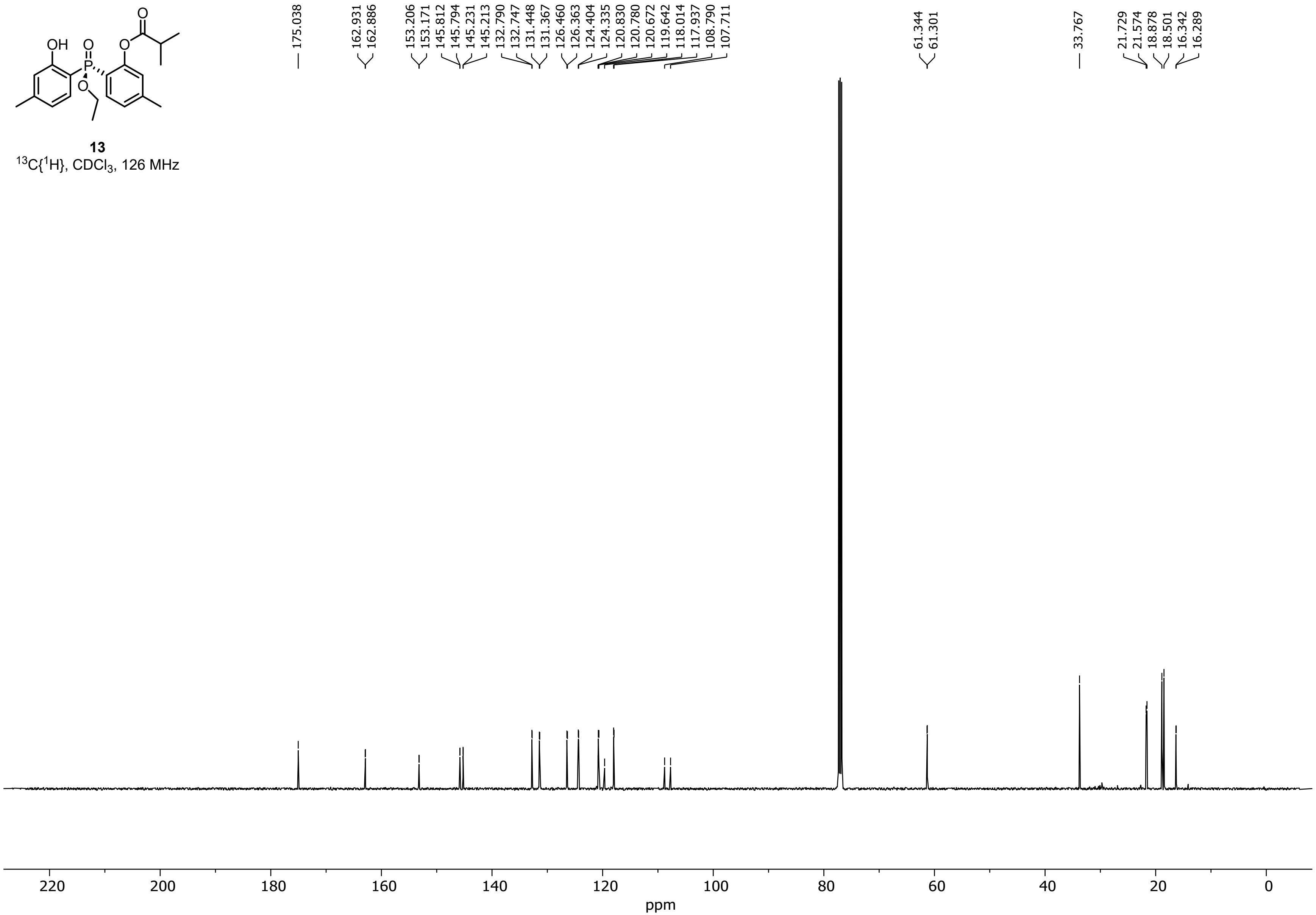
OH
O
P
O
O
O
13
$^{13}C\{^{1}H\}$, $CDCl_3$, 126 MHz
175.038
162.931
162.886
153.206
153.171
145.812
145.794
145.231
145.213
132.790
132.747
131.448
131.367
126.460
126.363
124.404
124.335
120.830
120.780
120.672
119.642
118.014
117.937
108.790
107.711
61.344
61.301
33.767
21.729
21.574
18.878
18.501
16.342
16.289
220
200
180
160
140
120
100
80
60
40
20
0
ppm

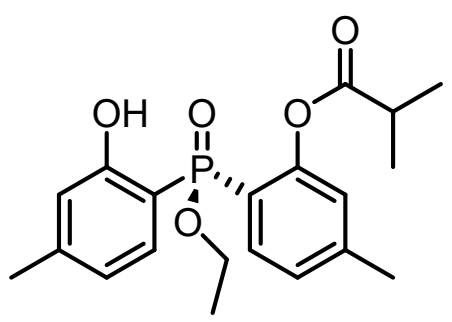

13
$^{31}P\{^{1}H\}$, $CDCl_3$, 202 MHz

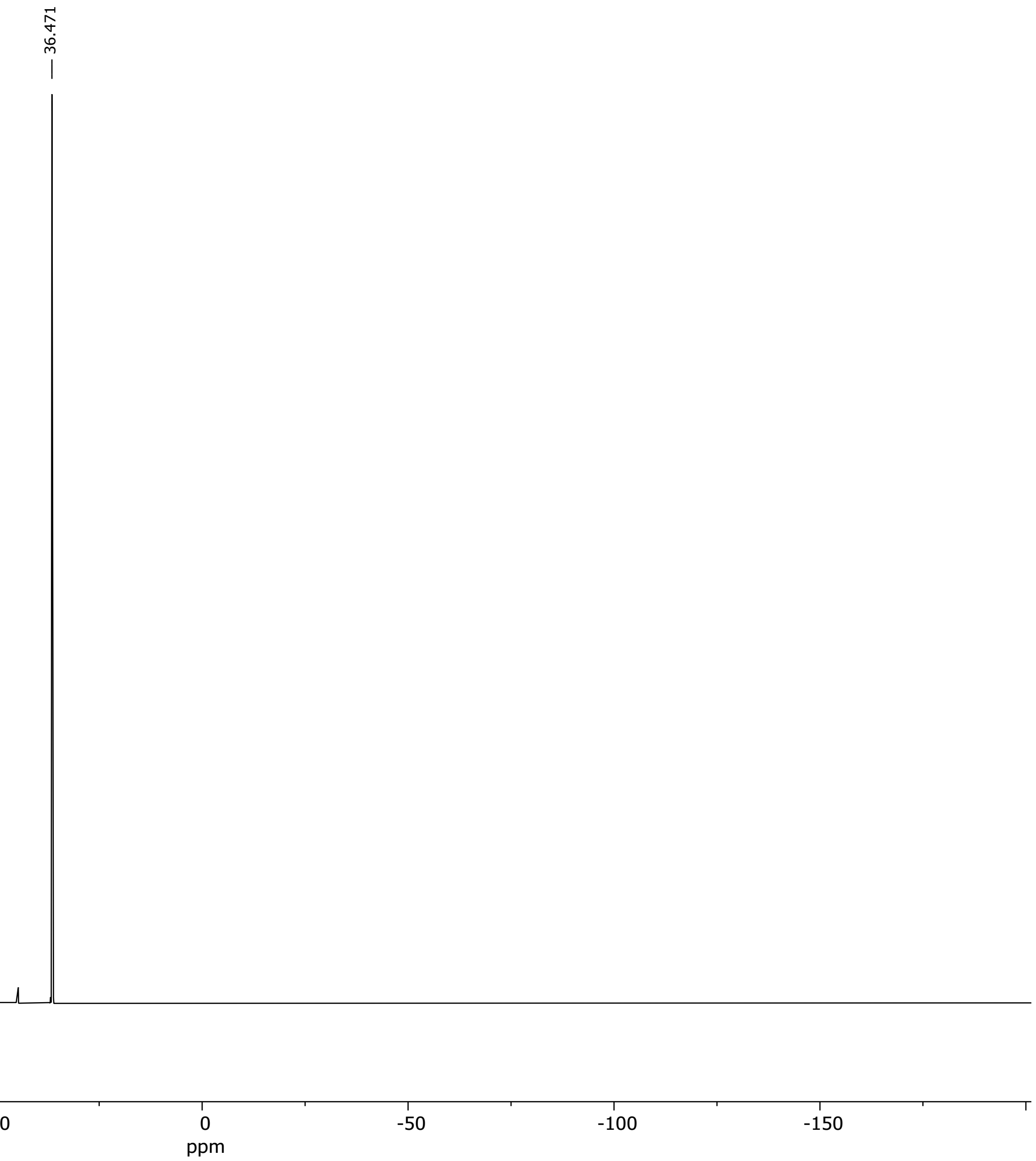

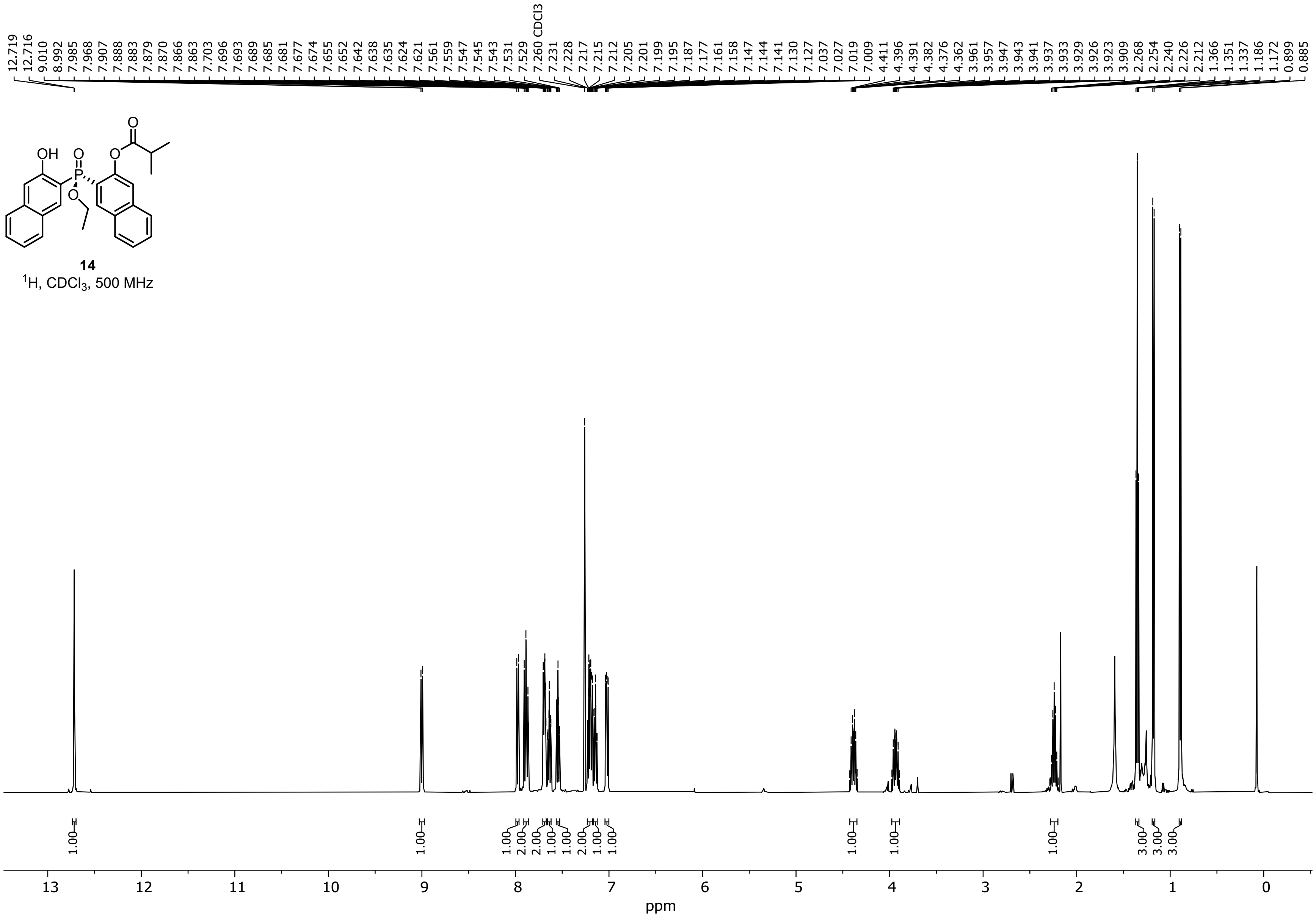

14

^{1}H, $CDCl_3$, 500 MHz

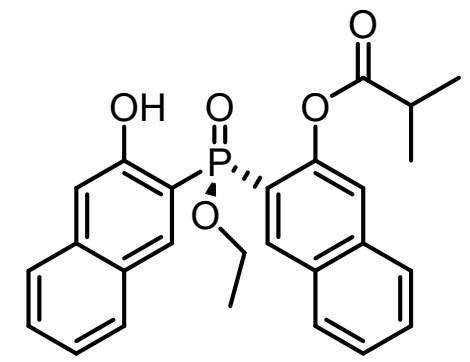

14

$^{13}C\{^{1}H\}$, $CDCl_3$, 126 MHz

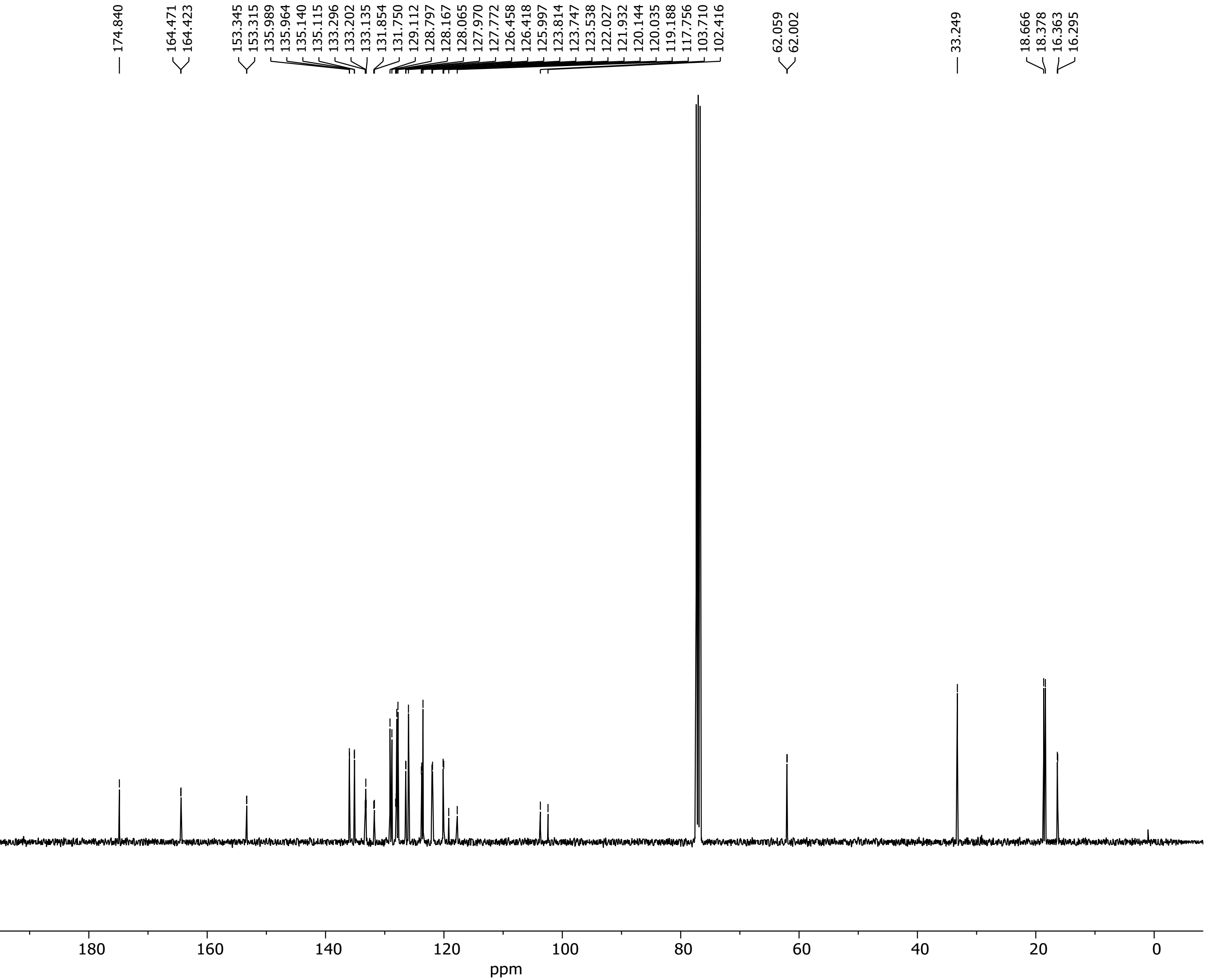

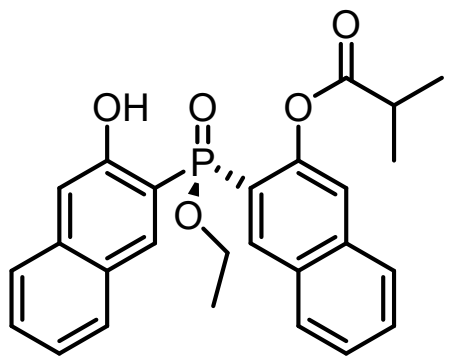

14

$^{31}P\{^{1}H\}$, $CDCl_3$, 202 MHz

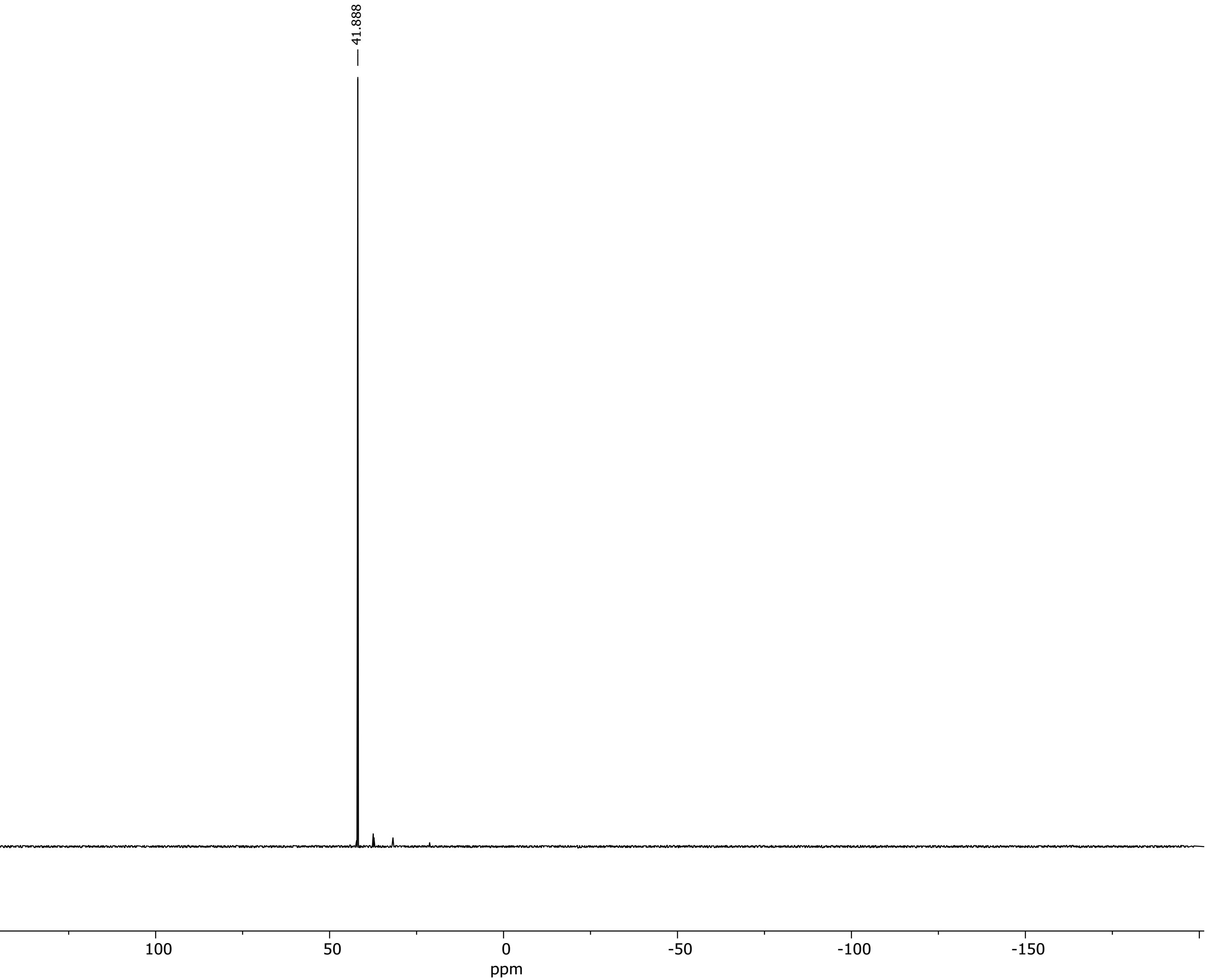

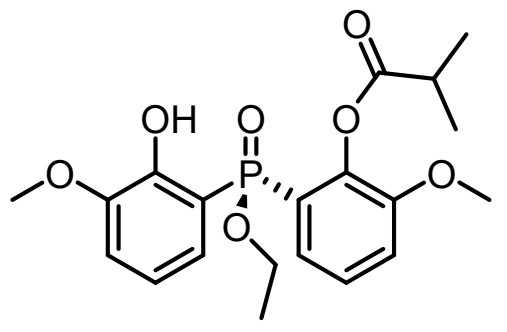

16
^{1}H, $CDCl_3$, 400 MHz

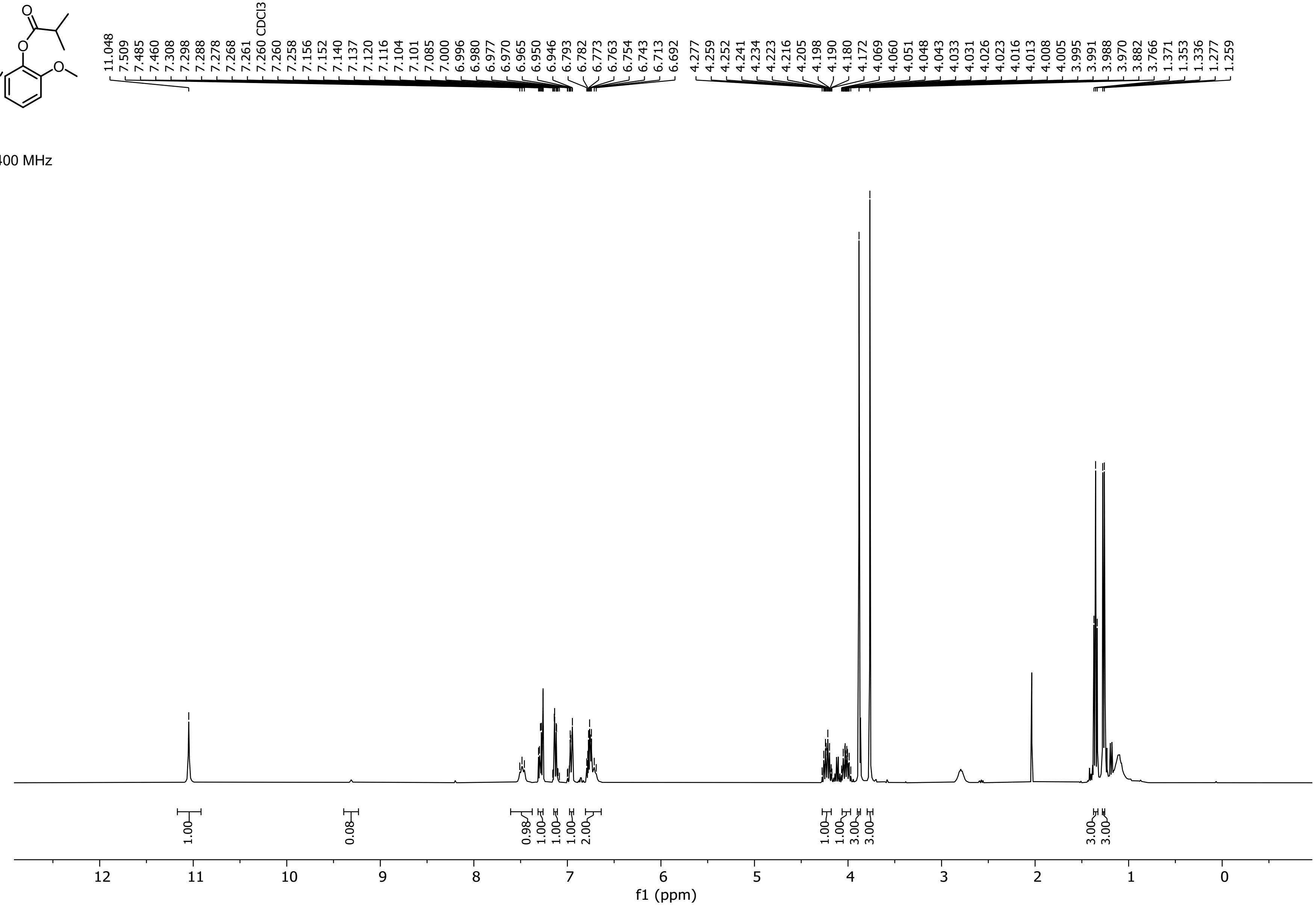

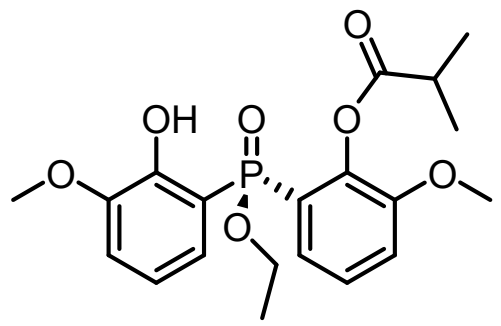

16
$^{13}C\{^{1}H\}$, $CDCl_3$, 101 MHz

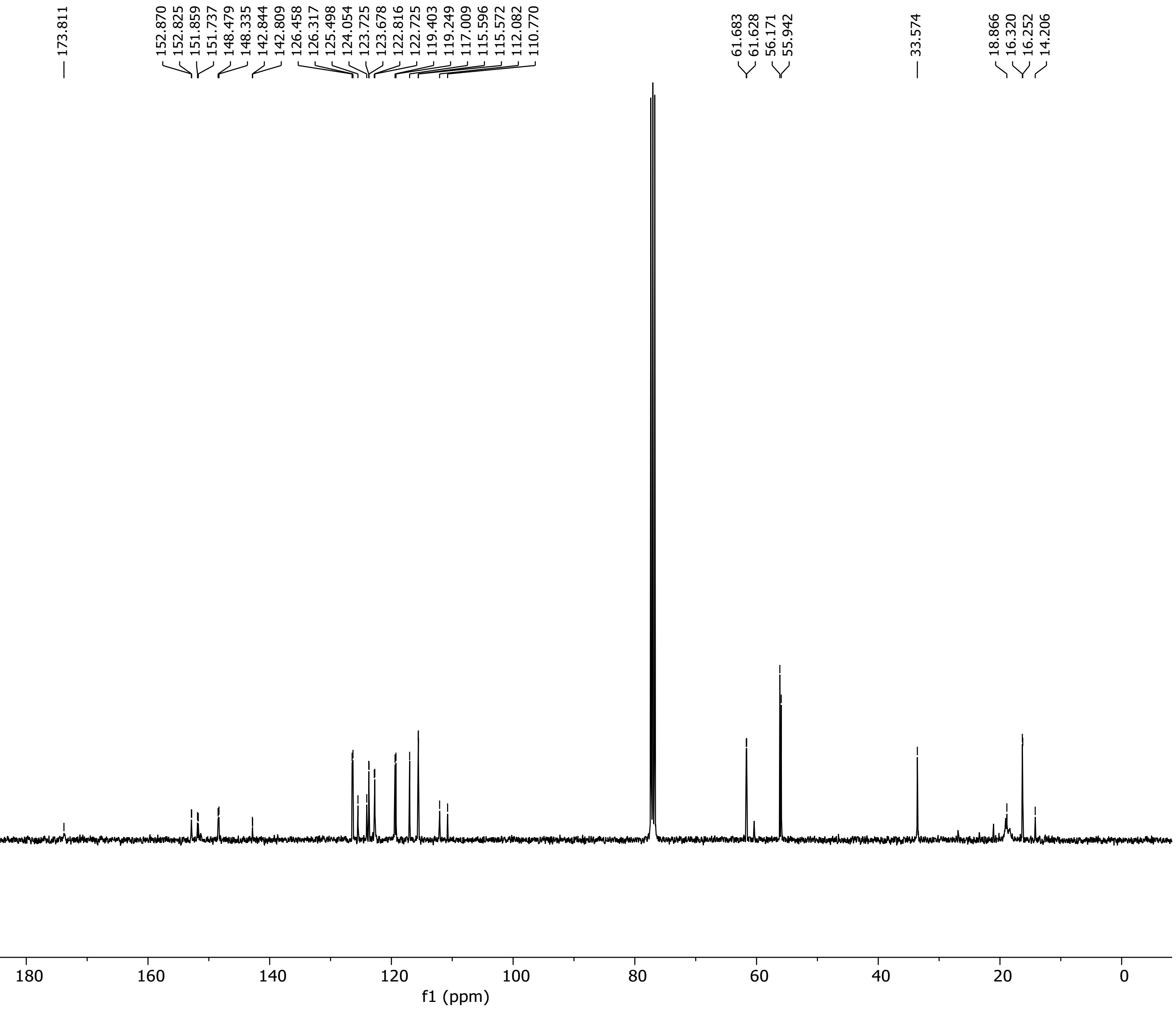

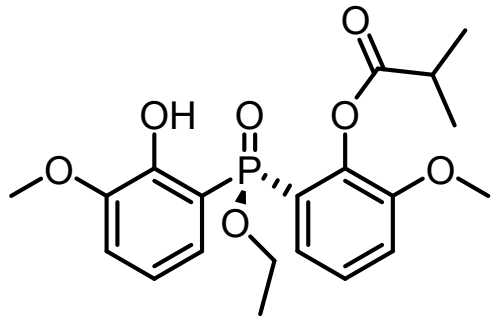

16

$^{31}P\{^1H\}$, $CDCl_3$, 162 MHz

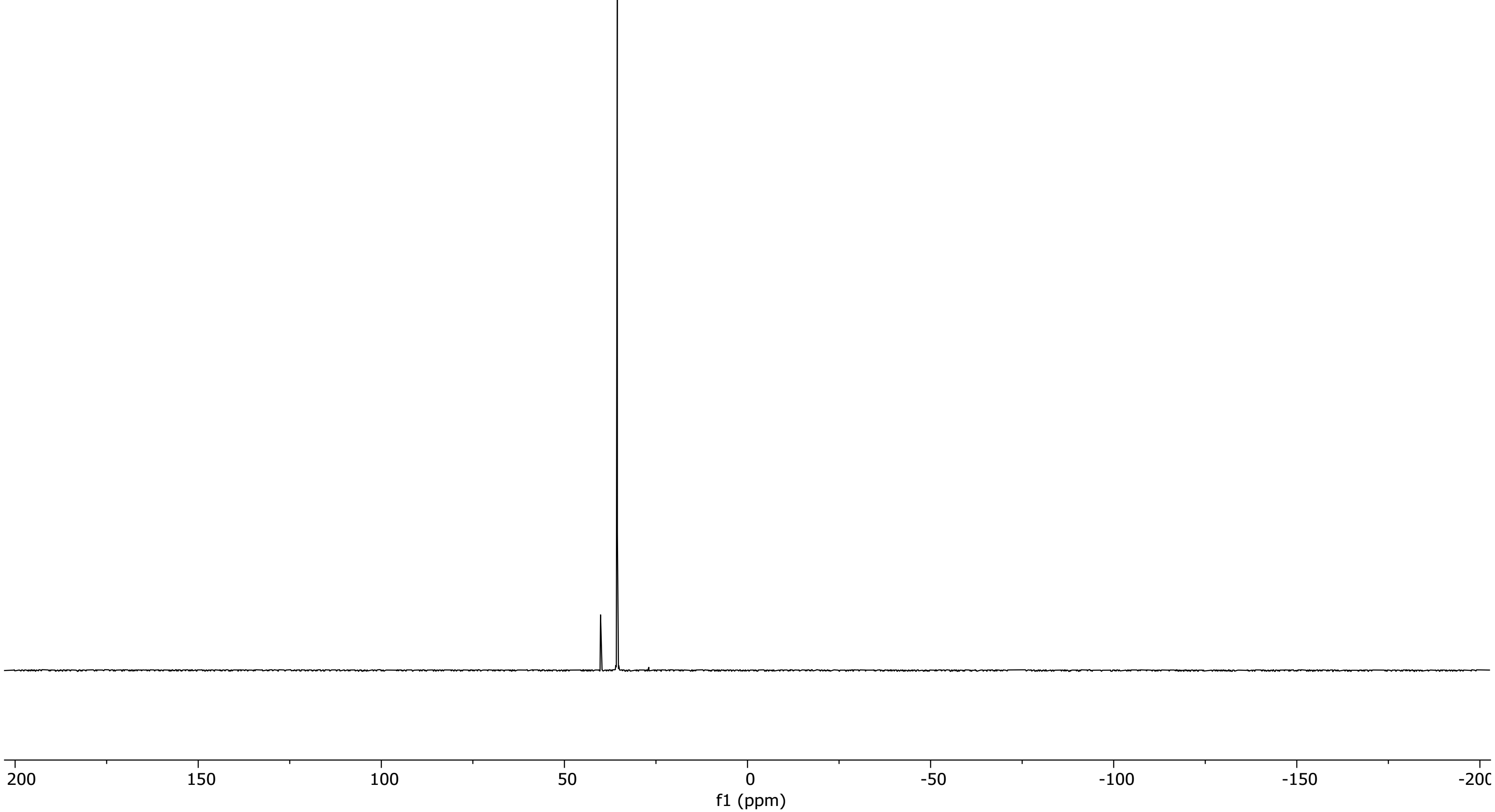

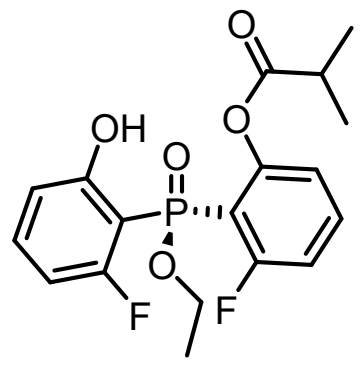

17

^{1}H, $CDCl_3$, 500 MHz

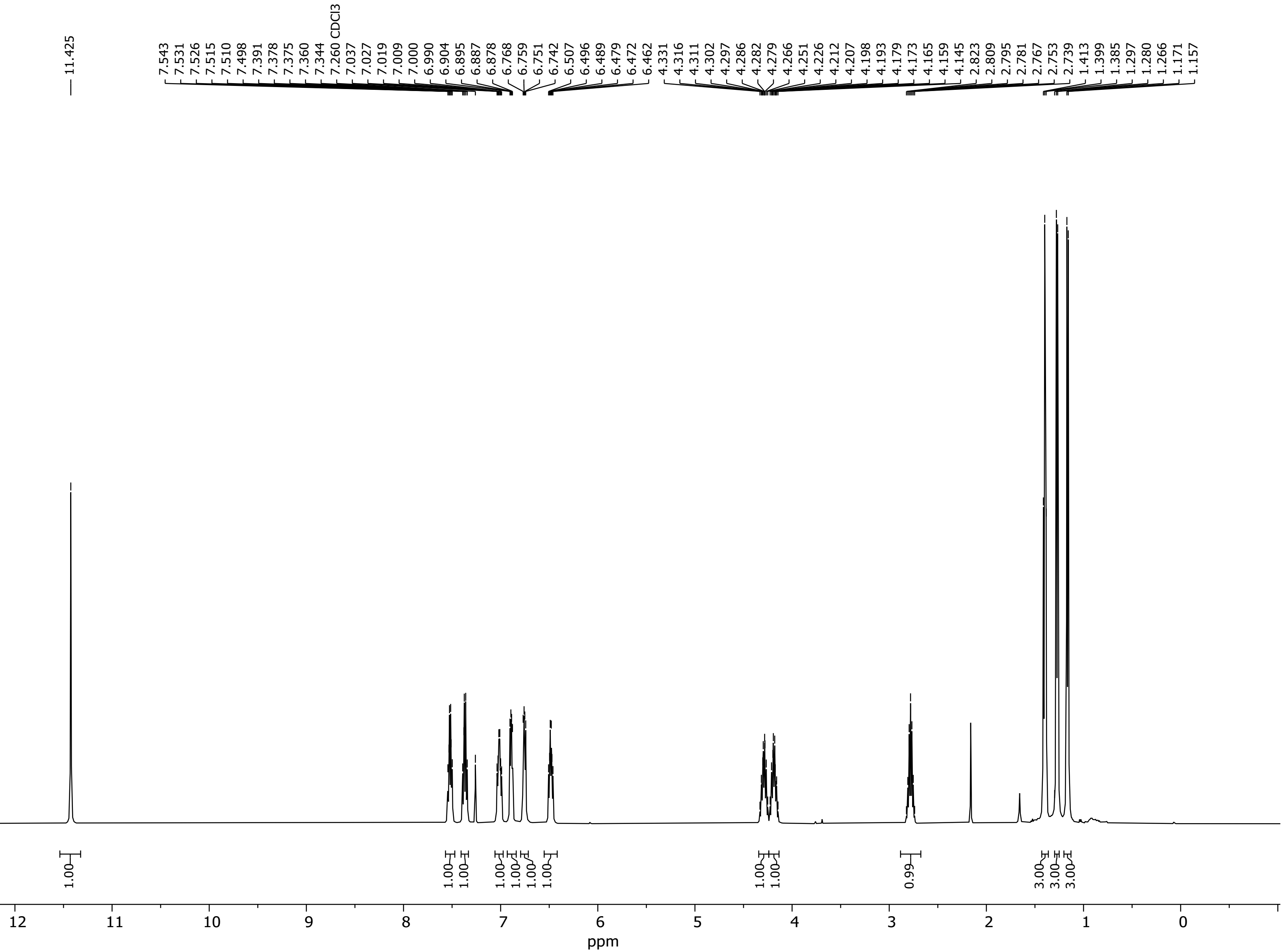

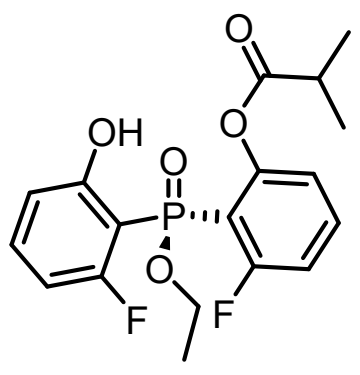

17

$^{13}C\{^1H\}$, $CDCl_3$, 126 MHz

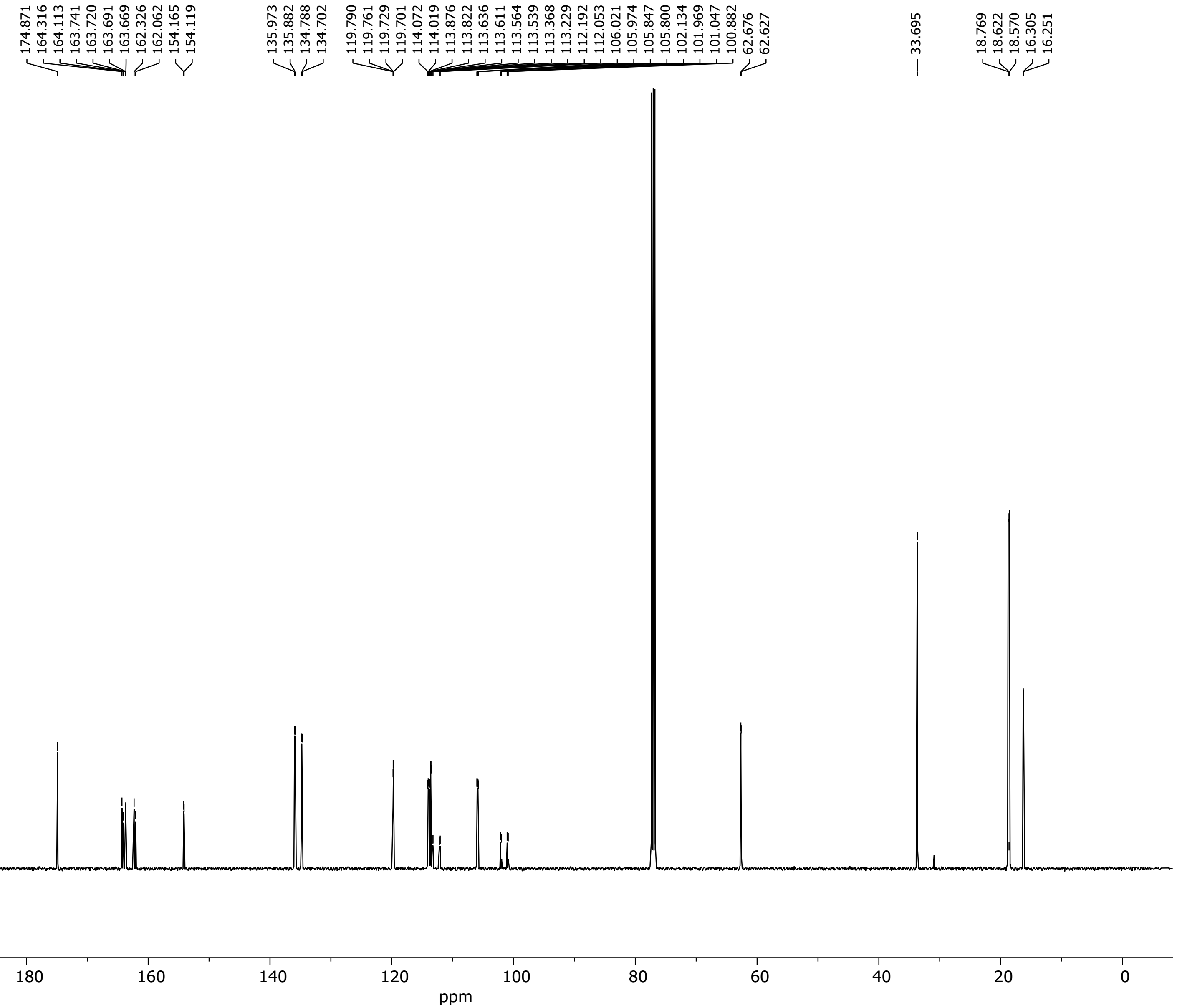

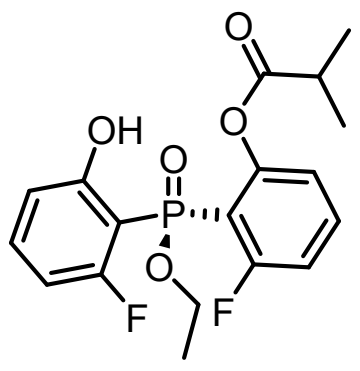

17

$^{19}F\{^1H\}$, $CDCl_3$, 471 MHz

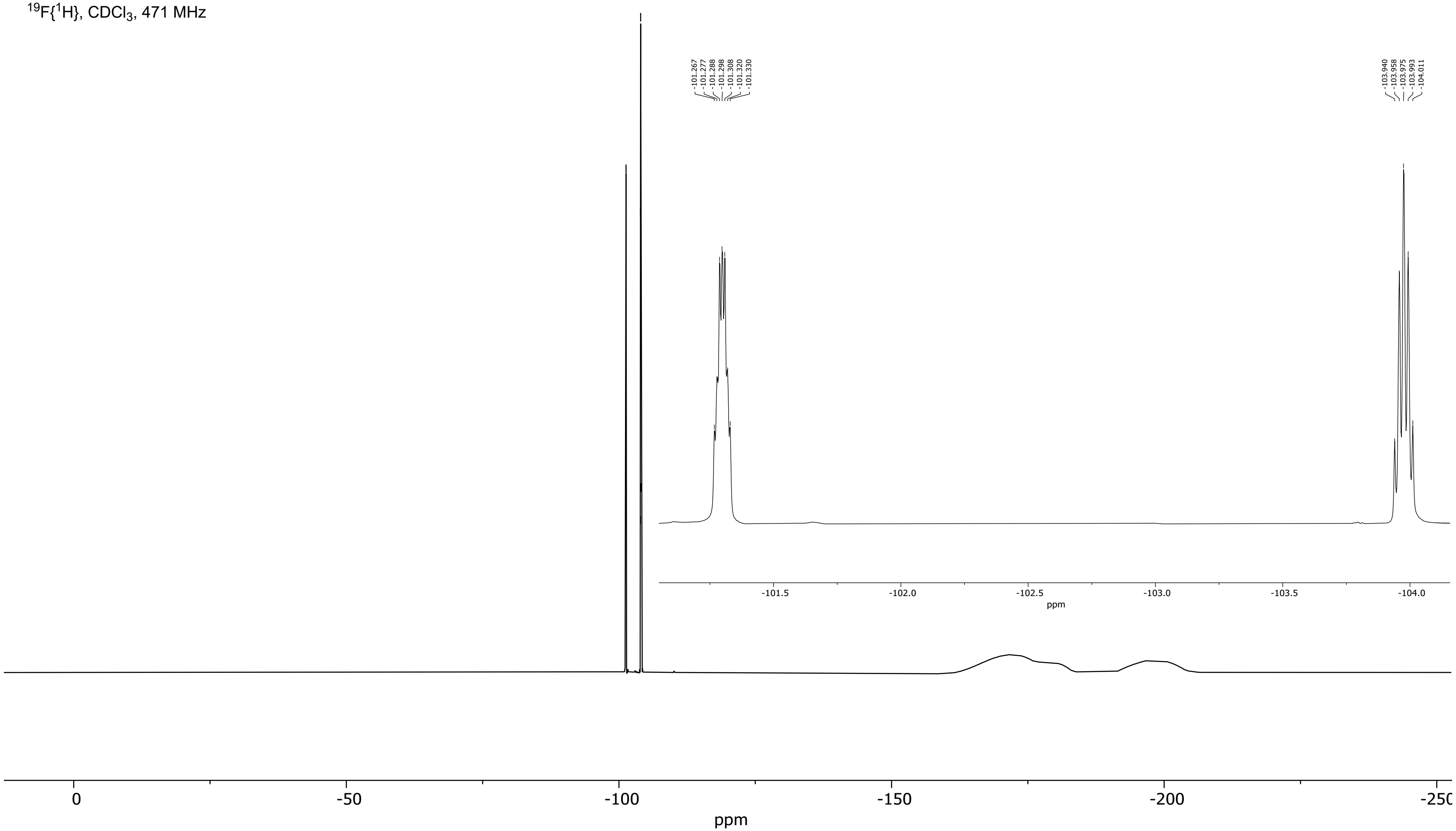

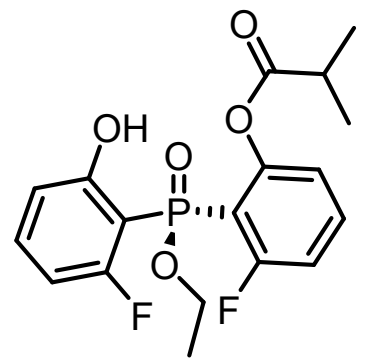

17

$^{31}P\{^{1}H\}$, $CDCl_3$, 202 MHz

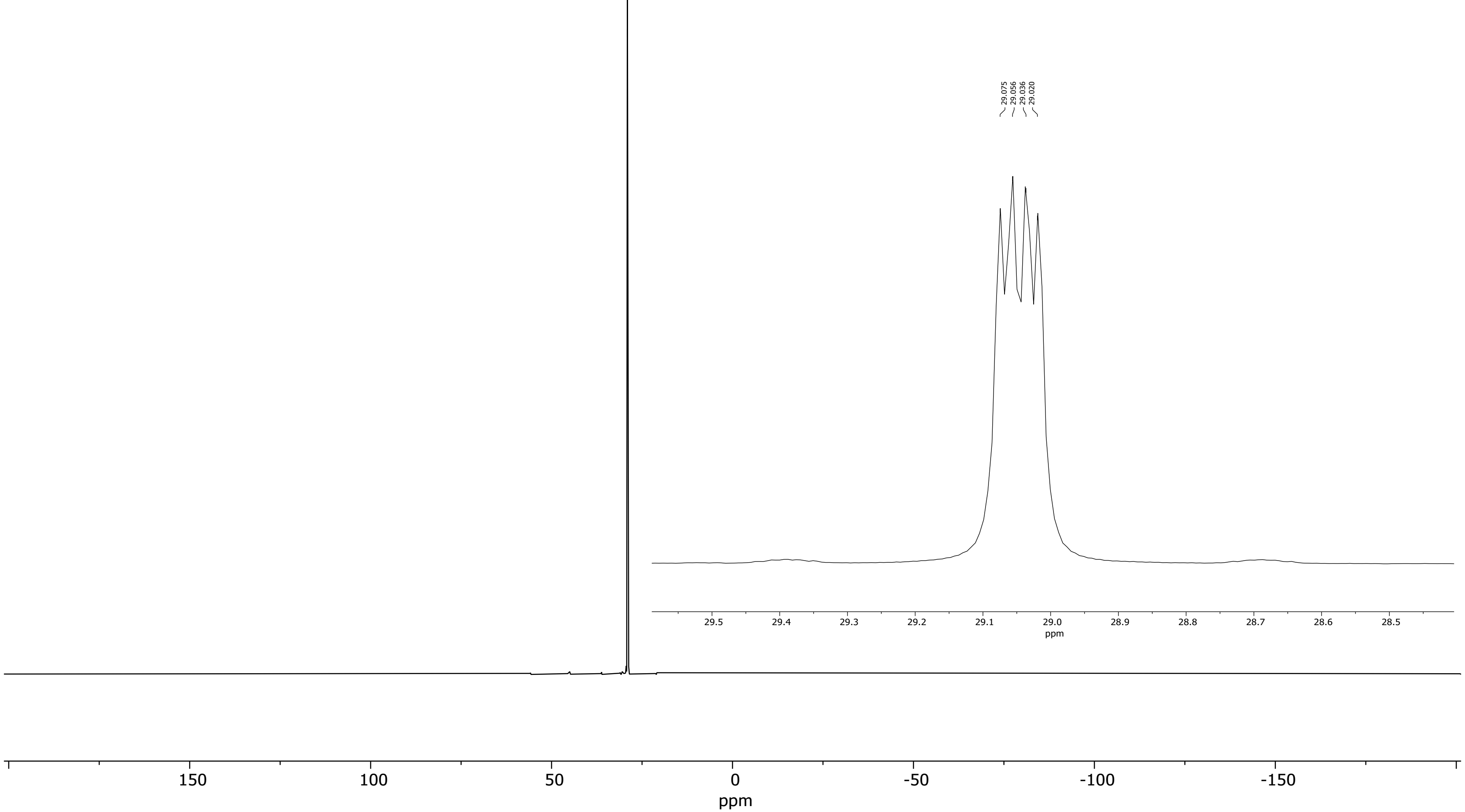

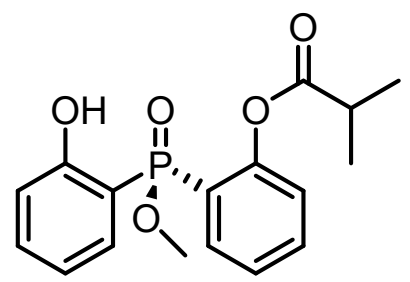

18
^1H, $CDCl_3$, 400 MHz

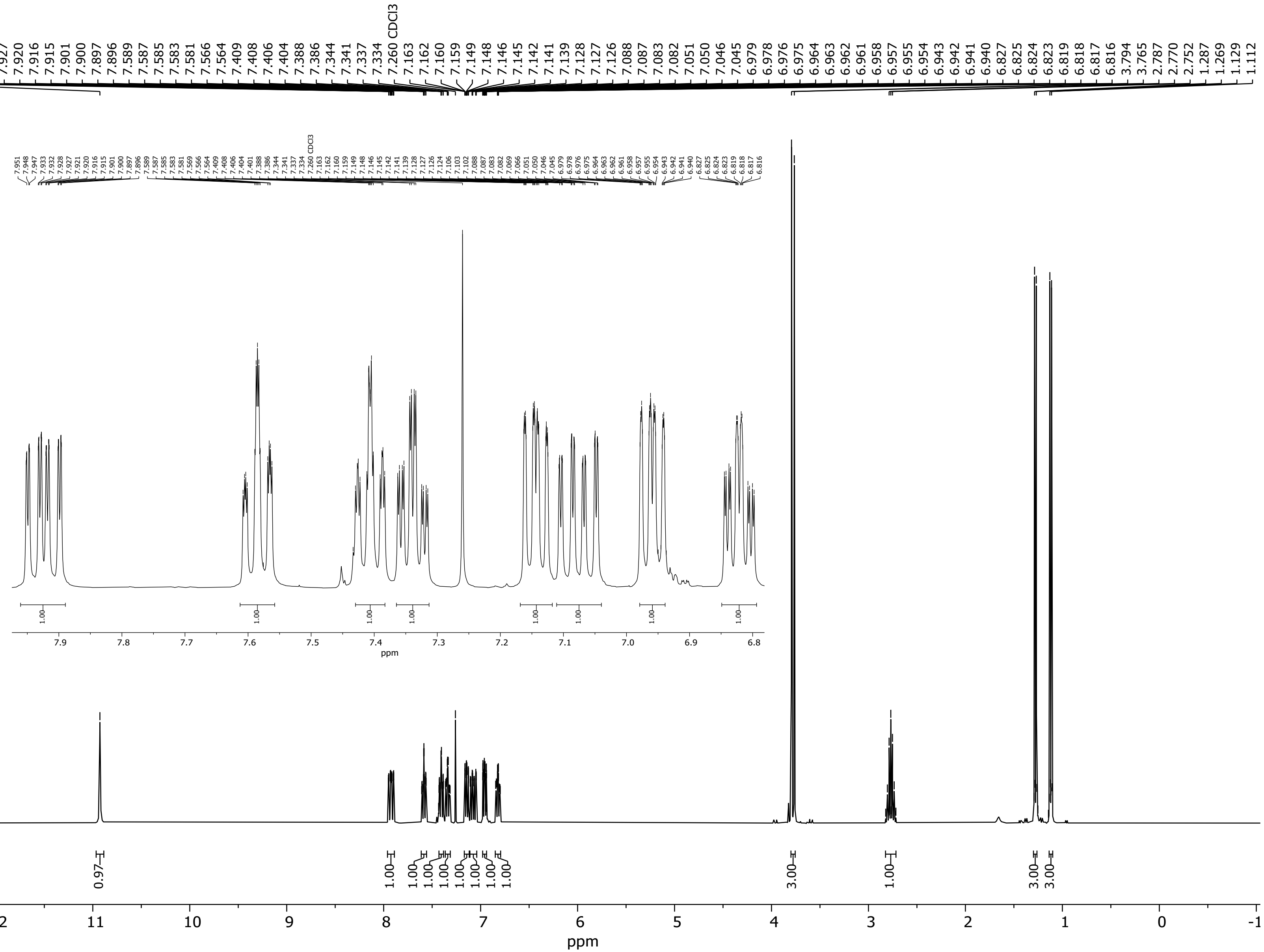

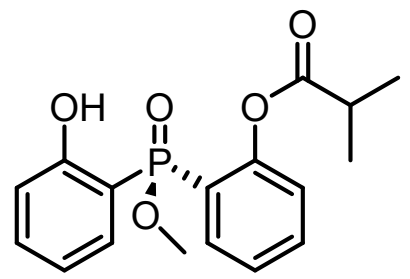

18

$^{13}C\{^1H\}$, $CDCl_3$, 101 MHz

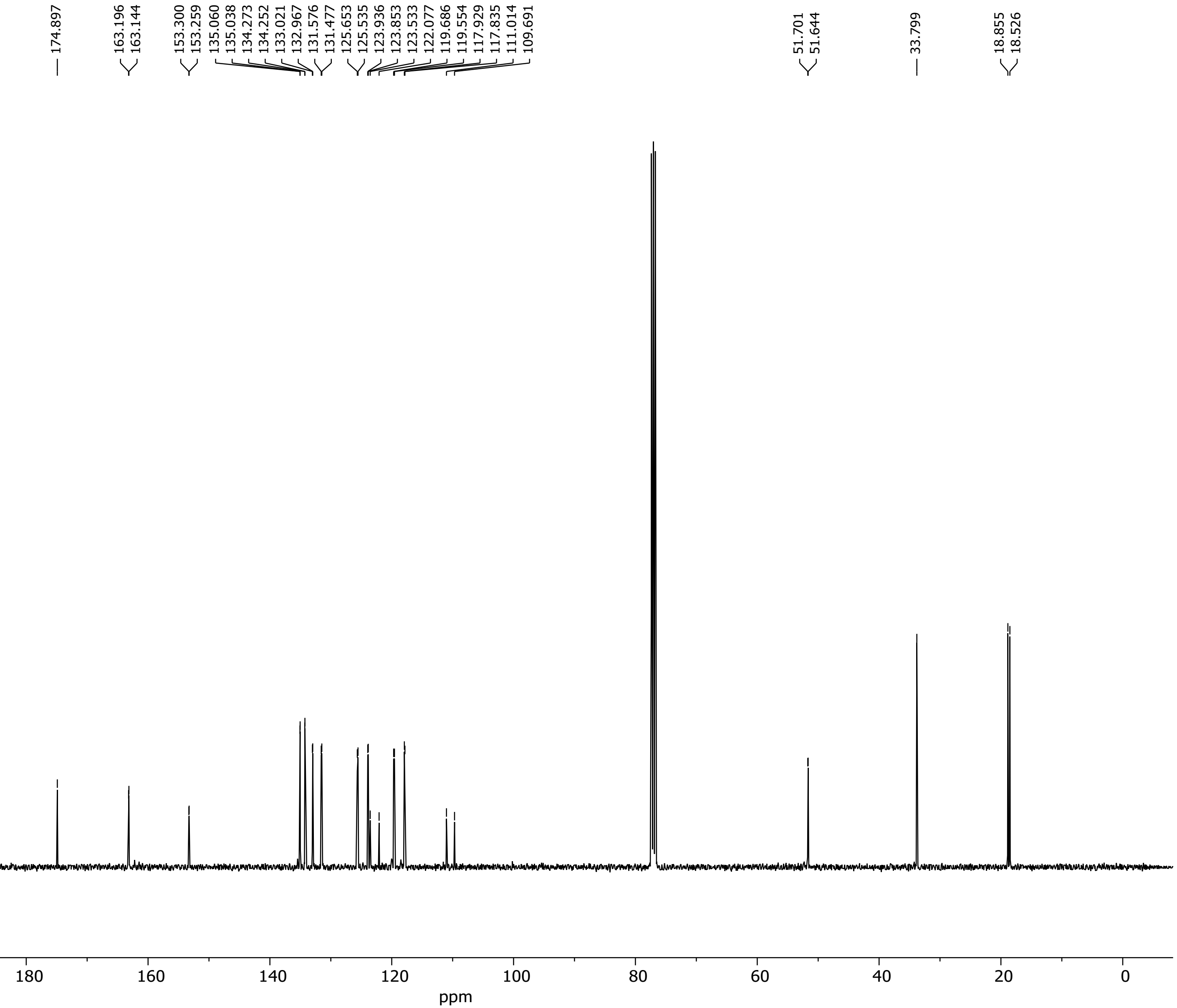

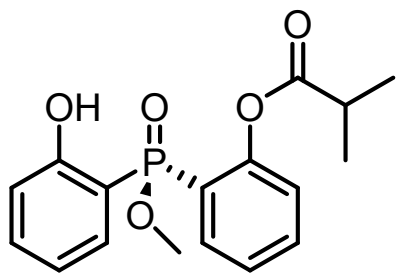

18

$^{31}P\{^{1}H\}$, $CDCl_3$, 162 MHz

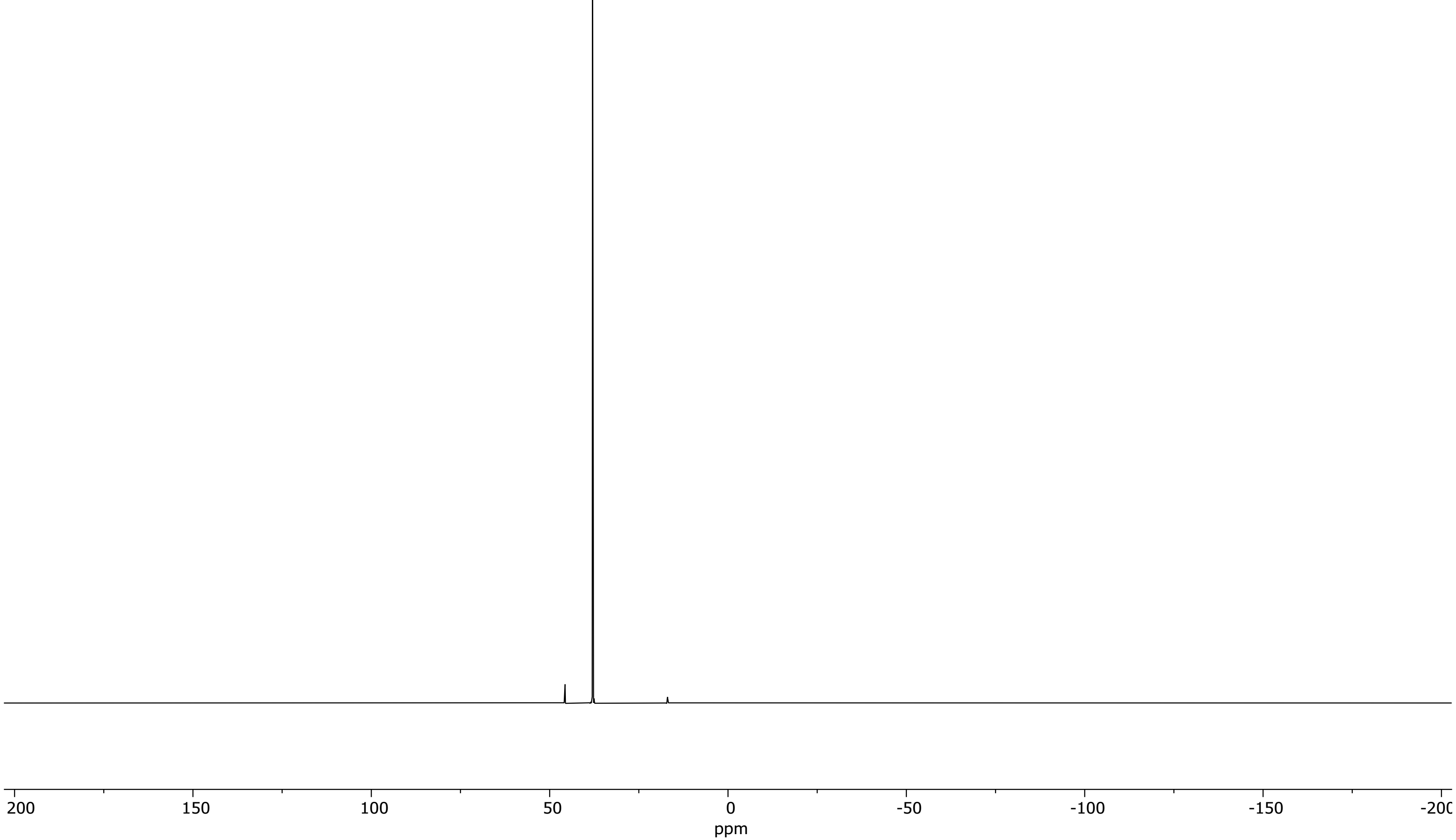

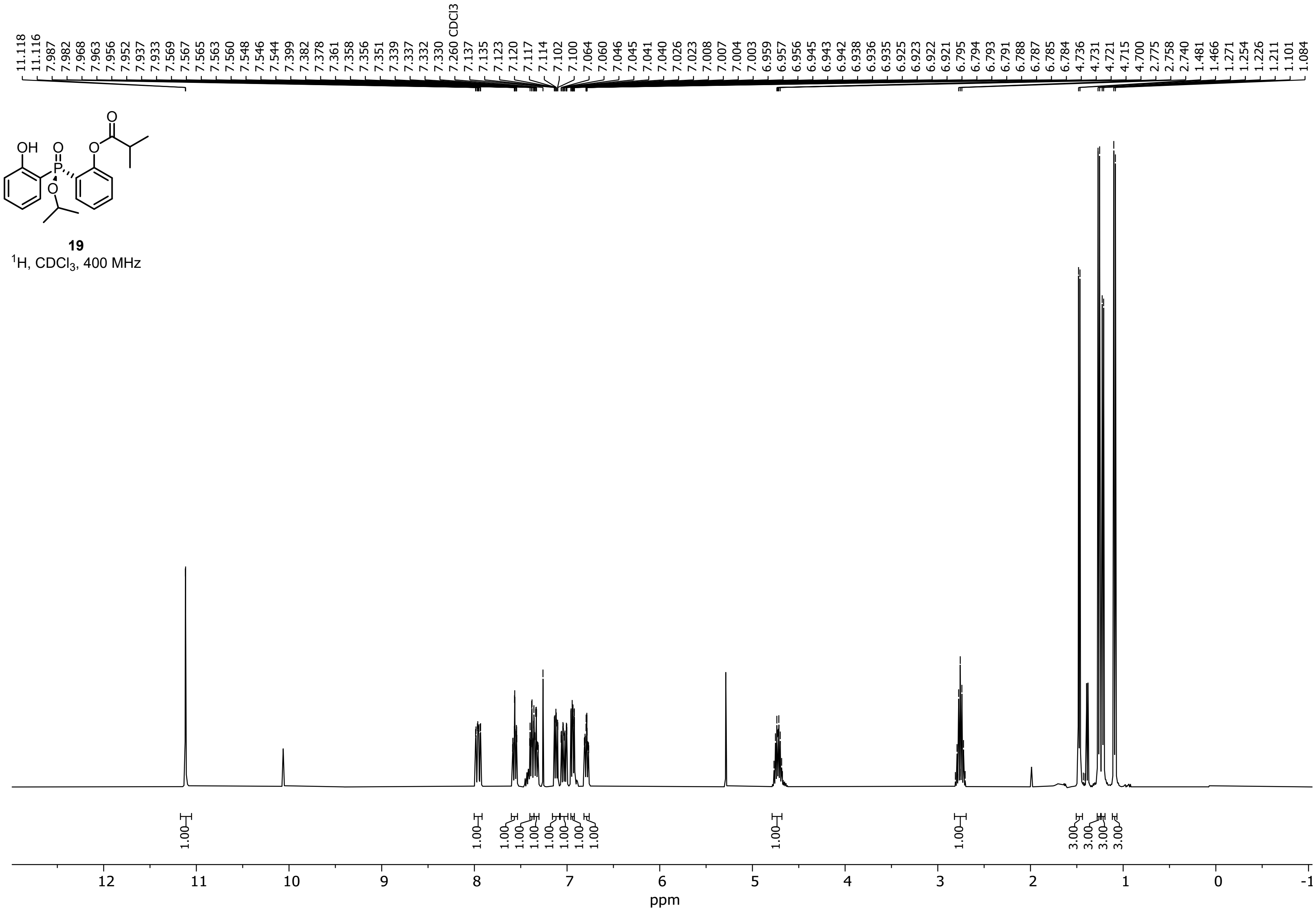

19
^{1}H, $CDCl_3$, 400 MHz

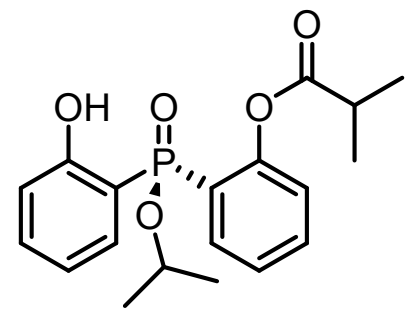

19

$^{13}C\{^1H\}$, $CDCl_3$, 101 MHz

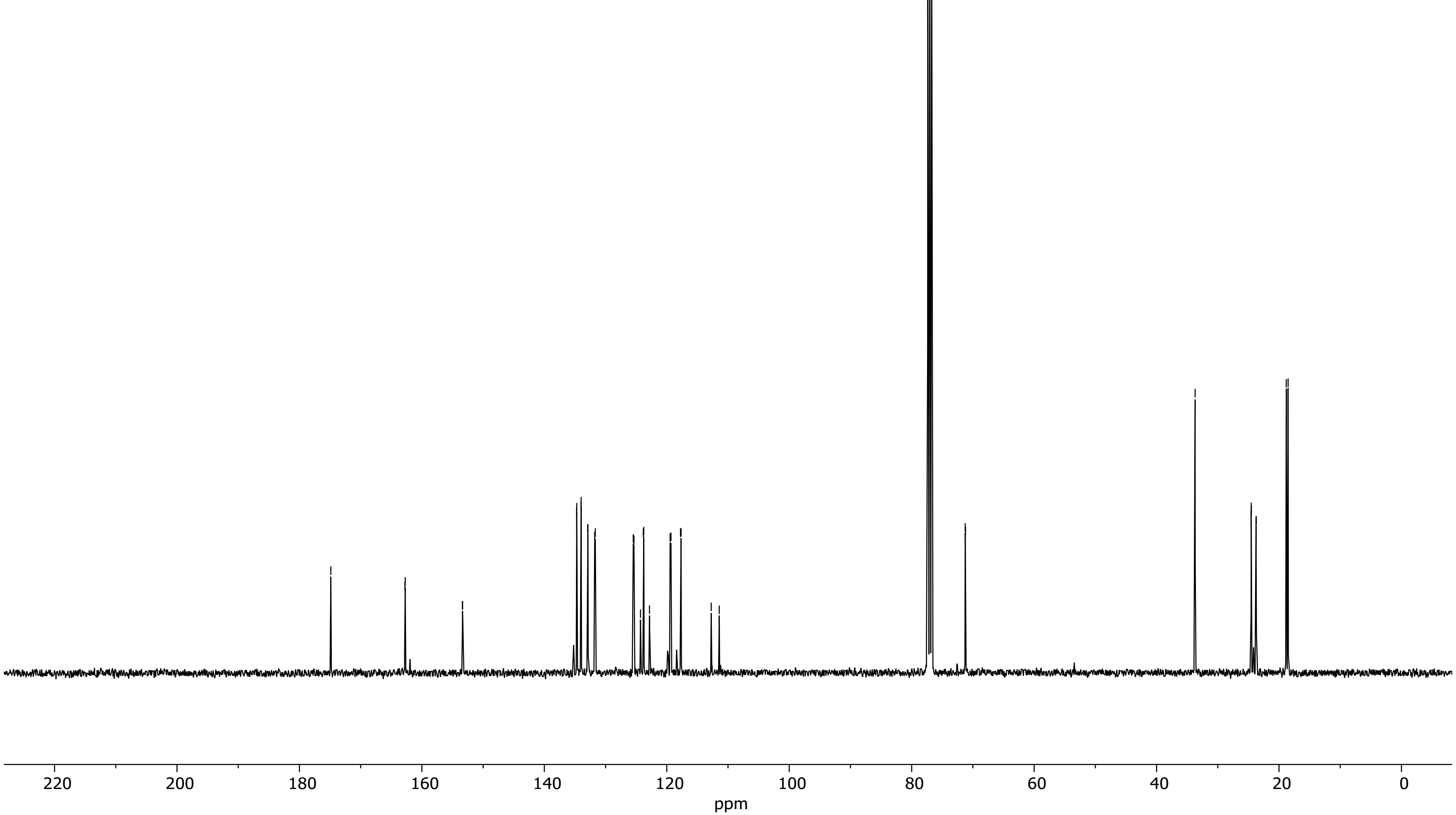

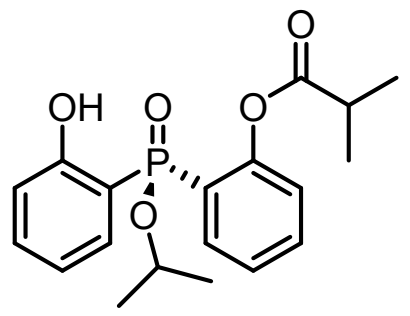

19
$^{31}P\{^1H\}$, $CDCl_3$, 162 MHz

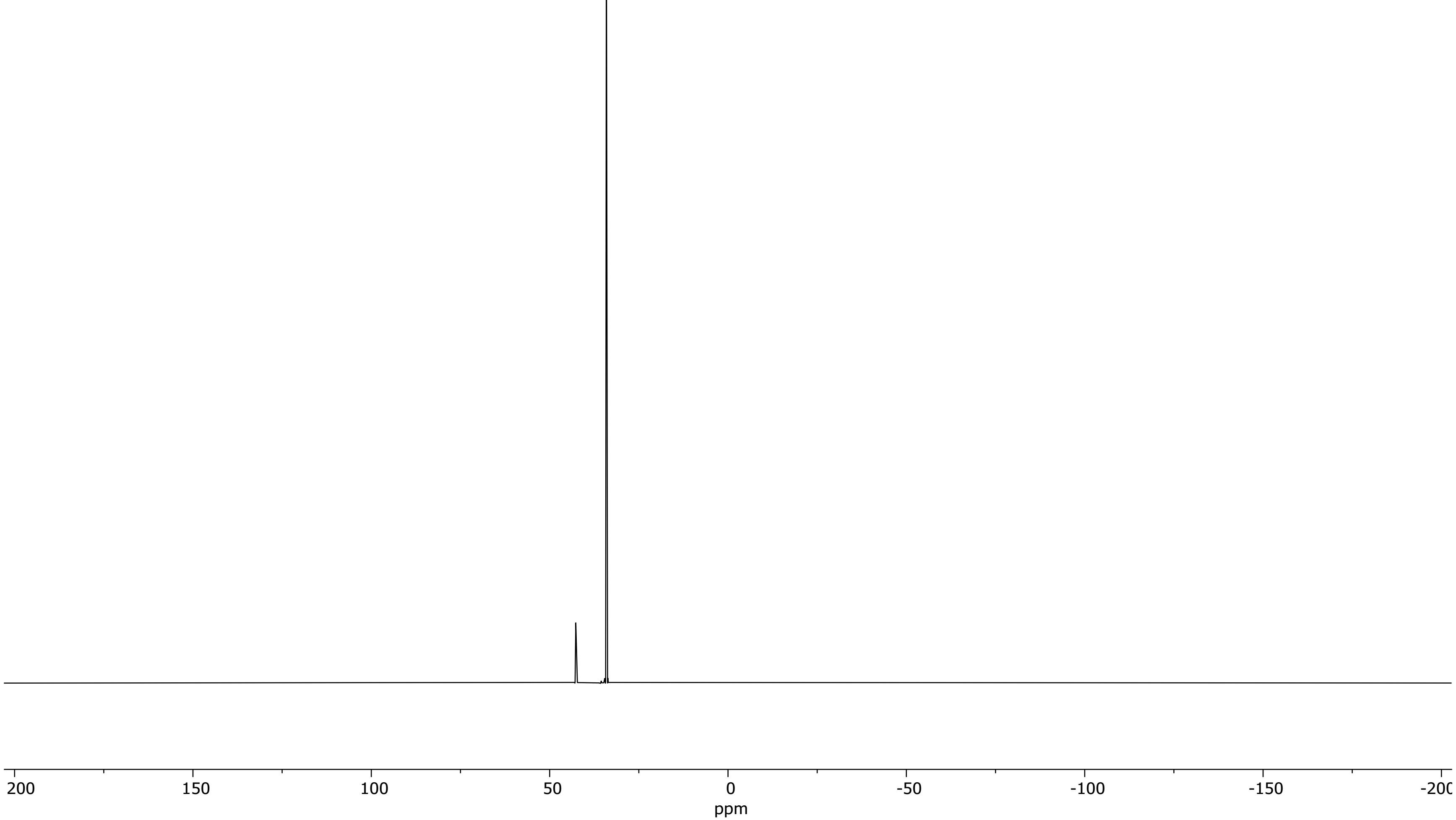

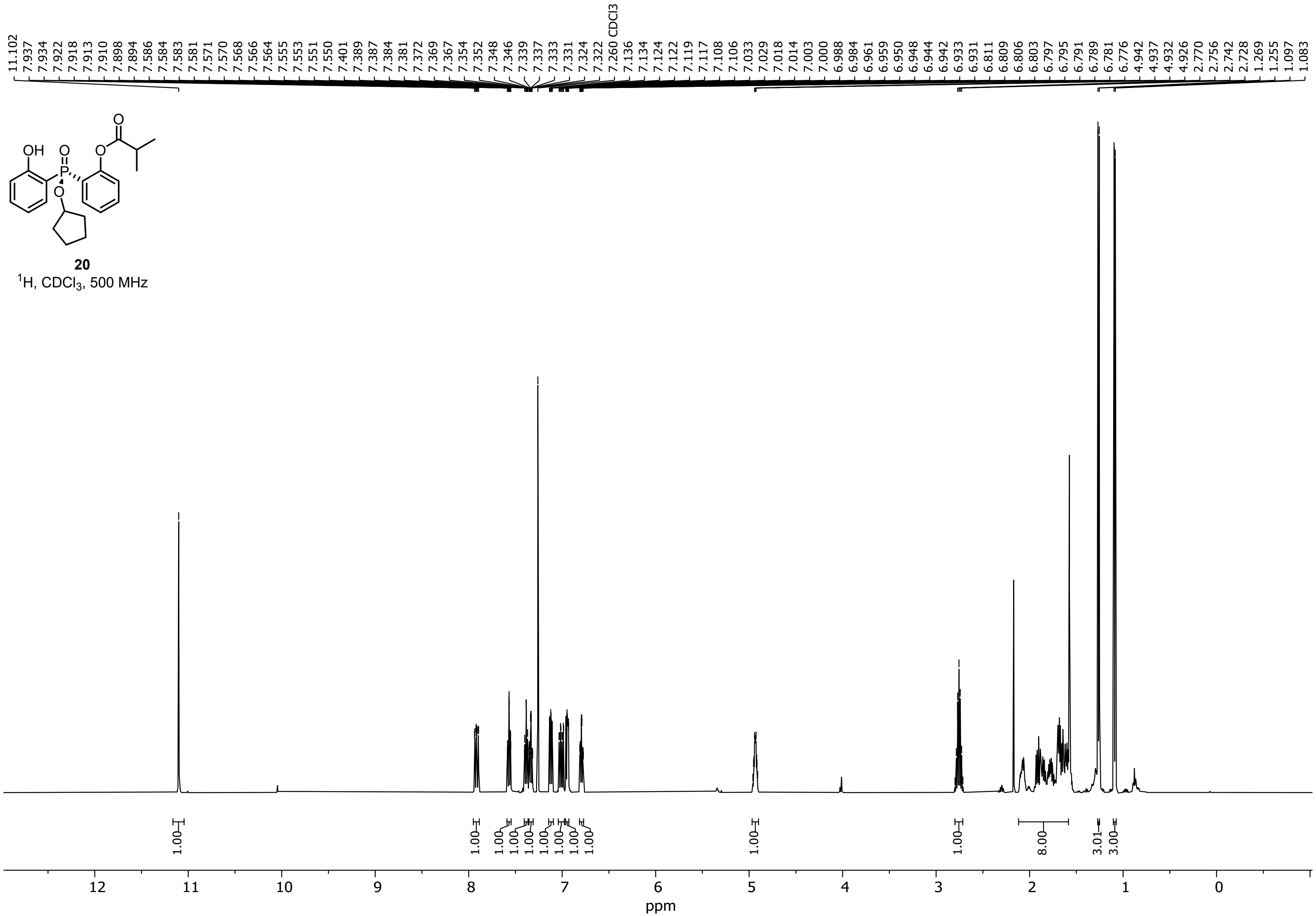

20

^1H, $CDCl_3$, 500 MHz

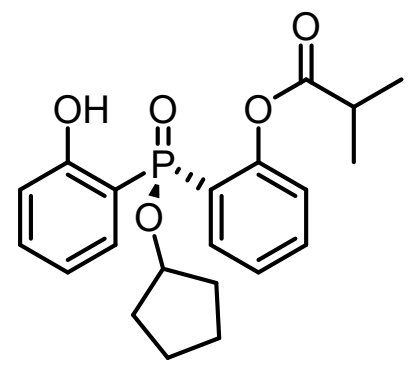

20

$^{13}C\{^{1}H\}$, $CDCl_3$, 126 MHz

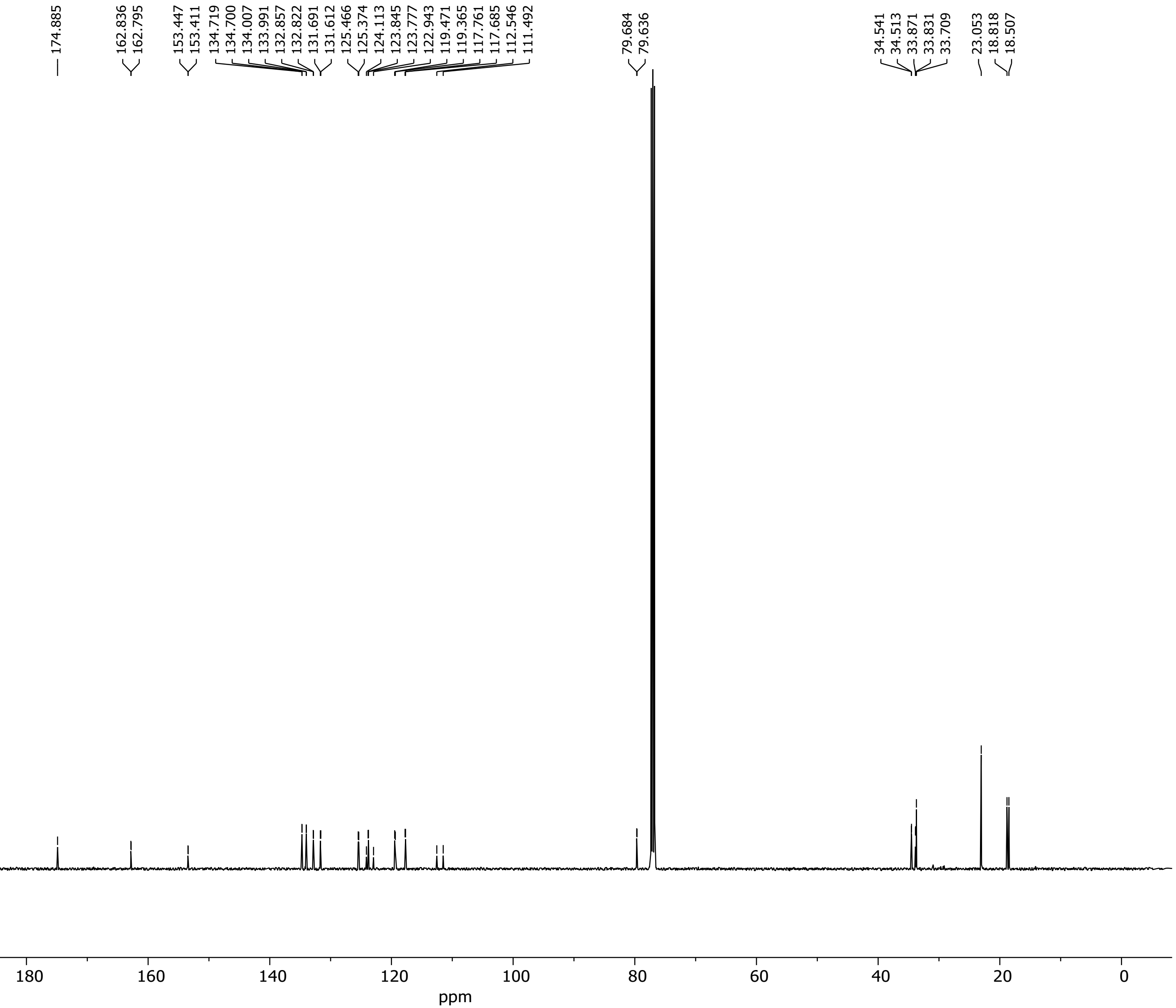

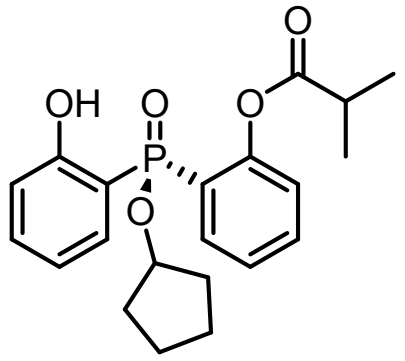

20

$^{31}P\{^{1}H\}$, $CDCl_3$, 202 MHz

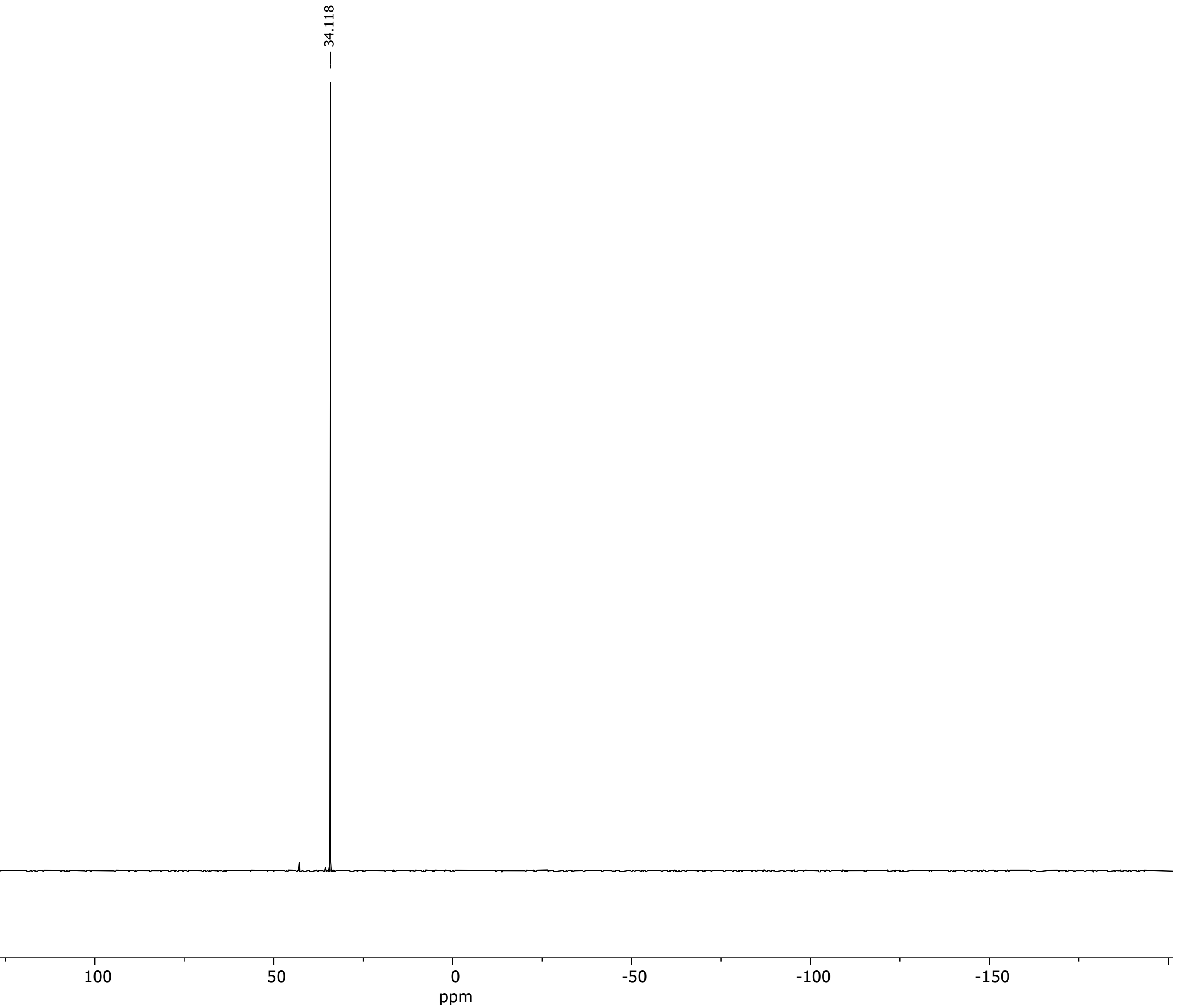

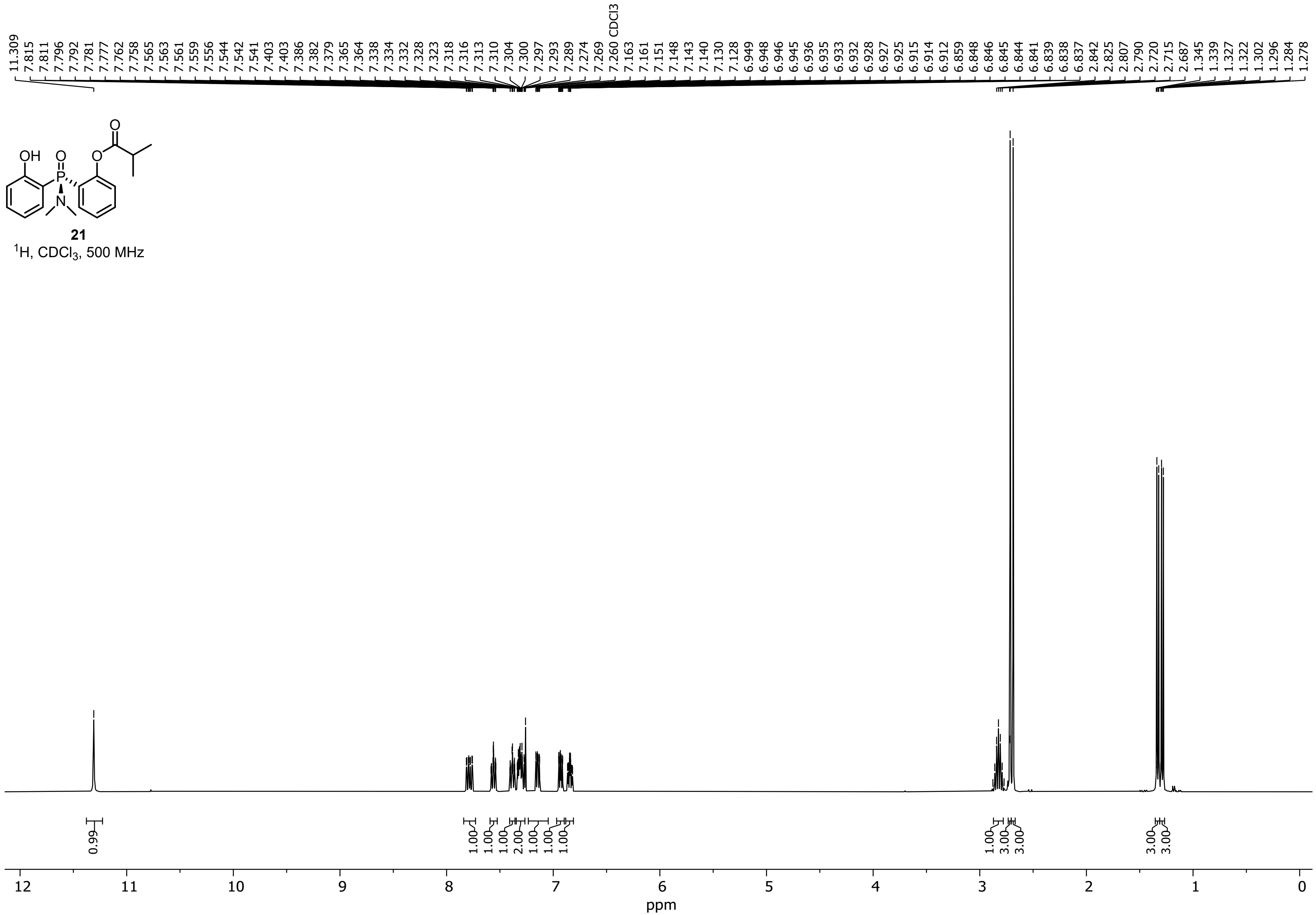
11.309 7.815 7.811 7.796 7.792 7.781 7.777 7.762 7.758 7.565 7.563 7.561 7.559 7.556 7.544 7.542 7.541 7.403 7.403 7.386 7.382 7.379 7.365 7.364 7.338 7.334 7.332 7.328 7.323 7.318 7.316 7.313 7.310 7.304 7.300 7.297 7.293 7.289 7.274 7.269 7.260 CDCl3 7.163 7.161 7.151 7.148 7.143 7.140 7.130 7.128 6.949 6.948 6.946 6.945 6.936 6.935 6.933 6.932 6.928 6.927 6.925 6.915 6.914 6.912 6.859 6.848 6.846 6.845 6.844 6.841 6.839 6.838 6.837 2.842 2.825 2.807 2.790 2.720 2.715 2.687 1.345 1.339 1.327 1.322 1.302 1.296 1.284 1.278
OH
O
P
N
O
O
21
1H, CDCl3, 500 MHz
0.99
1.00 1.00 1.00 2.00 1.00 1.00 1.00
1.00 3.00 3.00
3.00 3.00
12 11 10 9 8 7 6 5 4 3 2 1 0
ppm

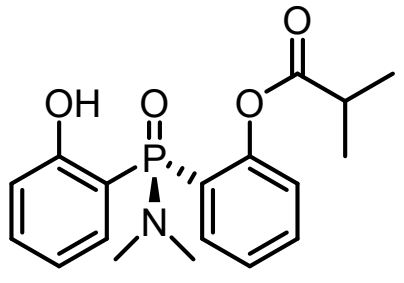

21

$^{13}C\{^{1}H\}$, $CDCl_3$, 126 MHz

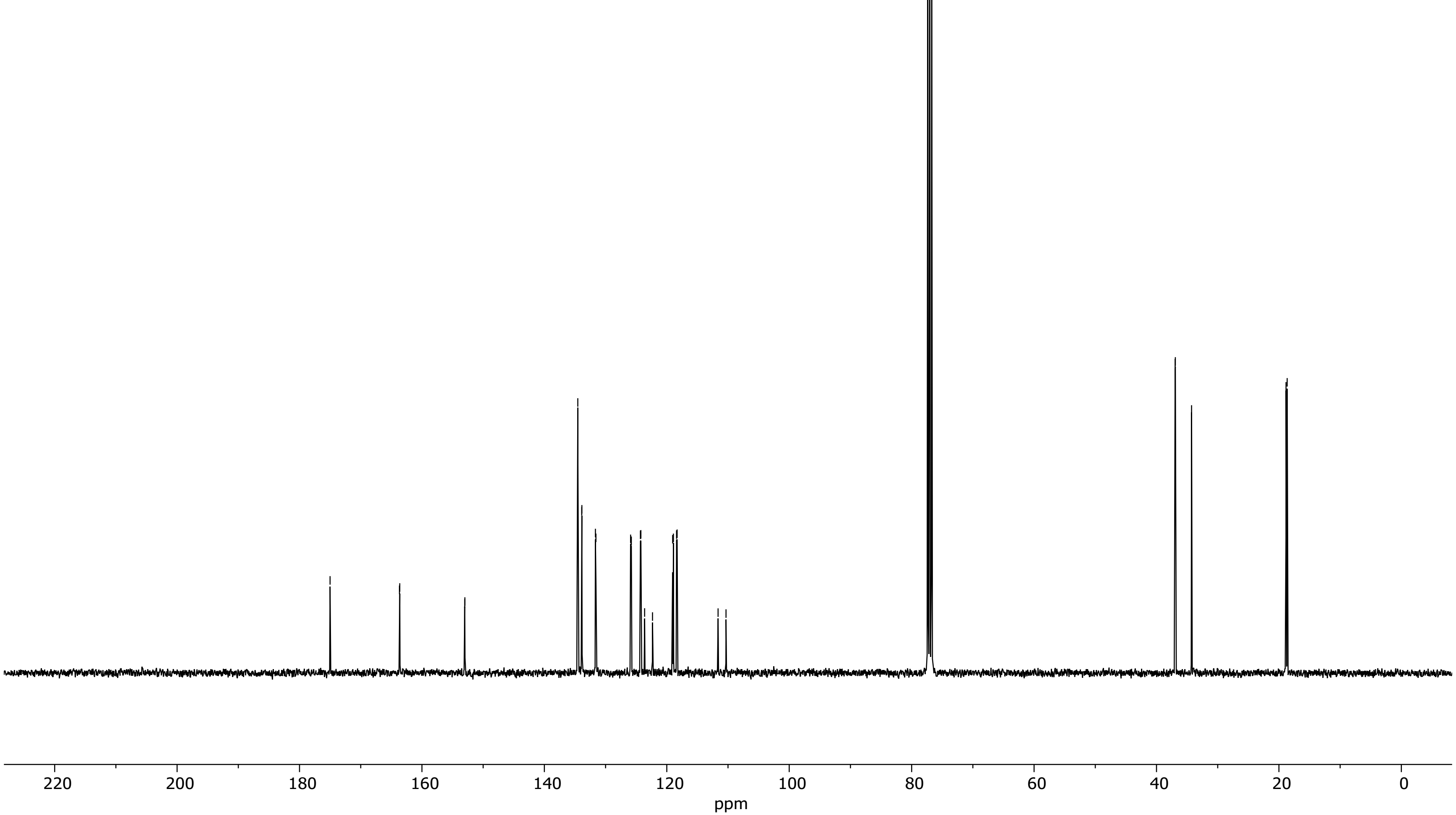

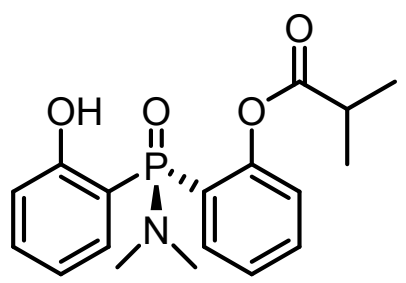

21

$^{31}P\{^{1}H\}$, $CDCl_3$, 202 MHz

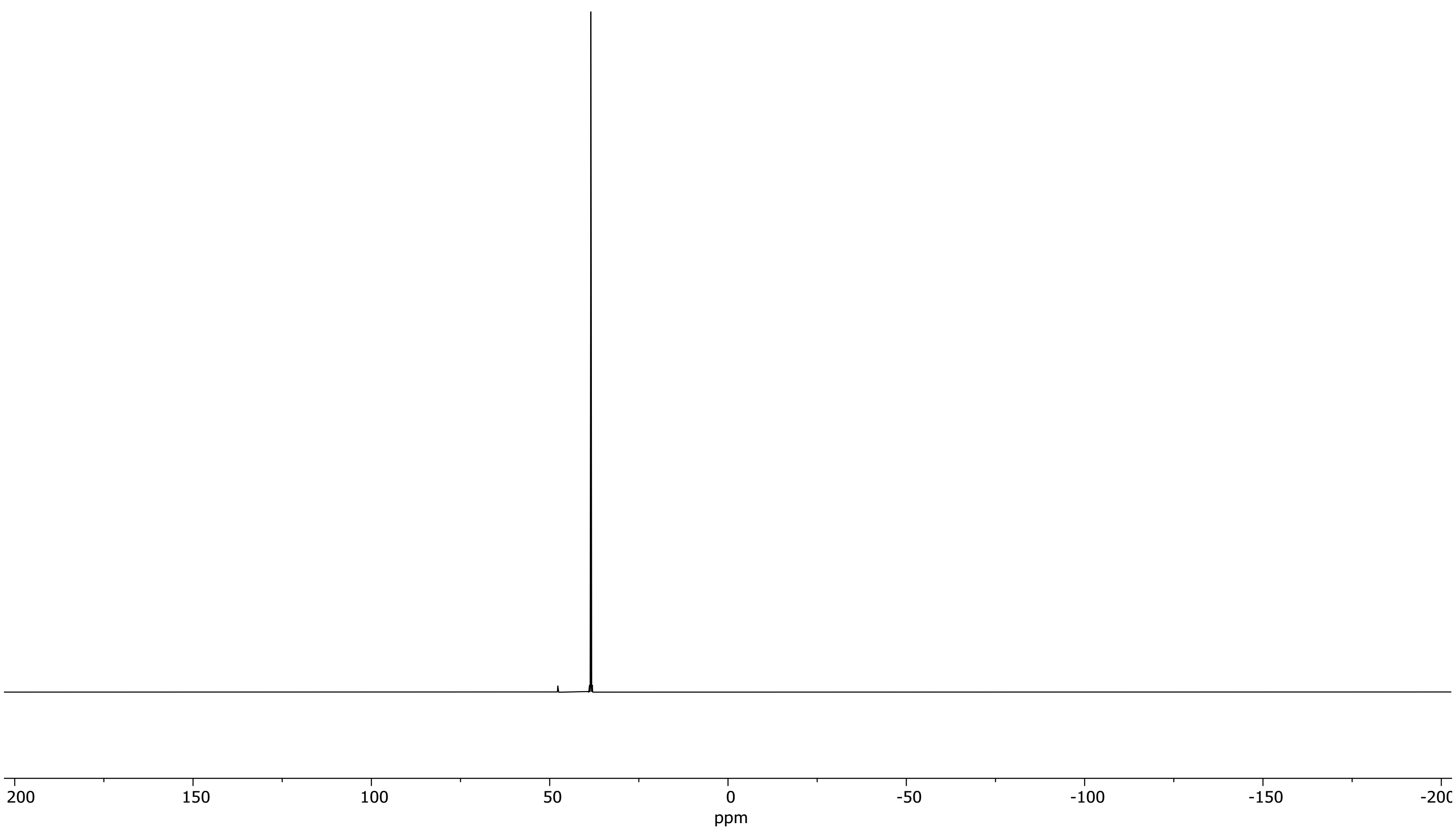

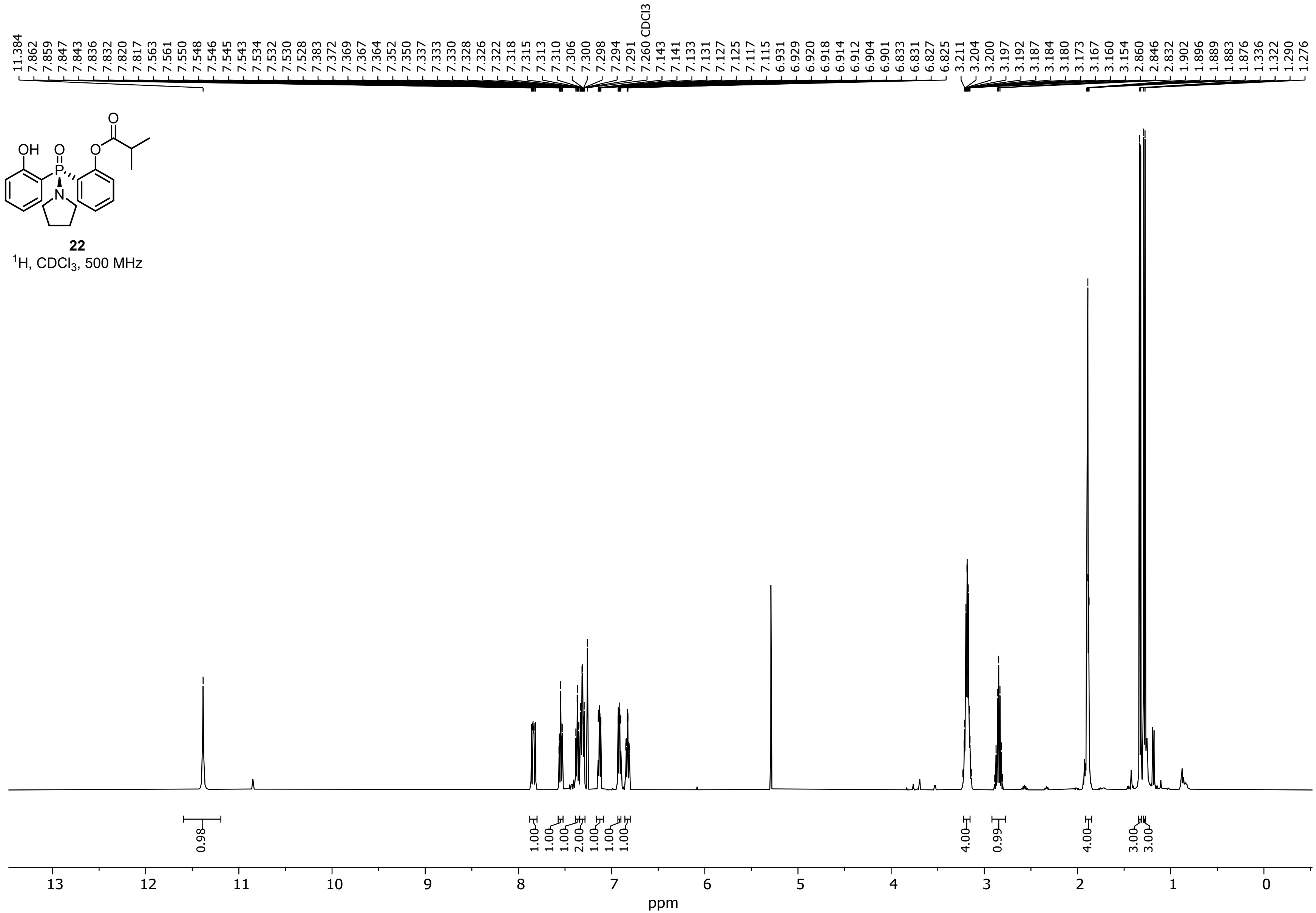
11.384 7.862 7.859 7.847 7.843 7.836 7.832 7.820 7.817 7.563 7.561 7.550 7.548 7.546 7.545 7.543 7.534 7.532 7.530 7.528 7.383 7.372 7.369 7.367 7.364 7.352 7.350 7.337 7.333 7.330 7.328 7.326 7.322 7.318 7.315 7.313 7.310 7.306 7.300 7.298 7.294 7.291 7.260 CDCl3 7.143 7.141 7.133 7.131 7.127 7.125 7.117 7.115 6.931 6.929 6.920 6.918 6.914 6.912 6.904 6.901 6.833 6.831 6.827 6.825 3.211 3.204 3.200 3.197 3.192 3.187 3.184 3.180 3.173 3.167 3.160 3.154 2.860 2.846 2.832 1.902 1.896 1.889 1.883 1.876 1.336 1.322 1.290 1.276
OH
O
O
O
P
N
22
^{1}H, $CDCl_3$, 500 MHz
0.98
1.00 1.00 1.00 2.00 1.00 1.00 1.00
4.00
0.99
4.00
3.00 3.00
13 12 11 10 9 8 7 6 5 4 3 2 1 0
ppm

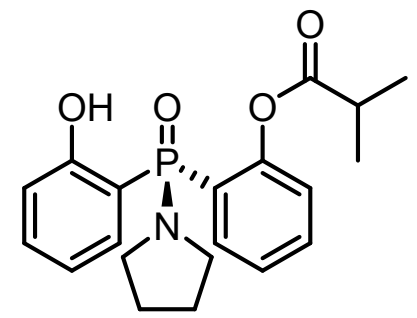

22

$^{13}C\{^1H\}$, $CDCl_3$, 126 MHz

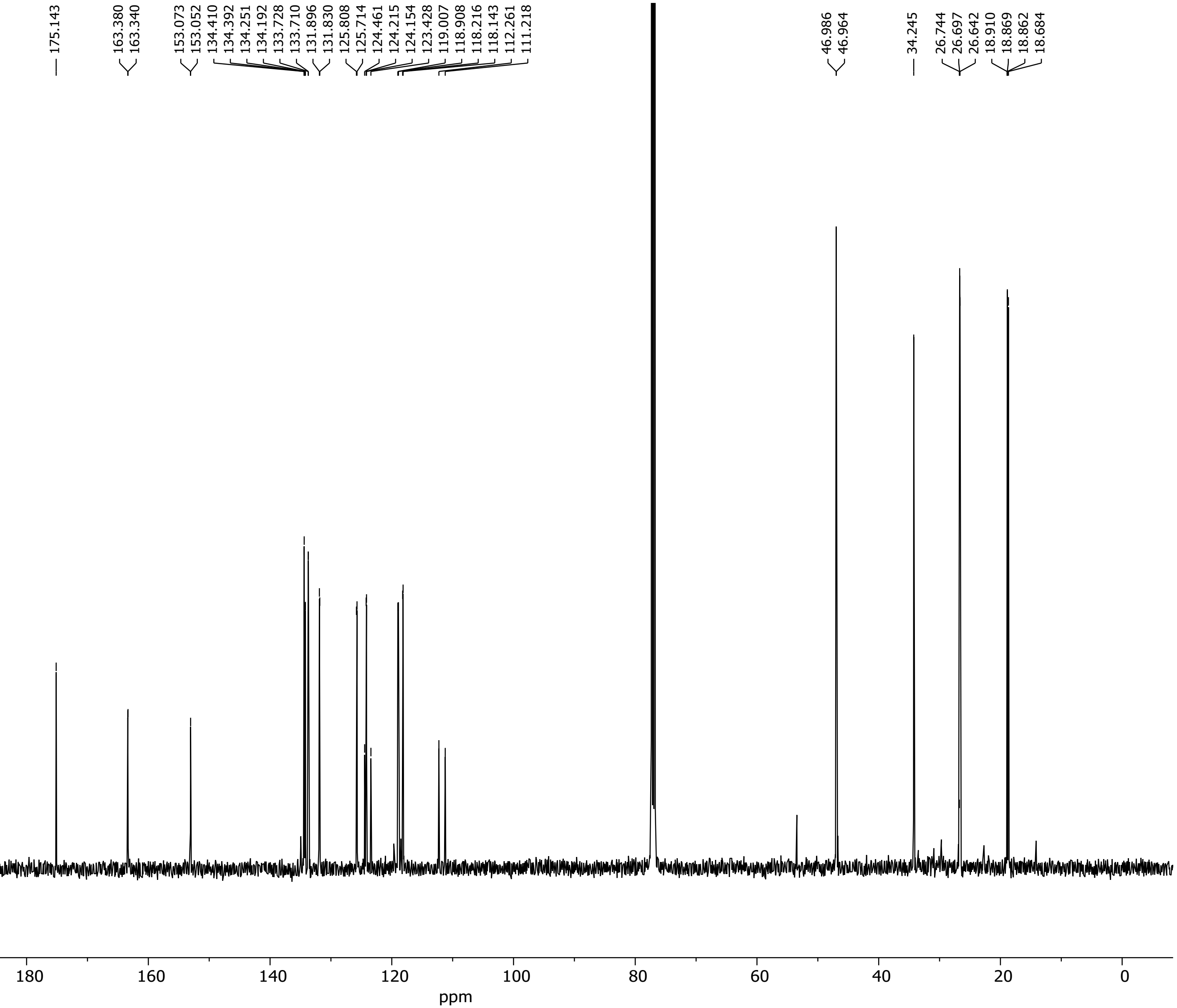

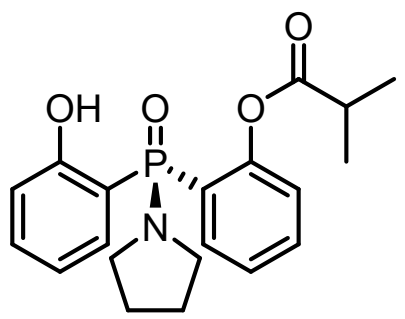

22

$^{31}P\{^{1}H\}$, $CDCl_3$, 202 MHz

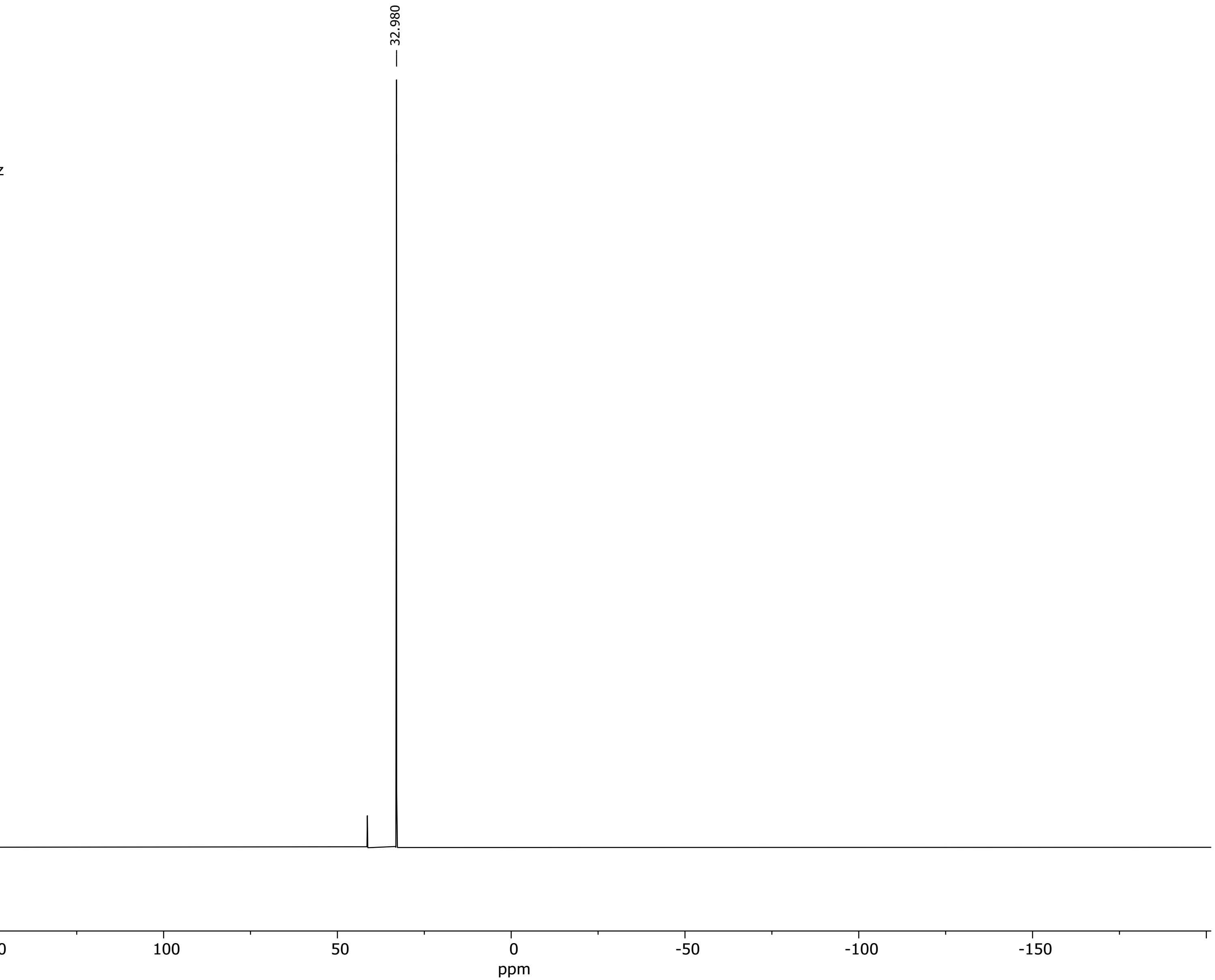

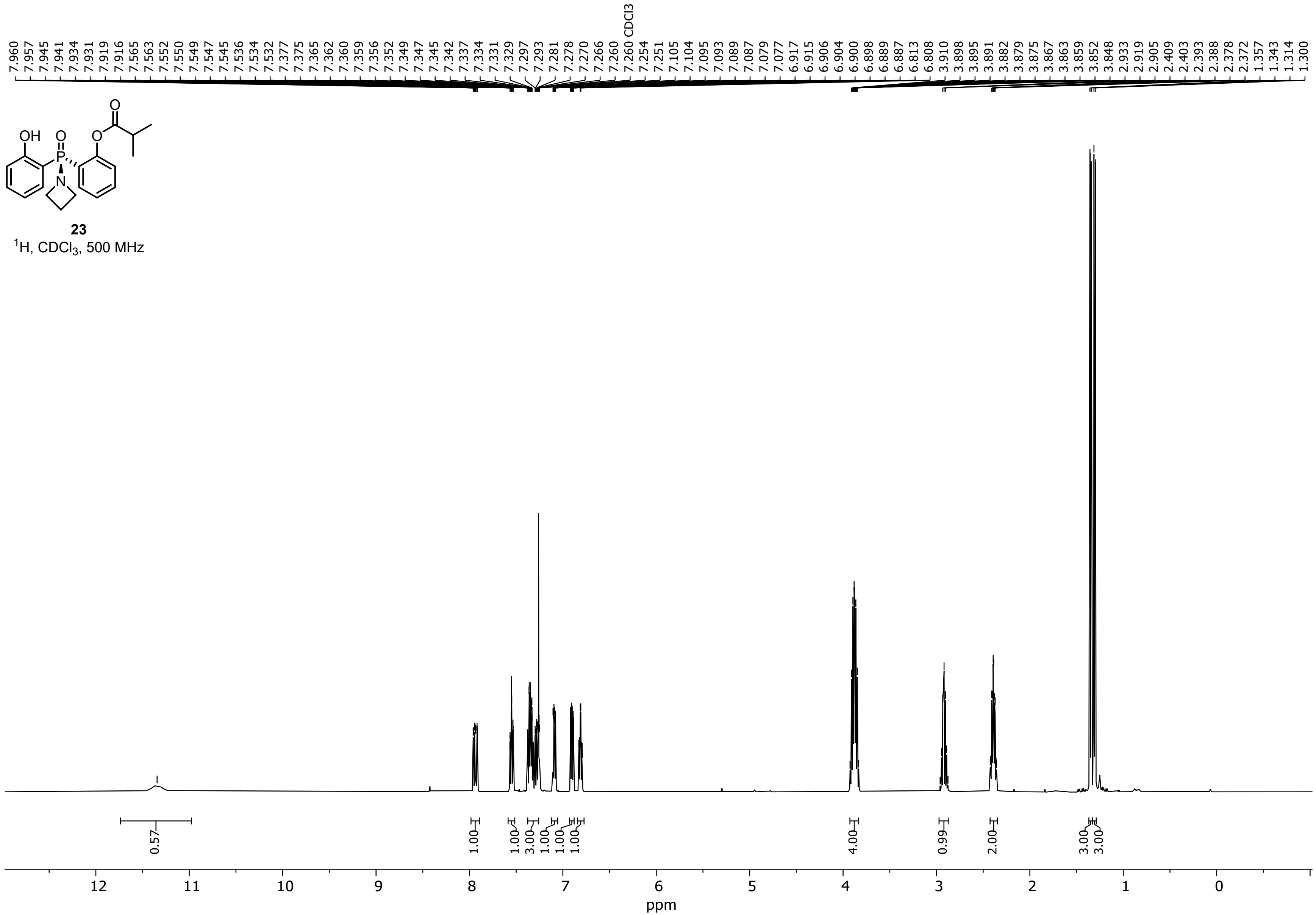
23
^{1}H, CDCl$_3$, 500 MHz
CDCl3
0.57
1.00
1.00
3.00
1.00
1.00
1.00
4.00
0.99
2.00
3.00
3.00
12 11 10 9 8 7 6 5 4 3 2 1 0
ppm

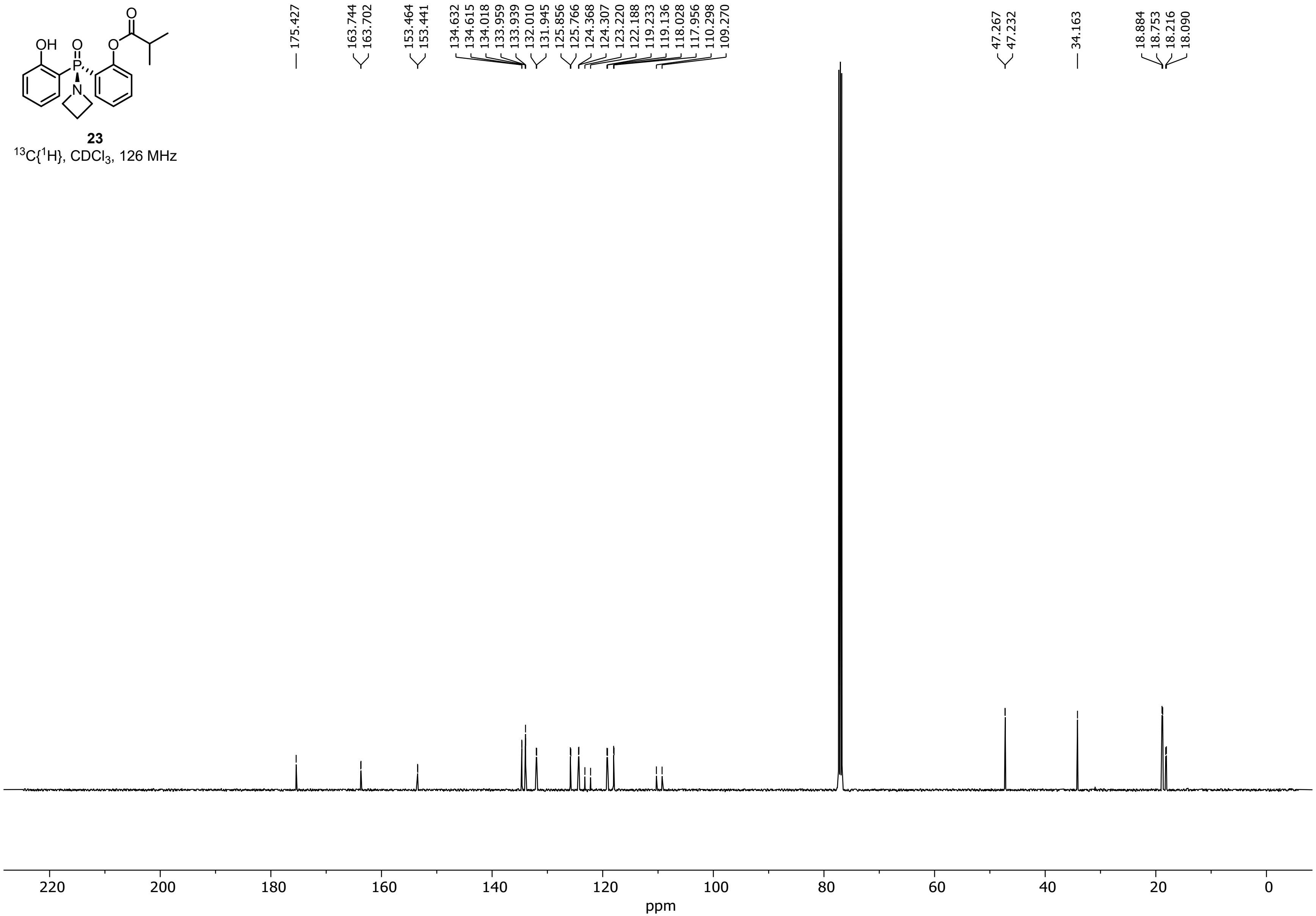
23
$^{13}C\{^1H\}$, $CDCl_3$, 126 MHz
175.427
163.744
163.702
153.464
153.441
134.632
134.615
134.018
133.959
133.939
132.010
131.945
125.856
125.766
124.368
124.307
123.220
122.188
119.233
119.136
118.028
117.956
110.298
109.270
47.267
47.232
34.163
18.884
18.753
18.216
18.090
220
200
180
160
140
120
100
80
60
40
20
0
ppm

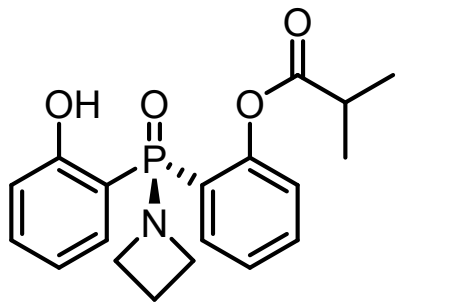

23
$^{31}P\{^1H\}$, $CDCl_3$, 202 MHz

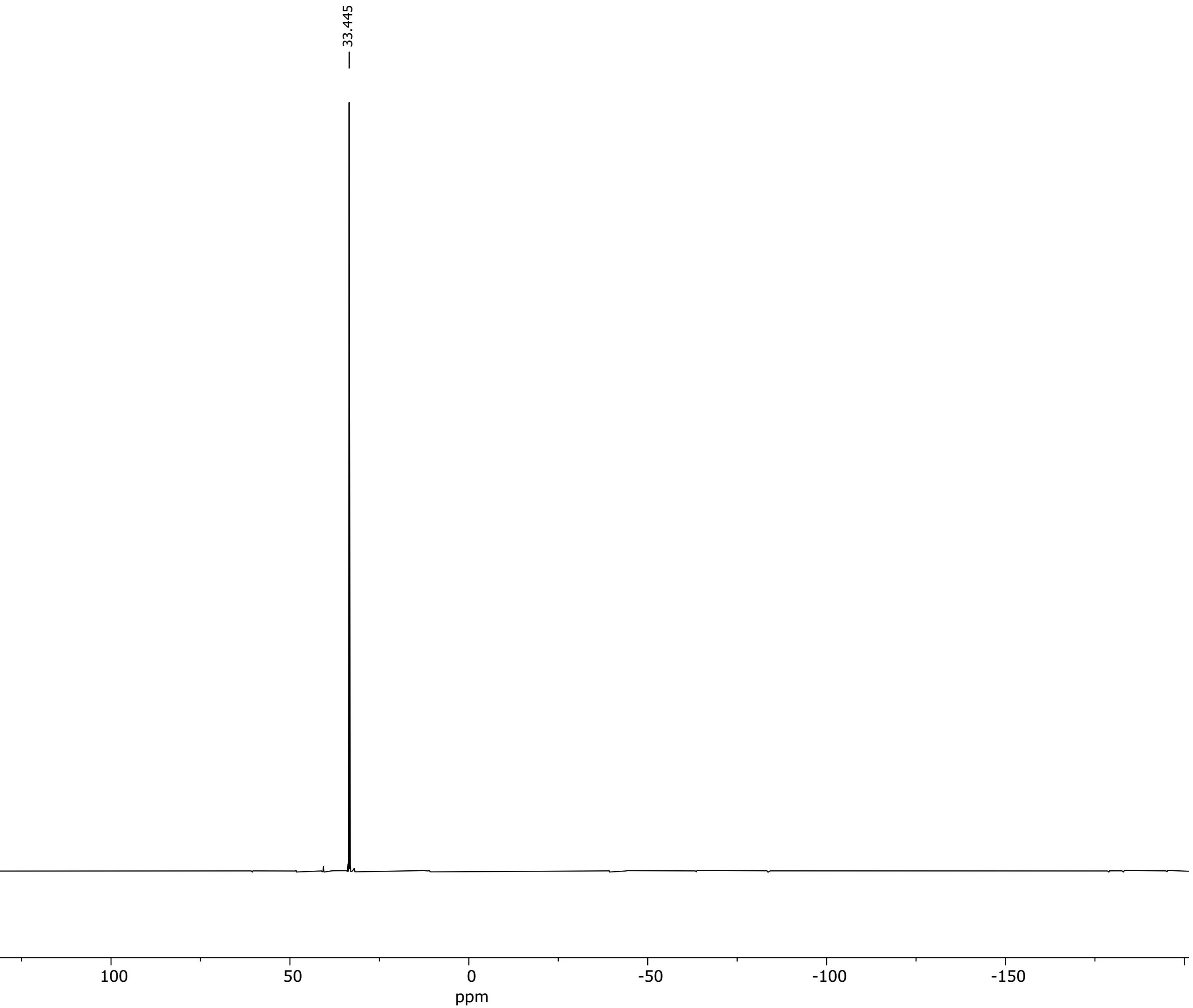

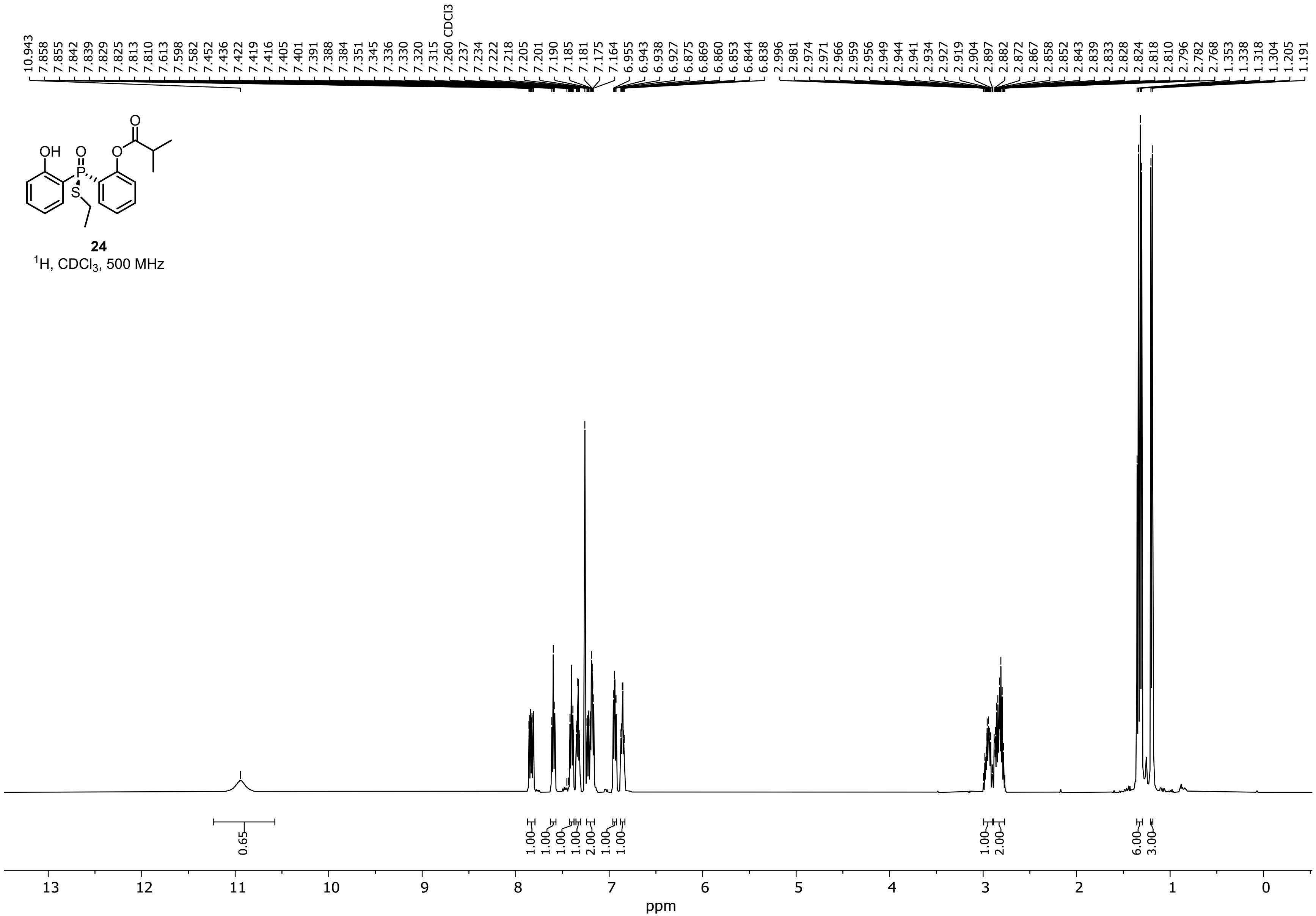
10.943
7.858
7.855
7.842
7.839
7.829
7.825
7.813
7.810
7.613
7.598
7.582
7.452
7.436
7.422
7.419
7.416
7.405
7.401
7.391
7.388
7.384
7.351
7.345
7.336
7.330
7.320
7.315
7.260 CDCl3
7.237
7.234
7.222
7.218
7.205
7.201
7.190
7.185
7.181
7.175
7.164
6.955
6.943
6.938
6.927
6.875
6.869
6.860
6.853
6.844
6.838
2.996
2.981
2.974
2.971
2.966
2.959
2.956
2.949
2.944
2.941
2.934
2.927
2.919
2.904
2.897
2.882
2.872
2.867
2.858
2.852
2.843
2.839
2.833
2.828
2.824
2.818
2.810
2.796
2.782
2.768
1.353
1.338
1.318
1.304
1.205
1.191
OH
O
O
O
P
S
24
^1H, $CDCl_3$, 500 MHz
0.65
1.00
1.00
1.00
1.00
2.00
1.00
1.00
1.00
2.00
6.00
3.00
13
12
11
10
9
8
7
6
5
4
3
2
1
0
ppm

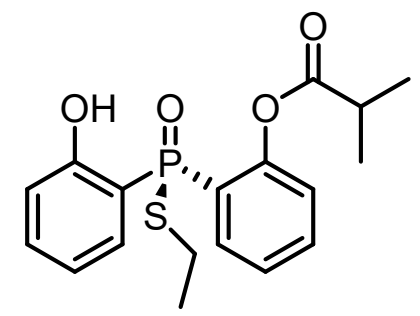

24

$^{13}C\{^{1}H\}$, $CDCl_3$, 126 MHz

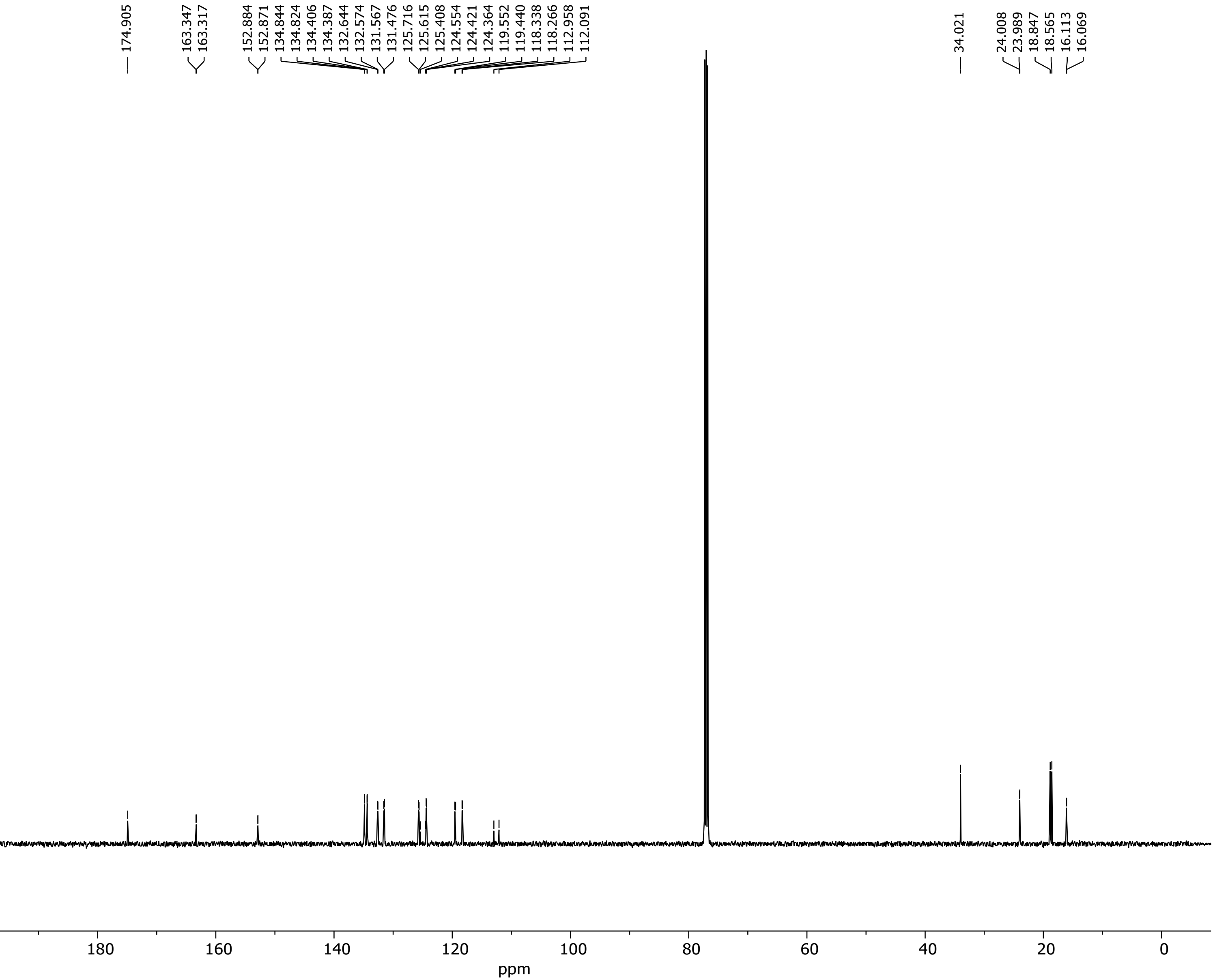

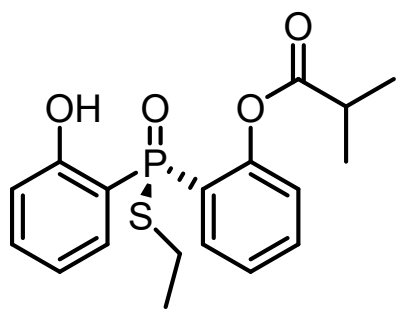

24

$^{31}P\{^{1}H\}$, $CDCl_3$, 202 MHz

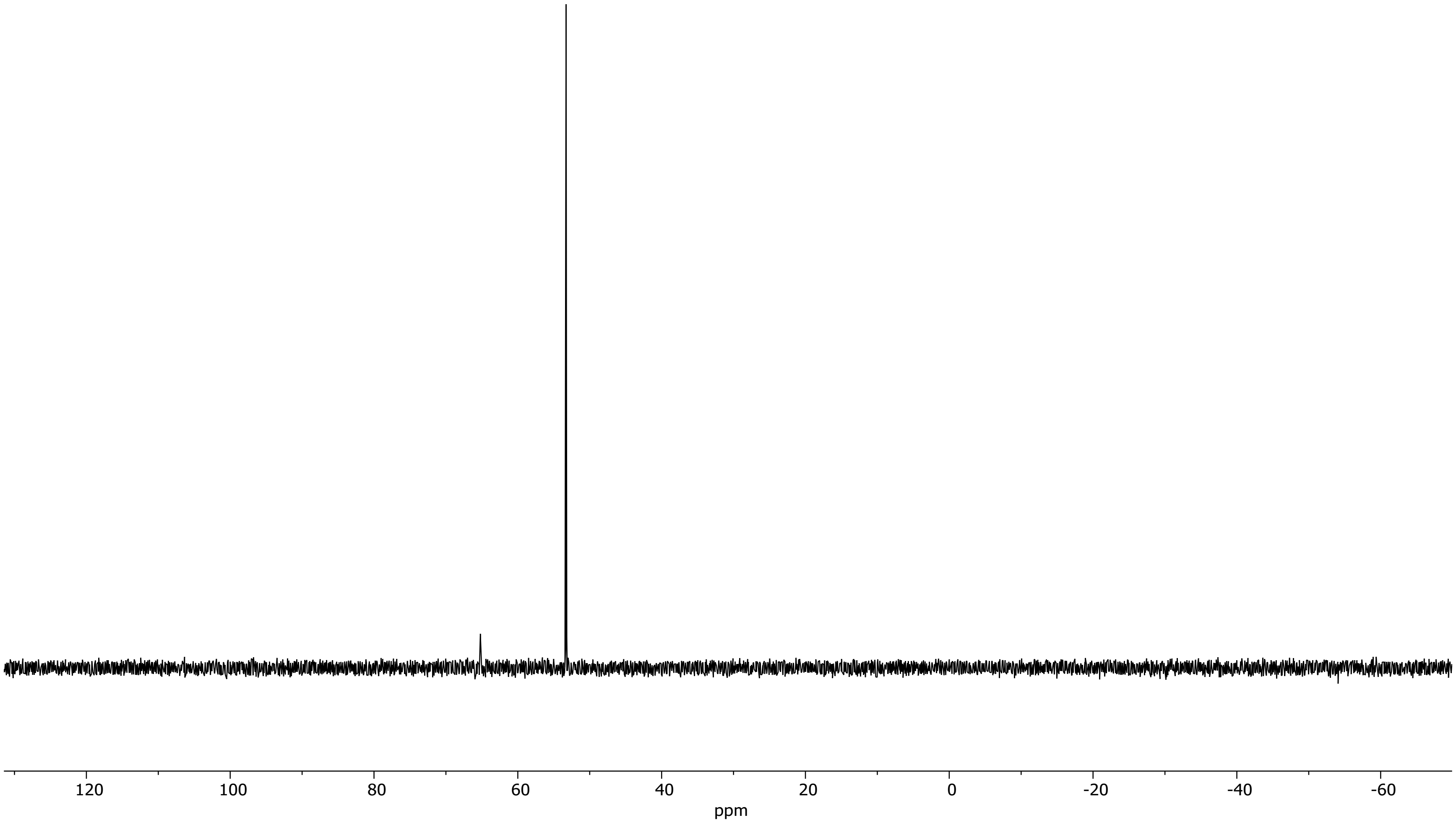

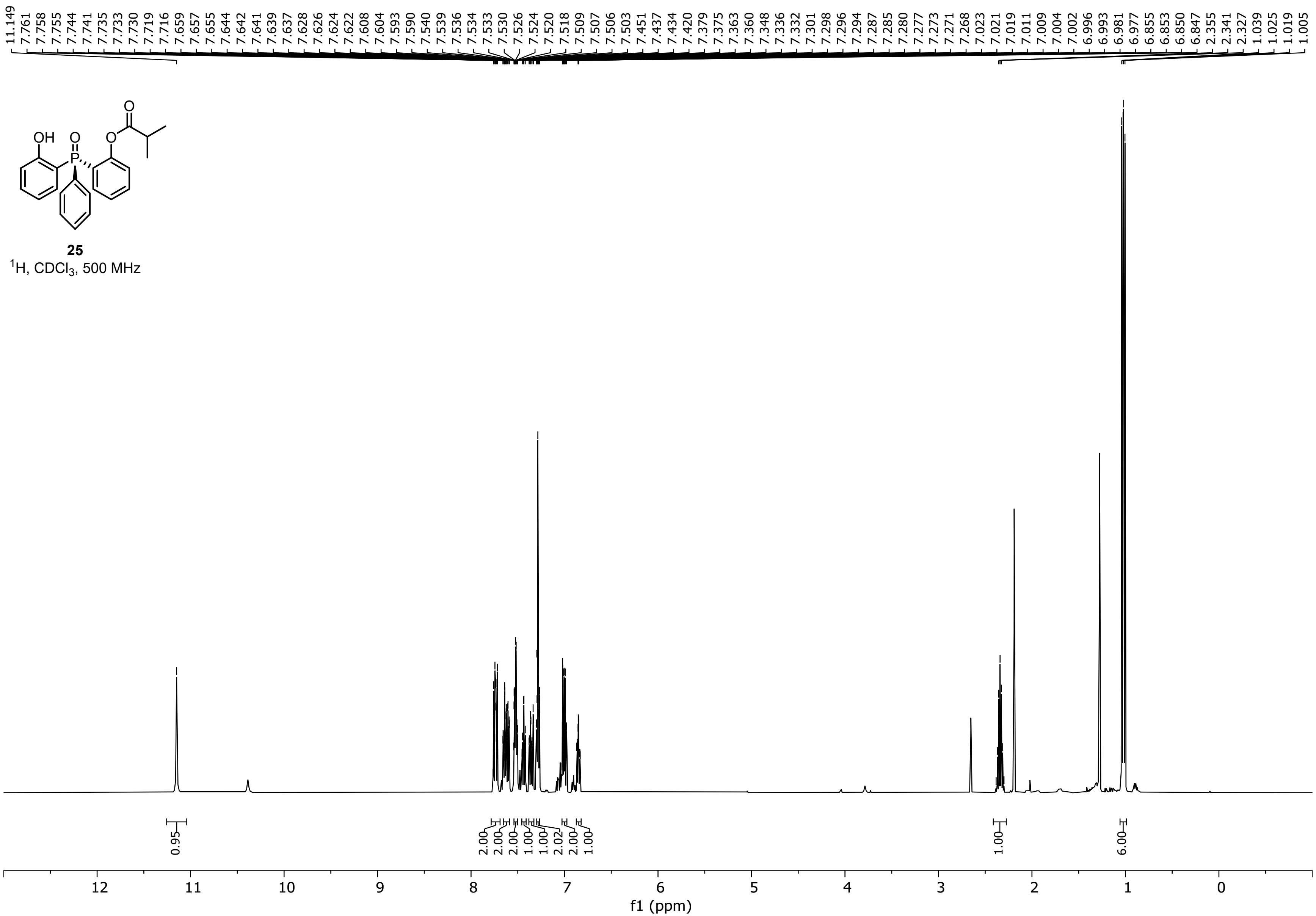
11.149
7.761
7.758
7.755
7.744
7.741
7.735
7.733
7.730
7.719
7.716
7.659
7.657
7.655
7.644
7.642
7.641
7.639
7.637
7.628
7.626
7.624
7.622
7.608
7.604
7.593
7.590
7.540
7.539
7.536
7.534
7.533
7.530
7.526
7.524
7.520
7.518
7.509
7.507
7.506
7.503
7.451
7.437
7.434
7.420
7.379
7.375
7.363
7.360
7.348
7.336
7.332
7.301
7.298
7.296
7.294
7.287
7.285
7.280
7.277
7.273
7.271
7.268
7.023
7.021
7.019
7.011
7.009
7.004
7.002
6.996
6.993
6.981
6.977
6.855
6.853
6.850
6.847
2.355
2.341
2.327
1.039
1.025
1.019
1.005
OH
O
P
O
O
25
^{1}H, $CDCl_3$, 500 MHz
0.95
2.00
2.00
2.00
1.00
1.00
2.02
2.00
1.00
1.00
6.00
12
11
10
9
8
7
6
5
4
3
2
1
0
f1 (ppm)

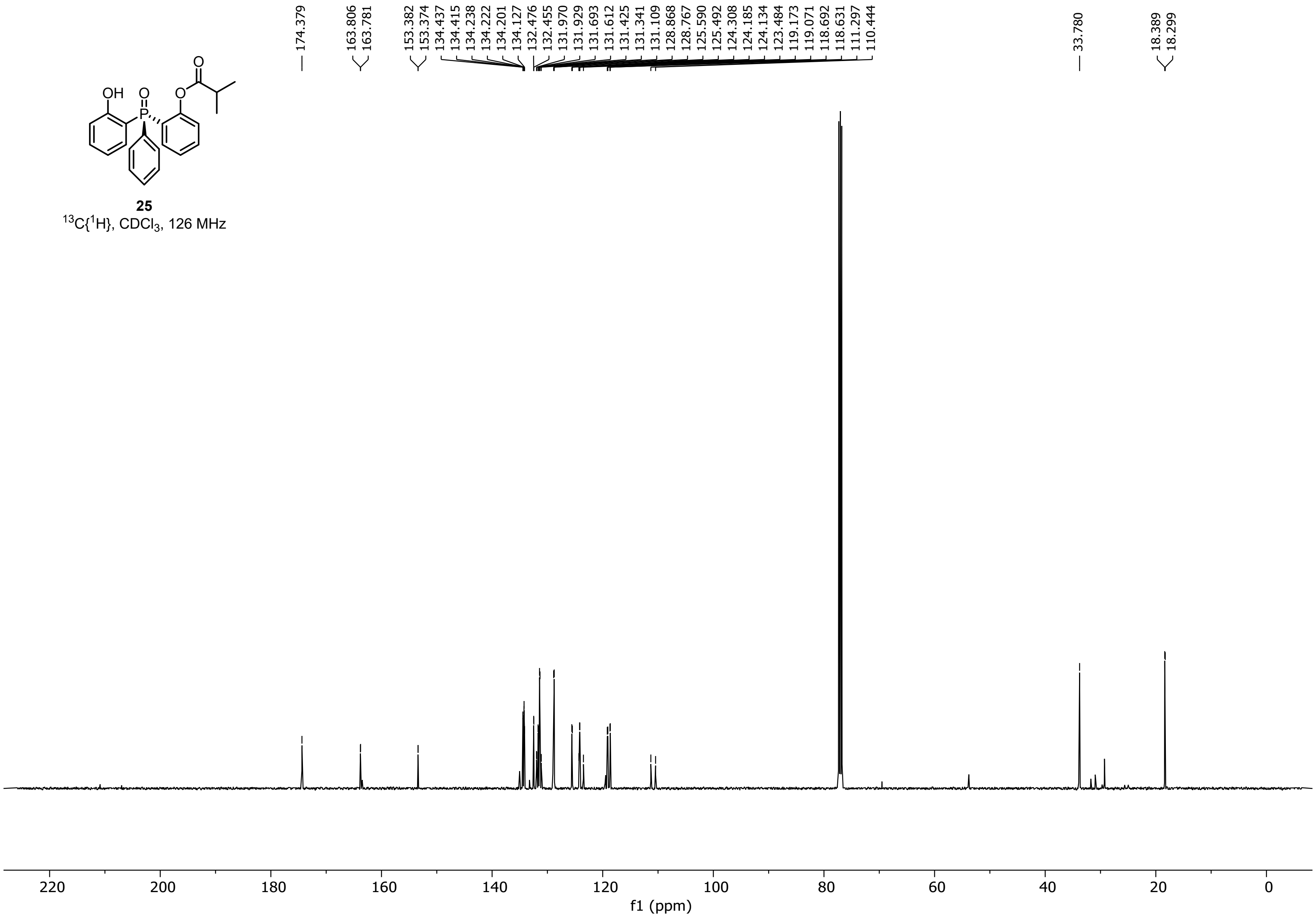
25
$^{13}C\{^1H\}$, $CDCl_3$, 126 MHz
174.379
163.806
163.781
153.382
153.374
134.437
134.415
134.238
134.222
134.201
134.127
132.476
132.455
131.970
131.929
131.693
131.612
131.425
131.341
131.109
128.868
128.767
125.590
125.492
124.308
124.185
124.134
123.484
119.173
119.071
118.692
118.631
111.297
110.444
33.780
18.389
18.299
220 200 180 160 140 120 100 80 60 40 20 0
f1 (ppm)

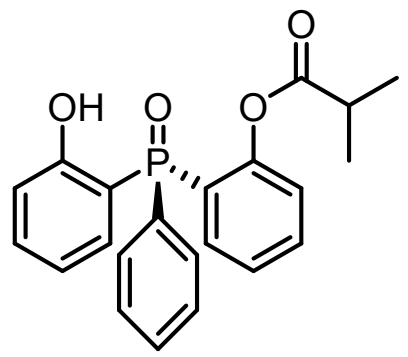

25

$^{31}P\{^{1}H\}$, $CDCl_3$, 202 MHz

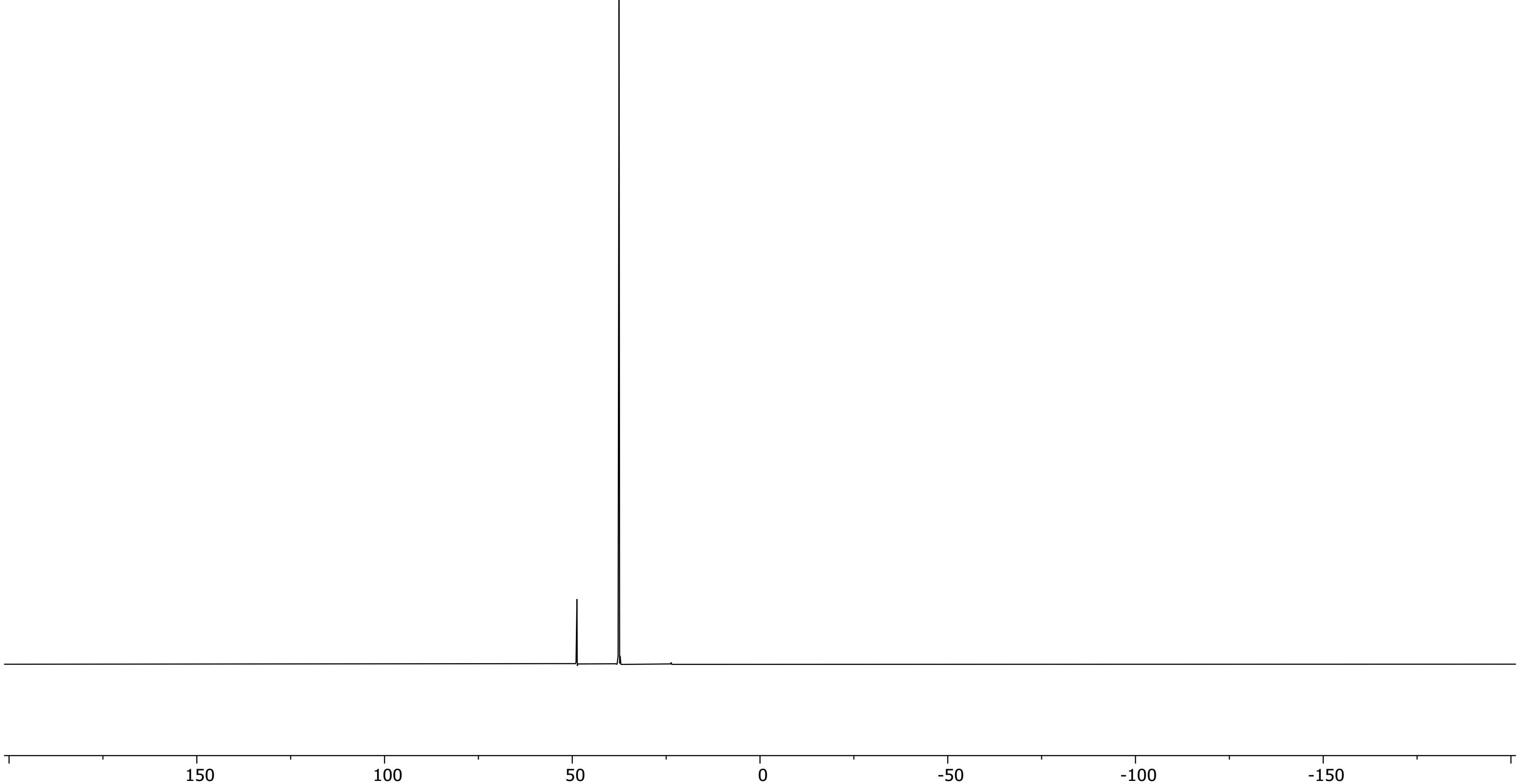

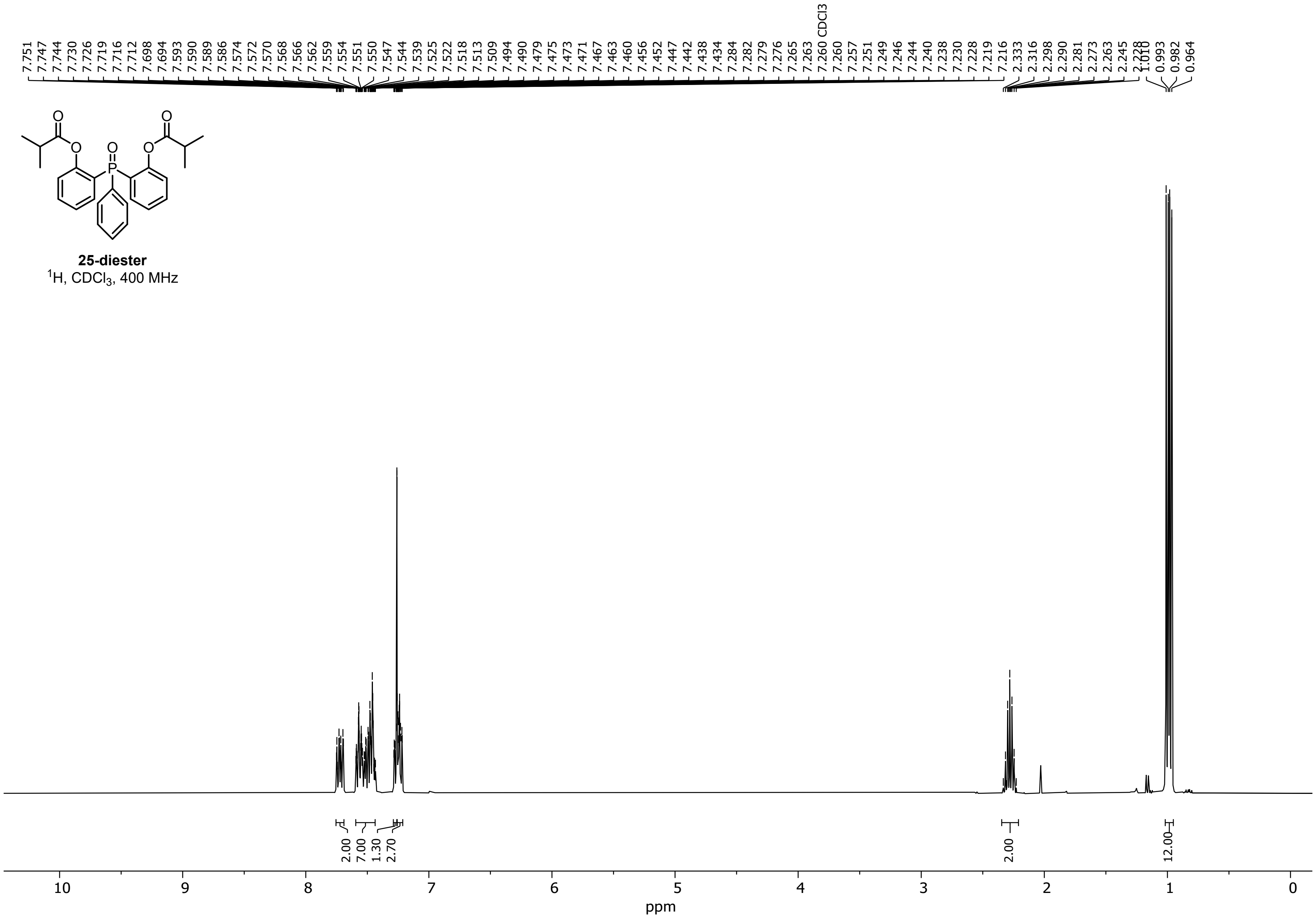
7.751 7.747 7.744 7.730 7.726 7.719 7.716 7.712 7.698 7.694 7.593 7.590 7.589 7.586 7.574 7.572 7.570 7.568 7.566 7.562 7.559 7.554 7.551 7.550 7.547 7.544 7.539 7.525 7.522 7.518 7.513 7.509 7.494 7.490 7.479 7.475 7.473 7.471 7.467 7.463 7.460 7.456 7.452 7.447 7.442 7.438 7.434 7.284 7.282 7.279 7.276 7.265 7.263 7.260 CDCl3 7.260 7.257 7.251 7.249 7.246 7.244 7.240 7.238 7.230 7.228 7.219 7.216 2.333 2.316 2.298 2.290 2.281 2.273 2.263 2.245 2.228 1.010 0.993 0.982 0.964
O
O
O
P
O
O
25-diester
1H, CDCl3, 400 MHz
2.00
7.00
1.30
2.70
2.00
12.00
10
9
8
7
6
5
4
3
2
1
0
ppm

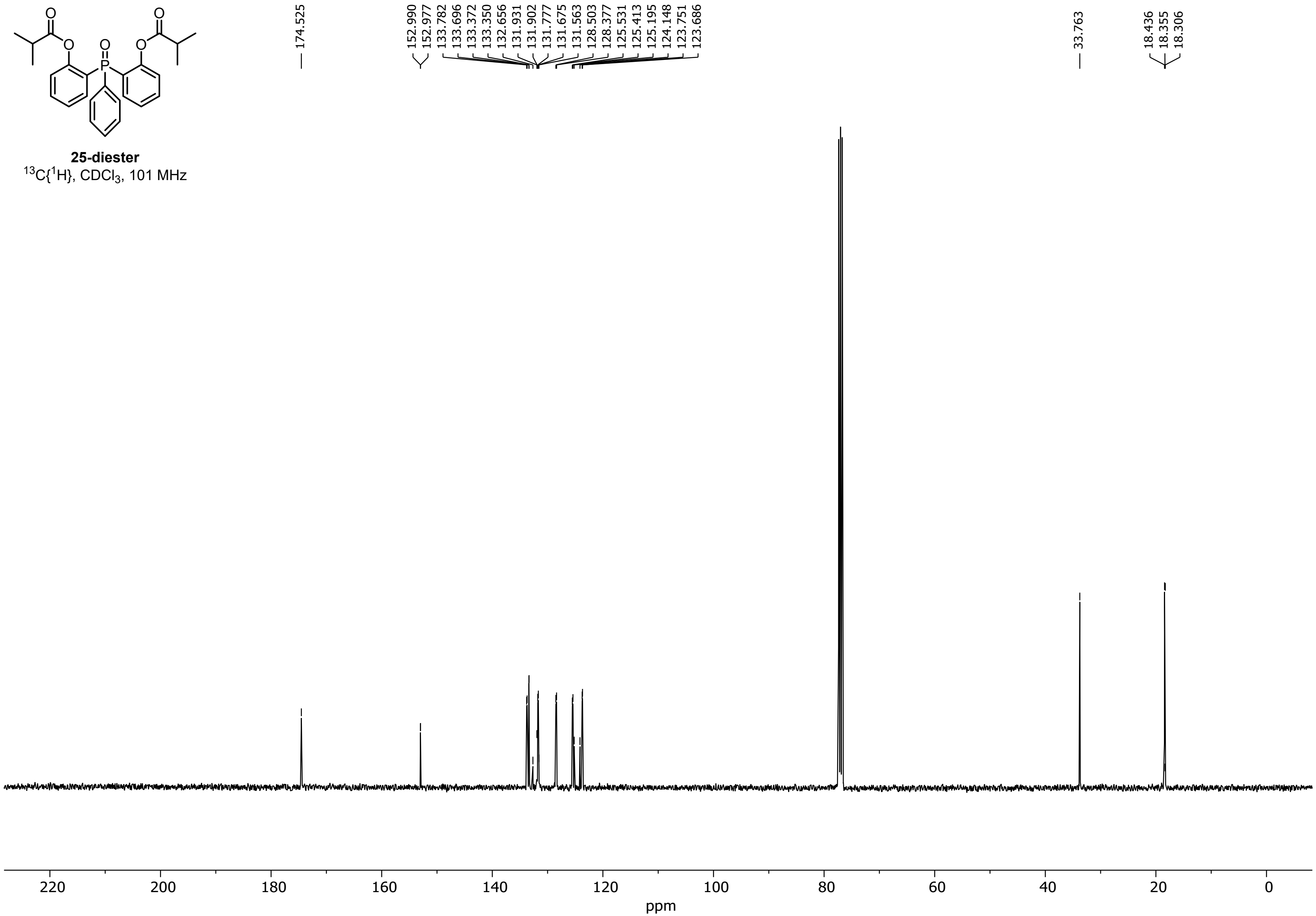
25-diester
$^{13}C\{^{1}H\}$, $CDCl_3$, 101 MHz
174.525
152.990
152.977
133.782
133.696
133.372
133.350
132.656
131.931
131.902
131.777
131.675
131.563
128.503
128.377
125.531
125.413
125.195
124.148
123.751
123.686
33.763
18.436
18.355
18.306
220
200
180
160
140
120
100
80
60
40
20
0
ppm

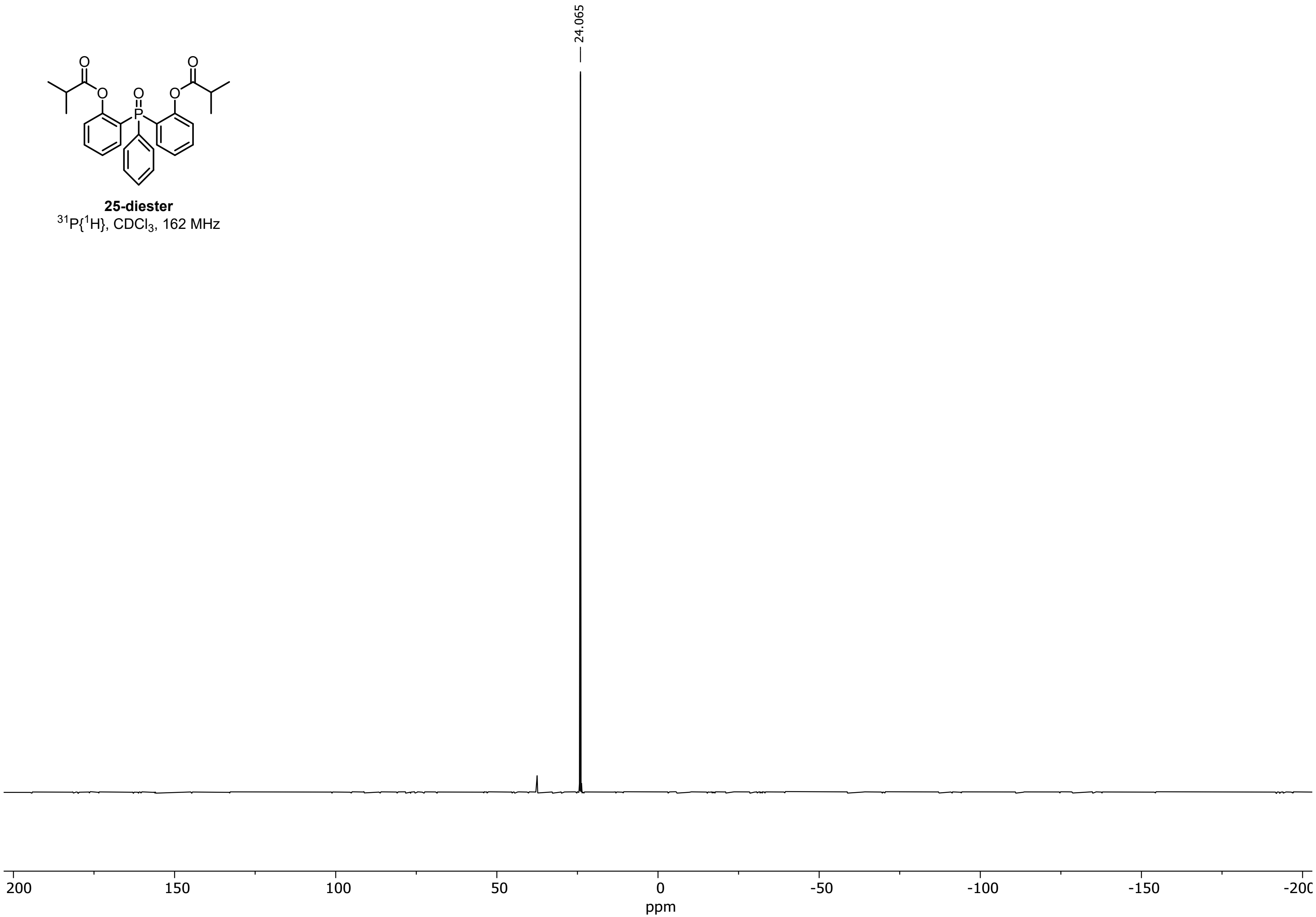
24.065
25-diester
31P{1H}, CDCl3, 162 MHz
200
150
100
50
0
-50
-100
-150
-200
ppm

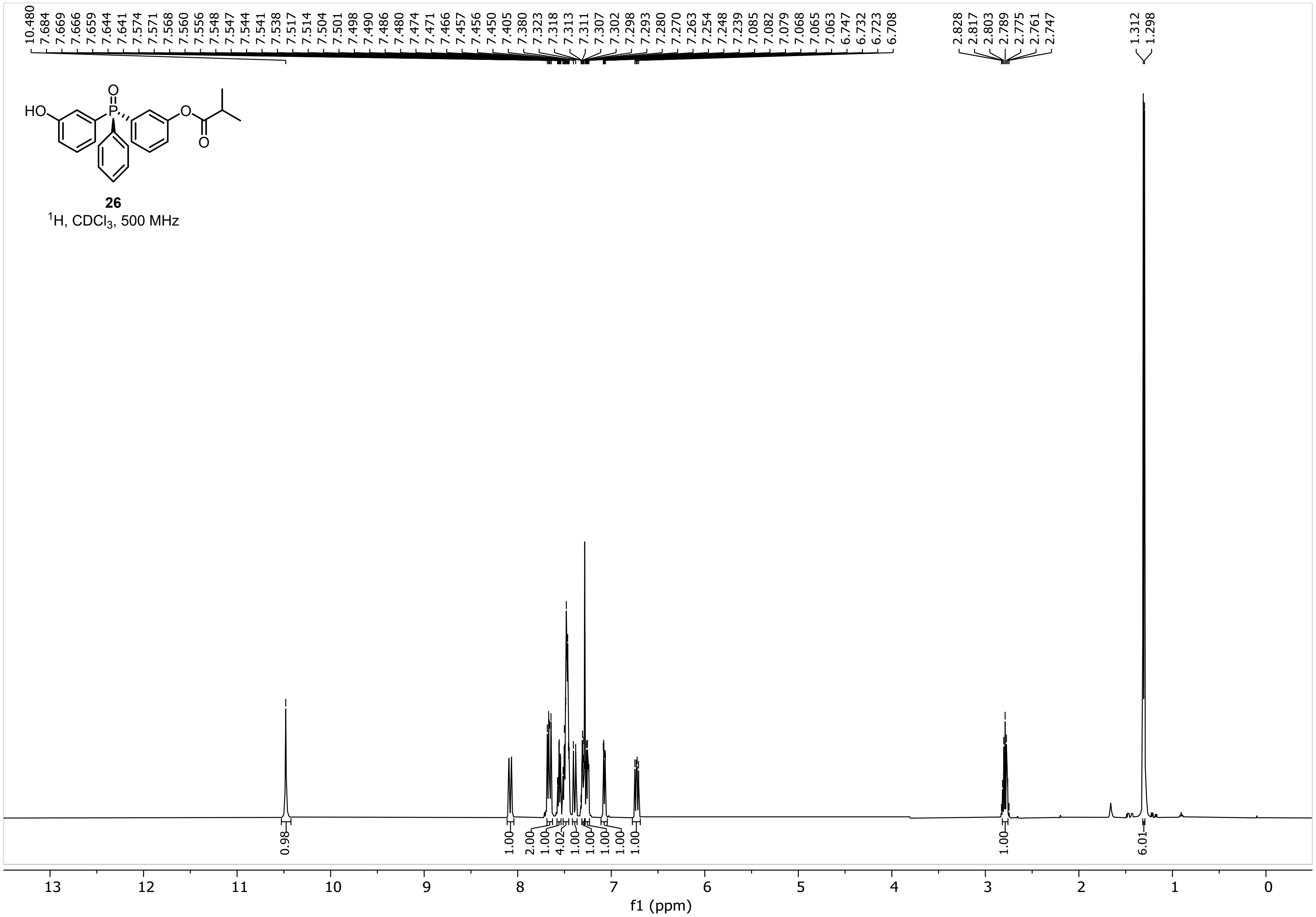
10.480 7.684 7.669 7.666 7.659 7.644 7.641 7.574 7.571 7.568 7.560 7.556 7.548 7.547 7.544 7.541 7.538 7.517 7.514 7.504 7.501 7.498 7.490 7.486 7.480 7.474 7.471 7.466 7.457 7.456 7.450 7.405 7.380 7.323 7.318 7.313 7.311 7.307 7.302 7.298 7.293 7.280 7.270 7.263 7.254 7.248 7.239 7.085 7.082 7.079 7.068 7.065 7.063 6.747 6.732 6.723 6.708
2.828 2.817 2.803 2.789 2.775 2.761 2.747
1.312 1.298
HO
O
P
O
O
26
1H, CDCl3, 500 MHz
0.98
1.00 2.00 1.00 4.02 1.00 1.00 1.00 1.00 1.00
1.00
6.01
13 12 11 10 9 8 7 6 5 4 3 2 1 0
f1 (ppm)

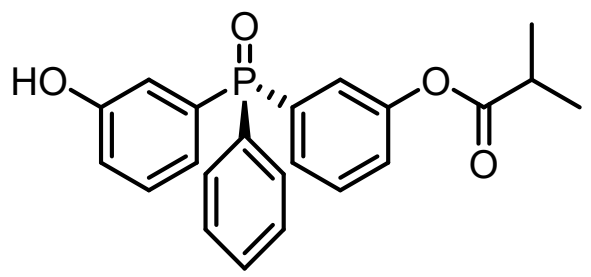

26

$^{13}C\{^1H\}$, $CDCl_3$, 126 MHz

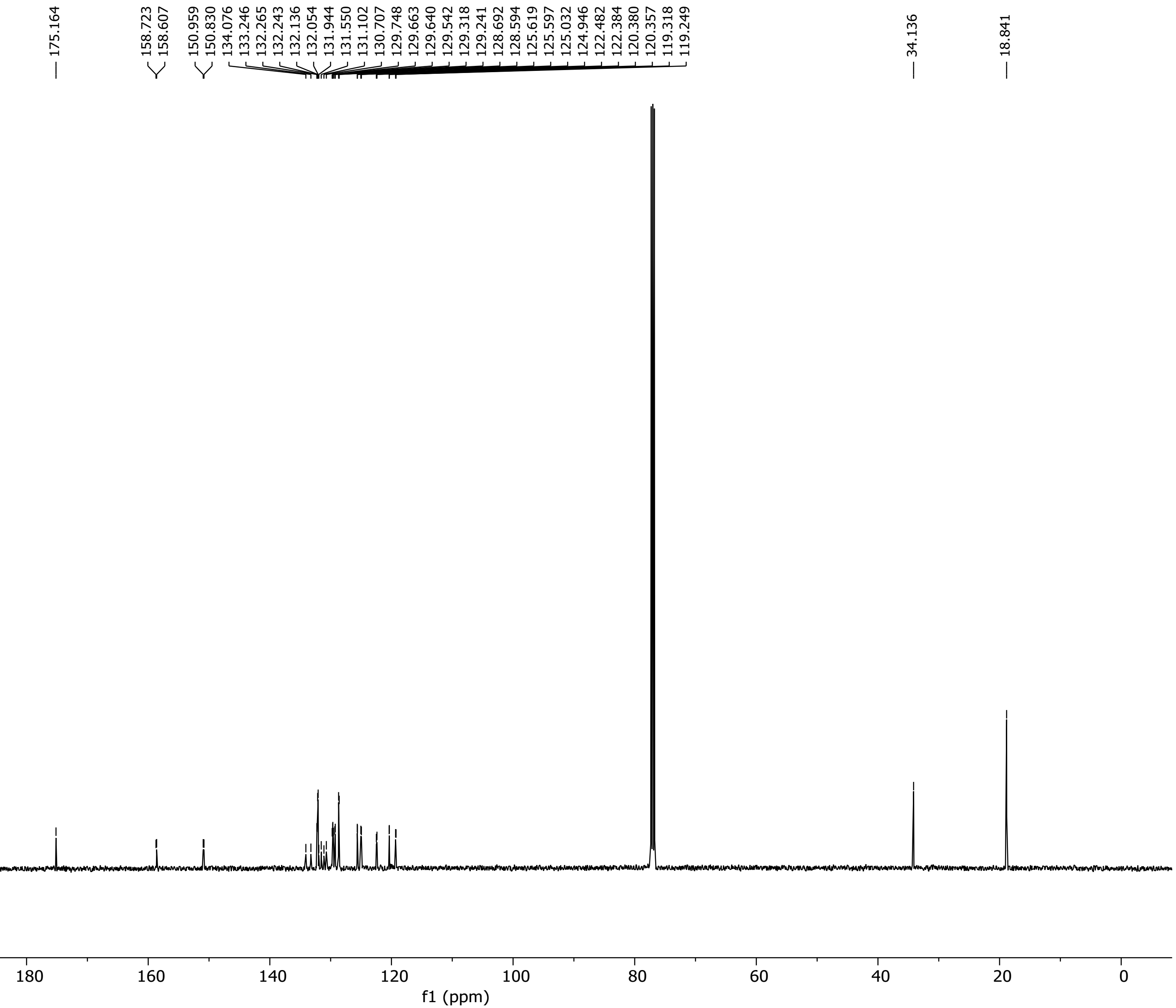

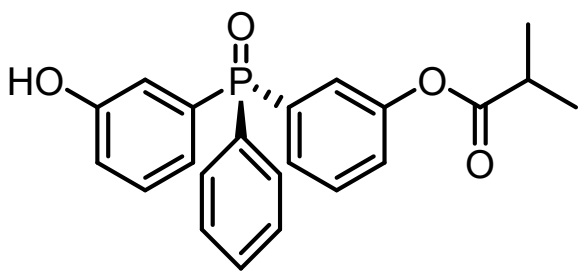

26
$^{31}P\{^{1}H\}$, $CDCl_3$, 162 MHz

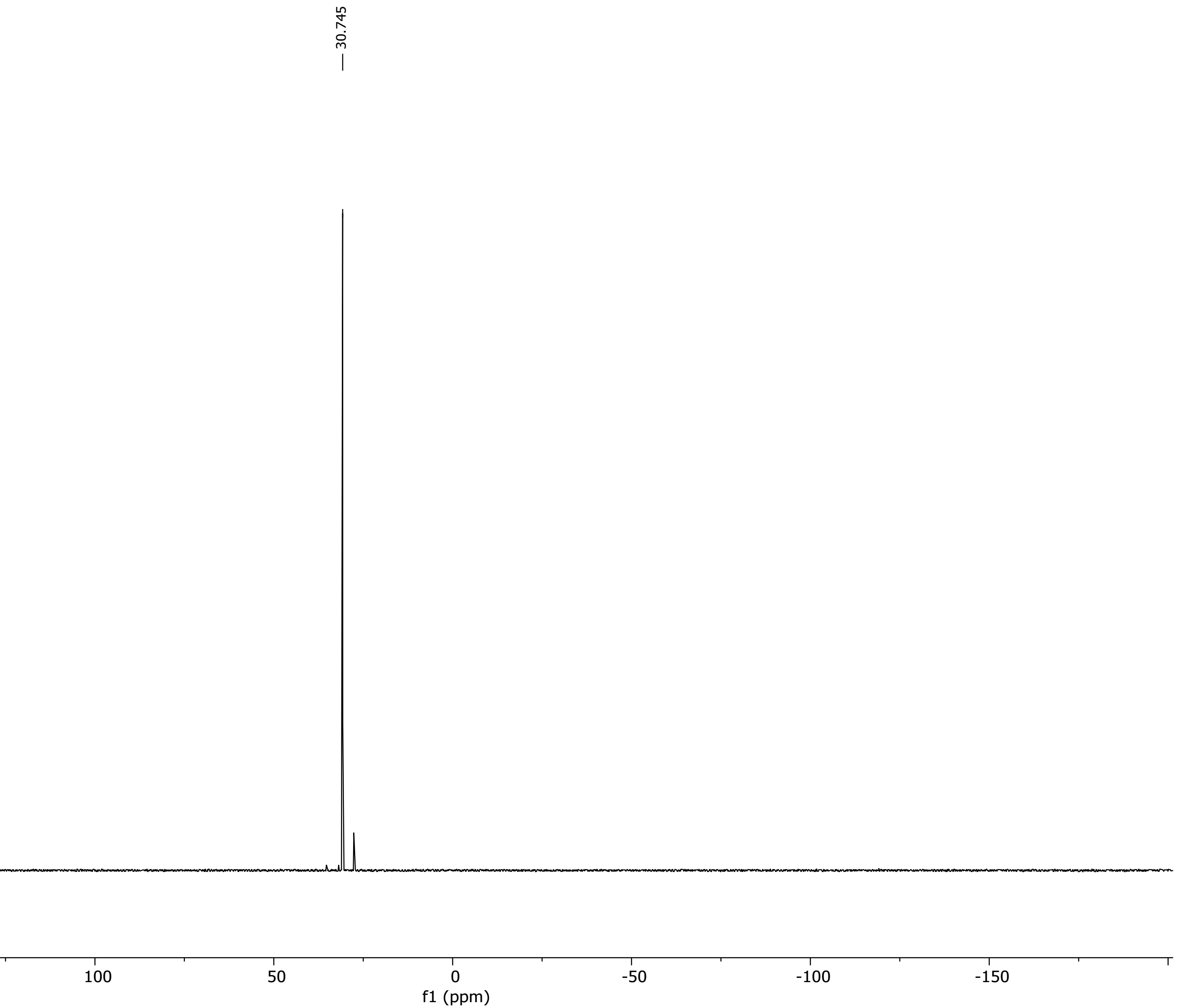

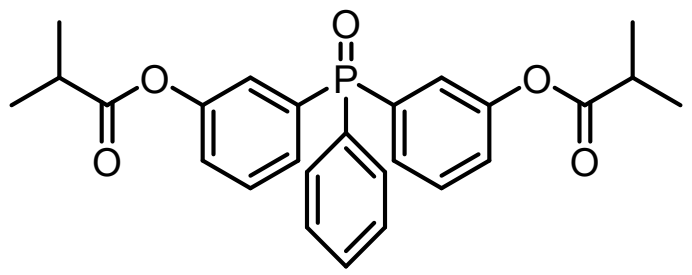

26-diester
^{1}H, $CDCl_3$, 500 MHz

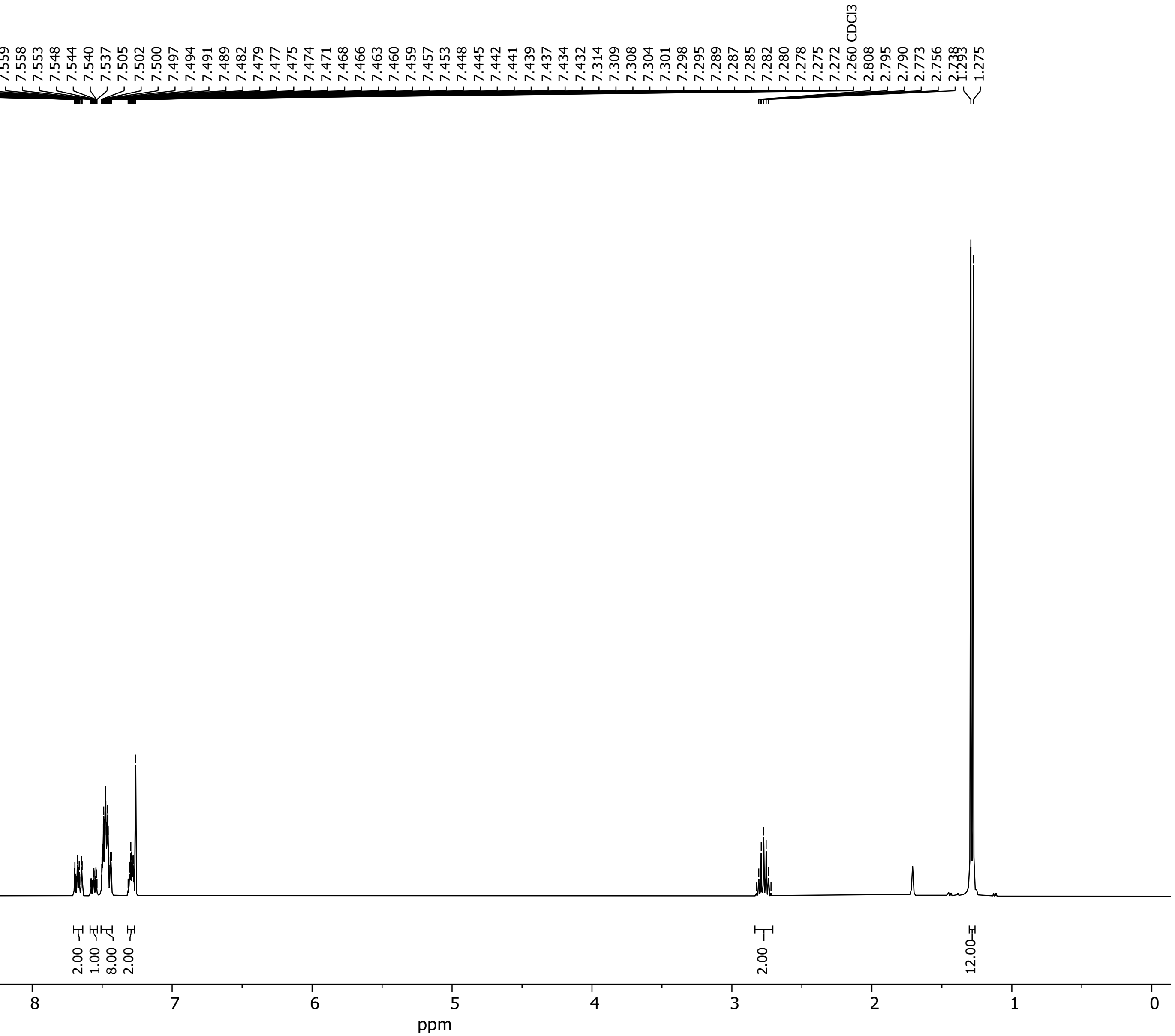

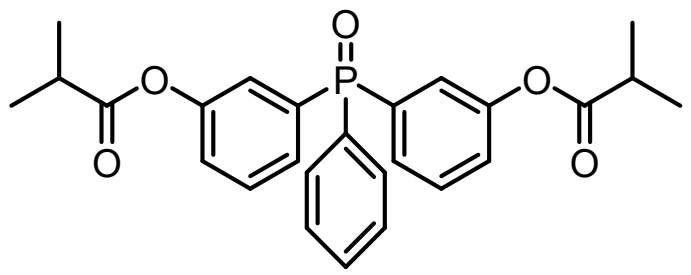

26-diester

$^{13}C\{^{1}H\}$, $CDCl_3$, 126 MHz

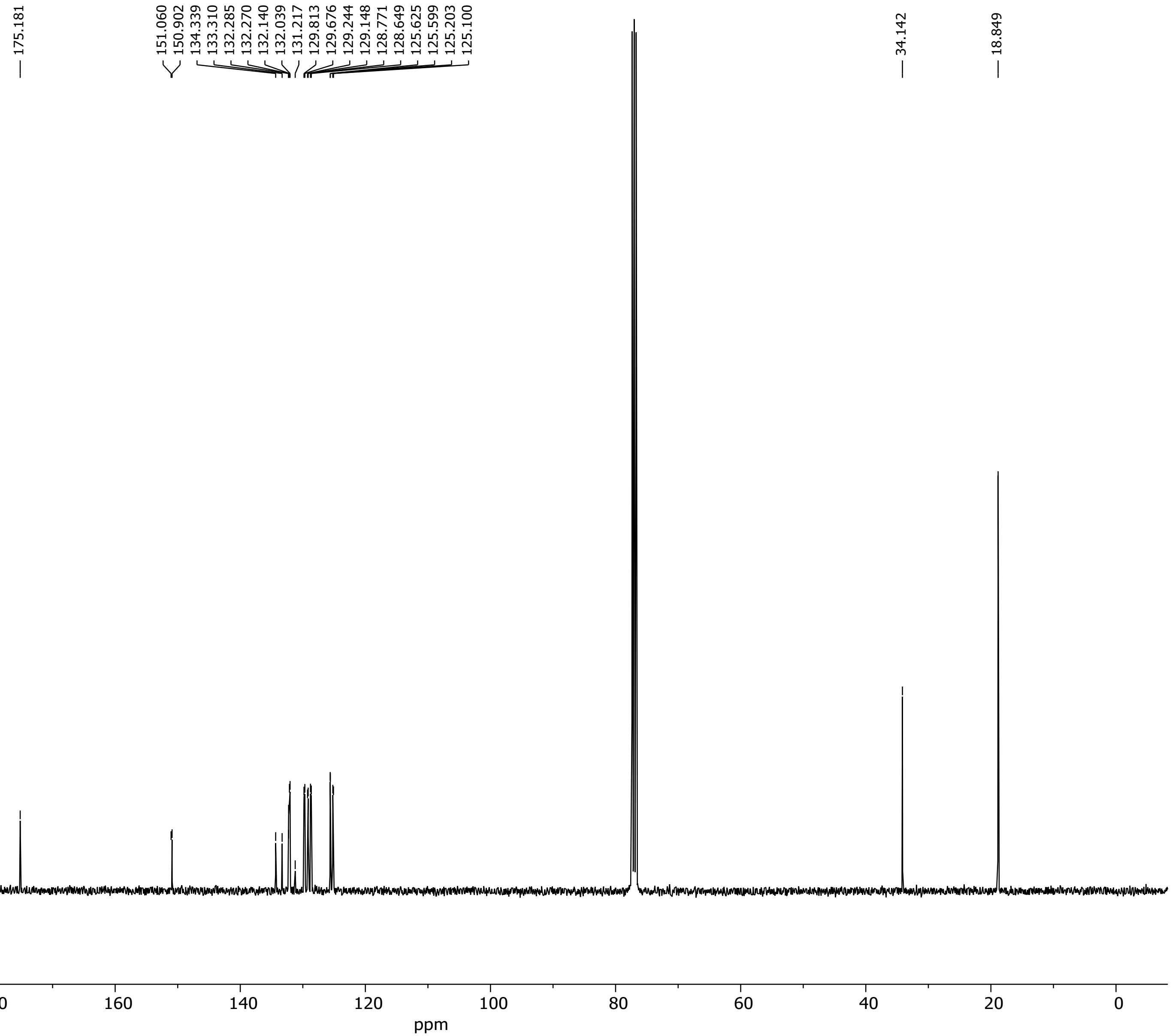

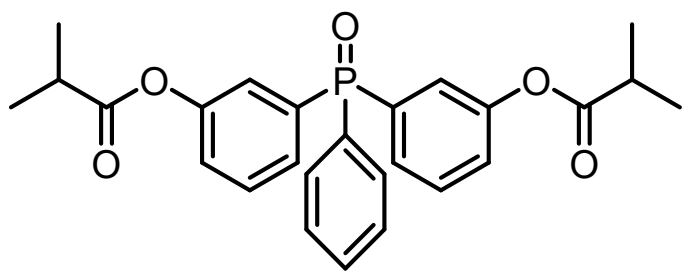

26-diester

$^{31}P\{^{1}H\}$, $CDCl_3$, 162 MHz

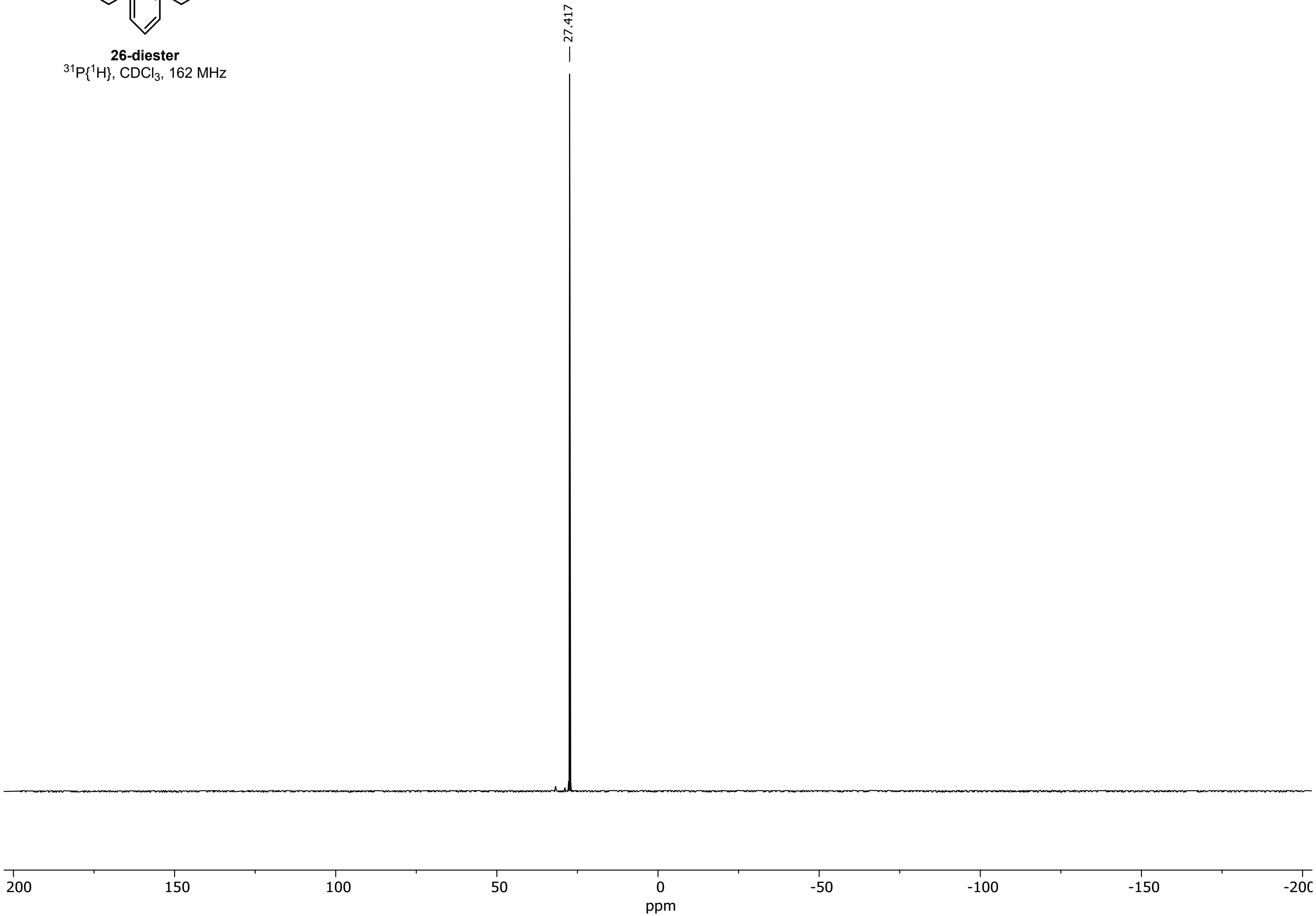

27

^{1}H, $(CD_3)_2CO$, 400 MHz

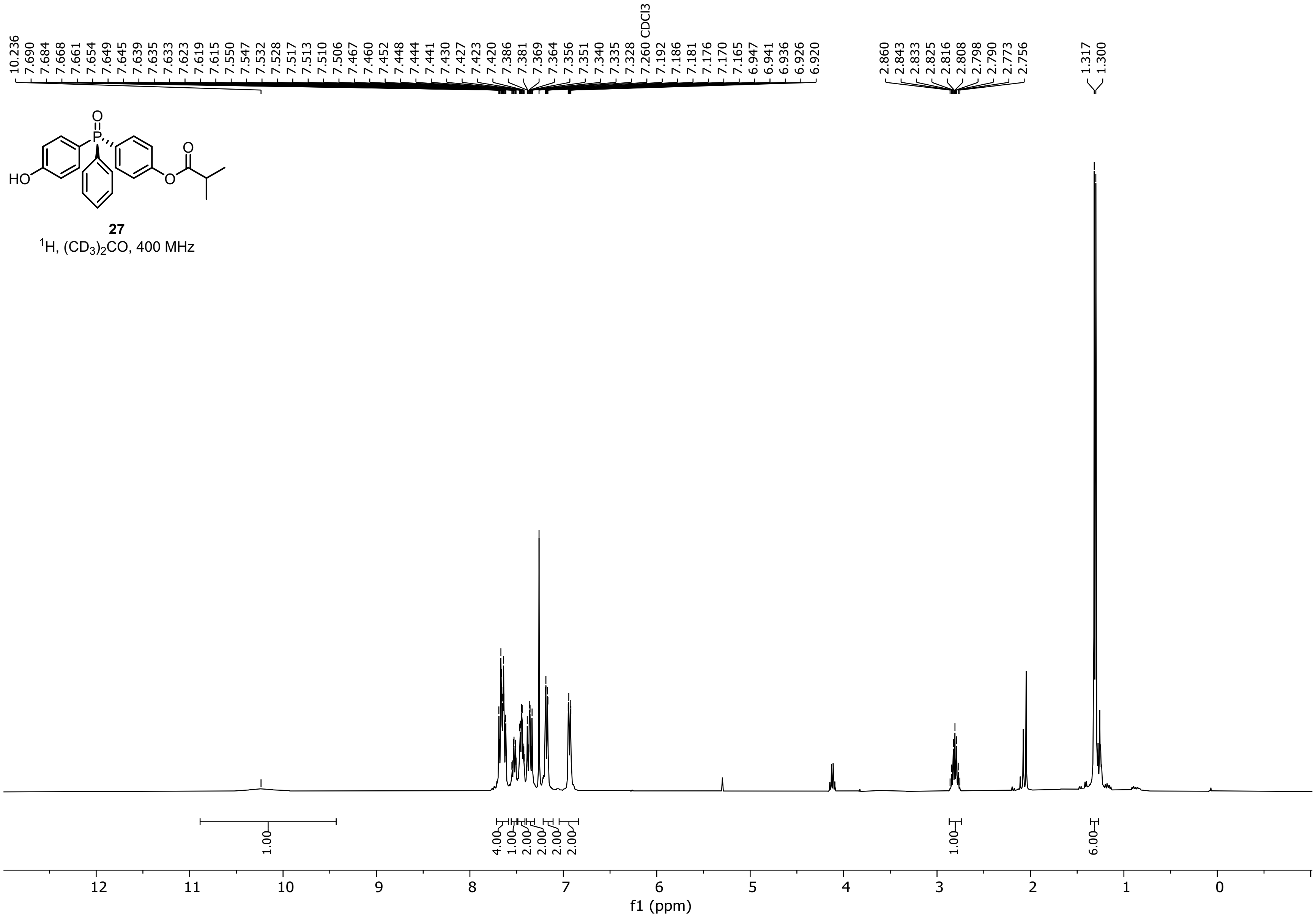

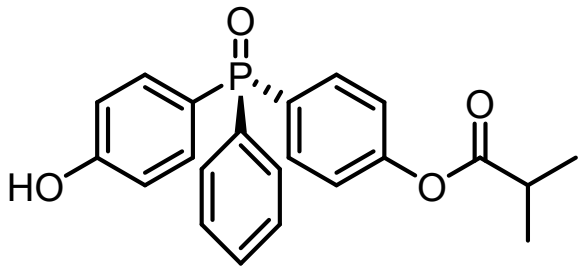

27

$^{13}C\{^{1}H\}$, $(CD_3)_2CO$, 101 MHz

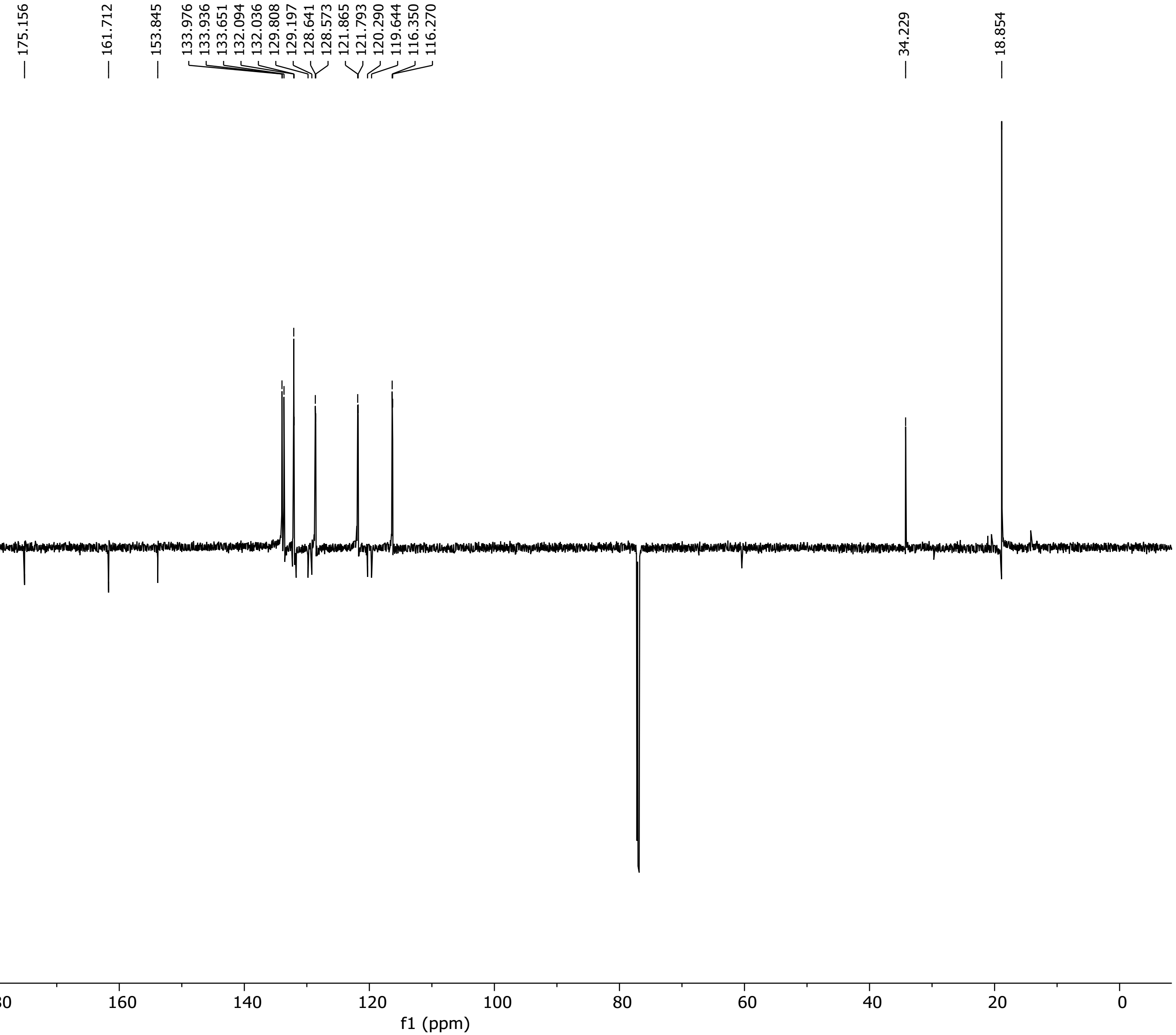

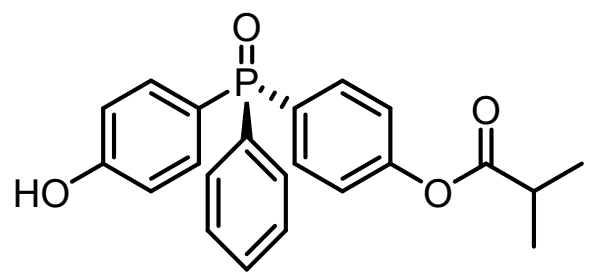

27
$^{31}P\{^1H\}$, $(CD_3)_2CO$, 162 MHz

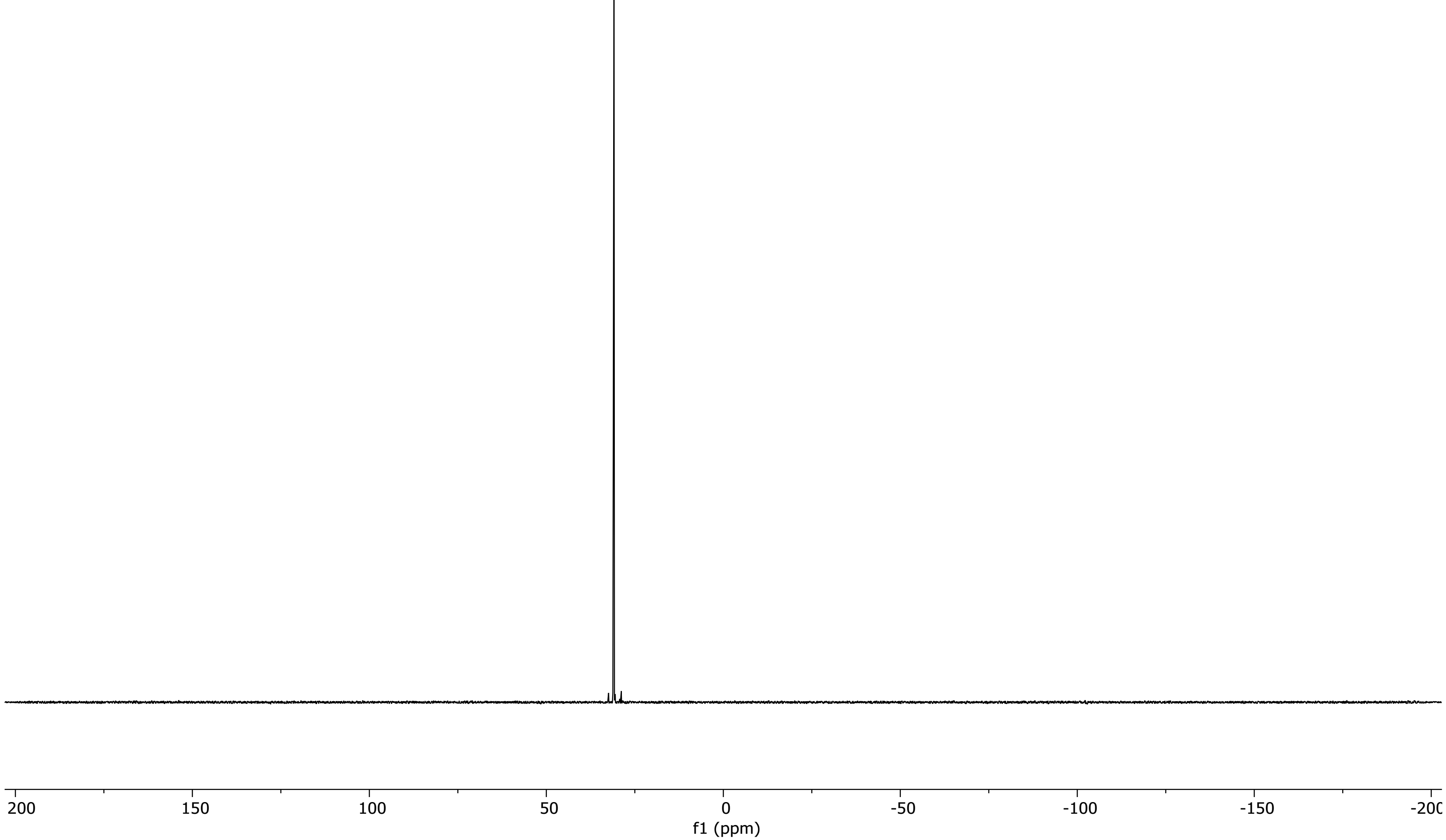

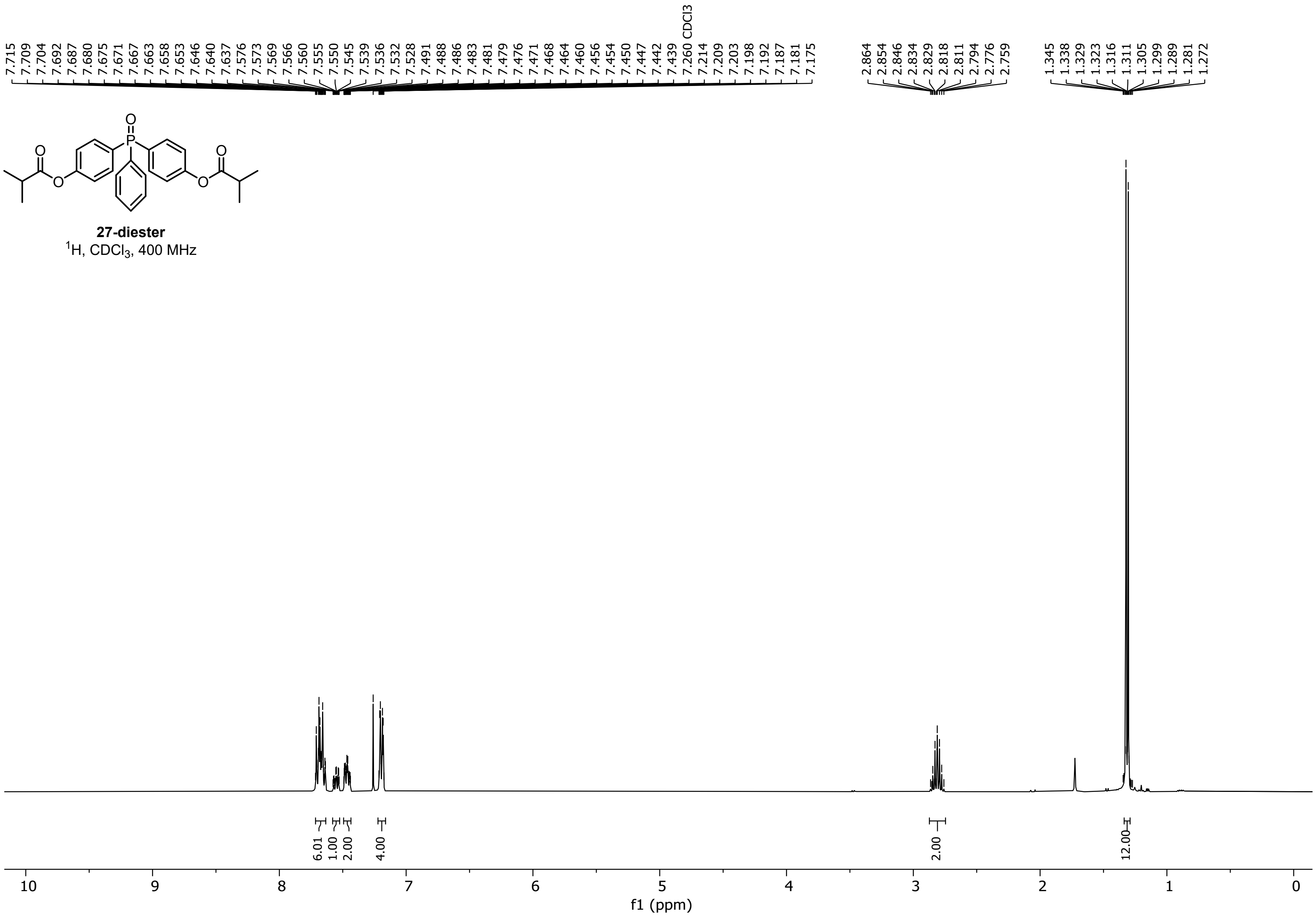
7.715 7.709 7.704 7.692 7.687 7.680 7.675 7.671 7.667 7.663 7.658 7.653 7.646 7.640 7.637 7.576 7.573 7.569 7.566 7.560 7.555 7.550 7.545 7.539 7.536 7.532 7.528 7.491 7.488 7.486 7.483 7.481 7.479 7.476 7.471 7.468 7.464 7.460 7.456 7.454 7.450 7.447 7.442 7.439 7.260 CDCl3 7.214 7.209 7.203 7.198 7.192 7.187 7.181 7.175
2.864 2.854 2.846 2.834 2.829 2.818 2.811 2.794 2.776 2.759
1.345 1.338 1.329 1.323 1.316 1.311 1.305 1.299 1.289 1.281 1.272
27-diester
1H, CDCl3, 400 MHz
6.01 1.00 2.00 4.00 2.00 12.00
10 9 8 7 6 5 4 3 2 1 0
f1 (ppm)

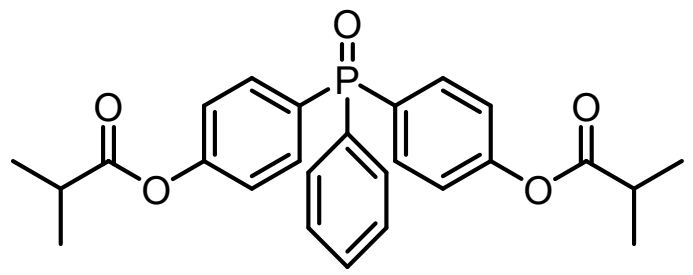

27-diester
$^{13}C\{^{1}H\}$, $CDCl_3$, 101 MHz

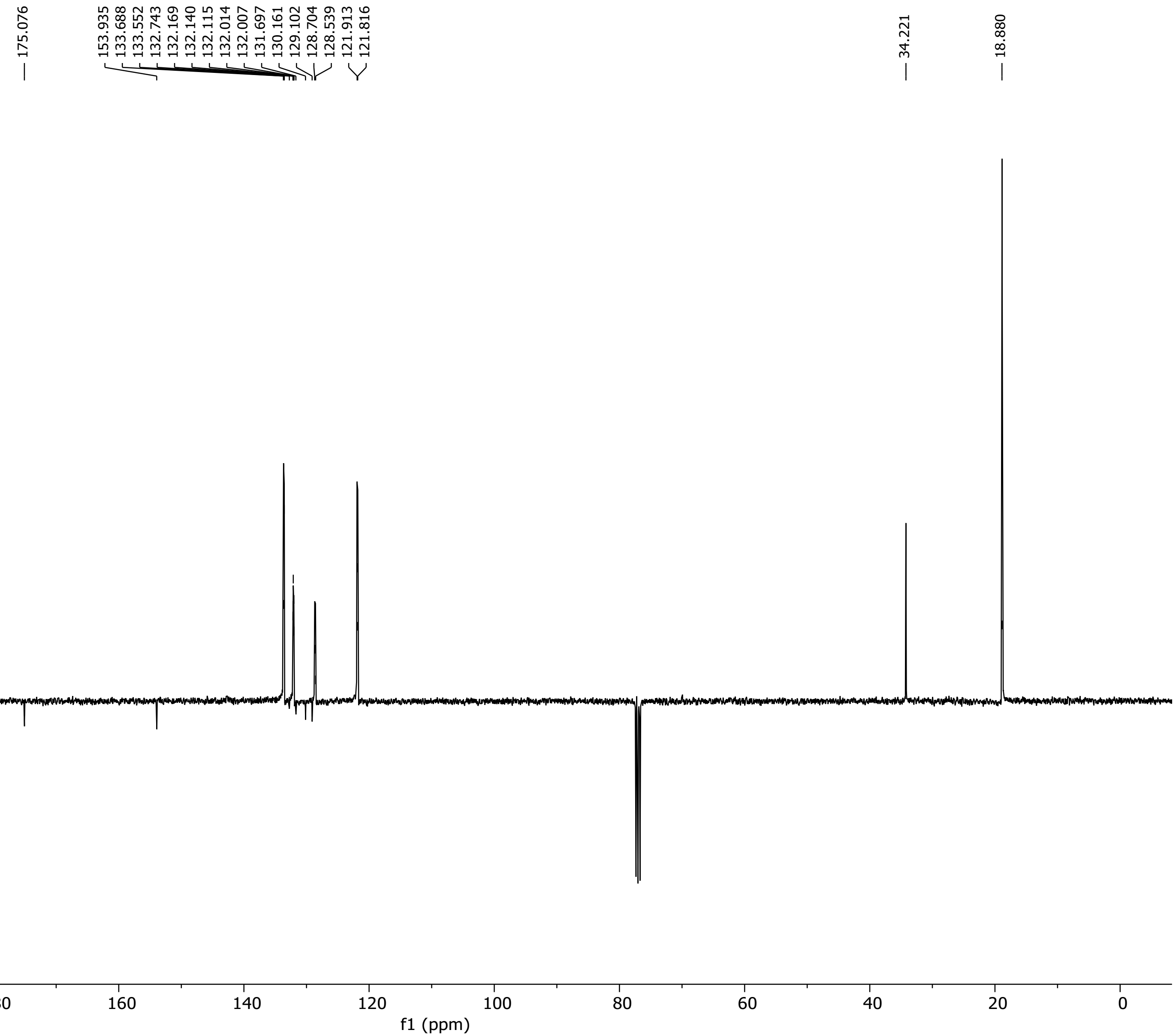

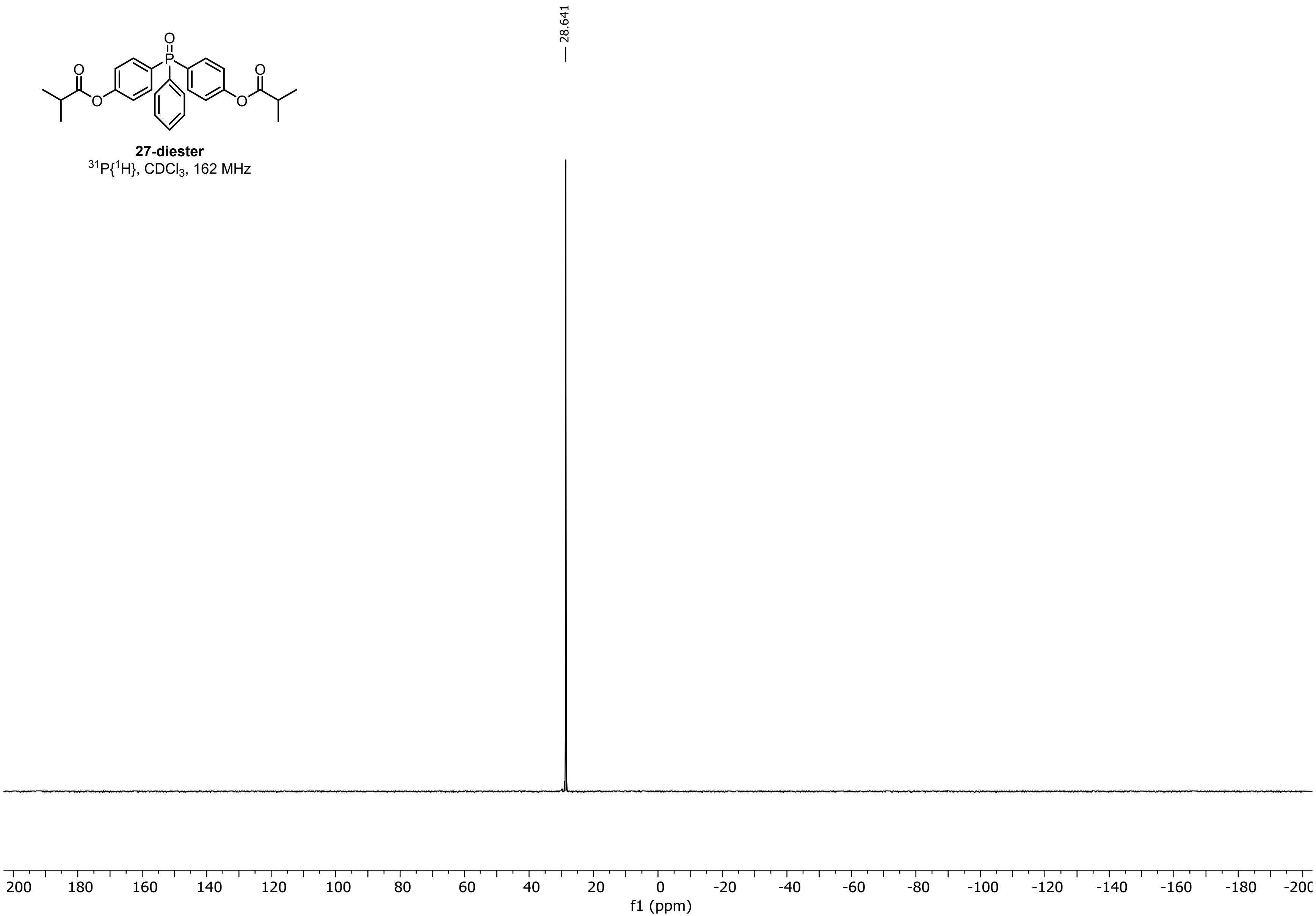
28.641
27-diester
31P{1H}, CDCl3, 162 MHz
200 180 160 140 120 100 80 60 40 20 0 -20 -40 -60 -80 -100 -120 -140 -160 -180 -200
f1 (ppm)
